# Income-related differences in survival across disease categories in Sweden: a retrospective population-based register study

**DOI:** 10.1016/j.lanepe.2026.101896

**Published:** 2026-10-15

**Authors:** Vincent Mougin, Authia Gray, Mohsen Naghavi, Peter Allebeck, Jonas Minet Kinge, Eve E. Wool, Nicole Davis Weaver, Christopher J.L. Murray, Emilie E. Agardh

**Affiliations:** a Department of Global Public Health, Karolinska Institutet, Stockholm, Sweden; b Institute for Health Metrics and Evaluation, University of Washington, Seattle, WA, USA; c Department of Health Management and Health Economics, University of Oslo, Oslo, Norway; d Norwegian Institute of Public Health, Oslo, Norway

**Keywords:** Income inequality, Survival, Non-communicable diseases, Register data, Sweden, Social determinants of health

## Abstract

**Background:**

Lower income is consistently associated with poorer survival after disease diagnosis, even in countries with universal healthcare such as Sweden. While income-related survival differences have been documented for specific conditions, comparative evidence across a range of disease categories with long-term follow-up is lacking. We quantify income-related differences in survival across 100 disease categories focussing on leading non-communicable diseases (NCDs).

**Methods:**

All individuals aged 16–95 years registered in Sweden between 2001 and 2023 were followed from first diagnosis until death or the end of 2023. By disease category cohort, individuals were matched to five general population controls (no prior diagnosis of that category) on birth year, sex, and income quartile. Flexible parametric models were used to estimate standardised survival curves for diagnosed individuals and their matched controls.

**Findings:**

For all 100 disease categories, estimated 10-year survival was lower for the lowest income quartile (Q1) than higher quartiles. For 37 conditions, the Q1 vs Q4 10-year survival gap exceeded 10 percentage points; the largest gaps were for other pharynx cancer (10-year survival 34.2% [95% CI 32.5–35.7] in Q1 vs 58.2% [56.3–60.1] in Q4), larynx cancer, mouth cancer, COPD, and endocarditis. Income-related survival disparities accounted for 6.2 million (95% CI 5.9–6.6 million) person-years of survival loss within 10 years of diagnosis across all cohorts compared to a high-income survival counterfactual, with cardiovascular diseases accounting for the largest share for Q1 (1.3 million). Survival loss after diagnosis relative to undiagnosed controls was significantly greater for Q1 compared to Q4 for 73 of 100 conditions.

**Interpretation:**

Substantial income-related disparities in survival persist in Sweden across all the most prevalent NCDs. Policies addressing income inequality alongside targeted disease-specific interventions are likely required to reduce income disparities in survival.

**Funding:**

Swedish Research Council for Health, Working Life and Welfare.

**Research in context**

**Evidence before this study**

We searched PubMed for studies published between January 1, 2000 and December 31, 2024 using the terms (survival) AND (income OR socioeconomic) AND (Sweden OR register OR population-based) AND (cancer OR cardiovascular). This search identified 992 studies. We screened titles for studies examining survival by income across multiple disease categories and reviewed reference lists of relevant systematic reviews. Existing studies consistently demonstrate that lower income is associated with poorer survival after diagnosis, but nearly all prior research examined single diseases or narrow disease categories in isolation, using heterogeneous methods, follow-up periods, and income measures. Several studies examined survival disparities across multiple cancers, including systematic reviews of socioeconomic inequality in cancer survival in the Nordic countries, but these were limited to cancer and did not extend to cardiovascular diseases, respiratory conditions, or other non-communicable diseases. Large-scale international studies such as CONCORD-3 examined survival across 18 cancers in 71 countries but did not systematically stratify by income within countries. No study has compared survival by income level across a broad range of non-communicable diseases using harmonised methods and consistent long-term follow-up.

**Added value of this study**

This study provides a systematic comparison of survival by income level across 100 disease categories, spanning cancers, cardiovascular diseases, chronic respiratory diseases, neurological disorders, diabetes, kidney diseases, digestive diseases, and mental disorders. Using 23 years of Swedish national register data with near-complete population coverage, we applied harmonised flexible parametric survival models with regression standardisation to enable direct comparison of income-related survival disparities across the full spectrum of major non-communicable diseases. We quantified both per-person survival differences (using 10-year marginal survival probabilities and restricted mean survival times) and population-level burden (person-years lost), and employed matched controls to assess whether disparities exceed baseline differences in the general population. The largest per-person survival disparities by income were found in head and neck cancers (related to smoking, alcohol, and human papillomavirus [HPV]). A pattern that emerged was that diseases with intermediate prognoses such as cardiovascular conditions, chronic obstructive pulmonary disease (COPD), and cirrhosis showed consistently large survival gaps. Highly prevalent cardiovascular conditions such as hypertension and ischaemic heart disease contributed the greatest absolute burden of survival inequality.

**Implications of all the available evidence**

The persistence of substantial income-related survival disparities in Sweden adds to the available evidence that universal healthcare access has not eliminated inequalities in health outcomes by income level. These findings highlight the importance of considering broader social and economic determinants of health alongside healthcare-related factors when seeking to reduce disparities in health outcomes. Our results identify diseases that contribute disproportionately to overall survival inequality and may therefore represent important areas for further investigation and policy attention, though the observational nature of this study does not allow conclusions about the specific mechanisms underlying these differences or the interventions that would be most effective for particular conditions. The systematic approach employed provides a framework for monitoring survival disparities that can be applied in other settings to evaluate the equity implications of healthcare systems and social policies.

## Introduction

Income inequality is a well-documented determinant of health, affecting disease incidence, outcomes, and survival.^1^ Even in high-income countries with universal healthcare systems such as Sweden, individuals with lower incomes experience shorter life expectancy, and evidence suggests this gap is widening in tandem with rising income inequality.^2^ In high-income countries, non-communicable diseases (NCDs) account for most of the disease burden, but the burden is not distributed evenly. In Sweden, research on hospital utilisation has shown that NCDs are more prevalent among low-income populations.^3^ Alongside higher prevalence of NCDs, poorer survival outcomes after diagnosis may contribute to the life expectancy gap between income groups.

Research on survival following disease incidence is extensive, with cancer survival occupying a central role. Examples are numerous and include the large-scale CONCORD-3 study on 18 cancers in 71 countries with up to 20 years of follow-up,^4^ though most cancer studies report 5-year survival. Beyond cancer, survival analyses are also widely applied to cardiovascular diseases,^5^ diabetes and kidney diseases,^6^ and neurological diseases,^7^ as patient survival is considered a primary criterion for effective treatment. When studies examine survival following disease incidence by income level, low-income patients are consistently shown to have reduced survival compared to their high-income counterparts across many major categories of NCDs. In England, for example, survival for different forms of cancer was shown to decrease for individuals living in areas of higher deprivation.^8^ In Korea, low income and inferior insurance were shown to reduce long-term survival following a myocardial infarction diagnosis.^9^ Swedish studies similarly show reduced short-term survival following cardiac arrest^10^ and decreased long-term survival following stroke in low-income individuals.^11^

Despite this large body of evidence, existing studies typically focus on single diseases or disease categories. This limits opportunities for systematic comparisons across diseases, as methods, reference populations, follow-up times, and survival measures differ. Some studies examine only specific patient populations, such as those receiving particular procedures. For example, income has been associated with survival following heart surgery^12^ and pulmonary resection for lung cancer in Sweden.^13^ While of great clinical importance, such studies are difficult to compare with broader population-based survival analyses. Jointly quantifying survival for NCDs by income level allows for comparisons, which can help improve policy by identifying where inequalities are greatest, informing prioritisation in health services, targeting interventions, and ultimately contributing to more equitable health outcomes. To our knowledge, no study has systematically compared survival by income level across a wide variety of NCDs, using harmonised methods and follow-up time. The aim of this study is to quantify survival across 100 disease categories by income level in Sweden using harmonised methods, enabling direct comparisons across disease categories.

## Methods

### Study design and participants

This is a register-based longitudinal study based on individually linked records from: the Swedish Total Population Register (TPR), the National Patient Register (NPR), the Swedish Cause of Death Register (DORS), and the Longitudinal Integrated Database for Health Insurance and Labour Market Studies (LISA). Sweden provides an ideal setting for studying income-related health inequalities due to its national registers with near-complete coverage and minimal missing data, combined with a universal healthcare system where out-of-pocket costs are low or nonexistent, allowing us to study the effects of income beyond direct financial barriers to care. The study population included all registered Swedish residents between the ages of 16 and 95, diagnosed with a disease of interest between January 1st 2001 and December 31st 2023, surviving at least one day after diagnosis and with known income. Records from the inpatient setting for the years 1997–2000 were used to exclude individuals diagnosed prior to the start of the study.

### Procedures

ICD-10 diagnostic codes from the inpatient and specialised outpatient setting were classified into analytic disease categories using mappings from the Global Burden of Diseases, Injuries, and Risk Factors Study (GBD) multiple/contributing cause framework. A cohort was created for each disease category, with the date of first diagnosis representing entry into the cohort. We produced estimates for 100 disease categories, encompassing cancers, cardiovascular diseases, chronic respiratory diseases, neurological disorders, diabetes and kidney diseases, digestive diseases, musculoskeletal disorders, skin and subcutaneous diseases, substance use disorders, sense organ diseases, mental disorders, additional NCDs from other groups, HIV, tuberculosis, and self-harm. Further details on disease selection, classification, and the registers used are presented in the appendix (p 1; Appendix Table S1). Multimorbidity was common given the prevalence of diseases analysed (Appendix Table S2).

### Income and sociodemographic factors

We classified individuals into income quartiles using consumption-equivalized family disposable income, defined as total family income after tax adjusted for family size. For each calendar year, we ranked individuals by the average of the five preceding years of this income measure and sorted them into quartiles within sex and birth-year strata. Five-year lagged income was used to reduce short-term income volatility and minimise the influence of disease-related changes in income before diagnosis. Annual income values were inflation-adjusted to 2024 Swedish kronor, and individuals with income below the 2nd percentile were excluded because this group largely comprised persons with zero, negative, or implausibly low reported incomes. See Appendix for details (pp 2 and 3).

### Outcomes

All measures were estimated separately for each diagnosis. The primary measures of income-related survival inequality were marginal 10-year survival probabilities and 10-year restricted mean survival time (RMST; the area under the marginal survival curve up to ten years). Median survival time, 10-year survival probabilities, and 10-year RMST from Kaplan–Meier estimates are reported as descriptive statistics. 10-year risk ratios (RR) between the diagnosed and matched general population cohorts are presented, which are adjusted for age, sex, and income level by the matching design. The income-associated survival gap among diagnosed individuals is quantified as the difference in 10-year RMST between the highest income quartile (Q4) and the lower three quartiles (Q1–Q3), expressed as person-years. We contrast the survival loss associated with diagnosis (the difference in 10-year RMST between diagnosed and matched general population controls) between Q1 and Q4. This difference-in-differences quantifies the extent to which the survival loss following diagnosis varies by income level, net of background income differences in survival.

### Statistical analysis

The primary results were estimated using flexible parametric survival models, which directly estimate the baseline hazard function and model time-varying effects smoothly without assuming proportional hazards, enabling regression standardisation and adjusted absolute measures. The models used restricted cubic splines to capture the baseline hazard function and to model non-linear effects of age and calendar year. For these models, income level was categorised into quartiles, with the middle quartiles (2nd and 3rd) combined as the reference group to contrast extremes while maintaining adequate sample size in the reference group. We included interactions between the income variable and both age and calendar year to allow income gradients to vary across these dimensions. We additionally adjusted for sex with income–sex interactions (except in the case of sex-specific diseases). To assess survival loss by disease compared to the general population, we randomly matched those diagnosed to five individuals with no history of the disease by birth year, sex, and income quartile. We fitted separate models for diagnosed individuals and their matched general population controls. See Appendix for details (pp 4–6).

From the fitted models, we generated standardised (or marginal) survival curves by setting income to a specific level (eg, Q1) for all individuals while retaining their actual values for age, sex, and diagnosis year, then averaging the predicted survival probabilities. This was repeated for each income category. This approach estimates the survival that would be observed if the entire cohort had a given income level, enabling direct comparison across income groups with identical distributions of confounders. Models were fitted using up to 23 years of available follow-up, with predictions extending to 10 years post-diagnosis. For sex-restricted diagnoses (cervical cancer, uterine cancer, and testicular cancer), data were restricted to the relevant sex, and the sex variable was dropped from the model. The marginal survival curves were integrated to obtain 10-year RMST, defined as the average number of years lived within 10 years of diagnosis.^14^ Uncertainty intervals (95%) for both survival probabilities and RMST were obtained via parametric bootstrap with 1000 replications, sampling from the multivariate normal distribution of the estimated regression coefficients; confidence intervals were calculated as the 2.5th and 97.5th percentiles of the bootstrap distribution. Estimates were robust in sensitivity analyses excluding patients with less than 30 days of follow-up and restricting to diagnoses from 2005 onward (Appendix pp. 8 and 9).

To estimate the income-associated survival gap among diagnosed individuals in person-years, we computed the difference in 10-year RMST between Q1 and Q4 and Q2–Q3 and Q4, then multiplied these differences by the number of Q1 and Q2–Q3 individuals respectively. Confidence intervals for person-years lost within each disease category were calculated assuming independence between income groups. We aggregated the differences across all disease categories, excluding subcauses to avoid double-counting. This measure reflects total disparities between diagnosed cohorts rather than survival loss among unique individuals, and to accommodate the positive correlation induced by individuals appearing in multiple disease cohorts confidence bounds were summed directly, a conservative approach that produces wider intervals. We then calculated disease-related survival loss, defined as the difference in 10-year RMST between the diagnosed and matched general population, and contrasted this measure for Q1, Q2-Q3, and Q4. See Appendix for details (p. 6).

We used python (version 3.13.12) for data processing, R (version 4.5.2) for statistical modelling, and matplotlib for figures.

### Ethics approval

The authors assert that all procedures contributing to this work comply with the ethical standards of the relevant national and institutional committees on human experimentation and with the Helsinki Declaration of 1975, as revised in 2008. The registration number for the ethical approvals from the Swedish Ethical Review Authority; DNR: 2018/1339-31/5, 2018/2292-32, 2019-02185, 2021-00657 and 2022-03111-02, 2023-07509-02.

### Role of the funding source

The funder of the study had no role in study design, data collection, data analysis, interpretation, writing of the report, or decision to publish.

## Results

The population eligible for inclusion comprised 12,167,578 individuals aged 16–95 years registered in Sweden between January 1, 2001 and December 31, 2023 (Fig. 1). 7,192,290 unique individuals had a recorded diagnosis of at least one disease category of interest during the observation period, and there were 31,520,218 cohort entries across all disease categories. The number of cohort entries exceeds the number of unique individuals because the same person may contribute to multiple disease cohorts. This reflects multimorbidity and the inclusion of subcategories for certain causes (ischaemic heart disease, stroke, dementia, diabetes, inflammatory bowel disease, drug use disorders, and depressive disorders). Of the cohort entries, 2,077,773 were excluded due to having missing or unreasonably low average income. A further 2211 cohort entries were excluded due to unavailability of matched controls. Finally, 1,401,461 cohort entries were excluded due to having zero follow-up time because the first recorded diagnosis was on the same day as their death. The final analysis sample comprised 6,849,397 unique individuals, 28,038,773 cohort entries, and 196,882,327 person-years.

**Fig. 1:**
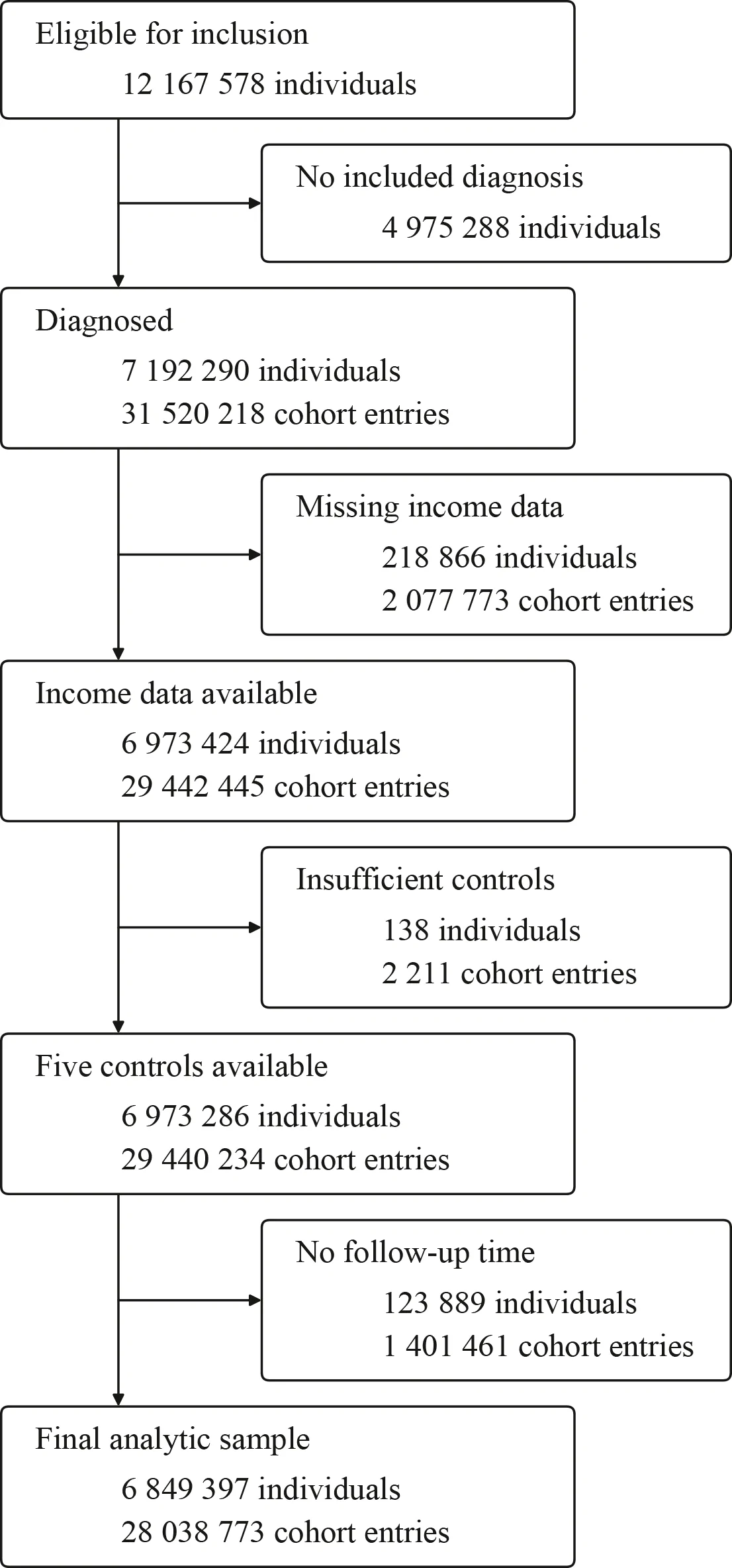
**Derivation of the study population.** Flow through the inclusion and exclusion criteria applied to derive the analytic cohort from Swedish population registers.

Income distribution varied across disease cohorts. Table 1 presents the mean age and sex distribution of each disease cohort and the proportions of individuals in the lowest and highest income quartiles. Summary statistics stratified by income quartile are presented in Appendix Table S3. The disease cohort with the highest proportion of low-income individuals was schizophrenia, with 62.1% of the cohort made up of individuals in the bottom income quartile. The disease cohort with the highest proportion of high-income individuals was melanoma, with 32.1% of that cohort in the top income quartile. The disease cohort with the largest age difference by income quartile was cirrhosis: mean age of diagnosis for Q1 (low) was 50.0 years and mean age of diagnosis for Q4 (high) was 58.1 years. The largest analytic diagnosis cohort was hypertension, with 2,023,132 individuals, and the smallest was nasopharynx cancer, with 1728 individuals.

**Table 1:** Characteristics of the diagnosed cohorts, Sweden, 2001–2023.

Group	Diagnosis	Count	PY	% Q1	% Q4	% Female	Mean age	10 y Surv % (95% CI)
Cancers	Bladder cancer	71,838	427,958	25.4	24.0	25.7	73.2	41.66 (41.24–42.08)
Cancers	Bone & cartilage cancer	7832	39,885	26.2	24.1	44.5	63.3	31.61 (30.5–32.72)
Cancers	Brain cancer	28,259	100,210	24.2	25.3	45.7	62.3	21.7 (21.17–22.22)
Cancers	Breast cancer	189,557	1,652,690	22.7	27.6	98.4	64.2	68.2 (67.96–68.44)
Cancers	Cervical cancer	15,595	127,327	27.8	22.8	99.9	55.6	61.58 (60.74–62.41)
Cancers	Colorectal cancer	158,366	816,928	25.4	24.1	48.8	72.1	36.79 (36.51–37.06)
Cancers	Esophageal cancer	15,232	30,191	30.3	20.4	27.8	71.1	10.35 (9.8–10.93)
Cancers	Gallbladder cancer	17,199	27,906	27.5	22.3	57.7	72.5	8.56 (8.08–9.06)
Cancers	Hodgkin lymphoma	8346	76,694	25.2	26.0	45.6	52.6	66.98 (65.86–68.07)
Cancers	Kidney cancer	39,633	205,590	26.9	22.8	38.6	70.2	38.12 (37.56–38.68)
Cancers	Larynx cancer	5702	35,727	36.9	17.8	21.4	68.8	37.83 (36.43–39.23)
Cancers	Leukemia	44,601	235,656	24.5	25.7	42.4	69.1	35.89 (35.38–36.4)
Cancers	Lip oral cavity cancer	29,800	194,581	26.7	24.3	48.6	68.4	47.89 (47.24–48.55)
Cancers	Liver cancer	16,240	31,243	35.2	19.1	35.1	69.5	11.09 (10.5–11.7)
Cancers	Lung cancer	108,917	226,771	31.4	18.7	50.4	71.6	11.16 (10.94–11.39)
Cancers	Lymphoma	50,944	316,602	24.5	25.5	44.5	67.9	44.65 (44.15–45.14)
Cancers	Melanoma	93,519	673,408	17.1	32.1	49.3	64.8	65.61 (65.24–65.98)
Cancers	Myeloma	29,132	134,740	23.9	27.4	43.5	71.5	28.0 (27.37–28.64)
Cancers	Nasopharynx cancer	1728	11,248	32.4	19.5	37.8	62.4	42.82 (40.26–45.36)
Cancers	Other pharynx cancer	10,698	64,264	31.0	22.1	31.7	64.5	45.48 (44.41–46.56)
Cancers	Ovarian cancer	26,798	157,313	24.3	25.5	99.9	65.3	35.68 (35.05–36.31)
Cancers	Pancreatic cancer	46,014	61,356	26.1	23.9	51.4	72.6	6.88 (6.61–7.17)
Cancers	Prostate cancer	253,328	1,798,152	20.7	28.7	0.1	71.6	53.8 (53.57–54.02)
Cancers	Soft tissue sarcoma	18,536	111,545	23.5	27.3	52.6	64.4	43.48 (42.67–44.28)
Cancers	Stomach cancer	26,047	71,745	28.5	21.2	37.1	71.9	16.34 (15.83–16.86)
Cancers	Testicular cancer	10,313	112,889	20.7	27.5	0.1	41.6	88.04 (87.34–88.71)
Cancers	Thyroid cancer	14,590	122,058	26.5	24.3	71.5	56.2	72.32 (71.48–73.15)
Cancers	Uterine cancer	39,372	305,778	26.1	24.4	99.9	69.2	57.22 (56.67–57.76)
Cardiovascular	Aortic aneurysm	110,830	622,363	26.6	21.6	28.0	71.8	42.57 (42.21–42.92)
Cardiovascular	Atrial fibrillation	814,748	4,810,247	25.9	24.1	45.0	75.1	39.79 (39.67–39.92)
Cardiovascular	Cardiomyopathy	85,014	610,789	28.2	22.7	35.3	60.3	60.83 (60.44–61.23)
Cardiovascular	Endocarditis	23,046	118,033	31.6	21.2	36.5	69.0	36.34 (35.61–37.06)
Cardiovascular	Heart failure	708,074	2,943,435	30.0	20.3	47.7	78.1	21.59 (21.47–21.7)
Cardiovascular	Hypertension	2,023,132	15,712,722	26.7	22.9	50.3	68.9	60.45 (60.37–60.53)
Cardiovascular	Hypertensive heart disease	69,273	543,564	28.5	21.4	44.6	72.5	44.82 (44.42–45.21)
Cardiovascular	Ischemic heart disease	812,154	6,033,257	28.5	21.1	41.9	71.8	50.16 (50.04–50.28)
Cardiovascular	Acute myocardial infarction	451,526	2,825,484	29.6	19.8	39.6	73.4	42.3 (42.14–42.46)
Cardiovascular	Angina	446,351	3,889,011	28.1	21.2	41.6	71.3	56.21 (56.05–56.37)
Cardiovascular	Chronic ischemic heart disease	609,891	4,366,146	28.9	20.5	35.6	72.4	48.7 (48.56–48.85)
Cardiovascular	Nonrheum valv diseases	272,699	1,685,814	25.7	24.0	46.6	72.9	44.67 (44.44 - 44.89)
Cardiovascular	PAH	13,422	40,424	31.5	18.7	58.6	76.1	15.99 (15.2–16.8)
Cardiovascular	Peripheral artery	193,190	1,129,782	30.1	19.3	49.0	73.2	35.55 (35.31–35.8)
Cardiovascular	Rheumatic heart disease	31,613	154,301	28.8	23.2	57.2	73.7	36.51 (35.84–37.19)
Cardiovascular	Stroke	781,926	4,875,371	27.8	22.8	49.0	73.2	42.14 (42.02–42.27)
Cardiovascular	Intracerebral hem	95,308	443,131	30.2	21.7	44.7	72.2	30.19 (29.85–30.52)
Cardiovascular	Ischemic stroke	661,949	4,173,350	27.5	23.0	49.0	73.7	42.58 (42.44–42.72)
Cardiovascular	Subarachnoid hem	75,556	465,076	28.1	23.1	45.4	68.8	45.47 (45.06–45.87)
Chronic respiratory	Asthma	400,545	3,939,215	27.3	22.7	61.3	53.2	78.65 (78.51–78.79)
Chronic respiratory	COPD	373,459	2,275,492	34.4	17.1	51.9	71.2	39.0 (38.82–39.18)
Chronic respiratory	Interstitial lung disease	66,040	429,691	26.1	22.6	41.8	66.0	48.56 (48.12–49.0)
Chronic respiratory	Pneumoconiosis	7985	58,151	24.5	17.5	8.5	73.6	40.06 (38.91–41.2)
Chronic respiratory	Sleep Apnea	293,509	2,725,405	24.5	23.9	30.1	56.1	87.03 (86.88–87.17)
Diabetes & CKD	Acute glomerulonephritis	4245	43,581	25.1	25.4	47.8	53.3	76.26 (74.84–77.61)
Diabetes & CKD	Chronic kidney disease	456,498	1,913,543	30.6	20.5	41.1	73.5	28.87 (28.71–29.03)
Diabetes & CKD	Diabetes	749,652	6,019,606	32.3	18.7	43.5	66.1	59.44 (59.31–59.57)
Diabetes & CKD	Diabetes type 1	180,139	1,756,646	31.3	19.3	43.2	61.8	56.53 (56.28–56.77)
Diabetes & CKD	Diabetes type 2	697,339	5,249,549	33.1	18.0	43.3	67.6	56.11 (55.97–56.24)
Digestive	Cirrhosis	158,425	1,372,857	43.2	15.6	42.6	53.4	65.74 (65.48–66.0)
Digestive	Diverticular disease	376,321	2,931,086	23.8	24.6	56.5	67.2	68.16 (67.98–68.34)
Digestive	Gallbladder & biliary	499,559	4,549,039	28.7	20.7	62.5	56.9	76.02 (75.88–76.15)
Digestive	Inflammatory bowel	235,177	2,365,227	25.5	23.7	56.2	52.8	76.01 (75.82–76.2)
Digestive	Crohn's disease	53,613	584,516	25.5	23.6	51.9	46.2	86.01 (85.67–86.33)
Digestive	Ulcerative colitis	207,932	2,059,266	25.4	23.8	56.7	53.6	74.81 (74.61–75.02)
Digestive	Pancreatitis	110,236	794,498	30.6	20.3	48.6	62.0	62.47 (62.13–62.81)
Digestive	Upper digest diseases	914,898	8,585,912	31.0	20.3	54.4	57.0	73.3 (73.2–73.4)
Digestive	Vascular intestinal	31,430	138,242	30.2	20.1	54.0	72.8	34.16 (33.51–34.82)
Mental & substance use	ADHD	198,591	1,424,289	37.2	14.7	51.5	30.1	96.0 (95.88–96.12)
Mental & substance use	Alcohol use disorders	369,308	3,434,020	34.4	18.6	35.7	44.1	79.54 (79.39–79.69)
Mental & substance use	Anorexia nervosa	17,392	170,677	22.2	27.3	93.8	24.5	96.05 (95.69–96.38)
Mental & substance use	Anxiety disorders	912,440	8,614,282	34.0	18.2	61.3	41.1	87.61 (87.53–87.68)
Mental & substance use	Bipolar disorder	105,060	1,042,827	36.3	15.8	62.1	41.4	87.3 (87.07–87.53)
Mental & substance use	Depressive disorders	690,423	6,699,939	33.2	18.2	59.7	44.7	80.87 (80.77–80.97)
Mental & substance use	Dysthymia	29,346	312,722	41.0	14.4	55.4	41.2	89.69 (89.28–90.07)
Mental & substance use	Major depression	683,332	6,602,547	33.1	18.3	59.8	44.8	80.74 (80.64–80.84)
Mental & substance use	Drug use disorders	183,777	1,674,435	43.8	13.2	39.5	36.5	83.58 (83.38–83.78)
Mental & substance use	Opioid use disorders	45,670	380,322	51.6	10.4	39.1	40.2	76.8 (76.35–77.26)
Mental & substance use	Schizophrenia	41,120	466,518	62.1	6.2	45.3	46.7	77.15 (76.71–77.58)
Mental & substance use	Self-harm	136,115	1,373,186	41.4	13.5	56.4	38.2	82.43 (82.2–82.65)
Musculoskeletal	Gout	134,140	782,222	27.1	22.6	30.6	71.9	42.42 (42.1–42.74)
Musculoskeletal	Low back pain	567,352	5,703,096	29.2	22.7	53.6	53.2	83.37 (83.26–83.48)
Musculoskeletal	Lupus	17,427	198,758	27.1	23.3	80.2	53.2	80.03 (79.37–80.67)
Musculoskeletal	Neck pain	249,816	2,467,284	28.3	22.4	56.2	50.2	90.07 (89.93–90.2)
Musculoskeletal	Osteoarthritis	844,730	8,179,587	22.3	26.4	56.7	64.8	79.1 (79.0–79.2)
Musculoskeletal	Rheumatoid arthritis	122,790	1,253,520	25.8	22.8	68.8	61.0	72.82 (72.54–73.09)
Neurological	Alzheimer's & other dementias	374,264	1,175,121	27.0	23.7	58.5	81.9	7.64 (7.54–7.75)
Neurological	Alzheimer's disease	133,400	516,576	22.0	28.5	60.1	79.9	11.17 (10.96–11.39)
Neurological	Dementia as a sign with others	119,384	470,725	21.7	29.1	59.0	79.4	11.22 (11.0–11.45)
Neurological	Unspecified dementia	226,475	569,664	28.9	21.9	59.9	83.6	4.16 (4.06–4.26)
Neurological	Vascular dementia	78,849	236,992	27.3	23.2	51.6	81.6	6.49 (6.28–6.7)
Neurological	Idiopathic epilepsy	148,081	1,245,506	33.2	20.2	46.7	57.4	57.05 (56.78–57.33)
Neurological	Motor neuron disease	10,196	36,388	23.5	26.0	41.8	67.0	18.36 (17.54–19.2)
Neurological	Multiple sclerosis	27,843	338,448	24.4	25.1	68.4	45.2	89.3 (88.89 - 89.69)
Neurological	Parkinson's disease	65,735	382,753	23.7	27.2	40.4	73.6	33.17 (32.74–33.6)
Other	Decubitus ulcer	76,832	233,339	33.6	20.0	52.4	76.0	16.67 (16.34–17.0)
Other	Endo/metab/blood/immune	1,693,901	14,362,618	27.5	23.3	60.0	58.6	72.09 (72.01–72.17)
Other	HIV/AIDS	43,671	488,722	27.8	25.0	47.0	43.1	86.39 (86.04–86.73)
Other	Hemoglobinopathies	638,150	3,100,659	31.0	21.2	55.9	69.7	34.89 (34.75–35.02)
Other	Psoriasis	233,026	2,552,075	24.3	24.0	52.5	53.0	84.98 (84.82–85.14)
Other	Tuberculosis	40,699	418,623	46.9	15.6	54.9	49.3	78.71 (78.28–79.13)
Other	UTI & interstitial nephritis	1,189,438	8,569,611	29.0	22.8	62.4	63.6	52.96 (52.86–53.06)

Diseases categories sorted by group. Count is the number of diagnosed individuals contributing to that cohort and person-years the total observed follow-up time. % Q1 and % Q4 give the share of the cohort in the lowest and highest income quartiles. Mean age is age at first recorded diagnosis. Ten-year survival is an unadjusted Kaplan–Meier estimate with a 95% CI, pooled across income quartiles and both sexes, measured from date of diagnosis.

With the exception of osteoarthritis, diagnosis was associated with higher mortality across all disease categories compared to general population controls (Appendix Table S4). Opioid use disorder had the highest mortality risk ratio, followed by self-harm and brain cancer, reflecting the poor prognoses and younger mean diagnosis age for these causes. The modest protective association found for osteoarthritis likely reflects detection bias among healthier individuals seeking elective care. Unadjusted survival estimates by income group are presented in Appendix Table S3, but should be interpreted cautiously given demographic differences between income groups; age- and sex-stratified estimates are reported in Appendix Tables S5–S7. Median survival time after diagnosis varied widely by disease (Fig. 2). Six aggressive cancers had median survival times under one year: pancreas cancer, gallbladder cancer, liver cancer, lung cancer, oesophageal cancer, and stomach cancer. For 30 of 100 disease categories, median survival time exceeded 23 years and was not reported in Fig. 2.

**Fig. 2:**
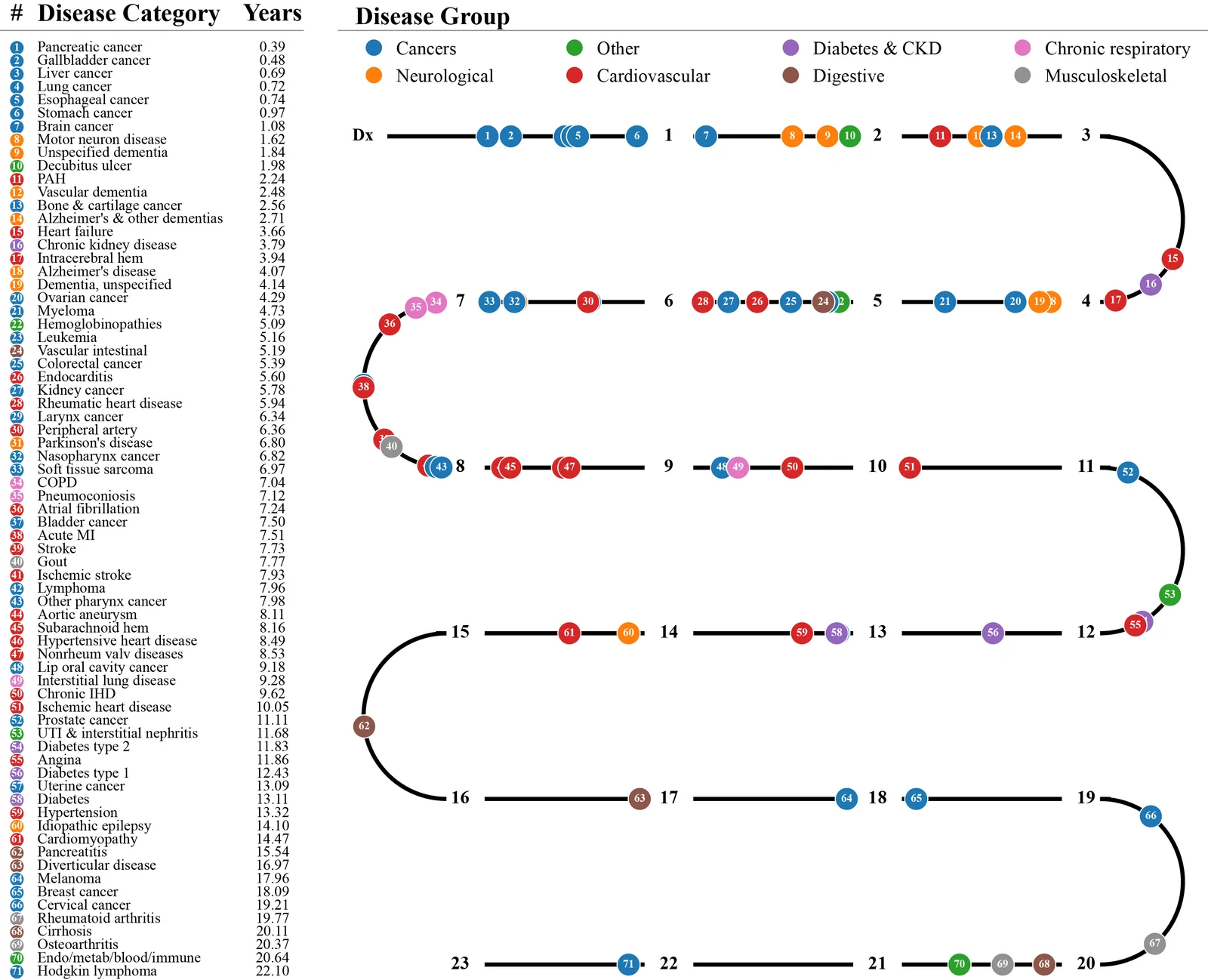
**Timeline of median survival after diagnosis by disease category.** Years from diagnosis until the Kaplan–Meier survival probability of the diagnosed cohort falls to 50%, plotted along a curved timeline. Dx marks the date of diagnosis. Each numbered marker is one disease category, coloured by disease group, with median survival time listed in the legend. Estimates are unadjusted, pooled across income quartiles, both sexes and all ages at diagnosis, for diagnoses recorded 2001–2023. Only conditions whose survival curve reached 50% within the observation period appear here.

After standardising for differences in age, sex, and diagnosis year between income groups, individuals in the bottom income quartile (Q1) had lower 10-year survival probabilities than those in the top quartile (Q4) for all 100 disease categories analysed (Fig. 3). The largest disparities were observed for smoking- and alcohol-related conditions. Other pharynx cancer exhibited the greatest difference: 10-year marginal survival was 34.2% (95% CI: 32.5–35.7) in Q1 compared to 58.2% (95% CI: 56.3–60.1) in Q4. Larynx cancer, lip and oral cavity cancer, chronic obstructive pulmonary disease (COPD), endocarditis, and aortic aneurysm also showed differences exceeding 14 percentage points. In general, survival differences by income tended to be larger for diseases with moderate 10-year survival rather than those with very high or very low survival (Appendix Fig. S1). All disease groups analysed contained at least one disease with 10-year survival differences exceeding 8 percentage points between Q1 and Q4.

**Fig. 3:**
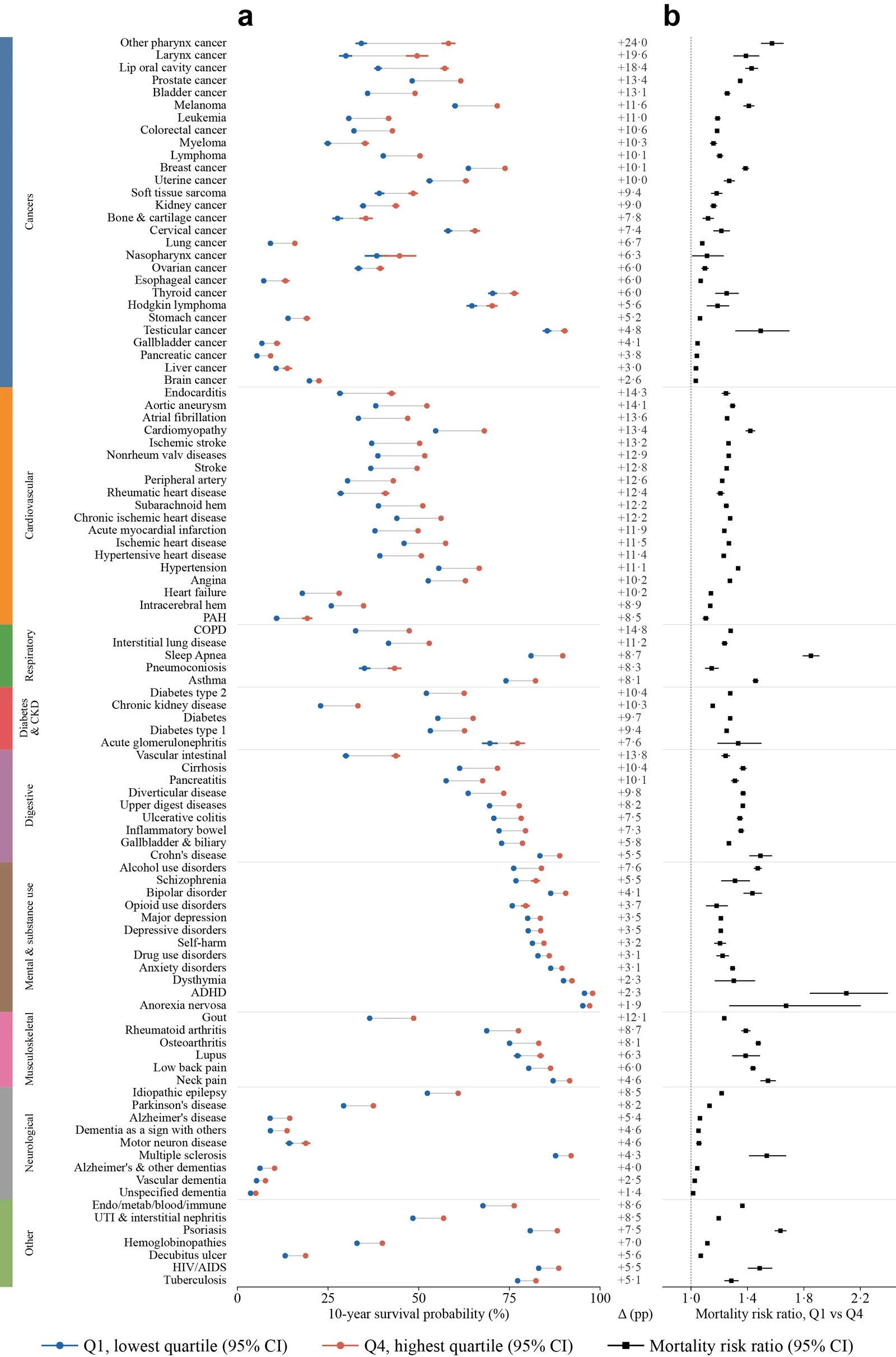
**Income-related differences in 10-year survival across 100 disease categories.** (a) Marginal 10-year survival probability with 95% CIs for the lowest (Q1) and highest (Q4) income quartiles; the difference column reports the absolute difference in percentage points (pp). (b) Mortality risk ratio at 10 years, Q1 vs Q4, with 95% CIs; the dashed line marks no difference. Conditions are grouped by disease category and ordered within category by the size of the survival difference.

The divergence in survival between income quartiles in the years following diagnosis showed different trends by disease: for some diagnosis categories such as other pharynx cancer or vascular intestinal disease, the low-income and high-income diagnosed populations diverged early in their survival probabilities, whereas for others like type 2 diabetes and alcohol use disorders the divergence occurred progressively. Estimated marginal survival curves are presented for the disease categories with the largest 10-year survival differences within each disease group in Fig. 4. The survival curves of the general population matches also show a clear income difference in survival, as well as considerable variation by cause due to sharing the age, sex, and income composition of the diagnosed groups.

**Fig. 4:**
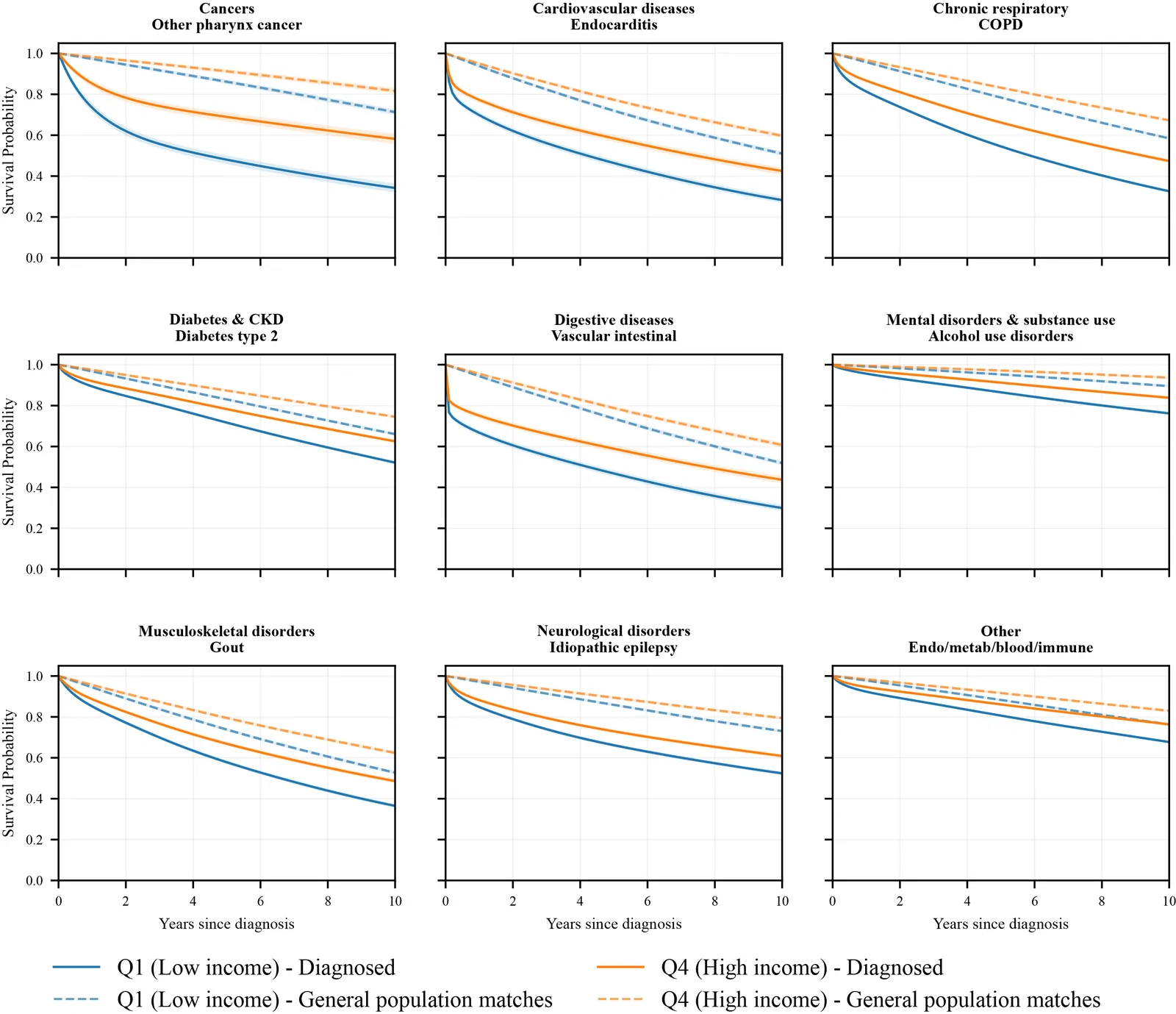
**Survival curves for lowest and highest income quartiles and matched controls.** Model-estimated marginal survival curves over the ten years following diagnosis for the diseases with the largest income-related survival difference at ten years within each disease group. Solid lines for diagnosed individuals and dashed lines for matched undiagnosed comparators; blue denotes the lowest income quartile (Q1) and orange the highest (Q4). Shaded bands are 95% CIs.

To quantify the magnitude of survival disparities in a clinically interpretable metric, we calculated the 10-year restricted mean survival time (RMST) for each income group (Appendix Table S8). The diseases with the largest RMST differences between high-income (Q4) and low-income (Q1) individuals were other pharynx cancer (1.90 years), larynx cancer (1.46 years), lip and oral cavity cancer (1.31 years), vascular intestinal disorders (1.15 years), endocarditis (1.12 years), COPD (1.06 years), and aortic aneurysm (0.97 years). The 95% confidence interval for the RMST difference between Q1 and Q4 excluded zero for all 100 disease categories.

To quantify the population burden of income-related survival disparities, we calculated the total person-years of survival lost among low-income (Q1) individuals and middle-income individuals (Q2–Q3) compared to a counterfactual scenario where they experienced high-income (Q4) survival patterns (Appendix Table S8). We estimated that approximately 6.2 million person-years of survival were lost within 10 years of diagnosis compared to the counterfactual scenario where low- and middle-income diagnosed individuals experienced high-income survival trajectories for the diseases analysed (Appendix Table S8). The diseases contributing the largest absolute burden of person-years lost for the lowest income quartile (Q1) were: hypertension (352,494 person-years), endocrine/metabolic/blood/immune disorders (244,265 person-years), urinary tract infections and interstitial nephritis (206,089 person-years), atrial fibrillation (197,145 person-years), stroke (193,196 person-years) and heart failure (181,085 person-years). For low-income individuals, the cardiovascular diseases group accounted for the largest share of person-years lost (1.3 million person-years, 37% of the burden), followed by other NCDs, digestive diseases, and diabetes and chronic kidney disease.

To assess whether income-related survival differences persisted after accounting for background mortality, we calculated disease-related survival loss as the difference in 10-year restricted mean survival time (RMST) between diagnosed individuals and their matched general population controls. We then compared this measure across income groups (Q1, Q2–Q3, and Q4) (Fig. 5). This framework is conceptually aligned with relative survival approaches used in cancer epidemiology to quantify excess mortality,^15^ but replaces life-table expected survival with individually matched undiagnosed controls, allowing application across disease categories where standard relative survival infrastructure is limited. Fig. 5 presents the deviation in disease-related survival loss relative to the middle-income reference (Q2–Q3 combined). For 73 disease categories, Q1 experienced greater disease-related survival loss after diagnosis than Q4, with non-overlapping 95% CI. Cancers of the head and neck showed the largest differences in disease-related survival loss; in general the disease categories with the greatest overall survival gaps by income tended to also have the greatest gaps after netting out background mortality differences. Two diagnoses (osteoarthritis and unspecified dementia) showed reversed patterns, likely reflecting age-related selection effects.

**Fig. 5:**
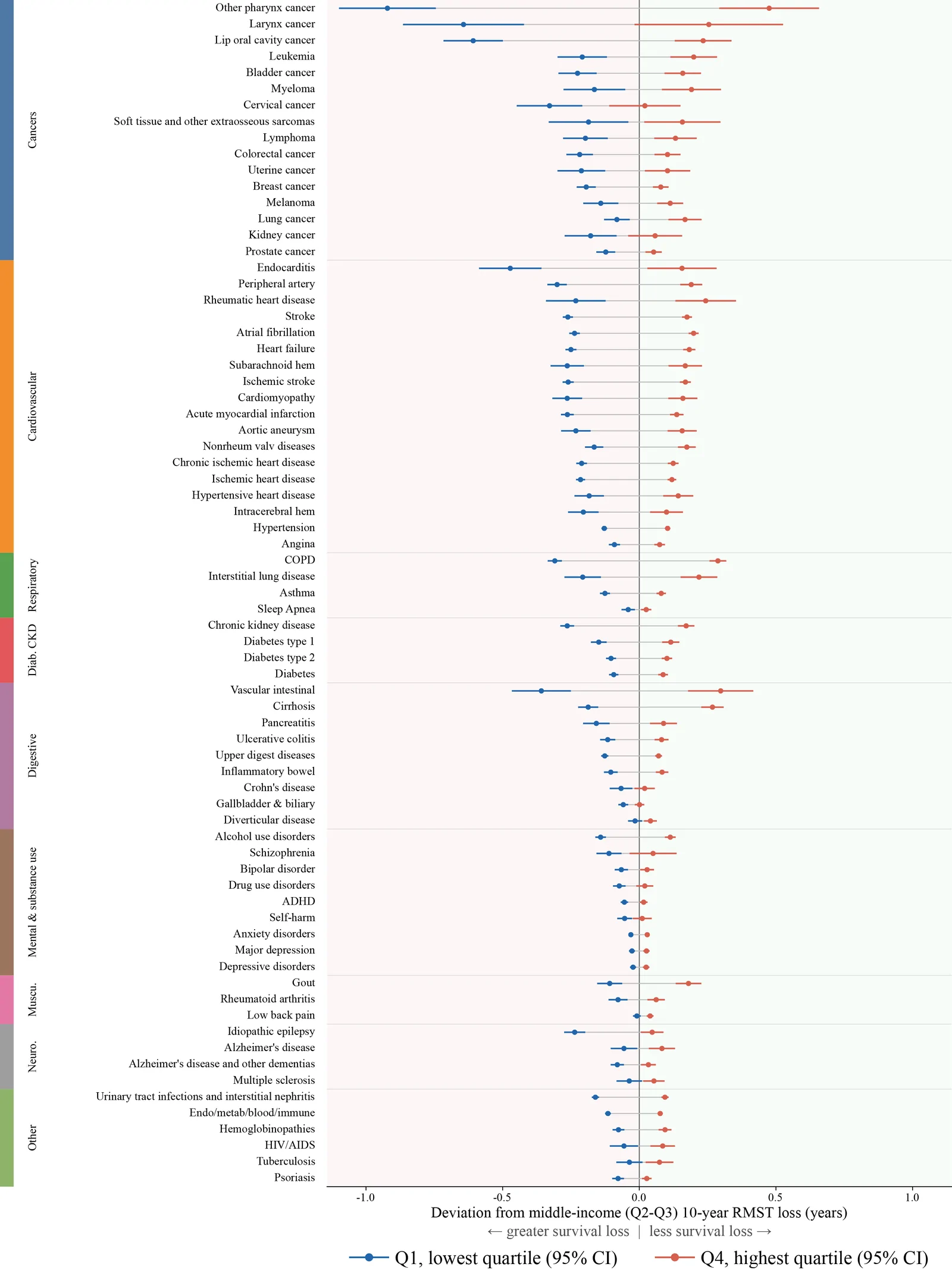
**Income-related differences in 10-year survival time lost after diagnosis.** Deviation, in years, of each income quartile's loss of restricted mean survival time (RMST) within 10 years of diagnosis from the middle-income reference. RMST loss is the difference between the RMST of a matched undiagnosed comparison group and that of the diagnosed cohort, and represents survival time lost following diagnosis. Values are plotted as the middle-income loss minus the quartile's own loss. Blue circles denote Q1 (lowest income quartile) and orange circles Q4 (highest); horizontal bars are 95% CIs. Forest plot limited to 73 disease categories with no overlap between Q1 and Q4 RMST loss 95% CI intervals, excluding osteoarthritis and unspecified dementia (reversed pattern).

## Discussion

In this study, we systematically examined survival following diagnosis across 100 disease categories by income level in Sweden over more than two decades. We found significant 10-year survival differences after diagnosis between the lowest and highest income quartiles for all disease categories, with differences exceeding 10 percentage points for 37 conditions. We quantified the total income-associated survival gap among diagnosed individuals by calculating the counterfactual scenario where low- and middle-income individuals experienced high-income survival patterns for the ten years after diagnosis, and found that this gap amounted to over 6 million person-years. As this total is inclusive of background survival differences, we compared survival loss relative to matched general population controls across income groups, and found that low income was associated with greater disease-related survival loss for the lowest income quartile across most diagnosis categories, beyond the baseline income gradient in the general population.

Income inequalities in modelled 10-year survival probability after adjusting for age, sex, and year of diagnosis with regression standardisation were observed across all 100 disease categories. The diseases with the largest per-person survival disparities (pharynx cancer, larynx cancer, lip and oral cavity cancer, and COPD) are predominantly smoking and alcohol related conditions, risk factors whose prevalence (smoking) and associated harm (alcohol) are concentrated among low-income individuals.^16^^,^^17^ Because smoking and alcohol affect many organ systems, they plausibly contribute to survival reductions across many of the disease categories we analysed. Healthcare access and treatment adherence play a role as well, particularly for aggressive cancers where delays between diagnosis and treatment substantially worsen survival^18^ and lower income is associated with later-stage presentation.^19^ For other pharynx cancer, which showed the largest survival difference of any category, HPV likely plays a part: HPV-positive oropharyngeal squamous cell carcinoma increased sharply in Sweden in recent decades and accounts for the majority of that cancer.^20^

The population-level interpretation for diagnosis categories with an overall 10-year probability of death around 50% is striking: in Sweden, ten years after an ischaemic heart disease diagnosis, those in the top income quartile are more likely to be alive than dead, while those in the bottom income quartile are more likely to be dead than alive. The same can be said for chronic ischaemic heart disease, hypertensive heart disease, ischaemic stroke, haemorrhagic stroke, aortic aneurysm, interstitial lung disease, prostate cancer, lip and oral cavity cancer, other pharynx cancer, and urinary tract infections and interstitial nephritis. Consistently large gaps in absolute survival probability by income were observed for conditions with overall 10-year survival probabilities between 40 and 60 percent, which were primarily from the cardiovascular group (Fig. 3; Appendix Fig. S1). Mean age of diagnosis explains part of this pattern, but it may also reflect that income-related risk factors such as smoking, drinking, and physical inactivity, or protective factors like timely and regular healthcare use, treatment adherence, and comorbidity management play a larger role among these diseases. However, ceiling and floor effects are a consideration, since for highly survivable conditions most patients achieve good outcomes regardless of resources, while for rapidly fatal conditions even optimal care yields low survival. While absolute percentage-point differences are mechanically constrained near the extremes of survival, these intermediate-prognosis diseases also share clinical features where care quality, treatment adherence, and behavioural factors can strongly influence survival.

Disease rankings by total person-years lost by income differed substantially from the ranking by per-person survival inequality by income. While other pharynx cancer, larynx cancer, and lip and oral cavity cancer exhibited the largest per-person differences in 10-year restricted mean survival time (1.9, 1.5, and 1.3 years, respectively), their relatively low incidence meant they contributed modestly to the total population burden. Conversely, common conditions such as hypertension and ischaemic heart disease, despite having smaller per-person disparities (0.3–0.5 years), accounted for disproportionate shares of the total person-years lost due to their high prevalence in the population. Policy targeting prevalent cardiovascular conditions may therefore yield the largest absolute reductions in survival inequalities, whereas action on the rarer conditions with the largest income gaps in survival could deliver the greatest benefit per person.

We observed a baseline gradient in survival in the general population by income for all disease categories assessed, with lower income individuals more likely to die regardless of disease status. If the impact of disease on mortality were equal across income groups (for example, if a disease reduced 10-year survival by the same absolute amount for each group) we would expect survival gaps after diagnosis to simply reflect these baseline differences. Instead, we consistently observed that the survival gap between the diagnosed and matched general population was larger for low-income individuals. This finding aligns with observations in cancer epidemiology, where deprivation-stratified relative survival analyses have shown that socioeconomic inequalities in cancer-specific survival persist after accounting for background mortality differences.^15^ Our extension of this approach to 100 disease categories, including cardiovascular diseases, chronic respiratory conditions, and mental disorders, suggests that this pattern is not unique to cancer but general across diverse conditions.

Income differences in survival could arise through two non-exclusive pathways that our design cannot fully distinguish. First, lower income may worsen the course of disease after onset, through differential access to or quality of treatment, adherence, comorbidity management, and related factors. Second, because our outcome is survival from first recorded diagnosis rather than from disease onset, given ascertainment limited to inpatient and specialised outpatient care, lower-income individuals may enter the cohort at a later or more advanced stage; this delayed detection would produce a wider post-diagnosis survival gap even in the absence of any difference in disease course thereafter. These two pathways imply different points of intervention: earlier detection and access to care vs post-diagnosis treatment, management, and risk-factor modification. The number and heterogeneity of disease categories found to have substantial survival gaps net of background income differences, spanning aggressive cancers, where prognosis turns heavily on how early disease is caught, to conditions where outcomes depend more on long-term management and underlying risk factors, suggests that no single mechanism accounts for the gradient. Prior studies have documented income gradients for individual diseases in Sweden,^10^, ^11^, ^12^, ^13^^,^^21^^,^^22^ the Nordic context,^23^ and beyond.^6^, ^7^, ^8^, ^9^ Our findings show that these separately observed gradients hold jointly across 100 disease categories under harmonised methods, indicating that income acts broadly across the course of illness rather than through any one disease-specific route.

Our estimates are likely conservative in two respects. First, the income indicator is sensitive to life-cycle effects: older individuals with modest pension income but otherwise secure circumstances may be classified as low-income, attenuating the measured association, although there is evidence of real gaps in income among those over 65 in Sweden and associated gaps in longevity.^24^ Second, this study was conducted in Sweden, a country with relatively low income inequality,^25^ universal healthcare, and established social safety nets, and disparities may be larger in a country with higher out-of-pocket costs or greater income inequality. That said, many health inequalities have persisted and widened in western European welfare states despite universal healthcare systems explicitly designed to deliver care independent of ability to pay,^26^ and income inequality in Sweden has risen faster than in most other OECD countries over recent decades, with the life expectancy gap between high- and low-income groups widening in parallel.^2^^,^^25^

Economic disadvantage operates across the whole of life, with material and psychosocial influences extending to most diseases and causes of death.^27^ Higher income affords a better diet, since those on low incomes tend to substitute cheaper processed foods for fresh produce, and more secure work with greater job control, low levels of which are associated with raised cardiovascular risk. It also protects against the chronic stress of financial insecurity, which contributes to heart disease, stroke, diabetes, and depression. These disadvantages tend to concentrate among the same people and accumulate over the life course.^27^ Differences in healthcare also persist under universal coverage. Although the Health and Medical Services Act commits the Swedish system to care on equal terms for the entire population,^28^ variation in the availability of care persists across socioeconomic groups and geographical regions.^29^ Lower income is associated with reduced use of primary and specialised outpatient care,^3^ and greater financial means ease the healthcare-adjacent costs of illness that can delay or deter care, such as prescription charges, travel to appointments, and lost earnings. Lower health literacy can impair recognition of early symptoms, hinder navigation of the healthcare system, and worsen patient-provider communication, delaying help-seeking and contributing to later-stage diagnosis and poorer survival.^19^ Policies aimed at helping low-income individuals engage with and navigate the healthcare system may improve health outcomes for this group, whether through higher chances of timely treatment or improved quality and continuity of care.

This study has several strengths. First, the 23-year observation period with near-complete population coverage enabled analysis of long-term survival patterns. Second, the systematic analysis of 100 disease categories using harmonised methods allows direct comparison of survival disparities across the full spectrum of major NCDs. Third, the Swedish national registers provide high-quality data on hospital diagnoses, mortality, and income level with minimal loss to follow-up. Fourth, the matched cohort design enables estimation of survival loss net of background mortality differences. Fifth, the use of flexible parametric models with regression standardisation produces survival estimates that are adjusted for age, sex, and calendar year while remaining interpretable.

This study also has important limitations. First, we did not control for multimorbidity, which is more prevalent among low-income individuals and likely mediates part of the income–survival association.^30^ As a result, the observed survival differences by income likely reflect both disease-specific effects and broader differences in underlying health, limiting the ability to isolate diagnosis-specific mechanisms. Second, primary care data were unavailable. Differential utilisation of specialist care by income could lead to differential ascertainment and bias estimated survival disparities. Although Sweden's healthcare system has traditionally been specialist-oriented,^29^ the direction and magnitude of any resulting bias remain uncertain. Third, cohort entry was defined by first observable adult contact rather than disease onset. The interval between cohort entry and true onset likely varies by condition, and findings should be interpreted as reflecting adult ascertainment rather than incident disease. Fourth, the quality of outpatient records, particularly for mental disorders, improved over the study period, which may affect the completeness and comparability of case ascertainment for these conditions. Fifth, income was measured using tax-reported data, which may underestimate resources available to individuals with unreported income sources or wealth. Sixth, while appropriate for most NCDs, the 10-year time horizon may not fully capture differences for all conditions. Finally, sex-specific patterns and temporal trends were not examined and represent important directions for future research.

Our findings support the view that income inequality is a fundamental cause of mortality differences, operating through multiple pathways across a wide range of diseases. Even in comparatively egalitarian societies, economic disadvantage remains a powerful factor for differences in health outcomes. Survival gaps by income were present for all diseases studied, and substantial differences in person-years lost persisted even after accounting for baseline income-related survival differences. Interventions targeting the most prevalent conditions, such as hypertension and ischaemic heart disease, could have the greatest overall impact, while the diseases with the largest per-person gaps, such as smoking- and alcohol-related cancers, may benefit from focused prevention and early detection. Addressing survival inequalities will require not only healthcare system interventions, such as enhanced screening, care coordination, and follow-up, but also broader upstream policies that reduce income inequality.

## Contributors

VM and EEA conceptualised the study and designed the analysis with AG, MN, PA, and CJLM, with VM, AG, MN, and CJLM responsible for the methods. EEA obtained the funding and provided resources and access to the register data. VM and AG were responsible for the formal analysis, investigation, validation, and software, with visualisation by VM. EEA, EEW, and VM administered the project. MN, PA, JMK, EEA, and EW supervised the study. VM wrote the first draft, which AG, MN, PA, JMK, EEW, NDW, CJLM, and EEA reviewed and edited. VM and AG directly accessed and verified the underlying data. All authors approved the final version and were responsible for the decision to submit for publication.

## Data sharing statement

Due to ethical restrictions, the individual level data used in this study are not publicly available. However, the data supporting the findings of this study can be obtained from the following sources: Statistics Sweden (www.scb.se) and The Swedish National Board of Health and Welfare (www.socialstyrelsen.se).

## Declaration of generative AI and AI-assisted technologies in the manuscript preparation process

During the preparation of this work the primary author used Claude Opus 4.5 (Anthropic) to facilitate writing the Python code that plots the figures with matplotlib from the underlying estimates. The authors reviewed and edited the code and output as needed and take full responsibility for the content of the published article.

## Declaration of interests

We declare no competing interests.

## Appendix A Supplementary data

Supplement

Supplementary Captions
