# Supplementary material for: Income-related differences in survival across disease categories in Sweden: a retrospective population-based register study
Source: Lancet Reg Health Eur. 2026 Oct 15;71:101896. doi: 10.1016/j.lanepe.2026.101896 (PMC13694925; doi:10.1016/j.lanepe.2026.101896)
Supplement: Supplementary file 1 [file mmc1.pdf]

Supplemental Methods Appendix

Sections

A. **Diagnosis records and disease classification**
B. **The disposable family income measure**
C. **The method for creating matched control cohorts**
D. **Analysis and modelling strategy**
E. **Model validations and robustness checks**
F. **STROBE Statement**
G. **References**

A. Diagnosis records and disease classification

Microdata were obtained from multiple Swedish national registers maintained by Statistics Sweden and the National Board of Health and Welfare and made available for research purposes by application (1). These registers, assembled by the National Board of Health and Welfare in collaboration with Statistics Sweden, include the Total Population Register (TPR), the National Patient Register (NPR) (inpatient and outpatient specialized care), the Cause of Death Register (DORS), and the Longitudinal Integrated Database for Health Insurance and Labour Market Studies (LISA), and integrated register system built by Statistics Sweden that combines multiple administrative registers. All Swedish residents are assigned a unique personal identification number (PIN), which has been used as the unique identifier for all hospital discharges since 1993. An adapted serial number derived from the PIN enabled individual-level linkage across registers. These registers are frequently used for population-based research in Sweden and are described individually in detail by Ludvigsson et al. (2–4) and Everhov et al. (5).

Basic demographic information, including sex and dates of birth, was obtained from the TPR, which provides virtually complete coverage of births and deaths. Death dates were linked from DORS. Disease classification was based on diagnoses recorded in the Swedish National Patient Register (NPR) using International Classification of Diseases, 10th Revision (ICD-10) codes. The NPR provides nationwide comprehensive coverage of specialized inpatient care from 1964 and specialized outpatient care from 2001, but does not include primary care data. In our study we included NPR data from 1997 through 2023.

The data are extracted from the medical records of healthcare providers and recorded in their patient administrative systems. These data must be submitted in a standardized and coherent format for all units within a healthcare provider via the National Board of Health and Welfare's electronic file transfer services.

The NPR has undergone external validation in previous research. This show generally good diagnostic accuracy overall, with particularly high validity for conditions such as myocardial infarction, stroke, heart failure, hip fracture, appendicitis, gallstone disease requiring surgery, inflammatory bowel disease, cancer diagnoses, and obstetric outcomes. In contrast, validity is more variable for psychiatric disorders, conditions primarily managed in primary care, and diagnoses based on less specific or symptom-based coding (5)

Cohorts were defined based on first recorded diagnoses and subsequently matched to individuals without a prior record of the condition in the NPR to enable relative survival comparisons.

B. The disposable family income measure

Socioeconomic status was assessed using consumption-equivalized family disposable income from the LISA database. Disposable income was defined as total income net of taxes and included earned income (wages), business income (self-employment), parental allowances, sick leave benefits, disability pension, unemployment benefits, and social welfare or general assistance. Income was aggregated at the family level, where family was defined as individuals connected by marriage, registered partnership, or parent-child relationships, in accordance with Statistics Sweden definitions.

This disposable income variable is adjusted for family size by Statistics Sweden using consumption weights from the Statistics Sweden (SCB) equivalence scale, a slightly modified version of the OECD equivalence scale (“Svenska konsumtionsvikter”). Each individual in the family unit contributes a weight to the total family weight, and the total disposable income of the family for that year is divided by this weight. The possible weights are:

- One adult: 1.16
- Two adults: 1.92
- Child 0-3 years: 0.56
- Child 4-10 years: 0.66
- Child 11-17 years: 0.76
- Another adult (18+): 0.96

For example, a family consisting of two adults, one child 4-10 years, and one child 11-17 years, would have a total consumption weight of 1.92 (two adults) + 0.66 (child 4-10 years) + 0.76 (child 11-17 years) = 3.34. If both adults work and the total family income net of taxes is 500,000 kroner, then the consumption equivalized family disposable income for each individual is 500,000 / 3.34 = 149,700.

Equivalized family disposable income was calculated annually, inflation-adjusted to 2024 Swedish kronor using the Consumer Price Index, and averaged over the five preceding years to reduce short-term income volatility. Another reason for using 5-year lagged income rather than

current income or the prior year income is that disease onset may have occurred one year or more before first diagnosis and impacted family income. Individuals in the bottom 2% of the income distribution were excluded because this group comprised a mixture of negative, zero, or implausibly low incomes likely reflecting unreported income sources. We excluded individuals below the 2nd percentile of income because this group likely does not represent the economically disadvantaged population in Sweden. The country's robust social transfer system typically places individuals experiencing poverty above this threshold, meaning the bottom 2% disproportionately consists of individuals with low reported income but alternative sources of support, such as students, artists, entrepreneurs, or those with wealth but low reported earnings (in the case of negative incomes). The remaining individuals were assigned an income percentile for the calendar year by age and sex, and categorized into quartiles.

Details on SCB's definition of the family unit are presented in the table below.

**Situation**	**How SCB defines the family**
Married or registered partners with or without common children	One family
Cohabiting partners **with common children**	One family
Cohabiting partners **without common children**	**Two separate families** (each person is their own family)
Single parent with children	One family
Adult child (≥18) living with parents but without partner or own children	Belongs to parents' family
Several generations in same home (e.g., grandparents, parents, kids)	Each parent–child pair forms their own family unit
Unrelated adults sharing a home	Separate families

C. The method for creating matched control cohorts

ICD-10 codes were mapped to cause categories using mappings developed for the Global Burden of Disease (GBD) multiple/contributing-cause framework. These mappings were adopted to maintain consistency and reproducibility across cause definitions.

One feature of this mapping schema is that some cause categories include ICD-10 codes denoting personal history, family history, perinatal conditions, screening, preventive care, or other non-active disease encounters in addition to codes representing active disease. We retained these codes as specified in the source mappings. This approach prioritizes complete case ascertainment and reduces the likelihood that individuals with evidence of a condition are misclassified into the general-population comparison cohort. In practice, such codes are uncommon relative to active-disease codes, and most individuals assigned to a cause category also have corresponding active-disease diagnoses; therefore, their impact on overall estimates is expected to be small.

For each diagnosed individual, five controls were selected from the general population who shared the same birth year, sex, and income level but had no recorded diagnosis of the condition of interest prior to or during the diagnosis year of the case. Controls entered follow-up on the date of diagnosis of the corresponding diagnosed individual.

Matching was performed with replacement, allowing a single control individual to be selected for more than one case if eligibility criteria were met. This approach preserves the representativeness of the control cohort and improves statistical efficiency, per Iwagami et al. (6). Matched controls needed to be undiagnosed with the analytic disease category prior to the date of matching, but were not excluded if they were subsequently diagnosed with the disease (7).

Following matching, the analytic dataset comprised all individuals aged 16 years or older who were diagnosed with a given condition in inpatient or specialized outpatient care in Sweden between 2001 and 2023, along with a matched control cohort five times larger and identical in its joint distribution of birth year, sex, and income level. Risk ratios derived from these cohorts are therefore unbiased with respect to the matching variables, although residual confounding by unmeasured factors cannot be excluded (8).

D. Analysis and modelling strategy

Individuals in the study population were followed from the date of cohort entry until death or the end of follow-up, whichever occurred first. Time at risk was defined as the duration between study entry and exit. All analyses were conducted separately for each diagnosis of interest.

Exposure variable and covariates

The income variable was categorized into quartiles. For modeling purposes, the middle two quartiles were combined and used as the reference category. The lowest income quartile (SDH_1) and the highest income quartile (SDH_4) were each compared against this reference.

Age at diagnosis was centered by subtracting the mean age to improve numerical stability and interpretability of interaction terms. Sex was coded as a categorical variable, with the more prevalent sex among cases set as the reference category.

Model Specification

We employed flexible parametric survival models (Royston–Parmar models), implemented using the stpm2 function in the **rstpm2** R package. These models use restricted cubic splines to model the log cumulative baseline hazard, allowing for smooth and flexible hazard functions while avoiding restrictive parametric assumptions.

For diagnoses affecting both sexes, the general model was specified as:

$$\log H(t \mid X) = s(\log t; \gamma) + \beta_1 \cdot \text{SDH}_1 + \beta_2 \cdot \text{SDH}_4 + f_1(\text{age}_{\text{centered}}) + f_2(\text{diag\_year}) + \beta_3 \cdot \text{sex}$$

$$+\text{SDH}_1 \cdot f_1(\text{age}_{\text{centered}}) + \text{SDH}_4 \cdot f_1(\text{age}_{\text{centered}})$$

$$+\text{SDH}_1 \cdot f_2(\text{diag\_year}) + \text{SDH}_4 \cdot f_2(\text{diag\_year}) + \beta_4 \cdot \text{SDH}_1 \cdot \text{sex} + \beta_5 \cdot \text{SDH}_4 \cdot \text{sex}$$

where:

- $H(t \mid X)$ is the cumulative hazard at time t given covariates X
- $s(\log t; \gamma)$ is a restricted cubic spline of log time with 5 degrees of freedom
- SDH_1 and SDH_4 indicate the lowest and highest income quartiles (reference = middle quartiles)
- $f_1(\text{age}_{\text{centered}})$ is a natural spline of centered age at diagnosis with 4 degrees of freedom
- $f_2(\text{diag\_year})$ is a natural spline of calendar year of diagnosis with 3 degrees of freedom
- Sex is coded with the more prevalent sex among cases as the reference level

For sex-restricted diagnoses, sex terms were omitted:

$$\log H(t \mid X) = s(\log t; \gamma) + \beta_1 \cdot \text{SDH}_1 + \beta_2 \cdot \text{SDH}_4 + f_1(\text{age}_{\text{centered}}) + f_2(\text{diag\_year})$$

$$+\text{SDH}_1 \cdot f_1(\text{age}_{\text{centered}}) + \text{SDH}_4 \cdot f_1(\text{age}_{\text{centered}}) + \text{SDH}_1 \cdot f_2(\text{diag\_year}) + \text{SDH}_4 \cdot f_2(\text{diag\_year})$$

Interaction terms between income and age at diagnosis as well as calendar year were included to allow the socioeconomic gradient in survival to vary across age groups and over time.

Regression Standardization

To obtain adjusted survival curves that are comparable across income groups, we applied regression standardization (also known as marginal standardization or g-computation). This approach estimates the counterfactual survival that would be observed if the entire population had a given income level, while preserving the observed joint distribution of confounders.

The standardized survival probability for income group k at time t was computed as:

$$\bar{S}_k(t) = \frac{1}{N} \sum_{i=1}^{N} \hat{S}(t \mid \text{SDH}_k, X_i)$$

where:

- N is the total number of individuals in the analysis population
- $\hat{S}(t \mid \text{SDH}_k, X_i)$ is the predicted survival probability for individual i, obtained by setting income to level k while retaining that individual's observed values of other covariates

Equivalently, when predictions are aggregated over unique covariate patterns, the standardized survival can be expressed as:

$$\bar{S}_k(t) = \frac{\sum_j w_j \cdot \hat{S}(t \mid \text{SDH}_k, \tilde{X}_j)}{\sum_j w_j}$$

where:

- $\tilde{X}_j$ denotes a unique covariate pattern
- w_j is the number of individuals exhibiting covariate pattern $\tilde{X}_j$

Uncertainty Quantification

Uncertainty in standardized survival estimates was quantified using a parametric bootstrap procedure:

1. Maximum likelihood estimates of model coefficients and their variance–covariance matrix were obtained
2. A total of 1,000 bootstrap samples of the coefficient vector were drawn from a multivariate normal distribution
3. Standardized survival curves were computed for each bootstrap sample
4. The 2.5th and 97.5th percentiles across bootstrap samples were used to construct 95% confidence intervals
5. A fixed random seed (12345) was used to ensure reproducibility

This approach captures uncertainty in all model parameters simultaneously without requiring repeated model refitting.

Survival Predictions

Survival probabilities were predicted at regular intervals from 0 to 10 years post-diagnosis, at increments of 0.1 years (approximately monthly). For each time point and income category, we estimated:

- Point estimates (mean bootstrap survival probability)
- 95% confidence intervals (2.5th and 97.5th percentiles)
- Standard errors (standard deviation of bootstrap estimates)

Predictions were generated separately for diagnosed individuals and general population controls, enabling the calculation of relative survival and excess mortality outcomes.

Person-years lost calculation

As a measure of the total burden of survival disparities associated with differences in income levels, we calculated person-years of survival lost within 10 years of diagnosis for each disease category (9). The 10-year RMST, the area under the survival curve up to 10 years: 10-year RMST is the average number of years lived within 10 years of diagnosis (9). In the literature, this is also referred to as the life expectancy between cohort entry and a particular time horizon (9). Differences in RMST between income groups can be directly interpreted as years of life lost (10).

Person-years lost was calculated as the product of the RMST difference between income groups and the number of individuals in the lower-income group: $PY\_lost = (RMST\_high - RMST\_low) \times N\_low$. We computed this measure separately for two comparisons: low income (Q1) versus high income (Q4), and middle income (Q2-Q3 combined) versus high income (Q4). Subcauses were excluded for this calculation as this would have led to double counting (for example, major depression and chronic ischemic heart disease were excluded as these are captured in the depression and ischemic heart disease cause categories).

For each comparison within a disease category, 95% confidence intervals for the RMST difference were calculated assuming independence of the estimates, with standard errors computed as $SE\_diff = \sqrt{SE\_low^2 + SE\_high^2}$. Statistical significance was determined by whether the 95% confidence interval for the RMST difference excluded zero (equivalent to $p<0.05$).

For the aggregate total across all disease categories, confidence intervals were computed by summing the individual lower and upper confidence bounds. This approach is conservative, producing wider intervals than would be obtained under an assumption of independence across disease categories. Because individuals may contribute to multiple disease cohorts, estimates across categories are positively correlated; summing bounds provides an upper bound on sampling variability that accommodates this correlation structure. The combined total represents the counterfactual person-years of survival within 10 years of diagnosis in the scenario where individuals in the lowest 75% of the income distribution experienced the same survival as those in the highest income quartile after diagnosis, inclusive of background mortality.

The person-years measure above captures the total survival gap between income groups among diagnosed individuals, but part of this gap reflects the baseline survival disadvantage of lower-income individuals in the general population, independent of any specific diagnosis. To isolate the survival loss attributable to disease over and above this background gradient, we used an additive difference-in-differences approach.

For each income group g and disease category, we defined disease-related survival loss as the difference in 10-year RMST between the diagnosed cohort and a matched general-population reference of the same income group:

$$\Delta RMST\_Qx = RMST\_general\_Qx - RMST\_diagnosed\_Qx$$

$$DiD = \Delta RMST\_Q1 - \Delta RMST\_Q4$$

A DiD of zero indicates that the diagnosis reduces 10-year survival by the same absolute amount in both income groups, so that any observed survival gap among the diagnosed simply mirrors the pre-existing background gap. A positive DiD indicates that the disease erodes survival more steeply in the lower-income group than in the high-income group, over and above background differences. We computed this contrast for Q1 versus Q4 and for Q2–Q3 versus Q4. Uncertainty intervals were obtained by propagating the standard errors of the four constituent RMST estimates, assuming independence between the diagnosed and general-population estimates and between income groups.

Software

All analyses were conducted in R using the following packages: rstpm2 for flexible parametric survival models, survival for survival data structures, dplyr and tidyverse for data manipulation, MASS for multivariate normal sampling in the bootstrap procedure, and arrow for reading parquet data files.

E. **Model validation**

We compared model-based survival estimates with non-parametric Kaplan-Meier survival curves within each income category. Kaplan-Meier survival functions were estimated separately for individuals in the lowest income quartile (Q1), the combined middle quartiles (Q2-Q3), and the highest income quartile (Q4).

For each income stratum, model-predicted survival curves were generated using observed covariate distributions, without regression standardization, to ensure comparability with the Kaplan-Meier estimates. Kaplan-Meier and model-based curves were overlaid for visual inspection across the first ten years after diagnosis.

Model performance was additionally summarized using restricted mean survival time (RMST) estimates and maximum absolute deviations between Kaplan–Meier and model-predicted survival probabilities. Across income strata, model-predicted survival closely tracked the corresponding Kaplan-Meier estimates (largest difference under 5%), with no evidence of systematic over- or under-estimation at specific time points. Differences in RMST were small

(under 5%), supporting the adequacy of the flexible parametric survival models for the primary analyses

Sensitivity analysis: Exclusion of patients with less than 30 days follow-up

We conducted a sensitivity analysis excluding individuals who died within 30 days of their first recorded diagnosis (n = 1,401,461 cohort entries in the main analysis). This exclusion addresses the possibility that acute case-fatality events could disproportionately affect estimates if early deaths differ systematically by income group.

Flexible parametric survival models were re-fitted for all 100 disease categories using the restricted cohort. We compared 10-year RMST estimates, 10-year survival probabilities, and income gradients (Q4 minus Q1) between the main analysis and the sensitivity analysis.

Results were highly concordant between the two approaches. Across 100 diagnoses, the mean change in 10-year RMST was 0.24 years (4.8%), with changes ranging from 0.00 (ADHD) to 1.41 years (intracerebral haemorrhage). Correlations between main and sensitivity analysis estimates were very high: for 10-year RMST, Pearson $r = 0.996$ and Spearman $\rho = 0.995$; for 10-year survival probability, Pearson $r = 0.999$ and Spearman $\rho = 0.998$. Importantly, the income gradient in survival (Q4 minus Q1 RMST) was also highly correlated between analyses (Pearson $r = 0.993$, Spearman $\rho = 0.991$), with no diagnoses showing a reversal in the direction of the income-survival association. These findings indicate that our primary results are robust to the exclusion of early deaths and that income-related survival disparities are not driven by differential patterns of early deaths by income group.

Sensitivity analysis: Extended Washout Period (2005+ Diagnoses)

We conducted a sensitivity analysis excluding all diagnoses from 2001–2004, extending the washout period to eight years. This analysis compared 10-year restricted mean survival time (RMST) estimates across 100 diagnoses and 300 diagnosis-by-income-quartile combinations.

Agreement between the main and sensitivity analyses was excellent (Pearson $r = 0.999$, Spearman $\rho = 0.999$). Although paired statistical tests detected a statistically significant mean difference in RMST (paired t-test $p = 0.004$; Wilcoxon $p < 0.001$), the magnitude was clinically negligible: the mean RMST difference was 0.014 years (approximately 5 days), with 78% of RMST estimates differing by less than 0.1 years and 100% differing by less than 0.5 years (95% limits of agreement: -0.15 to 0.18 years). Importantly, changes were bidirectional rather than systematically upward as would be expected if prevalent case misclassification inflated survival estimates: 58% of RMST estimates increased while 42% decreased. The income gradient was fully preserved, with no diagnoses showing a reversal in the direction of the socioeconomic survival gap. Results for 10-year survival probability point estimates showed similar agreement

(Pearson r = 0.999). These results indicate that our findings are robust to the potential of including more prevalent cases in the early study years with different survival patterns.

F. STROBE statement

Item No	Topic	Recommendation	Reporting location / status
**Title and abstract**			
**1**	**Title and abstract**	(a) Indicate the study's design with a commonly used term in the title or the abstract<br>(b) Provide in the abstract an informative and balanced summary of what was done and what was found	(a) Title and abstract<br>(b) Summary (Background/Methods/Findings/Interpretation) and Research in context.
**Introduction**			
**2**	**Background/ rationale**	Explain the scientific background and rationale for the investigation being reported	Introduction
**3**	**Objectives**	State specific objectives, including any prespecified hypotheses	Summary, Introduction
**Methods**			
**4**	**Study design**	Present key elements of study design early in the paper	Methods "Study design and participants"
**5**	**Setting**	Describe the setting, locations, and relevant dates, including periods of recruitment, exposure, follow-up, and data collection	Methods "Study design and participants"
**6**	**Participants**	(a) Give the eligibility criteria, and the sources and methods of selection of participants. Describe methods of follow-up<br>(b) For matched studies, give matching criteria and number of exposed and unexposed	(a) Methods "Study design and participants", Figure 1 (Inclusions)<br>(b) Methods "Statistical analysis"
**7**	**Variables**	Clearly define all outcomes, exposures, predictors, potential confounders, and effect modifiers. Give diagnostic criteria, if applicable	Methods: Outcomes, Statistical analysis.
**8***	**Data sources/ measurement**	For each variable of interest, give sources of data and details of methods of assessment (measurement). Describe comparability of assessment methods if there is more than one group	Methods "Study design and participants"
**9**	**Bias**	Describe any efforts to address potential sources of bias	Methods: 5-year lagged income (reduce reverse causation/volatility); exclusion of bottom 2% incomes; matching on age/sex/income; sensitivity analyses (exclude <30 days follow-up; restrict to 2005 onward). Discussion, limitations (ascertainment, multimorbidity, primary-care absence).
**10**	**Study size**	Explain how the study size was arrived at	Results (first paragraph).<br>Figure 1 inclusions
**11**	**Quantitative variables**	Explain how quantitative variables were handled in the analyses. If applicable, describe which groupings were chosen and why	Methods (Income and sociodemographic factors, Statistical analysis)

Item No	Topic	Recommendation	Reporting location / status
**12**	**Statistical methods**	(a) Describe all statistical methods, including those used to control for confounding<br>(b) Describe any methods used to examine subgroups and interactions<br>(c) Explain how missing data were addressed<br>(d) If applicable, explain how loss to follow-up was addressed<br>(e) Describe any sensitivity analyses	(a) "Statistical analysis" (flexible parametric models + regression standardisation; income*age, income*year, income*sex interactions).<br>(b) Income×age and income×calendar-year interactions described<br>(c) Methods, Supplementary methods appendix<br>(d) Register-based study with administrative censoring at end of 2023<br>(e) Methods (Statistical analysis) / Results: exclude <30 days follow-up; restrict to 2005 onward, Appendix.
**Results**			
**13***	**Participants**	(a) Report numbers of individuals at each stage of study—eg numbers potentially eligible, examined for eligibility, confirmed eligible, included in the study, completing follow-up, and analysed<br>(b) Give reasons for non-participation at each stage<br>(c) Consider use of a flow diagram	(a) Results, first paragraph (12,167,578 eligible; 7,192,290 with a diagnosis; 31,520,218 cohort entries; exclusions itemised; final 6,849,397 individuals / 28,038,773 entries / 196,882,327 person-years).<br>(b) Reasons given (missing income/bottom 2%; <5 matched controls; zero follow-up due to same-day death).<br>(c) Figure 1 (flow diagram).
**14***	**Descriptive data**	(a) Give characteristics of study participants (eg demographic, clinical, social) and information on exposures and potential confounders<br>(b) Indicate number of participants with missing data for each variable of interest<br>(c) Summarise follow-up time (eg, average and total amount)	(a) Table 1, appendix table 2 (by income quartile); multimorbidity (appendix table 2).<br>(b) Figure 1 (inclusions), linkage of available records<br>(c) Figure 1, Table 1
**15***	**Outcome data**	Report numbers of outcome events or summary measures over time	Table 2, Appendix Tables A4-A6
**16**	**Main results**	(a) Give unadjusted estimates and, if applicable, confounder-adjusted estimates and their precision (eg, 95% confidence interval). Make clear which confounders were adjusted for and why they were included<br>(b) Report category boundaries when continuous variables were categorized<br>(c) If relevant, consider translating estimates of relative risk into absolute risk for a meaningful time period	(a) Results: unadjusted (appendix table A2) standardised marginal survival and RMST with 95% CIs (figures 3–4; appendix table 7); adjustment for age, sex, year via standardisation dscribed in Methods (Statistical analysis)<br>(b) Methods (Income and sociodemographic factors) Methods appendix<br>(c) Results present absolute measures throughout (10-year survival probabilities, RMST, person-years)
**17**	**Other analyses**	Report other analyses done—eg analyses of subgroups and interactions, and sensitivity analyses	Results: person-years-lost burden analysis; RMST-loss vs matched controls (figure 5); sensitivity analyses (<30 days excluded; 2005-onward) in appendix pp 8–9.
**Discussion**			
**18**	**Key results**	Summarise key results with reference to study objectives	Discussion, first paragraph (significant gaps across all 100 categories; >10 pp for 37; >6 million person-years; excess disease-related loss in Q1).
**19**	**Limitations**	Discuss limitations of the study, taking into account sources of potential bias or imprecision. Discuss both direction and magnitude of any potential bias	Discussion limitations paragraph (multimorbidity not controlled; no primary-care data; cohort entry ≠ onset; outpatient data quality over time;

Item No	Topic	Recommendation	Reporting location / status
			tax- (Inclment; 10-year horizon; sexes/period combined).
**20**	**Interpretation**	Give a cautious overall interpretation of results considering objectives, limitations, multiplicity of analyses, results from similar studies, and other relevant evidence	Discussion, Limitations, and Conclusion.
**21**	**Generalisability**	Discuss the generalisability (external validity) of the study results	Discussion (Sweden context paragraph) Conclusion (framework applicable to other settings).
**Other information**			
**22**	**Funding**	Give the source of funding and the role of the funders for the present study and, if applicable, for the original study on which the present article is based	"Role of the funding source" paragraph; Summary "Funding" (Swedish Research Council for Health, Working Life and Welfare).

**Give information separately for exposed and unexposed groups.*

G. References

1. Statistikmyndigheten SCB [Internet]. [cited 2026 Jan 21]. Register. Available from: https://www.scb.se/vara-tjanster/bestall-data-och-statistik/register/

2. Ludvigsson JF, Andersson E, Ekbom A, Feychting M, Kim JL, Reuterwall C, et al. External review and validation of the Swedish national inpatient register. BMC Public Health. 2011 Jun 9;11:450. doi:10.1186/1471-2458-11-450 PubMed PMID: 21658213; PubMed Central PMCID: PMC3142234.

3. Ludvigsson JF, Almqvist C, Bonamy AKE, Ljung R, Michaëlsson K, Neovius M, et al. Registers of the Swedish total population and their use in medical research. Eur J Epidemiol. 2016 Feb 1;31(2):125–36. doi:10.1007/s10654-016-0117-y

4. Ludvigsson JF, Svedberg P, Olén O, Bruze G, Neovius M. The longitudinal integrated database for health insurance and labour market studies (LISA) and its use in medical research. Eur J Epidemiol. 2019 Apr;34(4):423–37. doi:10.1007/s10654-019-00511-8

5. Everhov ÅH, Frisell T, Osooli M, Brooke HL, Carlsen HK, Modig K, et al. Diagnostic accuracy in the Swedish national patient register: a review including diagnoses in the outpatient register. Eur J Epidemiol. 2025;40(3):359–69. doi:10.1007/s10654-025-01221-0 PubMed PMID: 40140143; PubMed Central PMCID: PMC12137447.

6. Iwagami M, Shinozaki T. Introduction to Matching in Case-Control and Cohort Studies. Ann Clin Epidemiol. 2022 Apr 4;4(2):33–40. doi:10.37737/ace.22005 PubMed PMID: 38504854; PubMed Central PMCID: PMC10760465.

7. Ederer F, Axtell LM, Cutler SJ. The relative survival rate: a statistical methodology. Natl Cancer Inst Monogr. 1961 Sep;6:101–21. PubMed PMID: 13889176.

8. Sjölander A, Greenland S. Ignoring the matching variables in cohort studies – when is it valid and why? Statistics in Medicine. 2013 Nov 30;32(27):4696–708. doi:10.1002/sim.5879

9. Royston P, Parmar MK. Restricted mean survival time: an alternative to the hazard ratio for the design and analysis of randomized trials with a time-to-event outcome. BMC Med Res Methodol. 2013 Dec 7;13(1):152. doi:10.1186/1471-2288-13-152

10. Mozumder SI, Rutherford MJ, Lambert PC. Estimating restricted mean survival time and expected life-years lost in the presence of competing risks within flexible parametric survival models. BMC Med Res Methodol. 2021 Mar 11;21(1):52. doi:10.1186/s12874-021-01213-0 PubMed PMID: 33706711; PubMed Central PMCID: PMC7953595.

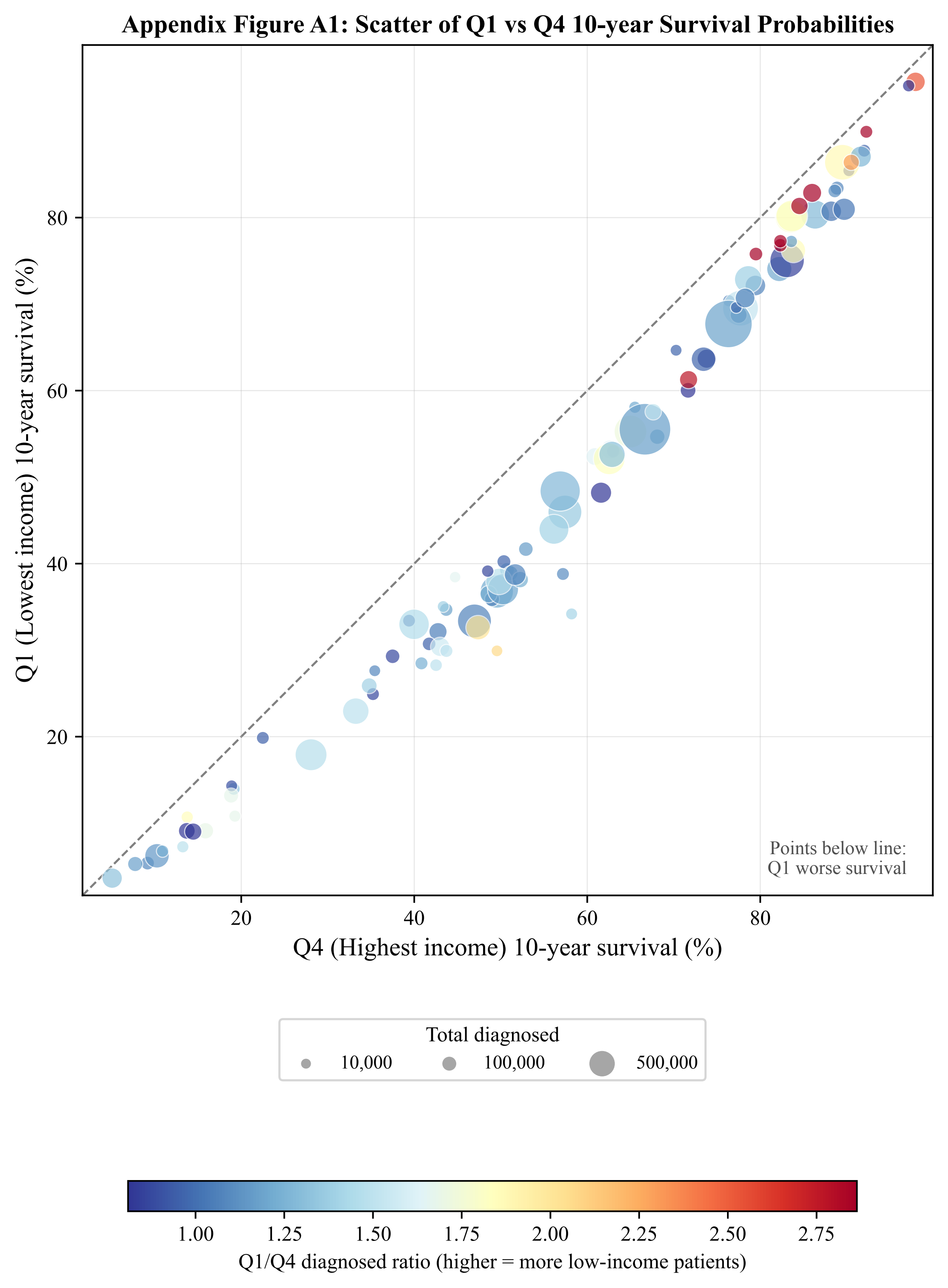
Appendix Figure A1: Scatter of Q1 vs Q4 10-year Survival Probabilities
Q1 (Lowest income) 10-year survival (%)
Q4 (Highest income) 10-year survival (%)
20
40
60
80
Points below line:
Q1 worse survival
Total diagnosed
10,000
100,000
500,000
1.00
1.25
1.50
1.75
2.00
2.25
2.50
2.75
Q1/Q4 diagnosed ratio (higher = more low-income patients)

Appendix Table A1: ICD Code Mapping for Disease Categories (Page 1 of 4)

Group	Diagnosis Category	Code List
Cancers	Bladder cancer	C67-C679, Z8052, Z8551
Cancers	Brain cancer	C70-C729, C751-C753, Z85841-Z85848, Z86011
Cancers	Breast cancer	C50-C50929, Z86000
Cancers	Cervical cancer	C53-C539, Z8542
Cancers	Colorectal cancer	C18-C219, Z8503-Z85048, Z86010
Cancers	Esophageal cancer	C15-C159, Z8501
Cancers	Gallbladder cancer	C23-C249
Cancers	Hodgkin lymphoma	C81-C8199, Z8571-Z8572
Cancers	Kidney cancer	C64-C659, Z8051, Z8552-Z8554
Cancers	Larynx cancer	C32-C329, Z8521
Cancers	Leukemia	C91-C9592, Z806, Z856
Cancers	Lip oral cavity cancer	C00-C089, C430, C440-C4400, C4402-C4409, Z8581-Z85810
Cancers	Liver cancer	C22-C228, Z8505
Cancers	Lung cancer	C33-C3492
Cancers	Lymphoma	C82-C866, C96-C969
Cancers	Malignant neoplasm of bone & articular cartilage	C40-C419
Cancers	Melanoma	C43, C431-C439, Z8582-Z85828
Cancers	Myeloma	C88-C909
Cancers	Nasopharynx cancer	C11-C119
Cancers	Other pharynx cancer	C09-C109, C12-C139
Cancers	Ovarian cancer	C56-C569, Z8041, Z8543
Cancers	Pancreatic cancer	C25-C259, Z8507
Cancers	Prostate cancer	C61-C619, Z8042, Z8546
Cancers	Soft tissue and other extraosseous sarcomas	C49-C499
Cancers	Stomach cancer	C16-C169, Z8502-Z85028
Cancers	Testicular cancer	C62-C6292, Z8043, Z8547-Z8548
Cancers	Thyroid cancer	C73-C739, Z85850
Cancers	Uterine cancer	C54-C549, Z86001
Cardiovascular diseases	Acute myocardial infarction	I21-I249
Cardiovascular diseases	Angina	I20-I209

Appendix Table A1: ICD Code Mapping for Disease Categories (Page 2 of 4)

Group	Diagnosis Category	Code List
Cardiovascular diseases	Aortic aneurysm	I71-I719
Cardiovascular diseases	Atrial fibrillation	I48-I4892
Cardiovascular diseases	Cardiomyopathy	B332-B3320, B3322-B3324, D8685, I40-I411, I418-I439, I514-I516
Cardiovascular diseases	Chronic ischemic heart disease	I25-I259
Cardiovascular diseases	Endocarditis	B3321, I33-I339, I38-I399
Cardiovascular diseases	Heart failure	I27, I278-I279, I50-I509, J81-J811
Cardiovascular diseases	Hypertension	I10-I109, I15-I159, I674, R03-R030
Cardiovascular diseases	Hypertensive heart disease	I11-I119
Cardiovascular diseases	Intracerebral hem	I61-I62, I621-I629, I681-I682, I691-I69298
Cardiovascular diseases	Ischemic heart disease	I20-I259, Z824-Z8249
Cardiovascular diseases	Ischemic stroke	G45-G468, I63-I639, I65-I669, I672-I673, I675-I676, I693-I69398
Cardiovascular diseases	Nonrheum valv diseases	I34-I379
Cardiovascular diseases	PAH	I270
Cardiovascular diseases	Peripheral artery	I702-I70799, I73-I739
Cardiovascular diseases	Rheumatic heart disease	I01-I019, I020, I05-I099
Cardiovascular diseases	Stroke	G45-G468, I60-I673, I675-I676, I678-I68, I681-I69398, I698-I69998, Z823
Cardiovascular diseases	Subarachnoid hem	I60-I609, I620-I6203, I670-I671, I690-I69098
Chronic respiratory	Asthma	J45-J469, Z825
Chronic respiratory	COPD	J40-J449, J47-J479
Chronic respiratory	Interstitial lung disease	D86-D862, D8689-D869, J84-J849
Chronic respiratory	Pneumoconiosis	J60-J650, J920
Chronic respiratory	Sleep Apnea	G473-G4739
Diabetes & CKD	Acute glomerulonephritis	N00-N019
Diabetes & CKD	Chronic kidney disease	D631, E102-E1029, E112-E1129, I12-I139, N02-N09, N150, N17-N199, Z49-Z4932, Z524, Z992
Diabetes & CKD	Diabetes	E08-E089, E10-E1011, E103-E111, E113-E149, P702, R73-R739
Diabetes & CKD	Diabetes type 1	E10-E1011, E103-E109, P702
Diabetes & CKD	Diabetes type 2	E11-E111, E113-E119
Digestive diseases	Cirrhosis	B18-B189, I85-I859, I982, K70-K7031, K717, K73-K75, K752, K754-K762, K764-K769, K778, Z526, Z944
Digestive diseases	Crohn's disease	K50-K50919, M074, M091
Digestive diseases	Diverticular disease	K57-K5793

Appendix Table A1: ICD Code Mapping for Disease Categories (Page 3 of 4)

Group	Diagnosis Category	Code List
Digestive diseases	Gallbladder & biliary	K80-K839, K87-K871
Digestive diseases	Inflammatory bowel	K50-K52, K528-K529, M074-M075, M091-M092
Digestive diseases	Pancreatitis	K85-K869
Digestive diseases	Ulcerative colitis	K51-K52, K528-K529, M075, M092
Digestive diseases	Upper digest diseases	K21-K219, K227-K22719, K25-K30, K84, R12-R120
Digestive diseases	Vascular intestinal	K55-K559
Mental disorders & substance use	ADHD	F90-F909
Mental disorders & substance use	Alcohol use disorders	E244, F10-F1099, G312, G621, G721, P043, Q860, R780, X45-X459, X65-X659, Y15-Y159, Y90-Y919, Z811
Mental disorders & substance use	Anorexia nervosa	F500-F501, F504-F505
Mental disorders & substance use	Anxiety disorders	F40-F449, F93-F932
Mental disorders & substance use	Bipolar disorder	F30-F319, F340
Mental disorders & substance use	Depressive disorders	F32-F339, F341
Mental disorders & substance use	Drug use disorders	F11-F1699, F18-F1999, P044-P0449, P961, R781-R789, Z812-Z814
Mental disorders & substance use	Dysthymia	F341
Mental disorders & substance use	Major depression	F32-F339
Mental disorders & substance use	Opioid use disorders	F11-F1199, P961, R781
Mental disorders & substance use	Schizophrenia	F20-F209, F25-F259
Mental disorders & substance use	Self-harm	X60-X649, X66-X849, Y870
Musculoskeletal disorders	Gout	M10-M1019, M103-M109
Musculoskeletal disorders	Low back pain	G544, M47015-M47019, M4715-M4718, M4725-M4728, M47815-M47818, M47896-M47899, M4805-M4808, M4816-M4819, M4825-M4827, M4835-M4838, M4845-M4848, M4855-M4858, M4985-M4988, M5105-M5107, M5115-M5117, M5125-M5127, M5135-M5137, M5145-M5147, M5185-M5187, M533, M5385-M5388, M5405-M5409, M5415-M5418, M543-M545, M9903-M9904, M9913-M9914, M9923-M9924, M9933-M9934, M9943-M9944, M9953-M9954, M9963-M9964, M9973-M9974, M9983-M9984
Musculoskeletal disorders	Lupus	H0112-H01129, L93-L932, M32-M329
Musculoskeletal disorders	Neck pain	G542, M47011-M47013, M4711-M4713, M4721-M4723, M47811-M47813, M47892-M47894, M4801-M4803, M4812-M4814, M4821-M4823, M4831-M4833, M4841-M4843, M4851-M4853, M4981-M4983, M50-M5093, M530-M531, M5381-M5383, M5401-M5403, M5411-M5413, M542, M5481, M9901, M9911, M9921, M9931, M9941, M9951, M9961, M9971, M9981
Musculoskeletal disorders	Osteoarthritis	M16-M189
Musculoskeletal disorders	Rheumatoid arthritis	M05-M069, M080-M0848
Neurological disorders	Alzheimer's disease	G30-G309
Neurological disorders	Alzheimer's disease and other dementias	F00-F020, F028-F0391, F062, G30-G309, G32-G3289
Neurological disorders	Dementia as a sign with others	F00-F009, F02-F020, F028-F0281
Neurological disorders	Idiopathic epilepsy	G40-G419, Z820
Neurological disorders	Motor neuron disease	G12-G129
Neurological disorders	Multiple sclerosis	G35-G359

Appendix Table A1: ICD Code Mapping for Disease Categories (Page 4 of 4)

Group	Diagnosis Category	Code List
Neurological disorders	Parkinson's disease	F023, G20-G209
Neurological disorders	Unspecified dementia	F03-F0391
Neurological disorders	Vascular dementia	F01-F019
Other	Decubitus ulcer	L89-L8995
Other	Endo/metab/blood/immune	D66-D67, D680-D6949, D696-D698, D70-D700, D704-D7581, D7589, D76-D77, D79, D85, D868, D8682-D8684, D8686-D8687, D87-D892, D90-D99, E03-E031, E033-E063, E065-E079, E15-E16, E161-E230, E232-E241, E243, E248-E272, E274-E281, E283-E34, E341-E358, E37-E39, E62, E65-E6609, E662-E7142, E7144-E859, E88-E882, E8830-E889, E90-E999, P721, Z834-Z8349
Other	HIV/AIDS	B20-B249, C46-C469, D80-D849, D898-D899, F024, Z114, Z206, Z21, Z830
Other	Hemoglobinopathies	D55-D59, D591, D593-D595, D598-D630, D638-D649, D68, D699, D759, R580-R589
Other	Psoriasis	L40-L419, M070-M073, M090
Other	Tuberculosis	A10-A14, A15-A199, B90-B909, K230, K673, K930, M490, N740-N741, P370, U843, Z030, Z111, Z201, Z232
Other	Urinary tract infections and interstitial nephritis	N10-N129, N136, N15, N151-N168, N30-N3031, N308-N3091, N34-N343, N390-N392

Appendix Table A2: Multi-morbidity Burden by Income Quartile

Number of disease groups = number of distinct groups in which the patient has ≥1 first-instance diagnosis.
Disease groups: Cancers, Cardiovascular diseases, Chronic respiratory, Diabetes & CKD,
Digestive diseases, Mental disorders & substance use, Musculoskeletal disorders, Neurological disorders, Other.
Each patient is assigned to the income quartile (disposable income, consumption-equivalent, 5-year lagged)
in which they were observed for the most calendar years during 2001–2023; ties broken by lower quartile.
Restricted to the final analytic sample (eligible, valid income, ≥5 matched controls, ≥1 day follow-up).

Income Quartile (modal, study period)	N	% 1 group only	% 2 groups	% 3 groups	% 4+ groups
Q1	2,070,262	32.7	23.8	18.0	25.5
Q2	1,691,617	35.0	24.1	17.7	23.3
Q3	1,538,729	35.9	24.1	17.5	22.5
Q4	1,548,789	38.5	24.4	16.9	20.3
all-income	6,849,397	35.3	24.1	17.6	23.1

Appendix Table A3: Summary Statistics by Income Quartile (Page 1 of 10)

Diagnosis	Group	Quartile	Count	PY	% Cohort	% Female	Mean Age	10-year Survival, % (95% CI)	Median Survival, y (95% CI)	10-year RMST, y (95% CI)
Stomach cancer	Cancers	All	26,047	71,745	100.0	37.1	71.9	16.3 (15.83 - 16.86)	1.0 (0.99 - 0.94)	2.92 (2.87 - 2.97)
Stomach cancer	Cancers	Q1 (Lowest)	7,407	19,108	28.5	37.1	70.9	15.1 (14.22 - 16.1)	0.9 (0.93 - 0.83)	2.78 (2.69 - 2.86)
Stomach cancer	Cancers	Q2	6,667	18,069	25.6	36.4	72.3	16.1 (15.15 - 17.16)	0.9 (0.98 - 0.87)	2.86 (2.77 - 2.95)
Stomach cancer	Cancers	Q3	6,446	18,042	24.7	37.6	72.1	16.3 (15.31 - 17.36)	1.0 (1.06 - 0.95)	2.95 (2.86 - 3.04)
Stomach cancer	Cancers	Q4 (Highest)	5,527	16,525	21.2	37.5	72.7	18.2 (17.07 - 19.38)	1.1 (1.18 - 1.03)	3.15 (3.04 - 3.25)
Lung cancer	Cancers	All	108,917	226,771	100.0	50.4	71.6	11.2 (10.94 - 11.39)	0.7 (0.73 - 0.71)	2.35 (2.33 - 2.37)
Lung cancer	Cancers	Q1 (Lowest)	34,259	64,670	31.4	47.9	70.2	9.8 (9.42 - 10.18)	0.6 (0.66 - 0.63)	2.15 (2.12 - 2.19)
Lung cancer	Cancers	Q2	29,200	58,262	26.8	51.2	71.9	10.0 (9.6 - 10.44)	0.7 (0.72 - 0.68)	2.26 (2.22 - 2.3)
Lung cancer	Cancers	Q3	25,130	54,981	23.1	51.2	72.4	12.0 (11.54 - 12.5)	0.8 (0.79 - 0.74)	2.45 (2.4 - 2.49)
Lung cancer	Cancers	Q4 (Highest)	20,328	48,859	18.7	52.5	72.5	14.0 (13.44 - 14.57)	0.9 (0.88 - 0.83)	2.68 (2.63 - 2.73)
Breast cancer	Cancers	All	189,557	1,652,690	100.0	98.4	64.2	68.2 (67.96 - 68.44)	18.1 (18.24 - 17.92)	8.20 (8.18 - 8.21)
Breast cancer	Cancers	Q1 (Lowest)	43,078	353,546	22.7	98.3	64.2	62.6 (62.08 - 63.13)	15.2 (15.47 - 14.94)	7.83 (7.79 - 7.86)
Breast cancer	Cancers	Q2	46,101	393,644	24.3	98.4	64.1	67.3 (66.77 - 67.76)	17.7 (18.06 - 17.43)	8.13 (8.1 - 8.16)
Breast cancer	Cancers	Q3	48,129	426,013	25.4	98.5	64.2	70.0 (69.56 - 70.49)	18.9 (19.22 - 18.64)	8.31 (8.28 - 8.34)
Breast cancer	Cancers	Q4 (Highest)	52,249	479,486	27.6	98.5	64.3	71.9 (71.48 - 72.36)	19.9 (20.32 - 19.65)	8.45 (8.43 - 8.48)
Prostate cancer	Cancers	All	253,328	1,798,152	100.0	0.1	71.6	53.8 (53.57 - 54.02)	11.1 (11.18 - 11.04)	7.37 (7.35 - 7.38)
Prostate cancer	Cancers	Q1 (Lowest)	52,560	332,560	20.7	0.1	72.4	44.4 (43.92 - 44.92)	8.4 (8.56 - 8.31)	6.69 (6.65 - 6.72)
Prostate cancer	Cancers	Q2	61,751	425,455	24.4	0.1	71.7	52.1 (51.63 - 52.56)	10.6 (10.74 - 10.49)	7.27 (7.24 - 7.3)
Prostate cancer	Cancers	Q3	66,495	482,254	26.2	0.1	71.4	55.9 (55.46 - 56.35)	11.8 (11.91 - 11.62)	7.52 (7.49 - 7.55)
Prostate cancer	Cancers	Q4 (Highest)	72,522	557,883	28.7	0.1	71.2	60.1 (59.65 - 60.48)	13.3 (13.4 - 13.14)	7.80 (7.78 - 7.83)
Colorectal cancer	Cancers	All	158,366	816,928	100.0	48.8	72.1	36.8 (36.51 - 37.06)	5.4 (5.47 - 5.31)	5.51 (5.48 - 5.53)
Colorectal cancer	Cancers	Q1 (Lowest)	40,151	189,593	25.4	48.5	71.9	32.2 (31.7 - 32.77)	4.2 (4.35 - 4.12)	5.07 (5.03 - 5.11)
Colorectal cancer	Cancers	Q2	40,491	204,806	25.5	48.4	72.0	36.1 (35.54 - 36.64)	5.2 (5.32 - 5.02)	5.43 (5.39 - 5.47)
Colorectal cancer	Cancers	Q3	39,634	209,782	25.0	48.9	72.0	38.4 (37.81 - 38.93)	5.9 (6.05 - 5.75)	5.66 (5.62 - 5.71)
Colorectal cancer	Cancers	Q4 (Highest)	38,090	212,747	24.1	49.4	72.3	40.7 (40.08 - 41.23)	6.6 (6.79 - 6.44)	5.87 (5.83 - 5.92)
Pancreatic cancer	Cancers	All	46,014	61,356	100.0	51.4	72.6	6.9 (6.61 - 7.17)	0.4 (0.4 - 0.38)	1.52 (1.5 - 1.55)
Pancreatic cancer	Cancers	Q1 (Lowest)	12,051	15,056	26.1	51.4	71.8	6.1 (5.58 - 6.6)	0.3 (0.34 - 0.32)	1.41 (1.36 - 1.46)
Pancreatic cancer	Cancers	Q2	11,588	14,837	25.2	52.9	72.5	6.9 (6.39 - 7.5)	0.4 (0.38 - 0.35)	1.48 (1.43 - 1.53)
Pancreatic cancer	Cancers	Q3	11,393	15,300	24.8	51.5	73.1	6.6 (6.05 - 7.17)	0.4 (0.44 - 0.4)	1.53 (1.48 - 1.59)
Pancreatic cancer	Cancers	Q4 (Highest)	10,982	16,164	23.9	49.8	73.2	8.0 (7.44 - 8.65)	0.5 (0.49 - 0.45)	1.68 (1.62 - 1.73)
Ischemic heart disease	Cardiovascular diseases	All	812,154	6,033,257	100.0	41.9	71.8	50.2 (50.04 - 50.28)	10.1 (10.09 - 10.01)	6.87 (6.86 - 6.88)
Ischemic heart disease	Cardiovascular diseases	Q1 (Lowest)	231,612	1,642,771	28.5	42.5	70.7	46.9 (46.71 - 47.17)	9.0 (9.1 - 8.98)	6.63 (6.61 - 6.65)
Ischemic heart disease	Cardiovascular diseases	Q2	212,051	1,562,508	26.1	42.3	71.9	49.2 (48.99 - 49.46)	9.8 (9.84 - 9.7)	6.80 (6.79 - 6.82)
Ischemic heart disease	Cardiovascular diseases	Q3	197,066	1,494,488	24.3	41.6	72.3	51.6 (51.34 - 51.83)	10.5 (10.61 - 10.45)	6.97 (6.95 - 6.99)
Ischemic heart disease	Cardiovascular diseases	Q4 (Highest)	171,425	1,333,490	21.1	40.7	72.5	54.0 (53.77 - 54.3)	11.3 (11.38 - 11.2)	7.18 (7.16 - 7.2)
Stroke	Cardiovascular diseases	All	781,926	4,875,371	100.0	49.0	73.2	42.1 (42.02 - 42.27)	7.7 (7.76 - 7.7)	6.27 (6.26 - 6.28)
Stroke	Cardiovascular diseases	Q1 (Lowest)	217,136	1,284,787	27.8	48.7	72.0	38.5 (38.21 - 38.68)	6.8 (6.89 - 6.78)	5.98 (5.97 - 6.0)
Stroke	Cardiovascular diseases	Q2	197,820	1,222,169	25.3	49.4	73.1	41.5 (41.25 - 41.75)	7.5 (7.61 - 7.48)	6.21 (6.19 - 6.23)
Stroke	Cardiovascular diseases	Q3	188,618	1,194,748	24.1	49.1	73.7	43.4 (43.12 - 43.63)	8.0 (8.1 - 7.96)	6.35 (6.33 - 6.37)
Stroke	Cardiovascular diseases	Q4 (Highest)	178,352	1,173,667	22.8	48.8	74.1	46.1 (45.82 - 46.35)	8.8 (8.91 - 8.76)	6.59 (6.57 - 6.61)
Hypertensive heart disease	Cardiovascular diseases	All	69,273	543,564	100.0	44.6	72.5	44.8 (44.42 - 45.21)	8.5 (8.59 - 8.38)	6.61 (6.58 - 6.63)
Hypertensive heart disease	Cardiovascular diseases	Q1 (Lowest)	19,705	146,030	28.5	45.8	71.4	41.5 (40.73 - 42.19)	7.6 (7.82 - 7.44)	6.38 (6.32 - 6.43)
Hypertensive heart disease	Cardiovascular diseases	Q2	17,901	138,670	25.8	45.5	72.5	43.8 (43.0 - 44.54)	8.1 (8.36 - 7.9)	6.50 (6.45 - 6.56)
Hypertensive heart disease	Cardiovascular diseases	Q3	16,835	136,290	24.3	44.8	72.9	46.4 (45.6 - 47.19)	8.9 (9.15 - 8.67)	6.69 (6.64 - 6.75)
Hypertensive heart disease	Cardiovascular diseases	Q4 (Highest)	14,832	122,572	21.4	42.0	73.3	48.8 (47.9 - 49.62)	9.7 (9.89 - 9.38)	6.94 (6.88 - 7.0)
Atrial fibrillation	Cardiovascular diseases	All	814,748	4,810,247	100.0	45.0	75.1	39.8 (39.67 - 39.92)	7.2 (7.27 - 7.21)	6.21 (6.2 - 6.22)
Atrial fibrillation	Cardiovascular diseases	Q1 (Lowest)	210,947	1,141,230	25.9	46.2	74.9	33.8 (33.6 - 34.08)	6.0 (6.05 - 5.95)	5.75 (5.74 - 5.77)
Atrial fibrillation	Cardiovascular diseases	Q2	206,173	1,185,875	25.3	45.6	75.1	38.5 (38.22 - 38.72)	6.9 (6.93 - 6.83)	6.08 (6.06 - 6.1)
Atrial fibrillation	Cardiovascular diseases	Q3	201,193	1,209,975	24.7	44.8	75.3	41.3 (41.02 - 41.53)	7.6 (7.66 - 7.53)	6.32 (6.31 - 6.34)
Atrial fibrillation	Cardiovascular diseases	Q4 (Highest)	196,435	1,273,166	24.1	43.2	75.1	46.0 (45.79 - 46.3)	8.9 (8.94 - 8.81)	6.71 (6.69 - 6.73)

Appendix Table A3: Summary Statistics by Income Quartile (Page 2 of 10)

Diagnosis	Group	Quartile	Count	PY	% Cohort	% Female	Mean Age	10-year Survival, % (95% CI)	Median Survival, y (95% CI)	10-year RMST, y (95% CI)
COPD	Chronic respiratory	All	373,459	2,275,492	100.0	51.9	71.2	39.0 (38.82 - 39.18)	7.0 (7.08 - 7.0)	6.14 (6.13 - 6.15)
COPD	Chronic respiratory	Q1 (Lowest)	128,490	759,372	34.4	49.8	68.9	38.2 (37.87 - 38.49)	6.8 (6.91 - 6.77)	6.07 (6.05 - 6.09)
COPD	Chronic respiratory	Q2	99,330	596,948	26.6	52.9	71.7	38.0 (37.6 - 38.3)	6.9 (6.93 - 6.78)	6.07 (6.04 - 6.09)
COPD	Chronic respiratory	Q3	81,702	506,670	21.9	52.8	72.7	39.4 (39.01 - 39.77)	7.1 (7.19 - 7.0)	6.16 (6.14 - 6.19)
COPD	Chronic respiratory	Q4 (Highest)	63,937	412,501	17.1	53.6	73.3	41.7 (41.28 - 42.15)	7.7 (7.83 - 7.62)	6.36 (6.33 - 6.39)
Alzheimer's disease and other dementias	Neurological disorders	All	374,264	1,175,121	100.0	58.5	81.9	7.6 (7.54 - 7.75)	2.7 (2.72 - 2.69)	3.52 (3.51 - 3.53)
Alzheimer's disease and other dementias	Neurological disorders	Q1 (Lowest)	101,123	312,755	27.0	57.3	80.4	8.4 (8.22 - 8.64)	2.6 (2.61 - 2.56)	3.48 (3.45 - 3.5)
Alzheimer's disease and other dementias	Neurological disorders	Q2	93,871	286,502	25.1	58.3	81.9	7.2 (7.01 - 7.41)	2.6 (2.61 - 2.55)	3.42 (3.4 - 3.44)
Alzheimer's disease and other dementias	Neurological disorders	Q3	90,659	283,308	24.2	58.7	82.4	7.0 (6.85 - 7.26)	2.7 (2.75 - 2.69)	3.51 (3.49 - 3.53)
Alzheimer's disease and other dementias	Neurological disorders	Q4 (Highest)	88,611	292,555	23.7	59.9	82.9	7.8 (7.63 - 8.07)	3.0 (3.01 - 2.95)	3.70 (3.68 - 3.73)
Diabetes	Diabetes & CKD	All	749,652	6,019,606	100.0	43.5	66.1	59.4 (59.31 - 59.57)	13.1 (13.15 - 13.06)	7.67 (7.66 - 7.68)
Diabetes	Diabetes & CKD	Q1 (Lowest)	242,652	1,863,474	32.3	45.2	64.7	57.7 (57.45 - 57.9)	12.4 (12.51 - 12.33)	7.58 (7.57 - 7.59)
Diabetes	Diabetes & CKD	Q2	196,081	1,557,698	26.2	44.3	66.6	58.3 (58.09 - 58.59)	12.7 (12.74 - 12.56)	7.60 (7.59 - 7.62)
Diabetes	Diabetes & CKD	Q3	170,640	1,404,954	22.8	42.7	66.8	60.5 (60.19 - 60.72)	13.5 (13.63 - 13.42)	7.71 (7.69 - 7.73)
Diabetes	Diabetes & CKD	Q4 (Highest)	140,279	1,193,480	18.7	40.2	67.0	62.7 (62.4 - 62.98)	14.5 (14.63 - 14.39)	7.85 (7.84 - 7.87)
Chronic kidney disease	Diabetes & CKD	All	456,498	1,913,543	100.0	41.1	73.5	28.9 (28.71 - 29.03)	3.8 (3.82 - 3.76)	4.76 (4.75 - 4.77)
Chronic kidney disease	Diabetes & CKD	Q1 (Lowest)	139,906	557,720	30.6	41.1	71.8	26.9 (26.58 - 27.16)	3.6 (3.65 - 3.55)	4.63 (4.61 - 4.65)
Chronic kidney disease	Diabetes & CKD	Q2	117,675	487,866	25.8	41.9	73.8	28.2 (27.93 - 28.56)	3.7 (3.73 - 3.62)	4.70 (4.68 - 4.73)
Chronic kidney disease	Diabetes & CKD	Q3	105,272	447,359	23.1	41.4	74.4	29.6 (29.29 - 29.96)	3.8 (3.83 - 3.72)	4.78 (4.76 - 4.81)
Chronic kidney disease	Diabetes & CKD	Q4 (Highest)	93,645	420,598	20.5	39.9	74.6	31.7 (31.36 - 32.08)	4.2 (4.31 - 4.17)	4.99 (4.96 - 5.02)
Esophageal cancer	Cancers	All	15,232	30,191	100.0	27.8	71.1	10.3 (9.8 - 10.93)	0.7 (0.76 - 0.72)	2.17 (2.12 - 2.22)
Esophageal cancer	Cancers	Q1 (Lowest)	4,616	8,198	30.3	27.8	70.0	8.7 (7.81 - 9.71)	0.7 (0.68 - 0.62)	1.95 (1.86 - 2.04)
Esophageal cancer	Cancers	Q2	3,947	7,721	25.9	26.6	71.2	10.3 (9.24 - 11.46)	0.7 (0.77 - 0.69)	2.17 (2.06 - 2.27)
Esophageal cancer	Cancers	Q3	3,569	7,261	23.4	27.6	71.6	10.6 (9.41 - 11.78)	0.8 (0.84 - 0.75)	2.23 (2.12 - 2.34)
Esophageal cancer	Cancers	Q4 (Highest)	3,100	7,011	20.4	29.5	72.2	12.5 (11.24 - 13.91)	0.8 (0.89 - 0.78)	2.42 (2.3 - 2.55)
Liver cancer	Cancers	All	16,240	31,243	100.0	35.1	69.5	11.1 (10.5 - 11.7)	0.7 (0.72 - 0.66)	2.28 (2.23 - 2.33)
Liver cancer	Cancers	Q1 (Lowest)	5,732	10,917	35.2	31.6	67.6	11.6 (10.54 - 12.6)	0.7 (0.73 - 0.63)	2.31 (2.22 - 2.4)
Liver cancer	Cancers	Q2	3,991	7,240	24.6	36.4	70.2	10.6 (9.44 - 11.85)	0.7 (0.73 - 0.61)	2.22 (2.11 - 2.32)
Liver cancer	Cancers	Q3	3,421	6,559	21.1	37.0	70.7	10.9 (9.69 - 12.23)	0.7 (0.73 - 0.62)	2.22 (2.11 - 2.34)
Liver cancer	Cancers	Q4 (Highest)	3,096	6,528	19.1	37.9	71.0	11.2 (9.86 - 12.57)	0.8 (0.86 - 0.7)	2.38 (2.26 - 2.5)
Larynx cancer	Cancers	All	5,702	35,727	100.0	21.4	68.8	37.8 (36.43 - 39.23)	6.3 (6.8 - 5.97)	5.87 (5.77 - 5.98)
Larynx cancer	Cancers	Q1 (Lowest)	2,102	11,627	36.9	20.3	67.5	31.1 (28.92 - 33.37)	5.1 (5.6 - 4.63)	5.38 (5.2 - 5.55)
Larynx cancer	Cancers	Q2	1,438	9,299	25.2	22.0	69.0	40.1 (37.34 - 42.94)	6.9 (7.92 - 6.07)	5.97 (5.76 - 6.19)
Larynx cancer	Cancers	Q3	1,145	7,437	20.1	21.1	70.0	40.0 (36.81 - 43.14)	7.1 (7.81 - 6.02)	6.09 (5.86 - 6.32)
Larynx cancer	Cancers	Q4 (Highest)	1,017	7,364	17.8	23.1	69.7	45.6 (42.21 - 48.94)	8.9 (9.65 - 7.76)	6.49 (6.24 - 6.74)
Cervical cancer	Cancers	All	15,595	127,327	100.0	99.9	55.6	61.6 (60.74 - 62.41)	19.2 (19.99 - 18.32)	7.22 (7.16 - 7.28)
Cervical cancer	Cancers	Q1 (Lowest)	4,334	33,317	27.8	100.0	56.8	57.0 (55.34 - 58.54)	15.5 (16.62 - 14.15)	6.82 (6.69 - 6.94)
Cervical cancer	Cancers	Q2	3,942	31,802	25.3	99.8	55.8	60.8 (59.06 - 62.42)	17.7 (20.35 - 16.33)	7.21 (7.08 - 7.33)
Cervical cancer	Cancers	Q3	3,759	31,350	24.1	100.0	54.9	64.7 (63.0 - 66.37)	22.2	7.46 (7.33 - 7.58)
Cervical cancer	Cancers	Q4 (Highest)	3,560	30,858	22.8	99.9	54.7	64.9 (63.13 - 66.54)	22.8	7.47 (7.34 - 7.6)
Uterine cancer	Cancers	All	39,372	305,778	100.0	99.9	69.2	57.2 (56.67 - 57.76)	13.1 (13.32 - 12.84)	7.24 (7.2 - 7.28)
Uterine cancer	Cancers	Q1 (Lowest)	10,258	74,690	26.1	99.9	68.6	53.4 (52.3 - 54.48)	11.4 (11.82 - 11.0)	6.97 (6.89 - 7.04)
Uterine cancer	Cancers	Q2	9,799	75,623	24.9	99.9	69.2	56.4 (55.27 - 57.47)	12.8 (13.3 - 12.18)	7.19 (7.11 - 7.26)
Uterine cancer	Cancers	Q3	9,694	76,342	24.6	99.9	69.3	58.5 (57.35 - 59.55)	13.4 (13.99 - 13.09)	7.33 (7.25 - 7.4)
Uterine cancer	Cancers	Q4 (Highest)	9,621	79,123	24.4	100.0	69.6	60.9 (59.75 - 61.93)	14.6 (15.02 - 14.05)	7.50 (7.42 - 7.57)
Lip oral cavity cancer	Cancers	All	29,800	194,581	100.0	48.6	68.4	47.9 (47.24 - 48.55)	9.2 (9.42 - 8.98)	6.60 (6.55 - 6.64)
Lip oral cavity cancer	Cancers	Q1 (Lowest)	7,964	46,448	26.7	46.1	67.7	39.8 (38.57 - 41.04)	6.6 (6.99 - 6.31)	5.89 (5.8 - 5.98)
Lip oral cavity cancer	Cancers	Q2	7,290	47,124	24.5	49.0	68.6	47.6 (46.3 - 48.94)	9.1 (9.51 - 8.66)	6.56 (6.46 - 6.65)
Lip oral cavity cancer	Cancers	Q3	7,303	49,617	24.5	49.6	68.6	51.0 (49.66 - 52.33)	10.5 (11.05 - 9.87)	6.89 (6.8 - 6.99)
Lip oral cavity cancer	Cancers	Q4 (Highest)	7,243	51,392	24.3	50.0	68.8	54.0 (52.69 - 55.36)	11.7 (12.34 - 11.11)	7.12 (7.03 - 7.21)

Appendix Table A3: Summary Statistics by Income Quartile (Page 3 of 10)

Diagnosis	Group	Quartile	Count	PY	% Cohort	% Female	Mean Age	10-year Survival, % (95% CI)	Median Survival, y (95% CI)	10-year RMST, y (95% CI)
Nasopharynx cancer	Cancers	All	1,728	11,248	100.0	37.8	62.4	42.8 (40.26 - 45.36)	6.8 (7.84 - 5.76)	5.92 (5.72 - 6.12)
Nasopharynx cancer	Cancers	Q1 (Lowest)	559	3,344	32.4	40.4	60.7	41.1 (36.53 - 45.6)	6.6 (8.02 - 4.86)	5.79 (5.43 - 6.14)
Nasopharynx cancer	Cancers	Q2	443	2,897	25.6	37.2	62.5	43.3 (38.19 - 48.35)	6.6 (9.33 - 5.16)	5.99 (5.6 - 6.38)
Nasopharynx cancer	Cancers	Q3	389	2,670	22.5	36.8	62.7	45.0 (39.63 - 50.23)	7.2 (10.08 - 5.33)	6.06 (5.64 - 6.47)
Nasopharynx cancer	Cancers	Q4 (Highest)	337	2,336	19.5	35.3	64.7	42.4 (36.67 - 47.9)	6.8 (8.78 - 5.08)	5.89 (5.44 - 6.33)
Other pharynx cancer	Cancers	All	10,698	64,264	100.0	31.7	64.5	45.5 (44.41 - 46.56)	8.0 (8.37 - 7.52)	6.09 (6.0 - 6.17)
Other pharynx cancer	Cancers	Q1 (Lowest)	3,318	16,836	31.0	30.2	64.3	34.0 (32.23 - 35.87)	4.0 (4.41 - 3.58)	5.08 (4.93 - 5.22)
Other pharynx cancer	Cancers	Q2	2,632	16,014	24.6	32.8	64.7	46.7 (44.56 - 48.89)	8.3 (9.27 - 7.32)	6.17 (6.01 - 6.34)
Other pharynx cancer	Cancers	Q3	2,385	15,102	22.3	32.4	64.5	50.6 (48.28 - 52.89)	10.2 (11.61 - 9.17)	6.50 (6.33 - 6.67)
Other pharynx cancer	Cancers	Q4 (Highest)	2,363	16,313	22.1	32.1	64.8	55.4 (53.02 - 57.62)	12.5 (13.81 - 10.97)	7.00 (6.83 - 7.16)
Gallbladder cancer	Cancers	All	17,199	27,906	100.0	57.7	72.5	8.6 (8.08 - 9.06)	0.5 (0.5 - 0.46)	1.79 (1.74 - 1.83)
Gallbladder cancer	Cancers	Q1 (Lowest)	4,743	6,807	27.5	58.8	72.2	7.5 (6.6 - 8.36)	0.4 (0.42 - 0.36)	1.59 (1.51 - 1.68)
Gallbladder cancer	Cancers	Q2	4,491	7,088	26.1	57.9	72.4	8.0 (7.08 - 8.94)	0.5 (0.49 - 0.43)	1.73 (1.64 - 1.82)
Gallbladder cancer	Cancers	Q3	4,138	7,094	24.1	57.8	72.7	9.0 (8.0 - 10.06)	0.5 (0.57 - 0.51)	1.90 (1.8 - 2.0)
Gallbladder cancer	Cancers	Q4 (Highest)	3,827	6,917	22.3	56.1	72.9	10.1 (9.08 - 11.28)	0.6 (0.62 - 0.54)	1.97 (1.86 - 2.07)
Melanoma	Cancers	All	93,519	673,408	100.0	49.3	64.8	65.6 (65.24 - 65.98)	18.0 (18.29 - 17.63)	7.94 (7.91 - 7.96)
Melanoma	Cancers	Q1 (Lowest)	16,032	109,730	17.1	50.4	65.9	57.6 (56.67 - 58.49)	13.2 (13.7 - 12.68)	7.34 (7.28 - 7.4)
Melanoma	Cancers	Q2	21,839	153,099	23.4	49.3	64.5	64.5 (63.69 - 65.23)	17.4 (18.11 - 16.76)	7.84 (7.79 - 7.89)
Melanoma	Cancers	Q3	25,611	184,346	27.4	49.5	64.3	67.3 (66.57 - 67.97)	18.8 (19.25 - 18.1)	8.04 (8.0 - 8.09)
Melanoma	Cancers	Q4 (Highest)	30,037	226,234	32.1	48.5	64.7	69.4 (68.76 - 70.03)	20.0 (20.64 - 19.41)	8.24 (8.2 - 8.28)
Ovarian cancer	Cancers	All	26,798	157,313	100.0	99.9	65.3	35.7 (35.05 - 36.31)	4.3 (4.43 - 4.17)	5.26 (5.21 - 5.31)
Ovarian cancer	Cancers	Q1 (Lowest)	6,525	35,964	24.3	99.9	64.9	34.5 (33.2 - 35.74)	3.9 (4.15 - 3.63)	5.07 (4.97 - 5.18)
Ovarian cancer	Cancers	Q2	6,612	38,078	24.7	99.8	65.5	34.7 (33.47 - 35.97)	4.0 (4.27 - 3.8)	5.16 (5.06 - 5.26)
Ovarian cancer	Cancers	Q3	6,836	40,339	25.5	99.9	65.3	35.3 (34.02 - 36.49)	4.3 (4.56 - 4.04)	5.26 (5.16 - 5.36)
Ovarian cancer	Cancers	Q4 (Highest)	6,825	42,932	25.5	99.9	65.4	38.2 (36.95 - 39.47)	5.0 (5.31 - 4.72)	5.54 (5.44 - 5.64)
Testicular cancer	Cancers	All	10,313	112,889	100.0	0.1	41.6	88.0 (87.34 - 88.71)	NR	9.21 (9.16 - 9.26)
Testicular cancer	Cancers	Q1 (Lowest)	2,136	22,939	20.7	0.1	41.9	84.5 (82.73 - 86.08)	NR	8.96 (8.84 - 9.07)
Testicular cancer	Cancers	Q2	2,576	27,773	25.0	0.1	41.4	88.2 (86.75 - 89.51)	NR	9.24 (9.15 - 9.34)
Testicular cancer	Cancers	Q3	2,761	29,948	26.8	0.1	41.7	88.2 (86.77 - 89.44)	NR	9.24 (9.15 - 9.33)
Testicular cancer	Cancers	Q4 (Highest)	2,840	32,229	27.5	0.1	41.5	90.4 (89.2 - 91.55)	NR	9.35 (9.26 - 9.43)
Kidney cancer	Cancers	All	39,633	205,590	100.0	38.6	70.2	38.1 (37.56 - 38.68)	5.8 (5.95 - 5.62)	5.55 (5.51 - 5.59)
Kidney cancer	Cancers	Q1 (Lowest)	10,677	51,501	26.9	39.8	69.3	35.3 (34.26 - 36.4)	5.0 (5.29 - 4.74)	5.28 (5.2 - 5.37)
Kidney cancer	Cancers	Q2	10,180	52,184	25.7	39.0	70.2	37.7 (36.58 - 38.79)	5.8 (6.1 - 5.42)	5.54 (5.45 - 5.62)
Kidney cancer	Cancers	Q3	9,751	51,956	24.6	38.3	70.7	39.6 (38.5 - 40.75)	6.2 (6.45 - 5.86)	5.67 (5.58 - 5.75)
Kidney cancer	Cancers	Q4 (Highest)	9,025	49,949	22.8	37.2	70.9	40.2 (39.05 - 41.39)	6.5 (6.78 - 6.09)	5.76 (5.67 - 5.84)
Bladder cancer	Cancers	All	71,838	427,958	100.0	25.7	73.2	41.7 (41.24 - 42.08)	7.5 (7.61 - 7.39)	6.17 (6.14 - 6.2)
Bladder cancer	Cancers	Q1 (Lowest)	18,216	100,469	25.4	26.0	72.3	37.6 (36.81 - 38.46)	6.4 (6.54 - 6.15)	5.80 (5.74 - 5.86)
Bladder cancer	Cancers	Q2	18,325	107,259	25.4	25.9	73.2	40.5 (39.68 - 41.35)	7.3 (7.45 - 7.05)	6.12 (6.06 - 6.18)
Bladder cancer	Cancers	Q3	18,089	109,975	25.2	25.3	73.6	42.6 (41.76 - 43.43)	7.7 (7.99 - 7.52)	6.25 (6.19 - 6.31)
Bladder cancer	Cancers	Q4 (Highest)	17,208	110,256	24.0	25.5	73.9	46.1 (45.22 - 46.95)	8.8 (9.07 - 8.58)	6.53 (6.47 - 6.59)
Brain cancer	Cancers	All	28,259	100,210	100.0	45.7	62.3	21.7 (21.17 - 22.22)	1.1 (1.1 - 1.05)	3.32 (3.27 - 3.36)
Brain cancer	Cancers	Q1 (Lowest)	6,835	23,755	24.2	46.3	62.0	21.2 (20.15 - 22.27)	1.0 (1.06 - 0.94)	3.26 (3.16 - 3.35)
Brain cancer	Cancers	Q2	7,164	25,635	25.4	46.1	62.2	22.0 (20.93 - 23.0)	1.1 (1.11 - 0.99)	3.31 (3.22 - 3.41)
Brain cancer	Cancers	Q3	7,087	24,970	25.1	45.1	62.3	21.5 (20.43 - 22.51)	1.1 (1.15 - 1.05)	3.30 (3.2 - 3.39)
Brain cancer	Cancers	Q4 (Highest)	7,173	25,850	25.3	45.4	62.7	22.1 (21.09 - 23.19)	1.1 (1.21 - 1.09)	3.40 (3.3 - 3.49)
Thyroid cancer	Cancers	All	14,590	122,058	100.0	71.5	56.2	72.3 (71.48 - 73.15)	NR	8.12 (8.06 - 8.18)
Thyroid cancer	Cancers	Q1 (Lowest)	3,862	31,223	26.5	72.6	55.4	70.9 (69.26 - 72.54)	NR	8.00 (7.88 - 8.12)
Thyroid cancer	Cancers	Q2	3,602	29,495	24.7	70.8	56.2	71.2 (69.47 - 72.88)	NR	8.06 (7.94 - 8.18)
Thyroid cancer	Cancers	Q3	3,576	29,890	24.5	71.5	56.7	72.8 (71.08 - 74.43)	NR	8.15 (8.03 - 8.27)
Thyroid cancer	Cancers	Q4 (Highest)	3,550	31,450	24.3	70.8	56.4	74.5 (72.78 - 76.04)	NR	8.29 (8.17 - 8.4)

Appendix Table A3: Summary Statistics by Income Quartile (Page 4 of 10)

Diagnosis	Group	Quartile	Count	PY	% Cohort	% Female	Mean Age	10-year Survival, % (95% CI)	Median Survival, y (95% CI)	10-year RMST, y (95% CI)
Hodgkin lymphoma	Cancers	All	8,346	76,694	100.0	45.6	52.6	67.0 (65.86 - 68.07)	22.1	7.73 (7.65 - 7.82)
Hodgkin lymphoma	Cancers	Q1 (Lowest)	2,104	18,174	25.2	46.2	52.4	64.4 (62.05 - 66.58)	19.9	7.56 (7.4 - 7.73)
Hodgkin lymphoma	Cancers	Q2	2,035	19,100	24.4	45.1	52.2	67.0 (64.75 - 69.2)	21.8	7.75 (7.59 - 7.91)
Hodgkin lymphoma	Cancers	Q3	2,036	18,688	24.4	46.6	52.6	67.2 (64.93 - 69.39)	23.0	7.73 (7.57 - 7.9)
Hodgkin lymphoma	Cancers	Q4 (Highest)	2,171	20,732	26.0	44.5	53.3	69.2 (67.02 - 71.25)	22.9	7.89 (7.73 - 8.04)
Lymphoma	Cancers	All	50,944	316,602	100.0	44.5	67.9	44.6 (44.15 - 45.14)	8.0 (8.13 - 7.8)	6.17 (6.13 - 6.21)
Lymphoma	Cancers	Q1 (Lowest)	12,502	72,534	24.5	45.2	67.1	41.0 (39.96 - 41.93)	6.7 (7.07 - 6.41)	5.82 (5.75 - 5.9)
Lymphoma	Cancers	Q2	12,658	77,066	24.8	44.4	67.8	43.7 (42.67 - 44.66)	7.8 (8.11 - 7.42)	6.12 (6.05 - 6.2)
Lymphoma	Cancers	Q3	12,830	81,741	25.2	44.8	68.1	46.0 (44.97 - 46.94)	8.3 (8.63 - 7.96)	6.27 (6.2 - 6.34)
Lymphoma	Cancers	Q4 (Highest)	12,954	85,261	25.5	43.8	68.4	47.9 (46.87 - 48.83)	9.1 (9.48 - 8.75)	6.45 (6.38 - 6.52)
Myeloma	Cancers	All	29,132	134,740	100.0	43.5	71.5	28.0 (27.37 - 28.64)	4.7 (4.84 - 4.62)	5.27 (5.22 - 5.32)
Myeloma	Cancers	Q1 (Lowest)	6,957	29,440	23.9	44.7	71.0	24.1 (22.85 - 25.36)	4.0 (4.19 - 3.88)	4.88 (4.78 - 4.97)
Myeloma	Cancers	Q2	7,052	31,946	24.2	43.6	71.3	27.9 (26.64 - 29.22)	4.6 (4.83 - 4.44)	5.19 (5.1 - 5.29)
Myeloma	Cancers	Q3	7,149	33,054	24.5	43.3	71.7	28.2 (26.89 - 29.48)	4.8 (5.02 - 4.58)	5.29 (5.2 - 5.39)
Myeloma	Cancers	Q4 (Highest)	7,974	40,300	27.4	42.4	72.0	31.3 (30.04 - 32.53)	5.5 (5.68 - 5.23)	5.66 (5.57 - 5.75)
Leukemia	Cancers	All	44,601	235,656	100.0	42.4	69.1	35.9 (35.38 - 36.4)	5.2 (5.3 - 5.03)	5.36 (5.32 - 5.4)
Leukemia	Cancers	Q1 (Lowest)	10,940	52,295	24.5	43.2	68.8	31.0 (29.98 - 31.98)	3.9 (4.14 - 3.68)	4.90 (4.82 - 4.98)
Leukemia	Cancers	Q2	11,150	57,645	25.0	41.6	68.9	34.7 (33.7 - 35.74)	4.9 (5.16 - 4.66)	5.27 (5.19 - 5.35)
Leukemia	Cancers	Q3	11,082	59,658	24.8	42.9	69.3	37.4 (36.33 - 38.4)	5.5 (5.85 - 5.2)	5.47 (5.39 - 5.55)
Leukemia	Cancers	Q4 (Highest)	11,429	66,058	25.7	42.0	69.5	40.3 (39.26 - 41.32)	6.5 (6.88 - 6.22)	5.79 (5.71 - 5.87)
Malignant neoplasm of bone & articular cartilage	Cancers	All	7,832	39,885	100.0	44.5	63.3	31.6 (30.5 - 32.72)	2.6 (2.77 - 2.33)	4.50 (4.4 - 4.6)
Malignant neoplasm of bone & articular cartilage	Cancers	Q1 (Lowest)	2,052	9,760	26.2	45.2	63.0	27.6 (25.58 - 29.74)	1.9 (2.17 - 1.61)	4.13 (3.94 - 4.31)
Malignant neoplasm of bone & articular cartilage	Cancers	Q2	1,954	9,783	24.9	44.6	63.2	30.7 (28.49 - 32.89)	2.5 (2.92 - 2.1)	4.44 (4.25 - 4.64)
Malignant neoplasm of bone & articular cartilage	Cancers	Q3	1,939	10,043	24.8	45.5	63.6	34.0 (31.7 - 36.28)	3.1 (3.53 - 2.6)	4.72 (4.53 - 4.92)
Malignant neoplasm of bone & articular cartilage	Cancers	Q4 (Highest)	1,887	10,298	24.1	42.8	63.5	34.5 (32.24 - 36.83)	3.0 (3.57 - 2.5)	4.74 (4.54 - 4.93)
Rheumatic heart disease	Cardiovascular diseases	All	31,613	154,301	100.0	57.2	73.7	36.5 (35.84 - 37.19)	5.9 (6.06 - 5.78)	5.75 (5.7 - 5.8)
Rheumatic heart disease	Cardiovascular diseases	Q1 (Lowest)	9,114	45,177	28.8	58.6	70.5	36.8 (35.52 - 38.01)	5.9 (6.15 - 5.62)	5.74 (5.65 - 5.83)
Rheumatic heart disease	Cardiovascular diseases	Q2	7,898	37,491	25.0	57.2	74.0	35.5 (34.1 - 36.8)	5.7 (5.92 - 5.39)	5.65 (5.55 - 5.74)
Rheumatic heart disease	Cardiovascular diseases	Q3	7,267	34,362	23.0	56.6	75.4	35.9 (34.5 - 37.33)	5.7 (6.01 - 5.38)	5.67 (5.57 - 5.77)
Rheumatic heart disease	Cardiovascular diseases	Q4 (Highest)	7,334	37,271	23.2	56.1	75.7	37.9 (36.53 - 39.35)	6.5 (6.87 - 6.19)	5.97 (5.87 - 6.06)
Cardiomyopathy	Cardiovascular diseases	All	85,014	610,789	100.0	35.3	60.3	60.8 (60.44 - 61.23)	14.5 (14.68 - 14.26)	7.64 (7.62 - 7.67)
Cardiomyopathy	Cardiovascular diseases	Q1 (Lowest)	23,932	160,431	28.2	35.5	60.7	54.1 (53.38 - 54.9)	11.5 (11.75 - 11.21)	7.22 (7.17 - 7.27)
Cardiomyopathy	Cardiovascular diseases	Q2	21,522	156,137	25.3	35.5	60.2	60.9 (60.13 - 61.68)	14.7 (15.15 - 14.25)	7.63 (7.58 - 7.68)
Cardiomyopathy	Cardiovascular diseases	Q3	20,230	148,802	23.8	34.9	59.9	64.1 (63.28 - 64.87)	16.0 (16.58 - 15.57)	7.86 (7.81 - 7.91)
Cardiomyopathy	Cardiovascular diseases	Q4 (Highest)	19,330	145,420	22.7	35.4	60.5	65.7 (64.85 - 66.45)	16.9 (17.44 - 16.5)	7.96 (7.91 - 8.01)
Peripheral artery	Cardiovascular diseases	All	193,190	1,129,782	100.0	49.0	73.2	35.5 (35.31 - 35.8)	6.4 (6.41 - 6.3)	5.89 (5.87 - 5.9)
Peripheral artery	Cardiovascular diseases	Q1 (Lowest)	58,200	321,018	30.1	46.2	72.0	32.6 (32.19 - 33.07)	5.8 (5.88 - 5.7)	5.66 (5.63 - 5.7)
Peripheral artery	Cardiovascular diseases	Q2	51,885	303,896	26.9	49.3	73.1	35.7 (35.27 - 36.22)	6.4 (6.49 - 6.27)	5.89 (5.85 - 5.92)
Peripheral artery	Cardiovascular diseases	Q3	45,826	274,781	23.7	50.8	74.0	36.5 (36.01 - 37.02)	6.6 (6.71 - 6.5)	5.96 (5.93 - 6.0)
Peripheral artery	Cardiovascular diseases	Q4 (Highest)	37,279	230,087	19.3	50.8	74.3	38.6 (38.04 - 39.17)	7.1 (7.2 - 6.94)	6.13 (6.09 - 6.17)
Endocarditis	Cardiovascular diseases	All	23,046	118,033	100.0	36.5	69.0	36.3 (35.61 - 37.06)	5.6 (5.77 - 5.4)	5.44 (5.39 - 5.5)
Endocarditis	Cardiovascular diseases	Q1 (Lowest)	7,292	35,360	31.6	38.3	65.5	34.7 (33.4 - 35.98)	5.0 (5.35 - 4.68)	5.26 (5.16 - 5.36)
Endocarditis	Cardiovascular diseases	Q2	5,774	29,523	25.1	36.8	69.2	36.0 (34.55 - 37.45)	5.5 (5.91 - 5.23)	5.44 (5.32 - 5.55)
Endocarditis	Cardiovascular diseases	Q3	5,094	27,116	22.1	35.3	71.0	37.3 (35.71 - 38.8)	5.9 (6.29 - 5.59)	5.55 (5.43 - 5.67)
Endocarditis	Cardiovascular diseases	Q4 (Highest)	4,886	26,034	21.2	34.6	72.0	38.2 (36.62 - 39.79)	6.0 (6.45 - 5.66)	5.61 (5.48 - 5.73)
Heart failure	Cardiovascular diseases	All	708,074	2,943,435	100.0	47.7	78.1	21.6 (21.47 - 21.7)	3.7 (3.67 - 3.64)	4.54 (4.53 - 4.55)
Heart failure	Cardiovascular diseases	Q1 (Lowest)	212,381	860,670	30.0	47.1	76.3	20.5 (20.32 - 20.73)	3.5 (3.53 - 3.47)	4.44 (4.43 - 4.46)
Heart failure	Cardiovascular diseases	Q2	185,206	759,291	26.2	48.4	78.1	21.0 (20.82 - 21.26)	3.6 (3.6 - 3.53)	4.48 (4.47 - 4.5)
Heart failure	Cardiovascular diseases	Q3	166,462	695,432	23.5	48.2	79.0	21.8 (21.59 - 22.06)	3.7 (3.72 - 3.65)	4.56 (4.54 - 4.58)
Heart failure	Cardiovascular diseases	Q4 (Highest)	144,025	628,041	20.3	47.3	79.5	23.6 (23.34 - 23.86)	4.0 (4.03 - 3.95)	4.74 (4.72 - 4.77)

Appendix Table A3: Summary Statistics by Income Quartile (Page 5 of 10)

Diagnosis	Group	Quartile	Count	PY	% Cohort	% Female	Mean Age	10-year Survival, % (95% CI)	Median Survival, y (95% CI)	10-year RMST, y (95% CI)
Hypertension	Cardiovascular diseases	All	2,023,132	15,712,722	100.0	50.3	68.9	60.5 (60.37 - 60.53)	13.3 (13.35 - 13.3)	7.80 (7.79 - 7.8)
Hypertension	Cardiovascular diseases	Q1 (Lowest)	540,820	3,990,202	26.7	51.3	68.3	56.1 (55.92 - 56.23)	11.8 (11.89 - 11.78)	7.51 (7.5 - 7.52)
Hypertension	Cardiovascular diseases	Q2	519,683	4,001,230	25.7	50.6	68.9	59.7 (59.54 - 59.85)	13.1 (13.12 - 13.01)	7.74 (7.74 - 7.75)
Hypertension	Cardiovascular diseases	Q3	499,945	3,954,351	24.7	50.0	69.2	62.1 (61.95 - 62.26)	13.9 (14.0 - 13.88)	7.90 (7.89 - 7.91)
Hypertension	Cardiovascular diseases	Q4 (Highest)	462,684	3,766,940	22.9	49.1	69.5	64.6 (64.45 - 64.77)	14.8 (14.87 - 14.73)	8.08 (8.07 - 8.09)
Pneumoconiosis	Chronic respiratory	All	7,985	58,151	100.0	8.5	73.6	40.1 (38.91 - 41.2)	7.1 (7.39 - 6.82)	6.11 (6.03 - 6.2)
Pneumoconiosis	Chronic respiratory	Q1 (Lowest)	1,953	13,501	24.5	10.1	72.7	37.3 (35.03 - 39.61)	6.6 (7.14 - 6.0)	5.96 (5.78 - 6.13)
Pneumoconiosis	Chronic respiratory	Q2	2,358	16,377	29.5	6.1	74.1	38.4 (36.27 - 40.46)	6.7 (7.12 - 6.17)	5.96 (5.8 - 6.12)
Pneumoconiosis	Chronic respiratory	Q3	2,274	17,071	28.5	8.0	74.0	41.6 (39.43 - 43.71)	7.5 (8.07 - 6.92)	6.21 (6.04 - 6.37)
Pneumoconiosis	Chronic respiratory	Q4 (Highest)	1,400	11,202	17.5	11.0	73.3	44.1 (41.34 - 46.79)	8.1 (9.06 - 7.43)	6.43 (6.23 - 6.64)
Asthma	Chronic respiratory	All	400,545	3,939,215	100.0	61.3	53.2	78.7 (78.51 - 78.79)	NR	8.78 (8.77 - 8.79)
Asthma	Chronic respiratory	Q1 (Lowest)	109,407	1,037,757	27.3	62.4	53.7	74.8 (74.52 - 75.1)	NR	8.55 (8.53 - 8.56)
Asthma	Chronic respiratory	Q2	103,032	1,008,146	25.7	61.5	53.3	77.8 (77.46 - 78.03)	NR	8.71 (8.7 - 8.73)
Asthma	Chronic respiratory	Q3	97,230	968,063	24.3	60.9	53.1	80.0 (79.71 - 80.27)	NR	8.86 (8.84 - 8.88)
Asthma	Chronic respiratory	Q4 (Highest)	90,876	925,249	22.7	60.3	52.5	82.8 (82.58 - 83.12)	NR	9.04 (9.02 - 9.06)
Interstitial lung disease	Chronic respiratory	All	66,040	429,691	100.0	41.8	66.0	48.6 (48.12 - 49.0)	9.3 (9.5 - 9.06)	6.45 (6.41 - 6.48)
Interstitial lung disease	Chronic respiratory	Q1 (Lowest)	17,286	106,670	26.1	43.6	65.8	44.2 (43.32 - 45.02)	7.5 (7.78 - 7.18)	6.11 (6.04 - 6.17)
Interstitial lung disease	Chronic respiratory	Q2	17,212	109,756	26.1	40.8	66.0	47.7 (46.82 - 48.54)	8.8 (9.22 - 8.39)	6.36 (6.3 - 6.43)
Interstitial lung disease	Chronic respiratory	Q3	16,646	110,158	25.2	40.8	66.0	49.9 (49.0 - 50.75)	9.9 (10.46 - 9.55)	6.53 (6.47 - 6.6)
Interstitial lung disease	Chronic respiratory	Q4 (Highest)	14,896	103,108	22.6	41.8	66.1	53.3 (52.36 - 54.21)	11.7 (12.44 - 11.12)	6.84 (6.77 - 6.91)
Cirrhosis	Digestive diseases	All	158,425	1,372,857	100.0	42.6	53.4	65.7 (65.48 - 66.0)	20.1 (20.33 - 19.81)	7.73 (7.71 - 7.74)
Cirrhosis	Digestive diseases	Q1 (Lowest)	68,480	611,345	43.2	38.9	50.0	67.0 (66.66 - 67.44)	20.8 (21.3 - 20.43)	7.85 (7.82 - 7.87)
Cirrhosis	Digestive diseases	Q2	36,640	313,158	23.1	44.5	54.2	64.5 (63.95 - 65.04)	19.1 (19.6 - 18.56)	7.61 (7.57 - 7.64)
Cirrhosis	Digestive diseases	Q3	28,599	240,341	18.1	45.8	56.4	64.3 (63.72 - 64.96)	19.5 (20.15 - 18.92)	7.61 (7.57 - 7.65)
Cirrhosis	Digestive diseases	Q4 (Highest)	24,706	208,012	15.6	46.6	58.1	65.5 (64.83 - 66.16)	20.0 (20.64 - 19.39)	7.70 (7.65 - 7.75)
Inflammatory bowel	Digestive diseases	All	235,177	2,365,227	100.0	56.2	52.8	76.0 (75.82 - 76.2)	NR	8.54 (8.53 - 8.56)
Inflammatory bowel	Digestive diseases	Q1 (Lowest)	59,929	577,701	25.5	56.2	53.1	72.6 (72.21 - 73.0)	NR	8.33 (8.3 - 8.35)
Inflammatory bowel	Digestive diseases	Q2	60,788	607,354	25.8	56.4	52.6	76.0 (75.58 - 76.33)	NR	8.54 (8.51 - 8.56)
Inflammatory bowel	Digestive diseases	Q3	58,830	599,519	25.0	56.5	52.6	76.9 (76.54 - 77.29)	NR	8.60 (8.58 - 8.63)
Inflammatory bowel	Digestive diseases	Q4 (Highest)	55,630	580,652	23.7	55.7	52.9	78.8 (78.39 - 79.14)	NR	8.72 (8.7 - 8.75)
Vascular intestinal	Digestive diseases	All	31,430	138,242	100.0	54.0	72.8	34.2 (33.51 - 34.82)	5.2 (5.34 - 5.06)	5.25 (5.2 - 5.3)
Vascular intestinal	Digestive diseases	Q1 (Lowest)	9,489	38,787	30.2	52.9	71.1	31.3 (30.13 - 32.49)	4.5 (4.78 - 4.28)	4.99 (4.9 - 5.08)
Vascular intestinal	Digestive diseases	Q2	8,272	36,259	26.3	56.0	73.1	33.6 (32.39 - 34.92)	5.0 (5.27 - 4.73)	5.19 (5.09 - 5.28)
Vascular intestinal	Digestive diseases	Q3	7,351	32,783	23.4	55.3	73.6	35.1 (33.78 - 36.5)	5.5 (5.77 - 5.19)	5.33 (5.23 - 5.44)
Vascular intestinal	Digestive diseases	Q4 (Highest)	6,318	30,413	20.1	51.6	73.8	37.9 (36.42 - 39.38)	6.2 (6.59 - 5.77)	5.63 (5.52 - 5.74)
Gallbladder & biliary	Digestive diseases	All	499,559	4,549,039	100.0	62.5	56.9	76.0 (75.88 - 76.15)	NR	8.52 (8.51 - 8.53)
Gallbladder & biliary	Digestive diseases	Q1 (Lowest)	143,523	1,266,743	28.7	63.8	56.1	73.8 (73.56 - 74.08)	NR	8.40 (8.38 - 8.42)
Gallbladder & biliary	Digestive diseases	Q2	131,210	1,191,367	26.3	62.9	56.7	76.0 (75.7 - 76.23)	NR	8.51 (8.5 - 8.53)
Gallbladder & biliary	Digestive diseases	Q3	121,253	1,123,181	24.3	62.2	57.2	77.2 (76.92 - 77.45)	NR	8.59 (8.57 - 8.6)
Gallbladder & biliary	Digestive diseases	Q4 (Highest)	103,573	967,748	20.7	60.3	58.1	77.7 (77.43 - 78.0)	NR	8.62 (8.6 - 8.64)
Pancreatitis	Digestive diseases	All	110,236	794,498	100.0	48.6	62.0	62.5 (62.13 - 62.81)	15.5 (15.77 - 15.35)	7.65 (7.63 - 7.67)
Pancreatitis	Digestive diseases	Q1 (Lowest)	33,823	239,007	30.6	48.2	60.7	59.5 (58.87 - 60.12)	13.8 (14.17 - 13.55)	7.48 (7.44 - 7.52)
Pancreatitis	Digestive diseases	Q2	28,552	204,853	25.9	48.6	62.0	62.3 (61.63 - 62.97)	15.5 (15.93 - 15.02)	7.63 (7.58 - 7.67)
Pancreatitis	Digestive diseases	Q3	25,526	186,722	23.2	49.1	62.5	63.8 (63.07 - 64.47)	16.5 (16.94 - 16.12)	7.73 (7.69 - 7.78)
Pancreatitis	Digestive diseases	Q4 (Highest)	22,335	163,916	20.3	48.8	63.2	65.8 (65.02 - 66.51)	17.7 (18.13 - 17.09)	7.84 (7.79 - 7.89)
Diverticular disease	Digestive diseases	All	376,321	2,931,086	100.0	56.5	67.2	68.2 (67.98 - 68.34)	17.0 (17.09 - 16.88)	8.28 (8.27 - 8.29)
Diverticular disease	Digestive diseases	Q1 (Lowest)	89,710	677,436	23.8	57.6	67.0	63.9 (63.48 - 64.24)	15.1 (15.21 - 14.85)	8.02 (7.99 - 8.04)
Diverticular disease	Digestive diseases	Q2	97,451	752,665	26.0	56.7	66.8	67.9 (67.58 - 68.28)	17.1 (17.27 - 16.83)	8.26 (8.24 - 8.28)
Diverticular disease	Digestive diseases	Q3	96,485	757,142	25.6	56.2	67.3	69.3 (68.93 - 69.63)	17.3 (17.56 - 17.08)	8.34 (8.32 - 8.36)
Diverticular disease	Digestive diseases	Q4 (Highest)	92,675	743,843	24.6	55.4	67.9	71.4 (71.06 - 71.76)	18.4 (18.67 - 18.15)	8.48 (8.46 - 8.5)

Appendix Table A3: Summary Statistics by Income Quartile (Page 6 of 10)

Diagnosis	Group	Quartile	Count	PY	% Cohort	% Female	Mean Age	10-year Survival, % (95% CI)	Median Survival, y (95% CI)	10-year RMST, y (95% CI)
Upper digest diseases	Digestive diseases	All	914,898	8,585,912	100.0	54.4	57.0	73.3 (73.2 - 73.4)	NR	8.41 (8.4 - 8.42)
Upper digest diseases	Digestive diseases	Q1 (Lowest)	283,066	2,601,694	31.0	53.9	55.0	72.2 (72.04 - 72.41)	NR	8.34 (8.33 - 8.35)
Upper digest diseases	Digestive diseases	Q2	234,061	2,195,400	25.6	54.9	57.1	72.9 (72.71 - 73.11)	NR	8.38 (8.37 - 8.39)
Upper digest diseases	Digestive diseases	Q3	211,624	2,011,441	23.1	54.6	58.1	73.7 (73.52 - 73.94)	NR	8.43 (8.42 - 8.45)
Upper digest diseases	Digestive diseases	Q4 (Highest)	186,147	1,777,377	20.3	54.2	58.8	74.9 (74.68 - 75.12)	NR	8.52 (8.5 - 8.53)
Parkinson's disease	Neurological disorders	All	65,735	382,753	100.0	40.4	73.6	33.2 (32.74 - 33.6)	6.8 (6.87 - 6.73)	6.28 (6.26 - 6.31)
Parkinson's disease	Neurological disorders	Q1 (Lowest)	15,589	87,203	23.7	41.4	73.3	30.0 (29.18 - 30.89)	6.2 (6.36 - 6.07)	6.00 (5.94 - 6.06)
Parkinson's disease	Neurological disorders	Q2	15,866	92,176	24.1	40.5	73.3	33.0 (32.13 - 33.88)	6.8 (6.94 - 6.63)	6.25 (6.19 - 6.31)
Parkinson's disease	Neurological disorders	Q3	16,412	97,553	25.0	40.3	73.7	34.8 (33.96 - 35.71)	7.0 (7.12 - 6.84)	6.39 (6.33 - 6.45)
Parkinson's disease	Neurological disorders	Q4 (Highest)	17,868	105,822	27.2	39.6	74.2	34.5 (33.71 - 35.4)	7.2 (7.34 - 7.06)	6.47 (6.41 - 6.52)
Idiopathic epilepsy	Neurological disorders	All	148,081	1,245,506	100.0	46.7	57.4	57.0 (56.78 - 57.33)	14.1 (14.29 - 13.9)	7.12 (7.1 - 7.14)
Idiopathic epilepsy	Neurological disorders	Q1 (Lowest)	49,106	401,651	33.2	44.9	56.0	55.3 (54.82 - 55.78)	12.7 (12.98 - 12.4)	7.06 (7.03 - 7.1)
Idiopathic epilepsy	Neurological disorders	Q2	36,890	320,936	24.9	47.2	56.6	58.9 (58.35 - 59.45)	15.5 (15.84 - 15.05)	7.24 (7.2 - 7.28)
Idiopathic epilepsy	Neurological disorders	Q3	32,112	272,902	21.7	47.8	58.3	57.8 (57.16 - 58.34)	14.9 (15.46 - 14.42)	7.11 (7.07 - 7.15)
Idiopathic epilepsy	Neurological disorders	Q4 (Highest)	29,973	250,017	20.2	47.9	59.4	56.9 (56.26 - 57.49)	14.3 (14.8 - 13.84)	7.08 (7.03 - 7.12)
Multiple sclerosis	Neurological disorders	All	27,843	338,448	100.0	68.4	45.2	89.3 (88.89 - 89.69)	NR	9.47 (9.45 - 9.5)
Multiple sclerosis	Neurological disorders	Q1 (Lowest)	6,794	79,757	24.4	68.3	45.6	86.1 (85.17 - 86.99)	NR	9.32 (9.27 - 9.37)
Multiple sclerosis	Neurological disorders	Q2	7,169	86,311	25.8	67.7	45.2	89.0 (88.13 - 89.74)	NR	9.44 (9.4 - 9.49)
Multiple sclerosis	Neurological disorders	Q3	6,887	84,734	24.7	68.3	44.9	90.6 (89.81 - 91.33)	NR	9.53 (9.49 - 9.58)
Multiple sclerosis	Neurological disorders	Q4 (Highest)	6,993	87,646	25.1	69.2	45.2	91.5 (90.7 - 92.15)	NR	9.59 (9.55 - 9.63)
Motor neuron disease	Neurological disorders	All	10,196	36,388	100.0	41.8	67.0	18.4 (17.54 - 19.2)	1.6 (1.68 - 1.57)	3.40 (3.32 - 3.47)
Motor neuron disease	Neurological disorders	Q1 (Lowest)	2,400	8,118	23.5	42.8	65.8	17.1 (15.52 - 18.85)	1.4 (1.52 - 1.3)	3.21 (3.06 - 3.36)
Motor neuron disease	Neurological disorders	Q2	2,556	9,158	25.1	41.4	66.5	18.7 (17.04 - 20.37)	1.6 (1.71 - 1.5)	3.40 (3.25 - 3.55)
Motor neuron disease	Neurological disorders	Q3	2,588	9,310	25.4	41.3	67.6	18.7 (17.09 - 20.4)	1.7 (1.84 - 1.63)	3.47 (3.32 - 3.62)
Motor neuron disease	Neurological disorders	Q4 (Highest)	2,652	9,802	26.0	41.7	68.1	18.8 (17.16 - 20.46)	1.7 (1.81 - 1.61)	3.49 (3.35 - 3.64)
Anxiety disorders	Mental disorders & substance use	All	912,440	8,614,282	100.0	61.3	41.1	87.6 (87.53 - 87.68)	NR	9.28 (9.27 - 9.28)
Anxiety disorders	Mental disorders & substance use	Q1 (Lowest)	310,667	3,075,292	34.0	58.9	41.3	87.3 (87.15 - 87.42)	NR	9.27 (9.26 - 9.28)
Anxiety disorders	Mental disorders & substance use	Q2	242,632	2,289,538	26.6	61.6	40.4	88.0 (87.87 - 88.17)	NR	9.30 (9.29 - 9.31)
Anxiety disorders	Mental disorders & substance use	Q3	193,099	1,772,685	21.2	62.3	41.1	87.7 (87.5 - 87.84)	NR	9.27 (9.26 - 9.28)
Anxiety disorders	Mental disorders & substance use	Q4 (Highest)	166,042	1,476,766	18.2	64.3	41.6	87.6 (87.41 - 87.78)	NR	9.27 (9.26 - 9.28)
ADHD	Mental disorders & substance use	All	198,591	1,424,289	100.0	51.5	30.1	96.0 (95.88 - 96.12)	NR	9.81 (9.81 - 9.82)
ADHD	Mental disorders & substance use	Q1 (Lowest)	73,785	576,542	37.2	50.2	32.2	94.3 (94.11 - 94.54)	NR	9.73 (9.72 - 9.74)
ADHD	Mental disorders & substance use	Q2	56,297	401,957	28.3	52.4	29.4	96.8 (96.55 - 96.95)	NR	9.85 (9.84 - 9.86)
ADHD	Mental disorders & substance use	Q3	39,293	258,924	19.8	51.7	28.7	97.3 (97.1 - 97.56)	NR	9.87 (9.86 - 9.88)
ADHD	Mental disorders & substance use	Q4 (Highest)	29,216	186,866	14.7	52.5	28.0	97.8 (97.51 - 98.01)	NR	9.90 (9.89 - 9.91)
Rheumatoid arthritis	Musculoskeletal disorders	All	122,790	1,253,520	100.0	68.8	61.0	72.8 (72.54 - 73.09)	19.8 (19.95 - 19.6)	8.58 (8.57 - 8.6)
Rheumatoid arthritis	Musculoskeletal disorders	Q1 (Lowest)	31,738	311,414	25.8	69.7	61.2	68.6 (68.05 - 69.19)	17.4 (17.83 - 17.06)	8.33 (8.3 - 8.37)
Rheumatoid arthritis	Musculoskeletal disorders	Q2	32,422	330,928	26.5	67.9	60.8	72.5 (71.94 - 73.02)	19.8 (20.11 - 19.33)	8.57 (8.54 - 8.6)
Rheumatoid arthritis	Musculoskeletal disorders	Q3	30,635	315,925	24.9	68.6	61.0	74.6 (74.09 - 75.18)	20.7 (21.18 - 20.16)	8.69 (8.65 - 8.72)
Rheumatoid arthritis	Musculoskeletal disorders	Q4 (Highest)	27,995	295,253	22.8	69.1	61.1	76.0 (75.44 - 76.55)	22.0 (22.6 - 21.42)	8.77 (8.74 - 8.8)
Osteoarthritis	Musculoskeletal disorders	All	844,730	8,179,587	100.0	56.7	64.8	79.1 (79.0 - 79.2)	20.4 (20.45 - 20.29)	9.06 (9.05 - 9.06)
Osteoarthritis	Musculoskeletal disorders	Q1 (Lowest)	188,012	1,791,113	22.3	59.7	64.6	75.0 (74.74 - 75.19)	18.5 (18.67 - 18.41)	8.83 (8.82 - 8.84)
Osteoarthritis	Musculoskeletal disorders	Q2	211,576	2,059,124	25.0	56.2	64.3	79.1 (78.87 - 79.27)	20.4 (20.55 - 20.21)	9.04 (9.03 - 9.05)
Osteoarthritis	Musculoskeletal disorders	Q3	222,056	2,164,183	26.3	55.6	64.7	80.3 (80.08 - 80.47)	21.1 (21.17 - 20.94)	9.12 (9.11 - 9.13)
Osteoarthritis	Musculoskeletal disorders	Q4 (Highest)	223,086	2,165,166	26.4	55.8	65.6	81.5 (81.33 - 81.71)	21.3 (21.46 - 21.12)	9.20 (9.19 - 9.2)
Low back pain	Musculoskeletal disorders	All	567,352	5,703,096	100.0	53.6	53.2	83.4 (83.26 - 83.48)	NR	9.13 (9.13 - 9.14)
Low back pain	Musculoskeletal disorders	Q1 (Lowest)	165,380	1,669,846	29.2	53.6	51.4	83.5 (83.29 - 83.69)	NR	9.13 (9.12 - 9.14)
Low back pain	Musculoskeletal disorders	Q2	141,918	1,435,596	25.0	53.9	52.8	83.1 (82.9 - 83.34)	NR	9.11 (9.1 - 9.12)
Low back pain	Musculoskeletal disorders	Q3	131,122	1,325,903	23.1	53.5	54.1	83.1 (82.9 - 83.36)	NR	9.12 (9.1 - 9.13)
Low back pain	Musculoskeletal disorders	Q4 (Highest)	128,932	1,271,751	22.7	53.4	55.1	83.7 (83.49 - 83.95)	NR	9.17 (9.16 - 9.19)

Appendix Table A3: Summary Statistics by Income Quartile (Page 7 of 10)

Diagnosis	Group	Quartile	Count	PY	% Cohort	% Female	Mean Age	10-year Survival, % (95% CI)	Median Survival, y (95% CI)	10-year RMST, y (95% CI)
Neck pain	Musculoskeletal disorders	All	249,816	2,467,284	100.0	56.2	50.2	90.1 (89.93 - 90.2)	NR	9.50 (9.49 - 9.51)
Neck pain	Musculoskeletal disorders	Q1 (Lowest)	70,505	698,054	28.3	57.0	49.8	89.0 (88.76 - 89.3)	NR	9.44 (9.43 - 9.46)
Neck pain	Musculoskeletal disorders	Q2	64,531	643,181	25.8	56.2	49.7	90.2 (89.94 - 90.47)	NR	9.51 (9.5 - 9.53)
Neck pain	Musculoskeletal disorders	Q3	58,790	586,154	23.5	55.5	50.4	90.4 (90.12 - 90.67)	NR	9.52 (9.51 - 9.54)
Neck pain	Musculoskeletal disorders	Q4 (Highest)	55,990	539,894	22.4	56.1	51.2	90.9 (90.61 - 91.17)	NR	9.54 (9.53 - 9.56)
Gout	Musculoskeletal disorders	All	134,140	782,222	100.0	30.6	71.9	42.4 (42.1 - 42.74)	7.8 (7.85 - 7.69)	6.43 (6.4 - 6.45)
Gout	Musculoskeletal disorders	Q1 (Lowest)	36,494	205,155	27.1	32.6	70.8	39.5 (38.89 - 40.1)	7.1 (7.2 - 6.92)	6.23 (6.18 - 6.27)
Gout	Musculoskeletal disorders	Q2	34,556	196,440	25.8	31.9	72.2	40.9 (40.26 - 41.51)	7.3 (7.5 - 7.21)	6.30 (6.26 - 6.34)
Gout	Musculoskeletal disorders	Q3	32,827	191,316	24.5	30.3	72.6	42.8 (42.12 - 43.41)	7.8 (8.01 - 7.67)	6.45 (6.4 - 6.49)
Gout	Musculoskeletal disorders	Q4 (Highest)	30,263	189,312	22.6	27.0	72.3	47.3 (46.63 - 47.98)	9.2 (9.34 - 8.95)	6.78 (6.74 - 6.83)
Hemoglobinopathies	Other	All	638,150	3,100,659	100.0	55.9	69.7	34.9 (34.75 - 35.02)	5.1 (5.13 - 5.06)	5.38 (5.37 - 5.39)
Hemoglobinopathies	Other	Q1 (Lowest)	197,518	964,632	31.0	55.9	67.0	36.3 (36.02 - 36.53)	5.4 (5.49 - 5.37)	5.50 (5.48 - 5.52)
Hemoglobinopathies	Other	Q2	159,835	762,866	25.0	56.1	70.1	33.9 (33.6 - 34.16)	4.8 (4.9 - 4.77)	5.29 (5.27 - 5.31)
Hemoglobinopathies	Other	Q3	145,421	698,701	22.8	55.8	71.3	33.8 (33.49 - 34.06)	4.8 (4.89 - 4.76)	5.28 (5.26 - 5.3)
Hemoglobinopathies	Other	Q4 (Highest)	135,376	674,460	21.2	55.6	71.5	35.3 (34.96 - 35.56)	5.2 (5.27 - 5.14)	5.42 (5.39 - 5.44)
Endo/metab/blood/immune	Other	All	1,693,901	14,362,618	100.0	60.0	58.6	72.1 (72.01 - 72.17)	20.6 (20.71 - 20.56)	8.38 (8.37 - 8.38)
Endo/metab/blood/immune	Other	Q1 (Lowest)	466,093	3,818,249	27.5	61.0	57.4	69.6 (69.43 - 69.74)	19.1 (19.24 - 19.0)	8.22 (8.21 - 8.23)
Endo/metab/blood/immune	Other	Q2	424,588	3,585,435	25.1	59.7	58.8	71.4 (71.23 - 71.54)	20.2 (20.31 - 20.01)	8.33 (8.32 - 8.34)
Endo/metab/blood/immune	Other	Q3	409,035	3,510,744	24.1	59.7	59.1	73.0 (72.8 - 73.11)	21.1 (21.29 - 20.94)	8.43 (8.42 - 8.44)
Endo/metab/blood/immune	Other	Q4 (Highest)	394,185	3,448,190	23.3	59.5	59.1	74.9 (74.76 - 75.07)	22.4 (22.62 - 22.16)	8.56 (8.55 - 8.57)
Psoriasis	Other	All	233,026	2,552,075	100.0	52.5	53.0	85.0 (84.82 - 85.14)	NR	9.26 (9.25 - 9.27)
Psoriasis	Other	Q1 (Lowest)	56,678	606,410	24.3	53.3	52.5	81.6 (81.26 - 81.97)	NR	9.07 (9.05 - 9.09)
Psoriasis	Other	Q2	60,979	675,473	26.2	53.0	52.5	85.0 (84.69 - 85.32)	NR	9.26 (9.24 - 9.27)
Psoriasis	Other	Q3	59,328	658,654	25.5	52.2	53.0	86.3 (85.95 - 86.57)	NR	9.33 (9.32 - 9.35)
Psoriasis	Other	Q4 (Highest)	56,041	611,537	24.0	51.6	54.0	87.0 (86.69 - 87.32)	NR	9.38 (9.36 - 9.39)
Decubitus ulcer	Other	All	76,832	233,339	100.0	52.4	76.0	16.7 (16.34 - 17.0)	2.0 (2.03 - 1.94)	3.55 (3.53 - 3.58)
Decubitus ulcer	Other	Q1 (Lowest)	25,793	81,180	33.6	48.9	72.7	17.6 (16.98 - 18.16)	2.3 (2.38 - 2.22)	3.77 (3.72 - 3.82)
Decubitus ulcer	Other	Q2	18,855	57,801	24.5	53.6	76.3	16.7 (16.06 - 17.37)	2.0 (2.06 - 1.89)	3.53 (3.48 - 3.59)
Decubitus ulcer	Other	Q3	16,820	48,723	21.9	54.9	78.2	15.6 (14.95 - 16.3)	1.7 (1.77 - 1.62)	3.35 (3.29 - 3.41)
Decubitus ulcer	Other	Q4 (Highest)	15,364	45,635	20.0	54.0	78.9	16.2 (15.5 - 16.94)	1.9 (1.92 - 1.76)	3.45 (3.38 - 3.51)
Alcohol use disorders	Mental disorders & substance use	All	369,308	3,434,020	100.0	35.7	44.1	79.5 (79.39 - 79.69)	NR	8.88 (8.87 - 8.89)
Alcohol use disorders	Mental disorders & substance use	Q1 (Lowest)	126,889	1,201,741	34.4	34.8	45.1	76.1 (75.8 - 76.33)	23.0	8.67 (8.65 - 8.68)
Alcohol use disorders	Mental disorders & substance use	Q2	95,280	894,196	25.8	36.8	43.4	80.4 (80.07 - 80.65)	NR	8.92 (8.9 - 8.94)
Alcohol use disorders	Mental disorders & substance use	Q3	78,419	721,514	21.2	35.1	43.7	81.7 (81.41 - 82.04)	NR	9.01 (9.0 - 9.03)
Alcohol use disorders	Mental disorders & substance use	Q4 (Highest)	68,720	616,569	18.6	36.7	43.7	82.7 (82.38 - 83.05)	NR	9.07 (9.05 - 9.09)
Drug use disorders	Mental disorders & substance use	All	183,777	1,674,435	100.0	39.5	36.5	83.6 (83.38 - 83.78)	NR	9.08 (9.07 - 9.1)
Drug use disorders	Mental disorders & substance use	Q1 (Lowest)	80,615	748,845	43.8	38.5	37.4	82.7 (82.39 - 83.0)	NR	9.04 (9.03 - 9.06)
Drug use disorders	Mental disorders & substance use	Q2	46,975	433,352	25.6	41.0	35.4	84.7 (84.33 - 85.09)	NR	9.15 (9.13 - 9.18)
Drug use disorders	Mental disorders & substance use	Q3	31,920	283,077	17.4	39.5	35.9	84.3 (83.81 - 84.76)	NR	9.11 (9.09 - 9.14)
Drug use disorders	Mental disorders & substance use	Q4 (Highest)	24,267	209,161	13.2	39.8	36.4	83.5 (82.96 - 84.07)	NR	9.05 (9.02 - 9.08)
Acute glomerulonephritis	Diabetes & CKD	All	4,245	43,581	100.0	47.8	53.3	76.3 (74.84 - 77.61)	NR	8.58 (8.49 - 8.67)
Acute glomerulonephritis	Diabetes & CKD	Q1 (Lowest)	1,066	10,307	25.1	47.5	52.8	72.3 (69.3 - 75.14)	NR	8.27 (8.07 - 8.47)
Acute glomerulonephritis	Diabetes & CKD	Q2	1,022	10,530	24.1	45.9	53.4	75.5 (72.55 - 78.26)	NR	8.55 (8.36 - 8.74)
Acute glomerulonephritis	Diabetes & CKD	Q3	1,080	11,134	25.4	48.7	53.8	77.7 (74.82 - 80.26)	NR	8.72 (8.55 - 8.89)
Acute glomerulonephritis	Diabetes & CKD	Q4 (Highest)	1,077	11,610	25.4	48.9	53.2	79.3 (76.58 - 81.79)	NR	8.77 (8.6 - 8.94)
Schizophrenia	Mental disorders & substance use	All	41,120	466,518	100.0	45.3	46.7	77.2 (76.71 - 77.58)	NR	8.75 (8.72 - 8.77)
Schizophrenia	Mental disorders & substance use	Q1 (Lowest)	25,512	276,289	62.1	43.5	48.6	74.2 (73.6 - 74.76)	20.4 (20.77 - 19.96)	8.59 (8.56 - 8.63)
Schizophrenia	Mental disorders & substance use	Q2	9,025	113,315	21.9	48.4	43.8	82.0 (81.14 - 82.82)	NR	9.02 (8.96 - 9.07)
Schizophrenia	Mental disorders & substance use	Q3	4,034	48,230	9.8	47.7	43.3	82.4 (81.06 - 83.58)	NR	9.00 (8.92 - 9.08)
Schizophrenia	Mental disorders & substance use	Q4 (Highest)	2,549	28,685	6.2	49.6	43.7	80.9 (79.18 - 82.48)	NR	8.89 (8.79 - 9.0)

Appendix Table A3: Summary Statistics by Income Quartile (Page 8 of 10)

Diagnosis	Group	Quartile	Count	PY	% Cohort	% Female	Mean Age	10-year Survival, % (95% CI)	Median Survival, y (95% CI)	10-year RMST, y (95% CI)
Bipolar disorder	Mental disorders & substance use	All	105,060	1,042,827	100.0	62.1	41.4	87.3 (87.07 - 87.53)	NR	9.35 (9.33 - 9.36)
Bipolar disorder	Mental disorders & substance use	Q1 (Lowest)	38,126	379,795	36.3	62.5	42.4	85.7 (85.27 - 86.06)	NR	9.27 (9.24 - 9.29)
Bipolar disorder	Mental disorders & substance use	Q2	29,280	293,272	27.9	63.5	39.8	89.1 (88.68 - 89.49)	NR	9.44 (9.42 - 9.46)
Bipolar disorder	Mental disorders & substance use	Q3	21,040	208,074	20.0	61.1	40.9	88.0 (87.51 - 88.52)	NR	9.39 (9.36 - 9.41)
Bipolar disorder	Mental disorders & substance use	Q4 (Highest)	16,614	161,686	15.8	60.1	42.6	87.0 (86.46 - 87.63)	NR	9.31 (9.27 - 9.34)
Dementia as a sign with others	Neurological disorders	All	119,384	470,725	100.0	59.0	79.4	11.2 (11.0 - 11.45)	4.1 (4.17 - 4.11)	4.61 (4.59 - 4.62)
Dementia as a sign with others	Neurological disorders	Q1 (Lowest)	25,953	98,919	21.7	60.0	77.9	11.8 (11.3 - 12.31)	4.0 (4.02 - 3.89)	4.50 (4.45 - 4.54)
Dementia as a sign with others	Neurological disorders	Q2	27,890	107,939	23.4	58.6	78.9	11.0 (10.52 - 11.46)	4.0 (4.11 - 3.99)	4.55 (4.51 - 4.59)
Dementia as a sign with others	Neurological disorders	Q3	30,749	121,219	25.8	58.4	79.7	10.8 (10.41 - 11.3)	4.1 (4.19 - 4.08)	4.60 (4.56 - 4.64)
Dementia as a sign with others	Neurological disorders	Q4 (Highest)	34,792	142,647	29.1	59.3	80.5	11.4 (10.94 - 11.78)	4.3 (4.39 - 4.3)	4.73 (4.7 - 4.77)
Vascular dementia	Neurological disorders	All	78,849	236,992	100.0	51.6	81.6	6.5 (6.28 - 6.7)	2.5 (2.51 - 2.45)	3.32 (3.29 - 3.34)
Vascular dementia	Neurological disorders	Q1 (Lowest)	21,524	65,680	27.3	50.4	79.7	8.0 (7.59 - 8.48)	2.5 (2.56 - 2.43)	3.41 (3.37 - 3.45)
Vascular dementia	Neurological disorders	Q2	19,876	58,915	25.2	51.0	81.5	6.5 (6.11 - 6.94)	2.5 (2.52 - 2.4)	3.28 (3.24 - 3.33)
Vascular dementia	Neurological disorders	Q3	19,168	56,924	24.3	51.9	82.5	5.5 (5.15 - 5.93)	2.5 (2.52 - 2.41)	3.27 (3.22 - 3.31)
Vascular dementia	Neurological disorders	Q4 (Highest)	18,281	55,473	23.2	53.4	83.2	5.8 (5.41 - 6.2)	2.5 (2.56 - 2.44)	3.30 (3.26 - 3.35)
Unspecified dementia	Neurological disorders	All	226,475	569,664	100.0	59.9	83.6	4.2 (4.06 - 4.26)	1.8 (1.86 - 1.83)	2.74 (2.72 - 2.75)
Unspecified dementia	Neurological disorders	Q1 (Lowest)	65,367	169,504	28.9	58.1	81.9	5.2 (5.04 - 5.46)	1.9 (1.94 - 1.88)	2.84 (2.82 - 2.87)
Unspecified dementia	Neurological disorders	Q2	58,033	143,644	25.6	59.9	83.6	3.9 (3.74 - 4.11)	1.8 (1.81 - 1.75)	2.69 (2.66 - 2.71)
Unspecified dementia	Neurological disorders	Q3	53,440	131,849	23.6	60.3	84.2	3.6 (3.41 - 3.78)	1.8 (1.84 - 1.78)	2.67 (2.65 - 2.7)
Unspecified dementia	Neurological disorders	Q4 (Highest)	49,635	124,667	21.9	61.8	84.8	3.7 (3.5 - 3.89)	1.9 (1.88 - 1.82)	2.72 (2.69 - 2.74)
Alzheimer's disease	Neurological disorders	All	133,400	516,576	100.0	60.1	79.9	11.2 (10.96 - 11.39)	4.1 (4.1 - 4.04)	4.54 (4.52 - 4.56)
Alzheimer's disease	Neurological disorders	Q1 (Lowest)	29,414	108,668	22.0	60.8	78.8	11.1 (10.65 - 11.58)	3.8 (3.88 - 3.77)	4.37 (4.33 - 4.41)
Alzheimer's disease	Neurological disorders	Q2	31,762	120,101	23.8	59.7	79.5	10.8 (10.33 - 11.2)	3.9 (4.0 - 3.87)	4.45 (4.41 - 4.49)
Alzheimer's disease	Neurological disorders	Q3	34,269	133,221	25.7	59.3	80.2	10.9 (10.49 - 11.33)	4.1 (4.13 - 4.03)	4.55 (4.52 - 4.59)
Alzheimer's disease	Neurological disorders	Q4 (Highest)	37,955	154,585	28.5	60.6	80.9	11.8 (11.41 - 12.22)	4.3 (4.4 - 4.3)	4.73 (4.69 - 4.76)
Major depression	Mental disorders & substance use	All	683,332	6,602,547	100.0	59.8	44.8	80.7 (80.64 - 80.84)	NR	8.87 (8.87 - 8.88)
Major depression	Mental disorders & substance use	Q1 (Lowest)	226,776	2,266,431	33.1	58.3	44.4	82.0 (81.8 - 82.15)	NR	8.95 (8.94 - 8.96)
Major depression	Mental disorders & substance use	Q2	183,480	1,775,801	26.9	59.9	43.9	81.5 (81.32 - 81.71)	NR	8.92 (8.91 - 8.93)
Major depression	Mental disorders & substance use	Q3	148,313	1,403,898	21.7	60.1	45.1	79.8 (79.62 - 80.08)	NR	8.81 (8.79 - 8.82)
Major depression	Mental disorders & substance use	Q4 (Highest)	124,763	1,156,417	18.3	61.9	46.4	78.3 (78.09 - 78.6)	NR	8.73 (8.71 - 8.75)
Dysthymia	Mental disorders & substance use	All	29,346	312,722	100.0	55.4	41.2	89.7 (89.28 - 90.07)	NR	9.49 (9.47 - 9.51)
Dysthymia	Mental disorders & substance use	Q1 (Lowest)	12,038	129,594	41.0	54.8	42.6	90.1 (89.44 - 90.65)	NR	9.51 (9.48 - 9.54)
Dysthymia	Mental disorders & substance use	Q2	7,692	82,441	26.2	55.1	40.0	90.0 (89.21 - 90.74)	NR	9.52 (9.48 - 9.56)
Dysthymia	Mental disorders & substance use	Q3	5,401	57,103	18.4	56.1	40.1	89.0 (88.0 - 89.9)	NR	9.43 (9.37 - 9.48)
Dysthymia	Mental disorders & substance use	Q4 (Highest)	4,215	43,584	14.4	56.6	41.1	88.9 (87.75 - 89.92)	NR	9.43 (9.37 - 9.49)
Diabetes type 1	Diabetes & CKD	All	180,139	1,756,646	100.0	43.2	61.8	56.5 (56.28 - 56.77)	12.4 (12.53 - 12.33)	7.38 (7.36 - 7.4)
Diabetes type 1	Diabetes & CKD	Q1 (Lowest)	56,302	513,041	31.3	43.9	61.9	53.0 (52.58 - 53.45)	11.0 (11.17 - 10.86)	7.19 (7.16 - 7.22)
Diabetes type 1	Diabetes & CKD	Q2	47,097	451,050	26.1	44.2	62.4	55.1 (54.65 - 55.6)	11.8 (12.02 - 11.63)	7.30 (7.27 - 7.33)
Diabetes type 1	Diabetes & CKD	Q3	41,929	421,130	23.3	43.2	61.8	58.1 (57.6 - 58.59)	13.2 (13.45 - 13.02)	7.44 (7.41 - 7.48)
Diabetes type 1	Diabetes & CKD	Q4 (Highest)	34,811	371,426	19.3	40.7	60.9	62.2 (61.63 - 62.7)	15.5 (15.85 - 15.19)	7.72 (7.68 - 7.76)
Diabetes type 2	Diabetes & CKD	All	697,339	5,249,549	100.0	43.3	67.6	56.1 (55.97 - 56.24)	11.8 (11.88 - 11.78)	7.48 (7.47 - 7.49)
Diabetes type 2	Diabetes & CKD	Q1 (Lowest)	230,837	1,685,809	33.1	45.2	65.8	54.8 (54.57 - 55.04)	11.4 (11.48 - 11.34)	7.42 (7.4 - 7.43)
Diabetes type 2	Diabetes & CKD	Q2	183,778	1,374,972	26.4	44.1	68.0	55.1 (54.84 - 55.36)	11.5 (11.59 - 11.42)	7.42 (7.4 - 7.43)
Diabetes type 2	Diabetes & CKD	Q3	157,018	1,202,903	22.5	42.6	68.6	56.9 (56.63 - 57.19)	12.1 (12.21 - 12.01)	7.51 (7.5 - 7.53)
Diabetes type 2	Diabetes & CKD	Q4 (Highest)	125,706	985,865	18.0	39.9	69.1	58.9 (58.59 - 59.21)	12.8 (12.94 - 12.72)	7.64 (7.62 - 7.66)
Ischemic stroke	Cardiovascular diseases	All	661,949	4,173,350	100.0	49.0	73.7	42.6 (42.44 - 42.72)	7.9 (7.96 - 7.9)	6.40 (6.39 - 6.41)
Ischemic stroke	Cardiovascular diseases	Q1 (Lowest)	182,459	1,087,937	27.5	48.9	72.7	38.5 (38.29 - 38.81)	7.0 (7.04 - 6.93)	6.09 (6.07 - 6.11)
Ischemic stroke	Cardiovascular diseases	Q2	167,393	1,046,092	25.3	49.4	73.6	42.0 (41.72 - 42.27)	7.8 (7.83 - 7.7)	6.34 (6.32 - 6.36)
Ischemic stroke	Cardiovascular diseases	Q3	160,110	1,027,249	24.2	49.1	74.2	43.9 (43.65 - 44.21)	8.3 (8.33 - 8.19)	6.49 (6.47 - 6.51)
Ischemic stroke	Cardiovascular diseases	Q4 (Highest)	151,987	1,012,072	23.0	48.7	74.6	46.7 (46.38 - 46.96)	9.0 (9.12 - 8.96)	6.72 (6.7 - 6.74)

Appendix Table A3: Summary Statistics by Income Quartile (Page 9 of 10)

Diagnosis	Group	Quartile	Count	PY	% Cohort	% Female	Mean Age	10-year Survival, % (95% CI)	Median Survival, y (95% CI)	10-year RMST, y (95% CI)
Intracerebral hem	Cardiovascular diseases	All	95,308	443,131	100.0	44.7	72.2	30.2 (29.85 - 30.52)	3.9 (4.02 - 3.87)	4.75 (4.72 - 4.78)
Intracerebral hem	Cardiovascular diseases	Q1 (Lowest)	28,752	130,067	30.2	43.5	70.2	29.5 (28.87 - 30.08)	3.8 (3.92 - 3.68)	4.69 (4.64 - 4.74)
Intracerebral hem	Cardiovascular diseases	Q2	23,934	111,349	25.1	45.0	72.2	29.8 (29.12 - 30.44)	3.9 (4.09 - 3.79)	4.74 (4.68 - 4.79)
Intracerebral hem	Cardiovascular diseases	Q3	21,956	102,286	23.0	45.2	73.1	30.6 (29.89 - 31.28)	3.9 (4.09 - 3.77)	4.75 (4.69 - 4.8)
Intracerebral hem	Cardiovascular diseases	Q4 (Highest)	20,666	99,429	21.7	45.5	73.9	31.2 (30.5 - 31.93)	4.2 (4.35 - 4.03)	4.85 (4.79 - 4.91)
Subarachnoid hem	Cardiovascular diseases	All	75,556	465,076	100.0	45.4	68.8	45.5 (45.06 - 45.87)	8.2 (8.3 - 8.0)	6.16 (6.13 - 6.19)
Subarachnoid hem	Cardiovascular diseases	Q1 (Lowest)	21,234	124,830	28.1	45.2	67.1	42.8 (42.03 - 43.55)	7.2 (7.45 - 6.96)	5.96 (5.9 - 6.02)
Subarachnoid hem	Cardiovascular diseases	Q2	18,938	116,686	25.1	45.6	68.7	45.4 (44.56 - 46.17)	8.0 (8.34 - 7.73)	6.14 (6.08 - 6.2)
Subarachnoid hem	Cardiovascular diseases	Q3	17,937	112,371	23.7	46.1	69.5	46.5 (45.64 - 47.3)	8.6 (8.89 - 8.32)	6.24 (6.17 - 6.3)
Subarachnoid hem	Cardiovascular diseases	Q4 (Highest)	17,447	111,188	23.1	44.6	70.3	47.8 (46.96 - 48.65)	9.1 (9.44 - 8.74)	6.36 (6.3 - 6.42)
Urinary tract infections and interstitial nephritis	Other	All	1,189,438	8,569,611	100.0	62.4	63.6	53.0 (52.86 - 53.06)	11.7 (11.75 - 11.62)	6.86 (6.85 - 6.87)
Urinary tract infections and interstitial nephritis	Other	Q1 (Lowest)	344,206	2,437,706	29.0	63.2	62.1	52.1 (51.9 - 52.28)	11.1 (11.25 - 11.03)	6.81 (6.79 - 6.82)
Urinary tract infections and interstitial nephritis	Other	Q2	295,960	2,092,455	24.9	62.9	63.9	51.7 (51.54 - 51.94)	10.9 (11.05 - 10.82)	6.76 (6.75 - 6.78)
Urinary tract infections and interstitial nephritis	Other	Q3	277,695	1,998,523	23.3	61.9	64.7	52.5 (52.31 - 52.73)	11.4 (11.55 - 11.28)	6.83 (6.81 - 6.84)
Urinary tract infections and interstitial nephritis	Other	Q4 (Highest)	271,577	2,040,927	22.8	61.5	64.2	55.8 (55.6 - 56.01)	13.7 (13.83 - 13.52)	7.07 (7.05 - 7.08)
Ulcerative colitis	Digestive diseases	All	207,932	2,059,266	100.0	56.7	53.6	74.8 (74.61 - 75.02)	NR	8.46 (8.45 - 8.48)
Ulcerative colitis	Digestive diseases	Q1 (Lowest)	52,723	501,111	25.4	56.7	53.9	71.3 (70.84 - 71.7)	NR	8.23 (8.2 - 8.26)
Ulcerative colitis	Digestive diseases	Q2	53,797	529,046	25.8	56.8	53.4	74.8 (74.44 - 75.26)	NR	8.46 (8.43 - 8.48)
Ulcerative colitis	Digestive diseases	Q3	52,023	521,444	25.0	56.8	53.4	75.7 (75.31 - 76.12)	NR	8.53 (8.5 - 8.55)
Ulcerative colitis	Digestive diseases	Q4 (Highest)	49,389	507,664	23.8	56.3	53.7	77.6 (77.19 - 78.01)	NR	8.65 (8.62 - 8.67)
Crohn's disease	Digestive diseases	All	53,613	584,516	100.0	51.9	46.2	86.0 (85.67 - 86.33)	NR	9.23 (9.21 - 9.25)
Crohn's disease	Digestive diseases	Q1 (Lowest)	13,672	142,303	25.5	52.5	46.7	82.9 (82.22 - 83.64)	NR	9.06 (9.01 - 9.1)
Crohn's disease	Digestive diseases	Q2	13,727	148,854	25.6	52.4	46.1	85.8 (85.09 - 86.41)	NR	9.23 (9.19 - 9.27)
Crohn's disease	Digestive diseases	Q3	13,588	150,163	25.3	52.3	45.8	86.9 (86.24 - 87.51)	NR	9.28 (9.24 - 9.32)
Crohn's disease	Digestive diseases	Q4 (Highest)	12,626	143,197	23.6	50.1	46.0	88.6 (87.96 - 89.2)	NR	9.36 (9.33 - 9.4)
Depressive disorders	Mental disorders & substance use	All	690,423	6,699,939	100.0	59.7	44.7	80.9 (80.77 - 80.97)	NR	8.88 (8.87 - 8.89)
Depressive disorders	Mental disorders & substance use	Q1 (Lowest)	229,431	2,304,957	33.2	58.2	44.3	82.1 (81.96 - 82.31)	NR	8.96 (8.95 - 8.97)
Depressive disorders	Mental disorders & substance use	Q2	185,381	1,801,645	26.9	59.8	43.8	81.6 (81.43 - 81.83)	NR	8.93 (8.92 - 8.94)
Depressive disorders	Mental disorders & substance use	Q3	149,707	1,422,102	21.7	60.1	45.0	80.0 (79.72 - 80.18)	NR	8.82 (8.8 - 8.83)
Depressive disorders	Mental disorders & substance use	Q4 (Highest)	125,904	1,171,235	18.2	61.9	46.4	78.5 (78.21 - 78.72)	NR	8.74 (8.72 - 8.75)
Angina	Cardiovascular diseases	All	446,351	3,889,011	100.0	41.6	71.3	56.2 (56.05 - 56.37)	11.9 (11.91 - 11.81)	7.53 (7.52 - 7.54)
Angina	Cardiovascular diseases	Q1 (Lowest)	125,125	1,054,731	28.1	42.5	69.9	53.9 (53.63 - 54.24)	11.2 (11.25 - 11.07)	7.38 (7.36 - 7.4)
Angina	Cardiovascular diseases	Q2	116,948	1,009,385	26.2	42.1	71.3	55.1 (54.76 - 55.39)	11.5 (11.59 - 11.38)	7.45 (7.43 - 7.47)
Angina	Cardiovascular diseases	Q3	109,453	966,749	24.5	41.4	71.9	57.2 (56.85 - 57.49)	12.2 (12.28 - 12.07)	7.58 (7.56 - 7.6)
Angina	Cardiovascular diseases	Q4 (Highest)	94,825	858,146	21.2	39.9	72.3	59.5 (59.16 - 59.84)	12.8 (12.92 - 12.71)	7.77 (7.75 - 7.79)
Acute myocardial infarction	Cardiovascular diseases	All	451,526	2,825,484	100.0	39.6	73.4	42.3 (42.14 - 42.46)	7.5 (7.56 - 7.46)	6.07 (6.06 - 6.09)
Acute myocardial infarction	Cardiovascular diseases	Q1 (Lowest)	133,511	797,595	29.6	40.1	72.2	39.3 (39.0 - 39.59)	6.8 (6.83 - 6.67)	5.86 (5.83 - 5.88)
Acute myocardial infarction	Cardiovascular diseases	Q2	119,591	741,728	26.5	40.1	73.5	41.6 (41.25 - 41.87)	7.3 (7.41 - 7.25)	6.02 (6.0 - 6.04)
Acute myocardial infarction	Cardiovascular diseases	Q3	108,802	697,241	24.1	39.4	74.0	43.9 (43.57 - 44.23)	7.9 (8.03 - 7.82)	6.18 (6.15 - 6.2)
Acute myocardial infarction	Cardiovascular diseases	Q4 (Highest)	89,622	588,920	19.8	38.3	74.4	45.9 (45.48 - 46.21)	8.6 (8.71 - 8.46)	6.35 (6.33 - 6.38)
Chronic ischemic heart disease	Cardiovascular diseases	All	609,891	4,366,146	100.0	35.6	72.4	48.7 (48.56 - 48.85)	9.6 (9.67 - 9.58)	6.88 (6.87 - 6.89)
Chronic ischemic heart disease	Cardiovascular diseases	Q1 (Lowest)	175,665	1,203,448	28.9	37.0	71.1	45.7 (45.44 - 45.96)	8.8 (8.83 - 8.69)	6.66 (6.64 - 6.67)
Chronic ischemic heart disease	Cardiovascular diseases	Q2	161,115	1,143,233	26.4	36.3	72.5	47.6 (47.37 - 47.92)	9.3 (9.41 - 9.25)	6.80 (6.78 - 6.82)
Chronic ischemic heart disease	Cardiovascular diseases	Q3	147,797	1,079,194	24.2	35.3	73.0	50.0 (49.75 - 50.32)	10.0 (10.11 - 9.93)	6.97 (6.95 - 6.99)
Chronic ischemic heart disease	Cardiovascular diseases	Q4 (Highest)	125,314	940,271	20.5	33.1	73.3	52.7 (52.41 - 53.04)	10.8 (10.88 - 10.7)	7.19 (7.17 - 7.21)
HIV/AIDS	Other	All	43,671	488,722	100.0	47.0	43.1	86.4 (86.04 - 86.73)	NR	9.16 (9.14 - 9.18)
HIV/AIDS	Other	Q1 (Lowest)	12,162	135,500	27.8	46.4	42.4	84.8 (84.09 - 85.47)	NR	9.07 (9.03 - 9.12)
HIV/AIDS	Other	Q2	10,384	114,453	23.8	48.7	42.8	85.8 (85.06 - 86.51)	NR	9.12 (9.07 - 9.17)
HIV/AIDS	Other	Q3	10,202	115,802	23.4	47.2	43.3	86.9 (86.19 - 87.6)	NR	9.18 (9.13 - 9.23)
HIV/AIDS	Other	Q4 (Highest)	10,923	122,966	25.0	45.7	44.0	88.2 (87.58 - 88.89)	NR	9.28 (9.24 - 9.32)

Appendix Table A3: Summary Statistics by Income Quartile (Page 10 of 10)

Diagnosis	Group	Quartile	Count	PY	% Cohort	% Female	Mean Age	10-year Survival, % (95% CI)	Median Survival, y (95% CI)	10-year RMST, y (95% CI)
Self-harm	Mental disorders & substance use	All	136,115	1,373,186	100.0	56.4	38.2	82.4 (82.2 - 82.65)	NR	8.94 (8.93 - 8.96)
Self-harm	Mental disorders & substance use	Q1 (Lowest)	56,405	561,658	41.4	54.5	38.3	82.0 (81.67 - 82.38)	NR	8.94 (8.92 - 8.97)
Self-harm	Mental disorders & substance use	Q2	36,180	368,962	26.6	57.4	37.6	83.5 (83.02 - 83.87)	NR	9.00 (8.97 - 9.02)
Self-harm	Mental disorders & substance use	Q3	25,165	255,504	18.5	56.8	38.1	82.9 (82.34 - 83.39)	NR	8.96 (8.93 - 8.99)
Self-harm	Mental disorders & substance use	Q4 (Highest)	18,365	187,062	13.5	59.3	39.5	81.0 (80.41 - 81.66)	NR	8.82 (8.77 - 8.86)
Aortic aneurysm	Cardiovascular diseases	All	110,830	622,363	100.0	28.0	71.8	42.6 (42.21 - 42.92)	8.1 (8.2 - 8.03)	6.49 (6.47 - 6.52)
Aortic aneurysm	Cardiovascular diseases	Q1 (Lowest)	29,532	155,535	26.6	29.1	71.6	37.2 (36.48 - 37.83)	6.9 (7.03 - 6.76)	6.07 (6.03 - 6.12)
Aortic aneurysm	Cardiovascular diseases	Q2	29,743	165,345	26.9	28.6	71.9	41.5 (40.79 - 42.17)	7.9 (8.06 - 7.74)	6.42 (6.37 - 6.47)
Aortic aneurysm	Cardiovascular diseases	Q3	27,578	158,980	24.9	27.5	72.0	44.0 (43.26 - 44.71)	8.5 (8.67 - 8.29)	6.63 (6.58 - 6.68)
Aortic aneurysm	Cardiovascular diseases	Q4 (Highest)	23,977	142,503	21.6	26.2	71.8	49.1 (48.34 - 49.92)	9.8 (9.98 - 9.54)	6.96 (6.91 - 7.01)
Lupus	Musculoskeletal disorders	All	17,427	198,758	100.0	80.2	53.2	80.0 (79.37 - 80.67)	NR	8.94 (8.9 - 8.98)
Lupus	Musculoskeletal disorders	Q1 (Lowest)	4,739	52,381	27.1	80.3	52.7	78.2 (76.83 - 79.41)	NR	8.85 (8.77 - 8.92)
Lupus	Musculoskeletal disorders	Q2	4,386	49,743	25.2	80.1	53.1	79.9 (78.61 - 81.2)	NR	8.90 (8.82 - 8.98)
Lupus	Musculoskeletal disorders	Q3	4,249	49,205	24.4	79.9	53.5	80.7 (79.31 - 81.91)	NR	8.98 (8.9 - 9.06)
Lupus	Musculoskeletal disorders	Q4 (Highest)	4,053	47,429	23.3	80.7	53.5	81.7 (80.33 - 82.94)	NR	9.05 (8.97 - 9.13)
Opioid use disorders	Mental disorders & substance use	All	45,670	380,322	100.0	39.1	40.2	76.8 (76.35 - 77.26)	NR	8.63 (8.61 - 8.66)
Opioid use disorders	Mental disorders & substance use	Q1 (Lowest)	23,547	196,493	51.6	36.1	39.7	76.9 (76.27 - 77.54)	NR	8.68 (8.64 - 8.72)
Opioid use disorders	Mental disorders & substance use	Q2	10,748	91,583	23.5	40.9	39.2	78.1 (77.21 - 79.04)	NR	8.70 (8.64 - 8.76)
Opioid use disorders	Mental disorders & substance use	Q3	6,626	54,031	14.5	41.9	41.2	76.1 (74.88 - 77.29)	NR	8.54 (8.47 - 8.62)
Opioid use disorders	Mental disorders & substance use	Q4 (Highest)	4,749	38,216	10.4	45.3	43.3	74.2 (72.71 - 75.61)	NR	8.37 (8.28 - 8.47)
Sleep Apnea	Chronic respiratory	All	293,509	2,725,405	100.0	30.1	56.1	87.0 (86.88 - 87.17)	NR	9.37 (9.36 - 9.38)
Sleep Apnea	Chronic respiratory	Q1 (Lowest)	71,852	645,156	24.5	33.9	54.7	84.3 (84.01 - 84.65)	NR	9.21 (9.2 - 9.23)
Sleep Apnea	Chronic respiratory	Q2	76,705	711,050	26.1	30.6	55.4	87.3 (87.05 - 87.61)	NR	9.39 (9.37 - 9.4)
Sleep Apnea	Chronic respiratory	Q3	74,916	704,715	25.5	28.7	56.4	88.1 (87.84 - 88.4)	NR	9.43 (9.42 - 9.45)
Sleep Apnea	Chronic respiratory	Q4 (Highest)	70,036	664,482	23.9	27.2	57.9	88.2 (87.95 - 88.52)	NR	9.45 (9.43 - 9.46)
Anorexia nervosa	Mental disorders & substance use	All	17,392	170,677	100.0	93.8	24.5	96.0 (95.69 - 96.38)	NR	9.78 (9.76 - 9.8)
Anorexia nervosa	Mental disorders & substance use	Q1 (Lowest)	3,857	39,488	22.2	91.7	27.8	93.4 (92.47 - 94.27)	NR	9.61 (9.56 - 9.67)
Anorexia nervosa	Mental disorders & substance use	Q2	4,514	44,279	26.0	93.8	24.5	96.2 (95.52 - 96.85)	NR	9.79 (9.76 - 9.83)
Anorexia nervosa	Mental disorders & substance use	Q3	4,269	41,124	24.5	94.5	23.6	96.5 (95.82 - 97.14)	NR	9.81 (9.77 - 9.84)
Anorexia nervosa	Mental disorders & substance use	Q4 (Highest)	4,752	45,785	27.3	94.8	22.4	97.7 (97.07 - 98.13)	NR	9.88 (9.85 - 9.91)
Tuberculosis	Other	All	40,699	418,623	100.0	54.9	49.3	78.7 (78.28 - 79.13)	NR	8.69 (8.66 - 8.72)
Tuberculosis	Other	Q1 (Lowest)	19,090	198,313	46.9	53.1	44.2	83.8 (83.19 - 84.32)	NR	9.01 (8.98 - 9.05)
Tuberculosis	Other	Q2	8,443	85,491	20.7	56.3	51.8	75.5 (74.49 - 76.46)	NR	8.47 (8.41 - 8.54)
Tuberculosis	Other	Q3	6,821	69,523	16.8	56.2	54.4	73.6 (72.47 - 74.7)	NR	8.34 (8.26 - 8.42)
Tuberculosis	Other	Q4 (Highest)	6,345	65,297	15.6	57.4	55.9	73.6 (72.45 - 74.75)	NR	8.38 (8.31 - 8.46)
Soft tissue and other extraosseous sarcomas	Cancers	All	18,536	111,545	100.0	52.6	64.4	43.5 (42.67 - 44.28)	7.0 (7.25 - 6.65)	5.87 (5.8 - 5.93)
Soft tissue and other extraosseous sarcomas	Cancers	Q1 (Lowest)	4,359	24,512	23.5	53.7	63.6	39.7 (38.05 - 41.32)	5.5 (6.09 - 4.79)	5.52 (5.39 - 5.65)
Soft tissue and other extraosseous sarcomas	Cancers	Q2	4,423	26,033	23.9	52.1	64.6	41.5 (39.86 - 43.11)	5.9 (6.59 - 5.4)	5.70 (5.57 - 5.83)
Soft tissue and other extraosseous sarcomas	Cancers	Q3	4,688	28,152	25.3	52.8	64.7	44.3 (42.65 - 45.87)	7.3 (7.95 - 6.68)	5.94 (5.82 - 6.06)
Soft tissue and other extraosseous sarcomas	Cancers	Q4 (Highest)	5,066	32,848	27.3	51.8	64.4	47.7 (46.19 - 49.27)	8.8 (9.6 - 8.08)	6.24 (6.12 - 6.36)
Nonrheum valv diseases	Cardiovascular diseases	All	272,699	1,685,814	100.0	46.6	72.9	44.7 (44.44 - 44.89)	8.5 (8.59 - 8.47)	6.68 (6.67 - 6.7)
Nonrheum valv diseases	Cardiovascular diseases	Q1 (Lowest)	70,452	414,499	25.7	48.4	72.1	40.4 (39.97 - 40.84)	7.5 (7.58 - 7.37)	6.35 (6.32 - 6.38)
Nonrheum valv diseases	Cardiovascular diseases	Q2	68,890	422,928	25.3	46.8	72.6	44.1 (43.63 - 44.52)	8.3 (8.45 - 8.22)	6.62 (6.59 - 6.65)
Nonrheum valv diseases	Cardiovascular diseases	Q3	68,043	428,763	25.0	46.0	73.1	46.1 (45.65 - 46.55)	8.9 (9.05 - 8.82)	6.79 (6.76 - 6.82)
Nonrheum valv diseases	Cardiovascular diseases	Q4 (Highest)	65,314	419,625	24.0	45.1	73.6	48.5 (47.99 - 48.92)	9.6 (9.72 - 9.47)	7.01 (6.98 - 7.04)
PAH	Cardiovascular diseases	All	13,422	40,424	100.0	58.6	76.1	16.0 (15.2 - 16.8)	2.2 (2.33 - 2.14)	3.64 (3.57 - 3.7)
PAH	Cardiovascular diseases	Q1 (Lowest)	4,219	11,924	31.5	58.4	73.5	14.6 (13.25 - 16.05)	2.1 (2.25 - 1.89)	3.50 (3.39 - 3.62)
PAH	Cardiovascular diseases	Q2	3,639	10,824	27.1	59.8	76.7	15.5 (14.02 - 17.07)	2.2 (2.37 - 2.02)	3.60 (3.48 - 3.73)
PAH	Cardiovascular diseases	Q3	3,052	9,469	22.7	57.2	77.5	16.2 (14.58 - 17.88)	2.3 (2.43 - 2.12)	3.65 (3.51 - 3.79)
PAH	Cardiovascular diseases	Q4 (Highest)	2,512	8,206	18.7	58.8	77.9	18.7 (16.77 - 20.62)	2.5 (2.68 - 2.26)	3.89 (3.73 - 4.05)

Appendix Table A4: Risk Ratios for Diagnosed vs. Matched Undiagnosed Controls

Kaplan-Meier survival estimates comparing diagnosed individuals to matched general population controls

Group	Diagnosis	Diag. 10y Surv % (95% CI)	Ctrl. 10y Surv % (95% CI)	Risk Ratio (95% CI)
Cancers	Brain cancer	23.6 (23.11 - 24.1)	76.53 (76.3 - 76.76)	3.26 (3.22 - 3.29)
Cancers	Ovarian cancer	33.03 (32.46 - 33.6)	75.17 (74.93 - 75.41)	2.7 (2.66 - 2.73)
Cancers	Lung cancer	10.05 (9.85 - 10.25)	63.44 (63.3 - 63.58)	2.46 (2.45 - 2.47)
Cancers	Nasopharynx cancer	40.66 (38.3 - 43.0)	75.6 (74.64 - 76.52)	2.43 (2.3 - 2.57)
Cancers	Esophageal cancer	9.69 (9.19 - 10.21)	62.48 (62.1 - 62.86)	2.41 (2.38 - 2.44)
Cancers	Liver cancer	8.3 (7.87 - 8.73)	61.59 (61.25 - 61.92)	2.39 (2.36 - 2.41)
Cancers	Malignant neoplasm of bone & articular cartilage	31.57 (30.57 - 32.58)	71.28 (70.83 - 71.73)	2.38 (2.33 - 2.43)
Cancers	Other pharynx cancer	44.16 (43.14 - 45.18)	76.1 (75.7 - 76.51)	2.34 (2.28 - 2.4)
Cancers	Pancreatic cancer	6.16 (5.92 - 6.41)	59.36 (59.13 - 59.58)	2.31 (2.29 - 2.32)
Cancers	Cervical cancer	59.25 (58.45 - 60.04)	82.03 (81.74 - 82.32)	2.27 (2.21 - 2.33)
Cancers	Gallbladder cancer	7.1 (6.71 - 7.5)	58.03 (57.68 - 58.38)	2.21 (2.19 - 2.23)
Cancers	Soft tissue and other extraosseous sarcomas	42.21 (41.46 - 42.95)	72.17 (71.85 - 72.48)	2.08 (2.04 - 2.11)
Cancers	Hodgkin lymphoma	65.38 (64.33 - 66.41)	82.7 (82.31 - 83.07)	2.0 (1.93 - 2.08)
Cancers	Stomach cancer	14.74 (14.3 - 15.2)	57.12 (56.84 - 57.41)	1.99 (1.97 - 2.01)
Cancers	Myeloma	26.25 (25.66 - 26.84)	61.31 (61.02 - 61.59)	1.91 (1.89 - 1.93)
Cancers	Larynx cancer	35.59 (34.32 - 36.86)	65.71 (65.14 - 66.28)	1.88 (1.83 - 1.93)
Cancers	Leukemia	35.72 (35.25 - 36.19)	63.95 (63.73 - 64.16)	1.78 (1.77 - 1.8)
Cancers	Kidney cancer	35.57 (35.07 - 36.08)	63.04 (62.8 - 63.27)	1.74 (1.73 - 1.76)
Cancers	Lymphoma	42.09 (41.63 - 42.54)	66.77 (66.57 - 66.96)	1.74 (1.73 - 1.76)
Cancers	Testicular cancer	87.14 (86.45 - 87.8)	92.27 (92.01 - 92.52)	1.66 (1.56 - 1.77)
Cancers	Thyroid cancer	70.54 (69.72 - 71.34)	81.95 (81.63 - 82.27)	1.63 (1.58 - 1.69)
Cancers	Lip oral cavity cancer	46.96 (46.33 - 47.58)	67.16 (66.9 - 67.43)	1.62 (1.59 - 1.64)
Cancers	Colorectal cancer	34.71 (34.46 - 34.97)	59.29 (59.17 - 59.41)	1.6 (1.6 - 1.61)
Cancers	Uterine cancer	56.48 (55.96 - 57.0)	71.57 (71.36 - 71.79)	1.53 (1.51 - 1.55)
Cancers	Breast cancer	65.48 (65.25 - 65.71)	77.27 (77.18 - 77.36)	1.52 (1.51 - 1.53)
Cancers	Bladder cancer	40.69 (40.29 - 41.08)	57.17 (56.99 - 57.35)	1.38 (1.37 - 1.4)
Cancers	Melanoma	64.16 (63.8 - 64.52)	73.62 (73.47 - 73.77)	1.36 (1.34 - 1.37)
Cancers	Prostate cancer	51.16 (50.94 - 51.38)	60.51 (60.42 - 60.61)	1.24 (1.23 - 1.24)
Cardiovascular diseases	Cardiomyopathy	49.99 (49.66 - 50.33)	71.97 (71.83 - 72.11)	1.78 (1.77 - 1.8)
Cardiovascular diseases	PAH	16.85 (16.08 - 17.63)	52.57 (52.09 - 53.06)	1.75 (1.73 - 1.78)
Cardiovascular diseases	Intracerebral hem	26.19 (25.91 - 26.47)	55.59 (55.44 - 55.73)	1.66 (1.65 - 1.67)
Cardiovascular diseases	Endocarditis	28.97 (28.4 - 29.55)	55.88 (55.59 - 56.17)	1.61 (1.59 - 1.63)
Cardiovascular diseases	Subarachnoid hem	42.97 (42.6 - 43.35)	64.16 (63.99 - 64.33)	1.59 (1.58 - 1.6)
Cardiovascular diseases	Aortic aneurysm	37.49 (37.17 - 37.81)	58.72 (58.58 - 58.87)	1.51 (1.51 - 1.52)
Cardiovascular diseases	Rheumatic heart disease	33.51 (32.92 - 34.1)	54.65 (54.37 - 54.94)	1.47 (1.45 - 1.48)
Cardiovascular diseases	Peripheral artery	34.27 (34.05 - 34.5)	54.61 (54.5 - 54.72)	1.45 (1.44 - 1.45)
Cardiovascular diseases	Heart failure	17.17 (17.08 - 17.26)	40.53 (40.48 - 40.58)	1.39 (1.39 - 1.39)
Cardiovascular diseases	Acute myocardial infarction	33.39 (33.26 - 33.52)	52.06 (52.0 - 52.12)	1.39 (1.39 - 1.39)
Cardiovascular diseases	Stroke	37.74 (37.63 - 37.85)	53.72 (53.67 - 53.77)	1.35 (1.34 - 1.35)
Cardiovascular diseases	Hypertensive heart disease	43.41 (43.04 - 43.78)	58.15 (57.98 - 58.31)	1.35 (1.34 - 1.36)
Cardiovascular diseases	Ischemic heart disease	40.62 (40.52 - 40.72)	55.76 (55.72 - 55.81)	1.34 (1.34 - 1.34)
Cardiovascular diseases	Chronic ischemic heart disease	37.84 (37.73 - 37.96)	53.5 (53.45 - 53.56)	1.34 (1.33 - 1.34)
Cardiovascular diseases	Ischemic stroke	40.09 (39.97 - 40.22)	54.24 (54.19 - 54.3)	1.31 (1.31 - 1.31)
Cardiovascular diseases	Nonrheum valv diseases	43.07 (42.86 - 43.28)	56.35 (56.25 - 56.44)	1.3 (1.3 - 1.31)
Cardiovascular diseases	Atrial fibrillation	38.04 (37.92 - 38.15)	51.95 (51.9 - 52.0)	1.29 (1.29 - 1.29)
Cardiovascular diseases	Hypertension	58.36 (58.29 - 58.44)	67.65 (67.62 - 67.68)	1.29 (1.28 - 1.29)
Cardiovascular diseases	Angina	54.03 (53.88 - 54.18)	60.66 (60.59 - 60.72)	1.17 (1.16 - 1.17)
Chronic respiratory	Interstitial lung disease	46.26 (45.84 - 46.67)	68.61 (68.43 - 68.79)	1.71 (1.7 - 1.73)
Chronic respiratory	COPD	35.73 (35.57 - 35.9)	61.74 (61.67 - 61.81)	1.68 (1.67 - 1.69)
Chronic respiratory	Pneumoconiosis	37.42 (36.39 - 38.46)	52.91 (52.43 - 53.39)	1.33 (1.3 - 1.36)
Chronic respiratory	Asthma	85.04 (84.94 - 85.13)	88.55 (88.51 - 88.59)	1.31 (1.3 - 1.32)
Chronic respiratory	Sleep Apnea	87.27 (87.13 - 87.41)	89.22 (89.16 - 89.28)	1.18 (1.17 - 1.2)
Diabetes & CKD	Diabetes type 1	57.23 (57.01 - 57.45)	73.98 (73.89 - 74.07)	1.64 (1.63 - 1.65)
Diabetes & CKD	Acute glomerulonephritis	79.15 (78.01 - 80.23)	87.2 (86.77 - 87.61)	1.63 (1.53 - 1.73)
Diabetes & CKD	Chronic kidney disease	27.1 (26.96 - 27.25)	51.85 (51.78 - 51.93)	1.51 (1.51 - 1.52)
Diabetes & CKD	Diabetes type 2	54.01 (53.89 - 54.14)	68.51 (68.46 - 68.57)	1.46 (1.46 - 1.47)
Diabetes & CKD	Diabetes	57.03 (56.91 - 57.15)	70.26 (70.21 - 70.31)	1.44 (1.44 - 1.45)
Digestive diseases	Cirrhosis	62.52 (62.27 - 62.76)	86.03 (85.95 - 86.11)	2.68 (2.66 - 2.71)
Digestive diseases	Vascular intestinal	29.69 (29.13 - 30.25)	53.96 (53.68 - 54.23)	1.53 (1.51 - 1.54)
Digestive diseases	Pancreatitis	61.48 (61.16 - 61.8)	74.3 (74.17 - 74.43)	1.5 (1.48 - 1.51)
Digestive diseases	Crohn's disease	86.53 (86.23 - 86.82)	90.52 (90.4 - 90.63)	1.42 (1.39 - 1.46)
Digestive diseases	Ulcerative colitis	75.13 (74.94 - 75.31)	82.06 (81.98 - 82.13)	1.39 (1.37 - 1.4)
Digestive diseases	Inflammatory bowel	76.4 (76.23 - 76.57)	82.92 (82.85 - 82.99)	1.38 (1.37 - 1.39)
Digestive diseases	Upper digest diseases	73.08 (72.99 - 73.18)	79.64 (79.6 - 79.68)	1.32 (1.32 - 1.33)
Digestive diseases	Gallbladder & biliary	75.08 (74.95 - 75.21)	79.83 (79.77 - 79.88)	1.24 (1.23 - 1.24)
Digestive diseases	Diverticular disease	67.63 (67.46 - 67.8)	71.46 (71.38 - 71.53)	1.13 (1.13 - 1.14)
Mental disorders & substance use	Opioid use disorders	76.18 (75.74 - 76.61)	93.62 (93.5 - 93.73)	3.73 (3.64 - 3.83)
Mental disorders & substance use	Self-harm	73.39 (73.17 - 73.6)	92.43 (92.36 - 92.49)	3.51 (3.47 - 3.55)
Mental disorders & substance use	Drug use disorders	82.99 (82.8 - 83.18)	94.67 (94.62 - 94.72)	3.19 (3.15 - 3.24)
Mental disorders & substance use	Anorexia nervosa	97.02 (96.77 - 97.25)	99.06 (99.0 - 99.12)	3.17 (2.85 - 3.52)
Mental disorders & substance use	ADHD	97.41 (97.34 - 97.48)	99.15 (99.13 - 99.17)	3.04 (2.93 - 3.15)
Mental disorders & substance use	Alcohol use disorders	76.76 (76.62 - 76.9)	91.93 (91.89 - 91.98)	2.88 (2.86 - 2.9)
Mental disorders & substance use	Schizophrenia	75.2 (74.78 - 75.61)	88.42 (88.28 - 88.56)	2.14 (2.1 - 2.19)
Mental disorders & substance use	Bipolar disorder	86.57 (86.34 - 86.8)	92.71 (92.63 - 92.79)	1.84 (1.81 - 1.88)
Mental disorders & substance use	Anxiety disorders	87.97 (87.9 - 88.04)	92.71 (92.68 - 92.74)	1.65 (1.64 - 1.66)
Mental disorders & substance use	Dysthymia	89.33 (88.94 - 89.71)	93.42 (93.27 - 93.55)	1.62 (1.55 - 1.69)
Mental disorders & substance use	Major depression	79.93 (79.82 - 80.03)	87.14 (87.1 - 87.18)	1.56 (1.55 - 1.57)
Mental disorders & substance use	Depressive disorders	80.07 (79.97 - 80.17)	87.22 (87.18 - 87.26)	1.56 (1.55 - 1.57)
Musculoskeletal disorders	Lupus	78.92 (78.28 - 79.54)	86.71 (86.47 - 86.95)	1.59 (1.53 - 1.64)
Musculoskeletal disorders	Gout	41.43 (41.12 - 41.74)	56.84 (56.7 - 56.98)	1.36 (1.35 - 1.37)
Musculoskeletal disorders	Rheumatoid arthritis	71.95 (71.69 - 72.21)	78.61 (78.5 - 78.71)	1.31 (1.3 - 1.32)
Musculoskeletal disorders	Low back pain	83.36 (83.25 - 83.47)	85.69 (85.64 - 85.73)	1.16 (1.15 - 1.17)
Musculoskeletal disorders	Neck pain	90.18 (90.04 - 90.31)	90.96 (90.9 - 91.01)	1.09 (1.07 - 1.1)
Musculoskeletal disorders	Osteoarthritis	78.7 (78.6 - 78.8)	76.84 (76.79 - 76.88)	0.92 (0.91 - 0.92)
Neurological disorders	Motor neuron disease	17.72 (16.98 - 18.48)	70.33 (69.9 - 70.75)	2.77 (2.73 - 2.82)
Neurological disorders	Idiopathic epilepsy	60.67 (60.43 - 60.9)	79.34 (79.25 - 79.43)	1.9 (1.89 - 1.92)
Neurological disorders	Multiple sclerosis	86.75 (86.33 - 87.16)	92.95 (92.81 - 93.09)	1.88 (1.81 - 1.95)
Neurological disorders	Parkinson's disease	29.45 (29.06 - 29.83)	54.88 (54.7 - 55.07)	1.56 (1.55 - 1.57)
Neurological disorders	Dementia as a sign with others	9.37 (9.18 - 9.56)	40.58 (40.44 - 40.72)	1.53 (1.52 - 1.53)
Neurological disorders	Alzheimer's disease	9.1 (8.93 - 9.28)	38.68 (38.55 - 38.8)	1.48 (1.48 - 1.49)
Neurological disorders	Vascular dementia	4.76 (4.62 - 4.91)	30.94 (30.8 - 31.08)	1.38 (1.38 - 1.38)
Neurological disorders	Alzheimer's disease and other dementias	5.74 (5.67 - 5.82)	30.83 (30.76 - 30.9)	1.36 (1.36 - 1.36)
Neurological disorders	Unspecified dementia	2.8 (2.74 - 2.87)	25.43 (25.36 - 25.51)	1.3 (1.3 - 1.31)
Other	HIV/AIDS	84.45 (84.12 - 84.78)	92.35 (92.24 - 92.46)	2.03 (1.98 - 2.09)
Other	Decubitus ulcer	16.62 (16.31 - 16.93)	47.43 (47.24 - 47.63)	1.59 (1.58 - 1.59)
Other	Hemoglobinopathies	34.93 (34.79 - 35.06)	58.07 (58.01 - 58.13)	1.55 (1.55 - 1.56)
Other	Endo/metab/blood/immune	73.18 (73.1 - 73.25)	80.9 (80.87 - 80.93)	1.4 (1.4 - 1.41)
Other	Tuberculosis	80.97 (80.63 - 81.31)	86.28 (86.14 - 86.42)	1.39 (1.36 - 1.42)
Other	Urinary tract infections and interstitial nephritis	53.99 (53.9 - 54.09)	66.41 (66.37 - 66.45)	1.37 (1.37 - 1.37)
Other	Psoriasis	85.28 (85.12 - 85.43)	87.76 (87.69 - 87.82)	1.2 (1.19 - 1.22)

Appendix Table A5: Unadjusted Median Survival and Survival Probabilities by Age, both sexes (Page 1 of 18)

Group	Diagnosis Category	Sex	Age	N	Median Survival (y)	1y (%)	3y (%)	5y (%)	10y (%)	15y (%)	20y (%)
Cancers	Bladder cancer	Both sexes	All	71838	7.5 (7.61 - 7.39)	82.7 (82.4 - 83.0)	68.4 (68.0 - 68.7)	59.3 (58.9 - 59.7)	41.7 (41.2 - 42.1)	28.8 (28.4 - 29.3)	19.2 (18.7 - 19.7)
Cancers	Bladder cancer	Both sexes	16-24	85	NR	100.0 (100.0 - 100.0)	97.5 (90.3 - 99.4)	96.1 (88.4 - 98.7)	96.1 (88.4 - 98.7)	96.1 (88.4 - 98.7)	96.1 (88.4 - 98.7)
Cancers	Bladder cancer	Both sexes	25-34	305	NR	97.9 (95.5 - 99.1)	96.9 (94.1 - 98.4)	96.1 (93.0 - 97.8)	94.3 (90.6 - 96.5)	93.7 (89.9 - 96.2)	92.1 (86.5 - 95.5)
Cancers	Bladder cancer	Both sexes	35-44	842	NR	94.2 (92.4 - 95.6)	89.1 (86.7 - 91.0)	87.2 (84.6 - 89.3)	84.8 (82.0 - 87.2)	83.0 (80.0 - 85.7)	79.5 (75.6 - 82.9)
Cancers	Bladder cancer	Both sexes	45-54	3162	NR	93.5 (92.5 - 94.3)	87.4 (86.2 - 88.5)	84.4 (83.0 - 85.7)	79.0 (77.4 - 80.5)	74.0 (72.2 - 75.8)	67.5 (65.1 - 69.8)
Cancers	Bladder cancer	Both sexes	55-64	10507	18.7 (19.24 - 18.16)	91.9 (91.4 - 92.4)	83.9 (83.2 - 84.6)	79.3 (78.4 - 80.1)	69.9 (68.9 - 70.8)	59.4 (58.2 - 60.5)	46.7 (45.2 - 48.2)
Cancers	Bladder cancer	Both sexes	65-74	22497	10.82 (11.06 - 10.57)	88.1 (87.7 - 88.5)	76.9 (76.4 - 77.5)	69.6 (68.9 - 70.2)	52.6 (51.8 - 53.4)	34.9 (34.0 - 35.8)	17.4 (16.4 - 18.5)
Cancers	Bladder cancer	Both sexes	75-84	24946	5.06 (5.16 - 4.96)	79.9 (79.3 - 80.4)	62.3 (61.7 - 62.9)	50.4 (49.7 - 51.0)	24.6 (23.9 - 25.2)	8.6 (8.1 - 9.2)	2.0 (1.6 - 2.4)
Cancers	Bladder cancer	Both sexes	85-95	9257	1.77 (1.86 - 1.7)	62.4 (61.4 - 63.4)	37.1 (36.0 - 38.1)	22.0 (21.1 - 23.0)	4.6 (4.1 - 5.2)	0.5 (0.3 - 0.8)	0.1 (0.0 - 0.5)
Cancers	Brain cancer	Both sexes	All	28259	1.08 (1.1 - 1.05)	51.6 (51.0 - 52.2)	33.1 (32.5 - 33.7)	28.0 (27.5 - 28.6)	21.7 (21.2 - 22.2)	18.1 (17.6 - 18.7)	15.2 (14.7 - 15.8)
Cancers	Brain cancer	Both sexes	16-24	944	NR	92.5 (90.6 - 94.0)	84.4 (81.9 - 86.7)	80.6 (77.9 - 83.1)	74.7 (71.6 - 77.5)	71.3 (67.9 - 74.3)	69.8 (66.2 - 73.0)
Cancers	Brain cancer	Both sexes	25-34	1509	22.63	93.9 (92.5 - 95.0)	83.3 (81.3 - 85.2)	76.2 (73.9 - 78.4)	64.3 (61.6 - 66.9)	60.1 (57.2 - 62.9)	56.2 (53.0 - 59.3)
Cancers	Brain cancer	Both sexes	35-44	2153	12.1 (13.81 - 9.91)	84.9 (83.3 - 86.3)	68.3 (66.3 - 70.3)	61.2 (59.0 - 63.3)	52.3 (50.0 - 54.6)	45.9 (43.4 - 48.3)	40.3 (37.5 - 43.1)
Cancers	Brain cancer	Both sexes	45-54	3652	2.34 (2.56 - 2.15)	71.1 (69.6 - 72.6)	45.6 (43.9 - 47.2)	39.3 (37.7 - 41.0)	31.6 (30.0 - 33.3)	27.5 (25.8 - 29.2)	22.7 (20.8 - 24.6)
Cancers	Brain cancer	Both sexes	55-64	5853	1.27 (1.33 - 1.22)	58.0 (56.7 - 59.3)	30.8 (29.6 - 32.1)	24.8 (23.7 - 26.0)	18.4 (17.3 - 19.5)	14.5 (13.5 - 15.6)	10.4 (9.3 - 11.6)
Cancers	Brain cancer	Both sexes	65-74	7107	0.74 (0.77 - 0.71)	40.8 (39.6 - 41.9)	20.9 (20.0 - 21.9)	16.4 (15.5 - 17.3)	10.7 (9.9 - 11.5)	7.1 (6.3 - 7.9)	3.7 (2.9 - 4.7)
Cancers	Brain cancer	Both sexes	75-84	5430	0.37 (0.39 - 0.35)	24.5 (23.3 - 25.7)	13.5 (12.6 - 14.4)	9.9 (9.0 - 10.7)	4.2 (3.6 - 4.9)	1.3 (0.9 - 1.8)	0.4 (0.2 - 0.8)
Cancers	Brain cancer	Both sexes	85-95	1565	0.21 (0.23 - 0.19)	15.1 (13.4 - 17.0)	6.6 (5.4 - 8.0)	3.5 (2.6 - 4.6)	0.9 (0.5 - 1.6)	0.0 (0.0 - 0.0)	0.0 (0.0 - 0.0)
Cancers	Breast cancer	Both sexes	All	189557	18.09 (18.24 - 17.92)	95.0 (94.9 - 95.1)	87.4 (87.2 - 87.5)	81.0 (80.8 - 81.2)	68.2 (68.0 - 68.4)	56.7 (56.4 - 57.0)	45.9 (45.5 - 46.2)
Cancers	Breast cancer	Both sexes	16-24	273	NR	98.2 (95.6 - 99.2)	97.4 (94.6 - 98.7)	96.6 (93.5 - 98.2)	92.8 (88.6 - 95.5)	91.9 (87.1 - 94.9)	89.7 (82.6 - 94.1)
Cancers	Breast cancer	Both sexes	25-34	2800	NR	98.2 (97.6 - 98.6)	91.7 (90.5 - 92.7)	87.3 (85.9 - 88.5)	81.1 (79.4 - 82.7)	78.4 (76.4 - 80.1)	74.1 (71.6 - 76.5)
Cancers	Breast cancer	Both sexes	35-44	13854	NR	99.1 (98.9 - 99.2)	95.6 (95.2 - 96.0)	92.4 (91.9 - 92.8)	86.1 (85.4 - 86.8)	81.7 (80.8 - 82.5)	77.3 (76.2 - 78.4)
Cancers	Breast cancer	Both sexes	45-54	33832	NR	98.7 (98.6 - 98.8)	95.6 (95.4 - 95.8)	92.8 (92.5 - 93.1)	87.3 (86.9 - 87.7)	81.5 (81.0 - 82.0)	74.9 (74.2 - 75.6)
Cancers	Breast cancer	Both sexes	55-64	46359	NR	97.7 (97.6 - 97.9)	93.6 (93.3 - 93.8)	89.8 (89.6 - 90.1)	81.6 (81.2 - 82.0)	72.9 (72.4 - 73.4)	62.0 (61.3 - 62.7)
Cancers	Breast cancer	Both sexes	65-74	50235	15.83 (16.0 - 15.65)	96.5 (96.3 - 96.6)	90.8 (90.6 - 91.1)	85.4 (85.0 - 85.7)	71.3 (70.9 - 71.8)	53.3 (52.6 - 53.9)	32.5 (31.6 - 33.3)
Cancers	Breast cancer	Both sexes	75-84	28365	7.06 (7.17 - 6.91)	89.3 (89.0 - 89.7)	74.1 (73.5 - 74.6)	61.7 (61.1 - 62.3)	35.1 (34.4 - 35.7)	14.3 (13.7 - 14.9)	3.8 (3.4 - 4.3)
Cancers	Breast cancer	Both sexes	85-95	13314	3.24 (3.31 - 3.15)	79.2 (78.5 - 79.9)	52.4 (51.5 - 53.3)	32.5 (31.7 - 33.4)	8.0 (7.4 - 8.5)	0.9 (0.7 - 1.2)	0.2 (0.0 - 0.4)
Cancers	Colorectal cancer	Both sexes	All	158366	5.39 (5.47 - 5.31)	77.3 (77.1 - 77.5)	60.6 (60.4 - 60.9)	51.4 (51.2 - 51.7)	36.8 (36.5 - 37.1)	26.2 (25.9 - 26.5)	17.8 (17.5 - 18.2)
Cancers	Colorectal cancer	Both sexes	16-24	314	NR	93.8 (90.5 - 96.0)	89.0 (84.9 - 92.1)	86.8 (82.3 - 90.2)	85.3 (80.6 - 89.0)	85.3 (80.6 - 89.0)	83.5 (77.2 - 88.2)
Cancers	Colorectal cancer	Both sexes	25-34	1039	NR	89.6 (87.6 - 91.4)	78.0 (75.3 - 80.5)	74.6 (71.6 - 77.2)	71.6 (68.5 - 74.5)	69.8 (66.5 - 72.9)	67.3 (63.4 - 70.9)
Cancers	Colorectal cancer	Both sexes	35-44	3311	NR	88.8 (87.7 - 89.9)	75.0 (73.5 - 76.5)	69.3 (67.7 - 71.0)	64.4 (62.6 - 66.2)	62.0 (60.1 - 63.9)	59.7 (57.4 - 61.8)
Cancers	Colorectal cancer	Both sexes	45-54	10015	21.06	87.7 (87.0 - 88.3)	74.4 (73.5 - 75.3)	67.8 (66.8 - 68.8)	61.0 (59.9 - 62.0)	56.4 (55.2 - 57.6)	51.9 (50.4 - 53.3)
Cancers	Colorectal cancer	Both sexes	55-64	25390	14.12 (14.6 - 13.61)	86.5 (86.1 - 86.9)	72.2 (71.7 - 72.8)	65.2 (64.5 - 65.8)	56.1 (55.4 - 56.8)	48.6 (47.8 - 49.4)	39.5 (38.5 - 40.5)
Cancers	Colorectal cancer	Both sexes	65-74	46470	8.08 (8.27 - 7.87)	82.0 (81.7 - 82.4)	67.0 (66.6 - 67.5)	58.8 (58.3 - 59.3)	45.1 (44.5 - 45.6)	31.9 (31.3 - 32.5)	17.6 (16.8 - 18.3)
Cancers	Colorectal cancer	Both sexes	75-84	51008	3.94 (4.03 - 3.86)	73.8 (73.4 - 74.2)	55.6 (55.1 - 56.0)	44.7 (44.2 - 45.1)	24.0 (23.5 - 24.4)	9.2 (8.8 - 9.6)	1.8 (1.5 - 2.1)
Cancers	Colorectal cancer	Both sexes	85-95	20294	1.43 (1.49 - 1.38)	56.8 (56.1 - 57.5)	34.8 (34.1 - 35.4)	22.4 (21.8 - 23.0)	5.8 (5.4 - 6.2)	0.8 (0.6 - 1.0)	0.0 (0.0 - 0.0)
Cancers	Esophageal cancer	Both sexes	All	15232	0.74 (0.76 - 0.72)	41.3 (40.5 - 42.1)	20.3 (19.6 - 20.9)	15.3 (14.7 - 16.0)	10.4 (9.8 - 10.9)	7.2 (6.7 - 7.8)	4.3 (3.7 - 5.0)
Cancers	Esophageal cancer	Both sexes	16-24	14	1.64	64.3 (34.3 - 83.3)	40.8 (15.6 - 64.9)	40.8 (15.6 - 64.9)	40.8 (15.6 - 64.9)	40.8 (15.6 - 64.9)	40.8 (15.6 - 64.9)
Cancers	Esophageal cancer	Both sexes	25-34	42	8.93	64.3 (47.9 - 76.7)	52.3 (36.3 - 66.0)	52.3 (36.3 - 66.0)	48.8 (32.6 - 63.1)	48.8 (32.6 - 63.1)	39.0 (18.7 - 58.9)
Cancers	Esophageal cancer	Both sexes	35-44	183	1.57 (2.37 - 1.09)	60.3 (52.7 - 67.0)	38.0 (30.8 - 45.2)	34.8 (27.7 - 41.9)	31.2 (24.2 - 38.3)	27.6 (20.6 - 35.1)	27.6 (20.6 - 35.1)
Cancers	Esophageal cancer	Both sexes	45-54	932	1.24 (1.41 - 1.06)	54.7 (51.4 - 57.9)	31.7 (28.6 - 34.8)	25.4 (22.5 - 28.3)	20.6 (17.8 - 23.5)	17.8 (15.0 - 20.7)	13.8 (10.3 - 17.7)
Cancers	Esophageal cancer	Both sexes	55-64	3062	1.01 (1.09 - 0.95)	50.2 (48.4 - 52.0)	25.7 (24.1 - 27.3)	20.9 (19.4 - 22.4)	14.9 (13.5 - 16.3)	11.7 (10.4 - 13.1)	7.5 (6.0 - 9.2)
Cancers	Esophageal cancer	Both sexes	65-74	5159	0.84 (0.88 - 0.81)	45.0 (43.7 - 46.4)	23.3 (22.1 - 24.5)	17.9 (16.8 - 19.1)	11.9 (10.9 - 13.0)	7.0 (6.0 - 8.2)	3.0 (1.9 - 4.4)
Cancers	Esophageal cancer	Both sexes	75-84	4317	0.58 (0.61 - 0.56)	34.8 (33.3 - 36.2)	14.4 (13.3 - 15.5)	9.2 (8.2 - 10.1)	4.4 (3.7 - 5.3)	1.4 (0.9 - 2.1)	0.2 (0.1 - 0.8)
Cancers	Esophageal cancer	Both sexes	85-95	1481	0.31 (0.34 - 0.29)	18.2 (16.2 - 20.2)	5.0 (3.9 - 6.3)	2.2 (1.4 - 3.1)	0.2 (0.0 - 0.9)	0.2 (0.0 - 0.9)	0.0 (0.0 - 0.0)
Cancers	Gallbladder cancer	Both sexes	All	17199	0.48 (0.5 - 0.46)	34.2 (33.5 - 34.9)	16.5 (15.9 - 17.1)	12.5 (12.0 - 13.0)	8.6 (8.1 - 9.1)	6.5 (6.0 - 7.0)	5.2 (4.6 - 5.7)
Cancers	Gallbladder cancer	Both sexes	16-24	28	3.49	63.5 (42.7 - 78.4)	52.3 (32.3 - 68.9)	48.5 (29.0 - 65.5)	44.1 (25.0 - 61.7)	44.1 (25.0 - 61.7)	44.1 (25.0 - 61.7)
Cancers	Gallbladder cancer	Both sexes	25-34	109	1.91 (4.87 - 0.95)	58.2 (48.3 - 66.9)	47.8 (38.1 - 56.9)	40.0 (30.4 - 49.3)	33.0 (23.6 - 42.6)	31.4 (22.1 - 41.1)	31.4 (22.1 - 41.1)
Cancers	Gallbladder cancer	Both sexes	35-44	318	1.34 (1.65 - 1.08)	59.6 (53.9 - 64.8)	33.4 (28.1 - 38.8)	27.8 (22.7 - 33.1)	24.4 (19.4 - 29.7)	24.4 (19.4 - 29.7)	20.8 (14.7 - 27.5)
Cancers	Gallbladder cancer	Both sexes	45-54	924	0.98 (1.1 - 0.89)	49.8 (46.5 - 53.0)	29.0 (26.0 - 32.0)	24.6 (21.7 - 27.6)	21.8 (19.0 - 24.8)	21.0 (18.1 - 24.0)	20.6 (17.7 - 23.7)

Appendix Table A5: Unadjusted Median Survival and Survival Probabilities by Age, both sexes (Page 2 of 18)

Group	Diagnosis Category	Sex	Age	N	Median Survival (y)	1y (%)	3y (%)	5y (%)	10y (%)	15y (%)	20y (%)
Cancers	Gallbladder cancer	Both sexes	55-64	2619	0.79 (0.87 - 0.73)	45.5 (43.6 - 47.4)	23.8 (22.1 - 25.5)	19.4 (17.9 - 21.0)	14.2 (12.8 - 15.8)	11.4 (10.0 - 13.0)	10.1 (8.6 - 11.8)
Cancers	Gallbladder cancer	Both sexes	65-74	5189	0.62 (0.67 - 0.59)	39.0 (37.7 - 40.4)	18.9 (17.8 - 20.0)	14.2 (13.2 - 15.2)	9.9 (8.9 - 10.9)	6.5 (5.6 - 7.6)	3.7 (2.7 - 4.9)
Cancers	Gallbladder cancer	Both sexes	75-84	5747	0.35 (0.37 - 0.33)	27.6 (26.4 - 28.8)	11.6 (10.8 - 12.5)	7.9 (7.2 - 8.7)	3.9 (3.3 - 4.6)	1.6 (1.1 - 2.3)	0.7 (0.4 - 1.4)
Cancers	Gallbladder cancer	Both sexes	85-95	2204	0.17 (0.18 - 0.16)	15.4 (13.9 - 17.0)	4.9 (4.0 - 5.9)	2.6 (1.9 - 3.4)	0.3 (0.1 - 0.7)	0.0 (0.0 - 0.0)	0.0 (0.0 - 0.0)
Cancers	Hodgkin lymphoma	Both sexes	All	8346	22.1	88.4 (87.7 - 89.1)	80.6 (79.8 - 81.5)	75.7 (74.8 - 76.7)	67.0 (65.9 - 68.1)	59.8 (58.5 - 61.0)	52.3 (50.9 - 53.8)
Cancers	Hodgkin lymphoma	Both sexes	16-24	997	NR	99.2 (98.4 - 99.6)	98.1 (97.0 - 98.8)	97.5 (96.3 - 98.3)	96.8 (95.4 - 97.8)	96.4 (94.8 - 97.5)	95.6 (93.6 - 97.0)
Cancers	Hodgkin lymphoma	Both sexes	25-34	1275	NR	99.0 (98.3 - 99.5)	98.0 (97.0 - 98.6)	97.0 (95.9 - 97.9)	95.5 (94.0 - 96.6)	94.4 (92.7 - 95.7)	92.1 (89.9 - 93.9)
Cancers	Hodgkin lymphoma	Both sexes	35-44	1049	NR	98.6 (97.7 - 99.2)	96.8 (95.5 - 97.7)	95.6 (94.0 - 96.7)	92.9 (91.0 - 94.5)	88.7 (86.1 - 90.8)	83.8 (80.5 - 86.6)
Cancers	Hodgkin lymphoma	Both sexes	45-54	947	NR	93.8 (92.1 - 95.2)	90.0 (87.9 - 91.8)	86.9 (84.5 - 89.0)	80.9 (78.1 - 83.4)	72.6 (69.1 - 75.8)	60.7 (56.3 - 64.8)
Cancers	Hodgkin lymphoma	Both sexes	55-64	1173	15.73 (16.9 - 14.45)	90.3 (88.4 - 91.9)	82.7 (80.4 - 84.8)	77.2 (74.6 - 79.6)	63.8 (60.7 - 66.7)	52.6 (49.2 - 56.0)	36.5 (32.4 - 40.6)
Cancers	Hodgkin lymphoma	Both sexes	65-74	1426	8.87 (9.48 - 7.83)	83.6 (81.6 - 85.5)	69.9 (67.4 - 72.3)	62.6 (59.9 - 65.1)	45.0 (42.0 - 47.9)	29.8 (26.6 - 33.0)	13.5 (10.4 - 17.1)
Cancers	Hodgkin lymphoma	Both sexes	75-84	1128	2.89 (3.27 - 2.23)	67.8 (64.9 - 70.5)	49.3 (46.2 - 52.2)	36.9 (33.9 - 39.9)	18.6 (15.9 - 21.5)	5.6 (3.8 - 7.9)	1.1 (0.2 - 3.2)
Cancers	Hodgkin lymphoma	Both sexes	85-95	340	1.1 (1.46 - 0.89)	53.3 (47.8 - 58.5)	30.6 (25.6 - 35.7)	14.6 (10.7 - 18.9)	3.0 (1.3 - 5.9)	0.0 (0.0 - 0.0)	0.0 (0.0 - 0.0)
Cancers	Kidney cancer	Both sexes	All	39633	5.78 (5.95 - 5.62)	75.0 (74.6 - 75.4)	60.6 (60.1 - 61.1)	52.7 (52.2 - 53.2)	38.1 (37.6 - 38.7)	27.2 (26.6 - 27.8)	18.7 (18.0 - 19.4)
Cancers	Kidney cancer	Both sexes	16-24	89	NR	95.5 (88.4 - 98.3)	87.1 (78.0 - 92.7)	84.6 (75.0 - 90.8)	82.0 (71.8 - 88.7)	76.1 (64.2 - 84.5)	73.6 (60.9 - 82.7)
Cancers	Kidney cancer	Both sexes	25-34	292	NR	92.6 (88.8 - 95.1)	87.1 (82.4 - 90.5)	85.8 (80.9 - 89.4)	84.1 (78.9 - 88.1)	80.1 (73.8 - 85.0)	80.1 (73.8 - 85.0)
Cancers	Kidney cancer	Both sexes	35-44	1022	NR	92.2 (90.3 - 93.7)	87.1 (84.8 - 89.0)	84.4 (81.9 - 86.6)	78.8 (75.8 - 81.5)	75.5 (72.1 - 78.5)	70.8 (66.3 - 74.7)
Cancers	Kidney cancer	Both sexes	45-54	3144	22.62	88.8 (87.6 - 89.9)	79.9 (78.4 - 81.3)	75.1 (73.4 - 76.6)	68.4 (66.6 - 70.2)	62.8 (60.6 - 64.8)	54.7 (51.8 - 57.6)
Cancers	Kidney cancer	Both sexes	55-64	7318	12.68 (13.4 - 12.15)	83.7 (82.8 - 84.6)	72.3 (71.2 - 73.3)	66.7 (65.6 - 67.8)	56.0 (54.7 - 57.3)	45.5 (44.0 - 47.0)	34.0 (32.1 - 36.0)
Cancers	Kidney cancer	Both sexes	65-74	12457	7.38 (7.67 - 7.09)	79.0 (78.3 - 79.7)	65.6 (64.8 - 66.5)	57.9 (57.0 - 58.8)	41.9 (40.9 - 43.0)	27.3 (26.2 - 28.5)	13.6 (12.2 - 15.0)
Cancers	Kidney cancer	Both sexes	75-84	11630	3.12 (3.29 - 2.95)	68.1 (67.3 - 69.0)	50.6 (49.7 - 51.6)	39.8 (38.9 - 40.8)	19.4 (18.5 - 20.3)	6.2 (5.5 - 6.9)	1.0 (0.7 - 1.6)
Cancers	Kidney cancer	Both sexes	85-95	3605	0.9 (0.97 - 0.83)	47.9 (46.2 - 49.5)	25.2 (23.8 - 26.7)	15.3 (14.0 - 16.6)	3.1 (2.5 - 3.9)	0.7 (0.4 - 1.2)	0.0 (0.0 - 0.0)
Cancers	Larynx cancer	Both sexes	All	5702	6.34 (6.8 - 5.97)	82.0 (80.9 - 83.0)	65.6 (64.3 - 66.9)	55.6 (54.2 - 56.9)	37.8 (36.4 - 39.2)	26.2 (24.8 - 27.6)	16.9 (15.4 - 18.4)
Cancers	Larynx cancer	Both sexes	16-24	17	NR	100.0 (100.0 - 100.0)	100.0 (100.0 - 100.0)	100.0 (100.0 - 100.0)	100.0 (100.0 - 100.0)	100.0 (100.0 - 100.0)	87.5 (38.7 - 98.1)
Cancers	Larynx cancer	Both sexes	25-34	46	NR	95.6 (83.5 - 98.9)	91.1 (78.0 - 96.6)	91.1 (78.0 - 96.6)	91.1 (78.0 - 96.6)	91.1 (78.0 - 96.6)	87.1 (70.9 - 94.6)
Cancers	Larynx cancer	Both sexes	35-44	119	NR	94.1 (88.0 - 97.1)	87.9 (80.3 - 92.6)	86.0 (78.1 - 91.2)	81.7 (73.1 - 87.9)	75.2 (65.2 - 82.7)	75.2 (65.2 - 82.7)
Cancers	Larynx cancer	Both sexes	45-54	437	16.3 (19.18 - 14.85)	89.8 (86.5 - 92.3)	78.2 (74.0 - 81.8)	73.1 (68.6 - 77.0)	64.9 (60.0 - 69.4)	54.8 (49.3 - 59.9)	43.4 (37.3 - 49.4)
Cancers	Larynx cancer	Both sexes	55-64	1382	10.75 (11.89 - 9.77)	87.8 (85.9 - 89.4)	73.6 (71.1 - 75.9)	66.5 (63.8 - 69.0)	52.3 (49.4 - 55.1)	39.5 (36.5 - 42.5)	25.3 (21.9 - 28.8)
Cancers	Larynx cancer	Both sexes	65-74	1917	6.76 (7.43 - 6.08)	84.4 (82.7 - 86.0)	67.7 (65.5 - 69.8)	57.1 (54.7 - 59.4)	37.1 (34.6 - 39.6)	21.5 (19.1 - 24.0)	10.0 (7.9 - 12.5)
Cancers	Larynx cancer	Both sexes	75-84	1398	3.66 (4.02 - 3.32)	74.4 (72.0 - 76.6)	55.1 (52.3 - 57.7)	41.2 (38.5 - 44.0)	16.7 (14.4 - 19.1)	5.8 (4.2 - 7.6)	1.4 (0.6 - 2.8)
Cancers	Larynx cancer	Both sexes	85-95	379	1.94 (2.4 - 1.42)	61.3 (56.2 - 66.0)	38.6 (33.6 - 43.6)	22.7 (18.4 - 27.3)	2.4 (1.0 - 4.7)	0.0 (0.0 - 0.0)	0.0 (0.0 - 0.0)
Cancers	Leukemia	Both sexes	All	44601	5.16 (5.3 - 5.03)	73.9 (73.5 - 74.3)	59.3 (58.8 - 59.8)	50.6 (50.1 - 51.1)	35.9 (35.4 - 36.4)	26.5 (25.9 - 27.0)	20.7 (20.1 - 21.3)
Cancers	Leukemia	Both sexes	16-24	884	NR	93.3 (91.5 - 94.8)	86.1 (83.6 - 88.3)	82.7 (80.0 - 85.1)	80.6 (77.7 - 83.2)	79.2 (76.1 - 81.9)	78.6 (75.4 - 81.4)
Cancers	Leukemia	Both sexes	25-34	1077	NR	92.3 (90.6 - 93.8)	84.4 (82.0 - 86.5)	82.5 (80.0 - 84.7)	80.6 (78.0 - 83.0)	78.6 (75.7 - 81.2)	76.7 (73.6 - 79.5)
Cancers	Leukemia	Both sexes	35-44	1644	NR	91.4 (89.9 - 92.7)	82.5 (80.6 - 84.3)	79.1 (77.0 - 81.1)	73.3 (70.8 - 75.5)	69.7 (67.0 - 72.2)	65.4 (62.1 - 68.5)
Cancers	Leukemia	Both sexes	45-54	3405	22.84	90.2 (89.2 - 91.2)	81.8 (80.4 - 83.1)	78.3 (76.8 - 79.7)	71.2 (69.5 - 72.8)	63.9 (61.8 - 65.8)	55.4 (52.8 - 57.9)
Cancers	Leukemia	Both sexes	55-64	7239	12.79 (13.41 - 12.27)	86.4 (85.6 - 87.2)	76.2 (75.2 - 77.2)	69.8 (68.7 - 70.9)	57.5 (56.2 - 58.8)	44.7 (43.2 - 46.2)	34.8 (33.0 - 36.5)
Cancers	Leukemia	Both sexes	65-74	12157	6.54 (6.8 - 6.31)	78.7 (77.9 - 79.4)	64.8 (63.9 - 65.6)	55.8 (54.8 - 56.7)	37.7 (36.7 - 38.7)	22.4 (21.4 - 23.5)	11.7 (10.5 - 12.9)
Cancers	Leukemia	Both sexes	75-84	12760	2.46 (2.56 - 2.35)	64.6 (63.7 - 65.4)	46.0 (45.1 - 46.9)	34.1 (33.2 - 35.0)	13.2 (12.4 - 13.9)	3.7 (3.3 - 4.3)	0.8 (0.5 - 1.2)
Cancers	Leukemia	Both sexes	85-95	5260	0.82 (0.88 - 0.73)	46.5 (45.2 - 47.9)	24.9 (23.7 - 26.1)	13.2 (12.2 - 14.2)	1.8 (1.4 - 2.3)	0.1 (0.0 - 0.4)	0.1 (0.0 - 0.4)
Cancers	Lip oral cavity cancer	Both sexes	All	29800	9.18 (9.42 - 8.98)	85.5 (85.1 - 85.9)	72.2 (71.7 - 72.7)	63.5 (62.9 - 64.1)	47.9 (47.2 - 48.5)	36.5 (35.8 - 37.3)	27.8 (26.9 - 28.6)
Cancers	Lip oral cavity cancer	Both sexes	16-24	248	NR	97.6 (94.6 - 98.9)	94.2 (90.4 - 96.5)	93.7 (89.8 - 96.2)	92.1 (87.7 - 95.0)	91.4 (86.8 - 94.5)	91.4 (86.8 - 94.5)
Cancers	Lip oral cavity cancer	Both sexes	25-34	618	NR	96.7 (94.9 - 97.9)	91.3 (88.7 - 93.3)	90.2 (87.4 - 92.3)	87.8 (84.8 - 90.3)	85.8 (82.4 - 88.6)	84.5 (80.7 - 87.5)
Cancers	Lip oral cavity cancer	Both sexes	35-44	1183	NR	95.5 (94.2 - 96.6)	90.0 (88.1 - 91.6)	87.7 (85.6 - 89.5)	83.4 (81.0 - 85.5)	79.5 (76.7 - 82.0)	75.8 (72.4 - 78.9)
Cancers	Lip oral cavity cancer	Both sexes	45-54	2907	22.7	93.2 (92.2 - 94.1)	84.9 (83.6 - 86.2)	81.0 (79.5 - 82.4)	74.3 (72.5 - 76.0)	68.0 (65.9 - 69.9)	59.4 (56.7 - 62.0)
Cancers	Lip oral cavity cancer	Both sexes	55-64	5962	16.65 (17.5 - 15.97)	90.1 (89.3 - 90.8)	79.4 (78.3 - 80.4)	73.6 (72.4 - 74.7)	62.3 (60.9 - 63.7)	52.9 (51.3 - 54.5)	42.5 (40.5 - 44.5)
Cancers	Lip oral cavity cancer	Both sexes	65-74	8254	10.12 (10.52 - 9.83)	86.8 (86.1 - 87.6)	74.3 (73.3 - 75.3)	66.2 (65.1 - 67.3)	50.6 (49.3 - 51.9)	34.3 (32.8 - 35.8)	18.0 (16.1 - 19.9)
Cancers	Lip oral cavity cancer	Both sexes	75-84	7236	5.62 (5.83 - 5.39)	82.2 (81.3 - 83.1)	66.0 (64.8 - 67.1)	53.6 (52.3 - 54.9)	27.2 (25.9 - 28.5)	9.8 (8.8 - 10.9)	1.5 (0.9 - 2.3)
Cancers	Lip oral cavity cancer	Both sexes	85-95	3249	2.59 (2.73 - 2.41)	69.0 (67.4 - 70.6)	45.5 (43.7 - 47.3)	28.2 (26.5 - 29.9)	6.7 (5.7 - 7.9)	0.7 (0.4 - 1.3)	0.0 (0.0 - 0.0)
Cancers	Liver cancer	Both sexes	All	16240	0.69 (0.72 - 0.66)	42.9 (42.2 - 43.7)	23.3 (22.6 - 24.0)	16.7 (16.1 - 17.4)	11.1 (10.5 - 11.7)	7.9 (7.3 - 8.6)	6.6 (6.0 - 7.3)

Appendix Table A5: Unadjusted Median Survival and Survival Probabilities by Age, both sexes (Page 3 of 18)

Group	Diagnosis Category	Sex	Age	N	Median Survival (y)	1y (%)	3y (%)	5y (%)	10y (%)	15y (%)	20y (%)
Cancers	Liver cancer	Both sexes	16-24	68	14.7	72.1 (59.8 - 81.2)	56.8 (44.1 - 67.6)	53.5 (40.8 - 64.7)	51.8 (39.1 - 63.1)	49.5 (36.6 - 61.1)	49.5 (36.6 - 61.1)
Cancers	Liver cancer	Both sexes	25-34	147	3.95 (7.35 - 1.85)	67.4 (58.9 - 74.4)	52.7 (44.0 - 60.7)	44.9 (36.2 - 53.2)	38.9 (30.2 - 47.4)	38.9 (30.2 - 47.4)	35.9 (26.2 - 45.6)
Cancers	Liver cancer	Both sexes	35-44	342	1.24 (1.66 - 1.01)	55.5 (50.0 - 60.6)	37.8 (32.6 - 43.0)	35.0 (29.9 - 40.2)	29.6 (24.5 - 34.8)	28.9 (23.8 - 34.2)	28.9 (23.8 - 34.2)
Cancers	Liver cancer	Both sexes	45-54	1175	1.08 (1.29 - 0.94)	51.6 (48.7 - 54.5)	34.3 (31.5 - 37.1)	28.0 (25.3 - 30.8)	24.6 (21.9 - 27.3)	19.9 (17.1 - 22.9)	17.3 (14.1 - 20.8)
Cancers	Liver cancer	Both sexes	55-64	3393	0.98 (1.06 - 0.9)	49.6 (47.8 - 51.2)	29.2 (27.6 - 30.8)	22.5 (21.1 - 24.1)	16.0 (14.6 - 17.5)	11.8 (10.3 - 13.5)	9.8 (8.1 - 11.7)
Cancers	Liver cancer	Both sexes	65-74	5417	0.81 (0.86 - 0.74)	45.3 (44.0 - 46.7)	24.2 (23.0 - 25.4)	16.7 (15.6 - 17.8)	9.4 (8.4 - 10.5)	4.5 (3.5 - 5.7)	2.7 (1.8 - 4.0)
Cancers	Liver cancer	Both sexes	75-84	4553	0.49 (0.54 - 0.46)	35.9 (34.5 - 37.3)	16.0 (14.9 - 17.2)	8.9 (8.0 - 10.0)	3.4 (2.7 - 4.3)	1.1 (0.6 - 1.9)	0.2 (0.0 - 1.1)
Cancers	Liver cancer	Both sexes	85-95	1129	0.23 (0.25 - 0.2)	22.1 (19.7 - 24.6)	7.3 (5.8 - 9.1)	3.6 (2.5 - 5.1)	0.9 (0.3 - 2.4)	0.0 (0.0 - 0.0)	0.0 (0.0 - 0.0)
Cancers	Lung cancer	Both sexes	All	108917	0.72 (0.73 - 0.71)	42.8 (42.5 - 43.1)	23.8 (23.6 - 24.1)	17.9 (17.7 - 18.2)	11.2 (10.9 - 11.4)	7.4 (7.2 - 7.6)	5.2 (5.0 - 5.5)
Cancers	Lung cancer	Both sexes	16-24	124	NR	82.9 (75.0 - 88.5)	75.3 (66.6 - 82.1)	74.4 (65.6 - 81.3)	71.7 (62.6 - 78.9)	71.7 (62.6 - 78.9)	69.7 (60.0 - 77.5)
Cancers	Lung cancer	Both sexes	25-34	352	NR	81.6 (77.1 - 85.3)	68.0 (62.7 - 72.7)	64.9 (59.4 - 69.8)	61.8 (56.2 - 66.9)	59.1 (53.3 - 64.5)	56.4 (49.5 - 62.6)
Cancers	Lung cancer	Both sexes	35-44	1131	2.33 (2.78 - 2.05)	67.5 (64.7 - 70.2)	46.4 (43.4 - 49.4)	42.0 (39.0 - 45.0)	37.4 (34.4 - 40.4)	35.6 (32.6 - 38.7)	34.8 (31.7 - 37.9)
Cancers	Lung cancer	Both sexes	45-54	5276	1.22 (1.28 - 1.14)	54.4 (53.0 - 55.7)	33.7 (32.4 - 35.0)	28.7 (27.4 - 30.0)	23.4 (22.2 - 24.6)	19.4 (18.2 - 20.7)	16.6 (15.2 - 17.9)
Cancers	Lung cancer	Both sexes	55-64	19742	0.98 (1.02 - 0.96)	49.6 (48.9 - 50.3)	28.8 (28.2 - 29.5)	23.0 (22.4 - 23.6)	16.4 (15.9 - 17.0)	12.1 (11.6 - 12.7)	8.7 (8.1 - 9.4)
Cancers	Lung cancer	Both sexes	65-74	38908	0.84 (0.86 - 0.82)	46.2 (45.7 - 46.7)	26.3 (25.9 - 26.8)	19.8 (19.4 - 20.3)	11.6 (11.2 - 12.0)	6.3 (6.0 - 6.7)	2.7 (2.3 - 3.1)
Cancers	Lung cancer	Both sexes	75-84	34377	0.55 (0.56 - 0.54)	37.6 (37.1 - 38.1)	19.0 (18.6 - 19.5)	12.7 (12.3 - 13.1)	5.1 (4.8 - 5.4)	1.5 (1.2 - 1.7)	0.4 (0.2 - 0.7)
Cancers	Lung cancer	Both sexes	85-95	8830	0.23 (0.24 - 0.22)	21.3 (20.4 - 22.1)	8.3 (7.7 - 8.9)	4.2 (3.7 - 4.7)	0.6 (0.4 - 0.9)	0.1 (0.0 - 0.3)	0.1 (0.0 - 0.3)
Cancers	Lymphoma	Both sexes	All	50944	7.96 (8.13 - 7.8)	79.7 (79.4 - 80.1)	67.4 (67.0 - 67.9)	59.4 (58.9 - 59.8)	44.6 (44.2 - 45.1)	34.1 (33.6 - 34.6)	27.0 (26.4 - 27.6)
Cancers	Lymphoma	Both sexes	16-24	784	NR	95.5 (93.8 - 96.8)	92.2 (90.0 - 93.9)	91.3 (89.0 - 93.1)	89.6 (87.1 - 91.6)	88.4 (85.7 - 90.6)	88.1 (85.3 - 90.3)
Cancers	Lymphoma	Both sexes	25-34	1400	NR	96.5 (95.4 - 97.4)	93.3 (91.8 - 94.5)	92.3 (90.7 - 93.6)	90.8 (89.0 - 92.2)	89.4 (87.5 - 91.1)	87.9 (85.6 - 89.9)
Cancers	Lymphoma	Both sexes	35-44	2338	NR	93.9 (92.9 - 94.8)	89.4 (88.0 - 90.6)	87.2 (85.8 - 88.6)	84.0 (82.3 - 85.5)	81.0 (79.1 - 82.8)	77.8 (75.5 - 80.0)
Cancers	Lymphoma	Both sexes	45-54	4520	NR	92.5 (91.7 - 93.2)	86.6 (85.5 - 87.5)	83.3 (82.1 - 84.4)	76.8 (75.4 - 78.1)	70.4 (68.8 - 72.0)	63.6 (61.5 - 65.6)
Cancers	Lymphoma	Both sexes	55-64	9038	15.86 (16.54 - 15.2)	89.3 (88.7 - 89.9)	80.3 (79.4 - 81.1)	75.1 (74.2 - 76.0)	63.4 (62.3 - 64.5)	51.7 (50.4 - 53.0)	40.8 (39.2 - 42.3)
Cancers	Lymphoma	Both sexes	65-74	14093	8.34 (8.67 - 8.1)	82.5 (81.8 - 83.1)	70.3 (69.5 - 71.1)	62.1 (61.3 - 63.0)	44.6 (43.6 - 45.5)	27.6 (26.6 - 28.7)	14.5 (13.3 - 15.7)
Cancers	Lymphoma	Both sexes	75-84	13933	3.69 (3.85 - 3.56)	71.3 (70.5 - 72.1)	54.7 (53.8 - 55.5)	42.2 (41.3 - 43.1)	18.7 (17.9 - 19.5)	6.2 (5.6 - 6.8)	1.5 (1.1 - 2.0)
Cancers	Lymphoma	Both sexes	85-95	4717	1.1 (1.21 - 1.02)	51.9 (50.4 - 53.3)	30.9 (29.5 - 32.3)	17.9 (16.7 - 19.1)	4.0 (3.3 - 4.8)	0.4 (0.2 - 0.8)	0.3 (0.1 - 0.7)
Cancers	Malignant neoplasm of bone & articular cartilage	Both sexes	All	7832	2.56 (2.77 - 2.33)	63.4 (62.3 - 64.4)	47.6 (46.5 - 48.7)	40.7 (39.6 - 41.8)	31.6 (30.5 - 32.7)	26.0 (24.9 - 27.2)	21.9 (20.7 - 23.1)
Cancers	Malignant neoplasm of bone & articular cartilage	Both sexes	16-24	417	NR	92.7 (89.7 - 94.8)	77.9 (73.5 - 81.7)	71.6 (66.8 - 75.8)	67.9 (62.9 - 72.4)	66.5 (61.4 - 71.1)	64.3 (58.7 - 69.4)
Cancers	Malignant neoplasm of bone & articular cartilage	Both sexes	25-34	401	NR	90.1 (86.7 - 92.7)	79.0 (74.6 - 82.8)	75.7 (71.0 - 79.7)	72.4 (67.5 - 76.7)	72.0 (67.1 - 76.3)	70.6 (65.4 - 75.2)
Cancers	Malignant neoplasm of bone & articular cartilage	Both sexes	35-44	530	NR	84.1 (80.7 - 87.0)	72.4 (68.3 - 76.0)	68.5 (64.3 - 72.4)	63.0 (58.5 - 67.1)	59.8 (55.1 - 64.2)	56.3 (51.0 - 61.2)
Cancers	Malignant neoplasm of bone & articular cartilage	Both sexes	45-54	763	11.38 (17.58 - 9.13)	78.0 (74.9 - 80.8)	66.7 (63.2 - 70.0)	60.6 (57.0 - 64.1)	51.4 (47.5 - 55.1)	47.7 (43.7 - 51.6)	44.6 (40.3 - 48.8)
Cancers	Malignant neoplasm of bone & articular cartilage	Both sexes	55-64	1437	3.67 (4.38 - 2.98)	68.3 (65.8 - 70.7)	52.6 (50.0 - 55.2)	45.7 (43.0 - 48.3)	36.9 (34.3 - 39.5)	29.9 (27.3 - 32.6)	22.1 (19.2 - 25.2)
Cancers	Malignant neoplasm of bone & articular cartilage	Both sexes	65-74	1920	1.94 (2.27 - 1.63)	59.5 (57.2 - 61.6)	42.6 (40.4 - 44.9)	35.6 (33.4 - 37.8)	24.0 (21.9 - 26.1)	13.9 (11.9 - 16.0)	6.8 (4.9 - 9.1)
Cancers	Malignant neoplasm of bone & articular cartilage	Both sexes	75-84	1743	0.86 (0.97 - 0.75)	47.2 (44.9 - 49.6)	29.0 (26.9 - 31.2)	19.9 (17.9 - 21.9)	9.0 (7.6 - 10.6)	3.5 (2.5 - 4.7)	0.8 (0.3 - 1.9)
Cancers	Malignant neoplasm of bone & articular cartilage	Both sexes	85-95	600	0.49 (0.63 - 0.39)	36.6 (32.7 - 40.5)	17.0 (14.1 - 20.2)	10.1 (7.7 - 12.8)	0.7 (0.2 - 2.1)	0.3 (0.0 - 1.7)	0.0 (0.0 - 0.0)
Cancers	Melanoma	Both sexes	All	93519	17.96 (18.29 - 17.63)	93.2 (93.1 - 93.4)	84.5 (84.3 - 84.8)	78.2 (77.9 - 78.5)	65.6 (65.2 - 66.0)	54.9 (54.5 - 55.4)	46.5 (45.9 - 47.1)
Cancers	Melanoma	Both sexes	16-24	963	NR	98.5 (97.5 - 99.1)	96.9 (95.5 - 97.8)	95.2 (93.6 - 96.4)	94.2 (92.4 - 95.6)	93.7 (91.8 - 95.2)	92.3 (89.2 - 94.5)
Cancers	Melanoma	Both sexes	25-34	3662	NR	98.5 (98.0 - 98.8)	96.3 (95.6 - 96.8)	94.9 (94.1 - 95.6)	93.1 (92.2 - 94.0)	91.8 (90.6 - 92.8)	90.9 (89.6 - 92.1)
Cancers	Melanoma	Both sexes	35-44	7609	NR	98.1 (97.7 - 98.3)	95.7 (95.2 - 96.1)	93.8 (93.2 - 94.3)	91.0 (90.2 - 91.6)	88.4 (87.4 - 89.2)	86.3 (85.1 - 87.5)
Cancers	Melanoma	Both sexes	45-54	12320	NR	97.0 (96.7 - 97.3)	94.0 (93.6 - 94.5)	91.7 (91.1 - 92.2)	87.2 (86.5 - 87.9)	83.6 (82.7 - 84.4)	79.2 (77.9 - 80.4)
Cancers	Melanoma	Both sexes	55-64	17822	NR	95.6 (95.3 - 95.9)	90.5 (90.1 - 91.0)	86.9 (86.4 - 87.4)	79.8 (79.1 - 80.5)	72.5 (71.6 - 73.4)	62.4 (61.0 - 63.8)
Cancers	Melanoma	Both sexes	65-74	23507	14.98 (15.23 - 14.74)	93.9 (93.6 - 94.2)	86.4 (85.9 - 86.8)	80.8 (80.3 - 81.4)	67.2 (66.5 - 68.0)	49.9 (48.8 - 51.0)	30.3 (28.7 - 31.9)
Cancers	Melanoma	Both sexes	75-84	19871	7.69 (7.84 - 7.53)	90.2 (89.8 - 90.6)	76.2 (75.6 - 76.9)	65.2 (64.4 - 65.9)	38.4 (37.4 - 39.3)	14.9 (13.9 - 15.8)	4.7 (3.8 - 5.6)
Cancers	Melanoma	Both sexes	85-95	7439	3.28 (3.39 - 3.16)	80.1 (79.1 - 81.0)	52.8 (51.6 - 54.0)	34.1 (32.9 - 35.3)	8.6 (7.7 - 9.4)	0.7 (0.4 - 1.1)	0.1 (0.0 - 0.4)
Cancers	Myeloma	Both sexes	All	29132	4.73 (4.84 - 4.62)	81.6 (81.2 - 82.1)	62.6 (62.1 - 63.2)	48.4 (47.8 - 49.1)	28.0 (27.4 - 28.6)	17.9 (17.2 - 18.5)	12.3 (11.6 - 13.1)
Cancers	Myeloma	Both sexes	16-24	102	NR	90.1 (82.3 - 94.5)	86.0 (77.5 - 91.5)	83.9 (75.0 - 89.8)	83.9 (75.0 - 89.8)	83.9 (75.0 - 89.8)	80.2 (68.1 - 88.1)
Cancers	Myeloma	Both sexes	25-34	190	NR	94.6 (90.3 - 97.1)	89.0 (83.5 - 92.8)	85.9 (79.8 - 90.3)	83.6 (77.0 - 88.4)	82.4 (75.3 - 87.5)	79.3 (69.5 - 86.3)
Cancers	Myeloma	Both sexes	35-44	612	NR	95.5 (93.4 - 96.9)	89.3 (86.5 - 91.6)	83.4 (79.9 - 86.3)	70.4 (65.9 - 74.4)	64.5 (59.4 - 69.1)	58.7 (51.9 - 64.8)
Cancers	Myeloma	Both sexes	45-54	1901	13.76 (15.52 - 12.74)	93.2 (91.9 - 94.2)	84.6 (82.8 - 86.2)	76.6 (74.5 - 78.6)	60.7 (58.0 - 63.2)	48.0 (44.8 - 51.1)	39.4 (35.5 - 43.3)
Cancers	Myeloma	Both sexes	55-64	4860	9.5 (9.9 - 8.95)	91.0 (90.2 - 91.8)	79.7 (78.5 - 80.8)	68.7 (67.3 - 70.1)	47.9 (46.2 - 49.5)	34.8 (33.0 - 36.6)	25.0 (22.8 - 27.2)

Appendix Table A5: Unadjusted Median Survival and Survival Probabilities by Age, both sexes (Page 4 of 18)

Group	Diagnosis Category	Sex	Age	N	Median Survival (y)	1y (%)	3y (%)	5y (%)	10y (%)	15y (%)	20y (%)
Cancers	Myeloma	Both sexes	65-74	8746	5.81 (6.01 - 5.61)	86.6 (85.8 - 87.3)	69.5 (68.5 - 70.5)	55.2 (54.1 - 56.3)	30.3 (29.0 - 31.5)	15.0 (13.8 - 16.3)	6.4 (5.3 - 7.8)
Cancers	Myeloma	Both sexes	75-84	9426	3.25 (3.37 - 3.14)	77.0 (76.2 - 77.9)	52.6 (51.6 - 53.7)	33.8 (32.7 - 34.9)	10.8 (10.0 - 11.7)	2.6 (2.1 - 3.3)	0.8 (0.5 - 1.3)
Cancers	Myeloma	Both sexes	85-95	3237	1.41 (1.52 - 1.3)	57.7 (55.9 - 59.4)	26.8 (25.2 - 28.4)	12.8 (11.5 - 14.1)	2.2 (1.6 - 2.9)	0.3 (0.1 - 0.8)	0.2 (0.1 - 0.7)
Cancers	Nasopharynx cancer	Both sexes	All	1728	6.82 (7.84 - 5.76)	79.9 (77.9 - 81.8)	64.7 (62.3 - 66.9)	55.6 (53.2 - 58.0)	42.8 (40.3 - 45.4)	33.1 (30.4 - 35.8)	24.6 (21.6 - 27.7)
Cancers	Nasopharynx cancer	Both sexes	16-24	31	NR	96.8 (79.2 - 99.5)	85.0 (64.5 - 94.1)	85.0 (64.5 - 94.1)	80.2 (58.5 - 91.4)	80.2 (58.5 - 91.4)	80.2 (58.5 - 91.4)
Cancers	Nasopharynx cancer	Both sexes	25-34	73	NR	97.2 (89.3 - 99.3)	88.2 (77.7 - 93.9)	84.9 (73.7 - 91.6)	81.1 (68.9 - 88.9)	78.6 (65.7 - 87.2)	67.4 (49.8 - 80.0)
Cancers	Nasopharynx cancer	Both sexes	35-44	127	NR	93.6 (87.6 - 96.7)	82.8 (74.8 - 88.4)	79.2 (70.8 - 85.5)	69.9 (60.3 - 77.6)	66.1 (55.4 - 74.7)	64.0 (52.9 - 73.2)
Cancers	Nasopharynx cancer	Both sexes	45-54	263	18.77	90.3 (85.9 - 93.3)	82.4 (77.1 - 86.6)	74.9 (68.9 - 79.9)	68.4 (61.8 - 74.0)	58.7 (51.2 - 65.5)	47.3 (38.3 - 55.7)
Cancers	Nasopharynx cancer	Both sexes	55-64	431	9.55 (12.69 - 7.4)	83.2 (79.3 - 86.5)	69.2 (64.5 - 73.4)	60.8 (55.8 - 65.4)	48.6 (43.4 - 53.7)	37.7 (32.2 - 43.2)	26.3 (20.0 - 33.1)
Cancers	Nasopharynx cancer	Both sexes	65-74	418	4.31 (5.72 - 3.62)	75.3 (70.9 - 79.2)	58.7 (53.7 - 63.3)	48.2 (43.1 - 53.0)	34.2 (29.2 - 39.2)	20.9 (16.1 - 26.1)	7.2 (3.0 - 13.8)
Cancers	Nasopharynx cancer	Both sexes	75-84	305	2.43 (3.17 - 1.96)	68.1 (62.5 - 73.1)	46.2 (40.4 - 51.8)	34.4 (29.0 - 39.9)	13.9 (9.9 - 18.5)	3.5 (1.5 - 6.9)	0.0 (0.0 - 0.0)
Cancers	Nasopharynx cancer	Both sexes	85-95	78	1.16 (1.93 - 0.59)	53.5 (41.8 - 63.8)	27.2 (17.8 - 37.5)	13.6 (7.0 - 22.4)	0.0 (0.0 - 0.0)	0.0 (0.0 - 0.0)	0.0 (0.0 - 0.0)
Cancers	Other pharynx cancer	Both sexes	All	10698	7.98 (8.37 - 7.52)	79.3 (78.5 - 80.0)	64.9 (64.0 - 65.8)	57.6 (56.6 - 58.6)	45.5 (44.4 - 46.6)	36.0 (34.8 - 37.2)	28.2 (26.8 - 29.6)
Cancers	Other pharynx cancer	Both sexes	16-24	54	NR	98.1 (87.1 - 99.7)	98.1 (87.1 - 99.7)	98.1 (87.1 - 99.7)	96.0 (85.0 - 99.0)	96.0 (85.0 - 99.0)	96.0 (85.0 - 99.0)
Cancers	Other pharynx cancer	Both sexes	25-34	125	NR	96.7 (91.6 - 98.8)	92.6 (86.3 - 96.1)	90.8 (84.0 - 94.8)	88.0 (80.5 - 92.7)	85.4 (77.1 - 90.9)	85.4 (77.1 - 90.9)
Cancers	Other pharynx cancer	Both sexes	35-44	425	NR	94.7 (92.1 - 96.5)	87.3 (83.6 - 90.2)	85.7 (81.9 - 88.7)	81.7 (77.5 - 85.3)	77.7 (72.9 - 81.8)	73.8 (68.2 - 78.6)
Cancers	Other pharynx cancer	Both sexes	45-54	1580	NR	92.0 (90.5 - 93.3)	84.5 (82.5 - 86.2)	79.4 (77.2 - 81.4)	72.7 (70.2 - 75.0)	64.5 (61.5 - 67.3)	56.4 (52.7 - 60.0)
Cancers	Other pharynx cancer	Both sexes	55-64	3232	12.21 (13.47 - 11.3)	84.3 (83.0 - 85.5)	71.9 (70.2 - 73.4)	65.4 (63.6 - 67.1)	54.2 (52.3 - 56.2)	44.1 (41.8 - 46.3)	32.0 (29.1 - 34.9)
Cancers	Other pharynx cancer	Both sexes	65-74	3137	4.91 (5.53 - 4.31)	76.3 (74.7 - 77.7)	58.4 (56.6 - 60.1)	49.7 (47.8 - 51.5)	33.6 (31.6 - 35.6)	19.4 (17.2 - 21.6)	8.8 (6.6 - 11.4)
Cancers	Other pharynx cancer	Both sexes	75-84	1717	2.22 (2.54 - 1.93)	64.4 (62.0 - 66.6)	45.2 (42.7 - 47.7)	34.4 (31.9 - 36.8)	16.4 (14.3 - 18.7)	6.2 (4.6 - 8.1)	2.0 (0.6 - 4.7)
Cancers	Other pharynx cancer	Both sexes	85-95	416	1.17 (1.35 - 0.93)	53.9 (48.9 - 58.6)	30.0 (25.5 - 34.6)	20.2 (16.2 - 24.4)	5.5 (3.1 - 8.8)	1.2 (0.1 - 4.7)	0.0 (0.0 - 0.0)
Cancers	Pancreatic cancer	Both sexes	All	46014	0.39 (0.4 - 0.38)	29.3 (28.9 - 29.7)	13.5 (13.2 - 13.9)	10.2 (9.9 - 10.5)	6.9 (6.6 - 7.2)	5.2 (4.9 - 5.5)	3.8 (3.5 - 4.1)
Cancers	Pancreatic cancer	Both sexes	16-24	65	NR	92.1 (81.9 - 96.6)	88.7 (77.7 - 94.5)	86.6 (74.8 - 93.1)	81.7 (68.2 - 89.9)	81.7 (68.2 - 89.9)	81.7 (68.2 - 89.9)
Cancers	Pancreatic cancer	Both sexes	25-34	171	NR	84.8 (78.3 - 89.4)	77.7 (70.4 - 83.4)	72.4 (64.5 - 78.8)	64.6 (55.8 - 72.1)	60.9 (51.0 - 69.3)	60.9 (51.0 - 69.3)
Cancers	Pancreatic cancer	Both sexes	35-44	609	2.51 (3.9 - 1.72)	63.6 (59.6 - 67.3)	47.5 (43.4 - 51.5)	44.0 (39.8 - 48.0)	39.7 (35.5 - 43.9)	36.5 (32.0 - 41.0)	33.3 (27.8 - 38.9)
Cancers	Pancreatic cancer	Both sexes	45-54	2348	1.0 (1.1 - 0.92)	50.0 (48.0 - 52.1)	28.4 (26.6 - 30.3)	24.2 (22.4 - 26.1)	19.4 (17.6 - 21.2)	17.4 (15.6 - 19.3)	14.1 (12.0 - 16.4)
Cancers	Pancreatic cancer	Both sexes	55-64	7494	0.68 (0.71 - 0.65)	39.5 (38.4 - 40.6)	19.0 (18.1 - 19.9)	14.4 (13.6 - 15.3)	10.7 (9.9 - 11.5)	8.7 (7.9 - 9.5)	6.2 (5.2 - 7.3)
Cancers	Pancreatic cancer	Both sexes	65-74	14448	0.51 (0.53 - 0.49)	33.6 (32.8 - 34.3)	14.3 (13.7 - 14.9)	10.5 (10.0 - 11.0)	6.6 (6.1 - 7.1)	4.4 (3.9 - 4.9)	2.7 (2.1 - 3.4)
Cancers	Pancreatic cancer	Both sexes	75-84	14872	0.28 (0.29 - 0.27)	22.1 (21.4 - 22.8)	9.5 (9.0 - 10.0)	6.3 (5.8 - 6.7)	2.9 (2.6 - 3.3)	1.1 (0.8 - 1.5)	0.1 (0.0 - 0.6)
Cancers	Pancreatic cancer	Both sexes	85-95	5838	0.15 (0.16 - 0.14)	10.3 (9.5 - 11.1)	2.8 (2.4 - 3.3)	1.6 (1.3 - 2.0)	0.3 (0.2 - 0.6)	0.0 (0.0 - 0.0)	0.0 (0.0 - 0.0)
Cancers	Soft tissue and other extraosseous sarcomas	Both sexes	All	18536	6.97 (7.25 - 6.65)	77.9 (77.3 - 78.5)	62.9 (62.2 - 63.6)	54.9 (54.2 - 55.7)	43.5 (42.7 - 44.3)	35.2 (34.3 - 36.0)	29.4 (28.4 - 30.3)
Cancers	Soft tissue and other extraosseous sarcomas	Both sexes	16-24	563	NR	91.1 (88.4 - 93.2)	77.1 (73.3 - 80.4)	73.4 (69.3 - 76.9)	70.4 (66.2 - 74.2)	70.4 (66.2 - 74.2)	69.0 (64.4 - 73.1)
Cancers	Soft tissue and other extraosseous sarcomas	Both sexes	25-34	809	NR	90.3 (88.1 - 92.2)	82.9 (80.1 - 85.4)	79.5 (76.5 - 82.2)	75.3 (72.0 - 78.4)	73.8 (70.3 - 77.0)	72.6 (68.8 - 76.0)
Cancers	Soft tissue and other extraosseous sarcomas	Both sexes	35-44	1320	NR	89.6 (87.8 - 91.1)	80.1 (77.8 - 82.2)	75.2 (72.6 - 77.5)	69.7 (66.9 - 72.3)	65.9 (62.9 - 68.7)	63.2 (59.8 - 66.4)
Cancers	Soft tissue and other extraosseous sarcomas	Both sexes	45-54	2163	NR	87.9 (86.5 - 89.3)	77.3 (75.4 - 79.0)	72.1 (70.1 - 74.0)	66.0 (63.8 - 68.1)	60.7 (58.3 - 63.1)	55.8 (52.9 - 58.6)
Cancers	Soft tissue and other extraosseous sarcomas	Both sexes	55-64	3472	11.3 (12.81 - 10.22)	81.5 (80.2 - 82.8)	67.5 (65.8 - 69.0)	61.3 (59.6 - 62.9)	52.1 (50.2 - 53.9)	43.7 (41.7 - 45.7)	38.1 (35.8 - 40.4)
Cancers	Soft tissue and other extraosseous sarcomas	Both sexes	65-74	4567	6.14 (6.72 - 5.58)	77.2 (76.0 - 78.4)	61.7 (60.2 - 63.1)	53.2 (51.6 - 54.7)	40.8 (39.2 - 42.4)	27.9 (26.1 - 29.7)	15.1 (13.1 - 17.3)
Cancers	Soft tissue and other extraosseous sarcomas	Both sexes	75-84	3994	3.4 (3.66 - 3.14)	70.3 (68.9 - 71.7)	52.5 (50.9 - 54.1)	41.8 (40.2 - 43.5)	21.3 (19.8 - 22.9)	8.3 (7.0 - 9.7)	2.2 (1.4 - 3.3)
Cancers	Soft tissue and other extraosseous sarcomas	Both sexes	85-95	1595	1.5 (1.73 - 1.33)	58.1 (55.6 - 60.5)	34.5 (32.1 - 36.9)	20.3 (18.2 - 22.5)	5.9 (4.6 - 7.4)	1.0 (0.5 - 1.9)	0.3 (0.1 - 1.1)
Cancers	Stomach cancer	Both sexes	All	26047	0.97 (0.99 - 0.94)	49.2 (48.6 - 49.8)	29.7 (29.1 - 30.3)	23.7 (23.2 - 24.3)	16.3 (15.8 - 16.9)	11.3 (10.8 - 11.8)	7.7 (7.2 - 8.3)
Cancers	Stomach cancer	Both sexes	16-24	37	NR	69.5 (51.7 - 81.9)	53.7 (35.7 - 68.7)	50.1 (32.2 - 65.6)	50.1 (32.2 - 65.6)	50.1 (32.2 - 65.6)	50.1 (32.2 - 65.6)
Cancers	Stomach cancer	Both sexes	25-34	172	2.54 (9.44 - 1.23)	64.1 (56.3 - 70.9)	47.6 (39.7 - 55.1)	44.7 (36.9 - 52.3)	41.4 (33.3 - 49.3)	41.4 (33.3 - 49.3)	39.2 (30.6 - 47.8)
Cancers	Stomach cancer	Both sexes	35-44	582	2.04 (2.61 - 1.59)	65.3 (61.2 - 69.0)	43.9 (39.7 - 48.0)	39.9 (35.8 - 44.1)	37.4 (33.2 - 41.5)	34.1 (29.7 - 38.5)	34.1 (29.7 - 38.5)
Cancers	Stomach cancer	Both sexes	45-54	1630	1.78 (2.01 - 1.61)	63.2 (60.8 - 65.5)	41.5 (39.0 - 44.0)	35.6 (33.2 - 38.0)	31.2 (28.8 - 33.6)	28.1 (25.6 - 30.6)	25.5 (22.6 - 28.5)
Cancers	Stomach cancer	Both sexes	55-64	4281	1.58 (1.71 - 1.46)	59.6 (58.1 - 61.1)	38.6 (37.1 - 40.2)	32.7 (31.2 - 34.1)	26.5 (25.0 - 27.9)	21.7 (20.3 - 23.2)	16.7 (15.0 - 18.4)
Cancers	Stomach cancer	Both sexes	65-74	7607	1.29 (1.35 - 1.23)	55.3 (54.1 - 56.4)	34.5 (33.4 - 35.6)	28.3 (27.3 - 29.4)	20.1 (19.0 - 21.1)	13.0 (12.0 - 14.1)	6.6 (5.5 - 8.0)
Cancers	Stomach cancer	Both sexes	75-84	8388	0.72 (0.76 - 0.68)	42.8 (41.7 - 43.8)	24.3 (23.4 - 25.3)	17.9 (17.1 - 18.8)	8.6 (7.9 - 9.3)	2.7 (2.2 - 3.3)	0.5 (0.3 - 0.9)
Cancers	Stomach cancer	Both sexes	85-95	3275	0.36 (0.39 - 0.33)	27.8 (26.3 - 29.4)	11.5 (10.4 - 12.6)	6.0 (5.2 - 6.9)	1.3 (0.9 - 1.8)	0.1 (0.0 - 0.5)	0.0 (0.0 - 0.0)
Cancers	Thyroid cancer	Both sexes	All	14590	NR	88.9 (88.4 - 89.4)	84.2 (83.6 - 84.8)	80.5 (79.8 - 81.2)	72.3 (71.5 - 73.1)	64.7 (63.7 - 65.7)	57.9 (56.6 - 59.1)
Cancers	Thyroid cancer	Both sexes	16-24	502	NR	99.6 (98.4 - 99.9)	99.4 (98.1 - 99.8)	98.9 (97.3 - 99.5)	97.3 (95.1 - 98.6)	96.4 (93.6 - 98.0)	96.4 (93.6 - 98.0)

Appendix Table A5: Unadjusted Median Survival and Survival Probabilities by Age, both sexes (Page 5 of 18)

Group	Diagnosis Category	Sex	Age	N	Median Survival (y)	1y (%)	3y (%)	5y (%)	10y (%)	15y (%)	20y (%)
Cancers	Thyroid cancer	Both sexes	25-34	1595	NR	99.4 (98.9 - 99.7)	99.4 (98.8 - 99.7)	99.0 (98.4 - 99.4)	98.5 (97.7 - 99.1)	97.8 (96.5 - 98.6)	95.7 (93.3 - 97.2)
Cancers	Thyroid cancer	Both sexes	35-44	2263	NR	99.5 (99.0 - 99.7)	98.6 (98.0 - 99.0)	98.2 (97.6 - 98.7)	97.2 (96.3 - 97.9)	95.4 (94.1 - 96.4)	93.5 (91.7 - 94.9)
Cancers	Thyroid cancer	Both sexes	45-54	2491	NR	96.0 (95.1 - 96.7)	94.4 (93.4 - 95.3)	92.8 (91.7 - 93.8)	89.3 (87.9 - 90.6)	85.9 (84.0 - 87.5)	81.6 (79.2 - 83.7)
Cancers	Thyroid cancer	Both sexes	55-64	2592	NR	91.8 (90.7 - 92.8)	87.7 (86.3 - 88.9)	84.7 (83.2 - 86.1)	76.2 (74.3 - 78.0)	68.2 (65.9 - 70.5)	59.8 (56.9 - 62.6)
Cancers	Thyroid cancer	Both sexes	65-74	2662	11.68 (12.51 - 10.68)	82.2 (80.7 - 83.6)	74.2 (72.5 - 75.9)	68.6 (66.7 - 70.4)	55.3 (53.0 - 57.5)	39.5 (36.8 - 42.2)	24.7 (21.8 - 27.7)
Cancers	Thyroid cancer	Both sexes	75-84	1944	5.21 (5.77 - 4.61)	73.2 (71.2 - 75.2)	60.9 (58.7 - 63.2)	50.7 (48.2 - 53.0)	29.0 (26.5 - 31.5)	11.8 (9.7 - 14.1)	3.5 (2.1 - 5.4)
Cancers	Thyroid cancer	Both sexes	85-95	519	0.81 (1.51 - 0.58)	49.0 (44.6 - 53.2)	35.1 (30.9 - 39.3)	23.0 (19.3 - 27.0)	6.0 (3.8 - 8.9)	1.4 (0.4 - 3.4)	1.4 (0.4 - 3.4)
Cardiovascular diseases	Acute myocardial infarction	Both sexes	All	451526	7.51 (7.56 - 7.46)	78.7 (78.6 - 78.9)	67.8 (67.7 - 68.0)	59.2 (59.0 - 59.3)	42.3 (42.1 - 42.5)	29.6 (29.4 - 29.8)	19.7 (19.5 - 19.9)
Cardiovascular diseases	Acute myocardial infarction	Both sexes	16-24	205	NR	94.1 (89.9 - 96.6)	89.9 (84.7 - 93.3)	88.1 (82.6 - 92.0)	84.2 (78.0 - 88.8)	83.2 (76.7 - 88.1)	83.2 (76.7 - 88.1)
Cardiovascular diseases	Acute myocardial infarction	Both sexes	25-34	1080	NR	96.3 (94.9 - 97.2)	94.4 (92.8 - 95.7)	93.8 (92.2 - 95.1)	90.8 (88.6 - 92.5)	87.8 (85.2 - 90.0)	84.6 (81.2 - 87.4)
Cardiovascular diseases	Acute myocardial infarction	Both sexes	35-44	7579	NR	97.3 (96.9 - 97.6)	96.3 (95.8 - 96.7)	95.1 (94.5 - 95.5)	91.6 (90.9 - 92.3)	87.0 (86.0 - 87.9)	80.0 (78.4 - 81.4)
Cardiovascular diseases	Acute myocardial infarction	Both sexes	45-54	32724	NR	96.7 (96.5 - 96.9)	95.0 (94.7 - 95.2)	93.5 (93.2 - 93.7)	88.5 (88.1 - 88.8)	81.2 (80.7 - 81.8)	71.4 (70.6 - 72.3)
Cardiovascular diseases	Acute myocardial infarction	Both sexes	55-64	74201	19.94 (20.14 - 19.75)	94.1 (93.9 - 94.2)	90.8 (90.6 - 91.0)	87.6 (87.4 - 87.8)	78.1 (77.7 - 78.4)	65.7 (65.2 - 66.1)	49.8 (49.2 - 50.4)
Cardiovascular diseases	Acute myocardial infarction	Both sexes	65-74	110435	11.82 (11.91 - 11.72)	87.9 (87.7 - 88.1)	81.2 (80.9 - 81.4)	74.7 (74.5 - 75.0)	57.1 (56.8 - 57.5)	37.0 (36.6 - 37.4)	17.5 (17.1 - 18.0)
Cardiovascular diseases	Acute myocardial infarction	Both sexes	75-84	135399	4.65 (4.69 - 4.6)	74.3 (74.0 - 74.5)	60.0 (59.8 - 60.3)	47.8 (47.6 - 48.1)	22.7 (22.5 - 23.0)	7.3 (7.1 - 7.5)	1.4 (1.2 - 1.5)
Cardiovascular diseases	Acute myocardial infarction	Both sexes	85-95	85638	1.32 (1.35 - 1.29)	54.2 (53.8 - 54.5)	32.7 (32.4 - 33.1)	18.8 (18.5 - 19.0)	3.3 (3.2 - 3.5)	0.3 (0.2 - 0.3)	0.1 (0.0 - 0.1)
Cardiovascular diseases	Angina	Both sexes	All	446351	11.86 (11.91 - 11.81)	91.7 (91.6 - 91.8)	82.9 (82.8 - 83.0)	74.8 (74.6 - 74.9)	56.2 (56.1 - 56.4)	40.5 (40.3 - 40.6)	27.6 (27.4 - 27.8)
Cardiovascular diseases	Angina	Both sexes	16-24	178	NR	98.8 (95.4 - 99.7)	98.2 (94.6 - 99.4)	97.0 (92.9 - 98.7)	95.7 (91.2 - 97.9)	95.7 (91.2 - 97.9)	95.7 (91.2 - 97.9)
Cardiovascular diseases	Angina	Both sexes	25-34	713	NR	98.7 (97.6 - 99.3)	97.2 (95.7 - 98.2)	96.9 (95.3 - 98.0)	92.8 (90.4 - 94.6)	89.7 (86.8 - 92.0)	85.5 (81.7 - 88.6)
Cardiovascular diseases	Angina	Both sexes	35-44	7093	NR	99.5 (99.3 - 99.6)	98.5 (98.1 - 98.7)	97.4 (97.0 - 97.8)	94.3 (93.7 - 94.9)	90.1 (89.2 - 90.9)	84.2 (82.9 - 85.3)
Cardiovascular diseases	Angina	Both sexes	45-54	36899	NR	98.9 (98.8 - 99.0)	97.5 (97.4 - 97.7)	96.2 (96.0 - 96.4)	91.6 (91.3 - 91.9)	85.2 (84.8 - 85.6)	76.4 (75.8 - 77.1)
Cardiovascular diseases	Angina	Both sexes	55-64	90913	22.15 (22.32 - 21.99)	98.2 (98.1 - 98.3)	95.7 (95.5 - 95.8)	93.0 (92.8 - 93.1)	84.7 (84.4 - 84.9)	73.3 (73.0 - 73.7)	57.7 (57.2 - 58.1)
Cardiovascular diseases	Angina	Both sexes	65-74	127644	14.2 (14.27 - 14.13)	95.9 (95.7 - 96.0)	90.9 (90.7 - 91.0)	85.4 (85.2 - 85.6)	68.3 (68.0 - 68.6)	46.4 (46.1 - 46.8)	23.6 (23.2 - 24.0)
Cardiovascular diseases	Angina	Both sexes	75-84	123979	7.31 (7.36 - 7.26)	89.3 (89.1 - 89.5)	76.8 (76.6 - 77.1)	64.5 (64.2 - 64.8)	34.0 (33.7 - 34.3)	12.0 (11.8 - 12.2)	2.5 (2.3 - 2.6)
Cardiovascular diseases	Angina	Both sexes	85-95	56366	2.88 (2.92 - 2.85)	73.2 (72.8 - 73.6)	48.8 (48.4 - 49.2)	30.2 (29.8 - 30.6)	6.1 (5.9 - 6.3)	0.7 (0.6 - 0.8)	0.1 (0.1 - 0.2)
Cardiovascular diseases	Aortic aneurysm	Both sexes	All	110830	8.11 (8.2 - 8.03)	85.1 (84.9 - 85.3)	73.9 (73.6 - 74.1)	63.9 (63.6 - 64.2)	42.6 (42.2 - 42.9)	26.3 (25.9 - 26.7)	16.0 (15.5 - 16.5)
Cardiovascular diseases	Aortic aneurysm	Both sexes	16-24	403	NR	99.3 (97.7 - 99.8)	98.6 (96.7 - 99.4)	98.3 (96.1 - 99.2)	95.2 (91.4 - 97.3)	92.9 (88.1 - 95.9)	92.9 (88.1 - 95.9)
Cardiovascular diseases	Aortic aneurysm	Both sexes	25-34	933	NR	98.4 (97.3 - 99.0)	97.8 (96.5 - 98.6)	96.5 (95.0 - 97.6)	94.2 (92.0 - 95.8)	92.4 (89.5 - 94.5)	88.4 (83.6 - 91.9)
Cardiovascular diseases	Aortic aneurysm	Both sexes	35-44	2003	NR	97.8 (97.0 - 98.3)	96.8 (95.9 - 97.5)	96.1 (95.0 - 96.9)	92.8 (91.3 - 94.0)	89.5 (87.4 - 91.3)	79.6 (74.9 - 83.6)
Cardiovascular diseases	Aortic aneurysm	Both sexes	45-54	5152	NR	96.8 (96.2 - 97.2)	94.6 (93.9 - 95.2)	92.8 (92.0 - 93.5)	86.5 (85.3 - 87.7)	76.7 (74.8 - 78.4)	64.9 (61.9 - 67.7)
Cardiovascular diseases	Aortic aneurysm	Both sexes	55-64	16231	16.6 (16.94 - 16.05)	94.6 (94.2 - 94.9)	90.5 (90.0 - 91.0)	85.9 (85.3 - 86.4)	71.9 (71.0 - 72.7)	55.2 (54.0 - 56.4)	37.9 (36.2 - 39.6)
Cardiovascular diseases	Aortic aneurysm	Both sexes	65-74	38224	10.71 (10.86 - 10.58)	91.5 (91.2 - 91.7)	83.8 (83.4 - 84.2)	75.9 (75.4 - 76.3)	53.4 (52.7 - 54.0)	29.1 (28.4 - 29.9)	11.7 (10.8 - 12.7)
Cardiovascular diseases	Aortic aneurysm	Both sexes	75-84	36344	4.9 (4.98 - 4.83)	80.1 (79.7 - 80.5)	64.1 (63.6 - 64.6)	49.3 (48.7 - 49.9)	19.9 (19.3 - 20.4)	5.1 (4.7 - 5.5)	0.9 (0.7 - 1.2)
Cardiovascular diseases	Aortic aneurysm	Both sexes	85-95	11217	1.57 (1.65 - 1.49)	58.2 (57.3 - 59.1)	32.9 (32.0 - 33.8)	17.3 (16.5 - 18.0)	2.0 (1.7 - 2.4)	0.2 (0.1 - 0.4)	0.1 (0.0 - 0.3)
Cardiovascular diseases	Atrial fibrillation	Both sexes	All	814748	7.24 (7.27 - 7.21)	84.0 (83.9 - 84.1)	71.0 (70.9 - 71.1)	60.2 (60.1 - 60.3)	39.8 (39.7 - 39.9)	26.2 (26.0 - 26.3)	17.2 (17.0 - 17.3)
Cardiovascular diseases	Atrial fibrillation	Both sexes	16-24	1940	NR	98.3 (97.6 - 98.8)	97.5 (96.7 - 98.1)	97.1 (96.2 - 97.8)	95.6 (94.5 - 96.5)	94.5 (93.1 - 95.6)	94.0 (92.5 - 95.2)
Cardiovascular diseases	Atrial fibrillation	Both sexes	25-34	5320	NR	99.2 (98.9 - 99.4)	98.5 (98.1 - 98.8)	98.1 (97.7 - 98.5)	96.9 (96.3 - 97.4)	95.7 (94.9 - 96.3)	94.2 (93.1 - 95.0)
Cardiovascular diseases	Atrial fibrillation	Both sexes	35-44	12381	NR	98.7 (98.5 - 98.9)	97.6 (97.3 - 97.9)	96.7 (96.3 - 97.0)	94.6 (94.1 - 95.0)	91.9 (91.2 - 92.4)	87.5 (86.4 - 88.4)
Cardiovascular diseases	Atrial fibrillation	Both sexes	45-54	35185	NR	97.7 (97.5 - 97.8)	95.5 (95.3 - 95.7)	93.6 (93.3 - 93.9)	88.4 (88.0 - 88.8)	81.9 (81.3 - 82.4)	74.2 (73.4 - 74.9)
Cardiovascular diseases	Atrial fibrillation	Both sexes	55-64	98362	20.19 (20.35 - 20.01)	95.4 (95.3 - 95.5)	91.4 (91.3 - 91.6)	87.8 (87.5 - 88.0)	77.7 (77.4 - 78.0)	65.4 (65.0 - 65.8)	50.6 (50.0 - 51.2)
Cardiovascular diseases	Atrial fibrillation	Both sexes	65-74	204529	11.98 (12.06 - 11.92)	91.7 (91.6 - 91.8)	84.4 (84.2 - 84.6)	77.6 (77.4 - 77.8)	58.5 (58.2 - 58.8)	36.9 (36.6 - 37.2)	17.4 (17.0 - 17.7)
Cardiovascular diseases	Atrial fibrillation	Both sexes	75-84	277666	5.75 (5.78 - 5.72)	83.8 (83.6 - 83.9)	69.0 (68.8 - 69.2)	55.1 (54.9 - 55.3)	25.0 (24.8 - 25.2)	7.3 (7.2 - 7.5)	1.3 (1.2 - 1.4)
Cardiovascular diseases	Atrial fibrillation	Both sexes	85-95	169889	2.2 (2.22 - 2.18)	66.1 (65.9 - 66.3)	41.0 (40.7 - 41.2)	23.2 (23.0 - 23.4)	3.6 (3.5 - 3.8)	0.3 (0.3 - 0.3)	0.0 (0.0 - 0.1)
Cardiovascular diseases	Cardiomyopathy	Both sexes	All	85014	14.47 (14.68 - 14.26)	90.8 (90.6 - 91.0)	82.6 (82.3 - 82.8)	75.6 (75.3 - 75.9)	60.8 (60.4 - 61.2)	48.9 (48.4 - 49.4)	39.0 (38.4 - 39.6)
Cardiovascular diseases	Cardiomyopathy	Both sexes	16-24	6144	NR	99.5 (99.3 - 99.6)	99.0 (98.8 - 99.3)	98.6 (98.2 - 98.9)	97.6 (97.1 - 98.0)	96.7 (96.1 - 97.3)	94.1 (92.6 - 95.2)
Cardiovascular diseases	Cardiomyopathy	Both sexes	25-34	5344	NR	99.1 (98.8 - 99.3)	98.1 (97.7 - 98.5)	97.6 (97.1 - 98.0)	96.0 (95.3 - 96.5)	94.1 (93.3 - 94.9)	91.9 (90.6 - 93.1)
Cardiovascular diseases	Cardiomyopathy	Both sexes	35-44	6437	NR	98.0 (97.7 - 98.4)	96.3 (95.8 - 96.7)	95.1 (94.5 - 95.6)	91.3 (90.5 - 92.1)	86.9 (85.8 - 87.9)	79.9 (78.1 - 81.6)
Cardiovascular diseases	Cardiomyopathy	Both sexes	45-54	10297	NR	96.6 (96.3 - 97.0)	93.4 (92.9 - 93.9)	90.7 (90.0 - 91.2)	82.4 (81.5 - 83.3)	71.8 (70.6 - 73.0)	60.5 (58.7 - 62.2)
Cardiovascular diseases	Cardiomyopathy	Both sexes	55-64	16290	16.79 (17.17 - 16.51)	94.5 (94.1 - 94.8)	88.9 (88.4 - 89.4)	84.0 (83.4 - 84.6)	70.9 (70.1 - 71.8)	55.6 (54.5 - 56.6)	39.9 (38.5 - 41.3)
Cardiovascular diseases	Cardiomyopathy	Both sexes	65-74	19478	10.07 (10.28 - 9.87)	90.5 (90.1 - 91.0)	81.3 (80.7 - 81.9)	72.5 (71.8 - 73.2)	50.3 (49.5 - 51.2)	29.7 (28.6 - 30.7)	13.8 (12.6 - 15.0)

Appendix Table A5: Unadjusted Median Survival and Survival Probabilities by Age, both sexes (Page 6 of 18)

Group	Diagnosis Category	Sex	Age	N	Median Survival (y)	1y (%)	3y (%)	5y (%)	10y (%)	15y (%)	20y (%)
Cardiovascular diseases	Cardiomyopathy	Both sexes	75-84	15929	5.23 (5.36 - 5.12)	82.9 (82.3 - 83.5)	66.2 (65.5 - 67.0)	51.7 (50.8 - 52.5)	22.8 (21.9 - 23.7)	7.3 (6.6 - 8.0)	1.4 (1.0 - 2.0)
Cardiovascular diseases	Cardiomyopathy	Both sexes	85-95	4966	2.19 (2.32 - 2.08)	65.1 (63.7 - 66.4)	40.7 (39.3 - 42.2)	23.3 (22.0 - 24.6)	3.4 (2.8 - 4.2)	0.3 (0.1 - 0.8)	0.0 (0.0 - 0.0)
Cardiovascular diseases	Chronic ischemic heart disease	Both sexes	All	609891	9.62 (9.67 - 9.58)	87.3 (87.2 - 87.3)	76.6 (76.5 - 76.8)	67.7 (67.5 - 67.8)	48.7 (48.6 - 48.8)	33.7 (33.6 - 33.9)	22.3 (22.1 - 22.5)
Cardiovascular diseases	Chronic ischemic heart disease	Both sexes	16-24	169	NR	94.6 (90.0 - 97.2)	92.7 (87.5 - 95.8)	92.0 (86.6 - 95.3)	84.6 (77.4 - 89.7)	84.6 (77.4 - 89.7)	77.5 (63.6 - 86.6)
Cardiovascular diseases	Chronic ischemic heart disease	Both sexes	25-34	1068	NR	98.6 (97.7 - 99.1)	97.2 (95.9 - 98.0)	96.6 (95.2 - 97.5)	93.1 (91.1 - 94.7)	88.5 (85.7 - 90.8)	84.3 (80.4 - 87.6)
Cardiovascular diseases	Chronic ischemic heart disease	Both sexes	35-44	8972	NR	98.8 (98.6 - 99.0)	97.6 (97.3 - 97.9)	96.5 (96.1 - 96.9)	92.9 (92.3 - 93.5)	87.6 (86.7 - 88.4)	80.5 (79.1 - 81.8)
Cardiovascular diseases	Chronic ischemic heart disease	Both sexes	45-54	43601	NR	98.4 (98.3 - 98.5)	96.6 (96.4 - 96.8)	94.9 (94.7 - 95.1)	89.3 (89.0 - 89.7)	81.5 (81.0 - 81.9)	71.2 (70.5 - 71.9)
Cardiovascular diseases	Chronic ischemic heart disease	Both sexes	55-64	111685	20.23 (20.36 - 20.07)	97.1 (97.0 - 97.2)	93.8 (93.7 - 94.0)	90.5 (90.4 - 90.7)	80.4 (80.1 - 80.6)	67.3 (66.9 - 67.6)	50.8 (50.3 - 51.3)
Cardiovascular diseases	Chronic ischemic heart disease	Both sexes	65-74	170138	12.68 (12.73 - 12.61)	93.6 (93.5 - 93.7)	87.1 (86.9 - 87.3)	80.5 (80.3 - 80.7)	61.7 (61.4 - 61.9)	39.7 (39.4 - 40.0)	18.9 (18.5 - 19.2)
Cardiovascular diseases	Chronic ischemic heart disease	Both sexes	75-84	179002	6.08 (6.12 - 6.04)	84.3 (84.2 - 84.5)	70.1 (69.9 - 70.3)	56.9 (56.7 - 57.1)	27.6 (27.4 - 27.9)	8.8 (8.6 - 9.0)	1.7 (1.6 - 1.8)
Cardiovascular diseases	Chronic ischemic heart disease	Both sexes	85-95	90757	2.09 (2.12 - 2.06)	64.6 (64.3 - 64.9)	39.9 (39.5 - 40.2)	23.4 (23.1 - 23.7)	4.2 (4.1 - 4.4)	0.4 (0.3 - 0.4)	0.0 (0.0 - 0.1)
Cardiovascular diseases	Endocarditis	Both sexes	All	23046	5.6 (5.77 - 5.4)	73.2 (72.7 - 73.8)	60.9 (60.3 - 61.6)	52.2 (51.5 - 52.8)	36.3 (35.6 - 37.1)	26.5 (25.7 - 27.3)	19.9 (19.0 - 20.8)
Cardiovascular diseases	Endocarditis	Both sexes	16-24	398	NR	96.2 (93.8 - 97.7)	93.8 (90.9 - 95.8)	91.8 (88.6 - 94.2)	87.9 (83.9 - 91.0)	83.0 (77.7 - 87.2)	72.6 (62.3 - 80.6)
Cardiovascular diseases	Endocarditis	Both sexes	25-34	908	NR	95.4 (93.9 - 96.6)	92.1 (90.1 - 93.7)	89.0 (86.6 - 90.9)	84.2 (81.3 - 86.7)	81.7 (78.3 - 84.5)	78.6 (74.3 - 82.3)
Cardiovascular diseases	Endocarditis	Both sexes	35-44	1096	NR	92.1 (90.3 - 93.5)	87.8 (85.7 - 89.6)	84.4 (82.0 - 86.5)	79.2 (76.4 - 81.7)	72.3 (68.9 - 75.4)	65.6 (61.1 - 69.7)
Cardiovascular diseases	Endocarditis	Both sexes	45-54	1807	20.28	86.4 (84.7 - 87.9)	80.2 (78.3 - 82.0)	76.3 (74.2 - 78.3)	66.8 (64.3 - 69.1)	58.4 (55.5 - 61.2)	50.3 (46.6 - 54.0)
Cardiovascular diseases	Endocarditis	Both sexes	55-64	3292	12.57 (13.27 - 11.97)	83.7 (82.4 - 84.9)	76.0 (74.5 - 77.5)	70.0 (68.3 - 71.6)	55.8 (53.9 - 57.7)	44.4 (42.1 - 46.6)	32.9 (30.0 - 35.8)
Cardiovascular diseases	Endocarditis	Both sexes	65-74	5464	7.19 (7.54 - 6.84)	76.5 (75.3 - 77.6)	66.2 (64.9 - 67.4)	58.3 (56.9 - 59.7)	38.8 (37.2 - 40.3)	22.4 (20.8 - 24.1)	9.0 (7.3 - 10.9)
Cardiovascular diseases	Endocarditis	Both sexes	75-84	6791	2.94 (3.12 - 2.78)	66.2 (65.0 - 67.3)	49.6 (48.4 - 50.8)	37.0 (35.8 - 38.3)	14.1 (13.1 - 15.2)	3.6 (2.9 - 4.4)	1.1 (0.6 - 1.8)
Cardiovascular diseases	Endocarditis	Both sexes	85-95	3181	0.96 (1.11 - 0.86)	49.6 (47.9 - 51.4)	27.6 (26.0 - 29.2)	13.9 (12.6 - 15.3)	2.0 (1.5 - 2.8)	0.1 (0.0 - 0.4)	0.1 (0.0 - 0.4)
Cardiovascular diseases	Heart failure	Both sexes	All	708074	3.66 (3.67 - 3.64)	72.9 (72.8 - 73.0)	54.7 (54.6 - 54.9)	41.5 (41.4 - 41.6)	21.6 (21.5 - 21.7)	11.7 (11.6 - 11.8)	6.5 (6.4 - 6.6)
Cardiovascular diseases	Heart failure	Both sexes	16-24	1215	NR	92.1 (90.4 - 93.5)	88.4 (86.4 - 90.2)	85.6 (83.4 - 87.6)	81.1 (78.5 - 83.5)	77.2 (74.0 - 80.1)	70.9 (66.0 - 75.2)
Cardiovascular diseases	Heart failure	Both sexes	25-34	2881	NR	93.7 (92.8 - 94.6)	89.7 (88.5 - 90.8)	87.5 (86.1 - 88.7)	81.7 (79.9 - 83.3)	77.1 (75.0 - 79.1)	72.3 (69.5 - 75.0)
Cardiovascular diseases	Heart failure	Both sexes	35-44	6955	NR	94.3 (93.7 - 94.8)	90.6 (89.9 - 91.3)	87.7 (86.9 - 88.5)	80.3 (79.2 - 81.4)	72.4 (71.0 - 73.8)	62.1 (60.0 - 64.1)
Cardiovascular diseases	Heart failure	Both sexes	45-54	22428	19.07 (19.55 - 18.67)	92.7 (92.4 - 93.1)	87.6 (87.1 - 88.0)	83.0 (82.5 - 83.5)	72.1 (71.4 - 72.8)	59.9 (59.1 - 60.8)	47.4 (46.2 - 48.6)
Cardiovascular diseases	Heart failure	Both sexes	55-64	61990	12.48 (12.63 - 12.31)	89.1 (88.9 - 89.4)	81.3 (81.0 - 81.6)	74.6 (74.2 - 74.9)	58.0 (57.6 - 58.5)	42.4 (41.9 - 42.9)	27.8 (27.1 - 28.4)
Cardiovascular diseases	Heart failure	Both sexes	65-74	138033	7.31 (7.38 - 7.25)	83.7 (83.5 - 83.8)	71.5 (71.3 - 71.8)	61.2 (61.0 - 61.5)	37.9 (37.6 - 38.2)	19.5 (19.2 - 19.8)	7.6 (7.3 - 7.9)
Cardiovascular diseases	Heart failure	Both sexes	75-84	253857	3.67 (3.69 - 3.64)	74.3 (74.1 - 74.5)	55.4 (55.2 - 55.6)	39.8 (39.6 - 40.0)	14.1 (14.0 - 14.3)	3.4 (3.3 - 3.5)	0.6 (0.5 - 0.7)
Cardiovascular diseases	Heart failure	Both sexes	85-95	207531	1.52 (1.53 - 1.5)	57.9 (57.7 - 58.1)	32.0 (31.8 - 32.2)	16.2 (16.0 - 16.4)	2.1 (2.0 - 2.1)	0.2 (0.1 - 0.2)	0.0 (0.0 - 0.1)
Cardiovascular diseases	Hypertension	Both sexes	All	2023132	13.32 (13.35 - 13.3)	92.5 (92.5 - 92.6)	85.0 (84.9 - 85.0)	77.7 (77.6 - 77.7)	60.5 (60.4 - 60.5)	45.1 (45.0 - 45.2)	32.5 (32.4 - 32.7)
Cardiovascular diseases	Hypertension	Both sexes	16-24	7092	NR	99.5 (99.2 - 99.6)	99.0 (98.8 - 99.2)	98.6 (98.3 - 98.9)	97.6 (97.1 - 98.0)	96.3 (95.6 - 96.9)	94.6 (93.5 - 95.6)
Cardiovascular diseases	Hypertension	Both sexes	25-34	22779	NR	99.5 (99.4 - 99.6)	98.8 (98.7 - 99.0)	98.2 (98.0 - 98.3)	96.7 (96.4 - 97.0)	94.4 (94.0 - 94.9)	91.6 (90.9 - 92.3)
Cardiovascular diseases	Hypertension	Both sexes	35-44	70161	NR	99.3 (99.3 - 99.4)	98.4 (98.3 - 98.5)	97.5 (97.4 - 97.6)	95.0 (94.8 - 95.1)	91.2 (90.9 - 91.5)	85.8 (85.3 - 86.3)
Cardiovascular diseases	Hypertension	Both sexes	45-54	210217	NR	98.7 (98.7 - 98.8)	97.3 (97.2 - 97.4)	95.9 (95.8 - 96.0)	91.6 (91.5 - 91.8)	85.3 (85.1 - 85.5)	76.5 (76.1 - 76.8)
Cardiovascular diseases	Hypertension	Both sexes	55-64	423549	22.05 (22.19 - 21.96)	97.5 (97.5 - 97.6)	94.8 (94.7 - 94.9)	92.1 (92.0 - 92.2)	84.1 (83.9 - 84.2)	72.8 (72.6 - 73.0)	57.5 (57.2 - 57.8)
Cardiovascular diseases	Hypertension	Both sexes	65-74	563820	14.33 (14.37 - 14.29)	95.0 (94.9 - 95.0)	89.8 (89.7 - 89.9)	84.5 (84.4 - 84.6)	68.3 (68.1 - 68.4)	46.9 (46.7 - 47.1)	24.2 (23.9 - 24.5)
Cardiovascular diseases	Hypertension	Both sexes	75-84	499706	7.63 (7.65 - 7.6)	89.4 (89.3 - 89.5)	78.1 (78.0 - 78.2)	66.2 (66.1 - 66.3)	35.8 (35.7 - 36.0)	12.9 (12.7 - 13.0)	2.6 (2.5 - 2.7)
Cardiovascular diseases	Hypertension	Both sexes	85-95	216486	3.33 (3.35 - 3.31)	76.3 (76.1 - 76.5)	53.5 (53.2 - 53.7)	34.4 (34.2 - 34.7)	7.5 (7.4 - 7.7)	0.8 (0.8 - 0.9)	0.1 (0.1 - 0.1)
Cardiovascular diseases	Hypertensive heart disease	Both sexes	All	69273	8.49 (8.59 - 8.38)	86.9 (86.6 - 87.1)	74.6 (74.3 - 74.9)	64.3 (63.9 - 64.6)	44.8 (44.4 - 45.2)	30.0 (29.6 - 30.4)	19.7 (19.3 - 20.1)
Cardiovascular diseases	Hypertensive heart disease	Both sexes	16-24	65	NR	96.9 (88.3 - 99.2)	95.3 (86.1 - 98.5)	91.8 (81.3 - 96.5)	91.8 (81.3 - 96.5)	89.5 (78.0 - 95.2)	89.5 (78.0 - 95.2)
Cardiovascular diseases	Hypertensive heart disease	Both sexes	25-34	290	NR	99.0 (96.8 - 99.7)	98.6 (96.3 - 99.5)	98.2 (95.7 - 99.3)	96.1 (92.8 - 97.9)	92.8 (88.4 - 95.6)	91.2 (86.0 - 94.5)
Cardiovascular diseases	Hypertensive heart disease	Both sexes	35-44	1247	NR	98.6 (97.8 - 99.1)	96.3 (95.0 - 97.2)	94.8 (93.3 - 95.9)	90.2 (88.2 - 91.8)	83.4 (80.9 - 85.6)	74.3 (70.7 - 77.5)
Cardiovascular diseases	Hypertensive heart disease	Both sexes	45-54	4583	NR	97.9 (97.5 - 98.3)	95.4 (94.7 - 95.9)	93.1 (92.3 - 93.8)	86.0 (84.9 - 87.0)	77.7 (76.3 - 79.1)	67.3 (65.5 - 69.1)
Cardiovascular diseases	Hypertensive heart disease	Both sexes	55-64	12043	18.9 (19.21 - 18.5)	96.3 (95.9 - 96.6)	91.9 (91.4 - 92.4)	87.4 (86.8 - 88.0)	75.8 (75.0 - 76.6)	62.0 (61.0 - 63.0)	46.1 (44.9 - 47.3)
Cardiovascular diseases	Hypertensive heart disease	Both sexes	65-74	18865	11.4 (11.58 - 11.24)	92.7 (92.3 - 93.1)	84.8 (84.3 - 85.3)	76.7 (76.1 - 77.3)	56.5 (55.8 - 57.3)	34.3 (33.5 - 35.1)	15.8 (15.0 - 16.7)
Cardiovascular diseases	Hypertensive heart disease	Both sexes	75-84	21670	5.46 (5.55 - 5.35)	84.3 (83.8 - 84.7)	67.8 (67.2 - 68.5)	52.9 (52.3 - 53.6)	24.4 (23.7 - 25.0)	7.2 (6.8 - 7.6)	1.2 (1.0 - 1.4)
Cardiovascular diseases	Hypertensive heart disease	Both sexes	85-95	10126	2.03 (2.1 - 1.96)	65.1 (64.1 - 66.0)	38.7 (37.7 - 39.6)	21.5 (20.7 - 22.3)	3.7 (3.3 - 4.1)	0.3 (0.2 - 0.4)	0.1 (0.0 - 0.2)
Cardiovascular diseases	Intracerebral hem	Both sexes	All	95308	3.94 (4.02 - 3.87)	65.2 (64.9 - 65.5)	54.4 (54.1 - 54.7)	45.6 (45.2 - 45.9)	30.2 (29.9 - 30.5)	20.9 (20.5 - 21.2)	14.9 (14.5 - 15.3)
Cardiovascular diseases	Intracerebral hem	Both sexes	16-24	971	NR	92.7 (90.8 - 94.1)	91.5 (89.5 - 93.1)	90.6 (88.5 - 92.3)	88.6 (86.3 - 90.6)	87.2 (84.5 - 89.5)	86.8 (84.0 - 89.1)
Cardiovascular diseases	Intracerebral hem	Both sexes	25-34	1530	NR	92.1 (90.6 - 93.4)	90.3 (88.7 - 91.7)	89.2 (87.5 - 90.7)	86.7 (84.7 - 88.5)	83.4 (80.9 - 85.5)	82.1 (79.1 - 84.6)

Appendix Table A5: Unadjusted Median Survival and Survival Probabilities by Age, both sexes (Page 7 of 18)

Group	Diagnosis Category	Sex	Age	N	Median Survival (y)	1y (%)	3y (%)	5y (%)	10y (%)	15y (%)	20y (%)
Cardiovascular diseases	Intracerebral hem	Both sexes	35-44	2705	NR	86.9 (85.6 - 88.1)	84.2 (82.7 - 85.5)	82.2 (80.7 - 83.6)	78.2 (76.5 - 79.8)	73.9 (71.8 - 75.8)	69.2 (66.6 - 71.7)
Cardiovascular diseases	Intracerebral hem	Both sexes	45-54	6842	22.41	83.6 (82.6 - 84.4)	79.9 (78.9 - 80.9)	77.0 (76.0 - 78.0)	70.3 (69.1 - 71.5)	63.0 (61.6 - 64.4)	55.2 (53.4 - 57.0)
Cardiovascular diseases	Intracerebral hem	Both sexes	55-64	13639	13.1 (13.49 - 12.78)	79.4 (78.7 - 80.1)	74.2 (73.5 - 74.9)	69.6 (68.8 - 70.4)	57.8 (56.9 - 58.7)	45.0 (44.0 - 46.1)	31.3 (30.0 - 32.6)
Cardiovascular diseases	Intracerebral hem	Both sexes	65-74	21716	6.21 (6.39 - 6.01)	71.1 (70.5 - 71.7)	62.5 (61.9 - 63.2)	54.9 (54.2 - 55.6)	35.4 (34.6 - 36.1)	18.9 (18.2 - 19.7)	8.3 (7.5 - 9.0)
Cardiovascular diseases	Intracerebral hem	Both sexes	75-84	29667	2.25 (2.33 - 2.16)	58.9 (58.3 - 59.4)	45.1 (44.5 - 45.7)	32.7 (32.2 - 33.3)	12.1 (11.6 - 12.5)	3.0 (2.7 - 3.3)	0.5 (0.3 - 0.7)
Cardiovascular diseases	Intracerebral hem	Both sexes	85-95	17483	0.55 (0.6 - 0.5)	44.5 (43.8 - 45.2)	26.4 (25.7 - 27.1)	14.0 (13.4 - 14.5)	1.6 (1.4 - 1.8)	0.1 (0.0 - 0.2)	0.1 (0.0 - 0.2)
Cardiovascular diseases	Ischemic heart disease	Both sexes	All	812154	10.05 (10.09 - 10.01)	85.6 (85.6 - 85.7)	76.0 (75.9 - 76.1)	67.7 (67.6 - 67.8)	50.2 (50.0 - 50.3)	35.9 (35.8 - 36.0)	24.4 (24.3 - 24.6)
Cardiovascular diseases	Ischemic heart disease	Both sexes	16-24	1884	NR	99.0 (98.5 - 99.4)	98.3 (97.6 - 98.8)	97.7 (96.9 - 98.4)	96.4 (95.2 - 97.3)	96.1 (94.7 - 97.1)	94.9 (92.3 - 96.6)
Cardiovascular diseases	Ischemic heart disease	Both sexes	25-34	5405	NR	99.1 (98.8 - 99.3)	98.3 (97.9 - 98.6)	98.0 (97.6 - 98.4)	96.0 (95.2 - 96.7)	93.5 (92.3 - 94.6)	90.6 (88.7 - 92.2)
Cardiovascular diseases	Ischemic heart disease	Both sexes	35-44	16001	NR	98.4 (98.1 - 98.5)	97.4 (97.1 - 97.6)	96.4 (96.1 - 96.7)	93.4 (93.0 - 93.8)	89.1 (88.5 - 89.7)	83.0 (82.0 - 83.9)
Cardiovascular diseases	Ischemic heart disease	Both sexes	45-54	64543	NR	97.9 (97.7 - 98.0)	96.3 (96.1 - 96.4)	94.8 (94.7 - 95.0)	90.1 (89.9 - 90.4)	83.6 (83.2 - 83.9)	74.6 (74.1 - 75.1)
Cardiovascular diseases	Ischemic heart disease	Both sexes	55-64	149758	21.49 (21.61 - 21.36)	96.2 (96.1 - 96.3)	93.4 (93.2 - 93.5)	90.5 (90.3 - 90.6)	81.8 (81.6 - 82.0)	70.3 (70.0 - 70.6)	54.9 (54.5 - 55.3)
Cardiovascular diseases	Ischemic heart disease	Both sexes	65-74	214187	13.27 (13.34 - 13.21)	92.2 (92.1 - 92.3)	86.4 (86.3 - 86.6)	80.6 (80.4 - 80.7)	63.5 (63.3 - 63.8)	42.7 (42.4 - 43.0)	21.5 (21.1 - 21.8)
Cardiovascular diseases	Ischemic heart disease	Both sexes	75-84	230103	6.04 (6.08 - 6.01)	81.7 (81.6 - 81.9)	68.5 (68.3 - 68.7)	56.2 (56.0 - 56.4)	28.5 (28.3 - 28.7)	9.8 (9.6 - 9.9)	2.0 (1.9 - 2.1)
Cardiovascular diseases	Ischemic heart disease	Both sexes	85-95	124067	2.0 (2.02 - 1.97)	62.2 (61.9 - 62.4)	39.7 (39.5 - 40.0)	23.8 (23.6 - 24.1)	4.6 (4.5 - 4.7)	0.5 (0.4 - 0.5)	0.1 (0.0 - 0.1)
Cardiovascular diseases	Ischemic stroke	Both sexes	All	661949	7.93 (7.96 - 7.9)	84.1 (84.0 - 84.2)	72.7 (72.6 - 72.8)	62.5 (62.4 - 62.7)	42.6 (42.4 - 42.7)	28.6 (28.4 - 28.7)	18.7 (18.6 - 18.9)
Cardiovascular diseases	Ischemic stroke	Both sexes	16-24	1806	NR	96.1 (95.0 - 96.9)	95.3 (94.2 - 96.2)	94.6 (93.4 - 95.6)	93.6 (92.3 - 94.7)	92.2 (90.5 - 93.6)	91.4 (89.5 - 93.0)
Cardiovascular diseases	Ischemic stroke	Both sexes	25-34	4540	NR	97.1 (96.6 - 97.6)	96.3 (95.7 - 96.8)	95.5 (94.8 - 96.1)	93.6 (92.7 - 94.3)	92.0 (91.0 - 93.0)	90.1 (88.7 - 91.4)
Cardiovascular diseases	Ischemic stroke	Both sexes	35-44	12164	NR	97.4 (97.1 - 97.7)	96.2 (95.9 - 96.6)	95.3 (94.9 - 95.7)	92.5 (92.0 - 93.0)	89.2 (88.5 - 89.9)	83.9 (82.8 - 85.1)
Cardiovascular diseases	Ischemic stroke	Both sexes	45-54	37433	NR	96.6 (96.4 - 96.8)	94.6 (94.3 - 94.8)	92.7 (92.4 - 93.0)	87.4 (87.0 - 87.8)	80.4 (79.8 - 80.9)	71.0 (70.2 - 71.8)
Cardiovascular diseases	Ischemic stroke	Both sexes	55-64	94306	19.74 (19.92 - 19.55)	95.0 (94.9 - 95.2)	91.5 (91.3 - 91.6)	88.0 (87.8 - 88.2)	77.8 (77.5 - 78.1)	65.1 (64.7 - 65.5)	49.2 (48.6 - 49.7)
Cardiovascular diseases	Ischemic stroke	Both sexes	65-74	168862	12.03 (12.1 - 11.96)	91.4 (91.3 - 91.5)	84.8 (84.6 - 85.0)	77.9 (77.7 - 78.1)	58.6 (58.3 - 58.8)	37.2 (36.9 - 37.5)	18.1 (17.7 - 18.5)
Cardiovascular diseases	Ischemic stroke	Both sexes	75-84	214460	5.7 (5.74 - 5.67)	82.6 (82.4 - 82.7)	68.4 (68.2 - 68.6)	54.6 (54.4 - 54.8)	25.4 (25.1 - 25.6)	8.1 (7.9 - 8.3)	1.6 (1.5 - 1.7)
Cardiovascular diseases	Ischemic stroke	Both sexes	85-95	122662	2.2 (2.22 - 2.17)	64.7 (64.4 - 64.9)	41.5 (41.2 - 41.8)	24.3 (24.0 - 24.5)	4.3 (4.2 - 4.5)	0.5 (0.4 - 0.5)	0.0 (0.0 - 0.1)
Cardiovascular diseases	Nonrheum valv diseases	Both sexes	All	272699	8.53 (8.59 - 8.47)	88.1 (88.0 - 88.2)	75.7 (75.6 - 75.9)	65.3 (65.1 - 65.5)	44.7 (44.4 - 44.9)	30.4 (30.1 - 30.6)	20.7 (20.5 - 21.0)
Cardiovascular diseases	Nonrheum valv diseases	Both sexes	16-24	3228	NR	99.5 (99.1 - 99.7)	99.0 (98.6 - 99.3)	98.6 (98.1 - 99.0)	97.4 (96.7 - 98.0)	95.8 (94.7 - 96.6)	94.3 (92.8 - 95.5)
Cardiovascular diseases	Nonrheum valv diseases	Both sexes	25-34	4304	NR	99.1 (98.8 - 99.3)	98.5 (98.0 - 98.8)	97.9 (97.3 - 98.3)	96.5 (95.8 - 97.1)	95.4 (94.6 - 96.1)	93.3 (92.0 - 94.4)
Cardiovascular diseases	Nonrheum valv diseases	Both sexes	35-44	6549	NR	98.9 (98.6 - 99.1)	97.5 (97.0 - 97.8)	96.2 (95.7 - 96.6)	93.3 (92.5 - 93.9)	89.2 (88.2 - 90.2)	83.6 (82.0 - 85.0)
Cardiovascular diseases	Nonrheum valv diseases	Both sexes	45-54	13774	NR	97.8 (97.6 - 98.1)	95.4 (95.0 - 95.7)	93.2 (92.7 - 93.6)	87.4 (86.7 - 88.0)	80.0 (79.1 - 80.9)	71.5 (70.2 - 72.7)
Cardiovascular diseases	Nonrheum valv diseases	Both sexes	55-64	33291	19.54 (19.77 - 19.27)	96.5 (96.3 - 96.7)	92.4 (92.1 - 92.7)	88.5 (88.1 - 88.8)	77.7 (77.2 - 78.2)	65.1 (64.4 - 65.8)	48.0 (47.0 - 49.0)
Cardiovascular diseases	Nonrheum valv diseases	Both sexes	65-74	69867	12.0 (12.11 - 11.89)	93.9 (93.7 - 94.0)	86.6 (86.3 - 86.9)	79.4 (79.1 - 79.7)	58.9 (58.5 - 59.4)	35.8 (35.2 - 36.3)	15.9 (15.2 - 16.5)
Cardiovascular diseases	Nonrheum valv diseases	Both sexes	75-84	94692	6.12 (6.17 - 6.07)	87.2 (87.0 - 87.4)	72.2 (71.9 - 72.5)	57.7 (57.4 - 58.1)	26.4 (26.0 - 26.7)	7.9 (7.6 - 8.2)	1.4 (1.2 - 1.6)
Cardiovascular diseases	Nonrheum valv diseases	Both sexes	85-95	45249	2.38 (2.42 - 2.34)	69.9 (69.5 - 70.3)	42.5 (42.0 - 43.0)	24.1 (23.7 - 24.6)	3.8 (3.6 - 4.1)	0.3 (0.2 - 0.4)	0.1 (0.0 - 0.2)
Cardiovascular diseases	PAH	Both sexes	All	13422	2.24 (2.33 - 2.14)	66.0 (65.2 - 66.8)	42.8 (41.9 - 43.7)	30.1 (29.2 - 30.9)	16.0 (15.2 - 16.8)	10.0 (9.2 - 10.8)	6.5 (5.7 - 7.4)
Cardiovascular diseases	PAH	Both sexes	16-24	82	NR	90.2 (81.4 - 95.0)	84.9 (75.0 - 91.2)	80.4 (69.4 - 87.7)	76.1 (63.9 - 84.7)	69.6 (54.5 - 80.5)	53.0 (32.5 - 69.8)
Cardiovascular diseases	PAH	Both sexes	25-34	155	NR	94.1 (89.0 - 96.9)	91.2 (85.3 - 94.8)	87.7 (80.9 - 92.2)	82.1 (73.7 - 88.0)	71.5 (59.8 - 80.3)	57.7 (39.4 - 72.3)
Cardiovascular diseases	PAH	Both sexes	35-44	206	NR	92.1 (87.5 - 95.1)	87.2 (81.6 - 91.2)	82.4 (76.1 - 87.2)	72.3 (64.6 - 78.6)	66.6 (57.9 - 73.9)	59.7 (48.4 - 69.2)
Cardiovascular diseases	PAH	Both sexes	45-54	418	11.54 (14.29 - 8.7)	87.1 (83.4 - 90.0)	75.6 (71.1 - 79.6)	66.9 (61.8 - 71.4)	52.7 (46.9 - 58.1)	42.7 (36.3 - 48.9)	34.8 (27.7 - 41.9)
Cardiovascular diseases	PAH	Both sexes	55-64	1087	5.14 (5.94 - 4.64)	78.7 (76.1 - 81.0)	62.2 (59.1 - 65.1)	50.6 (47.3 - 53.8)	35.7 (32.3 - 39.1)	24.2 (20.7 - 28.0)	15.2 (11.3 - 19.7)
Cardiovascular diseases	PAH	Both sexes	65-74	2951	3.08 (3.28 - 2.87)	72.9 (71.2 - 74.5)	50.6 (48.7 - 52.5)	37.4 (35.4 - 39.3)	20.3 (18.5 - 22.2)	10.7 (9.0 - 12.6)	4.1 (2.5 - 6.5)
Cardiovascular diseases	PAH	Both sexes	75-84	5476	1.99 (2.11 - 1.88)	64.5 (63.2 - 65.8)	39.4 (38.0 - 40.7)	25.2 (23.9 - 26.5)	7.8 (6.8 - 8.9)	2.1 (1.5 - 3.0)	0.3 (0.1 - 0.9)
Cardiovascular diseases	PAH	Both sexes	85-95	2962	1.04 (1.12 - 0.96)	51.1 (49.3 - 52.9)	22.5 (20.8 - 24.1)	9.7 (8.5 - 11.0)	1.0 (0.5 - 1.7)	0.0 (0.0 - 0.0)	0.0 (0.0 - 0.0)
Cardiovascular diseases	Peripheral artery	Both sexes	All	193190	6.36 (6.41 - 6.3)	82.2 (82.0 - 82.3)	67.9 (67.6 - 68.1)	56.6 (56.4 - 56.8)	35.6 (35.3 - 35.8)	22.1 (21.8 - 22.3)	13.6 (13.3 - 13.9)
Cardiovascular diseases	Peripheral artery	Both sexes	16-24	1896	NR	99.8 (99.5 - 99.9)	99.8 (99.5 - 99.9)	99.6 (99.2 - 99.8)	99.2 (98.5 - 99.5)	99.0 (98.2 - 99.4)	98.1 (96.7 - 99.0)
Cardiovascular diseases	Peripheral artery	Both sexes	25-34	2464	NR	99.5 (99.1 - 99.7)	98.8 (98.3 - 99.2)	98.2 (97.6 - 98.7)	97.2 (96.3 - 97.8)	96.3 (95.1 - 97.1)	94.5 (92.6 - 95.9)
Cardiovascular diseases	Peripheral artery	Both sexes	35-44	4394	NR	98.9 (98.5 - 99.1)	97.3 (96.7 - 97.7)	96.1 (95.4 - 96.7)	93.3 (92.4 - 94.1)	89.5 (88.2 - 90.7)	85.2 (83.3 - 86.9)
Cardiovascular diseases	Peripheral artery	Both sexes	45-54	9194	NR	97.2 (96.9 - 97.6)	93.6 (93.1 - 94.1)	90.3 (89.6 - 90.9)	81.0 (80.1 - 81.9)	70.3 (69.1 - 71.5)	58.2 (56.5 - 59.8)
Cardiovascular diseases	Peripheral artery	Both sexes	55-64	25182	14.4 (14.65 - 14.14)	94.6 (94.3 - 94.9)	88.1 (87.7 - 88.5)	81.6 (81.1 - 82.1)	64.8 (64.1 - 65.5)	48.0 (47.2 - 48.8)	31.1 (30.1 - 32.0)
Cardiovascular diseases	Peripheral artery	Both sexes	65-74	51392	8.92 (9.02 - 8.82)	90.4 (90.2 - 90.7)	79.4 (79.0 - 79.7)	69.5 (69.0 - 69.9)	44.7 (44.2 - 45.2)	23.8 (23.3 - 24.3)	9.0 (8.5 - 9.5)
Cardiovascular diseases	Peripheral artery	Both sexes	75-84	62344	4.65 (4.7 - 4.59)	80.2 (79.9 - 80.5)	62.5 (62.1 - 62.9)	47.5 (47.1 - 47.9)	19.4 (19.0 - 19.7)	5.2 (5.0 - 5.5)	0.7 (0.6 - 0.9)

Appendix Table A5: Unadjusted Median Survival and Survival Probabilities by Age, both sexes (Page 8 of 18)

Group	Diagnosis Category	Sex	Age	N	Median Survival (y)	1y (%)	3y (%)	5y (%)	10y (%)	15y (%)	20y (%)
Cardiovascular diseases	Peripheral artery	Both sexes	85-95	34560	1.59 (1.63 - 1.54)	58.3 (57.8 - 58.9)	33.6 (33.1 - 34.1)	18.5 (18.1 - 18.9)	2.9 (2.7 - 3.1)	0.3 (0.2 - 0.4)	0.0 (0.0 - 0.1)
Cardiovascular diseases	Rheumatic heart disease	Both sexes	All	31613	5.94 (6.06 - 5.78)	81.1 (80.6 - 81.5)	65.6 (65.1 - 66.2)	54.3 (53.7 - 54.9)	36.5 (35.8 - 37.2)	25.9 (25.2 - 26.6)	19.1 (18.3 - 19.9)
Cardiovascular diseases	Rheumatic heart disease	Both sexes	16-24	368	NR	98.6 (96.7 - 99.4)	97.1 (94.7 - 98.5)	95.5 (92.7 - 97.3)	94.2 (90.9 - 96.4)	91.4 (87.0 - 94.3)	91.4 (87.0 - 94.3)
Cardiovascular diseases	Rheumatic heart disease	Both sexes	25-34	699	NR	98.8 (97.7 - 99.4)	96.8 (95.1 - 97.9)	95.3 (93.3 - 96.7)	92.3 (89.7 - 94.3)	91.0 (88.0 - 93.2)	89.4 (85.9 - 92.1)
Cardiovascular diseases	Rheumatic heart disease	Both sexes	35-44	978	NR	97.9 (96.8 - 98.7)	96.7 (95.4 - 97.7)	94.7 (93.0 - 96.0)	91.2 (89.0 - 93.0)	87.3 (84.5 - 89.7)	80.5 (76.4 - 84.0)
Cardiovascular diseases	Rheumatic heart disease	Both sexes	45-54	1516	NR	95.1 (93.9 - 96.1)	91.3 (89.7 - 92.6)	88.0 (86.2 - 89.6)	80.9 (78.5 - 83.0)	73.7 (70.7 - 76.4)	64.7 (60.9 - 68.2)
Cardiovascular diseases	Rheumatic heart disease	Both sexes	55-64	3052	16.1 (16.91 - 15.16)	91.9 (90.8 - 92.8)	84.5 (83.1 - 85.8)	79.0 (77.4 - 80.5)	66.3 (64.3 - 68.2)	52.8 (50.4 - 55.1)	38.7 (35.8 - 41.6)
Cardiovascular diseases	Rheumatic heart disease	Both sexes	65-74	6595	9.12 (9.51 - 8.67)	87.5 (86.6 - 88.2)	76.3 (75.2 - 77.4)	66.6 (65.3 - 67.8)	46.4 (44.9 - 48.0)	26.9 (25.2 - 28.7)	11.2 (9.5 - 12.9)
Cardiovascular diseases	Rheumatic heart disease	Both sexes	75-84	11810	4.63 (4.77 - 4.5)	80.4 (79.7 - 81.1)	62.5 (61.5 - 63.4)	46.9 (45.8 - 47.9)	19.3 (18.3 - 20.3)	5.2 (4.5 - 6.0)	0.9 (0.5 - 1.4)
Cardiovascular diseases	Rheumatic heart disease	Both sexes	85-95	6355	1.78 (1.88 - 1.7)	62.3 (61.1 - 63.5)	34.3 (33.0 - 35.5)	18.3 (17.2 - 19.5)	2.7 (2.1 - 3.4)	0.1 (0.0 - 0.5)	0.1 (0.0 - 0.5)
Cardiovascular diseases	Stroke	Both sexes	All	781926	7.73 (7.76 - 7.7)	82.1 (82.0 - 82.2)	71.0 (70.9 - 71.1)	61.2 (61.1 - 61.4)	42.1 (42.0 - 42.3)	28.8 (28.7 - 29.0)	19.5 (19.3 - 19.6)
Cardiovascular diseases	Stroke	Both sexes	16-24	3579	NR	95.6 (94.9 - 96.3)	94.5 (93.7 - 95.2)	93.9 (93.0 - 94.6)	92.5 (91.5 - 93.3)	90.9 (89.8 - 92.0)	89.4 (87.8 - 90.8)
Cardiovascular diseases	Stroke	Both sexes	25-34	7550	NR	96.0 (95.6 - 96.4)	95.0 (94.4 - 95.4)	94.2 (93.7 - 94.7)	92.3 (91.6 - 92.9)	90.4 (89.5 - 91.2)	88.1 (86.9 - 89.2)
Cardiovascular diseases	Stroke	Both sexes	35-44	17550	NR	95.5 (95.2 - 95.8)	94.1 (93.8 - 94.5)	93.1 (92.7 - 93.5)	90.2 (89.7 - 90.6)	86.9 (86.3 - 87.5)	81.9 (81.0 - 82.8)
Cardiovascular diseases	Stroke	Both sexes	45-54	48859	NR	94.6 (94.3 - 94.8)	92.4 (92.1 - 92.6)	90.4 (90.1 - 90.7)	85.1 (84.8 - 85.5)	78.4 (77.9 - 78.9)	69.4 (68.7 - 70.1)
Cardiovascular diseases	Stroke	Both sexes	55-64	112787	19.35 (19.48 - 19.17)	93.0 (92.9 - 93.2)	89.3 (89.2 - 89.5)	85.9 (85.7 - 86.1)	75.8 (75.5 - 76.0)	63.4 (63.0 - 63.7)	47.8 (47.3 - 48.3)
Cardiovascular diseases	Stroke	Both sexes	65-74	194544	11.66 (11.72 - 11.6)	89.3 (89.2 - 89.5)	82.7 (82.5 - 82.9)	75.8 (75.6 - 76.0)	56.8 (56.6 - 57.1)	36.0 (35.7 - 36.3)	17.6 (17.2 - 17.9)
Cardiovascular diseases	Stroke	Both sexes	75-84	246719	5.42 (5.46 - 5.39)	80.1 (80.0 - 80.3)	66.3 (66.1 - 66.5)	52.7 (52.5 - 52.9)	24.3 (24.1 - 24.5)	7.7 (7.5 - 7.8)	1.5 (1.4 - 1.6)
Cardiovascular diseases	Stroke	Both sexes	85-95	143461	2.01 (2.03 - 1.98)	62.1 (61.8 - 62.3)	39.7 (39.4 - 39.9)	23.1 (22.9 - 23.3)	4.1 (4.0 - 4.2)	0.4 (0.4 - 0.5)	0.0 (0.0 - 0.1)
Cardiovascular diseases	Subarachnoid hem	Both sexes	All	75556	8.16 (8.3 - 8.0)	78.3 (78.0 - 78.6)	68.0 (67.6 - 68.3)	59.8 (59.4 - 60.2)	45.5 (45.1 - 45.9)	36.4 (35.9 - 36.8)	29.2 (28.7 - 29.8)
Cardiovascular diseases	Subarachnoid hem	Both sexes	16-24	917	NR	96.7 (95.3 - 97.7)	95.3 (93.7 - 96.5)	95.0 (93.4 - 96.3)	93.8 (92.0 - 95.3)	91.7 (89.3 - 93.5)	89.4 (85.9 - 92.1)
Cardiovascular diseases	Subarachnoid hem	Both sexes	25-34	1825	NR	96.8 (95.8 - 97.5)	95.6 (94.5 - 96.4)	95.1 (94.0 - 96.0)	93.6 (92.3 - 94.7)	91.7 (90.0 - 93.1)	89.0 (86.5 - 91.1)
Cardiovascular diseases	Subarachnoid hem	Both sexes	35-44	3931	NR	94.0 (93.2 - 94.7)	92.5 (91.7 - 93.3)	91.6 (90.6 - 92.4)	88.9 (87.8 - 89.9)	86.3 (85.0 - 87.5)	82.1 (80.3 - 83.8)
Cardiovascular diseases	Subarachnoid hem	Both sexes	45-54	8174	NR	92.0 (91.3 - 92.5)	89.6 (88.9 - 90.3)	87.6 (86.8 - 88.3)	82.8 (81.9 - 83.6)	76.7 (75.6 - 77.8)	68.8 (67.2 - 70.3)
Cardiovascular diseases	Subarachnoid hem	Both sexes	55-64	12649	17.78 (18.23 - 17.34)	87.9 (87.3 - 88.5)	83.3 (82.6 - 83.9)	79.1 (78.3 - 79.8)	68.6 (67.7 - 69.5)	57.3 (56.2 - 58.4)	43.4 (41.9 - 44.9)
Cardiovascular diseases	Subarachnoid hem	Both sexes	65-74	16910	9.57 (9.78 - 9.37)	82.5 (81.9 - 83.1)	74.2 (73.5 - 74.9)	66.6 (65.9 - 67.4)	48.2 (47.3 - 49.1)	31.1 (30.1 - 32.2)	16.4 (15.1 - 17.8)
Cardiovascular diseases	Subarachnoid hem	Both sexes	75-84	19334	3.94 (4.07 - 3.81)	71.5 (70.8 - 72.1)	56.4 (55.7 - 57.2)	43.2 (42.4 - 43.9)	18.1 (17.4 - 18.8)	5.8 (5.3 - 6.4)	1.5 (1.1 - 2.0)
Cardiovascular diseases	Subarachnoid hem	Both sexes	85-95	11266	1.36 (1.44 - 1.27)	54.7 (53.8 - 55.7)	32.2 (31.3 - 33.1)	17.2 (16.5 - 18.0)	2.7 (2.3 - 3.1)	0.2 (0.1 - 0.5)	0.0 (0.0 - 0.3)
Chronic respiratory	Asthma	Both sexes	All	400545	NR	95.7 (95.7 - 95.8)	91.3 (91.2 - 91.4)	87.5 (87.4 - 87.6)	78.7 (78.5 - 78.8)	70.8 (70.7 - 71.0)	64.1 (63.9 - 64.4)
Chronic respiratory	Asthma	Both sexes	16-24	47236	NR	99.9 (99.9 - 99.9)	99.8 (99.7 - 99.8)	99.7 (99.6 - 99.7)	99.3 (99.2 - 99.4)	99.0 (98.8 - 99.1)	98.5 (98.4 - 98.7)
Chronic respiratory	Asthma	Both sexes	25-34	47935	NR	99.8 (99.8 - 99.9)	99.6 (99.5 - 99.7)	99.4 (99.3 - 99.4)	98.8 (98.7 - 98.9)	98.1 (97.9 - 98.2)	97.1 (96.8 - 97.3)
Chronic respiratory	Asthma	Both sexes	35-44	53295	NR	99.7 (99.6 - 99.7)	99.3 (99.2 - 99.4)	98.9 (98.8 - 99.0)	97.6 (97.5 - 97.7)	95.7 (95.5 - 95.9)	93.2 (92.9 - 93.6)
Chronic respiratory	Asthma	Both sexes	45-54	57570	NR	99.2 (99.1 - 99.3)	98.0 (97.9 - 98.2)	97.0 (96.8 - 97.1)	94.1 (93.9 - 94.3)	89.6 (89.2 - 89.9)	83.4 (82.9 - 83.9)
Chronic respiratory	Asthma	Both sexes	55-64	61654	NR	97.7 (97.6 - 97.8)	95.1 (94.9 - 95.2)	92.6 (92.4 - 92.9)	85.4 (85.1 - 85.8)	75.4 (74.9 - 75.8)	61.9 (61.2 - 62.6)
Chronic respiratory	Asthma	Both sexes	65-74	61040	14.4 (14.51 - 14.25)	95.2 (95.0 - 95.3)	89.5 (89.3 - 89.8)	84.0 (83.7 - 84.3)	67.5 (67.1 - 68.0)	47.2 (46.6 - 47.8)	25.5 (24.8 - 26.3)
Chronic respiratory	Asthma	Both sexes	75-84	49881	7.17 (7.25 - 7.09)	88.2 (87.9 - 88.5)	75.7 (75.3 - 76.1)	63.5 (63.0 - 63.9)	33.1 (32.6 - 33.6)	11.9 (11.5 - 12.4)	2.8 (2.5 - 3.2)
Chronic respiratory	Asthma	Both sexes	85-95	21026	2.83 (2.89 - 2.77)	72.7 (72.1 - 73.3)	48.0 (47.3 - 48.7)	29.7 (29.0 - 30.3)	6.1 (5.7 - 6.5)	0.6 (0.5 - 0.8)	0.1 (0.0 - 0.2)
Chronic respiratory	COPD	Both sexes	All	373459	7.04 (7.08 - 7.0)	83.7 (83.6 - 83.8)	70.2 (70.1 - 70.4)	59.3 (59.2 - 59.5)	39.0 (38.8 - 39.2)	25.8 (25.7 - 26.0)	17.5 (17.3 - 17.7)
Chronic respiratory	COPD	Both sexes	16-24	2056	NR	98.8 (98.2 - 99.2)	97.6 (96.8 - 98.2)	96.9 (96.0 - 97.6)	94.9 (93.7 - 95.8)	93.2 (91.8 - 94.4)	92.5 (90.8 - 93.8)
Chronic respiratory	COPD	Both sexes	25-34	3186	NR	98.9 (98.4 - 99.2)	97.6 (97.0 - 98.1)	96.7 (96.0 - 97.3)	94.8 (93.9 - 95.6)	93.0 (91.9 - 94.0)	90.4 (88.8 - 91.7)
Chronic respiratory	COPD	Both sexes	35-44	7430	NR	98.5 (98.2 - 98.8)	96.8 (96.4 - 97.2)	95.2 (94.7 - 95.7)	91.4 (90.7 - 92.1)	86.3 (85.3 - 87.2)	81.0 (79.7 - 82.2)
Chronic respiratory	COPD	Both sexes	45-54	23205	NR	96.5 (96.2 - 96.7)	92.7 (92.3 - 93.0)	89.4 (89.0 - 89.8)	80.2 (79.6 - 80.7)	69.6 (68.9 - 70.3)	57.3 (56.3 - 58.3)
Chronic respiratory	COPD	Both sexes	55-64	66027	14.21 (14.35 - 14.07)	92.8 (92.6 - 93.0)	85.9 (85.6 - 86.2)	79.6 (79.3 - 79.9)	63.7 (63.3 - 64.1)	47.4 (46.9 - 47.8)	32.1 (31.5 - 32.7)
Chronic respiratory	COPD	Both sexes	65-74	114205	8.36 (8.43 - 8.29)	87.4 (87.2 - 87.6)	76.2 (76.0 - 76.5)	66.2 (65.9 - 66.5)	42.3 (42.0 - 42.6)	22.4 (22.1 - 22.8)	9.3 (8.9 - 9.7)
Chronic respiratory	COPD	Both sexes	75-84	113699	4.3 (4.34 - 4.25)	78.7 (78.5 - 79.0)	60.4 (60.1 - 60.7)	44.7 (44.4 - 45.0)	17.0 (16.7 - 17.3)	4.7 (4.5 - 4.8)	0.9 (0.8 - 1.1)
Chronic respiratory	COPD	Both sexes	85-95	42319	1.81 (1.85 - 1.78)	62.5 (62.1 - 63.0)	35.5 (35.1 - 36.0)	19.3 (18.9 - 19.7)	3.0 (2.8 - 3.2)	0.2 (0.2 - 0.3)	0.0 (0.0 - 0.1)
Chronic respiratory	Interstitial lung disease	Both sexes	All	66040	9.28 (9.5 - 9.06)	83.2 (82.9 - 83.5)	70.3 (70.0 - 70.7)	61.7 (61.3 - 62.1)	48.6 (48.1 - 49.0)	40.9 (40.4 - 41.4)	35.1 (34.6 - 35.7)
Chronic respiratory	Interstitial lung disease	Both sexes	16-24	624	NR	97.7 (96.2 - 98.7)	97.1 (95.4 - 98.1)	96.4 (94.5 - 97.6)	95.7 (93.6 - 97.1)	94.0 (91.5 - 95.8)	92.0 (88.2 - 94.6)
Chronic respiratory	Interstitial lung disease	Both sexes	25-34	3380	NR	99.0 (98.6 - 99.3)	98.3 (97.8 - 98.7)	97.9 (97.3 - 98.3)	97.0 (96.3 - 97.6)	95.7 (94.8 - 96.4)	94.5 (93.4 - 95.4)
Chronic respiratory	Interstitial lung disease	Both sexes	35-44	5454	NR	98.9 (98.5 - 99.1)	97.9 (97.5 - 98.3)	97.2 (96.7 - 97.6)	95.3 (94.6 - 95.8)	92.8 (92.0 - 93.6)	89.9 (88.7 - 91.0)

Appendix Table A5: Unadjusted Median Survival and Survival Probabilities by Age, both sexes (Page 9 of 18)

Group	Diagnosis Category	Sex	Age	N	Median Survival (y)	1y (%)	3y (%)	5y (%)	10y (%)	15y (%)	20y (%)
Chronic respiratory	Interstitial lung disease	Both sexes	45-54	6889	NR	97.3 (96.9 - 97.6)	94.6 (94.0 - 95.1)	92.8 (92.1 - 93.4)	87.7 (86.8 - 88.5)	82.4 (81.3 - 83.5)	75.5 (73.9 - 77.1)
Chronic respiratory	Interstitial lung disease	Both sexes	55-64	10291	17.8 (18.36 - 17.29)	93.0 (92.5 - 93.5)	86.5 (85.8 - 87.2)	80.9 (80.1 - 81.7)	68.5 (67.5 - 69.5)	56.8 (55.6 - 58.1)	44.4 (42.9 - 46.0)
Chronic respiratory	Interstitial lung disease	Both sexes	65-74	15335	7.07 (7.3 - 6.89)	85.3 (84.7 - 85.8)	71.2 (70.4 - 71.9)	59.9 (59.1 - 60.8)	38.4 (37.5 - 39.4)	22.6 (21.6 - 23.6)	10.7 (9.6 - 11.8)
Chronic respiratory	Interstitial lung disease	Both sexes	75-84	17604	3.07 (3.16 - 3.0)	73.1 (72.5 - 73.8)	50.8 (50.0 - 51.5)	35.1 (34.3 - 35.9)	12.3 (11.7 - 13.0)	3.7 (3.2 - 4.2)	0.9 (0.6 - 1.3)
Chronic respiratory	Interstitial lung disease	Both sexes	85-95	6322	1.08 (1.13 - 1.02)	51.7 (50.4 - 52.9)	24.5 (23.4 - 25.6)	11.5 (10.6 - 12.4)	1.6 (1.2 - 2.1)	0.1 (0.0 - 0.4)	0.1 (0.0 - 0.4)
Chronic respiratory	Pneumoconiosis	Both sexes	All	7985	7.12 (7.39 - 6.82)	82.8 (82.0 - 83.6)	69.1 (68.1 - 70.2)	58.8 (57.7 - 59.9)	40.1 (38.9 - 41.2)	27.4 (26.3 - 28.5)	18.9 (17.8 - 20.0)
Chronic respiratory	Pneumoconiosis	Both sexes	16-24	39	NR	100.0 (100.0 - 100.0)	100.0 (100.0 - 100.0)	100.0 (100.0 - 100.0)	92.9 (74.6 - 98.2)	92.9 (74.6 - 98.2)	92.9 (74.6 - 98.2)
Chronic respiratory	Pneumoconiosis	Both sexes	25-34	49	NR	98.0 (86.4 - 99.7)	98.0 (86.4 - 99.7)	98.0 (86.4 - 99.7)	93.6 (81.3 - 97.9)	93.6 (81.3 - 97.9)	93.6 (81.3 - 97.9)
Chronic respiratory	Pneumoconiosis	Both sexes	35-44	94	NR	98.9 (92.5 - 99.8)	95.6 (88.6 - 98.3)	94.4 (87.1 - 97.6)	90.6 (82.1 - 95.2)	89.2 (80.3 - 94.3)	83.9 (71.4 - 91.2)
Chronic respiratory	Pneumoconiosis	Both sexes	45-54	229	NR	98.2 (95.4 - 99.3)	96.9 (93.6 - 98.5)	96.0 (92.4 - 97.9)	87.8 (82.5 - 91.6)	77.4 (70.6 - 82.9)	72.4 (64.7 - 78.6)
Chronic respiratory	Pneumoconiosis	Both sexes	55-64	1196	20.33 (21.09 - 19.16)	95.6 (94.3 - 96.7)	91.1 (89.3 - 92.5)	86.9 (84.8 - 88.7)	75.5 (72.9 - 77.9)	63.2 (60.2 - 66.0)	51.6 (48.1 - 54.9)
Chronic respiratory	Pneumoconiosis	Both sexes	65-74	2279	10.87 (11.41 - 10.2)	90.0 (88.6 - 91.1)	80.2 (78.5 - 81.8)	72.4 (70.5 - 74.2)	53.1 (50.9 - 55.2)	33.7 (31.4 - 35.9)	15.5 (13.3 - 17.9)
Chronic respiratory	Pneumoconiosis	Both sexes	75-84	3053	4.43 (4.67 - 4.21)	78.8 (77.3 - 80.2)	60.9 (59.2 - 62.7)	45.9 (44.1 - 47.7)	19.6 (18.0 - 21.1)	6.0 (4.9 - 7.1)	0.5 (0.1 - 1.4)
Chronic respiratory	Pneumoconiosis	Both sexes	85-95	1027	1.58 (1.83 - 1.34)	58.1 (54.9 - 61.0)	31.7 (28.8 - 34.7)	16.4 (14.0 - 18.9)	2.3 (1.4 - 3.6)	0.2 (0.0 - 0.8)	0.0 (0.0 - 0.0)
Chronic respiratory	Sleep Apnea	Both sexes	All	293509	NR	98.6 (98.6 - 98.7)	96.4 (96.3 - 96.5)	93.9 (93.8 - 94.0)	87.0 (86.9 - 87.2)	78.8 (78.6 - 79.0)	69.3 (69.0 - 69.6)
Chronic respiratory	Sleep Apnea	Both sexes	16-24	4635	NR	99.8 (99.6 - 99.9)	99.3 (99.0 - 99.5)	98.9 (98.5 - 99.2)	97.9 (97.4 - 98.4)	96.9 (96.1 - 97.5)	96.1 (95.0 - 97.0)
Chronic respiratory	Sleep Apnea	Both sexes	25-34	18321	NR	99.8 (99.7 - 99.9)	99.5 (99.3 - 99.6)	99.1 (98.9 - 99.2)	98.2 (98.0 - 98.5)	97.0 (96.6 - 97.3)	95.1 (94.4 - 95.7)
Chronic respiratory	Sleep Apnea	Both sexes	35-44	38461	NR	99.8 (99.7 - 99.8)	99.4 (99.3 - 99.4)	98.9 (98.8 - 99.0)	97.4 (97.2 - 97.6)	95.4 (95.1 - 95.6)	92.2 (91.7 - 92.8)
Chronic respiratory	Sleep Apnea	Both sexes	45-54	68756	NR	99.6 (99.5 - 99.6)	98.8 (98.8 - 98.9)	97.9 (97.8 - 98.1)	95.2 (95.0 - 95.4)	91.2 (90.9 - 91.5)	85.1 (84.6 - 85.6)
Chronic respiratory	Sleep Apnea	Both sexes	55-64	84242	NR	99.1 (99.0 - 99.2)	97.6 (97.4 - 97.7)	95.7 (95.5 - 95.8)	89.8 (89.5 - 90.0)	81.3 (81.0 - 81.7)	69.0 (68.3 - 69.6)
Chronic respiratory	Sleep Apnea	Both sexes	65-74	56873	16.42 (16.61 - 16.26)	97.7 (97.5 - 97.8)	93.6 (93.4 - 93.8)	88.9 (88.6 - 89.2)	75.3 (74.9 - 75.8)	56.5 (55.8 - 57.2)	33.2 (32.1 - 34.4)
Chronic respiratory	Sleep Apnea	Both sexes	75-84	20307	9.13 (9.3 - 8.98)	93.8 (93.4 - 94.1)	84.2 (83.7 - 84.7)	73.3 (72.6 - 74.0)	45.0 (44.1 - 46.0)	18.8 (17.7 - 20.0)	5.5 (4.4 - 6.8)
Chronic respiratory	Sleep Apnea	Both sexes	85-95	1890	4.09 (4.39 - 3.81)	82.3 (80.4 - 84.0)	61.8 (59.3 - 64.1)	42.4 (39.7 - 45.0)	12.2 (10.0 - 14.6)	1.3 (0.4 - 3.3)	0.0 (0.0 - 0.0)
Diabetes & CKD	Acute glomerulonephritis	Both sexes	All	4245	NR	94.0 (93.2 - 94.6)	89.8 (88.8 - 90.7)	85.6 (84.5 - 86.7)	76.3 (74.8 - 77.6)	67.2 (65.6 - 68.9)	58.0 (55.6 - 60.3)
Diabetes & CKD	Acute glomerulonephritis	Both sexes	16-24	529	NR	100.0 (100.0 - 100.0)	100.0 (100.0 - 100.0)	99.4 (98.1 - 99.8)	98.6 (97.0 - 99.4)	98.3 (96.3 - 99.2)	97.0 (92.6 - 98.8)
Diabetes & CKD	Acute glomerulonephritis	Both sexes	25-34	449	NR	99.3 (97.9 - 99.8)	98.9 (97.3 - 99.5)	97.9 (96.0 - 98.9)	96.6 (94.3 - 98.0)	96.2 (93.7 - 97.7)	95.4 (92.1 - 97.3)
Diabetes & CKD	Acute glomerulonephritis	Both sexes	35-44	507	NR	99.6 (98.4 - 99.9)	99.2 (97.9 - 99.7)	98.3 (96.7 - 99.2)	96.0 (93.8 - 97.5)	94.1 (91.3 - 96.1)	85.8 (79.1 - 90.4)
Diabetes & CKD	Acute glomerulonephritis	Both sexes	45-54	590	NR	97.4 (95.8 - 98.4)	95.8 (93.8 - 97.2)	95.1 (93.0 - 96.6)	92.4 (89.8 - 94.4)	88.4 (85.1 - 90.9)	76.8 (69.8 - 82.4)
Diabetes & CKD	Acute glomerulonephritis	Both sexes	55-64	718	21.91	96.4 (94.8 - 97.6)	93.8 (91.7 - 95.3)	90.1 (87.6 - 92.2)	80.2 (76.8 - 83.1)	70.5 (66.4 - 74.2)	52.1 (44.7 - 59.0)
Diabetes & CKD	Acute glomerulonephritis	Both sexes	65-74	757	12.2 (13.05 - 11.2)	92.2 (90.0 - 93.9)	85.8 (83.0 - 88.2)	78.2 (74.9 - 81.1)	59.0 (54.9 - 62.9)	37.3 (32.9 - 41.7)	15.5 (10.1 - 22.0)
Diabetes & CKD	Acute glomerulonephritis	Both sexes	75-84	572	5.56 (6.38 - 4.92)	80.5 (76.9 - 83.6)	66.4 (62.1 - 70.3)	53.6 (49.1 - 58.0)	27.5 (23.3 - 31.8)	8.9 (6.1 - 12.3)	3.3 (1.3 - 6.8)
Diabetes & CKD	Acute glomerulonephritis	Both sexes	85-95	123	2.33 (3.24 - 1.42)	63.2 (53.7 - 71.3)	44.0 (34.5 - 53.0)	29.1 (20.5 - 38.3)	13.4 (7.1 - 21.8)	3.0 (0.6 - 9.1)	1.5 (0.1 - 7.0)
Diabetes & CKD	Chronic kidney disease	Both sexes	All	456498	3.79 (3.82 - 3.76)	69.7 (69.6 - 69.8)	54.5 (54.3 - 54.6)	44.0 (43.9 - 44.2)	28.9 (28.7 - 29.0)	21.3 (21.1 - 21.5)	16.8 (16.7 - 17.0)
Diabetes & CKD	Chronic kidney disease	Both sexes	16-24	7365	NR	97.9 (97.5 - 98.2)	96.8 (96.4 - 97.2)	96.1 (95.6 - 96.6)	94.7 (94.1 - 95.3)	93.4 (92.7 - 94.1)	92.0 (91.1 - 92.9)
Diabetes & CKD	Chronic kidney disease	Both sexes	25-34	10906	NR	97.2 (96.9 - 97.5)	95.7 (95.3 - 96.1)	94.7 (94.3 - 95.2)	92.4 (91.8 - 92.9)	89.8 (89.1 - 90.5)	87.2 (86.2 - 88.1)
Diabetes & CKD	Chronic kidney disease	Both sexes	35-44	15185	NR	95.2 (94.8 - 95.5)	93.0 (92.6 - 93.4)	91.5 (91.0 - 91.9)	87.5 (87.0 - 88.1)	82.7 (81.9 - 83.4)	76.8 (75.8 - 77.8)
Diabetes & CKD	Chronic kidney disease	Both sexes	45-54	24995	22.37 (22.84 - 21.72)	90.4 (90.0 - 90.8)	86.2 (85.7 - 86.6)	82.7 (82.2 - 83.2)	73.9 (73.2 - 74.5)	64.8 (64.1 - 65.6)	54.6 (53.6 - 55.6)
Diabetes & CKD	Chronic kidney disease	Both sexes	55-64	47484	11.54 (11.75 - 11.34)	84.4 (84.1 - 84.7)	76.8 (76.4 - 77.1)	70.1 (69.6 - 70.5)	54.5 (54.0 - 55.0)	40.4 (39.8 - 41.0)	27.8 (27.1 - 28.6)
Diabetes & CKD	Chronic kidney disease	Both sexes	65-74	92630	6.01 (6.09 - 5.94)	77.3 (77.0 - 77.6)	65.0 (64.7 - 65.3)	54.8 (54.4 - 55.1)	32.9 (32.5 - 33.3)	16.5 (16.1 - 16.9)	6.8 (6.4 - 7.3)
Diabetes & CKD	Chronic kidney disease	Both sexes	75-84	143613	2.78 (2.81 - 2.74)	66.9 (66.7 - 67.2)	48.0 (47.8 - 48.3)	33.5 (33.2 - 33.8)	11.0 (10.8 - 11.3)	2.5 (2.4 - 2.7)	0.3 (0.3 - 0.5)
Diabetes & CKD	Chronic kidney disease	Both sexes	85-95	107495	0.95 (0.97 - 0.94)	49.2 (48.9 - 49.5)	25.4 (25.1 - 25.7)	12.0 (11.8 - 12.2)	1.4 (1.3 - 1.5)	0.1 (0.1 - 0.2)	0.0 (0.0 - 0.1)
Diabetes & CKD	Diabetes	Both sexes	All	749652	13.11 (13.15 - 13.06)	91.3 (91.2 - 91.4)	83.4 (83.3 - 83.5)	76.2 (76.1 - 76.3)	59.4 (59.3 - 59.6)	44.7 (44.5 - 44.8)	33.4 (33.3 - 33.6)
Diabetes & CKD	Diabetes	Both sexes	16-24	11316	NR	99.6 (99.4 - 99.7)	99.2 (99.0 - 99.4)	98.9 (98.6 - 99.0)	98.0 (97.7 - 98.2)	97.3 (96.9 - 97.6)	96.3 (95.7 - 96.7)
Diabetes & CKD	Diabetes	Both sexes	25-34	20068	NR	99.4 (99.3 - 99.5)	98.9 (98.8 - 99.0)	98.3 (98.1 - 98.5)	97.0 (96.7 - 97.2)	95.5 (95.1 - 95.8)	93.4 (92.9 - 93.9)
Diabetes & CKD	Diabetes	Both sexes	35-44	39245	NR	99.0 (98.9 - 99.1)	98.0 (97.8 - 98.1)	97.0 (96.8 - 97.1)	94.0 (93.7 - 94.3)	89.9 (89.5 - 90.2)	84.4 (83.9 - 85.0)
Diabetes & CKD	Diabetes	Both sexes	45-54	84621	NR	97.9 (97.8 - 98.0)	95.9 (95.8 - 96.1)	94.0 (93.9 - 94.2)	88.3 (88.0 - 88.5)	80.0 (79.7 - 80.4)	69.2 (68.7 - 69.7)
Diabetes & CKD	Diabetes	Both sexes	55-64	160008	19.31 (19.41 - 19.19)	96.1 (96.0 - 96.2)	92.5 (92.4 - 92.7)	89.0 (88.9 - 89.2)	78.6 (78.4 - 78.8)	64.6 (64.3 - 64.9)	47.3 (46.9 - 47.8)
Diabetes & CKD	Diabetes	Both sexes	65-74	205571	12.29 (12.35 - 12.23)	92.8 (92.7 - 92.9)	86.3 (86.1 - 86.4)	79.7 (79.5 - 79.9)	60.4 (60.2 - 60.7)	37.0 (36.7 - 37.3)	16.5 (16.1 - 16.8)
Diabetes & CKD	Diabetes	Both sexes	75-84	168166	6.4 (6.45 - 6.36)	86.1 (85.9 - 86.3)	72.5 (72.3 - 72.7)	59.0 (58.8 - 59.3)	28.3 (28.0 - 28.5)	8.4 (8.2 - 8.6)	1.5 (1.4 - 1.6)
Diabetes & CKD	Diabetes	Both sexes	85-95	58764	2.58 (2.61 - 2.54)	70.2 (69.8 - 70.6)	45.2 (44.8 - 45.7)	26.6 (26.2 - 27.0)	4.5 (4.3 - 4.8)	0.4 (0.3 - 0.4)	0.1 (0.0 - 0.1)

Appendix Table A5: Unadjusted Median Survival and Survival Probabilities by Age, both sexes (Page 10 of 18)

Group	Diagnosis Category	Sex	Age	N	Median Survival (y)	1y (%)	3y (%)	5y (%)	10y (%)	15y (%)	20y (%)
Diabetes & CKD	Diabetes type 1	Both sexes	All	180139	12.43 (12.53 - 12.33)	89.9 (89.8 - 90.1)	80.6 (80.4 - 80.8)	72.7 (72.5 - 72.9)	56.5 (56.3 - 56.8)	44.0 (43.8 - 44.3)	35.3 (35.0 - 35.6)
Diabetes & CKD	Diabetes type 1	Both sexes	16-24	8641	NR	99.8 (99.6 - 99.9)	99.5 (99.4 - 99.7)	99.2 (99.0 - 99.4)	98.5 (98.2 - 98.7)	97.9 (97.5 - 98.2)	96.8 (96.3 - 97.3)
Diabetes & CKD	Diabetes type 1	Both sexes	25-34	11512	NR	99.7 (99.6 - 99.8)	99.2 (99.1 - 99.4)	98.7 (98.4 - 98.9)	97.6 (97.3 - 97.9)	96.1 (95.7 - 96.5)	94.1 (93.6 - 94.7)
Diabetes & CKD	Diabetes type 1	Both sexes	35-44	14364	NR	99.0 (98.8 - 99.1)	98.0 (97.7 - 98.2)	96.8 (96.5 - 97.1)	93.4 (93.0 - 93.8)	89.2 (88.7 - 89.8)	84.4 (83.6 - 85.1)
Diabetes & CKD	Diabetes type 1	Both sexes	45-54	21430	NR	97.6 (97.4 - 97.8)	94.7 (94.4 - 95.0)	92.0 (91.7 - 92.4)	84.5 (84.0 - 85.0)	75.2 (74.6 - 75.9)	64.5 (63.7 - 65.3)
Diabetes & CKD	Diabetes type 1	Both sexes	55-64	34366	16.4 (16.57 - 16.22)	94.7 (94.4 - 94.9)	89.5 (89.2 - 89.8)	84.6 (84.2 - 84.9)	70.8 (70.3 - 71.3)	54.8 (54.2 - 55.3)	38.0 (37.3 - 38.7)
Diabetes & CKD	Diabetes type 1	Both sexes	65-74	40790	9.63 (9.75 - 9.52)	89.9 (89.6 - 90.2)	80.2 (79.8 - 80.6)	70.9 (70.5 - 71.4)	48.3 (47.8 - 48.8)	26.5 (26.0 - 27.0)	10.7 (10.2 - 11.1)
Diabetes & CKD	Diabetes type 1	Both sexes	75-84	36243	4.66 (4.72 - 4.58)	81.1 (80.7 - 81.5)	63.0 (62.5 - 63.5)	47.5 (47.0 - 48.0)	18.7 (18.3 - 19.2)	4.8 (4.5 - 5.1)	0.8 (0.6 - 0.9)
Diabetes & CKD	Diabetes type 1	Both sexes	85-95	12439	1.95 (2.0 - 1.88)	64.6 (63.7 - 65.4)	36.8 (35.9 - 37.6)	19.2 (18.5 - 20.0)	2.6 (2.3 - 3.0)	0.3 (0.2 - 0.4)	0.1 (0.0 - 0.2)
Diabetes & CKD	Diabetes type 2	Both sexes	All	697339	11.83 (11.88 - 11.78)	90.8 (90.7 - 90.9)	82.2 (82.1 - 82.2)	74.2 (74.1 - 74.3)	56.1 (56.0 - 56.2)	40.3 (40.1 - 40.5)	27.9 (27.7 - 28.1)
Diabetes & CKD	Diabetes type 2	Both sexes	16-24	3857	NR	99.3 (99.0 - 99.5)	98.7 (98.3 - 99.0)	98.0 (97.5 - 98.4)	96.2 (95.5 - 96.9)	94.3 (93.3 - 95.2)	92.7 (91.1 - 94.0)
Diabetes & CKD	Diabetes type 2	Both sexes	25-34	11319	NR	99.3 (99.1 - 99.4)	98.4 (98.2 - 98.6)	97.4 (97.0 - 97.7)	95.4 (94.9 - 95.8)	93.0 (92.3 - 93.6)	89.1 (88.1 - 90.1)
Diabetes & CKD	Diabetes type 2	Both sexes	35-44	30563	NR	98.9 (98.8 - 99.0)	97.8 (97.6 - 97.9)	96.5 (96.2 - 96.7)	92.8 (92.4 - 93.1)	87.3 (86.8 - 87.8)	79.9 (79.1 - 80.6)
Diabetes & CKD	Diabetes type 2	Both sexes	45-54	75282	NR	97.8 (97.7 - 97.9)	95.7 (95.5 - 95.8)	93.6 (93.4 - 93.7)	87.0 (86.7 - 87.3)	77.8 (77.4 - 78.2)	65.5 (64.9 - 66.0)
Diabetes & CKD	Diabetes type 2	Both sexes	55-64	150059	18.63 (18.75 - 18.51)	96.2 (96.1 - 96.3)	92.3 (92.2 - 92.4)	88.5 (88.3 - 88.7)	77.3 (77.0 - 77.5)	62.5 (62.2 - 62.8)	44.9 (44.5 - 45.4)
Diabetes & CKD	Diabetes type 2	Both sexes	65-74	199283	11.94 (12.0 - 11.88)	92.8 (92.7 - 93.0)	85.9 (85.8 - 86.1)	79.0 (78.8 - 79.2)	58.9 (58.7 - 59.2)	35.2 (34.9 - 35.5)	15.2 (14.9 - 15.6)
Diabetes & CKD	Diabetes type 2	Both sexes	75-84	166409	6.2 (6.25 - 6.16)	85.9 (85.7 - 86.1)	71.7 (71.5 - 71.9)	57.9 (57.7 - 58.2)	27.0 (26.7 - 27.3)	7.8 (7.6 - 8.0)	1.4 (1.2 - 1.5)
Diabetes & CKD	Diabetes type 2	Both sexes	85-95	58764	2.54 (2.57 - 2.5)	69.9 (69.6 - 70.3)	44.8 (44.4 - 45.2)	26.0 (25.6 - 26.3)	4.3 (4.1 - 4.5)	0.3 (0.2 - 0.4)	0.1 (0.0 - 0.1)
Digestive diseases	Cirrhosis	Both sexes	All	158425	20.11 (20.33 - 19.81)	88.6 (88.4 - 88.8)	81.4 (81.3 - 81.6)	76.2 (76.0 - 76.4)	65.7 (65.5 - 66.0)	57.3 (57.0 - 57.6)	50.1 (49.8 - 50.5)
Digestive diseases	Cirrhosis	Both sexes	16-24	9485	NR	98.9 (98.7 - 99.1)	97.3 (96.9 - 97.6)	96.1 (95.7 - 96.5)	93.5 (93.0 - 94.0)	90.8 (90.1 - 91.5)	88.1 (87.2 - 89.0)
Digestive diseases	Cirrhosis	Both sexes	25-34	19962	NR	98.7 (98.6 - 98.9)	97.2 (96.9 - 97.4)	95.8 (95.5 - 96.1)	92.5 (92.1 - 92.9)	89.8 (89.2 - 90.3)	86.2 (85.5 - 86.9)
Digestive diseases	Cirrhosis	Both sexes	35-44	22978	NR	97.9 (97.7 - 98.0)	95.9 (95.7 - 96.2)	94.0 (93.7 - 94.3)	89.2 (88.7 - 89.6)	83.4 (82.8 - 84.0)	77.8 (77.0 - 78.5)
Digestive diseases	Cirrhosis	Both sexes	45-54	28658	NR	93.8 (93.5 - 94.1)	89.0 (88.6 - 89.3)	85.0 (84.6 - 85.4)	75.1 (74.6 - 75.7)	65.5 (64.9 - 66.2)	55.9 (55.0 - 56.7)
Digestive diseases	Cirrhosis	Both sexes	55-64	31861	13.59 (13.92 - 13.31)	87.9 (87.5 - 88.3)	79.7 (79.2 - 80.1)	73.1 (72.6 - 73.6)	59.0 (58.4 - 59.6)	46.6 (45.8 - 47.3)	35.0 (34.2 - 35.9)
Digestive diseases	Cirrhosis	Both sexes	65-74	27010	7.19 (7.38 - 7.01)	80.1 (79.6 - 80.6)	67.8 (67.3 - 68.4)	58.7 (58.0 - 59.3)	40.4 (39.7 - 41.2)	25.4 (24.6 - 26.2)	13.0 (12.1 - 13.9)
Digestive diseases	Cirrhosis	Both sexes	75-84	14701	3.26 (3.4 - 3.13)	69.6 (68.8 - 70.3)	52.0 (51.1 - 52.8)	39.9 (39.0 - 40.7)	18.7 (17.9 - 19.5)	6.5 (5.8 - 7.2)	1.5 (1.1 - 2.0)
Digestive diseases	Cirrhosis	Both sexes	85-95	3699	1.15 (1.27 - 1.02)	51.9 (50.2 - 53.5)	30.0 (28.5 - 31.6)	17.6 (16.3 - 19.0)	4.2 (3.4 - 5.0)	0.4 (0.2 - 0.9)	0.0 (0.0 - 0.0)
Digestive diseases	Crohn's disease	Both sexes	All	53613	NR	97.5 (97.4 - 97.7)	94.7 (94.5 - 94.9)	92.2 (91.9 - 92.4)	86.0 (85.7 - 86.3)	79.8 (79.4 - 80.2)	73.4 (72.8 - 73.9)
Digestive diseases	Crohn's disease	Both sexes	16-24	8944	NR	100.0 (99.9 - 100.0)	99.8 (99.7 - 99.9)	99.6 (99.4 - 99.7)	99.1 (98.9 - 99.3)	98.5 (98.1 - 98.8)	97.7 (97.1 - 98.2)
Digestive diseases	Crohn's disease	Both sexes	25-34	9862	NR	99.9 (99.8 - 99.9)	99.6 (99.5 - 99.7)	99.3 (99.1 - 99.5)	98.6 (98.3 - 98.8)	97.7 (97.3 - 98.1)	96.2 (95.6 - 96.8)
Digestive diseases	Crohn's disease	Both sexes	35-44	8261	NR	99.7 (99.6 - 99.8)	99.2 (99.0 - 99.4)	98.6 (98.4 - 98.9)	97.0 (96.6 - 97.4)	95.2 (94.6 - 95.7)	93.2 (92.4 - 94.0)
Digestive diseases	Crohn's disease	Both sexes	45-54	8147	NR	99.2 (98.9 - 99.3)	98.2 (97.9 - 98.5)	97.0 (96.6 - 97.3)	93.4 (92.8 - 94.0)	88.9 (88.1 - 89.7)	82.3 (81.1 - 83.4)
Digestive diseases	Crohn's disease	Both sexes	55-64	7885	NR	97.9 (97.5 - 98.2)	94.7 (94.2 - 95.2)	92.0 (91.3 - 92.6)	84.7 (83.8 - 85.6)	75.3 (74.1 - 76.5)	62.2 (60.5 - 63.8)
Digestive diseases	Crohn's disease	Both sexes	65-74	5978	14.0 (14.43 - 13.56)	94.7 (94.1 - 95.2)	88.7 (87.9 - 89.5)	82.7 (81.7 - 83.7)	66.2 (64.8 - 67.6)	46.1 (44.3 - 47.9)	25.8 (23.8 - 27.9)
Digestive diseases	Crohn's disease	Both sexes	75-84	3534	6.65 (7.0 - 6.34)	87.2 (86.0 - 88.3)	73.2 (71.7 - 74.7)	60.5 (58.7 - 62.2)	31.3 (29.5 - 33.1)	10.9 (9.5 - 12.5)	3.0 (2.0 - 4.3)
Digestive diseases	Crohn's disease	Both sexes	85-95	968	2.81 (3.2 - 2.52)	72.8 (69.9 - 75.5)	48.6 (45.3 - 51.8)	31.3 (28.2 - 34.4)	7.2 (5.4 - 9.3)	0.4 (0.1 - 1.8)	0.4 (0.1 - 1.8)
Digestive diseases	Diverticular disease	Both sexes	All	376321	16.97 (17.09 - 16.88)	94.8 (94.7 - 94.9)	88.6 (88.5 - 88.7)	82.5 (82.4 - 82.7)	68.2 (68.0 - 68.3)	54.8 (54.6 - 55.1)	43.2 (42.9 - 43.6)
Digestive diseases	Diverticular disease	Both sexes	16-24	658	NR	99.8 (98.9 - 100.0)	99.5 (98.5 - 99.8)	98.8 (97.4 - 99.4)	97.9 (96.2 - 98.8)	97.2 (95.2 - 98.4)	96.6 (94.0 - 98.1)
Digestive diseases	Diverticular disease	Both sexes	25-34	5289	NR	99.8 (99.6 - 99.9)	99.6 (99.4 - 99.8)	99.2 (98.9 - 99.4)	98.5 (98.1 - 98.8)	97.5 (96.9 - 98.1)	96.7 (95.7 - 97.4)
Digestive diseases	Diverticular disease	Both sexes	35-44	19864	NR	99.8 (99.7 - 99.8)	99.4 (99.3 - 99.5)	99.0 (98.9 - 99.2)	97.7 (97.5 - 98.0)	96.1 (95.7 - 96.5)	93.7 (93.1 - 94.3)
Digestive diseases	Diverticular disease	Both sexes	45-54	44243	NR	99.3 (99.2 - 99.4)	98.4 (98.3 - 98.5)	97.6 (97.5 - 97.8)	95.2 (95.0 - 95.4)	91.8 (91.4 - 92.1)	86.3 (85.7 - 87.0)
Digestive diseases	Diverticular disease	Both sexes	55-64	81167	NR	98.4 (98.3 - 98.5)	96.4 (96.3 - 96.5)	94.5 (94.3 - 94.7)	88.7 (88.5 - 89.0)	80.4 (80.0 - 80.8)	68.5 (67.8 - 69.1)
Digestive diseases	Diverticular disease	Both sexes	65-74	105848	15.87 (15.98 - 15.76)	96.2 (96.1 - 96.3)	91.5 (91.3 - 91.6)	86.7 (86.5 - 87.0)	72.7 (72.4 - 73.1)	53.8 (53.3 - 54.3)	31.7 (31.0 - 32.5)
Digestive diseases	Diverticular disease	Both sexes	75-84	90268	8.12 (8.19 - 8.06)	91.3 (91.1 - 91.5)	80.1 (79.8 - 80.4)	68.4 (68.1 - 68.7)	39.3 (38.9 - 39.7)	15.5 (15.1 - 15.9)	3.8 (3.5 - 4.2)
Digestive diseases	Diverticular disease	Both sexes	85-95	28334	3.73 (3.81 - 3.67)	79.7 (79.2 - 80.2)	57.5 (56.9 - 58.1)	38.6 (37.9 - 39.2)	9.3 (8.9 - 9.8)	1.2 (1.0 - 1.5)	0.1 (0.0 - 0.3)
Digestive diseases	Gallbladder & biliary	Both sexes	All	499559	NR	93.6 (93.5 - 93.7)	88.6 (88.5 - 88.7)	84.7 (84.6 - 84.8)	76.0 (75.9 - 76.2)	68.2 (68.0 - 68.3)	60.8 (60.6 - 61.0)
Digestive diseases	Gallbladder & biliary	Both sexes	16-24	20662	NR	99.8 (99.8 - 99.9)	99.6 (99.5 - 99.7)	99.4 (99.2 - 99.5)	98.9 (98.7 - 99.0)	98.3 (98.1 - 98.5)	97.6 (97.3 - 97.9)
Digestive diseases	Gallbladder & biliary	Both sexes	25-34	57488	NR	99.8 (99.8 - 99.8)	99.6 (99.5 - 99.6)	99.4 (99.3 - 99.4)	98.8 (98.7 - 98.9)	98.1 (98.0 - 98.3)	97.2 (97.0 - 97.5)
Digestive diseases	Gallbladder & biliary	Both sexes	35-44	71217	NR	99.5 (99.4 - 99.5)	99.1 (99.0 - 99.2)	98.8 (98.7 - 98.9)	97.8 (97.6 - 97.9)	96.5 (96.3 - 96.6)	94.4 (94.1 - 94.6)
Digestive diseases	Gallbladder & biliary	Both sexes	45-54	78794	NR	98.6 (98.5 - 98.7)	97.5 (97.4 - 97.6)	96.7 (96.5 - 96.8)	94.4 (94.2 - 94.6)	91.3 (91.1 - 91.6)	86.6 (86.2 - 87.0)

Appendix Table A5: Unadjusted Median Survival and Survival Probabilities by Age, both sexes (Page 11 of 18)

Group	Diagnosis Category	Sex	Age	N	Median Survival (y)	1y (%)	3y (%)	5y (%)	10y (%)	15y (%)	20y (%)
Digestive diseases	Gallbladder & biliary	Both sexes	55-64	85612	NR	96.4 (96.3 - 96.6)	93.8 (93.7 - 94.0)	91.9 (91.7 - 92.1)	86.4 (86.1 - 86.6)	79.0 (78.6 - 79.3)	69.0 (68.5 - 69.5)
Digestive diseases	Gallbladder & biliary	Both sexes	65-74	83621	15.23 (15.37 - 15.11)	92.0 (91.8 - 92.2)	86.1 (85.9 - 86.4)	81.3 (81.0 - 81.6)	68.0 (67.6 - 68.4)	50.9 (50.4 - 51.4)	31.4 (30.7 - 32.0)
Digestive diseases	Gallbladder & biliary	Both sexes	75-84	69264	6.89 (6.97 - 6.82)	84.6 (84.3 - 84.9)	71.7 (71.4 - 72.1)	60.4 (60.0 - 60.8)	33.9 (33.5 - 34.3)	13.5 (13.1 - 13.8)	3.1 (2.8 - 3.4)
Digestive diseases	Gallbladder & biliary	Both sexes	85-95	31501	2.76 (2.81 - 2.7)	70.5 (70.0 - 71.0)	47.5 (46.9 - 48.1)	29.9 (29.3 - 30.4)	6.8 (6.5 - 7.2)	0.8 (0.6 - 1.0)	0.1 (0.0 - 0.2)
Digestive diseases	Inflammatory bowel	Both sexes	All	235177	NR	94.3 (94.2 - 94.4)	89.1 (88.9 - 89.2)	84.8 (84.6 - 84.9)	76.0 (75.8 - 76.2)	68.8 (68.5 - 69.0)	62.5 (62.2 - 62.8)
Digestive diseases	Inflammatory bowel	Both sexes	16-24	27794	NR	99.9 (99.8 - 99.9)	99.7 (99.6 - 99.7)	99.5 (99.4 - 99.6)	99.1 (98.9 - 99.2)	98.5 (98.3 - 98.6)	97.9 (97.6 - 98.1)
Digestive diseases	Inflammatory bowel	Both sexes	25-34	34549	NR	99.8 (99.7 - 99.8)	99.5 (99.4 - 99.6)	99.3 (99.2 - 99.4)	98.6 (98.5 - 98.8)	97.8 (97.6 - 98.0)	96.8 (96.6 - 97.1)
Digestive diseases	Inflammatory bowel	Both sexes	35-44	29384	NR	99.5 (99.4 - 99.6)	98.8 (98.7 - 99.0)	98.2 (98.1 - 98.4)	96.8 (96.6 - 97.0)	94.9 (94.6 - 95.2)	92.5 (92.1 - 92.9)
Digestive diseases	Inflammatory bowel	Both sexes	45-54	30412	NR	98.6 (98.4 - 98.7)	97.1 (96.9 - 97.2)	95.8 (95.6 - 96.0)	92.4 (92.0 - 92.7)	88.5 (88.1 - 88.9)	82.8 (82.2 - 83.4)
Digestive diseases	Inflammatory bowel	Both sexes	55-64	34807	NR	96.4 (96.2 - 96.6)	92.7 (92.4 - 93.0)	89.8 (89.5 - 90.2)	82.2 (81.8 - 82.6)	72.8 (72.2 - 73.3)	60.8 (60.1 - 61.6)
Digestive diseases	Inflammatory bowel	Both sexes	65-74	35136	13.18 (13.36 - 13.0)	92.2 (91.9 - 92.5)	84.9 (84.5 - 85.3)	78.6 (78.2 - 79.1)	61.8 (61.2 - 62.4)	43.2 (42.4 - 43.9)	24.1 (23.3 - 25.0)
Digestive diseases	Inflammatory bowel	Both sexes	75-84	30142	5.94 (6.06 - 5.84)	84.2 (83.7 - 84.6)	69.1 (68.5 - 69.6)	55.6 (55.0 - 56.2)	27.9 (27.3 - 28.5)	9.6 (9.1 - 10.1)	2.4 (2.1 - 2.7)
Digestive diseases	Inflammatory bowel	Both sexes	85-95	12474	2.56 (2.64 - 2.49)	70.5 (69.6 - 71.3)	45.1 (44.2 - 46.0)	27.0 (26.2 - 27.9)	5.5 (5.1 - 6.0)	0.6 (0.4 - 0.8)	0.0 (0.0 - 0.0)
Digestive diseases	Pancreatitis	Both sexes	All	110236	15.54 (15.77 - 15.35)	89.3 (89.2 - 89.5)	81.8 (81.5 - 82.0)	75.7 (75.4 - 76.0)	62.5 (62.1 - 62.8)	51.1 (50.7 - 51.5)	41.8 (41.2 - 42.3)
Digestive diseases	Pancreatitis	Both sexes	16-24	4009	NR	99.3 (99.0 - 99.5)	98.9 (98.5 - 99.2)	98.7 (98.2 - 99.0)	97.8 (97.2 - 98.2)	97.0 (96.3 - 97.6)	96.1 (95.1 - 97.0)
Digestive diseases	Pancreatitis	Both sexes	25-34	7610	NR	99.2 (99.0 - 99.4)	98.4 (98.1 - 98.7)	97.7 (97.4 - 98.1)	96.2 (95.7 - 96.7)	94.7 (93.9 - 95.3)	93.0 (92.0 - 94.0)
Digestive diseases	Pancreatitis	Both sexes	35-44	10056	NR	98.1 (97.8 - 98.3)	96.2 (95.8 - 96.6)	94.9 (94.4 - 95.3)	91.5 (90.9 - 92.1)	87.0 (86.1 - 87.9)	81.8 (80.4 - 83.0)
Digestive diseases	Pancreatitis	Both sexes	45-54	14607	NR	95.9 (95.6 - 96.2)	92.7 (92.2 - 93.1)	90.3 (89.8 - 90.8)	83.5 (82.8 - 84.2)	76.0 (75.1 - 76.9)	67.3 (65.9 - 68.6)
Digestive diseases	Pancreatitis	Both sexes	55-64	19685	19.87 (20.3 - 19.33)	93.1 (92.8 - 93.5)	87.6 (87.1 - 88.0)	83.9 (83.3 - 84.4)	73.2 (72.5 - 74.0)	62.5 (61.5 - 63.4)	49.4 (48.1 - 50.7)
Digestive diseases	Pancreatitis	Both sexes	65-74	23441	12.57 (12.82 - 12.38)	88.8 (88.4 - 89.2)	80.8 (80.3 - 81.3)	74.7 (74.1 - 75.3)	59.5 (58.7 - 60.2)	40.2 (39.2 - 41.2)	21.6 (20.3 - 22.8)
Digestive diseases	Pancreatitis	Both sexes	75-84	21188	6.42 (6.57 - 6.28)	82.4 (81.8 - 82.9)	69.9 (69.2 - 70.5)	58.1 (57.3 - 58.8)	31.1 (30.3 - 32.0)	10.6 (9.9 - 11.3)	2.0 (1.5 - 2.5)
Digestive diseases	Pancreatitis	Both sexes	85-95	9214	2.62 (2.75 - 2.5)	67.6 (66.7 - 68.6)	46.7 (45.6 - 47.7)	29.7 (28.7 - 30.8)	6.3 (5.6 - 6.9)	0.6 (0.4 - 1.0)	0.0 (0.0 - 0.0)
Digestive diseases	Ulcerative colitis	Both sexes	All	207932	NR	93.9 (93.8 - 94.0)	88.4 (88.2 - 88.5)	83.9 (83.7 - 84.1)	74.8 (74.6 - 75.0)	67.5 (67.3 - 67.7)	61.3 (61.0 - 61.6)
Digestive diseases	Ulcerative colitis	Both sexes	16-24	22921	NR	99.8 (99.8 - 99.9)	99.6 (99.5 - 99.7)	99.5 (99.4 - 99.6)	99.0 (98.9 - 99.2)	98.4 (98.2 - 98.6)	97.8 (97.5 - 98.1)
Digestive diseases	Ulcerative colitis	Both sexes	25-34	29837	NR	99.7 (99.7 - 99.8)	99.4 (99.3 - 99.5)	99.2 (99.1 - 99.3)	98.6 (98.4 - 98.7)	97.7 (97.5 - 97.9)	96.8 (96.5 - 97.1)
Digestive diseases	Ulcerative colitis	Both sexes	35-44	25442	NR	99.5 (99.4 - 99.5)	98.8 (98.6 - 98.9)	98.1 (98.0 - 98.3)	96.7 (96.5 - 96.9)	94.7 (94.4 - 95.0)	92.3 (91.8 - 92.7)
Digestive diseases	Ulcerative colitis	Both sexes	45-54	26361	NR	98.5 (98.3 - 98.6)	96.8 (96.6 - 97.1)	95.6 (95.4 - 95.9)	92.2 (91.8 - 92.5)	88.4 (88.0 - 88.9)	82.8 (82.1 - 83.4)
Digestive diseases	Ulcerative colitis	Both sexes	55-64	30847	NR	96.2 (96.0 - 96.4)	92.5 (92.2 - 92.8)	89.5 (89.1 - 89.9)	81.8 (81.3 - 82.3)	72.4 (71.8 - 73.0)	60.7 (59.9 - 61.6)
Digestive diseases	Ulcerative colitis	Both sexes	65-74	32059	13.06 (13.25 - 12.9)	92.0 (91.7 - 92.3)	84.5 (84.1 - 84.9)	78.2 (77.8 - 78.7)	61.4 (60.7 - 62.0)	42.8 (42.0 - 43.5)	23.9 (23.0 - 24.8)
Digestive diseases	Ulcerative colitis	Both sexes	75-84	28171	5.88 (6.01 - 5.78)	84.0 (83.5 - 84.4)	68.8 (68.3 - 69.4)	55.3 (54.7 - 55.9)	27.5 (26.9 - 28.2)	9.6 (9.1 - 10.0)	2.4 (2.0 - 2.7)
Digestive diseases	Ulcerative colitis	Both sexes	85-95	11845	2.56 (2.63 - 2.48)	70.4 (69.6 - 71.2)	45.0 (44.0 - 45.9)	26.7 (25.9 - 27.6)	5.4 (5.0 - 6.0)	0.6 (0.4 - 0.8)	0.0 (0.0 - 0.0)
Digestive diseases	Upper digest diseases	Both sexes	All	914898	NR	93.7 (93.7 - 93.8)	88.3 (88.2 - 88.3)	83.6 (83.5 - 83.7)	73.3 (73.2 - 73.4)	64.2 (64.1 - 64.4)	56.0 (55.8 - 56.1)
Digestive diseases	Upper digest diseases	Both sexes	16-24	67737	NR	99.9 (99.9 - 99.9)	99.7 (99.6 - 99.7)	99.5 (99.5 - 99.6)	99.1 (99.1 - 99.2)	98.6 (98.5 - 98.7)	98.2 (98.0 - 98.3)
Digestive diseases	Upper digest diseases	Both sexes	25-34	92163	NR	99.8 (99.8 - 99.8)	99.5 (99.5 - 99.6)	99.3 (99.2 - 99.3)	98.6 (98.6 - 98.7)	98.0 (97.8 - 98.1)	96.9 (96.7 - 97.1)
Digestive diseases	Upper digest diseases	Both sexes	35-44	104581	NR	99.5 (99.4 - 99.5)	98.9 (98.8 - 99.0)	98.4 (98.3 - 98.5)	97.0 (96.9 - 97.1)	95.2 (95.0 - 95.4)	92.7 (92.5 - 93.0)
Digestive diseases	Upper digest diseases	Both sexes	45-54	127944	NR	98.5 (98.5 - 98.6)	97.0 (96.9 - 97.1)	95.7 (95.6 - 95.8)	92.3 (92.2 - 92.5)	88.2 (88.0 - 88.4)	82.4 (82.0 - 82.7)
Digestive diseases	Upper digest diseases	Both sexes	55-64	158785	NR	96.5 (96.4 - 96.6)	93.1 (93.0 - 93.3)	90.3 (90.2 - 90.5)	83.1 (82.9 - 83.3)	74.4 (74.1 - 74.7)	62.8 (62.4 - 63.2)
Digestive diseases	Upper digest diseases	Both sexes	65-74	169425	14.31 (14.4 - 14.23)	92.8 (92.7 - 92.9)	86.3 (86.1 - 86.5)	80.5 (80.3 - 80.7)	65.4 (65.1 - 65.6)	47.3 (47.0 - 47.7)	27.4 (27.0 - 27.8)
Digestive diseases	Upper digest diseases	Both sexes	75-84	140024	6.63 (6.69 - 6.58)	85.1 (84.9 - 85.3)	71.9 (71.7 - 72.1)	59.7 (59.4 - 60.0)	32.0 (31.7 - 32.3)	12.3 (12.1 - 12.6)	2.8 (2.7 - 3.0)
Digestive diseases	Upper digest diseases	Both sexes	85-95	52715	2.77 (2.82 - 2.72)	70.7 (70.3 - 71.1)	47.7 (47.3 - 48.2)	30.3 (29.9 - 30.7)	6.7 (6.5 - 7.0)	0.7 (0.6 - 0.8)	0.1 (0.0 - 0.1)
Digestive diseases	Vascular intestinal	Both sexes	All	31430	5.19 (5.34 - 5.06)	70.5 (69.9 - 71.0)	60.0 (59.4 - 60.6)	50.8 (50.2 - 51.4)	34.2 (33.5 - 34.8)	23.9 (23.2 - 24.6)	18.1 (17.2 - 18.9)
Digestive diseases	Vascular intestinal	Both sexes	16-24	286	NR	98.2 (95.8 - 99.3)	95.9 (92.8 - 97.7)	94.6 (91.0 - 96.8)	92.8 (88.5 - 95.5)	89.5 (83.8 - 93.2)	88.3 (82.0 - 92.4)
Digestive diseases	Vascular intestinal	Both sexes	25-34	470	NR	95.3 (92.9 - 96.9)	93.8 (91.1 - 95.7)	92.4 (89.4 - 94.5)	89.9 (86.4 - 92.6)	89.4 (85.7 - 92.2)	88.6 (84.6 - 91.7)
Digestive diseases	Vascular intestinal	Both sexes	35-44	811	NR	93.6 (91.7 - 95.1)	92.1 (90.0 - 93.8)	90.6 (88.3 - 92.5)	88.1 (85.4 - 90.3)	84.5 (81.1 - 87.3)	78.0 (72.7 - 82.4)
Digestive diseases	Vascular intestinal	Both sexes	45-54	1557	NR	87.5 (85.8 - 89.1)	83.8 (81.8 - 85.5)	80.4 (78.2 - 82.3)	74.3 (71.7 - 76.7)	67.9 (64.7 - 70.8)	61.0 (56.6 - 65.0)
Digestive diseases	Vascular intestinal	Both sexes	55-64	3894	14.22 (14.98 - 13.28)	82.7 (81.5 - 83.9)	76.5 (75.1 - 77.8)	71.7 (70.2 - 73.2)	59.3 (57.4 - 61.1)	47.8 (45.5 - 49.9)	37.1 (34.3 - 40.0)
Digestive diseases	Vascular intestinal	Both sexes	65-74	8389	7.44 (7.76 - 7.12)	76.7 (75.8 - 77.6)	67.5 (66.4 - 68.5)	59.0 (57.9 - 60.1)	40.6 (39.3 - 42.0)	24.8 (23.2 - 26.5)	12.8 (10.9 - 15.0)
Digestive diseases	Vascular intestinal	Both sexes	75-84	10835	3.66 (3.81 - 3.49)	66.7 (65.8 - 67.6)	54.0 (53.0 - 55.0)	41.7 (40.7 - 42.8)	18.4 (17.4 - 19.4)	5.4 (4.7 - 6.3)	1.2 (0.7 - 1.9)
Digestive diseases	Vascular intestinal	Both sexes	85-95	5002	0.74 (0.86 - 0.63)	47.2 (45.8 - 48.5)	30.9 (29.5 - 32.2)	17.9 (16.7 - 19.1)	3.4 (2.7 - 4.1)	0.3 (0.1 - 0.8)	0.2 (0.0 - 0.7)
Mental disorders & substance use	ADHD	Both sexes	All	198591	NR	99.7 (99.6 - 99.7)	99.0 (98.9 - 99.0)	98.2 (98.1 - 98.2)	96.0 (95.9 - 96.1)	93.3 (93.0 - 93.5)	89.5 (88.8 - 90.1)

Appendix Table A5: Unadjusted Median Survival and Survival Probabilities by Age, both sexes (Page 12 of 18)

Group	Diagnosis Category	Sex	Age	N	Median Survival (y)	1y (%)	3y (%)	5y (%)	10y (%)	15y (%)	20y (%)
Mental disorders & substance use	ADHD	Both sexes	16-24	82701	NR	99.8 (99.8 - 99.9)	99.5 (99.4 - 99.5)	99.0 (99.0 - 99.1)	97.9 (97.8 - 98.1)	96.5 (96.3 - 96.8)	95.1 (94.6 - 95.6)
Mental disorders & substance use	ADHD	Both sexes	25-34	54827	NR	99.6 (99.6 - 99.7)	99.0 (98.9 - 99.0)	98.3 (98.1 - 98.4)	96.4 (96.2 - 96.6)	94.3 (93.9 - 94.7)	89.2 (87.3 - 90.9)
Mental disorders & substance use	ADHD	Both sexes	35-44	35607	NR	99.6 (99.5 - 99.6)	98.8 (98.6 - 98.9)	97.8 (97.6 - 98.0)	95.1 (94.8 - 95.4)	91.5 (90.9 - 92.0)	85.6 (83.5 - 87.4)
Mental disorders & substance use	ADHD	Both sexes	45-54	19073	NR	99.4 (99.3 - 99.5)	98.2 (98.0 - 98.4)	96.7 (96.4 - 96.9)	92.2 (91.7 - 92.7)	86.0 (85.0 - 87.0)	75.0 (70.3 - 79.0)
Mental disorders & substance use	ADHD	Both sexes	55-64	5530	21.03	99.0 (98.7 - 99.2)	96.7 (96.2 - 97.2)	94.7 (93.9 - 95.3)	86.2 (84.9 - 87.4)	75.4 (72.7 - 77.8)	63.6 (55.8 - 70.4)
Mental disorders & substance use	ADHD	Both sexes	65-74	701	NR	96.4 (94.7 - 97.6)	92.3 (89.9 - 94.1)	86.8 (83.6 - 89.4)	72.3 (67.4 - 76.7)	55.9 (47.6 - 63.5)	50.2 (39.4 - 60.1)
Mental disorders & substance use	ADHD	Both sexes	75-84	123	8.16 (8.88 - 7.12)	92.5 (86.0 - 96.0)	82.2 (73.7 - 88.1)	70.9 (60.9 - 78.8)	28.6 (16.5 - 41.9)	16.4 (5.8 - 31.6)	16.4 (5.8 - 31.6)
Mental disorders & substance use	ADHD	Both sexes	85-95	29	3.12 (5.2 - 1.84)	78.5 (58.3 - 89.7)	53.9 (33.0 - 70.8)	33.1 (16.0 - 51.5)	10.4 (2.0 - 27.1)	0.0 (0.0 - 0.0)	0.0 (0.0 - 0.0)
Mental disorders & substance use	Alcohol use disorders	Both sexes	All	369308	NR	96.5 (96.5 - 96.6)	92.4 (92.4 - 92.5)	88.6 (88.5 - 88.7)	79.5 (79.4 - 79.7)	70.9 (70.7 - 71.0)	62.0 (61.8 - 62.3)
Mental disorders & substance use	Alcohol use disorders	Both sexes	16-24	89747	NR	99.7 (99.7 - 99.8)	99.2 (99.2 - 99.3)	98.7 (98.6 - 98.8)	97.6 (97.5 - 97.7)	96.5 (96.3 - 96.6)	95.4 (95.2 - 95.7)
Mental disorders & substance use	Alcohol use disorders	Both sexes	25-34	49753	NR	99.2 (99.1 - 99.3)	98.1 (98.0 - 98.2)	97.2 (97.0 - 97.3)	94.6 (94.4 - 94.9)	91.7 (91.3 - 92.0)	88.5 (87.9 - 89.0)
Mental disorders & substance use	Alcohol use disorders	Both sexes	35-44	49392	NR	98.9 (98.8 - 99.0)	97.2 (97.0 - 97.3)	95.4 (95.2 - 95.6)	90.9 (90.6 - 91.2)	85.1 (84.7 - 85.5)	77.1 (76.4 - 77.7)
Mental disorders & substance use	Alcohol use disorders	Both sexes	45-54	60794	22.72	97.7 (97.6 - 97.8)	94.5 (94.4 - 94.7)	91.4 (91.2 - 91.6)	82.6 (82.3 - 82.9)	71.6 (71.2 - 72.1)	58.3 (57.6 - 59.0)
Mental disorders & substance use	Alcohol use disorders	Both sexes	55-64	57711	14.83 (14.99 - 14.66)	94.9 (94.8 - 95.1)	88.4 (88.2 - 88.7)	82.0 (81.7 - 82.4)	65.9 (65.4 - 66.3)	49.4 (48.9 - 50.0)	33.1 (32.4 - 33.8)
Mental disorders & substance use	Alcohol use disorders	Both sexes	65-74	42299	9.26 (9.38 - 9.11)	90.4 (90.1 - 90.7)	80.2 (79.8 - 80.6)	70.3 (69.8 - 70.7)	46.4 (45.9 - 47.0)	25.2 (24.6 - 25.9)	10.2 (9.4 - 11.0)
Mental disorders & substance use	Alcohol use disorders	Both sexes	75-84	16969	5.27 (5.39 - 5.14)	84.8 (84.2 - 85.3)	67.8 (67.0 - 68.5)	51.9 (51.0 - 52.7)	20.7 (19.9 - 21.6)	4.9 (4.3 - 5.6)	0.5 (0.2 - 1.1)
Mental disorders & substance use	Alcohol use disorders	Both sexes	85-95	2604	2.98 (3.13 - 2.81)	75.6 (73.9 - 77.2)	49.7 (47.6 - 51.7)	30.4 (28.5 - 32.4)	4.4 (3.4 - 5.6)	0.1 (0.0 - 0.7)	0.0 (0.0 - 0.0)
Mental disorders & substance use	Anorexia nervosa	Both sexes	All	17392	NR	99.3 (99.1 - 99.4)	98.4 (98.2 - 98.6)	97.8 (97.6 - 98.0)	96.1 (95.7 - 96.4)	94.6 (94.1 - 95.0)	92.5 (91.8 - 93.2)
Mental disorders & substance use	Anorexia nervosa	Both sexes	16-24	12244	NR	99.9 (99.8 - 99.9)	99.6 (99.5 - 99.7)	99.4 (99.2 - 99.5)	98.7 (98.4 - 98.9)	98.0 (97.6 - 98.3)	97.2 (96.6 - 97.7)
Mental disorders & substance use	Anorexia nervosa	Both sexes	25-34	3026	NR	99.8 (99.5 - 99.9)	99.2 (98.8 - 99.5)	98.8 (98.3 - 99.2)	97.8 (97.0 - 98.3)	96.2 (95.1 - 97.1)	94.4 (92.8 - 95.7)
Mental disorders & substance use	Anorexia nervosa	Both sexes	35-44	1094	NR	99.0 (98.1 - 99.4)	97.7 (96.6 - 98.5)	96.7 (95.4 - 97.7)	93.2 (91.2 - 94.7)	90.5 (88.0 - 92.5)	86.0 (82.1 - 89.1)
Mental disorders & substance use	Anorexia nervosa	Both sexes	45-54	498	NR	98.6 (97.0 - 99.3)	95.9 (93.7 - 97.4)	94.3 (91.7 - 96.1)	87.7 (83.9 - 90.6)	80.5 (75.4 - 84.6)	71.4 (63.6 - 77.9)
Mental disorders & substance use	Anorexia nervosa	Both sexes	55-64	280	17.03 (19.55 - 15.14)	91.2 (87.2 - 94.0)	82.4 (77.2 - 86.5)	78.0 (72.4 - 82.7)	67.4 (60.8 - 73.2)	58.9 (51.4 - 65.6)	39.9 (30.2 - 49.5)
Mental disorders & substance use	Anorexia nervosa	Both sexes	65-74	143	6.5 (7.27 - 4.19)	80.3 (72.7 - 85.9)	65.5 (56.9 - 72.8)	55.4 (46.5 - 63.4)	26.1 (17.3 - 35.9)	18.1 (9.9 - 28.3)	18.1 (9.9 - 28.3)
Mental disorders & substance use	Anorexia nervosa	Both sexes	75-84	77	3.04 (5.18 - 1.69)	71.2 (59.6 - 80.0)	51.3 (39.6 - 61.9)	40.1 (28.7 - 51.3)	10.2 (3.7 - 20.4)	2.5 (0.2 - 11.1)	0.0 (0.0 - 0.0)
Mental disorders & substance use	Anorexia nervosa	Both sexes	85-95	29	0.92 (3.48 - 0.55)	48.3 (29.5 - 64.8)	37.9 (20.9 - 54.9)	17.2 (6.3 - 32.7)	3.4 (0.3 - 14.9)	0.0 (0.0 - 0.0)	0.0 (0.0 - 0.0)
Mental disorders & substance use	Anxiety disorders	Both sexes	All	912440	NR	97.3 (97.3 - 97.4)	94.8 (94.7 - 94.8)	92.6 (92.5 - 92.6)	87.6 (87.5 - 87.7)	82.8 (82.7 - 82.9)	77.4 (77.3 - 77.6)
Mental disorders & substance use	Anxiety disorders	Both sexes	16-24	218736	NR	99.8 (99.7 - 99.8)	99.4 (99.4 - 99.4)	99.0 (99.0 - 99.1)	98.1 (98.0 - 98.2)	97.1 (97.0 - 97.2)	96.1 (95.9 - 96.2)
Mental disorders & substance use	Anxiety disorders	Both sexes	25-34	193908	NR	99.7 (99.6 - 99.7)	99.2 (99.2 - 99.3)	98.8 (98.7 - 98.8)	97.6 (97.5 - 97.7)	96.3 (96.2 - 96.4)	94.5 (94.3 - 94.7)
Mental disorders & substance use	Anxiety disorders	Both sexes	35-44	162536	NR	99.4 (99.4 - 99.5)	98.8 (98.8 - 98.9)	98.2 (98.1 - 98.2)	96.4 (96.3 - 96.5)	94.0 (93.9 - 94.2)	90.6 (90.4 - 90.9)
Mental disorders & substance use	Anxiety disorders	Both sexes	45-54	133576	NR	98.8 (98.7 - 98.9)	97.4 (97.3 - 97.5)	96.1 (96.0 - 96.2)	92.3 (92.2 - 92.5)	87.2 (86.9 - 87.4)	79.9 (79.5 - 80.3)
Mental disorders & substance use	Anxiety disorders	Both sexes	55-64	89380	22.71	96.7 (96.6 - 96.8)	93.4 (93.2 - 93.6)	90.4 (90.2 - 90.6)	82.4 (82.1 - 82.7)	72.1 (71.7 - 72.5)	58.8 (58.1 - 59.4)
Mental disorders & substance use	Anxiety disorders	Both sexes	65-74	50777	12.15 (12.32 - 11.99)	90.6 (90.3 - 90.8)	82.6 (82.3 - 83.0)	75.4 (75.0 - 75.8)	57.9 (57.3 - 58.4)	39.3 (38.7 - 40.0)	20.8 (20.0 - 21.7)
Mental disorders & substance use	Anxiety disorders	Both sexes	75-84	42043	5.86 (5.95 - 5.78)	83.7 (83.4 - 84.1)	68.9 (68.4 - 69.3)	55.4 (54.9 - 55.9)	27.1 (26.6 - 27.6)	9.5 (9.0 - 9.9)	2.2 (1.8 - 2.6)
Mental disorders & substance use	Anxiety disorders	Both sexes	85-95	20525	2.7 (2.76 - 2.65)	71.8 (71.1 - 72.4)	46.6 (45.8 - 47.3)	28.5 (27.8 - 29.1)	5.7 (5.3 - 6.1)	0.6 (0.4 - 0.8)	0.1 (0.0 - 0.3)
Mental disorders & substance use	Bipolar disorder	Both sexes	All	105060	NR	98.2 (98.1 - 98.3)	95.9 (95.7 - 96.0)	93.5 (93.3 - 93.6)	87.3 (87.1 - 87.5)	79.7 (79.4 - 80.0)	69.5 (68.9 - 70.1)
Mental disorders & substance use	Bipolar disorder	Both sexes	16-24	20203	NR	99.6 (99.5 - 99.7)	99.1 (99.0 - 99.3)	98.5 (98.3 - 98.7)	97.2 (96.9 - 97.5)	96.0 (95.6 - 96.4)	93.8 (92.7 - 94.7)
Mental disorders & substance use	Bipolar disorder	Both sexes	25-34	24720	NR	99.5 (99.5 - 99.6)	98.7 (98.6 - 98.9)	98.0 (97.8 - 98.2)	96.4 (96.1 - 96.7)	94.6 (94.2 - 95.0)	92.9 (92.2 - 93.5)
Mental disorders & substance use	Bipolar disorder	Both sexes	35-44	20326	NR	99.4 (99.2 - 99.5)	98.5 (98.3 - 98.6)	97.6 (97.4 - 97.8)	95.4 (95.1 - 95.7)	92.1 (91.6 - 92.6)	87.6 (86.6 - 88.5)
Mental disorders & substance use	Bipolar disorder	Both sexes	45-54	16605	NR	98.9 (98.7 - 99.0)	97.4 (97.1 - 97.6)	95.7 (95.3 - 96.0)	91.0 (90.5 - 91.4)	84.4 (83.7 - 85.1)	74.9 (73.6 - 76.1)
Mental disorders & substance use	Bipolar disorder	Both sexes	55-64	11950	19.97 (20.35 - 19.61)	97.5 (97.2 - 97.8)	93.6 (93.2 - 94.1)	90.3 (89.8 - 90.9)	80.6 (79.8 - 81.4)	67.6 (66.5 - 68.6)	49.7 (48.1 - 51.2)
Mental disorders & substance use	Bipolar disorder	Both sexes	65-74	6571	12.02 (12.37 - 11.73)	94.6 (94.0 - 95.1)	87.5 (86.6 - 88.3)	80.6 (79.6 - 81.6)	59.7 (58.3 - 60.9)	35.8 (34.3 - 37.3)	16.0 (14.4 - 17.6)
Mental disorders & substance use	Bipolar disorder	Both sexes	75-84	3690	6.08 (6.35 - 5.88)	87.7 (86.6 - 88.7)	73.4 (71.9 - 74.8)	57.8 (56.1 - 59.4)	25.0 (23.5 - 26.6)	7.3 (6.3 - 8.5)	0.9 (0.5 - 1.7)
Mental disorders & substance use	Bipolar disorder	Both sexes	85-95	981	2.83 (3.21 - 2.44)	75.1 (72.3 - 77.7)	48.5 (45.3 - 51.7)	27.5 (24.6 - 30.4)	4.0 (2.7 - 5.5)	0.4 (0.1 - 1.2)	0.0 (0.0 - 0.0)
Mental disorders & substance use	Depressive disorders	Both sexes	All	690423	NR	96.0 (96.0 - 96.1)	92.0 (91.9 - 92.0)	88.4 (88.3 - 88.4)	80.9 (80.8 - 81.0)	74.5 (74.4 - 74.6)	68.3 (68.1 - 68.4)
Mental disorders & substance use	Depressive disorders	Both sexes	16-24	149179	NR	99.7 (99.7 - 99.8)	99.3 (99.3 - 99.4)	98.9 (98.9 - 99.0)	97.9 (97.9 - 98.0)	97.0 (96.8 - 97.1)	96.0 (95.8 - 96.2)
Mental disorders & substance use	Depressive disorders	Both sexes	25-34	128704	NR	99.6 (99.6 - 99.6)	99.1 (99.0 - 99.1)	98.6 (98.5 - 98.7)	97.4 (97.3 - 97.5)	96.1 (95.9 - 96.2)	94.3 (94.0 - 94.5)
Mental disorders & substance use	Depressive disorders	Both sexes	35-44	107860	NR	99.4 (99.4 - 99.5)	98.7 (98.6 - 98.7)	97.9 (97.8 - 98.0)	95.9 (95.8 - 96.1)	93.4 (93.2 - 93.6)	89.7 (89.4 - 90.1)
Mental disorders & substance use	Depressive disorders	Both sexes	45-54	95977	NR	98.7 (98.6 - 98.8)	97.2 (97.1 - 97.3)	95.6 (95.5 - 95.8)	91.4 (91.2 - 91.6)	86.2 (85.9 - 86.4)	79.3 (78.8 - 79.7)
Mental disorders & substance use	Depressive disorders	Both sexes	55-64	72841	21.83 (22.06 - 21.6)	96.7 (96.6 - 96.9)	93.1 (92.9 - 93.3)	89.7 (89.4 - 89.9)	80.6 (80.2 - 80.9)	69.6 (69.2 - 70.0)	56.0 (55.4 - 56.6)

Group	Diagnosis Category	Sex	Age	N	Median Survival (y)	1y (%)	3y (%)	5y (%)	10y (%)	15y (%)	20y (%)
Mental disorders & substance use	Depressive disorders	Both sexes	65-74	52716	10.75 (10.86 - 10.64)	91.2 (90.9 - 91.4)	82.0 (81.7 - 82.4)	73.4 (73.0 - 73.8)	53.1 (52.6 - 53.6)	33.1 (32.6 - 33.6)	16.2 (15.6 - 16.8)
Mental disorders & substance use	Depressive disorders	Both sexes	75-84	55390	5.15 (5.21 - 5.09)	83.5 (83.2 - 83.8)	66.6 (66.2 - 67.0)	51.1 (50.6 - 51.5)	21.6 (21.2 - 22.0)	6.6 (6.3 - 6.9)	1.3 (1.1 - 1.5)
Mental disorders & substance use	Depressive disorders	Both sexes	85-95	26948	2.5 (2.55 - 2.44)	70.6 (70.1 - 71.2)	44.0 (43.4 - 44.6)	25.1 (24.6 - 25.7)	4.3 (4.0 - 4.5)	0.4 (0.3 - 0.5)	0.1 (0.0 - 0.2)
Mental disorders & substance use	Drug use disorders	Both sexes	All	183777	NR	97.1 (97.0 - 97.2)	93.7 (93.5 - 93.8)	90.6 (90.5 - 90.8)	83.6 (83.4 - 83.8)	76.5 (76.3 - 76.8)	69.3 (69.0 - 69.7)
Mental disorders & substance use	Drug use disorders	Both sexes	16-24	63595	NR	99.2 (99.2 - 99.3)	98.0 (97.8 - 98.1)	96.7 (96.5 - 96.8)	93.8 (93.6 - 94.0)	90.9 (90.6 - 91.2)	88.5 (88.0 - 89.0)
Mental disorders & substance use	Drug use disorders	Both sexes	25-34	42567	NR	98.7 (98.6 - 98.8)	97.0 (96.8 - 97.1)	95.5 (95.3 - 95.7)	91.9 (91.6 - 92.2)	88.1 (87.6 - 88.5)	83.9 (83.3 - 84.6)
Mental disorders & substance use	Drug use disorders	Both sexes	35-44	26291	NR	98.3 (98.1 - 98.5)	96.1 (95.8 - 96.3)	94.1 (93.8 - 94.4)	88.8 (88.4 - 89.2)	82.2 (81.6 - 82.8)	74.0 (73.1 - 74.9)
Mental disorders & substance use	Drug use disorders	Both sexes	45-54	21415	22.55	97.1 (96.8 - 97.3)	93.3 (93.0 - 93.6)	89.8 (89.3 - 90.2)	80.3 (79.7 - 80.9)	69.3 (68.5 - 70.1)	57.0 (55.8 - 58.1)
Mental disorders & substance use	Drug use disorders	Both sexes	55-64	14086	15.4 (15.77 - 15.12)	94.3 (93.9 - 94.6)	87.8 (87.2 - 88.3)	81.8 (81.1 - 82.4)	66.6 (65.7 - 67.5)	51.6 (50.4 - 52.7)	36.3 (34.8 - 37.8)
Mental disorders & substance use	Drug use disorders	Both sexes	65-74	8493	9.45 (9.79 - 9.09)	90.1 (89.4 - 90.7)	78.9 (78.0 - 79.8)	69.1 (68.1 - 70.1)	48.0 (46.7 - 49.3)	29.0 (27.5 - 30.4)	13.9 (12.3 - 15.7)
Mental disorders & substance use	Drug use disorders	Both sexes	75-84	5081	5.08 (5.27 - 4.88)	82.8 (81.7 - 83.8)	64.9 (63.5 - 66.2)	50.5 (49.0 - 51.9)	21.4 (20.0 - 22.8)	6.0 (5.0 - 7.2)	1.5 (0.8 - 2.5)
Mental disorders & substance use	Drug use disorders	Both sexes	85-95	2166	2.33 (2.48 - 2.13)	70.8 (68.8 - 72.7)	42.5 (40.3 - 44.7)	23.9 (21.9 - 25.8)	4.1 (3.1 - 5.3)	0.3 (0.1 - 0.9)	0.0 (0.0 - 0.0)
Mental disorders & substance use	Dysthymia	Both sexes	All	29346	NR	98.8 (98.7 - 98.9)	97.0 (96.8 - 97.2)	95.0 (94.7 - 95.2)	89.7 (89.3 - 90.1)	83.9 (83.3 - 84.4)	76.8 (75.9 - 77.6)
Mental disorders & substance use	Dysthymia	Both sexes	16-24	6161	NR	99.7 (99.5 - 99.8)	99.2 (99.0 - 99.4)	98.9 (98.5 - 99.1)	97.5 (97.0 - 97.9)	96.4 (95.7 - 97.0)	95.4 (94.4 - 96.2)
Mental disorders & substance use	Dysthymia	Both sexes	25-34	5993	NR	99.6 (99.4 - 99.7)	98.9 (98.5 - 99.1)	98.4 (98.0 - 98.7)	97.2 (96.6 - 97.6)	95.7 (94.9 - 96.3)	93.5 (92.0 - 94.7)
Mental disorders & substance use	Dysthymia	Both sexes	35-44	5257	NR	99.4 (99.2 - 99.6)	98.6 (98.2 - 98.9)	97.8 (97.3 - 98.1)	95.8 (95.2 - 96.4)	93.1 (92.2 - 93.9)	88.6 (86.9 - 90.0)
Mental disorders & substance use	Dysthymia	Both sexes	45-54	5427	NR	99.5 (99.2 - 99.6)	98.3 (98.0 - 98.7)	97.1 (96.6 - 97.5)	93.0 (92.2 - 93.7)	87.4 (86.3 - 88.5)	81.0 (79.2 - 82.6)
Mental disorders & substance use	Dysthymia	Both sexes	55-64	3885	NR	98.6 (98.2 - 98.9)	96.2 (95.5 - 96.8)	93.4 (92.6 - 94.2)	85.7 (84.4 - 86.8)	75.7 (74.0 - 77.4)	61.3 (58.5 - 64.0)
Mental disorders & substance use	Dysthymia	Both sexes	65-74	1492	12.86 (13.56 - 12.37)	96.4 (95.3 - 97.2)	90.7 (89.1 - 92.1)	83.6 (81.5 - 85.4)	63.6 (60.8 - 66.2)	41.4 (38.1 - 44.5)	20.8 (17.2 - 24.6)
Mental disorders & substance use	Dysthymia	Both sexes	75-84	877	6.78 (7.17 - 6.31)	90.8 (88.6 - 92.5)	78.3 (75.3 - 80.9)	64.0 (60.6 - 67.2)	31.5 (28.1 - 34.9)	11.6 (9.1 - 14.4)	2.3 (0.9 - 5.0)
Mental disorders & substance use	Dysthymia	Both sexes	85-95	251	3.37 (3.73 - 2.9)	76.7 (70.9 - 81.5)	56.0 (49.5 - 61.9)	31.7 (25.8 - 37.6)	6.4 (3.6 - 10.2)	0.8 (0.1 - 3.7)	0.8 (0.1 - 3.7)
Mental disorders & substance use	Major depression	Both sexes	All	683332	NR	96.0 (96.0 - 96.1)	91.9 (91.8 - 92.0)	88.3 (88.2 - 88.4)	80.7 (80.6 - 80.8)	74.4 (74.2 - 74.5)	68.1 (67.9 - 68.3)
Mental disorders & substance use	Major depression	Both sexes	16-24	147380	NR	99.7 (99.7 - 99.8)	99.3 (99.3 - 99.4)	98.9 (98.9 - 99.0)	97.9 (97.9 - 98.0)	96.9 (96.8 - 97.1)	96.0 (95.8 - 96.1)
Mental disorders & substance use	Major depression	Both sexes	25-34	127359	NR	99.6 (99.6 - 99.6)	99.0 (99.0 - 99.1)	98.6 (98.5 - 98.7)	97.4 (97.3 - 97.5)	96.0 (95.9 - 96.2)	94.3 (94.0 - 94.5)
Mental disorders & substance use	Major depression	Both sexes	35-44	106536	NR	99.4 (99.4 - 99.5)	98.7 (98.6 - 98.7)	97.9 (97.8 - 98.0)	95.9 (95.8 - 96.1)	93.4 (93.2 - 93.5)	89.7 (89.4 - 90.0)
Mental disorders & substance use	Major depression	Both sexes	45-54	94667	NR	98.7 (98.6 - 98.8)	97.1 (97.0 - 97.2)	95.6 (95.5 - 95.7)	91.4 (91.2 - 91.6)	86.1 (85.8 - 86.4)	79.2 (78.7 - 79.6)
Mental disorders & substance use	Major depression	Both sexes	55-64	72072	21.78 (21.99 - 21.55)	96.7 (96.6 - 96.8)	93.1 (92.9 - 93.2)	89.6 (89.4 - 89.8)	80.4 (80.1 - 80.7)	69.4 (69.0 - 69.8)	55.8 (55.1 - 56.4)
Mental disorders & substance use	Major depression	Both sexes	65-74	52446	10.73 (10.84 - 10.62)	91.1 (90.9 - 91.4)	82.0 (81.6 - 82.3)	73.3 (72.9 - 73.7)	53.0 (52.5 - 53.5)	33.0 (32.5 - 33.5)	16.2 (15.5 - 16.8)
Mental disorders & substance use	Major depression	Both sexes	75-84	55201	5.14 (5.2 - 5.07)	83.4 (83.1 - 83.8)	66.5 (66.1 - 66.9)	51.0 (50.5 - 51.4)	21.5 (21.1 - 21.9)	6.5 (6.3 - 6.8)	1.3 (1.1 - 1.5)
Mental disorders & substance use	Major depression	Both sexes	85-95	26865	2.49 (2.55 - 2.44)	70.6 (70.1 - 71.1)	43.9 (43.3 - 44.5)	25.1 (24.6 - 25.7)	4.2 (4.0 - 4.5)	0.4 (0.3 - 0.5)	0.1 (0.0 - 0.2)
Mental disorders & substance use	Opioid use disorders	Both sexes	All	45670	NR	95.2 (95.0 - 95.4)	90.0 (89.7 - 90.3)	85.8 (85.5 - 86.2)	76.8 (76.3 - 77.3)	68.4 (67.8 - 69.0)	60.7 (59.9 - 61.6)
Mental disorders & substance use	Opioid use disorders	Both sexes	16-24	9757	NR	98.2 (98.0 - 98.5)	95.2 (94.8 - 95.6)	92.9 (92.3 - 93.4)	87.4 (86.6 - 88.2)	82.3 (81.2 - 83.4)	78.7 (77.2 - 80.1)
Mental disorders & substance use	Opioid use disorders	Both sexes	25-34	12347	NR	97.6 (97.3 - 97.9)	94.6 (94.2 - 95.0)	91.9 (91.4 - 92.4)	86.3 (85.6 - 87.0)	80.5 (79.5 - 81.5)	75.0 (73.5 - 76.4)
Mental disorders & substance use	Opioid use disorders	Both sexes	35-44	8106	NR	97.5 (97.2 - 97.9)	94.6 (94.1 - 95.1)	91.7 (91.0 - 92.3)	84.9 (84.0 - 85.8)	76.8 (75.6 - 78.0)	68.7 (66.9 - 70.4)
Mental disorders & substance use	Opioid use disorders	Both sexes	45-54	6557	21.17 (22.5 - 20.39)	96.4 (95.9 - 96.8)	92.0 (91.3 - 92.7)	87.7 (86.8 - 88.5)	77.2 (76.0 - 78.3)	65.4 (63.8 - 67.0)	53.7 (51.2 - 56.1)
Mental disorders & substance use	Opioid use disorders	Both sexes	55-64	4123	14.67 (15.21 - 13.8)	92.7 (91.9 - 93.5)	85.3 (84.1 - 86.3)	78.7 (77.3 - 80.0)	63.0 (61.2 - 64.7)	48.6 (46.4 - 50.9)	30.5 (26.9 - 34.1)
Mental disorders & substance use	Opioid use disorders	Both sexes	65-74	2476	7.7 (8.3 - 7.37)	87.2 (85.8 - 88.4)	73.1 (71.3 - 74.9)	63.7 (61.6 - 65.7)	41.0 (38.5 - 43.4)	23.7 (20.8 - 26.7)	10.9 (7.4 - 15.2)
Mental disorders & substance use	Opioid use disorders	Both sexes	75-84	1476	3.59 (3.93 - 3.25)	75.8 (73.4 - 77.9)	54.7 (52.0 - 57.4)	41.0 (38.2 - 43.7)	17.2 (14.7 - 19.8)	3.2 (1.6 - 5.5)	0.0 (0.0 - 0.0)
Mental disorders & substance use	Opioid use disorders	Both sexes	85-95	787	1.58 (1.81 - 1.34)	60.2 (56.7 - 63.6)	31.9 (28.5 - 35.3)	17.0 (14.3 - 20.0)	1.4 (0.5 - 3.2)	0.0 (0.0 - 0.0)	0.0 (0.0 - 0.0)
Mental disorders & substance use	Schizophrenia	Both sexes	All	41120	NR	95.9 (95.7 - 96.1)	91.4 (91.1 - 91.7)	87.3 (87.0 - 87.7)	77.2 (76.7 - 77.6)	66.9 (66.4 - 67.4)	56.6 (55.9 - 57.3)
Mental disorders & substance use	Schizophrenia	Both sexes	16-24	4224	NR	99.3 (99.0 - 99.5)	97.9 (97.4 - 98.3)	96.9 (96.3 - 97.4)	94.6 (93.7 - 95.3)	92.4 (91.3 - 93.3)	89.2 (87.6 - 90.6)
Mental disorders & substance use	Schizophrenia	Both sexes	25-34	7734	NR	99.2 (99.0 - 99.4)	97.8 (97.4 - 98.1)	96.8 (96.4 - 97.2)	94.1 (93.5 - 94.7)	91.4 (90.6 - 92.2)	87.7 (86.5 - 88.8)
Mental disorders & substance use	Schizophrenia	Both sexes	35-44	8085	NR	99.0 (98.7 - 99.2)	97.5 (97.1 - 97.8)	96.2 (95.8 - 96.6)	92.4 (91.7 - 93.0)	87.0 (86.2 - 87.8)	80.2 (79.0 - 81.3)
Mental disorders & substance use	Schizophrenia	Both sexes	45-54	8120	22.9	98.1 (97.7 - 98.3)	95.2 (94.7 - 95.6)	92.8 (92.2 - 93.3)	84.3 (83.4 - 85.1)	73.7 (72.6 - 74.7)	59.3 (57.8 - 60.8)
Mental disorders & substance use	Schizophrenia	Both sexes	55-64	6500	14.98 (15.31 - 14.63)	95.3 (94.8 - 95.8)	89.7 (88.9 - 90.4)	84.2 (83.3 - 85.1)	68.2 (67.0 - 69.4)	49.9 (48.5 - 51.2)	32.6 (31.0 - 34.2)
Mental disorders & substance use	Schizophrenia	Both sexes	65-74	3769	8.53 (8.86 - 8.19)	89.9 (88.8 - 90.8)	79.7 (78.4 - 81.0)	68.2 (66.7 - 69.7)	43.2 (41.5 - 44.9)	21.7 (20.2 - 23.3)	7.7 (6.4 - 9.1)
Mental disorders & substance use	Schizophrenia	Both sexes	75-84	2143	4.15 (4.44 - 3.9)	79.1 (77.3 - 80.7)	59.1 (56.9 - 61.1)	43.6 (41.4 - 45.7)	16.0 (14.3 - 17.7)	4.3 (3.4 - 5.4)	1.2 (0.6 - 2.2)
Mental disorders & substance use	Schizophrenia	Both sexes	85-95	535	2.15 (2.43 - 1.79)	64.4 (60.1 - 68.3)	39.2 (35.0 - 43.4)	21.1 (17.6 - 24.7)	3.1 (1.8 - 5.0)	0.7 (0.1 - 2.1)	0.0 (0.0 - 0.0)
Mental disorders & substance use	Self-harm	Both sexes	All	136115	NR	96.0 (95.8 - 96.1)	92.2 (92.1 - 92.4)	89.0 (88.8 - 89.2)	82.4 (82.2 - 82.7)	76.8 (76.5 - 77.0)	71.2 (70.8 - 71.5)
Mental disorders & substance use	Self-harm	Both sexes	16-24	45794	NR	99.1 (99.0 - 99.2)	98.0 (97.9 - 98.1)	97.1 (96.9 - 97.2)	95.2 (94.9 - 95.4)	93.4 (93.1 - 93.7)	91.9 (91.5 - 92.2)

Appendix Table A5: Unadjusted Median Survival and Survival Probabilities by Age, both sexes (Page 14 of 18)

Group	Diagnosis Category	Sex	Age	N	Median Survival (y)	1y (%)	3y (%)	5y (%)	10y (%)	15y (%)	20y (%)
Mental disorders & substance use	Self-harm	Both sexes	25-34	26304	NR	97.9 (97.7 - 98.1)	96.0 (95.7 - 96.2)	94.5 (94.2 - 94.7)	91.4 (91.0 - 91.7)	88.6 (88.1 - 89.0)	85.6 (85.0 - 86.3)
Mental disorders & substance use	Self-harm	Both sexes	35-44	19975	NR	97.3 (97.0 - 97.5)	94.9 (94.6 - 95.2)	92.9 (92.5 - 93.3)	88.3 (87.8 - 88.8)	83.5 (82.9 - 84.1)	77.8 (77.0 - 78.6)
Mental disorders & substance use	Self-harm	Both sexes	45-54	17837	NR	96.1 (95.8 - 96.4)	92.6 (92.2 - 93.0)	89.5 (89.0 - 89.9)	81.8 (81.1 - 82.4)	72.7 (71.9 - 73.5)	62.3 (61.1 - 63.5)
Mental disorders & substance use	Self-harm	Both sexes	55-64	11433	17.06 (17.48 - 16.63)	93.2 (92.7 - 93.7)	87.2 (86.6 - 87.8)	81.6 (80.9 - 82.3)	68.7 (67.7 - 69.7)	55.9 (54.8 - 57.1)	42.1 (40.6 - 43.6)
Mental disorders & substance use	Self-harm	Both sexes	65-74	6879	9.14 (9.49 - 8.72)	88.4 (87.6 - 89.1)	77.6 (76.6 - 78.6)	67.3 (66.1 - 68.5)	46.5 (45.1 - 47.9)	28.1 (26.6 - 29.6)	14.5 (13.0 - 16.2)
Mental disorders & substance use	Self-harm	Both sexes	75-84	5164	4.98 (5.22 - 4.76)	82.0 (80.9 - 83.0)	65.6 (64.2 - 66.9)	49.8 (48.3 - 51.2)	19.9 (18.5 - 21.2)	5.9 (5.0 - 6.9)	1.1 (0.6 - 1.8)
Mental disorders & substance use	Self-harm	Both sexes	85-95	2629	2.57 (2.75 - 2.4)	71.1 (69.3 - 72.8)	45.6 (43.6 - 47.5)	26.8 (25.0 - 28.7)	4.5 (3.5 - 5.6)	0.1 (0.0 - 0.6)	0.0 (0.0 - 0.0)
Musculoskeletal disorders	Gout	Both sexes	All	134140	7.77 (7.85 - 7.69)	86.9 (86.7 - 87.0)	72.9 (72.7 - 73.2)	61.9 (61.6 - 62.2)	42.4 (42.1 - 42.7)	30.1 (29.7 - 30.5)	22.6 (22.1 - 23.0)
Musculoskeletal disorders	Gout	Both sexes	16-24	427	NR	99.8 (98.3 - 100.0)	99.5 (98.1 - 99.9)	99.3 (97.7 - 99.8)	98.2 (95.9 - 99.2)	95.5 (91.8 - 97.6)	94.7 (90.5 - 97.1)
Musculoskeletal disorders	Gout	Both sexes	25-34	2111	NR	99.5 (99.1 - 99.7)	98.9 (98.4 - 99.3)	98.2 (97.5 - 98.7)	96.3 (95.2 - 97.2)	94.1 (92.5 - 95.4)	90.9 (88.4 - 92.9)
Musculoskeletal disorders	Gout	Both sexes	35-44	5107	NR	99.4 (99.2 - 99.6)	98.5 (98.2 - 98.9)	97.6 (97.1 - 98.0)	94.9 (94.1 - 95.6)	90.7 (89.5 - 91.8)	85.9 (84.1 - 87.5)
Musculoskeletal disorders	Gout	Both sexes	45-54	10571	NR	98.5 (98.3 - 98.7)	96.4 (96.0 - 96.7)	94.3 (93.8 - 94.7)	88.4 (87.7 - 89.1)	80.4 (79.2 - 81.4)	71.5 (69.8 - 73.1)
Musculoskeletal disorders	Gout	Both sexes	55-64	18880	18.07 (18.45 - 17.67)	97.1 (96.8 - 97.3)	92.4 (92.0 - 92.8)	87.7 (87.2 - 88.2)	75.1 (74.4 - 75.8)	59.9 (58.8 - 60.9)	43.9 (42.4 - 45.4)
Musculoskeletal disorders	Gout	Both sexes	65-74	31987	10.29 (10.48 - 10.16)	92.9 (92.6 - 93.1)	83.4 (83.0 - 83.8)	74.3 (73.8 - 74.9)	51.4 (50.7 - 52.1)	30.0 (29.2 - 30.9)	14.3 (13.3 - 15.3)
Musculoskeletal disorders	Gout	Both sexes	75-84	40096	4.97 (5.04 - 4.9)	84.2 (83.9 - 84.6)	65.7 (65.3 - 66.2)	49.8 (49.2 - 50.3)	20.1 (19.6 - 20.7)	5.3 (4.9 - 5.7)	0.8 (0.6 - 1.1)
Musculoskeletal disorders	Gout	Both sexes	85-95	23786	2.13 (2.18 - 2.08)	67.7 (67.1 - 68.3)	39.1 (38.4 - 39.7)	21.0 (20.5 - 21.6)	3.1 (2.8 - 3.4)	0.2 (0.1 - 0.4)	0.0 (0.0 - 0.2)
Musculoskeletal disorders	Low back pain	Both sexes	All	567352	NR	97.7 (97.7 - 97.7)	94.5 (94.5 - 94.6)	91.3 (91.2 - 91.4)	83.4 (83.3 - 83.5)	75.8 (75.7 - 76.0)	68.9 (68.7 - 69.1)
Musculoskeletal disorders	Low back pain	Both sexes	16-24	31615	NR	99.9 (99.8 - 99.9)	99.7 (99.6 - 99.7)	99.5 (99.4 - 99.5)	99.0 (98.9 - 99.1)	98.5 (98.3 - 98.6)	97.9 (97.6 - 98.2)
Musculoskeletal disorders	Low back pain	Both sexes	25-34	71236	NR	99.8 (99.8 - 99.9)	99.6 (99.6 - 99.6)	99.4 (99.4 - 99.5)	98.8 (98.7 - 98.9)	98.1 (98.0 - 98.2)	97.1 (96.9 - 97.3)
Musculoskeletal disorders	Low back pain	Both sexes	35-44	101023	NR	99.8 (99.7 - 99.8)	99.5 (99.4 - 99.5)	99.1 (99.0 - 99.2)	98.2 (98.1 - 98.3)	96.7 (96.6 - 96.8)	94.8 (94.5 - 95.0)
Musculoskeletal disorders	Low back pain	Both sexes	45-54	107127	NR	99.5 (99.4 - 99.5)	98.8 (98.7 - 98.9)	98.1 (98.0 - 98.2)	95.8 (95.7 - 95.9)	92.3 (92.1 - 92.5)	87.2 (86.9 - 87.6)
Musculoskeletal disorders	Low back pain	Both sexes	55-64	97106	NR	98.6 (98.5 - 98.7)	96.8 (96.6 - 96.9)	94.9 (94.7 - 95.0)	88.9 (88.7 - 89.2)	80.5 (80.1 - 80.8)	68.4 (67.9 - 69.0)
Musculoskeletal disorders	Low back pain	Both sexes	65-74	76817	15.48 (15.62 - 15.34)	96.5 (96.4 - 96.6)	91.9 (91.7 - 92.1)	86.9 (86.6 - 87.1)	72.0 (71.6 - 72.4)	52.1 (51.6 - 52.7)	29.7 (29.0 - 30.5)
Musculoskeletal disorders	Low back pain	Both sexes	75-84	59982	8.19 (8.27 - 8.12)	92.9 (92.7 - 93.1)	82.3 (81.9 - 82.6)	70.3 (69.9 - 70.7)	38.9 (38.4 - 39.4)	14.7 (14.2 - 15.1)	3.8 (3.4 - 4.1)
Musculoskeletal disorders	Low back pain	Both sexes	85-95	21764	4.02 (4.08 - 3.96)	83.6 (83.1 - 84.1)	60.9 (60.2 - 61.6)	40.3 (39.6 - 41.0)	9.2 (8.8 - 9.7)	1.0 (0.8 - 1.3)	0.0 (0.0 - 0.2)
Musculoskeletal disorders	Lupus	Both sexes	All	17427	NR	97.0 (96.8 - 97.3)	93.1 (92.7 - 93.5)	89.4 (88.9 - 89.9)	80.0 (79.4 - 80.7)	71.0 (70.2 - 71.8)	61.4 (60.4 - 62.4)
Musculoskeletal disorders	Lupus	Both sexes	16-24	1157	NR	99.7 (99.2 - 99.9)	99.5 (98.8 - 99.8)	99.4 (98.6 - 99.7)	99.0 (98.1 - 99.5)	98.2 (96.9 - 99.0)	97.9 (96.4 - 98.8)
Musculoskeletal disorders	Lupus	Both sexes	25-34	2169	NR	99.6 (99.3 - 99.8)	99.5 (99.1 - 99.7)	99.2 (98.7 - 99.5)	98.5 (97.9 - 99.0)	97.5 (96.5 - 98.2)	96.1 (94.8 - 97.1)
Musculoskeletal disorders	Lupus	Both sexes	35-44	2637	NR	99.8 (99.5 - 99.9)	99.3 (98.9 - 99.6)	99.0 (98.5 - 99.3)	97.4 (96.6 - 98.0)	95.3 (94.2 - 96.2)	91.6 (89.9 - 93.0)
Musculoskeletal disorders	Lupus	Both sexes	45-54	3007	NR	99.2 (98.8 - 99.5)	97.9 (97.3 - 98.4)	96.5 (95.8 - 97.2)	92.8 (91.7 - 93.7)	87.0 (85.5 - 88.3)	77.1 (75.0 - 79.1)
Musculoskeletal disorders	Lupus	Both sexes	55-64	3384	21.7 (22.48 - 21.17)	98.1 (97.6 - 98.6)	95.3 (94.5 - 96.0)	91.6 (90.6 - 92.6)	82.8 (81.3 - 84.1)	71.3 (69.5 - 73.1)	57.1 (54.8 - 59.3)
Musculoskeletal disorders	Lupus	Both sexes	65-74	2944	13.55 (13.89 - 13.08)	95.3 (94.5 - 96.0)	88.5 (87.3 - 89.6)	81.9 (80.4 - 83.3)	63.5 (61.5 - 65.4)	43.7 (41.4 - 46.0)	21.8 (19.2 - 24.4)
Musculoskeletal disorders	Lupus	Both sexes	75-84	1697	6.92 (7.4 - 6.5)	89.4 (87.8 - 90.8)	75.5 (73.3 - 77.6)	64.0 (61.6 - 66.4)	33.0 (30.4 - 35.6)	11.4 (9.4 - 13.5)	2.2 (1.3 - 3.7)
Musculoskeletal disorders	Lupus	Both sexes	85-95	427	3.36 (3.93 - 3.01)	78.0 (73.7 - 81.7)	55.1 (50.2 - 59.8)	36.6 (31.8 - 41.4)	5.5 (3.2 - 8.6)	0.7 (0.1 - 3.2)	0.0 (0.0 - 0.0)
Musculoskeletal disorders	Neck pain	Both sexes	All	249816	NR	98.7 (98.7 - 98.8)	96.9 (96.9 - 97.0)	95.1 (95.0 - 95.2)	90.1 (89.9 - 90.2)	84.4 (84.2 - 84.6)	78.2 (77.9 - 78.5)
Musculoskeletal disorders	Neck pain	Both sexes	16-24	15615	NR	99.9 (99.9 - 100.0)	99.8 (99.7 - 99.9)	99.6 (99.5 - 99.7)	99.1 (98.9 - 99.3)	98.6 (98.3 - 98.8)	97.8 (97.3 - 98.2)
Musculoskeletal disorders	Neck pain	Both sexes	25-34	29003	NR	99.9 (99.8 - 99.9)	99.7 (99.6 - 99.7)	99.5 (99.4 - 99.6)	99.0 (98.9 - 99.1)	98.2 (98.0 - 98.4)	97.4 (97.1 - 97.7)
Musculoskeletal disorders	Neck pain	Both sexes	35-44	50183	NR	99.8 (99.8 - 99.9)	99.6 (99.5 - 99.6)	99.3 (99.2 - 99.4)	98.4 (98.3 - 98.5)	97.1 (96.9 - 97.3)	94.8 (94.4 - 95.1)
Musculoskeletal disorders	Neck pain	Both sexes	45-54	63066	NR	99.7 (99.6 - 99.7)	99.1 (99.0 - 99.2)	98.5 (98.4 - 98.6)	96.5 (96.4 - 96.7)	93.4 (93.1 - 93.7)	88.3 (87.7 - 88.8)
Musculoskeletal disorders	Neck pain	Both sexes	55-64	47667	NR	99.1 (99.0 - 99.2)	97.7 (97.6 - 97.8)	96.2 (96.0 - 96.4)	91.3 (91.0 - 91.6)	83.7 (83.2 - 84.1)	72.3 (71.5 - 73.1)
Musculoskeletal disorders	Neck pain	Both sexes	65-74	24908	16.36 (16.62 - 16.13)	97.3 (97.1 - 97.5)	93.6 (93.2 - 93.9)	89.3 (88.9 - 89.7)	75.4 (74.8 - 76.1)	55.7 (54.7 - 56.7)	32.6 (31.1 - 34.2)
Musculoskeletal disorders	Neck pain	Both sexes	75-84	14883	8.88 (9.03 - 8.7)	93.8 (93.4 - 94.1)	84.0 (83.3 - 84.6)	73.4 (72.6 - 74.1)	43.5 (42.4 - 44.5)	17.5 (16.4 - 18.7)	4.5 (3.6 - 5.6)
Musculoskeletal disorders	Neck pain	Both sexes	85-95	4355	3.97 (4.12 - 3.81)	82.5 (81.3 - 83.6)	59.8 (58.2 - 61.3)	40.3 (38.7 - 41.9)	9.8 (8.6 - 11.0)	1.5 (0.9 - 2.5)	0.6 (0.1 - 2.2)
Musculoskeletal disorders	Osteoarthritis	Both sexes	All	844730	20.37 (20.45 - 20.29)	98.4 (98.3 - 98.4)	95.1 (95.0 - 95.1)	91.1 (91.0 - 91.2)	79.1 (79.0 - 79.2)	65.1 (65.0 - 65.3)	50.9 (50.7 - 51.1)
Musculoskeletal disorders	Osteoarthritis	Both sexes	16-24	4000	NR	100.0 (100.0 - 100.0)	99.7 (99.5 - 99.9)	99.5 (99.2 - 99.7)	99.3 (99.0 - 99.5)	98.9 (98.4 - 99.2)	98.9 (98.4 - 99.2)
Musculoskeletal disorders	Osteoarthritis	Both sexes	25-34	12239	NR	99.9 (99.9 - 100.0)	99.7 (99.6 - 99.8)	99.6 (99.4 - 99.7)	99.0 (98.8 - 99.2)	98.2 (97.9 - 98.5)	97.4 (96.9 - 97.8)
Musculoskeletal disorders	Osteoarthritis	Both sexes	35-44	41859	NR	99.9 (99.8 - 99.9)	99.6 (99.6 - 99.7)	99.3 (99.2 - 99.4)	98.3 (98.2 - 98.4)	97.0 (96.7 - 97.1)	94.7 (94.3 - 95.0)
Musculoskeletal disorders	Osteoarthritis	Both sexes	45-54	126896	NR	99.8 (99.8 - 99.8)	99.3 (99.2 - 99.3)	98.6 (98.6 - 98.7)	96.5 (96.4 - 96.7)	93.2 (93.0 - 93.4)	87.8 (87.4 - 88.1)
Musculoskeletal disorders	Osteoarthritis	Both sexes	55-64	228141	NR	99.6 (99.6 - 99.6)	98.5 (98.4 - 98.5)	97.1 (97.0 - 97.2)	92.3 (92.2 - 92.4)	84.5 (84.3 - 84.7)	71.8 (71.5 - 72.2)
Musculoskeletal disorders	Osteoarthritis	Both sexes	65-74	236338	17.19 (17.26 - 17.12)	99.0 (98.9 - 99.0)	96.4 (96.4 - 96.5)	93.0 (92.9 - 93.1)	80.7 (80.5 - 80.9)	60.8 (60.5 - 61.1)	35.4 (35.0 - 35.8)

Appendix Table A5: Unadjusted Median Survival and Survival Probabilities by Age, both sexes (Page 15 of 18)

Group	Diagnosis Category	Sex	Age	N	Median Survival (y)	1y (%)	3y (%)	5y (%)	10y (%)	15y (%)	20y (%)
Musculoskeletal disorders	Osteoarthritis	Both sexes	75-84	156470	10.14 (10.19 - 10.1)	96.9 (96.8 - 96.9)	89.9 (89.7 - 90.1)	80.8 (80.6 - 81.0)	50.9 (50.6 - 51.2)	21.2 (20.9 - 21.5)	5.0 (4.8 - 5.2)
Musculoskeletal disorders	Osteoarthritis	Both sexes	85-95	37596	4.8 (4.86 - 4.74)	87.1 (86.7 - 87.4)	67.3 (66.8 - 67.8)	48.1 (47.6 - 48.7)	13.5 (13.1 - 14.0)	1.9 (1.7 - 2.1)	0.1 (0.1 - 0.2)
Musculoskeletal disorders	Rheumatoid arthritis	Both sexes	All	122790	19.77 (19.95 - 19.6)	96.3 (96.2 - 96.4)	91.0 (90.9 - 91.2)	85.8 (85.6 - 86.0)	72.8 (72.5 - 73.1)	60.5 (60.2 - 60.9)	49.5 (49.1 - 49.9)
Musculoskeletal disorders	Rheumatoid arthritis	Both sexes	16-24	4147	NR	100.0 (99.8 - 100.0)	99.8 (99.6 - 99.9)	99.7 (99.5 - 99.8)	99.4 (99.0 - 99.6)	99.0 (98.5 - 99.3)	98.7 (98.1 - 99.1)
Musculoskeletal disorders	Rheumatoid arthritis	Both sexes	25-34	6887	NR	99.9 (99.8 - 100.0)	99.9 (99.8 - 99.9)	99.7 (99.5 - 99.8)	99.2 (98.9 - 99.4)	98.5 (98.1 - 98.8)	97.3 (96.6 - 97.8)
Musculoskeletal disorders	Rheumatoid arthritis	Both sexes	35-44	10887	NR	99.8 (99.7 - 99.9)	99.6 (99.4 - 99.7)	99.1 (98.9 - 99.3)	97.9 (97.5 - 98.2)	95.8 (95.3 - 96.3)	93.2 (92.5 - 93.9)
Musculoskeletal disorders	Rheumatoid arthritis	Both sexes	45-54	17844	NR	99.5 (99.4 - 99.6)	98.6 (98.4 - 98.7)	97.7 (97.4 - 97.9)	94.6 (94.2 - 95.0)	90.3 (89.7 - 90.8)	83.0 (82.1 - 83.7)
Musculoskeletal disorders	Rheumatoid arthritis	Both sexes	55-64	27362	22.71	98.8 (98.7 - 99.0)	96.9 (96.7 - 97.1)	94.4 (94.1 - 94.7)	86.9 (86.4 - 87.3)	75.7 (75.1 - 76.3)	60.6 (59.7 - 61.4)
Musculoskeletal disorders	Rheumatoid arthritis	Both sexes	65-74	28197	13.83 (13.98 - 13.69)	97.0 (96.8 - 97.2)	91.8 (91.5 - 92.1)	86.0 (85.6 - 86.5)	67.4 (66.8 - 68.1)	44.4 (43.6 - 45.1)	21.9 (21.0 - 22.7)
Musculoskeletal disorders	Rheumatoid arthritis	Both sexes	75-84	21308	7.28 (7.39 - 7.18)	91.8 (91.4 - 92.2)	79.1 (78.6 - 79.7)	66.0 (65.3 - 66.6)	33.1 (32.4 - 33.9)	10.8 (10.2 - 11.4)	1.5 (1.2 - 1.9)
Musculoskeletal disorders	Rheumatoid arthritis	Both sexes	85-95	5997	2.98 (3.08 - 2.85)	76.3 (75.2 - 77.4)	49.7 (48.4 - 51.0)	30.3 (29.1 - 31.6)	6.6 (5.9 - 7.4)	0.7 (0.4 - 1.1)	0.0 (0.0 - 0.0)
Neurological disorders	Alzheimer's disease	Both sexes	All	133400	4.07 (4.1 - 4.04)	83.4 (83.2 - 83.6)	61.3 (61.0 - 61.5)	41.0 (40.7 - 41.3)	11.2 (11.0 - 11.4)	2.7 (2.6 - 2.9)	1.0 (0.9 - 1.1)
Neurological disorders	Alzheimer's disease	Both sexes	16-24	13	NR	100.0 (100.0 - 100.0)	100.0 (100.0 - 100.0)	87.5 (38.7 - 98.1)	87.5 (38.7 - 98.1)	87.5 (38.7 - 98.1)	87.5 (38.7 - 98.1)
Neurological disorders	Alzheimer's disease	Both sexes	25-34	26	NR	100.0 (100.0 - 100.0)	100.0 (100.0 - 100.0)	100.0 (100.0 - 100.0)	100.0 (100.0 - 100.0)	100.0 (100.0 - 100.0)	100.0 (100.0 - 100.0)
Neurological disorders	Alzheimer's disease	Both sexes	35-44	79	17.18	98.7 (91.4 - 99.8)	93.3 (84.6 - 97.2)	82.0 (70.9 - 89.1)	57.9 (44.1 - 69.4)	51.1 (37.2 - 63.5)	47.2 (32.4 - 60.6)
Neurological disorders	Alzheimer's disease	Both sexes	45-54	852	9.64 (10.49 - 8.9)	97.0 (95.6 - 98.0)	90.2 (87.9 - 92.1)	80.5 (77.4 - 83.2)	47.7 (43.4 - 51.8)	28.7 (24.5 - 33.0)	20.5 (16.3 - 25.1)
Neurological disorders	Alzheimer's disease	Both sexes	55-64	5558	8.64 (8.82 - 8.43)	97.0 (96.5 - 97.4)	90.7 (89.9 - 91.5)	78.7 (77.5 - 79.8)	39.9 (38.3 - 41.5)	16.1 (14.7 - 17.5)	8.5 (7.2 - 9.8)
Neurological disorders	Alzheimer's disease	Both sexes	65-74	25669	6.33 (6.41 - 6.26)	93.8 (93.5 - 94.1)	80.4 (79.8 - 80.9)	62.4 (61.8 - 63.1)	22.9 (22.2 - 23.5)	6.0 (5.6 - 6.5)	1.3 (1.0 - 1.7)
Neurological disorders	Alzheimer's disease	Both sexes	75-84	63756	4.19 (4.23 - 4.15)	85.6 (85.4 - 85.9)	63.4 (63.0 - 63.8)	41.3 (40.9 - 41.7)	8.9 (8.6 - 9.2)	1.2 (1.1 - 1.4)	0.2 (0.2 - 0.3)
Neurological disorders	Alzheimer's disease	Both sexes	85-95	36616	2.27 (2.31 - 2.23)	70.5 (70.0 - 70.9)	39.9 (39.4 - 40.4)	19.4 (19.0 - 19.9)	1.6 (1.4 - 1.7)	0.1 (0.1 - 0.2)	0.1 (0.0 - 0.1)
Neurological disorders	Alzheimer's disease and other dementias	Both sexes	All	374264	2.71 (2.72 - 2.69)	71.8 (71.7 - 72.0)	46.7 (46.6 - 46.9)	28.6 (28.5 - 28.8)	7.6 (7.5 - 7.7)	2.4 (2.4 - 2.5)	1.2 (1.1 - 1.3)
Neurological disorders	Alzheimer's disease and other dementias	Both sexes	16-24	158	NR	98.7 (95.0 - 99.7)	96.7 (92.3 - 98.6)	94.6 (89.4 - 97.2)	90.3 (83.8 - 94.3)	89.0 (81.8 - 93.4)	87.1 (78.8 - 92.4)
Neurological disorders	Alzheimer's disease and other dementias	Both sexes	25-34	315	NR	98.4 (96.2 - 99.3)	97.4 (94.8 - 98.7)	95.3 (92.1 - 97.2)	91.3 (87.3 - 94.1)	88.6 (83.7 - 92.1)	85.0 (78.2 - 89.8)
Neurological disorders	Alzheimer's disease and other dementias	Both sexes	35-44	626	NR	97.9 (96.4 - 98.8)	93.1 (90.8 - 94.9)	88.2 (85.3 - 90.6)	76.3 (72.4 - 79.8)	66.8 (62.2 - 71.0)	60.4 (55.0 - 65.4)
Neurological disorders	Alzheimer's disease and other dementias	Both sexes	45-54	2627	12.12 (13.15 - 11.52)	93.6 (92.6 - 94.5)	85.4 (84.0 - 86.7)	78.1 (76.4 - 79.7)	57.2 (55.0 - 59.3)	43.0 (40.6 - 45.4)	34.2 (31.5 - 37.0)
Neurological disorders	Alzheimer's disease and other dementias	Both sexes	55-64	12032	8.32 (8.48 - 8.21)	93.0 (92.5 - 93.5)	83.5 (82.8 - 84.2)	71.4 (70.5 - 72.2)	40.0 (39.0 - 41.1)	20.0 (19.0 - 21.0)	11.3 (10.4 - 12.4)
Neurological disorders	Alzheimer's disease and other dementias	Both sexes	65-74	52365	5.37 (5.43 - 5.31)	88.2 (87.9 - 88.5)	71.7 (71.3 - 72.1)	53.3 (52.9 - 53.8)	19.3 (18.9 - 19.8)	5.5 (5.2 - 5.8)	1.3 (1.1 - 1.6)
Neurological disorders	Alzheimer's disease and other dementias	Both sexes	75-84	157931	3.18 (3.2 - 3.16)	76.9 (76.7 - 77.2)	52.1 (51.8 - 52.3)	31.5 (31.3 - 31.8)	6.2 (6.0 - 6.3)	0.9 (0.8 - 0.9)	0.1 (0.1 - 0.2)
Neurological disorders	Alzheimer's disease and other dementias	Both sexes	85-95	141439	1.51 (1.53 - 1.49)	59.2 (58.9 - 59.4)	28.9 (28.7 - 29.2)	12.4 (12.2 - 12.6)	0.9 (0.8 - 0.9)	0.0 (0.0 - 0.1)	0.0 (0.0 - 0.0)
Neurological disorders	Dementia as a sign with others	Both sexes	All	119384	4.14 (4.17 - 4.11)	84.7 (84.5 - 84.9)	62.5 (62.2 - 62.8)	41.5 (41.2 - 41.8)	11.2 (11.0 - 11.5)	2.7 (2.5 - 2.8)	1.0 (0.9 - 1.1)
Neurological disorders	Dementia as a sign with others	Both sexes	16-24	14	NR	85.1 (52.3 - 96.1)	85.1 (52.3 - 96.1)	85.1 (52.3 - 96.1)	76.6 (43.3 - 91.9)	76.6 (43.3 - 91.9)	76.6 (43.3 - 91.9)
Neurological disorders	Dementia as a sign with others	Both sexes	25-34	27	NR	100.0 (100.0 - 100.0)	100.0 (100.0 - 100.0)	95.7 (72.9 - 99.4)	81.6 (58.0 - 92.7)	81.6 (58.0 - 92.7)	70.0 (37.3 - 87.9)
Neurological disorders	Dementia as a sign with others	Both sexes	35-44	101	8.98	94.0 (87.2 - 97.3)	85.6 (76.9 - 91.2)	68.9 (58.4 - 77.3)	44.4 (33.1 - 55.0)	42.7 (31.5 - 53.5)	38.8 (26.5 - 51.0)
Neurological disorders	Dementia as a sign with others	Both sexes	45-54	942	9.37 (10.07 - 8.67)	95.2 (93.6 - 96.4)	86.8 (84.4 - 88.9)	77.6 (74.6 - 80.3)	46.5 (42.6 - 50.4)	27.2 (23.3 - 31.2)	20.3 (16.2 - 24.7)
Neurological disorders	Dementia as a sign with others	Both sexes	55-64	5857	8.24 (8.43 - 8.06)	96.8 (96.3 - 97.2)	88.8 (87.9 - 89.6)	75.4 (74.1 - 76.5)	36.7 (35.2 - 38.2)	14.2 (12.9 - 15.5)	6.7 (5.6 - 8.0)
Neurological disorders	Dementia as a sign with others	Both sexes	65-74	24592	6.06 (6.13 - 5.98)	93.8 (93.5 - 94.1)	79.4 (78.9 - 80.0)	60.0 (59.4 - 60.7)	21.1 (20.5 - 21.8)	5.3 (4.9 - 5.8)	1.2 (0.9 - 1.5)
Neurological disorders	Dementia as a sign with others	Both sexes	75-84	56534	4.2 (4.24 - 4.16)	86.7 (86.4 - 86.9)	63.9 (63.4 - 64.3)	41.2 (40.8 - 41.7)	8.8 (8.5 - 9.1)	1.2 (1.0 - 1.3)	0.2 (0.2 - 0.4)
Neurological disorders	Dementia as a sign with others	Both sexes	85-95	30663	2.39 (2.42 - 2.34)	72.0 (71.5 - 72.5)	41.3 (40.8 - 41.9)	20.1 (19.6 - 20.6)	1.6 (1.5 - 1.8)	0.1 (0.0 - 0.1)	0.0 (0.0 - 0.1)
Neurological disorders	Idiopathic epilepsy	Both sexes	All	148081	14.1 (14.29 - 13.9)	86.5 (86.3 - 86.7)	76.4 (76.2 - 76.6)	69.3 (69.0 - 69.5)	57.1 (56.8 - 57.3)	48.7 (48.4 - 49.0)	42.7 (42.4 - 43.0)
Neurological disorders	Idiopathic epilepsy	Both sexes	16-24	14943	NR	99.2 (99.0 - 99.3)	98.1 (97.9 - 98.3)	97.4 (97.1 - 97.7)	95.6 (95.3 - 96.0)	94.1 (93.6 - 94.5)	92.4 (91.8 - 92.9)
Neurological disorders	Idiopathic epilepsy	Both sexes	25-34	14690	NR	98.4 (98.2 - 98.6)	96.7 (96.4 - 97.0)	95.1 (94.7 - 95.4)	92.1 (91.7 - 92.6)	89.8 (89.2 - 90.3)	87.4 (86.7 - 88.1)
Neurological disorders	Idiopathic epilepsy	Both sexes	35-44	14675	NR	96.9 (96.6 - 97.1)	93.7 (93.3 - 94.1)	91.7 (91.2 - 92.1)	86.6 (86.0 - 87.2)	82.0 (81.3 - 82.7)	77.2 (76.3 - 78.0)
Neurological disorders	Idiopathic epilepsy	Both sexes	45-54	18086	NR	93.1 (92.7 - 93.4)	87.0 (86.5 - 87.5)	83.1 (82.5 - 83.7)	74.8 (74.1 - 75.5)	66.7 (65.9 - 67.4)	57.5 (56.5 - 58.4)
Neurological disorders	Idiopathic epilepsy	Both sexes	55-64	22465	14.02 (14.29 - 13.69)	87.9 (87.5 - 88.4)	78.9 (78.3 - 79.4)	72.8 (72.2 - 73.4)	59.9 (59.2 - 60.6)	47.6 (46.8 - 48.4)	36.1 (35.2 - 37.0)
Neurological disorders	Idiopathic epilepsy	Both sexes	65-74	25903	7.87 (8.07 - 7.69)	83.1 (82.6 - 83.5)	71.0 (70.5 - 71.6)	61.8 (61.2 - 62.4)	41.8 (41.1 - 42.5)	24.3 (23.5 - 25.0)	11.8 (11.1 - 12.6)
Neurological disorders	Idiopathic epilepsy	Both sexes	75-84	25493	3.83 (3.9 - 3.74)	75.7 (75.1 - 76.2)	56.9 (56.2 - 57.5)	41.8 (41.2 - 42.5)	17.1 (16.6 - 17.7)	5.3 (4.9 - 5.7)	1.1 (0.9 - 1.4)
Neurological disorders	Idiopathic epilepsy	Both sexes	85-95	11487	1.67 (1.74 - 1.61)	60.9 (60.0 - 61.8)	33.9 (33.0 - 34.8)	17.9 (17.2 - 18.7)	2.8 (2.5 - 3.2)	0.3 (0.2 - 0.5)	0.0 (0.0 - 0.0)
Neurological disorders	Motor neuron disease	Both sexes	All	10196	1.62 (1.68 - 1.57)	63.4 (62.5 - 64.4)	34.8 (33.8 - 35.8)	26.3 (25.4 - 27.2)	18.4 (17.5 - 19.2)	14.1 (13.3 - 14.9)	11.3 (10.5 - 12.1)
Neurological disorders	Motor neuron disease	Both sexes	16-24	136	NR	97.1 (92.3 - 98.9)	97.1 (92.3 - 98.9)	94.5 (88.8 - 97.3)	89.2 (81.5 - 93.8)	82.5 (73.0 - 88.9)	82.5 (73.0 - 88.9)
Neurological disorders	Motor neuron disease	Both sexes	25-34	191	NR	97.3 (93.7 - 98.9)	91.7 (86.5 - 94.9)	88.0 (82.1 - 92.0)	81.5 (74.5 - 86.7)	76.7 (68.8 - 82.9)	70.7 (61.6 - 78.1)

Appendix Table A5: Unadjusted Median Survival and Survival Probabilities by Age, both sexes (Page 16 of 18)

Group	Diagnosis Category	Sex	Age	N	Median Survival (y)	1y (%)	3y (%)	5y (%)	10y (%)	15y (%)	20y (%)
Neurological disorders	Motor neuron disease	Both sexes	35-44	394	18.91	93.3 (90.4 - 95.4)	78.6 (74.0 - 82.4)	66.9 (61.8 - 71.5)	58.3 (53.0 - 63.3)	51.3 (45.6 - 56.8)	49.8 (43.8 - 55.5)
Neurological disorders	Motor neuron disease	Both sexes	45-54	946	4.43 (5.39 - 3.77)	87.3 (85.0 - 89.3)	59.5 (56.1 - 62.6)	47.5 (44.1 - 50.8)	37.9 (34.6 - 41.3)	34.2 (30.8 - 37.7)	27.5 (23.8 - 31.4)
Neurological disorders	Motor neuron disease	Both sexes	55-64	2167	2.13 (2.28 - 1.99)	75.7 (73.8 - 77.4)	40.1 (38.0 - 42.3)	29.8 (27.8 - 31.9)	21.5 (19.6 - 23.4)	16.6 (14.8 - 18.5)	12.0 (10.2 - 13.9)
Neurological disorders	Motor neuron disease	Both sexes	65-74	3254	1.39 (1.48 - 1.32)	61.0 (59.3 - 62.7)	27.8 (26.2 - 29.4)	19.5 (18.1 - 21.0)	11.4 (10.2 - 12.7)	6.4 (5.3 - 7.6)	3.2 (2.3 - 4.4)
Neurological disorders	Motor neuron disease	Both sexes	75-84	2558	0.84 (0.88 - 0.77)	44.8 (42.8 - 46.8)	19.7 (18.1 - 21.3)	13.1 (11.7 - 14.5)	5.4 (4.5 - 6.6)	1.3 (0.8 - 2.1)	0.3 (0.1 - 0.9)
Neurological disorders	Motor neuron disease	Both sexes	85-95	547	0.49 (0.6 - 0.42)	33.2 (29.3 - 37.2)	14.7 (11.8 - 18.0)	7.3 (5.2 - 9.8)	0.8 (0.2 - 2.1)	0.0 (0.0 - 0.0)	0.0 (0.0 - 0.0)
Neurological disorders	Multiple sclerosis	Both sexes	All	27843	NR	98.5 (98.4 - 98.6)	96.7 (96.4 - 96.9)	94.9 (94.6 - 95.1)	89.3 (88.9 - 89.7)	81.9 (81.3 - 82.4)	73.2 (72.4 - 73.9)
Neurological disorders	Multiple sclerosis	Both sexes	16-24	2130	NR	99.9 (99.6 - 100.0)	99.8 (99.4 - 99.9)	99.7 (99.3 - 99.9)	99.3 (98.8 - 99.6)	98.5 (97.6 - 99.1)	97.2 (95.6 - 98.2)
Neurological disorders	Multiple sclerosis	Both sexes	25-34	5933	NR	99.9 (99.8 - 100.0)	99.8 (99.6 - 99.9)	99.6 (99.3 - 99.7)	99.0 (98.7 - 99.3)	97.9 (97.4 - 98.4)	96.1 (95.3 - 96.9)
Neurological disorders	Multiple sclerosis	Both sexes	35-44	6478	NR	99.9 (99.8 - 99.9)	99.6 (99.4 - 99.7)	99.2 (98.9 - 99.4)	97.7 (97.2 - 98.1)	95.0 (94.3 - 95.6)	90.9 (89.8 - 91.9)
Neurological disorders	Multiple sclerosis	Both sexes	45-54	6026	NR	99.3 (99.0 - 99.5)	98.5 (98.2 - 98.8)	97.2 (96.7 - 97.6)	93.3 (92.6 - 94.0)	86.7 (85.7 - 87.7)	77.0 (75.5 - 78.5)
Neurological disorders	Multiple sclerosis	Both sexes	55-64	4254	21.14 (21.78 - 20.7)	98.2 (97.7 - 98.5)	95.5 (94.9 - 96.1)	93.1 (92.3 - 93.8)	84.1 (82.8 - 85.2)	69.7 (68.0 - 71.2)	54.1 (52.0 - 56.1)
Neurological disorders	Multiple sclerosis	Both sexes	65-74	2054	12.5 (12.98 - 11.9)	94.6 (93.5 - 95.5)	87.6 (86.1 - 89.0)	81.8 (80.0 - 83.4)	61.0 (58.7 - 63.3)	38.4 (35.8 - 41.0)	18.0 (15.5 - 20.6)
Neurological disorders	Multiple sclerosis	Both sexes	75-84	805	5.72 (6.34 - 5.18)	84.7 (82.0 - 87.0)	69.4 (66.0 - 72.5)	55.1 (51.5 - 58.6)	25.5 (22.3 - 28.9)	9.5 (7.2 - 12.2)	1.3 (0.4 - 3.4)
Neurological disorders	Multiple sclerosis	Both sexes	85-95	160	1.87 (2.92 - 1.52)	72.3 (64.7 - 78.6)	41.0 (33.2 - 48.7)	24.8 (18.0 - 32.1)	2.0 (0.4 - 6.1)	0.0 (0.0 - 0.0)	0.0 (0.0 - 0.0)
Neurological disorders	Parkinson's disease	Both sexes	All	65735	6.8 (6.87 - 6.73)	89.9 (89.7 - 90.1)	75.8 (75.5 - 76.2)	61.4 (61.0 - 61.8)	33.2 (32.7 - 33.6)	16.5 (16.1 - 16.9)	8.0 (7.6 - 8.4)
Neurological disorders	Parkinson's disease	Both sexes	16-24	64	NR	100.0 (100.0 - 100.0)	98.1 (87.6 - 99.7)	98.1 (87.6 - 99.7)	92.1 (76.7 - 97.5)	92.1 (76.7 - 97.5)	92.1 (76.7 - 97.5)
Neurological disorders	Parkinson's disease	Both sexes	25-34	148	NR	100.0 (100.0 - 100.0)	100.0 (100.0 - 100.0)	99.2 (94.2 - 99.9)	99.2 (94.2 - 99.9)	97.8 (90.9 - 99.5)	95.9 (87.3 - 98.7)
Neurological disorders	Parkinson's disease	Both sexes	35-44	600	NR	99.5 (98.4 - 99.8)	98.6 (97.3 - 99.3)	98.0 (96.5 - 98.9)	93.9 (91.4 - 95.8)	88.7 (85.1 - 91.5)	79.9 (74.0 - 84.7)
Neurological disorders	Parkinson's disease	Both sexes	45-54	2515	21.1 (21.82 - 20.44)	99.3 (98.8 - 99.5)	98.0 (97.3 - 98.5)	96.0 (95.1 - 96.8)	87.6 (86.0 - 89.0)	76.4 (74.1 - 78.6)	56.4 (52.9 - 59.8)
Neurological disorders	Parkinson's disease	Both sexes	55-64	8505	14.62 (14.85 - 14.33)	98.2 (97.9 - 98.4)	94.7 (94.2 - 95.2)	89.8 (89.1 - 90.5)	73.0 (71.9 - 74.1)	47.8 (46.3 - 49.2)	22.6 (21.0 - 24.2)
Neurological disorders	Parkinson's disease	Both sexes	65-74	21272	8.86 (8.99 - 8.76)	95.3 (95.0 - 95.6)	86.6 (86.1 - 87.1)	75.1 (74.4 - 75.7)	42.8 (42.0 - 43.6)	15.5 (14.8 - 16.3)	3.7 (3.2 - 4.3)
Neurological disorders	Parkinson's disease	Both sexes	75-84	25440	4.73 (4.79 - 4.66)	86.9 (86.5 - 87.4)	67.6 (67.0 - 68.2)	47.3 (46.6 - 48.0)	12.2 (11.7 - 12.7)	1.5 (1.3 - 1.8)	0.3 (0.2 - 0.4)
Neurological disorders	Parkinson's disease	Both sexes	85-95	7086	2.28 (2.37 - 2.21)	70.4 (69.3 - 71.5)	39.7 (38.5 - 40.9)	19.4 (18.4 - 20.4)	1.6 (1.2 - 2.0)	0.1 (0.0 - 0.3)	0.1 (0.0 - 0.3)
Neurological disorders	Unspecified dementia	Both sexes	All	226475	1.84 (1.86 - 1.83)	62.7 (62.5 - 62.9)	35.9 (35.7 - 36.1)	19.4 (19.2 - 19.5)	4.2 (4.1 - 4.3)	1.3 (1.2 - 1.3)	0.6 (0.6 - 0.7)
Neurological disorders	Unspecified dementia	Both sexes	16-24	36	NR	100.0 (100.0 - 100.0)	100.0 (100.0 - 100.0)	97.0 (80.4 - 99.6)	97.0 (80.4 - 99.6)	97.0 (80.4 - 99.6)	97.0 (80.4 - 99.6)
Neurological disorders	Unspecified dementia	Both sexes	25-34	61	22.36	98.3 (88.6 - 99.8)	96.6 (87.1 - 99.1)	96.6 (87.1 - 99.1)	90.1 (77.6 - 95.8)	86.6 (71.7 - 94.0)	77.9 (52.2 - 90.9)
Neurological disorders	Unspecified dementia	Both sexes	35-44	169	NR	96.4 (92.2 - 98.4)	92.0 (86.6 - 95.3)	88.0 (81.8 - 92.2)	75.7 (67.5 - 82.1)	63.1 (53.2 - 71.5)	60.2 (49.1 - 69.7)
Neurological disorders	Unspecified dementia	Both sexes	45-54	1015	11.78 (13.11 - 10.13)	89.7 (87.6 - 91.4)	80.3 (77.7 - 82.7)	72.0 (69.1 - 74.8)	53.9 (50.4 - 57.2)	43.0 (39.3 - 46.7)	36.6 (32.3 - 40.9)
Neurological disorders	Unspecified dementia	Both sexes	55-64	4642	6.95 (7.24 - 6.63)	87.4 (86.4 - 88.3)	74.6 (73.3 - 75.9)	61.3 (59.8 - 62.8)	35.0 (33.4 - 36.5)	19.2 (17.7 - 20.7)	12.1 (10.6 - 13.7)
Neurological disorders	Unspecified dementia	Both sexes	65-74	23018	4.01 (4.08 - 3.93)	80.0 (79.5 - 80.5)	59.8 (59.2 - 60.5)	41.0 (40.4 - 41.7)	13.0 (12.5 - 13.5)	3.8 (3.5 - 4.2)	1.0 (0.7 - 1.3)
Neurological disorders	Unspecified dementia	Both sexes	75-84	89514	2.32 (2.34 - 2.29)	68.4 (68.1 - 68.7)	41.9 (41.5 - 42.2)	23.0 (22.7 - 23.3)	3.8 (3.6 - 3.9)	0.5 (0.4 - 0.6)	0.1 (0.0 - 0.1)
Neurological disorders	Unspecified dementia	Both sexes	85-95	102208	1.21 (1.22 - 1.19)	53.9 (53.5 - 54.2)	24.2 (23.9 - 24.5)	9.5 (9.3 - 9.7)	0.6 (0.5 - 0.6)	0.0 (0.0 - 0.0)	0.0 (0.0 - 0.0)
Neurological disorders	Vascular dementia	Both sexes	All	78849	2.48 (2.51 - 2.45)	70.9 (70.6 - 71.2)	43.8 (43.5 - 44.2)	25.7 (25.3 - 26.0)	6.5 (6.3 - 6.7)	2.1 (2.0 - 2.3)	1.0 (0.8 - 1.1)
Neurological disorders	Vascular dementia	Both sexes	16-24	26	NR	100.0 (100.0 - 100.0)	96.0 (74.8 - 99.4)	96.0 (74.8 - 99.4)	92.0 (71.6 - 97.9)	85.4 (59.9 - 95.3)	85.4 (59.9 - 95.3)
Neurological disorders	Vascular dementia	Both sexes	25-34	48	NR	97.9 (86.1 - 99.7)	95.7 (84.0 - 98.9)	93.5 (81.1 - 97.8)	93.5 (81.1 - 97.8)	84.3 (64.0 - 93.7)	84.3 (64.0 - 93.7)
Neurological disorders	Vascular dementia	Both sexes	35-44	92	NR	98.9 (92.5 - 99.8)	96.6 (89.9 - 98.9)	92.9 (84.8 - 96.7)	76.0 (64.5 - 84.2)	63.5 (50.4 - 74.0)	58.4 (44.5 - 70.0)
Neurological disorders	Vascular dementia	Both sexes	45-54	393	14.32 (17.99 - 11.62)	92.3 (89.2 - 94.6)	82.8 (78.7 - 86.3)	77.4 (72.8 - 81.3)	60.1 (54.5 - 65.2)	48.2 (42.1 - 53.9)	38.2 (31.1 - 45.2)
Neurological disorders	Vascular dementia	Both sexes	55-64	2115	7.97 (8.35 - 7.65)	90.6 (89.3 - 91.8)	78.5 (76.7 - 80.3)	66.2 (64.0 - 68.3)	39.5 (37.1 - 41.9)	21.6 (19.3 - 24.0)	11.5 (9.2 - 14.1)
Neurological disorders	Vascular dementia	Both sexes	65-74	11108	4.52 (4.61 - 4.42)	84.4 (83.7 - 85.0)	64.0 (63.1 - 64.9)	45.7 (44.7 - 46.6)	16.3 (15.5 - 17.2)	4.7 (4.2 - 5.3)	1.1 (0.7 - 1.6)
Neurological disorders	Vascular dementia	Both sexes	75-84	36193	2.79 (2.83 - 2.75)	74.5 (74.0 - 74.9)	47.4 (46.9 - 48.0)	27.3 (26.8 - 27.8)	4.8 (4.5 - 5.0)	0.7 (0.6 - 0.8)	0.1 (0.0 - 0.2)
Neurological disorders	Vascular dementia	Both sexes	85-95	28058	1.53 (1.56 - 1.49)	59.8 (59.2 - 60.4)	28.2 (27.7 - 28.8)	11.6 (11.2 - 12.0)	0.8 (0.6 - 0.9)	0.1 (0.0 - 0.1)	0.0 (0.0 - 0.1)
Other	Decubitus ulcer	Both sexes	All	76832	1.98 (2.03 - 1.94)	60.2 (59.9 - 60.6)	41.9 (41.5 - 42.3)	30.6 (30.2 - 31.0)	16.7 (16.3 - 17.0)	10.7 (10.4 - 11.0)	7.6 (7.3 - 8.0)
Other	Decubitus ulcer	Both sexes	16-24	809	NR	96.5 (95.0 - 97.6)	92.9 (90.9 - 94.5)	91.0 (88.7 - 92.8)	86.5 (83.6 - 88.9)	83.6 (80.2 - 86.4)	78.9 (74.3 - 82.9)
Other	Decubitus ulcer	Both sexes	25-34	1106	NR	94.0 (92.4 - 95.3)	89.0 (87.0 - 90.8)	86.5 (84.2 - 88.5)	80.6 (77.8 - 83.2)	76.3 (72.9 - 79.3)	70.5 (66.3 - 74.4)
Other	Decubitus ulcer	Both sexes	35-44	1588	NR	91.8 (90.3 - 93.1)	86.8 (85.0 - 88.5)	83.1 (81.1 - 84.9)	75.0 (72.5 - 77.3)	64.4 (61.3 - 67.4)	55.3 (51.1 - 59.3)
Other	Decubitus ulcer	Both sexes	45-54	3070	11.63 (12.15 - 11.05)	83.9 (82.6 - 85.2)	76.6 (75.0 - 78.1)	69.4 (67.6 - 71.1)	55.1 (53.1 - 57.2)	40.9 (38.5 - 43.4)	30.4 (27.3 - 33.6)
Other	Decubitus ulcer	Both sexes	55-64	7140	5.96 (6.37 - 5.66)	76.1 (75.1 - 77.1)	63.4 (62.2 - 64.5)	54.0 (52.8 - 55.3)	36.4 (35.1 - 37.7)	23.9 (22.5 - 25.4)	15.3 (13.6 - 17.0)
Other	Decubitus ulcer	Both sexes	65-74	15726	3.13 (3.27 - 3.01)	67.1 (66.4 - 67.9)	50.9 (50.1 - 51.8)	39.7 (38.8 - 40.5)	20.6 (19.8 - 21.5)	10.5 (9.7 - 11.3)	4.8 (4.0 - 5.7)
Other	Decubitus ulcer	Both sexes	75-84	24581	1.64 (1.7 - 1.58)	57.6 (57.0 - 58.2)	37.0 (36.4 - 37.6)	23.6 (23.0 - 24.2)	7.1 (6.7 - 7.6)	1.7 (1.4 - 2.0)	0.2 (0.1 - 0.5)

Appendix Table A5: Unadjusted Median Survival and Survival Probabilities by Age, both sexes (Page 17 of 18)

Group	Diagnosis Category	Sex	Age	N	Median Survival (y)	1y (%)	3y (%)	5y (%)	10y (%)	15y (%)	20y (%)
Other	Decubitus ulcer	Both sexes	85-95	21094	0.76 (0.8 - 0.73)	45.7 (45.0 - 46.4)	22.6 (22.0 - 23.2)	10.3 (9.8 - 10.7)	1.1 (0.9 - 1.3)	0.1 (0.0 - 0.1)	0.0 (0.0 - 0.1)
Other	Endo/metab/blood/immune	Both sexes	All	1693901	20.64 (20.71 - 20.56)	93.6 (93.6 - 93.7)	88.2 (88.2 - 88.3)	83.4 (83.4 - 83.5)	72.1 (72.0 - 72.2)	61.1 (61.0 - 61.2)	51.1 (51.0 - 51.2)
Other	Endo/metab/blood/immune	Both sexes	16-24	74652	NR	99.7 (99.6 - 99.7)	99.3 (99.3 - 99.4)	99.1 (99.0 - 99.2)	98.5 (98.4 - 98.6)	97.8 (97.7 - 98.0)	97.0 (96.7 - 97.2)
Other	Endo/metab/blood/immune	Both sexes	25-34	187351	NR	99.7 (99.7 - 99.7)	99.4 (99.4 - 99.5)	99.2 (99.1 - 99.2)	98.6 (98.5 - 98.6)	97.6 (97.5 - 97.7)	96.2 (96.0 - 96.4)
Other	Endo/metab/blood/immune	Both sexes	35-44	183334	NR	99.3 (99.3 - 99.3)	98.6 (98.6 - 98.7)	98.1 (98.0 - 98.2)	96.6 (96.5 - 96.7)	94.4 (94.2 - 94.5)	91.0 (90.8 - 91.3)
Other	Endo/metab/blood/immune	Both sexes	45-54	218303	NR	98.2 (98.1 - 98.3)	96.6 (96.6 - 96.7)	95.4 (95.3 - 95.4)	91.7 (91.5 - 91.8)	86.6 (86.4 - 86.8)	79.3 (79.0 - 79.6)
Other	Endo/metab/blood/immune	Both sexes	55-64	303696	22.65 (22.78 - 22.55)	96.3 (96.2 - 96.4)	93.2 (93.1 - 93.3)	90.5 (90.4 - 90.6)	82.7 (82.6 - 82.9)	72.4 (72.2 - 72.6)	58.5 (58.2 - 58.8)
Other	Endo/metab/blood/immune	Both sexes	65-74	350388	14.03 (14.09 - 13.98)	92.9 (92.9 - 93.0)	87.0 (86.9 - 87.1)	81.5 (81.4 - 81.7)	65.7 (65.5 - 65.9)	45.8 (45.6 - 46.1)	24.9 (24.5 - 25.2)
Other	Endo/metab/blood/immune	Both sexes	75-84	273920	7.18 (7.22 - 7.15)	86.7 (86.6 - 86.9)	74.7 (74.5 - 74.8)	63.0 (62.8 - 63.2)	34.4 (34.2 - 34.6)	12.9 (12.7 - 13.1)	2.8 (2.7 - 3.0)
Other	Endo/metab/blood/immune	Both sexes	85-95	98550	2.86 (2.89 - 2.83)	71.7 (71.4 - 72.0)	48.5 (48.2 - 48.9)	30.4 (30.1 - 30.7)	6.7 (6.5 - 6.9)	0.7 (0.6 - 0.8)	0.1 (0.1 - 0.2)
Other	HIV/AIDS	Both sexes	All	43671	NR	96.6 (96.4 - 96.8)	93.5 (93.3 - 93.8)	91.1 (90.8 - 91.4)	86.4 (86.0 - 86.7)	82.1 (81.6 - 82.5)	76.5 (75.9 - 77.1)
Other	HIV/AIDS	Both sexes	16-24	7765	NR	99.6 (99.5 - 99.7)	99.2 (99.0 - 99.4)	98.8 (98.5 - 99.0)	98.2 (97.8 - 98.4)	97.4 (97.0 - 97.8)	96.4 (95.7 - 97.0)
Other	HIV/AIDS	Both sexes	25-34	10166	NR	99.6 (99.4 - 99.7)	99.2 (99.0 - 99.4)	98.8 (98.5 - 99.0)	97.7 (97.4 - 98.0)	96.8 (96.4 - 97.2)	95.3 (94.7 - 95.9)
Other	HIV/AIDS	Both sexes	35-44	7944	NR	99.0 (98.8 - 99.2)	98.1 (97.7 - 98.3)	97.3 (96.9 - 97.7)	95.5 (95.0 - 95.9)	93.5 (92.8 - 94.1)	90.1 (89.1 - 91.1)
Other	HIV/AIDS	Both sexes	45-54	6036	NR	98.1 (97.7 - 98.4)	95.8 (95.3 - 96.3)	93.9 (93.2 - 94.5)	89.4 (88.6 - 90.3)	84.0 (82.9 - 85.1)	76.6 (74.9 - 78.2)
Other	HIV/AIDS	Both sexes	55-64	5118	21.82 (22.65 - 21.13)	95.2 (94.6 - 95.8)	90.4 (89.6 - 91.2)	86.4 (85.3 - 87.3)	76.8 (75.5 - 78.1)	67.6 (66.0 - 69.2)	55.1 (52.9 - 57.2)
Other	HIV/AIDS	Both sexes	65-74	3992	12.17 (12.79 - 11.73)	90.2 (89.3 - 91.1)	80.6 (79.3 - 81.8)	72.6 (71.1 - 74.1)	56.8 (55.0 - 58.6)	40.9 (38.7 - 43.1)	23.0 (20.3 - 25.7)
Other	HIV/AIDS	Both sexes	75-84	2092	5.41 (5.85 - 5.05)	79.8 (78.0 - 81.5)	65.2 (63.0 - 67.3)	52.7 (50.3 - 55.0)	27.3 (25.0 - 29.7)	8.5 (6.8 - 10.6)	1.6 (0.6 - 3.4)
Other	HIV/AIDS	Both sexes	85-95	542	2.26 (2.52 - 1.99)	69.3 (65.1 - 73.0)	41.6 (37.3 - 45.9)	24.1 (20.3 - 28.1)	5.3 (3.2 - 8.0)	0.6 (0.1 - 2.6)	0.6 (0.1 - 2.6)
Other	Hemoglobinopathies	Both sexes	All	638150	5.09 (5.13 - 5.06)	75.7 (75.6 - 75.8)	60.4 (60.3 - 60.6)	50.4 (50.3 - 50.5)	34.9 (34.7 - 35.0)	26.2 (26.0 - 26.3)	20.9 (20.7 - 21.1)
Other	Hemoglobinopathies	Both sexes	16-24	15467	NR	98.5 (98.3 - 98.7)	97.2 (96.9 - 97.5)	96.6 (96.2 - 96.9)	95.5 (95.1 - 95.9)	94.7 (94.2 - 95.1)	93.8 (93.2 - 94.4)
Other	Hemoglobinopathies	Both sexes	25-34	30916	NR	98.2 (98.1 - 98.4)	97.0 (96.8 - 97.2)	96.3 (96.1 - 96.5)	94.8 (94.5 - 95.1)	93.6 (93.2 - 94.0)	91.9 (91.3 - 92.5)
Other	Hemoglobinopathies	Both sexes	35-44	35270	NR	95.6 (95.4 - 95.8)	93.0 (92.7 - 93.3)	91.5 (91.2 - 91.8)	88.5 (88.1 - 88.9)	85.5 (85.0 - 86.0)	81.8 (81.1 - 82.5)
Other	Hemoglobinopathies	Both sexes	45-54	44524	NR	89.1 (88.8 - 89.4)	83.6 (83.3 - 84.0)	80.2 (79.8 - 80.6)	73.6 (73.1 - 74.0)	67.2 (66.7 - 67.8)	61.3 (60.5 - 62.0)
Other	Hemoglobinopathies	Both sexes	55-64	71330	11.07 (11.29 - 10.89)	80.7 (80.5 - 81.0)	70.6 (70.3 - 70.9)	64.3 (63.9 - 64.7)	52.3 (51.9 - 52.7)	41.6 (41.1 - 42.1)	31.6 (31.0 - 32.2)
Other	Hemoglobinopathies	Both sexes	65-74	130680	6.1 (6.19 - 6.02)	76.5 (76.3 - 76.8)	63.3 (63.1 - 63.6)	54.4 (54.1 - 54.7)	36.6 (36.3 - 36.9)	22.1 (21.7 - 22.4)	10.6 (10.2 - 11.0)
Other	Hemoglobinopathies	Both sexes	75-84	180938	3.39 (3.42 - 3.36)	71.8 (71.6 - 72.0)	52.9 (52.6 - 53.1)	39.2 (38.9 - 39.4)	16.2 (16.0 - 16.4)	4.8 (4.6 - 5.0)	0.9 (0.8 - 1.0)
Other	Hemoglobinopathies	Both sexes	85-95	121839	1.65 (1.67 - 1.63)	60.4 (60.1 - 60.6)	33.8 (33.5 - 34.0)	18.0 (17.7 - 18.2)	2.7 (2.6 - 2.9)	0.2 (0.2 - 0.3)	0.0 (0.0 - 0.1)
Other	Psoriasis	Both sexes	All	233026	NR	98.3 (98.3 - 98.4)	95.5 (95.5 - 95.6)	92.7 (92.6 - 92.8)	85.0 (84.8 - 85.1)	76.6 (76.4 - 76.9)	68.0 (67.7 - 68.3)
Other	Psoriasis	Both sexes	16-24	16468	NR	100.0 (99.9 - 100.0)	99.9 (99.8 - 99.9)	99.7 (99.7 - 99.8)	99.4 (99.2 - 99.5)	98.9 (98.7 - 99.1)	98.5 (98.2 - 98.7)
Other	Psoriasis	Both sexes	25-34	26869	NR	99.9 (99.9 - 100.0)	99.8 (99.8 - 99.9)	99.7 (99.6 - 99.7)	99.2 (99.0 - 99.3)	98.6 (98.4 - 98.8)	97.8 (97.5 - 98.1)
Other	Psoriasis	Both sexes	35-44	33566	NR	99.9 (99.8 - 99.9)	99.6 (99.5 - 99.6)	99.2 (99.1 - 99.3)	98.1 (97.9 - 98.2)	96.5 (96.2 - 96.7)	93.9 (93.5 - 94.3)
Other	Psoriasis	Both sexes	45-54	42611	NR	99.6 (99.5 - 99.6)	98.8 (98.6 - 98.9)	97.9 (97.7 - 98.0)	95.0 (94.8 - 95.3)	90.6 (90.2 - 90.9)	83.9 (83.3 - 84.4)
Other	Psoriasis	Both sexes	55-64	49764	NR	99.1 (99.0 - 99.1)	97.1 (97.0 - 97.3)	95.0 (94.8 - 95.2)	88.3 (87.9 - 88.6)	78.9 (78.5 - 79.4)	65.3 (64.6 - 66.0)
Other	Psoriasis	Both sexes	65-74	39095	15.49 (15.64 - 15.34)	97.5 (97.4 - 97.7)	93.0 (92.7 - 93.2)	88.3 (88.0 - 88.7)	73.1 (72.6 - 73.6)	52.3 (51.6 - 53.0)	29.4 (28.5 - 30.4)
Other	Psoriasis	Both sexes	75-84	19719	8.47 (8.59 - 8.33)	93.4 (93.0 - 93.7)	82.8 (82.3 - 83.4)	70.9 (70.3 - 71.6)	40.4 (39.6 - 41.3)	16.1 (15.3 - 16.8)	3.9 (3.4 - 4.6)
Other	Psoriasis	Both sexes	85-95	4794	3.81 (3.96 - 3.66)	82.0 (80.9 - 83.1)	58.1 (56.7 - 59.5)	38.8 (37.3 - 40.3)	9.4 (8.4 - 10.4)	0.7 (0.4 - 1.2)	0.1 (0.0 - 0.4)
Other	Tuberculosis	Both sexes	All	40699	NR	94.4 (94.2 - 94.6)	90.0 (89.7 - 90.3)	86.3 (86.0 - 86.7)	78.7 (78.3 - 79.1)	72.0 (71.5 - 72.5)	65.0 (64.3 - 65.8)
Other	Tuberculosis	Both sexes	16-24	4850	NR	99.9 (99.7 - 99.9)	99.7 (99.5 - 99.8)	99.5 (99.3 - 99.7)	99.0 (98.7 - 99.3)	98.6 (98.2 - 99.0)	97.9 (97.0 - 98.5)
Other	Tuberculosis	Both sexes	25-34	7777	NR	99.7 (99.6 - 99.8)	99.5 (99.3 - 99.6)	99.3 (99.1 - 99.5)	98.9 (98.7 - 99.2)	98.3 (97.9 - 98.6)	97.1 (96.3 - 97.8)
Other	Tuberculosis	Both sexes	35-44	6818	NR	99.6 (99.4 - 99.7)	99.1 (98.8 - 99.3)	98.5 (98.2 - 98.8)	97.4 (97.0 - 97.8)	95.8 (95.2 - 96.4)	93.6 (92.5 - 94.5)
Other	Tuberculosis	Both sexes	45-54	5518	NR	98.2 (97.9 - 98.6)	96.8 (96.3 - 97.3)	95.4 (94.8 - 96.0)	92.3 (91.5 - 93.0)	88.4 (87.4 - 89.4)	81.8 (79.9 - 83.6)
Other	Tuberculosis	Both sexes	55-64	5003	22.73	96.6 (96.0 - 97.0)	93.1 (92.4 - 93.8)	89.5 (88.6 - 90.3)	81.1 (79.9 - 82.2)	70.7 (69.1 - 72.3)	57.5 (55.1 - 59.8)
Other	Tuberculosis	Both sexes	65-74	4627	11.89 (12.32 - 11.58)	90.8 (90.0 - 91.6)	83.6 (82.4 - 84.6)	76.2 (74.9 - 77.5)	57.8 (56.2 - 59.3)	38.7 (36.9 - 40.6)	21.1 (19.0 - 23.2)
Other	Tuberculosis	Both sexes	75-84	4356	5.07 (5.29 - 4.82)	79.8 (78.6 - 81.0)	63.4 (61.9 - 64.8)	50.6 (49.0 - 52.1)	24.6 (23.2 - 26.0)	8.1 (7.2 - 9.2)	1.5 (0.9 - 2.2)
Other	Tuberculosis	Both sexes	85-95	1698	1.94 (2.21 - 1.77)	62.7 (60.3 - 64.9)	39.6 (37.2 - 41.9)	23.9 (21.8 - 26.0)	4.3 (3.3 - 5.5)	0.6 (0.2 - 1.3)	0.0 (0.0 - 0.0)
Other	Urinary tract infections and interstitial nephritis	Both sexes	All	1189438	11.68 (11.75 - 11.62)	86.3 (86.2 - 86.3)	74.6 (74.6 - 74.7)	66.2 (66.1 - 66.3)	53.0 (52.9 - 53.1)	45.3 (45.1 - 45.4)	39.9 (39.8 - 40.0)
Other	Urinary tract infections and interstitial nephritis	Both sexes	16-24	105284	NR	99.9 (99.8 - 99.9)	99.7 (99.7 - 99.7)	99.6 (99.5 - 99.6)	99.2 (99.2 - 99.3)	98.9 (98.8 - 99.0)	98.5 (98.4 - 98.6)
Other	Urinary tract infections and interstitial nephritis	Both sexes	25-34	102994	NR	99.7 (99.7 - 99.8)	99.5 (99.4 - 99.5)	99.2 (99.2 - 99.3)	98.7 (98.6 - 98.8)	98.0 (97.9 - 98.1)	97.1 (96.9 - 97.3)
Other	Urinary tract infections and interstitial nephritis	Both sexes	35-44	80460	NR	99.1 (99.1 - 99.2)	98.4 (98.3 - 98.5)	97.8 (97.7 - 97.9)	96.3 (96.2 - 96.5)	94.4 (94.2 - 94.6)	92.0 (91.7 - 92.3)

Appendix Table A5: Unadjusted Median Survival and Survival Probabilities by Age, both sexes (Page 18 of 18)

Group	Diagnosis Category	Sex	Age	N	Median Survival (y)	1y (%)	3y (%)	5y (%)	10y (%)	15y (%)	20y (%)
Other	Urinary tract infections and interstitial nephritis	Both sexes	45-54	88058	NR	97.5 (97.4 - 97.6)	95.4 (95.3 - 95.6)	93.8 (93.7 - 94.0)	89.9 (89.7 - 90.1)	84.9 (84.6 - 85.2)	78.9 (78.4 - 79.3)
Other	Urinary tract infections and interstitial nephritis	Both sexes	55-64	124011	21.49 (21.75 - 21.27)	93.8 (93.7 - 94.0)	88.9 (88.8 - 89.1)	85.1 (84.9 - 85.3)	76.1 (75.9 - 76.4)	66.0 (65.6 - 66.3)	54.1 (53.6 - 54.6)
Other	Urinary tract infections and interstitial nephritis	Both sexes	65-74	195938	10.73 (10.81 - 10.66)	88.2 (88.1 - 88.3)	78.5 (78.3 - 78.6)	70.5 (70.3 - 70.7)	52.5 (52.3 - 52.8)	35.0 (34.7 - 35.3)	18.7 (18.3 - 19.1)
Other	Urinary tract infections and interstitial nephritis	Both sexes	75-84	279554	4.45 (4.48 - 4.42)	79.7 (79.6 - 79.9)	61.2 (61.0 - 61.3)	46.1 (45.9 - 46.3)	19.9 (19.7 - 20.1)	6.5 (6.3 - 6.6)	1.4 (1.3 - 1.5)
Other	Urinary tract infections and interstitial nephritis	Both sexes	85-95	201696	2.11 (2.12 - 2.09)	66.7 (66.5 - 66.9)	39.0 (38.7 - 39.2)	21.0 (20.9 - 21.2)	3.3 (3.2 - 3.4)	0.3 (0.3 - 0.4)	0.1 (0.0 - 0.1)

Appendix Table A6: Unadjusted Median Survival and Survival Probabilities by Age, males (Page 1 of 18)

Group	Diagnosis Category	Sex	Age	N	Median Survival (y)	1y (%)	3y (%)	5y (%)	10y (%)	15y (%)	20y (%)
Cancers	Bladder cancer	Male	All	53394	7.44 (7.55 - 7.3)	83.8 (83.5 - 84.1)	69.1 (68.7 - 69.5)	59.5 (59.0 - 59.9)	41.1 (40.6 - 41.6)	28.3 (27.8 - 28.8)	18.5 (17.9 - 19.1)
Cancers	Bladder cancer	Male	16-24	58	NR	100.0 (100.0 - 100.0)	98.2 (87.8 - 99.7)	98.2 (87.8 - 99.7)	98.2 (87.8 - 99.7)	98.2 (87.8 - 99.7)	98.2 (87.8 - 99.7)
Cancers	Bladder cancer	Male	25-34	212	NR	97.5 (94.2 - 99.0)	97.0 (93.5 - 98.7)	96.5 (92.7 - 98.3)	95.2 (91.0 - 97.5)	94.4 (89.7 - 97.0)	91.9 (83.6 - 96.1)
Cancers	Bladder cancer	Male	35-44	589	NR	96.6 (94.7 - 97.8)	92.6 (90.1 - 94.5)	90.3 (87.5 - 92.5)	88.1 (85.0 - 90.6)	86.7 (83.2 - 89.4)	83.2 (78.7 - 86.8)
Cancers	Bladder cancer	Male	45-54	2279	NR	94.3 (93.3 - 95.2)	89.0 (87.6 - 90.3)	85.3 (83.7 - 86.8)	79.3 (77.4 - 81.1)	74.0 (71.7 - 76.1)	66.3 (63.4 - 69.1)
Cancers	Bladder cancer	Male	55-64	7700	18.26 (18.92 - 17.74)	92.6 (91.9 - 93.1)	84.8 (83.9 - 85.6)	79.8 (78.8 - 80.7)	69.8 (68.6 - 70.9)	59.0 (57.7 - 60.4)	45.5 (43.8 - 47.3)
Cancers	Bladder cancer	Male	65-74	17225	10.47 (10.77 - 10.22)	88.7 (88.2 - 89.2)	77.4 (76.7 - 78.0)	69.6 (68.9 - 70.3)	51.6 (50.7 - 52.5)	33.8 (32.8 - 34.9)	16.3 (15.1 - 17.5)
Cancers	Bladder cancer	Male	75-84	18768	4.97 (5.08 - 4.87)	80.7 (80.1 - 81.2)	62.4 (61.7 - 63.1)	49.8 (49.0 - 50.6)	23.4 (22.6 - 24.1)	7.8 (7.2 - 8.4)	1.5 (1.2 - 1.9)
Cancers	Bladder cancer	Male	85-95	6433	1.87 (1.96 - 1.77)	64.4 (63.3 - 65.6)	37.4 (36.2 - 38.6)	21.1 (20.0 - 22.2)	3.8 (3.2 - 4.4)	0.2 (0.1 - 0.5)	0.1 (0.0 - 0.4)
Cancers	Brain cancer	Male	All	15339	1.05 (1.08 - 1.01)	51.1 (50.3 - 51.9)	31.2 (30.4 - 32.0)	26.1 (25.3 - 26.8)	19.8 (19.2 - 20.5)	16.5 (15.9 - 17.2)	13.9 (13.1 - 14.6)
Cancers	Brain cancer	Male	16-24	547	NR	91.5 (88.8 - 93.5)	82.8 (79.2 - 85.8)	79.3 (75.5 - 82.6)	72.4 (68.1 - 76.2)	70.0 (65.4 - 74.0)	67.7 (62.7 - 72.2)
Cancers	Brain cancer	Male	25-34	825	22.6	95.2 (93.5 - 96.5)	82.9 (80.0 - 85.3)	74.3 (71.0 - 77.3)	60.9 (57.0 - 64.5)	57.0 (53.0 - 60.8)	53.2 (48.8 - 57.4)
Cancers	Brain cancer	Male	35-44	1144	8.37 (10.03 - 6.63)	83.6 (81.3 - 85.6)	65.0 (62.1 - 67.8)	56.9 (53.8 - 59.8)	47.0 (43.8 - 50.2)	40.4 (37.1 - 43.8)	36.5 (32.9 - 40.1)
Cancers	Brain cancer	Male	45-54	2004	1.97 (2.15 - 1.78)	70.4 (68.3 - 72.3)	41.8 (39.5 - 44.0)	35.9 (33.7 - 38.0)	28.6 (26.5 - 30.8)	24.6 (22.4 - 26.8)	19.9 (17.5 - 22.3)
Cancers	Brain cancer	Male	55-64	3256	1.19 (1.26 - 1.14)	56.8 (55.1 - 58.5)	28.5 (27.0 - 30.2)	22.3 (20.8 - 23.8)	15.9 (14.6 - 17.3)	12.1 (10.8 - 13.5)	7.9 (6.6 - 9.5)
Cancers	Brain cancer	Male	65-74	3941	0.72 (0.76 - 0.69)	39.4 (37.8 - 40.9)	18.9 (17.6 - 20.2)	14.6 (13.4 - 15.7)	9.4 (8.4 - 10.5)	6.2 (5.2 - 7.2)	3.4 (2.4 - 4.7)
Cancers	Brain cancer	Male	75-84	2908	0.38 (0.4 - 0.36)	23.6 (22.1 - 25.2)	11.8 (10.6 - 13.1)	8.4 (7.3 - 9.5)	3.4 (2.6 - 4.3)	0.8 (0.4 - 1.4)	0.1 (0.0 - 0.7)
Cancers	Brain cancer	Male	85-95	702	0.2 (0.22 - 0.18)	13.1 (10.7 - 15.7)	5.0 (3.6 - 6.9)	2.5 (1.5 - 4.0)	0.0 (0.0 - 0.0)	0.0 (0.0 - 0.0)	0.0 (0.0 - 0.0)
Cancers	Breast cancer	Male	All	2975	9.72 (10.45 - 8.89)	82.7 (81.3 - 84.0)	70.5 (68.8 - 72.1)	61.5 (59.7 - 63.3)	49.2 (47.2 - 51.1)	37.9 (35.7 - 40.1)	29.5 (26.8 - 32.3)
Cancers	Breast cancer	Male	16-24	64	NR	96.9 (88.1 - 99.2)	96.9 (88.1 - 99.2)	96.9 (88.1 - 99.2)	92.0 (81.9 - 96.6)	92.0 (81.9 - 96.6)	92.0 (81.9 - 96.6)
Cancers	Breast cancer	Male	25-34	86	NR	93.0 (85.0 - 96.8)	91.8 (83.6 - 96.0)	91.8 (83.6 - 96.0)	86.7 (76.1 - 92.8)	86.7 (76.1 - 92.8)	86.7 (76.1 - 92.8)
Cancers	Breast cancer	Male	35-44	147	NR	95.2 (90.3 - 97.7)	88.3 (81.8 - 92.6)	84.7 (77.7 - 89.6)	81.5 (74.1 - 87.1)	81.5 (74.1 - 87.1)	75.8 (64.0 - 84.2)
Cancers	Breast cancer	Male	45-54	259	NR	84.4 (79.4 - 88.3)	77.1 (71.4 - 81.8)	73.2 (67.2 - 78.3)	67.2 (60.7 - 72.8)	60.8 (53.3 - 67.5)	56.4 (47.7 - 64.1)
Cancers	Breast cancer	Male	55-64	685	16.36 (19.06 - 14.11)	86.6 (83.8 - 89.0)	76.7 (73.3 - 79.8)	71.1 (67.4 - 74.4)	62.8 (58.8 - 66.4)	53.2 (48.8 - 57.5)	43.0 (37.0 - 48.9)
Cancers	Breast cancer	Male	65-74	933	9.44 (10.73 - 7.88)	82.7 (80.1 - 85.0)	71.4 (68.3 - 74.2)	62.7 (59.4 - 65.9)	48.3 (44.7 - 51.8)	30.9 (26.8 - 35.0)	14.1 (9.0 - 20.3)
Cancers	Breast cancer	Male	75-84	609	3.77 (4.32 - 3.2)	76.5 (72.9 - 79.7)	55.5 (51.3 - 59.6)	40.9 (36.6 - 45.1)	19.3 (15.6 - 23.3)	4.9 (2.8 - 7.8)	0.7 (0.1 - 3.2)
Cancers	Breast cancer	Male	85-95	188	2.62 (3.2 - 2.28)	67.5 (60.2 - 73.8)	45.6 (38.1 - 52.8)	18.2 (12.6 - 24.7)	1.5 (0.3 - 4.9)	0.0 (0.0 - 0.0)	0.0 (0.0 - 0.0)
Cancers	Colorectal cancer	Male	All	81075	5.0 (5.09 - 4.92)	77.6 (77.3 - 77.9)	60.0 (59.7 - 60.4)	50.0 (49.6 - 50.4)	34.8 (34.4 - 35.1)	24.1 (23.7 - 24.5)	16.3 (15.8 - 16.7)
Cancers	Colorectal cancer	Male	16-24	142	NR	91.3 (85.3 - 95.0)	86.7 (79.8 - 91.4)	85.0 (77.7 - 90.1)	82.8 (74.9 - 88.4)	82.8 (74.9 - 88.4)	79.6 (69.0 - 86.9)
Cancers	Colorectal cancer	Male	25-34	514	NR	88.3 (85.1 - 90.8)	75.1 (71.0 - 78.7)	72.1 (67.8 - 75.9)	68.7 (64.1 - 72.9)	67.3 (62.4 - 71.6)	65.0 (59.7 - 69.9)
Cancers	Colorectal cancer	Male	35-44	1689	NR	87.7 (86.1 - 89.2)	74.2 (72.0 - 76.3)	68.5 (66.1 - 70.8)	64.0 (61.5 - 66.5)	61.6 (58.9 - 64.2)	59.1 (56.0 - 62.1)
Cancers	Colorectal cancer	Male	45-54	5034	17.26 (19.72 - 15.74)	86.5 (85.5 - 87.4)	72.6 (71.3 - 73.8)	65.3 (63.9 - 66.7)	57.6 (56.1 - 59.2)	52.8 (51.1 - 54.5)	47.6 (45.5 - 49.7)
Cancers	Colorectal cancer	Male	55-64	13744	12.05 (12.55 - 11.43)	86.0 (85.4 - 86.6)	71.2 (70.4 - 72.0)	63.3 (62.5 - 64.2)	53.4 (52.5 - 54.4)	45.1 (44.0 - 46.1)	35.0 (33.6 - 36.4)
Cancers	Colorectal cancer	Male	65-74	25472	6.96 (7.22 - 6.72)	81.9 (81.4 - 82.3)	65.8 (65.2 - 66.4)	56.6 (55.9 - 57.2)	41.4 (40.7 - 42.2)	27.6 (26.9 - 28.4)	14.5 (13.6 - 15.5)
Cancers	Colorectal cancer	Male	75-84	25874	3.54 (3.62 - 3.44)	73.5 (72.9 - 74.0)	53.6 (53.0 - 54.3)	41.4 (40.8 - 42.1)	20.0 (19.4 - 20.6)	6.5 (6.0 - 7.0)	0.9 (0.6 - 1.2)
Cancers	Colorectal cancer	Male	85-95	8437	1.39 (1.46 - 1.31)	56.3 (55.3 - 57.4)	32.8 (31.7 - 33.8)	19.9 (18.9 - 20.8)	4.2 (3.6 - 4.8)	0.3 (0.1 - 0.5)	0.0 (0.0 - 0.2)
Cancers	Esophageal cancer	Male	All	11000	0.75 (0.78 - 0.73)	41.7 (40.8 - 42.6)	19.6 (18.8 - 20.4)	14.8 (14.1 - 15.6)	9.7 (9.1 - 10.4)	6.6 (6.0 - 7.3)	4.3 (3.6 - 5.1)
Cancers	Esophageal cancer	Male	16-24	12	1.64	66.7 (33.7 - 86.0)	38.9 (12.6 - 65.0)	38.9 (12.6 - 65.0)	38.9 (12.6 - 65.0)	38.9 (12.6 - 65.0)	38.9 (12.6 - 65.0)
Cancers	Esophageal cancer	Male	25-34	26	1.57 (16.68 - 0.57)	57.7 (36.8 - 73.9)	42.3 (23.5 - 60.0)	42.3 (23.5 - 60.0)	37.0 (18.7 - 55.5)	37.0 (18.7 - 55.5)	24.7 (6.3 - 49.3)
Cancers	Esophageal cancer	Male	35-44	129	1.22 (1.68 - 0.99)	57.6 (48.4 - 65.7)	29.6 (21.7 - 38.0)	26.7 (19.1 - 35.0)	24.3 (16.8 - 32.6)	22.3 (14.6 - 31.0)	22.3 (14.6 - 31.0)
Cancers	Esophageal cancer	Male	45-54	706	1.09 (1.31 - 0.93)	52.1 (48.4 - 55.8)	27.5 (24.1 - 30.9)	21.8 (18.7 - 25.1)	17.0 (14.1 - 20.1)	14.8 (12.0 - 18.0)	10.8 (7.3 - 15.0)
Cancers	Esophageal cancer	Male	55-64	2347	1.0 (1.09 - 0.93)	49.9 (47.9 - 52.0)	24.9 (23.1 - 26.7)	20.1 (18.4 - 21.8)	13.9 (12.4 - 15.5)	10.8 (9.4 - 12.4)	7.3 (5.7 - 9.1)
Cancers	Esophageal cancer	Male	65-74	3896	0.85 (0.89 - 0.81)	45.3 (43.7 - 46.9)	22.6 (21.2 - 24.0)	17.5 (16.2 - 18.8)	11.5 (10.3 - 12.7)	6.0 (4.9 - 7.3)	3.1 (1.9 - 4.7)
Cancers	Esophageal cancer	Male	75-84	3009	0.57 (0.61 - 0.54)	34.1 (32.4 - 35.8)	13.5 (12.3 - 14.8)	8.3 (7.3 - 9.5)	3.4 (2.6 - 4.4)	1.0 (0.5 - 1.8)	0.4 (0.1 - 1.3)
Cancers	Esophageal cancer	Male	85-95	860	0.3 (0.34 - 0.27)	18.3 (15.7 - 21.0)	3.8 (2.6 - 5.4)	1.4 (0.8 - 2.5)	0.2 (0.0 - 1.2)	0.2 (0.0 - 1.2)	0.0 (0.0 - 0.0)
Cancers	Gallbladder cancer	Male	All	7273	0.59 (0.62 - 0.56)	38.4 (37.3 - 39.5)	18.8 (17.9 - 19.8)	13.9 (13.0 - 14.8)	9.6 (8.8 - 10.4)	6.6 (5.8 - 7.4)	4.8 (3.9 - 5.8)
Cancers	Gallbladder cancer	Male	16-24	10	0.44 (3.49 - 0.02)	30.0 (7.1 - 57.8)	30.0 (7.1 - 57.8)	20.0 (3.1 - 47.5)	20.0 (3.1 - 47.5)	20.0 (3.1 - 47.5)	20.0 (3.1 - 47.5)
Cancers	Gallbladder cancer	Male	25-34	66	1.25 (3.61 - 0.58)	52.1 (39.3 - 63.4)	40.8 (28.7 - 52.5)	31.9 (20.2 - 44.2)	17.9 (7.6 - 31.7)	17.9 (7.6 - 31.7)	17.9 (7.6 - 31.7)
Cancers	Gallbladder cancer	Male	35-44	190	1.32 (1.58 - 1.05)	59.5 (52.1 - 66.2)	31.4 (24.7 - 38.3)	23.7 (17.6 - 30.4)	18.8 (13.1 - 25.4)	18.8 (13.1 - 25.4)	15.0 (8.0 - 24.2)
Cancers	Gallbladder cancer	Male	45-54	435	1.03 (1.17 - 0.89)	51.1 (46.2 - 55.7)	29.7 (25.2 - 34.2)	24.7 (20.5 - 29.2)	21.6 (17.4 - 26.2)	21.0 (16.7 - 25.6)	21.0 (16.7 - 25.6)

Appendix Table A6: Unadjusted Median Survival and Survival Probabilities by Age, males (Page 2 of 18)

Group	Diagnosis Category	Sex	Age	N	Median Survival (y)	1y (%)	3y (%)	5y (%)	10y (%)	15y (%)	20y (%)
Cancers	Gallbladder cancer	Male	55-64	1219	0.92 (1.04 - 0.78)	48.4 (45.6 - 51.2)	26.1 (23.6 - 28.6)	21.2 (18.8 - 23.6)	15.3 (13.1 - 17.7)	11.0 (8.8 - 13.4)	8.7 (6.1 - 11.9)
Cancers	Gallbladder cancer	Male	65-74	2262	0.71 (0.79 - 0.65)	42.4 (40.3 - 44.4)	22.1 (20.3 - 23.9)	16.5 (14.8 - 18.2)	12.0 (10.4 - 13.6)	7.0 (5.5 - 8.8)	3.5 (1.9 - 5.7)
Cancers	Gallbladder cancer	Male	75-84	2325	0.42 (0.46 - 0.38)	31.7 (29.8 - 33.7)	12.4 (11.0 - 13.9)	7.8 (6.6 - 9.1)	3.4 (2.5 - 4.5)	0.8 (0.3 - 1.9)	0.0 (0.0 - 0.0)
Cancers	Gallbladder cancer	Male	85-95	751	0.2 (0.24 - 0.18)	17.3 (14.7 - 20.1)	5.5 (4.0 - 7.4)	2.5 (1.5 - 4.0)	0.0 (0.0 - 0.0)	0.0 (0.0 - 0.0)	0.0 (0.0 - 0.0)
Cancers	Hodgkin lymphoma	Male	All	4543	20.12 (21.77 - 18.84)	87.6 (86.6 - 88.5)	79.6 (78.4 - 80.8)	74.1 (72.8 - 75.4)	64.6 (63.0 - 66.1)	57.7 (56.0 - 59.4)	50.1 (48.1 - 52.1)
Cancers	Hodgkin lymphoma	Male	16-24	486	NR	98.7 (97.2 - 99.4)	97.2 (95.2 - 98.4)	96.5 (94.3 - 97.8)	95.9 (93.6 - 97.4)	95.9 (93.6 - 97.4)	95.1 (92.0 - 97.0)
Cancers	Hodgkin lymphoma	Male	25-34	652	NR	98.3 (96.9 - 99.0)	96.8 (95.1 - 97.9)	95.7 (93.8 - 97.1)	93.9 (91.6 - 95.6)	93.6 (91.2 - 95.4)	91.4 (88.4 - 93.7)
Cancers	Hodgkin lymphoma	Male	35-44	582	NR	98.1 (96.5 - 98.9)	96.2 (94.2 - 97.5)	94.6 (92.3 - 96.2)	93.0 (90.4 - 94.9)	88.5 (84.9 - 91.3)	83.8 (79.2 - 87.5)
Cancers	Hodgkin lymphoma	Male	45-54	568	NR	93.6 (91.3 - 95.3)	89.0 (86.0 - 91.3)	85.6 (82.3 - 88.3)	78.4 (74.6 - 81.8)	70.1 (65.4 - 74.3)	59.5 (53.8 - 64.7)
Cancers	Hodgkin lymphoma	Male	55-64	682	13.96 (15.21 - 11.94)	89.3 (86.7 - 91.4)	81.9 (78.8 - 84.7)	76.0 (72.5 - 79.2)	59.5 (55.2 - 63.5)	46.6 (41.8 - 51.1)	28.6 (23.3 - 34.0)
Cancers	Hodgkin lymphoma	Male	65-74	822	7.45 (8.77 - 6.45)	81.2 (78.3 - 83.7)	67.9 (64.6 - 71.1)	60.0 (56.4 - 63.4)	40.7 (36.8 - 44.6)	26.4 (22.3 - 30.6)	8.4 (4.8 - 13.2)
Cancers	Hodgkin lymphoma	Male	75-84	588	2.68 (3.15 - 1.97)	67.9 (63.9 - 71.5)	47.8 (43.5 - 51.9)	32.7 (28.6 - 36.9)	13.7 (10.4 - 17.5)	4.5 (2.3 - 7.8)	0.9 (0.1 - 4.2)
Cancers	Hodgkin lymphoma	Male	85-95	158	0.89 (1.38 - 0.64)	48.5 (40.4 - 56.1)	27.3 (20.4 - 34.6)	10.3 (5.8 - 16.3)	2.8 (0.8 - 7.2)	0.0 (0.0 - 0.0)	0.0 (0.0 - 0.0)
Cancers	Kidney cancer	Male	All	24327	5.83 (6.05 - 5.64)	75.9 (75.4 - 76.5)	61.3 (60.6 - 61.9)	52.9 (52.3 - 53.6)	38.1 (37.4 - 38.9)	27.0 (26.3 - 27.8)	18.6 (17.7 - 19.6)
Cancers	Kidney cancer	Male	16-24	60	NR	93.3 (83.2 - 97.4)	86.4 (74.5 - 92.9)	84.4 (72.2 - 91.6)	82.4 (69.7 - 90.2)	76.0 (60.6 - 86.1)	72.0 (55.1 - 83.5)
Cancers	Kidney cancer	Male	25-34	171	NR	93.9 (88.9 - 96.7)	88.5 (82.3 - 92.6)	87.7 (81.3 - 92.0)	85.6 (78.6 - 90.5)	81.2 (72.4 - 87.4)	81.2 (72.4 - 87.4)
Cancers	Kidney cancer	Male	35-44	657	NR	92.3 (90.0 - 94.2)	86.9 (84.0 - 89.4)	84.3 (81.1 - 87.0)	77.6 (73.8 - 81.0)	74.1 (69.7 - 78.0)	69.6 (64.0 - 74.5)
Cancers	Kidney cancer	Male	45-54	2039	21.06	88.5 (87.0 - 89.8)	79.3 (77.4 - 81.0)	74.4 (72.3 - 76.3)	67.1 (64.7 - 69.3)	61.2 (58.5 - 63.9)	53.4 (49.8 - 56.9)
Cancers	Kidney cancer	Male	55-64	4742	12.27 (13.01 - 11.43)	83.6 (82.5 - 84.7)	72.1 (70.8 - 73.4)	66.2 (64.8 - 67.6)	54.9 (53.3 - 56.5)	44.4 (42.5 - 46.3)	32.5 (30.0 - 35.0)
Cancers	Kidney cancer	Male	65-74	7925	7.02 (7.34 - 6.63)	79.1 (78.1 - 79.9)	65.2 (64.1 - 66.2)	56.9 (55.7 - 58.0)	40.3 (39.1 - 41.6)	25.1 (23.6 - 26.5)	11.6 (9.9 - 13.3)
Cancers	Kidney cancer	Male	75-84	6846	3.02 (3.26 - 2.84)	68.6 (67.5 - 69.7)	50.2 (49.0 - 51.4)	38.8 (37.6 - 40.1)	18.2 (17.0 - 19.4)	5.1 (4.2 - 6.0)	0.4 (0.1 - 1.1)
Cancers	Kidney cancer	Male	85-95	1852	0.92 (1.08 - 0.83)	48.6 (46.3 - 50.9)	25.3 (23.3 - 27.4)	14.4 (12.7 - 16.1)	2.7 (1.9 - 3.8)	0.7 (0.3 - 1.5)	0.0 (0.0 - 0.0)
Cancers	Larynx cancer	Male	All	4483	6.21 (6.71 - 5.87)	82.6 (81.5 - 83.7)	65.4 (64.0 - 66.8)	55.3 (53.8 - 56.8)	37.3 (35.8 - 38.9)	25.4 (23.8 - 27.0)	16.0 (14.4 - 17.7)
Cancers	Larynx cancer	Male	16-24	12	NR	100.0 (100.0 - 100.0)	100.0 (100.0 - 100.0)	100.0 (100.0 - 100.0)	100.0 (100.0 - 100.0)	100.0 (100.0 - 100.0)	100.0 (100.0 - 100.0)
Cancers	Larynx cancer	Male	25-34	23	NR	100.0 (100.0 - 100.0)	91.3 (69.5 - 97.8)	91.3 (69.5 - 97.8)	91.3 (69.5 - 97.8)	91.3 (69.5 - 97.8)	83.0 (53.5 - 94.6)
Cancers	Larynx cancer	Male	35-44	75	NR	96.0 (88.0 - 98.7)	90.3 (80.7 - 95.3)	87.3 (76.9 - 93.2)	82.1 (70.4 - 89.4)	73.7 (60.3 - 83.2)	73.7 (60.3 - 83.2)
Cancers	Larynx cancer	Male	45-54	329	15.31 (16.85 - 12.89)	89.5 (85.6 - 92.3)	76.2 (71.1 - 80.5)	70.3 (65.0 - 75.0)	63.2 (57.5 - 68.4)	50.9 (44.5 - 56.9)	38.8 (31.8 - 45.7)
Cancers	Larynx cancer	Male	55-64	1095	11.1 (12.23 - 9.78)	88.8 (86.7 - 90.5)	73.9 (71.1 - 76.4)	67.2 (64.2 - 70.0)	53.0 (49.8 - 56.1)	39.6 (36.2 - 43.0)	25.6 (21.8 - 29.6)
Cancers	Larynx cancer	Male	65-74	1512	6.74 (7.43 - 5.93)	84.2 (82.3 - 86.0)	67.6 (65.1 - 69.9)	56.9 (54.2 - 59.4)	37.5 (34.7 - 40.2)	21.9 (19.2 - 24.7)	10.3 (7.8 - 13.1)
Cancers	Larynx cancer	Male	75-84	1134	3.8 (4.19 - 3.38)	76.4 (73.7 - 78.7)	55.8 (52.7 - 58.7)	42.4 (39.3 - 45.4)	16.4 (13.9 - 19.1)	5.8 (4.1 - 8.0)	0.9 (0.2 - 2.7)
Cancers	Larynx cancer	Male	85-95	298	1.98 (2.43 - 1.47)	63.6 (57.9 - 68.8)	38.4 (32.7 - 44.0)	21.7 (16.9 - 26.8)	1.8 (0.6 - 4.3)	0.0 (0.0 - 0.0)	0.0 (0.0 - 0.0)
Cancers	Leukemia	Male	All	25686	5.11 (5.28 - 4.93)	74.7 (74.1 - 75.2)	59.5 (58.9 - 60.2)	50.4 (49.7 - 51.0)	35.1 (34.4 - 35.7)	25.5 (24.8 - 26.2)	19.8 (19.0 - 20.6)
Cancers	Leukemia	Male	16-24	497	NR	92.6 (89.9 - 94.6)	83.9 (80.3 - 86.9)	80.4 (76.5 - 83.7)	79.0 (75.0 - 82.5)	77.1 (72.7 - 80.8)	76.6 (72.2 - 80.4)
Cancers	Leukemia	Male	25-34	579	NR	92.3 (89.8 - 94.2)	83.2 (79.8 - 86.0)	81.3 (77.7 - 84.3)	80.3 (76.6 - 83.4)	78.2 (74.3 - 81.6)	77.7 (73.6 - 81.2)
Cancers	Leukemia	Male	35-44	936	NR	92.2 (90.2 - 93.7)	83.2 (80.5 - 85.5)	80.4 (77.6 - 82.9)	73.6 (70.3 - 76.5)	69.6 (65.9 - 73.0)	65.1 (60.5 - 69.3)
Cancers	Leukemia	Male	45-54	1989	21.76 (22.84 - 19.64)	90.3 (88.9 - 91.6)	81.6 (79.8 - 83.3)	77.9 (76.0 - 79.8)	70.2 (67.9 - 72.3)	62.3 (59.5 - 64.9)	51.7 (48.1 - 55.2)
Cancers	Leukemia	Male	55-64	4425	12.24 (12.78 - 11.5)	86.8 (85.8 - 87.8)	76.6 (75.3 - 77.9)	69.9 (68.4 - 71.3)	56.1 (54.5 - 57.8)	42.6 (40.7 - 44.5)	31.9 (29.6 - 34.2)
Cancers	Leukemia	Male	65-74	7407	6.21 (6.5 - 5.88)	78.6 (77.7 - 79.6)	64.6 (63.4 - 65.7)	54.9 (53.7 - 56.1)	34.7 (33.4 - 36.0)	19.2 (17.9 - 20.6)	9.4 (8.1 - 10.8)
Cancers	Leukemia	Male	75-84	7293	2.31 (2.42 - 2.16)	64.3 (63.1 - 65.4)	44.2 (43.0 - 45.4)	31.2 (30.1 - 32.4)	10.8 (9.9 - 11.7)	2.6 (2.1 - 3.3)	0.7 (0.4 - 1.3)
Cancers	Leukemia	Male	85-95	2503	0.79 (0.89 - 0.69)	45.9 (43.9 - 47.9)	22.4 (20.7 - 24.1)	10.4 (9.2 - 11.8)	1.1 (0.7 - 1.7)	0.0 (0.0 - 0.0)	0.0 (0.0 - 0.0)
Cancers	Lip oral cavity cancer	Male	All	15309	7.67 (7.95 - 7.39)	83.6 (83.0 - 84.1)	68.9 (68.1 - 69.6)	59.3 (58.5 - 60.1)	43.3 (42.4 - 44.2)	31.6 (30.6 - 32.6)	22.8 (21.7 - 23.9)
Cancers	Lip oral cavity cancer	Male	16-24	104	NR	94.2 (87.5 - 97.3)	91.2 (83.8 - 95.3)	91.2 (83.8 - 95.3)	89.0 (81.0 - 93.8)	89.0 (81.0 - 93.8)	89.0 (81.0 - 93.8)
Cancers	Lip oral cavity cancer	Male	25-34	273	NR	95.9 (92.7 - 97.7)	88.3 (83.6 - 91.6)	87.4 (82.6 - 90.9)	83.4 (78.0 - 87.6)	80.8 (74.9 - 85.5)	77.7 (70.9 - 83.1)
Cancers	Lip oral cavity cancer	Male	35-44	516	NR	93.3 (90.8 - 95.2)	86.8 (83.5 - 89.5)	83.5 (79.8 - 86.5)	78.7 (74.6 - 82.2)	73.8 (69.2 - 77.8)	69.0 (63.3 - 74.0)
Cancers	Lip oral cavity cancer	Male	45-54	1510	20.98 (22.04 - 18.92)	91.6 (90.1 - 92.9)	82.0 (79.9 - 83.9)	76.7 (74.4 - 78.9)	69.1 (66.5 - 71.6)	62.6 (59.6 - 65.4)	51.7 (47.6 - 55.5)
Cancers	Lip oral cavity cancer	Male	55-64	3326	12.91 (13.71 - 11.94)	88.3 (87.2 - 89.4)	75.7 (74.1 - 77.1)	68.8 (67.1 - 70.4)	56.1 (54.1 - 57.9)	45.2 (43.0 - 47.3)	34.2 (31.5 - 36.8)
Cancers	Lip oral cavity cancer	Male	65-74	4438	8.18 (8.67 - 7.69)	84.4 (83.3 - 85.4)	69.5 (68.0 - 70.8)	60.2 (58.7 - 61.8)	43.9 (42.2 - 45.6)	26.3 (24.4 - 28.3)	11.4 (9.3 - 13.8)
Cancers	Lip oral cavity cancer	Male	75-84	3725	4.79 (5.07 - 4.52)	79.4 (78.0 - 80.6)	62.1 (60.4 - 63.7)	48.6 (46.8 - 50.3)	22.9 (21.3 - 24.7)	6.8 (5.6 - 8.2)	0.6 (0.2 - 1.4)
Cancers	Lip oral cavity cancer	Male	85-95	1381	2.34 (2.57 - 2.12)	65.8 (63.2 - 68.3)	42.5 (39.7 - 45.2)	24.5 (22.1 - 27.0)	4.2 (3.0 - 5.7)	0.3 (0.1 - 1.0)	0.0 (0.0 - 0.0)
Cancers	Liver cancer	Male	All	10537	0.72 (0.76 - 0.68)	43.6 (42.7 - 44.6)	23.5 (22.6 - 24.4)	16.5 (15.7 - 17.2)	10.6 (9.9 - 11.4)	7.0 (6.2 - 7.8)	5.3 (4.4 - 6.3)

Appendix Table A6: Unadjusted Median Survival and Survival Probabilities by Age, males (Page 3 of 18)

Group	Diagnosis Category	Sex	Age	N	Median Survival (y)	1y (%)	3y (%)	5y (%)	10y (%)	15y (%)	20y (%)
Cancers	Liver cancer	Male	16-24	36	3.71	66.7 (48.8 - 79.5)	51.5 (33.9 - 66.5)	45.5 (28.5 - 61.0)	42.2 (25.6 - 57.9)	37.5 (20.9 - 54.1)	37.5 (20.9 - 54.1)
Cancers	Liver cancer	Male	25-34	87	2.74 (5.82 - 1.44)	66.6 (55.4 - 75.6)	48.1 (36.7 - 58.6)	40.4 (29.2 - 51.2)	34.8 (23.8 - 46.0)	34.8 (23.8 - 46.0)	34.8 (23.8 - 46.0)
Cancers	Liver cancer	Male	35-44	213	1.14 (1.51 - 0.92)	54.1 (47.2 - 60.6)	32.6 (26.3 - 39.1)	29.9 (23.7 - 36.3)	25.9 (19.9 - 32.3)	24.7 (18.6 - 31.3)	24.7 (18.6 - 31.3)
Cancers	Liver cancer	Male	45-54	782	0.96 (1.17 - 0.8)	49.0 (45.5 - 52.5)	33.2 (29.8 - 36.6)	26.9 (23.7 - 30.3)	24.1 (20.8 - 27.4)	17.2 (13.6 - 21.2)	13.3 (9.0 - 18.3)
Cancers	Liver cancer	Male	55-64	2396	0.97 (1.07 - 0.88)	49.4 (47.4 - 51.4)	29.1 (27.3 - 31.0)	21.9 (20.1 - 23.6)	14.9 (13.2 - 16.7)	11.0 (9.1 - 13.0)	8.1 (5.8 - 11.0)
Cancers	Liver cancer	Male	65-74	3614	0.82 (0.89 - 0.75)	45.8 (44.1 - 47.4)	24.8 (23.3 - 26.3)	16.7 (15.3 - 18.1)	9.3 (8.0 - 10.6)	3.4 (2.3 - 4.8)	0.0 (0.0 - 0.0)
Cancers	Liver cancer	Male	75-84	2814	0.52 (0.59 - 0.46)	36.7 (34.8 - 38.5)	15.2 (13.8 - 16.7)	7.8 (6.7 - 9.0)	2.2 (1.5 - 3.2)	0.7 (0.3 - 1.8)	0.0 (0.0 - 0.0)
Cancers	Liver cancer	Male	85-95	591	0.24 (0.28 - 0.2)	24.6 (21.1 - 28.2)	8.2 (6.0 - 10.9)	5.2 (3.3 - 7.7)	1.1 (0.2 - 4.2)	0.0 (0.0 - 0.0)	0.0 (0.0 - 0.0)
Cancers	Lung cancer	Male	All	54008	0.6 (0.62 - 0.59)	38.7 (38.2 - 39.1)	20.1 (19.8 - 20.5)	14.6 (14.3 - 14.9)	8.6 (8.4 - 8.9)	5.5 (5.2 - 5.8)	3.9 (3.6 - 4.2)
Cancers	Lung cancer	Male	16-24	61	NR	73.7 (60.8 - 83.0)	66.6 (53.1 - 77.0)	66.6 (53.1 - 77.0)	62.7 (48.9 - 73.7)	62.7 (48.9 - 73.7)	62.7 (48.9 - 73.7)
Cancers	Lung cancer	Male	25-34	162	NR	81.4 (74.4 - 86.7)	64.7 (56.4 - 71.8)	60.6 (52.1 - 68.1)	57.8 (49.1 - 65.5)	54.3 (45.2 - 62.4)	50.9 (40.1 - 60.6)
Cancers	Lung cancer	Male	35-44	512	2.23 (2.68 - 1.78)	66.7 (62.4 - 70.7)	43.6 (39.1 - 47.9)	38.5 (34.1 - 42.9)	34.3 (30.0 - 38.7)	32.4 (28.0 - 36.8)	31.2 (26.7 - 35.8)
Cancers	Lung cancer	Male	45-54	2277	1.04 (1.15 - 0.96)	51.1 (49.0 - 53.1)	30.8 (28.9 - 32.8)	25.7 (23.8 - 27.6)	20.8 (19.0 - 22.6)	17.0 (15.2 - 18.9)	15.1 (13.2 - 17.0)
Cancers	Lung cancer	Male	55-64	9253	0.84 (0.87 - 0.81)	45.7 (44.7 - 46.7)	25.2 (24.3 - 26.1)	19.7 (18.8 - 20.5)	13.7 (12.9 - 14.5)	9.9 (9.2 - 10.7)	7.2 (6.4 - 8.1)
Cancers	Lung cancer	Male	65-74	19404	0.69 (0.71 - 0.68)	41.7 (40.9 - 42.4)	22.3 (21.7 - 22.9)	16.2 (15.6 - 16.8)	9.1 (8.7 - 9.6)	4.6 (4.2 - 5.1)	1.8 (1.4 - 2.4)
Cancers	Lung cancer	Male	75-84	17883	0.47 (0.49 - 0.46)	33.9 (33.2 - 34.6)	15.7 (15.2 - 16.3)	10.0 (9.5 - 10.5)	3.5 (3.1 - 3.8)	0.9 (0.6 - 1.2)	0.2 (0.1 - 0.6)
Cancers	Lung cancer	Male	85-95	4383	0.2 (0.21 - 0.19)	18.2 (17.1 - 19.4)	6.4 (5.7 - 7.2)	2.9 (2.4 - 3.5)	0.1 (0.0 - 0.4)	0.0 (0.0 - 0.0)	0.0 (0.0 - 0.0)
Cancers	Lymphoma	Male	All	28253	7.45 (7.64 - 7.23)	79.2 (78.7 - 79.7)	66.4 (65.8 - 66.9)	58.1 (57.5 - 58.7)	43.0 (42.3 - 43.6)	32.7 (31.9 - 33.4)	25.8 (25.0 - 26.6)
Cancers	Lymphoma	Male	16-24	460	NR	95.0 (92.5 - 96.6)	92.2 (89.3 - 94.3)	91.2 (88.1 - 93.5)	89.8 (86.5 - 92.3)	88.9 (85.4 - 91.6)	88.9 (85.4 - 91.6)
Cancers	Lymphoma	Male	25-34	755	NR	95.9 (94.3 - 97.1)	92.9 (90.8 - 94.6)	92.0 (89.7 - 93.8)	90.1 (87.5 - 92.1)	88.4 (85.4 - 90.7)	86.7 (83.4 - 89.5)
Cancers	Lymphoma	Male	35-44	1404	NR	93.1 (91.7 - 94.3)	88.9 (87.1 - 90.5)	86.2 (84.3 - 88.0)	82.6 (80.3 - 84.6)	79.5 (76.9 - 81.8)	76.1 (72.8 - 79.0)
Cancers	Lymphoma	Male	45-54	2578	NR	91.5 (90.4 - 92.6)	85.5 (84.0 - 86.8)	82.1 (80.5 - 83.5)	74.8 (72.9 - 76.6)	67.8 (65.5 - 69.9)	61.2 (58.3 - 63.9)
Cancers	Lymphoma	Male	55-64	5209	14.15 (14.87 - 13.61)	88.4 (87.5 - 89.2)	78.7 (77.6 - 79.9)	73.2 (71.9 - 74.4)	60.0 (58.5 - 61.5)	48.0 (46.3 - 49.7)	35.7 (33.6 - 37.8)
Cancers	Lymphoma	Male	65-74	8041	7.43 (7.74 - 7.13)	81.2 (80.3 - 82.0)	67.9 (66.9 - 69.0)	59.3 (58.2 - 60.4)	40.5 (39.2 - 41.7)	23.2 (21.8 - 24.6)	11.6 (10.2 - 13.1)
Cancers	Lymphoma	Male	75-84	7536	3.3 (3.46 - 3.12)	70.2 (69.1 - 71.2)	52.2 (51.0 - 53.3)	38.8 (37.6 - 40.0)	15.7 (14.6 - 16.8)	4.7 (3.9 - 5.5)	1.0 (0.6 - 1.7)
Cancers	Lymphoma	Male	85-95	2229	1.0 (1.09 - 0.87)	49.8 (47.7 - 51.9)	27.8 (25.8 - 29.8)	14.9 (13.3 - 16.6)	2.4 (1.7 - 3.4)	0.0 (0.0 - 0.0)	0.0 (0.0 - 0.0)
Cancers	Malignant neoplasm of bone & articular cartilage	Male	All	4343	2.45 (2.74 - 2.16)	62.6 (61.1 - 64.0)	47.0 (45.5 - 48.5)	40.4 (38.9 - 41.9)	31.5 (30.0 - 33.0)	25.4 (23.9 - 27.0)	21.3 (19.7 - 22.9)
Cancers	Malignant neoplasm of bone & articular cartilage	Male	16-24	265	NR	93.1 (89.2 - 95.6)	76.7 (71.0 - 81.4)	69.8 (63.6 - 75.1)	65.9 (59.5 - 71.6)	64.2 (57.5 - 70.1)	63.2 (56.3 - 69.3)
Cancers	Malignant neoplasm of bone & articular cartilage	Male	25-34	228	NR	89.2 (84.4 - 92.7)	77.9 (71.7 - 82.9)	75.3 (69.0 - 80.6)	71.9 (65.3 - 77.5)	71.9 (65.3 - 77.5)	69.5 (62.2 - 75.7)
Cancers	Malignant neoplasm of bone & articular cartilage	Male	35-44	272	NR	83.9 (79.0 - 87.8)	73.0 (67.2 - 78.0)	69.2 (63.1 - 74.4)	65.0 (58.7 - 70.5)	60.0 (53.2 - 66.2)	55.6 (47.9 - 62.6)
Cancers	Malignant neoplasm of bone & articular cartilage	Male	45-54	407	10.28 (18.39 - 7.94)	80.1 (75.8 - 83.7)	68.5 (63.7 - 72.9)	61.4 (56.4 - 66.1)	50.2 (44.9 - 55.3)	46.0 (40.5 - 51.4)	44.0 (38.2 - 49.5)
Cancers	Malignant neoplasm of bone & articular cartilage	Male	55-64	799	3.42 (4.31 - 2.56)	66.2 (62.8 - 69.4)	51.3 (47.8 - 54.8)	43.9 (40.4 - 47.4)	36.4 (32.9 - 39.9)	28.2 (24.6 - 31.8)	19.9 (16.2 - 23.9)
Cancers	Malignant neoplasm of bone & articular cartilage	Male	65-74	1101	1.79 (2.29 - 1.34)	58.0 (55.0 - 60.8)	41.8 (38.8 - 44.8)	35.1 (32.2 - 38.0)	23.7 (20.9 - 26.5)	13.6 (11.1 - 16.4)	5.8 (3.3 - 9.3)
Cancers	Malignant neoplasm of bone & articular cartilage	Male	75-84	959	0.82 (0.96 - 0.65)	45.7 (42.5 - 48.8)	27.3 (24.4 - 30.2)	19.6 (17.0 - 22.3)	8.0 (6.1 - 10.2)	2.1 (1.1 - 3.6)	0.3 (0.0 - 1.4)
Cancers	Malignant neoplasm of bone & articular cartilage	Male	85-95	304	0.45 (0.64 - 0.35)	35.2 (29.8 - 40.6)	15.1 (11.2 - 19.6)	9.0 (5.9 - 12.9)	0.8 (0.1 - 3.5)	0.0 (0.0 - 0.0)	0.0 (0.0 - 0.0)
Cancers	Melanoma	Male	All	47427	14.26 (14.58 - 13.99)	91.9 (91.7 - 92.2)	81.7 (81.3 - 82.1)	74.3 (73.9 - 74.7)	60.0 (59.4 - 60.5)	48.4 (47.7 - 49.1)	39.6 (38.8 - 40.5)
Cancers	Melanoma	Male	16-24	335	NR	97.3 (94.8 - 98.6)	93.8 (90.5 - 96.0)	91.7 (88.1 - 94.3)	90.5 (86.6 - 93.3)	90.0 (85.9 - 92.9)	90.0 (85.9 - 92.9)
Cancers	Melanoma	Male	25-34	1255	NR	97.6 (96.5 - 98.3)	94.2 (92.7 - 95.4)	92.2 (90.4 - 93.6)	89.4 (87.3 - 91.1)	87.9 (85.6 - 89.9)	87.0 (84.4 - 89.2)
Cancers	Melanoma	Male	35-44	2973	NR	97.4 (96.8 - 97.9)	94.6 (93.7 - 95.3)	91.9 (90.8 - 92.9)	88.5 (87.1 - 89.7)	85.2 (83.6 - 86.7)	82.7 (80.5 - 84.6)
Cancers	Melanoma	Male	45-54	5351	NR	96.3 (95.7 - 96.8)	92.3 (91.5 - 93.0)	89.3 (88.4 - 90.2)	83.6 (82.4 - 84.7)	80.1 (78.6 - 81.5)	75.8 (73.7 - 77.7)
Cancers	Melanoma	Male	55-64	9277	22.61	94.9 (94.4 - 95.3)	88.9 (88.3 - 89.6)	84.6 (83.8 - 85.4)	76.4 (75.4 - 77.4)	67.9 (66.6 - 69.2)	56.5 (54.4 - 58.4)
Cancers	Melanoma	Male	65-74	13207	13.55 (13.93 - 13.18)	93.0 (92.5 - 93.4)	84.3 (83.7 - 85.0)	78.0 (77.3 - 78.8)	62.7 (61.7 - 63.7)	44.0 (42.6 - 45.5)	24.4 (22.4 - 26.5)
Cancers	Melanoma	Male	75-84	11225	6.76 (6.97 - 6.6)	88.8 (88.2 - 89.4)	73.2 (72.3 - 74.0)	60.9 (59.8 - 61.9)	32.8 (31.5 - 34.0)	10.8 (9.6 - 12.0)	3.2 (2.3 - 4.4)
Cancers	Melanoma	Male	85-95	3685	2.95 (3.07 - 2.77)	77.8 (76.4 - 79.1)	49.1 (47.4 - 50.8)	29.9 (28.2 - 31.5)	6.2 (5.1 - 7.3)	0.3 (0.1 - 0.9)	0.0 (0.0 - 0.0)
Cancers	Myeloma	Male	All	16467	4.71 (4.84 - 4.56)	81.5 (80.9 - 82.1)	62.5 (61.7 - 63.3)	48.3 (47.5 - 49.1)	27.0 (26.2 - 27.9)	16.5 (15.7 - 17.4)	11.2 (10.2 - 12.2)
Cancers	Myeloma	Male	16-24	56	NR	87.4 (75.4 - 93.8)	83.6 (70.9 - 91.1)	81.7 (68.6 - 89.7)	81.7 (68.6 - 89.7)	81.7 (68.6 - 89.7)	72.6 (48.3 - 86.9)
Cancers	Myeloma	Male	25-34	105	NR	95.1 (88.7 - 97.9)	89.0 (81.1 - 93.8)	87.9 (79.7 - 93.0)	83.7 (74.1 - 89.9)	83.7 (74.1 - 89.9)	77.7 (60.7 - 88.0)
Cancers	Myeloma	Male	35-44	348	NR	95.5 (92.7 - 97.3)	88.4 (84.3 - 91.4)	81.6 (76.7 - 85.6)	65.7 (59.3 - 71.3)	60.6 (53.6 - 66.9)	55.0 (46.5 - 62.7)
Cancers	Myeloma	Male	45-54	1121	12.17 (13.46 - 10.93)	92.4 (90.6 - 93.8)	83.2 (80.8 - 85.3)	75.0 (72.2 - 77.6)	57.3 (53.8 - 60.7)	43.9 (39.8 - 48.0)	37.1 (32.0 - 42.2)
Cancers	Myeloma	Male	55-64	2869	8.5 (8.99 - 7.96)	90.5 (89.4 - 91.5)	78.4 (76.8 - 79.9)	67.2 (65.3 - 69.0)	44.1 (41.9 - 46.3)	29.8 (27.5 - 32.3)	20.1 (17.3 - 22.9)

Appendix Table A6: Unadjusted Median Survival and Survival Probabilities by Age, males (Page 4 of 18)

Group	Diagnosis Category	Sex	Age	N	Median Survival (y)	1y (%)	3y (%)	5y (%)	10y (%)	15y (%)	20y (%)
Cancers	Myeloma	Male	65-74	5106	5.66 (5.92 - 5.48)	86.6 (85.6 - 87.5)	69.4 (68.1 - 70.7)	55.2 (53.7 - 56.7)	29.3 (27.7 - 31.0)	13.4 (11.8 - 15.1)	5.3 (3.9 - 7.0)
Cancers	Myeloma	Male	75-84	5262	3.09 (3.23 - 2.98)	76.1 (74.9 - 77.2)	51.1 (49.6 - 52.5)	32.2 (30.8 - 33.6)	9.4 (8.3 - 10.6)	2.2 (1.5 - 3.0)	0.7 (0.3 - 1.6)
Cancers	Myeloma	Male	85-95	1574	1.33 (1.48 - 1.19)	55.3 (52.8 - 57.8)	24.5 (22.3 - 26.8)	10.7 (9.1 - 12.5)	1.4 (0.7 - 2.3)	0.2 (0.0 - 0.9)	0.0 (0.0 - 0.0)
Cancers	Nasopharynx cancer	Male	All	1075	5.36 (6.48 - 4.43)	78.0 (75.4 - 80.4)	60.8 (57.8 - 63.7)	51.7 (48.5 - 54.7)	38.4 (35.2 - 41.5)	29.3 (26.0 - 32.6)	22.0 (18.6 - 25.7)
Cancers	Nasopharynx cancer	Male	16-24	25	NR	96.0 (74.8 - 99.4)	85.3 (60.7 - 95.1)	85.3 (60.7 - 95.1)	78.8 (52.0 - 91.6)	78.8 (52.0 - 91.6)	78.8 (52.0 - 91.6)
Cancers	Nasopharynx cancer	Male	25-34	30	NR	96.7 (78.6 - 99.5)	89.9 (71.8 - 96.6)	82.4 (62.6 - 92.3)	78.5 (58.1 - 89.7)	78.5 (58.1 - 89.7)	64.4 (38.5 - 81.6)
Cancers	Nasopharynx cancer	Male	35-44	80	NR	93.6 (85.3 - 97.3)	78.9 (67.9 - 86.5)	74.8 (63.4 - 83.2)	63.2 (50.4 - 73.4)	56.7 (42.2 - 68.8)	53.3 (38.4 - 66.2)
Cancers	Nasopharynx cancer	Male	45-54	170	16.38 (20.92 - 13.62)	91.0 (85.5 - 94.5)	81.5 (74.7 - 86.7)	71.6 (63.8 - 78.0)	64.3 (55.9 - 71.5)	55.4 (46.1 - 63.7)	41.0 (30.3 - 51.3)
Cancers	Nasopharynx cancer	Male	55-64	266	7.53 (11.04 - 5.28)	82.0 (76.8 - 86.2)	65.3 (59.1 - 70.8)	58.1 (51.7 - 64.0)	45.2 (38.6 - 51.5)	34.2 (27.4 - 41.0)	25.9 (18.3 - 34.2)
Cancers	Nasopharynx cancer	Male	65-74	275	3.47 (4.29 - 2.22)	70.8 (65.0 - 75.9)	51.9 (45.7 - 57.8)	41.1 (35.0 - 47.1)	25.3 (19.7 - 31.2)	14.2 (9.3 - 20.1)	7.2 (2.8 - 14.2)
Cancers	Nasopharynx cancer	Male	75-84	195	2.07 (2.81 - 1.56)	65.0 (57.7 - 71.3)	42.2 (35.0 - 49.1)	31.0 (24.4 - 37.7)	11.2 (6.8 - 16.9)	2.5 (0.6 - 7.1)	0.0 (0.0 - 0.0)
Cancers	Nasopharynx cancer	Male	85-95	34	0.54 (1.84 - 0.31)	47.1 (29.8 - 62.5)	17.6 (7.2 - 31.9)	5.9 (1.1 - 17.2)	0.0 (0.0 - 0.0)	0.0 (0.0 - 0.0)	0.0 (0.0 - 0.0)
Cancers	Other pharynx cancer	Male	All	7304	7.11 (7.49 - 6.69)	78.5 (77.6 - 79.5)	63.0 (61.9 - 64.1)	55.6 (54.4 - 56.8)	43.2 (41.9 - 44.5)	33.8 (32.4 - 35.3)	25.4 (23.7 - 27.2)
Cancers	Other pharynx cancer	Male	16-24	24	NR	95.8 (73.9 - 99.4)	95.8 (73.9 - 99.4)	95.8 (73.9 - 99.4)	91.3 (69.3 - 97.7)	91.3 (69.3 - 97.7)	91.3 (69.3 - 97.7)
Cancers	Other pharynx cancer	Male	25-34	57	NR	94.7 (84.4 - 98.3)	91.1 (79.9 - 96.2)	87.2 (74.9 - 93.7)	82.9 (69.5 - 90.7)	77.3 (62.3 - 87.0)	77.3 (62.3 - 87.0)
Cancers	Other pharynx cancer	Male	35-44	248	NR	93.0 (88.9 - 95.6)	88.2 (83.4 - 91.7)	86.8 (81.8 - 90.6)	83.3 (77.8 - 87.6)	81.3 (75.4 - 86.0)	78.0 (70.9 - 83.5)
Cancers	Other pharynx cancer	Male	45-54	1065	20.27	92.4 (90.7 - 93.9)	83.4 (81.0 - 85.6)	77.8 (75.0 - 80.3)	70.7 (67.6 - 73.6)	61.8 (58.0 - 65.3)	51.5 (46.7 - 56.1)
Cancers	Other pharynx cancer	Male	55-64	2318	11.76 (12.78 - 10.19)	83.8 (82.2 - 85.2)	70.7 (68.8 - 72.6)	64.6 (62.5 - 66.6)	52.8 (50.4 - 55.1)	41.8 (39.1 - 44.5)	28.3 (24.8 - 32.0)
Cancers	Other pharynx cancer	Male	65-74	2261	4.11 (4.64 - 3.68)	75.0 (73.1 - 76.7)	55.9 (53.7 - 58.0)	46.1 (43.9 - 48.3)	29.6 (27.3 - 31.9)	17.2 (14.8 - 19.9)	7.4 (5.1 - 10.3)
Cancers	Other pharynx cancer	Male	75-84	1092	1.87 (2.16 - 1.54)	62.2 (59.2 - 65.1)	40.6 (37.5 - 43.6)	31.3 (28.3 - 34.4)	13.7 (11.1 - 16.4)	4.0 (2.4 - 6.4)	2.7 (1.2 - 5.0)
Cancers	Other pharynx cancer	Male	85-95	231	1.17 (1.41 - 0.77)	53.3 (46.6 - 59.6)	27.0 (21.3 - 33.1)	16.8 (12.0 - 22.3)	4.4 (1.9 - 8.6)	1.3 (0.2 - 5.5)	0.0 (0.0 - 0.0)
Cancers	Pancreatic cancer	Male	All	22345	0.39 (0.4 - 0.38)	30.0 (29.3 - 30.6)	14.0 (13.5 - 14.4)	10.3 (9.8 - 10.7)	6.8 (6.4 - 7.2)	5.1 (4.7 - 5.5)	3.4 (2.9 - 4.0)
Cancers	Pancreatic cancer	Male	16-24	33	NR	84.6 (66.9 - 93.3)	78.1 (59.4 - 88.9)	74.2 (54.8 - 86.3)	74.2 (54.8 - 86.3)	74.2 (54.8 - 86.3)	74.2 (54.8 - 86.3)
Cancers	Pancreatic cancer	Male	25-34	71	NR	84.2 (73.4 - 91.0)	79.8 (68.2 - 87.5)	72.2 (59.4 - 81.7)	65.9 (52.1 - 76.6)	57.6 (41.0 - 71.1)	57.6 (41.0 - 71.1)
Cancers	Pancreatic cancer	Male	35-44	291	1.57 (2.24 - 1.03)	56.7 (50.8 - 62.2)	42.2 (36.3 - 47.9)	39.2 (33.4 - 44.9)	34.3 (28.5 - 40.3)	32.2 (26.3 - 38.3)	26.4 (17.3 - 36.4)
Cancers	Pancreatic cancer	Male	45-54	1214	0.91 (1.01 - 0.82)	47.2 (44.3 - 50.0)	26.4 (23.8 - 28.9)	21.9 (19.5 - 24.4)	17.0 (14.7 - 19.4)	15.0 (12.7 - 17.6)	11.5 (8.7 - 14.8)
Cancers	Pancreatic cancer	Male	55-64	4044	0.64 (0.67 - 0.6)	38.2 (36.7 - 39.8)	19.0 (17.8 - 20.3)	14.0 (12.9 - 15.2)	10.2 (9.2 - 11.3)	8.0 (7.0 - 9.2)	4.7 (3.5 - 6.3)
Cancers	Pancreatic cancer	Male	65-74	7581	0.48 (0.5 - 0.45)	33.0 (32.0 - 34.1)	14.3 (13.4 - 15.1)	10.4 (9.6 - 11.1)	6.5 (5.8 - 7.2)	4.1 (3.4 - 4.9)	2.5 (1.7 - 3.5)
Cancers	Pancreatic cancer	Male	75-84	6968	0.26 (0.28 - 0.25)	23.0 (22.0 - 24.0)	9.7 (9.0 - 10.4)	6.2 (5.6 - 6.9)	2.5 (2.0 - 3.1)	0.7 (0.4 - 1.3)	0.0 (0.0 - 0.0)
Cancers	Pancreatic cancer	Male	85-95	2105	0.14 (0.15 - 0.13)	9.7 (8.4 - 11.0)	2.6 (1.9 - 3.3)	1.2 (0.8 - 1.9)	0.0 (0.0 - 0.0)	0.0 (0.0 - 0.0)	0.0 (0.0 - 0.0)
Cancers	Prostate cancer	Male	All	253100	11.12 (11.18 - 11.05)	92.6 (92.5 - 92.7)	81.4 (81.2 - 81.5)	72.4 (72.2 - 72.6)	53.8 (53.6 - 54.0)	38.3 (38.1 - 38.6)	25.5 (25.1 - 25.8)
Cancers	Prostate cancer	Male	16-24	50	NR	98.0 (86.6 - 99.7)	92.0 (80.1 - 96.9)	92.0 (80.1 - 96.9)	90.0 (77.5 - 95.7)	87.3 (73.7 - 94.1)	87.3 (73.7 - 94.1)
Cancers	Prostate cancer	Male	25-34	120	NR	94.9 (89.1 - 97.7)	91.5 (84.8 - 95.3)	90.7 (83.8 - 94.7)	86.8 (79.0 - 91.8)	84.7 (76.5 - 90.2)	82.7 (73.5 - 89.0)
Cancers	Prostate cancer	Male	35-44	459	NR	96.7 (94.6 - 98.0)	93.9 (91.2 - 95.8)	91.1 (88.0 - 93.5)	88.4 (84.8 - 91.2)	87.3 (83.3 - 90.3)	85.8 (81.3 - 89.3)
Cancers	Prostate cancer	Male	45-54	8935	NR	98.4 (98.2 - 98.7)	95.1 (94.6 - 95.6)	92.9 (92.3 - 93.4)	87.8 (87.0 - 88.6)	82.4 (81.3 - 83.4)	76.0 (74.3 - 77.6)
Cancers	Prostate cancer	Male	55-64	53931	22.6	98.2 (98.0 - 98.3)	94.2 (94.0 - 94.4)	90.8 (90.5 - 91.1)	82.4 (82.0 - 82.8)	72.3 (71.8 - 72.8)	58.9 (58.1 - 59.7)
Cancers	Prostate cancer	Male	65-74	97348	13.85 (13.96 - 13.76)	96.4 (96.3 - 96.5)	89.3 (89.1 - 89.5)	82.9 (82.6 - 83.1)	65.6 (65.3 - 66.0)	45.0 (44.5 - 45.5)	24.3 (23.7 - 24.9)
Cancers	Prostate cancer	Male	75-84	70426	5.91 (5.98 - 5.86)	89.0 (88.8 - 89.2)	71.4 (71.0 - 71.7)	56.5 (56.1 - 56.9)	25.8 (25.4 - 26.2)	7.8 (7.5 - 8.1)	1.2 (1.0 - 1.4)
Cancers	Prostate cancer	Male	85-95	21369	2.29 (2.34 - 2.26)	71.4 (70.8 - 72.0)	41.2 (40.5 - 41.9)	22.5 (21.9 - 23.1)	3.4 (3.1 - 3.7)	0.3 (0.2 - 0.4)	0.0 (0.0 - 0.2)
Cancers	Soft tissue and other extraosseous sarcomas	Male	All	8795	7.54 (7.97 - 7.11)	79.3 (78.4 - 80.1)	64.4 (63.4 - 65.5)	56.6 (55.5 - 57.7)	44.5 (43.3 - 45.7)	35.6 (34.3 - 36.9)	29.8 (28.3 - 31.4)
Cancers	Soft tissue and other extraosseous sarcomas	Male	16-24	305	NR	91.0 (87.1 - 93.7)	73.1 (67.6 - 77.8)	69.8 (64.1 - 74.7)	66.8 (60.9 - 72.0)	66.8 (60.9 - 72.0)	66.8 (60.9 - 72.0)
Cancers	Soft tissue and other extraosseous sarcomas	Male	25-34	403	NR	92.1 (89.0 - 94.4)	81.9 (77.6 - 85.4)	78.3 (73.7 - 82.1)	73.2 (68.1 - 77.6)	71.3 (65.9 - 75.9)	70.1 (64.4 - 75.2)
Cancers	Soft tissue and other extraosseous sarcomas	Male	35-44	615	NR	89.0 (86.2 - 91.3)	79.2 (75.7 - 82.3)	75.0 (71.2 - 78.3)	69.5 (65.3 - 73.2)	66.5 (62.1 - 70.5)	64.1 (58.9 - 68.8)
Cancers	Soft tissue and other extraosseous sarcomas	Male	45-54	1004	NR	88.9 (86.8 - 90.7)	78.9 (76.1 - 81.3)	74.6 (71.6 - 77.3)	67.8 (64.5 - 70.9)	63.4 (59.7 - 66.8)	58.5 (54.1 - 62.6)
Cancers	Soft tissue and other extraosseous sarcomas	Male	55-64	1620	12.78 (13.88 - 11.03)	83.1 (81.1 - 84.8)	70.6 (68.3 - 72.9)	65.0 (62.5 - 67.3)	55.1 (52.4 - 57.8)	44.4 (41.3 - 47.5)	37.8 (34.1 - 41.5)
Cancers	Soft tissue and other extraosseous sarcomas	Male	65-74	2245	6.82 (7.96 - 6.03)	78.6 (76.9 - 80.3)	64.1 (62.0 - 66.1)	55.2 (53.0 - 57.4)	43.1 (40.7 - 45.4)	27.1 (24.3 - 29.9)	12.6 (9.7 - 16.0)
Cancers	Soft tissue and other extraosseous sarcomas	Male	75-84	1909	3.59 (4.09 - 3.18)	72.3 (70.2 - 74.3)	53.4 (51.1 - 55.8)	42.3 (39.8 - 44.7)	18.2 (16.0 - 20.5)	5.3 (3.8 - 7.3)	1.2 (0.4 - 2.7)
Cancers	Soft tissue and other extraosseous sarcomas	Male	85-95	680	1.42 (1.7 - 1.14)	56.8 (52.9 - 60.5)	32.4 (28.7 - 36.0)	17.7 (14.7 - 20.9)	4.3 (2.6 - 6.6)	0.7 (0.2 - 2.3)	0.0 (0.0 - 0.0)
Cancers	Stomach cancer	Male	All	16383	0.98 (1.01 - 0.95)	49.4 (48.6 - 50.1)	29.0 (28.3 - 29.8)	22.8 (22.1 - 23.5)	15.4 (14.8 - 16.0)	10.7 (10.1 - 11.4)	7.0 (6.4 - 7.8)
Cancers	Stomach cancer	Male	16-24	22	1.2	57.8 (34.4 - 75.4)	47.3 (25.1 - 66.6)	41.3 (20.1 - 61.5)	41.3 (20.1 - 61.5)	41.3 (20.1 - 61.5)	41.3 (20.1 - 61.5)

Appendix Table A6: Unadjusted Median Survival and Survival Probabilities by Age, males (Page 5 of 18)

Group	Diagnosis Category	Sex	Age	N	Median Survival (y)	1y (%)	3y (%)	5y (%)	10y (%)	15y (%)	20y (%)
Cancers	Stomach cancer	Male	25-34	87	4.58	68.5 (57.5 - 77.2)	53.4 (42.1 - 63.5)	49.0 (37.6 - 59.4)	44.1 (32.1 - 55.4)	44.1 (32.1 - 55.4)	39.2 (25.5 - 52.6)
Cancers	Stomach cancer	Male	35-44	315	1.61 (2.38 - 1.41)	64.2 (58.5 - 69.2)	41.1 (35.4 - 46.6)	36.0 (30.4 - 41.5)	33.8 (28.3 - 39.5)	31.6 (25.9 - 37.4)	31.6 (25.9 - 37.4)
Cancers	Stomach cancer	Male	45-54	973	1.62 (1.87 - 1.41)	60.5 (57.3 - 63.5)	39.5 (36.3 - 42.6)	33.5 (30.4 - 36.6)	28.5 (25.5 - 31.6)	25.5 (22.4 - 28.7)	22.3 (18.7 - 26.1)
Cancers	Stomach cancer	Male	55-64	2829	1.48 (1.62 - 1.37)	58.7 (56.9 - 60.5)	37.2 (35.3 - 39.0)	30.9 (29.1 - 32.7)	24.9 (23.1 - 26.6)	20.2 (18.5 - 22.0)	14.7 (12.8 - 16.8)
Cancers	Stomach cancer	Male	65-74	5061	1.28 (1.34 - 1.21)	55.3 (54.0 - 56.7)	33.7 (32.4 - 35.1)	27.3 (26.0 - 28.6)	18.9 (17.6 - 20.1)	12.4 (11.2 - 13.7)	5.9 (4.5 - 7.5)
Cancers	Stomach cancer	Male	75-84	5240	0.75 (0.79 - 0.7)	43.1 (41.7 - 44.4)	23.5 (22.3 - 24.7)	16.9 (15.8 - 18.0)	7.6 (6.7 - 8.5)	2.4 (1.8 - 3.1)	0.6 (0.3 - 1.2)
Cancers	Stomach cancer	Male	85-95	1822	0.36 (0.39 - 0.32)	27.4 (25.4 - 29.5)	10.6 (9.1 - 12.1)	5.3 (4.3 - 6.5)	0.9 (0.5 - 1.6)	0.1 (0.0 - 0.7)	0.0 (0.0 - 0.0)
Cancers	Testicular cancer	Male	All	10300	NR	96.4 (96.0 - 96.7)	93.6 (93.1 - 94.1)	91.6 (91.1 - 92.2)	88.1 (87.4 - 88.7)	84.6 (83.8 - 85.5)	80.0 (78.9 - 81.1)
Cancers	Testicular cancer	Male	16-24	1129	NR	99.3 (98.6 - 99.6)	98.4 (97.4 - 99.0)	98.4 (97.4 - 99.0)	97.9 (96.8 - 98.6)	97.5 (96.3 - 98.4)	96.4 (94.5 - 97.6)
Cancers	Testicular cancer	Male	25-34	3189	NR	99.2 (98.8 - 99.5)	98.6 (98.1 - 99.0)	98.3 (97.8 - 98.7)	97.9 (97.3 - 98.4)	97.1 (96.3 - 97.7)	96.2 (95.2 - 97.1)
Cancers	Testicular cancer	Male	35-44	2596	NR	99.1 (98.6 - 99.4)	98.0 (97.4 - 98.5)	97.4 (96.7 - 98.0)	96.5 (95.6 - 97.2)	95.5 (94.5 - 96.4)	92.6 (91.0 - 94.0)
Cancers	Testicular cancer	Male	45-54	1478	NR	98.3 (97.5 - 98.8)	96.9 (95.8 - 97.7)	95.6 (94.4 - 96.6)	92.2 (90.4 - 93.6)	88.5 (86.3 - 90.4)	82.3 (79.1 - 85.1)
Cancers	Testicular cancer	Male	55-64	766	20.05	93.5 (91.5 - 95.1)	88.4 (85.9 - 90.5)	84.9 (82.1 - 87.3)	78.3 (74.9 - 81.3)	67.4 (63.2 - 71.3)	50.3 (44.6 - 55.6)
Cancers	Testicular cancer	Male	65-74	625	10.62 (11.97 - 8.95)	86.1 (83.1 - 88.6)	76.3 (72.7 - 79.5)	68.6 (64.7 - 72.2)	51.6 (47.1 - 55.9)	35.4 (30.6 - 40.3)	21.7 (16.5 - 27.3)
Cancers	Testicular cancer	Male	75-84	377	3.48 (4.4 - 2.88)	74.0 (69.2 - 78.1)	54.7 (49.4 - 59.8)	40.5 (35.2 - 45.8)	15.6 (11.6 - 20.2)	5.4 (3.0 - 8.8)	2.2 (0.8 - 4.9)
Cancers	Testicular cancer	Male	85-95	138	1.89 (2.53 - 1.12)	62.2 (53.5 - 69.7)	35.7 (27.6 - 43.8)	16.2 (10.4 - 23.1)	2.7 (0.6 - 7.6)	0.0 (0.0 - 0.0)	0.0 (0.0 - 0.0)
Cancers	Thyroid cancer	Male	All	4165	15.96 (17.51 - 15.08)	84.7 (83.5 - 85.7)	77.8 (76.5 - 79.1)	72.4 (70.9 - 73.8)	61.3 (59.5 - 63.0)	52.1 (50.1 - 54.1)	46.0 (43.7 - 48.3)
Cancers	Thyroid cancer	Male	16-24	101	NR	99.0 (93.1 - 99.9)	99.0 (93.1 - 99.9)	97.9 (91.8 - 99.5)	95.3 (87.9 - 98.2)	91.1 (80.4 - 96.1)	91.1 (80.4 - 96.1)
Cancers	Thyroid cancer	Male	25-34	337	NR	98.8 (96.8 - 99.5)	98.4 (96.3 - 99.4)	97.3 (94.7 - 98.7)	96.9 (94.1 - 98.4)	94.2 (89.3 - 96.9)	92.0 (85.8 - 95.5)
Cancers	Thyroid cancer	Male	35-44	514	NR	99.4 (98.1 - 99.8)	97.7 (95.9 - 98.7)	97.2 (95.2 - 98.3)	94.7 (91.8 - 96.5)	91.9 (87.8 - 94.6)	87.6 (81.9 - 91.7)
Cancers	Thyroid cancer	Male	45-54	628	NR	93.4 (91.1 - 95.1)	90.4 (87.8 - 92.5)	87.6 (84.6 - 90.1)	82.5 (78.9 - 85.5)	77.5 (73.1 - 81.2)	73.2 (67.9 - 77.8)
Cancers	Thyroid cancer	Male	55-64	836	17.12 (20.23 - 14.15)	87.2 (84.7 - 89.3)	82.2 (79.3 - 84.7)	78.3 (75.2 - 81.1)	64.9 (60.8 - 68.6)	53.8 (49.1 - 58.3)	44.7 (39.1 - 50.1)
Cancers	Thyroid cancer	Male	65-74	959	8.2 (9.14 - 6.92)	78.6 (75.8 - 81.1)	67.6 (64.4 - 70.5)	59.7 (56.3 - 62.9)	43.9 (40.1 - 47.6)	26.9 (22.8 - 31.1)	16.4 (12.2 - 21.1)
Cancers	Thyroid cancer	Male	75-84	655	4.0 (4.58 - 3.23)	69.3 (65.6 - 72.7)	55.2 (51.1 - 59.1)	43.6 (39.3 - 47.7)	20.8 (16.9 - 24.9)	9.4 (6.3 - 13.3)	5.4 (2.9 - 9.0)
Cancers	Thyroid cancer	Male	85-95	130	0.61 (1.62 - 0.23)	44.1 (35.4 - 52.5)	28.6 (21.0 - 36.7)	16.2 (10.1 - 23.6)	3.2 (0.7 - 9.1)	0.0 (0.0 - 0.0)	0.0 (0.0 - 0.0)
Cardiovascular diseases	Acute myocardial infarction	Male	All	272870	9.59 (9.66 - 9.51)	81.6 (81.5 - 81.8)	72.2 (72.0 - 72.3)	64.5 (64.3 - 64.7)	48.8 (48.6 - 49.0)	35.6 (35.4 - 35.8)	24.5 (24.3 - 24.8)
Cardiovascular diseases	Acute myocardial infarction	Male	16-24	141	NR	94.3 (88.9 - 97.1)	89.6 (83.1 - 93.7)	87.1 (80.0 - 91.8)	82.4 (74.4 - 88.1)	82.4 (74.4 - 88.1)	82.4 (74.4 - 88.1)
Cardiovascular diseases	Acute myocardial infarction	Male	25-34	779	NR	97.0 (95.5 - 98.0)	94.7 (92.8 - 96.1)	93.9 (91.9 - 95.4)	91.1 (88.6 - 93.1)	88.2 (85.1 - 90.8)	85.7 (81.7 - 88.8)
Cardiovascular diseases	Acute myocardial infarction	Male	35-44	5848	NR	97.6 (97.1 - 97.9)	96.5 (96.0 - 96.9)	95.4 (94.9 - 96.0)	92.0 (91.2 - 92.7)	87.2 (86.1 - 88.3)	80.1 (78.3 - 81.7)
Cardiovascular diseases	Acute myocardial infarction	Male	45-54	25271	NR	97.0 (96.8 - 97.2)	95.5 (95.3 - 95.8)	94.1 (93.8 - 94.4)	89.1 (88.7 - 89.5)	81.8 (81.2 - 82.4)	72.2 (71.3 - 73.1)
Cardiovascular diseases	Acute myocardial infarction	Male	55-64	55739	20.19 (20.4 - 19.95)	94.5 (94.3 - 94.7)	91.4 (91.1 - 91.6)	88.3 (88.0 - 88.6)	79.0 (78.6 - 79.4)	66.7 (66.2 - 67.2)	50.6 (49.8 - 51.3)
Cardiovascular diseases	Acute myocardial infarction	Male	65-74	73689	11.99 (12.11 - 11.9)	88.2 (88.0 - 88.5)	81.8 (81.5 - 82.0)	75.3 (75.0 - 75.7)	58.0 (57.6 - 58.4)	37.5 (37.1 - 38.0)	17.5 (17.0 - 18.1)
Cardiovascular diseases	Acute myocardial infarction	Male	75-84	74748	4.51 (4.58 - 4.45)	73.8 (73.5 - 74.1)	59.2 (58.8 - 59.5)	47.1 (46.7 - 47.5)	22.1 (21.7 - 22.4)	6.8 (6.5 - 7.0)	1.2 (1.0 - 1.3)
Cardiovascular diseases	Acute myocardial infarction	Male	85-95	35449	1.15 (1.18 - 1.11)	52.1 (51.5 - 52.6)	30.3 (29.8 - 30.8)	16.7 (16.3 - 17.1)	2.7 (2.5 - 2.9)	0.2 (0.1 - 0.3)	0.0 (0.0 - 0.1)
Cardiovascular diseases	Angina	Male	All	260808	12.87 (12.93 - 12.8)	92.6 (92.5 - 92.7)	84.6 (84.5 - 84.8)	77.2 (77.0 - 77.4)	59.5 (59.3 - 59.7)	43.6 (43.4 - 43.8)	30.0 (29.7 - 30.3)
Cardiovascular diseases	Angina	Male	16-24	103	NR	99.0 (93.1 - 99.9)	97.9 (91.9 - 99.5)	95.7 (89.0 - 98.4)	95.7 (89.0 - 98.4)	95.7 (89.0 - 98.4)	95.7 (89.0 - 98.4)
Cardiovascular diseases	Angina	Male	25-34	474	NR	98.9 (97.5 - 99.6)	96.9 (94.8 - 98.2)	96.4 (94.2 - 97.8)	92.1 (89.0 - 94.4)	87.9 (84.0 - 90.9)	82.3 (77.0 - 86.5)
Cardiovascular diseases	Angina	Male	35-44	4832	NR	99.5 (99.2 - 99.6)	98.4 (98.0 - 98.7)	97.2 (96.7 - 97.7)	93.8 (93.0 - 94.5)	89.3 (88.2 - 90.3)	83.3 (81.8 - 84.8)
Cardiovascular diseases	Angina	Male	45-54	24968	NR	98.9 (98.7 - 99.0)	97.4 (97.2 - 97.6)	95.9 (95.7 - 96.2)	91.0 (90.6 - 91.4)	84.0 (83.5 - 84.6)	74.7 (73.9 - 75.4)
Cardiovascular diseases	Angina	Male	55-64	62017	21.55 (21.72 - 21.39)	98.1 (98.0 - 98.2)	95.4 (95.2 - 95.6)	92.6 (92.4 - 92.8)	83.7 (83.4 - 84.1)	71.6 (71.2 - 72.0)	55.2 (54.6 - 55.8)
Cardiovascular diseases	Angina	Male	65-74	80679	13.6 (13.69 - 13.5)	95.7 (95.5 - 95.8)	90.3 (90.1 - 90.5)	84.6 (84.3 - 84.9)	66.4 (66.1 - 66.8)	43.6 (43.2 - 44.1)	21.1 (20.6 - 21.6)
Cardiovascular diseases	Angina	Male	75-84	65755	6.88 (6.93 - 6.82)	88.4 (88.1 - 88.6)	74.9 (74.6 - 75.2)	62.0 (61.6 - 62.4)	31.1 (30.7 - 31.5)	10.2 (9.9 - 10.5)	1.9 (1.7 - 2.1)
Cardiovascular diseases	Angina	Male	85-95	21385	2.56 (2.62 - 2.49)	70.6 (70.0 - 71.2)	45.1 (44.4 - 45.8)	26.5 (25.9 - 27.1)	4.4 (4.1 - 4.8)	0.4 (0.3 - 0.5)	0.1 (0.1 - 0.2)
Cardiovascular diseases	Aortic aneurysm	Male	All	79839	8.75 (8.87 - 8.67)	86.5 (86.2 - 86.7)	75.9 (75.6 - 76.2)	66.4 (66.1 - 66.8)	45.1 (44.7 - 45.5)	28.1 (27.6 - 28.6)	16.8 (16.2 - 17.4)
Cardiovascular diseases	Aortic aneurysm	Male	16-24	290	NR	99.3 (97.3 - 99.8)	98.4 (95.8 - 99.4)	97.9 (95.0 - 99.1)	94.5 (89.7 - 97.1)	91.5 (85.2 - 95.1)	91.5 (85.2 - 95.1)
Cardiovascular diseases	Aortic aneurysm	Male	25-34	633	NR	98.4 (97.1 - 99.1)	97.7 (96.1 - 98.6)	96.6 (94.7 - 97.8)	95.1 (92.7 - 96.8)	92.5 (88.9 - 95.0)	89.5 (84.2 - 93.2)
Cardiovascular diseases	Aortic aneurysm	Male	35-44	1439	NR	97.5 (96.6 - 98.2)	96.7 (95.6 - 97.6)	96.1 (94.9 - 97.0)	92.5 (90.6 - 93.9)	89.3 (86.8 - 91.3)	81.6 (76.7 - 85.5)
Cardiovascular diseases	Aortic aneurysm	Male	45-54	3916	NR	96.7 (96.1 - 97.2)	94.6 (93.9 - 95.3)	92.8 (91.8 - 93.6)	86.4 (85.0 - 87.7)	75.7 (73.5 - 77.8)	64.6 (61.2 - 67.7)
Cardiovascular diseases	Aortic aneurysm	Male	55-64	12636	16.62 (17.02 - 16.04)	94.7 (94.3 - 95.0)	90.6 (90.0 - 91.1)	86.0 (85.4 - 86.7)	72.0 (71.0 - 72.9)	55.4 (54.1 - 56.7)	37.2 (35.3 - 39.2)
Cardiovascular diseases	Aortic aneurysm	Male	65-74	29024	11.06 (11.19 - 10.93)	92.1 (91.8 - 92.4)	84.8 (84.4 - 85.3)	77.3 (76.8 - 77.8)	55.1 (54.4 - 55.8)	30.2 (29.3 - 31.1)	11.5 (10.4 - 12.6)

Appendix Table A6: Unadjusted Median Survival and Survival Probabilities by Age, males (Page 6 of 18)

Group	Diagnosis Category	Sex	Age	N	Median Survival (y)	1y (%)	3y (%)	5y (%)	10y (%)	15y (%)	20y (%)
Cardiovascular diseases	Aortic aneurysm	Male	75-84	24960	5.06 (5.15 - 4.95)	81.0 (80.5 - 81.5)	65.0 (64.3 - 65.6)	50.3 (49.7 - 51.0)	20.3 (19.7 - 21.0)	5.0 (4.6 - 5.5)	0.9 (0.6 - 1.3)
Cardiovascular diseases	Aortic aneurysm	Male	85-95	6812	1.62 (1.71 - 1.52)	58.9 (57.8 - 60.1)	33.5 (32.4 - 34.7)	17.6 (16.6 - 18.6)	1.9 (1.5 - 2.3)	0.2 (0.1 - 0.4)	0.0 (0.0 - 0.0)
Cardiovascular diseases	Atrial fibrillation	Male	All	448173	8.29 (8.33 - 8.24)	85.6 (85.5 - 85.7)	73.5 (73.4 - 73.6)	63.5 (63.4 - 63.7)	44.1 (44.0 - 44.3)	30.4 (30.2 - 30.5)	20.8 (20.6 - 21.0)
Cardiovascular diseases	Atrial fibrillation	Male	16-24	1469	NR	98.5 (97.7 - 99.0)	97.7 (96.8 - 98.3)	97.2 (96.2 - 97.9)	95.7 (94.4 - 96.7)	94.6 (93.1 - 95.9)	94.0 (92.2 - 95.4)
Cardiovascular diseases	Atrial fibrillation	Male	25-34	4048	NR	99.3 (99.0 - 99.5)	98.6 (98.2 - 98.9)	98.3 (97.9 - 98.7)	97.1 (96.4 - 97.6)	95.9 (95.1 - 96.6)	94.1 (92.9 - 95.1)
Cardiovascular diseases	Atrial fibrillation	Male	35-44	9528	NR	98.8 (98.5 - 99.0)	97.8 (97.5 - 98.1)	96.9 (96.5 - 97.3)	94.8 (94.3 - 95.3)	92.1 (91.4 - 92.8)	87.5 (86.3 - 88.6)
Cardiovascular diseases	Atrial fibrillation	Male	45-54	25985	NR	97.7 (97.5 - 97.9)	95.5 (95.3 - 95.8)	93.5 (93.2 - 93.8)	88.1 (87.7 - 88.6)	81.3 (80.7 - 81.9)	73.4 (72.5 - 74.3)
Cardiovascular diseases	Atrial fibrillation	Male	55-64	68581	19.61 (19.82 - 19.42)	95.4 (95.2 - 95.6)	91.3 (91.1 - 91.5)	87.4 (87.2 - 87.7)	76.7 (76.3 - 77.1)	64.0 (63.6 - 64.5)	48.8 (48.1 - 49.5)
Cardiovascular diseases	Atrial fibrillation	Male	65-74	126562	11.44 (11.53 - 11.36)	91.6 (91.4 - 91.7)	83.7 (83.5 - 84.0)	76.6 (76.3 - 76.8)	56.4 (56.1 - 56.7)	34.3 (33.9 - 34.7)	15.5 (15.1 - 16.0)
Cardiovascular diseases	Atrial fibrillation	Male	75-84	142415	5.35 (5.39 - 5.31)	82.8 (82.6 - 83.0)	66.9 (66.6 - 67.1)	52.3 (52.1 - 52.6)	22.5 (22.2 - 22.8)	6.1 (5.9 - 6.3)	0.9 (0.8 - 1.1)
Cardiovascular diseases	Atrial fibrillation	Male	85-95	66989	1.97 (2.0 - 1.94)	64.2 (63.8 - 64.6)	37.7 (37.3 - 38.0)	20.2 (19.8 - 20.5)	2.7 (2.6 - 2.9)	0.2 (0.1 - 0.3)	0.0 (0.0 - 0.1)
Cardiovascular diseases	Cardiomyopathy	Male	All	54974	15.25 (15.5 - 14.92)	91.0 (90.8 - 91.3)	82.8 (82.5 - 83.2)	76.1 (75.7 - 76.5)	61.9 (61.4 - 62.4)	50.4 (49.8 - 51.0)	40.3 (39.6 - 41.1)
Cardiovascular diseases	Cardiomyopathy	Male	16-24	5077	NR	99.6 (99.4 - 99.8)	99.2 (98.9 - 99.4)	98.8 (98.4 - 99.0)	97.8 (97.3 - 98.3)	97.1 (96.4 - 97.6)	94.4 (92.8 - 95.6)
Cardiovascular diseases	Cardiomyopathy	Male	25-34	4030	NR	99.2 (98.8 - 99.4)	98.2 (97.7 - 98.6)	97.7 (97.2 - 98.2)	96.2 (95.4 - 96.8)	94.4 (93.4 - 95.3)	91.9 (90.3 - 93.3)
Cardiovascular diseases	Cardiomyopathy	Male	35-44	4722	NR	98.3 (97.9 - 98.6)	96.4 (95.8 - 96.9)	95.2 (94.6 - 95.8)	91.3 (90.3 - 92.2)	86.9 (85.5 - 88.1)	79.5 (77.3 - 81.5)
Cardiovascular diseases	Cardiomyopathy	Male	45-54	7191	NR	96.7 (96.3 - 97.1)	93.4 (92.8 - 94.0)	90.5 (89.7 - 91.2)	81.7 (80.6 - 82.7)	70.9 (69.4 - 72.3)	58.6 (56.5 - 60.7)
Cardiovascular diseases	Cardiomyopathy	Male	55-64	10862	15.69 (16.08 - 15.3)	94.2 (93.7 - 94.6)	88.0 (87.4 - 88.7)	82.7 (82.0 - 83.5)	68.2 (67.1 - 69.2)	52.2 (50.9 - 53.5)	35.8 (34.2 - 37.5)
Cardiovascular diseases	Cardiomyopathy	Male	65-74	11911	9.13 (9.38 - 8.9)	89.3 (88.7 - 89.8)	79.0 (78.3 - 79.8)	69.2 (68.3 - 70.1)	45.8 (44.7 - 46.9)	25.4 (24.2 - 26.6)	11.0 (9.7 - 12.3)
Cardiovascular diseases	Cardiomyopathy	Male	75-84	8872	4.46 (4.6 - 4.3)	80.4 (79.6 - 81.3)	61.3 (60.2 - 62.4)	45.9 (44.8 - 47.1)	17.6 (16.6 - 18.7)	5.0 (4.2 - 5.8)	0.9 (0.5 - 1.5)
Cardiovascular diseases	Cardiomyopathy	Male	85-95	2264	1.69 (1.87 - 1.51)	60.3 (58.2 - 62.3)	34.4 (32.3 - 36.5)	17.7 (15.9 - 19.5)	1.6 (1.0 - 2.6)	0.0 (0.0 - 0.0)	0.0 (0.0 - 0.0)
Cardiovascular diseases	Chronic ischemic heart disease	Male	All	392661	11.0 (11.06 - 10.95)	89.0 (88.9 - 89.1)	79.5 (79.4 - 79.7)	71.4 (71.2 - 71.5)	53.3 (53.1 - 53.5)	38.0 (37.8 - 38.2)	25.7 (25.4 - 25.9)
Cardiovascular diseases	Chronic ischemic heart disease	Male	16-24	105	NR	94.3 (87.7 - 97.4)	93.2 (86.3 - 96.7)	92.1 (84.8 - 96.0)	81.6 (71.4 - 88.4)	81.6 (71.4 - 88.4)	71.1 (51.3 - 84.0)
Cardiovascular diseases	Chronic ischemic heart disease	Male	25-34	741	NR	98.9 (97.8 - 99.5)	97.7 (96.3 - 98.6)	96.9 (95.3 - 98.0)	93.6 (91.1 - 95.4)	89.0 (85.6 - 91.7)	85.2 (80.3 - 88.9)
Cardiovascular diseases	Chronic ischemic heart disease	Male	35-44	6887	NR	99.0 (98.8 - 99.2)	97.8 (97.4 - 98.1)	96.9 (96.4 - 97.3)	93.3 (92.6 - 93.9)	87.7 (86.7 - 88.7)	80.6 (79.0 - 82.0)
Cardiovascular diseases	Chronic ischemic heart disease	Male	45-54	33788	NR	98.5 (98.4 - 98.6)	96.8 (96.6 - 97.0)	95.1 (94.9 - 95.4)	89.6 (89.3 - 90.0)	81.7 (81.2 - 82.2)	71.4 (70.6 - 72.2)
Cardiovascular diseases	Chronic ischemic heart disease	Male	55-64	85020	20.27 (20.42 - 20.09)	97.2 (97.1 - 97.3)	94.1 (93.9 - 94.2)	90.8 (90.6 - 91.0)	80.7 (80.4 - 81.0)	67.5 (67.1 - 67.9)	50.9 (50.4 - 51.5)
Cardiovascular diseases	Chronic ischemic heart disease	Male	65-74	118415	12.61 (12.69 - 12.54)	93.7 (93.6 - 93.8)	87.2 (87.1 - 87.4)	80.6 (80.4 - 80.8)	61.6 (61.3 - 61.9)	39.2 (38.8 - 39.6)	18.1 (17.7 - 18.6)
Cardiovascular diseases	Chronic ischemic heart disease	Male	75-84	106040	5.89 (5.95 - 5.85)	84.1 (83.9 - 84.3)	69.3 (69.0 - 69.6)	55.8 (55.5 - 56.1)	26.4 (26.1 - 26.7)	8.1 (7.9 - 8.3)	1.4 (1.2 - 1.6)
Cardiovascular diseases	Chronic ischemic heart disease	Male	85-95	40367	1.88 (1.91 - 1.84)	62.4 (61.9 - 62.9)	37.0 (36.6 - 37.5)	20.9 (20.5 - 21.4)	3.4 (3.2 - 3.7)	0.2 (0.2 - 0.3)	0.0 (0.0 - 0.1)
Cardiovascular diseases	Endocarditis	Male	All	14637	6.38 (6.68 - 6.11)	75.0 (74.3 - 75.7)	63.0 (62.2 - 63.8)	54.9 (54.0 - 55.7)	39.6 (38.7 - 40.5)	29.3 (28.3 - 30.3)	21.9 (20.7 - 23.1)
Cardiovascular diseases	Endocarditis	Male	16-24	258	NR	96.5 (93.4 - 98.2)	93.2 (89.3 - 95.7)	91.4 (87.1 - 94.3)	88.2 (83.2 - 91.8)	84.5 (78.2 - 89.1)	67.7 (52.0 - 79.2)
Cardiovascular diseases	Endocarditis	Male	25-34	597	NR	95.9 (94.0 - 97.3)	92.7 (90.2 - 94.5)	90.0 (87.2 - 92.3)	85.5 (82.0 - 88.4)	82.6 (78.5 - 86.0)	79.9 (74.7 - 84.2)
Cardiovascular diseases	Endocarditis	Male	35-44	744	NR	92.4 (90.2 - 94.1)	88.1 (85.4 - 90.2)	84.7 (81.8 - 87.2)	80.3 (77.0 - 83.2)	72.2 (67.9 - 75.9)	65.1 (59.5 - 70.2)
Cardiovascular diseases	Endocarditis	Male	45-54	1252	21.29	86.8 (84.8 - 88.6)	80.8 (78.4 - 82.9)	77.4 (74.9 - 79.7)	67.6 (64.7 - 70.4)	59.4 (55.8 - 62.7)	51.3 (46.7 - 55.7)
Cardiovascular diseases	Endocarditis	Male	55-64	2363	12.83 (13.55 - 12.08)	84.9 (83.4 - 86.3)	77.7 (75.9 - 79.4)	71.6 (69.7 - 73.5)	57.1 (54.8 - 59.4)	44.6 (42.0 - 47.3)	33.0 (29.5 - 36.6)
Cardiovascular diseases	Endocarditis	Male	65-74	3758	7.27 (7.76 - 6.94)	77.5 (76.1 - 78.8)	66.9 (65.3 - 68.4)	59.1 (57.4 - 60.7)	39.9 (38.0 - 41.8)	22.4 (20.3 - 24.5)	7.8 (5.7 - 10.2)
Cardiovascular diseases	Endocarditis	Male	75-84	4078	2.84 (3.04 - 2.63)	66.1 (64.7 - 67.6)	48.6 (47.0 - 50.2)	36.3 (34.7 - 38.0)	13.7 (12.3 - 15.1)	3.5 (2.6 - 4.6)	0.5 (0.1 - 1.5)
Cardiovascular diseases	Endocarditis	Male	85-95	1542	0.9 (1.09 - 0.79)	48.6 (46.1 - 51.1)	25.1 (22.8 - 27.4)	11.9 (10.1 - 13.8)	1.5 (0.8 - 2.5)	0.0 (0.0 - 0.0)	0.0 (0.0 - 0.0)
Cardiovascular diseases	Heart failure	Male	All	369972	4.08 (4.1 - 4.05)	74.4 (74.2 - 74.5)	57.1 (57.0 - 57.3)	44.6 (44.4 - 44.8)	24.9 (24.7 - 25.1)	14.3 (14.1 - 14.4)	8.2 (8.0 - 8.4)
Cardiovascular diseases	Heart failure	Male	16-24	787	NR	92.5 (90.4 - 94.1)	88.4 (85.8 - 90.5)	85.5 (82.6 - 87.9)	81.3 (78.0 - 84.2)	77.3 (73.2 - 80.8)	67.9 (60.9 - 73.9)
Cardiovascular diseases	Heart failure	Male	25-34	1649	NR	93.7 (92.4 - 94.8)	89.0 (87.4 - 90.5)	86.1 (84.2 - 87.8)	79.2 (76.7 - 81.4)	72.9 (69.7 - 75.8)	67.8 (63.5 - 71.6)
Cardiovascular diseases	Heart failure	Male	35-44	4676	NR	95.1 (94.4 - 95.7)	91.6 (90.7 - 92.4)	88.8 (87.8 - 89.7)	80.6 (79.2 - 81.9)	72.2 (70.4 - 73.9)	60.2 (57.5 - 62.9)
Cardiovascular diseases	Heart failure	Male	45-54	15892	19.24 (19.79 - 18.76)	93.7 (93.3 - 94.1)	88.8 (88.3 - 89.3)	84.4 (83.8 - 85.0)	73.3 (72.5 - 74.1)	60.9 (59.8 - 61.9)	47.4 (46.0 - 48.9)
Cardiovascular diseases	Heart failure	Male	55-64	43073	12.69 (12.87 - 12.5)	90.0 (89.7 - 90.3)	82.8 (82.4 - 83.1)	76.2 (75.8 - 76.7)	59.0 (58.5 - 59.6)	42.6 (42.0 - 43.3)	27.7 (26.9 - 28.5)
Cardiovascular diseases	Heart failure	Male	65-74	85149	7.28 (7.36 - 7.21)	84.1 (83.9 - 84.3)	71.9 (71.6 - 72.2)	61.4 (61.1 - 61.8)	37.3 (36.9 - 37.7)	18.3 (17.9 - 18.7)	6.7 (6.3 - 7.1)
Cardiovascular diseases	Heart failure	Male	75-84	131967	3.36 (3.4 - 3.33)	73.1 (72.8 - 73.3)	53.2 (52.9 - 53.5)	37.2 (36.9 - 37.5)	12.1 (11.9 - 12.4)	2.6 (2.5 - 2.8)	0.4 (0.3 - 0.5)
Cardiovascular diseases	Heart failure	Male	85-95	83057	1.29 (1.31 - 1.26)	54.8 (54.4 - 55.1)	28.0 (27.7 - 28.3)	13.1 (12.8 - 13.3)	1.3 (1.2 - 1.4)	0.1 (0.1 - 0.1)	0.0 (0.0 - 0.1)
Cardiovascular diseases	Hypertension	Male	All	1005395	13.93 (13.98 - 13.89)	92.7 (92.7 - 92.8)	85.4 (85.3 - 85.5)	78.5 (78.4 - 78.6)	62.2 (62.1 - 62.3)	46.9 (46.7 - 47.0)	33.8 (33.7 - 34.0)
Cardiovascular diseases	Hypertension	Male	16-24	4028	NR	99.5 (99.2 - 99.7)	99.0 (98.6 - 99.2)	98.6 (98.1 - 98.9)	97.1 (96.5 - 97.7)	95.7 (94.6 - 96.5)	93.4 (91.7 - 94.9)
Cardiovascular diseases	Hypertension	Male	25-34	11443	NR	99.3 (99.1 - 99.5)	98.5 (98.2 - 98.7)	97.6 (97.2 - 97.9)	95.7 (95.3 - 96.2)	92.9 (92.2 - 93.6)	89.4 (88.3 - 90.5)

Appendix Table A6: Unadjusted Median Survival and Survival Probabilities by Age, males (Page 7 of 18)

Group	Diagnosis Category	Sex	Age	N	Median Survival (y)	1y (%)	3y (%)	5y (%)	10y (%)	15y (%)	20y (%)
Cardiovascular diseases	Hypertension	Male	35-44	40494	NR	99.2 (99.1 - 99.3)	98.2 (98.1 - 98.3)	97.2 (97.0 - 97.4)	94.2 (93.9 - 94.4)	89.6 (89.2 - 90.0)	83.3 (82.6 - 84.0)
Cardiovascular diseases	Hypertension	Male	45-54	119887	NR	98.6 (98.5 - 98.7)	97.0 (96.9 - 97.1)	95.3 (95.2 - 95.5)	90.4 (90.2 - 90.5)	83.0 (82.7 - 83.3)	73.1 (72.6 - 73.6)
Cardiovascular diseases	Hypertension	Male	55-64	241695	20.9 (20.99 - 20.8)	97.3 (97.3 - 97.4)	94.3 (94.2 - 94.4)	91.4 (91.3 - 91.5)	82.4 (82.2 - 82.6)	69.7 (69.5 - 70.0)	53.2 (52.8 - 53.6)
Cardiovascular diseases	Hypertension	Male	65-74	294761	13.42 (13.47 - 13.36)	94.5 (94.4 - 94.6)	88.7 (88.6 - 88.9)	82.8 (82.7 - 83.0)	65.1 (64.9 - 65.3)	42.5 (42.2 - 42.7)	20.2 (19.8 - 20.6)
Cardiovascular diseases	Hypertension	Male	75-84	218162	6.78 (6.82 - 6.75)	87.6 (87.5 - 87.8)	74.7 (74.5 - 74.9)	61.7 (61.5 - 61.9)	30.8 (30.6 - 31.1)	9.6 (9.4 - 9.8)	1.6 (1.4 - 1.7)
Cardiovascular diseases	Hypertension	Male	85-95	72820	2.74 (2.78 - 2.71)	72.3 (71.9 - 72.6)	47.0 (46.7 - 47.4)	28.1 (27.8 - 28.5)	4.9 (4.7 - 5.1)	0.4 (0.3 - 0.5)	0.1 (0.0 - 0.2)
Cardiovascular diseases	Hypertensive heart disease	Male	All	38352	9.6 (9.77 - 9.43)	88.0 (87.7 - 88.4)	76.6 (76.2 - 77.1)	67.0 (66.6 - 67.5)	48.7 (48.2 - 49.3)	33.6 (33.0 - 34.1)	22.4 (21.9 - 23.0)
Cardiovascular diseases	Hypertensive heart disease	Male	16-24	38	NR	97.4 (82.8 - 99.6)	94.5 (79.7 - 98.6)	91.4 (75.4 - 97.1)	91.4 (75.4 - 97.1)	87.2 (68.8 - 95.1)	87.2 (68.8 - 95.1)
Cardiovascular diseases	Hypertensive heart disease	Male	25-34	184	NR	99.5 (96.2 - 99.9)	98.9 (95.7 - 99.7)	98.3 (94.7 - 99.4)	94.8 (89.9 - 97.4)	93.1 (87.5 - 96.3)	90.4 (83.1 - 94.7)
Cardiovascular diseases	Hypertensive heart disease	Male	35-44	916	NR	98.5 (97.4 - 99.1)	96.0 (94.5 - 97.1)	94.7 (93.0 - 96.0)	89.8 (87.5 - 91.7)	82.7 (79.7 - 85.4)	73.0 (68.5 - 76.9)
Cardiovascular diseases	Hypertensive heart disease	Male	45-54	3244	NR	97.9 (97.3 - 98.3)	95.2 (94.4 - 95.9)	92.6 (91.6 - 93.5)	85.6 (84.2 - 86.8)	76.3 (74.6 - 77.9)	65.9 (63.6 - 68.1)
Cardiovascular diseases	Hypertensive heart disease	Male	55-64	8295	18.05 (18.56 - 17.7)	96.2 (95.7 - 96.6)	91.7 (91.1 - 92.3)	87.0 (86.3 - 87.7)	75.0 (74.0 - 75.9)	60.0 (58.8 - 61.2)	43.5 (42.0 - 45.0)
Cardiovascular diseases	Hypertensive heart disease	Male	65-74	11411	10.89 (11.1 - 10.7)	92.2 (91.7 - 92.7)	83.8 (83.1 - 84.5)	75.0 (74.2 - 75.8)	54.1 (53.1 - 55.0)	31.6 (30.6 - 32.6)	14.0 (12.9 - 15.0)
Cardiovascular diseases	Hypertensive heart disease	Male	75-84	10488	4.89 (5.03 - 4.76)	82.7 (82.0 - 83.4)	64.5 (63.5 - 65.4)	49.2 (48.2 - 50.2)	21.7 (20.9 - 22.6)	5.9 (5.3 - 6.5)	0.9 (0.6 - 1.2)
Cardiovascular diseases	Hypertensive heart disease	Male	85-95	3687	1.71 (1.83 - 1.6)	61.1 (59.5 - 62.7)	33.7 (32.1 - 35.3)	17.4 (16.2 - 18.7)	2.4 (1.9 - 3.0)	0.1 (0.0 - 0.3)	0.0 (0.0 - 0.0)
Cardiovascular diseases	Intracerebral hem	Male	All	52694	4.35 (4.46 - 4.24)	66.7 (66.3 - 67.1)	56.0 (55.6 - 56.4)	47.5 (47.1 - 47.9)	32.4 (32.0 - 32.9)	22.5 (22.0 - 23.0)	15.8 (15.2 - 16.3)
Cardiovascular diseases	Intracerebral hem	Male	16-24	632	NR	93.3 (91.1 - 95.0)	92.3 (89.9 - 94.2)	91.2 (88.6 - 93.2)	88.9 (85.9 - 91.2)	87.8 (84.4 - 90.5)	87.1 (83.4 - 90.0)
Cardiovascular diseases	Intracerebral hem	Male	25-34	851	NR	91.1 (89.0 - 92.9)	88.9 (86.5 - 90.9)	87.7 (85.2 - 89.8)	84.9 (82.0 - 87.3)	80.5 (76.7 - 83.8)	79.2 (74.5 - 83.2)
Cardiovascular diseases	Intracerebral hem	Male	35-44	1673	NR	87.8 (86.1 - 89.3)	84.5 (82.7 - 86.2)	82.1 (80.2 - 83.9)	77.4 (75.2 - 79.5)	73.1 (70.5 - 75.5)	68.6 (65.1 - 71.8)
Cardiovascular diseases	Intracerebral hem	Male	45-54	4237	20.9 (22.03 - 20.06)	83.2 (82.0 - 84.3)	79.2 (78.0 - 80.5)	75.9 (74.6 - 77.2)	68.5 (66.9 - 70.0)	60.7 (58.8 - 62.4)	52.3 (50.0 - 54.6)
Cardiovascular diseases	Intracerebral hem	Male	55-64	8764	12.7 (13.1 - 12.21)	79.8 (79.0 - 80.7)	74.3 (73.3 - 75.2)	69.5 (68.5 - 70.5)	56.9 (55.8 - 58.1)	43.0 (41.7 - 44.3)	29.3 (27.7 - 30.9)
Cardiovascular diseases	Intracerebral hem	Male	65-74	13405	5.97 (6.2 - 5.77)	70.9 (70.1 - 71.7)	62.1 (61.2 - 62.9)	54.1 (53.2 - 54.9)	34.1 (33.2 - 35.0)	17.5 (16.6 - 18.4)	7.0 (6.1 - 8.0)
Cardiovascular diseases	Intracerebral hem	Male	75-84	15780	2.03 (2.13 - 1.92)	58.1 (57.3 - 58.9)	43.3 (42.5 - 44.1)	30.5 (29.8 - 31.3)	10.8 (10.2 - 11.5)	2.7 (2.3 - 3.1)	0.3 (0.1 - 0.5)
Cardiovascular diseases	Intracerebral hem	Male	85-95	7142	0.49 (0.55 - 0.43)	42.7 (41.5 - 43.8)	23.4 (22.4 - 24.5)	11.8 (11.0 - 12.6)	1.3 (1.0 - 1.6)	0.0 (0.0 - 0.0)	0.0 (0.0 - 0.0)
Cardiovascular diseases	Ischemic heart disease	Male	All	472227	11.41 (11.47 - 11.36)	87.3 (87.2 - 87.4)	78.6 (78.5 - 78.7)	71.0 (70.9 - 71.2)	54.4 (54.2 - 54.5)	39.8 (39.7 - 40.0)	27.5 (27.3 - 27.7)
Cardiovascular diseases	Ischemic heart disease	Male	16-24	851	NR	98.4 (97.3 - 99.1)	97.3 (95.9 - 98.2)	96.5 (94.9 - 97.6)	94.4 (92.2 - 96.0)	94.4 (92.2 - 96.0)	92.1 (87.5 - 95.1)
Cardiovascular diseases	Ischemic heart disease	Male	25-34	1758	NR	98.5 (97.8 - 99.0)	96.7 (95.7 - 97.5)	96.0 (94.9 - 96.9)	93.3 (91.7 - 94.5)	90.2 (88.1 - 91.9)	87.0 (84.3 - 89.4)
Cardiovascular diseases	Ischemic heart disease	Male	35-44	10544	NR	98.3 (98.1 - 98.6)	97.3 (97.0 - 97.6)	96.3 (95.9 - 96.7)	93.0 (92.4 - 93.5)	88.4 (87.6 - 89.1)	82.0 (80.8 - 83.1)
Cardiovascular diseases	Ischemic heart disease	Male	45-54	45463	NR	97.9 (97.8 - 98.0)	96.4 (96.2 - 96.5)	94.9 (94.7 - 95.1)	89.9 (89.6 - 90.2)	82.9 (82.5 - 83.3)	73.4 (72.8 - 74.1)
Cardiovascular diseases	Ischemic heart disease	Male	55-64	104503	20.98 (21.12 - 20.84)	96.3 (96.1 - 96.4)	93.3 (93.2 - 93.5)	90.4 (90.2 - 90.6)	81.3 (81.1 - 81.6)	69.2 (68.8 - 69.5)	53.2 (52.7 - 53.7)
Cardiovascular diseases	Ischemic heart disease	Male	65-74	136236	12.9 (12.96 - 12.82)	92.1 (91.9 - 92.2)	86.2 (86.0 - 86.4)	80.0 (79.8 - 80.3)	62.3 (62.0 - 62.6)	40.8 (40.5 - 41.2)	19.5 (19.1 - 19.9)
Cardiovascular diseases	Ischemic heart disease	Male	75-84	122519	5.68 (5.73 - 5.63)	80.9 (80.7 - 81.2)	66.9 (66.6 - 67.1)	54.1 (53.8 - 54.4)	26.2 (25.9 - 26.5)	8.4 (8.2 - 8.6)	1.5 (1.4 - 1.7)
Cardiovascular diseases	Ischemic heart disease	Male	85-95	48762	1.71 (1.74 - 1.67)	59.4 (59.0 - 59.9)	36.1 (35.7 - 36.6)	20.5 (20.1 - 20.9)	3.5 (3.3 - 3.7)	0.3 (0.2 - 0.3)	0.1 (0.0 - 0.1)
Cardiovascular diseases	Ischemic stroke	Male	All	337433	8.5 (8.55 - 8.45)	85.6 (85.5 - 85.7)	74.4 (74.2 - 74.5)	64.5 (64.4 - 64.7)	44.7 (44.5 - 44.9)	30.2 (30.0 - 30.4)	19.7 (19.5 - 20.0)
Cardiovascular diseases	Ischemic stroke	Male	16-24	857	NR	95.2 (93.5 - 96.4)	93.8 (91.9 - 95.2)	93.1 (91.1 - 94.6)	91.6 (89.3 - 93.4)	89.0 (86.0 - 91.5)	87.8 (84.2 - 90.6)
Cardiovascular diseases	Ischemic stroke	Male	25-34	2074	NR	96.8 (96.0 - 97.5)	96.0 (95.0 - 96.8)	95.0 (93.9 - 95.9)	92.6 (91.2 - 93.8)	90.9 (89.2 - 92.4)	87.7 (85.0 - 89.9)
Cardiovascular diseases	Ischemic stroke	Male	35-44	6657	NR	97.5 (97.1 - 97.8)	96.1 (95.6 - 96.6)	95.1 (94.5 - 95.6)	91.9 (91.1 - 92.6)	88.5 (87.4 - 89.4)	83.0 (81.3 - 84.4)
Cardiovascular diseases	Ischemic stroke	Male	45-54	22398	NR	96.5 (96.2 - 96.7)	94.2 (93.9 - 94.5)	92.2 (91.8 - 92.5)	86.5 (86.0 - 87.0)	78.8 (78.1 - 79.5)	68.3 (67.1 - 69.4)
Cardiovascular diseases	Ischemic stroke	Male	55-64	57438	18.71 (18.85 - 18.5)	94.8 (94.7 - 95.0)	91.0 (90.7 - 91.2)	87.2 (86.9 - 87.5)	75.9 (75.5 - 76.3)	62.2 (61.6 - 62.7)	45.5 (44.8 - 46.2)
Cardiovascular diseases	Ischemic stroke	Male	65-74	96448	11.24 (11.32 - 11.16)	91.0 (90.8 - 91.2)	83.8 (83.5 - 84.0)	76.0 (75.8 - 76.3)	55.5 (55.2 - 55.9)	33.5 (33.1 - 34.0)	15.1 (14.6 - 15.6)
Cardiovascular diseases	Ischemic stroke	Male	75-84	105197	5.27 (5.32 - 5.22)	81.8 (81.6 - 82.0)	66.3 (66.0 - 66.6)	51.7 (51.4 - 52.1)	22.2 (21.9 - 22.5)	6.2 (6.0 - 6.5)	1.0 (0.8 - 1.1)
Cardiovascular diseases	Ischemic stroke	Male	85-95	45027	2.06 (2.1 - 2.03)	64.5 (64.1 - 65.0)	39.2 (38.7 - 39.7)	21.9 (21.5 - 22.3)	3.2 (3.0 - 3.4)	0.2 (0.2 - 0.3)	0.0 (0.0 - 0.1)
Cardiovascular diseases	Nonrheum valv diseases	Male	All	145638	9.2 (9.29 - 9.11)	88.6 (88.4 - 88.7)	76.8 (76.5 - 77.0)	67.0 (66.7 - 67.2)	47.3 (47.0 - 47.6)	33.0 (32.6 - 33.3)	23.1 (22.7 - 23.5)
Cardiovascular diseases	Nonrheum valv diseases	Male	16-24	1804	NR	99.4 (98.9 - 99.7)	98.9 (98.3 - 99.3)	98.4 (97.6 - 98.9)	97.1 (96.1 - 97.9)	95.3 (93.8 - 96.4)	93.0 (90.7 - 94.8)
Cardiovascular diseases	Nonrheum valv diseases	Male	25-34	2281	NR	98.8 (98.2 - 99.2)	98.1 (97.5 - 98.6)	97.4 (96.6 - 98.0)	95.7 (94.7 - 96.6)	93.9 (92.5 - 95.0)	91.6 (89.7 - 93.2)
Cardiovascular diseases	Nonrheum valv diseases	Male	35-44	3842	NR	98.7 (98.2 - 99.0)	97.3 (96.7 - 97.8)	95.9 (95.2 - 96.6)	92.8 (91.8 - 93.6)	88.0 (86.7 - 89.3)	82.7 (80.7 - 84.5)
Cardiovascular diseases	Nonrheum valv diseases	Male	45-54	8917	NR	97.6 (97.2 - 97.9)	95.2 (94.7 - 95.7)	92.9 (92.3 - 93.4)	87.0 (86.1 - 87.8)	79.3 (78.1 - 80.4)	70.4 (68.8 - 72.0)
Cardiovascular diseases	Nonrheum valv diseases	Male	55-64	21602	18.99 (19.27 - 18.76)	96.3 (96.0 - 96.6)	92.1 (91.8 - 92.5)	88.0 (87.5 - 88.4)	76.6 (75.9 - 77.3)	63.2 (62.3 - 64.1)	45.9 (44.6 - 47.1)
Cardiovascular diseases	Nonrheum valv diseases	Male	65-74	41430	11.42 (11.57 - 11.27)	93.5 (93.3 - 93.8)	85.6 (85.2 - 85.9)	77.9 (77.5 - 78.3)	56.4 (55.8 - 57.0)	32.6 (31.9 - 33.4)	13.8 (13.1 - 14.6)
Cardiovascular diseases	Nonrheum valv diseases	Male	75-84	47724	5.57 (5.64 - 5.49)	85.7 (85.4 - 86.1)	69.3 (68.8 - 69.7)	54.1 (53.6 - 54.6)	23.3 (22.8 - 23.8)	6.4 (6.0 - 6.8)	0.9 (0.7 - 1.2)

Appendix Table A6: Unadjusted Median Survival and Survival Probabilities by Age, males (Page 8 of 18)

Group	Diagnosis Category	Sex	Age	N	Median Survival (y)	1y (%)	3y (%)	5y (%)	10y (%)	15y (%)	20y (%)
Cardiovascular diseases	Nonrheum valv diseases	Male	85-95	17554	2.07 (2.13 - 2.02)	66.8 (66.1 - 67.5)	38.5 (37.7 - 39.3)	21.1 (20.4 - 21.7)	2.6 (2.3 - 3.0)	0.2 (0.1 - 0.3)	0.0 (0.0 - 0.2)
Cardiovascular diseases	PAH	Male	All	5561	1.98 (2.09 - 1.86)	64.3 (63.0 - 65.6)	40.4 (39.0 - 41.8)	28.0 (26.7 - 29.3)	14.6 (13.5 - 15.9)	9.9 (8.7 - 11.1)	6.8 (5.5 - 8.2)
Cardiovascular diseases	PAH	Male	16-24	33	16.47	97.0 (80.4 - 99.6)	87.2 (69.3 - 95.0)	76.3 (56.4 - 88.0)	72.0 (51.4 - 85.0)	59.1 (34.4 - 77.1)	47.3 (19.9 - 70.6)
Cardiovascular diseases	PAH	Male	25-34	50	NR	89.7 (77.1 - 95.6)	85.0 (71.0 - 92.6)	85.0 (71.0 - 92.6)	78.9 (62.9 - 88.6)	68.8 (48.2 - 82.6)	51.6 (18.5 - 77.1)
Cardiovascular diseases	PAH	Male	35-44	78	NR	88.4 (78.9 - 93.8)	82.8 (72.2 - 89.6)	79.8 (68.6 - 87.3)	72.2 (59.5 - 81.5)	65.2 (49.8 - 76.9)	59.8 (42.0 - 73.8)
Cardiovascular diseases	PAH	Male	45-54	173	10.67 (15.06 - 8.16)	90.0 (84.4 - 93.7)	76.7 (69.4 - 82.5)	69.0 (61.1 - 75.6)	52.3 (43.2 - 60.7)	41.6 (31.5 - 51.4)	34.2 (23.9 - 44.7)
Cardiovascular diseases	PAH	Male	55-64	481	5.37 (6.43 - 4.78)	78.3 (74.3 - 81.8)	64.5 (59.8 - 68.7)	53.2 (48.2 - 57.9)	34.3 (29.1 - 39.5)	24.6 (19.3 - 30.3)	15.1 (9.2 - 22.5)
Cardiovascular diseases	PAH	Male	65-74	1339	2.74 (3.01 - 2.48)	71.0 (68.5 - 73.4)	47.5 (44.6 - 50.3)	34.7 (31.9 - 37.5)	18.9 (16.3 - 21.6)	11.0 (8.4 - 13.8)	4.8 (2.0 - 9.5)
Cardiovascular diseases	PAH	Male	75-84	2348	1.68 (1.81 - 1.54)	62.3 (60.3 - 64.3)	35.0 (32.9 - 37.1)	20.5 (18.6 - 22.4)	4.2 (3.1 - 5.7)	0.9 (0.3 - 2.1)	0.0 (0.0 - 0.0)
Cardiovascular diseases	PAH	Male	85-95	1034	0.8 (0.91 - 0.68)	45.8 (42.7 - 48.9)	18.0 (15.5 - 20.7)	6.5 (4.8 - 8.4)	0.4 (0.0 - 1.7)	0.0 (0.0 - 0.0)	0.0 (0.0 - 0.0)
Cardiovascular diseases	Peripheral artery	Male	All	98523	6.12 (6.18 - 6.05)	82.5 (82.2 - 82.7)	67.5 (67.2 - 67.8)	55.7 (55.4 - 56.0)	33.9 (33.6 - 34.3)	20.3 (20.0 - 20.6)	11.8 (11.5 - 12.2)
Cardiovascular diseases	Peripheral artery	Male	16-24	510	NR	99.6 (98.4 - 99.9)	99.6 (98.4 - 99.9)	99.1 (97.6 - 99.7)	98.1 (95.9 - 99.1)	97.2 (94.7 - 98.6)	95.9 (91.1 - 98.1)
Cardiovascular diseases	Peripheral artery	Male	25-34	801	NR	99.4 (98.5 - 99.7)	98.2 (97.0 - 99.0)	97.6 (96.2 - 98.5)	96.2 (94.3 - 97.4)	94.1 (91.5 - 96.0)	92.6 (88.9 - 95.1)
Cardiovascular diseases	Peripheral artery	Male	35-44	1646	NR	98.3 (97.6 - 98.8)	96.1 (95.0 - 97.0)	94.6 (93.3 - 95.7)	91.2 (89.5 - 92.7)	86.0 (83.6 - 88.1)	81.8 (78.7 - 84.6)
Cardiovascular diseases	Peripheral artery	Male	45-54	4610	20.25 (20.82 - 19.59)	96.5 (95.9 - 97.0)	91.8 (91.0 - 92.6)	87.8 (86.8 - 88.8)	76.9 (75.5 - 78.2)	64.5 (62.7 - 66.2)	50.7 (48.4 - 53.0)
Cardiovascular diseases	Peripheral artery	Male	55-64	15244	13.19 (13.42 - 12.95)	94.3 (93.9 - 94.6)	87.1 (86.6 - 87.7)	79.9 (79.2 - 80.5)	61.3 (60.4 - 62.1)	43.7 (42.7 - 44.7)	26.7 (25.5 - 27.8)
Cardiovascular diseases	Peripheral artery	Male	65-74	29522	8.13 (8.26 - 8.01)	89.7 (89.3 - 90.0)	77.3 (76.8 - 77.7)	66.4 (65.8 - 67.0)	40.8 (40.1 - 41.4)	20.1 (19.5 - 20.8)	6.7 (6.1 - 7.3)
Cardiovascular diseases	Peripheral artery	Male	75-84	31986	4.1 (4.17 - 4.02)	78.5 (78.1 - 79.0)	59.3 (58.7 - 59.9)	43.4 (42.8 - 44.0)	15.7 (15.2 - 16.2)	3.7 (3.4 - 4.0)	0.4 (0.3 - 0.7)
Cardiovascular diseases	Peripheral artery	Male	85-95	13747	1.41 (1.46 - 1.35)	56.7 (55.9 - 57.5)	30.2 (29.4 - 31.0)	15.5 (14.8 - 16.1)	1.7 (1.5 - 2.0)	0.1 (0.1 - 0.3)	0.0 (0.0 - 0.2)
Cardiovascular diseases	Rheumatic heart disease	Male	All	13526	5.99 (6.22 - 5.73)	80.8 (80.1 - 81.5)	65.3 (64.5 - 66.2)	54.2 (53.3 - 55.2)	37.2 (36.2 - 38.2)	27.0 (25.9 - 28.0)	19.5 (18.4 - 20.7)
Cardiovascular diseases	Rheumatic heart disease	Male	16-24	187	NR	99.5 (96.2 - 99.9)	97.1 (93.2 - 98.8)	94.6 (89.9 - 97.2)	92.9 (87.5 - 96.1)	89.7 (82.8 - 93.9)	89.7 (82.8 - 93.9)
Cardiovascular diseases	Rheumatic heart disease	Male	25-34	256	NR	99.2 (96.9 - 99.8)	96.6 (93.4 - 98.3)	94.7 (90.9 - 97.0)	91.8 (87.1 - 94.9)	89.2 (83.3 - 93.0)	86.5 (79.3 - 91.3)
Cardiovascular diseases	Rheumatic heart disease	Male	35-44	409	NR	97.3 (95.1 - 98.5)	95.5 (92.9 - 97.1)	92.8 (89.6 - 95.0)	88.7 (84.8 - 91.6)	85.0 (80.4 - 88.7)	78.5 (72.2 - 83.6)
Cardiovascular diseases	Rheumatic heart disease	Male	45-54	745	NR	94.7 (92.8 - 96.1)	90.1 (87.7 - 92.1)	87.2 (84.5 - 89.5)	79.1 (75.6 - 82.2)	72.2 (68.1 - 75.9)	62.2 (56.8 - 67.0)
Cardiovascular diseases	Rheumatic heart disease	Male	55-64	1588	15.5 (16.59 - 13.95)	91.4 (89.9 - 92.7)	83.6 (81.6 - 85.4)	77.8 (75.5 - 79.9)	65.0 (62.2 - 67.7)	50.9 (47.7 - 54.0)	35.8 (31.9 - 39.6)
Cardiovascular diseases	Rheumatic heart disease	Male	65-74	3143	8.41 (8.97 - 7.85)	86.4 (85.2 - 87.6)	74.3 (72.6 - 75.8)	64.5 (62.6 - 66.3)	42.9 (40.7 - 45.1)	24.8 (22.4 - 27.2)	9.8 (7.6 - 12.3)
Cardiovascular diseases	Rheumatic heart disease	Male	75-84	5008	4.18 (4.32 - 3.97)	78.6 (77.4 - 79.7)	59.0 (57.5 - 60.4)	42.1 (40.5 - 43.7)	15.9 (14.4 - 17.4)	4.3 (3.3 - 5.4)	0.5 (0.2 - 1.2)
Cardiovascular diseases	Rheumatic heart disease	Male	85-95	2136	1.45 (1.56 - 1.33)	58.4 (56.2 - 60.5)	29.7 (27.5 - 31.9)	15.0 (13.2 - 16.9)	2.4 (1.5 - 3.7)	0.0 (0.0 - 0.0)	0.0 (0.0 - 0.0)
Cardiovascular diseases	Stroke	Male	All	399130	8.27 (8.32 - 8.22)	83.4 (83.3 - 83.5)	72.5 (72.4 - 72.7)	63.0 (62.9 - 63.2)	44.1 (43.9 - 44.2)	30.3 (30.1 - 30.5)	20.3 (20.1 - 20.5)
Cardiovascular diseases	Stroke	Male	16-24	1930	NR	95.1 (94.0 - 96.0)	93.7 (92.5 - 94.7)	93.0 (91.8 - 94.1)	91.0 (89.5 - 92.2)	88.9 (87.1 - 90.5)	86.6 (84.1 - 88.8)
Cardiovascular diseases	Stroke	Male	25-34	3578	NR	95.3 (94.6 - 96.0)	94.1 (93.2 - 94.8)	93.1 (92.1 - 93.9)	90.7 (89.5 - 91.7)	88.3 (86.9 - 89.5)	85.5 (83.6 - 87.2)
Cardiovascular diseases	Stroke	Male	35-44	9498	NR	95.4 (95.0 - 95.8)	93.8 (93.3 - 94.3)	92.5 (92.0 - 93.1)	89.1 (88.4 - 89.8)	85.6 (84.7 - 86.4)	80.0 (78.6 - 81.3)
Cardiovascular diseases	Stroke	Male	45-54	28509	NR	94.5 (94.3 - 94.8)	92.1 (91.7 - 92.4)	89.9 (89.5 - 90.2)	84.0 (83.6 - 84.5)	76.5 (75.9 - 77.2)	66.6 (65.6 - 67.5)
Cardiovascular diseases	Stroke	Male	55-64	67989	18.31 (18.46 - 18.11)	92.9 (92.7 - 93.1)	88.9 (88.6 - 89.1)	85.1 (84.8 - 85.3)	73.9 (73.5 - 74.2)	60.5 (60.0 - 61.0)	44.2 (43.5 - 44.8)
Cardiovascular diseases	Stroke	Male	65-74	111057	10.91 (10.98 - 10.83)	88.9 (88.7 - 89.1)	81.7 (81.5 - 82.0)	74.1 (73.8 - 74.4)	53.9 (53.5 - 54.2)	32.5 (32.1 - 32.9)	14.8 (14.3 - 15.2)
Cardiovascular diseases	Stroke	Male	75-84	121444	4.97 (5.02 - 4.93)	79.2 (79.0 - 79.5)	64.1 (63.8 - 64.4)	49.8 (49.5 - 50.1)	21.3 (21.1 - 21.6)	6.0 (5.8 - 6.2)	0.9 (0.8 - 1.1)
Cardiovascular diseases	Stroke	Male	85-95	53461	1.86 (1.89 - 1.82)	61.5 (61.0 - 61.9)	37.2 (36.7 - 37.6)	20.6 (20.3 - 21.0)	3.0 (2.8 - 3.2)	0.2 (0.2 - 0.3)	0.0 (0.0 - 0.1)
Cardiovascular diseases	Subarachnoid hem	Male	All	41257	7.21 (7.38 - 7.03)	77.7 (77.3 - 78.1)	66.3 (65.9 - 66.8)	57.6 (57.1 - 58.1)	42.5 (41.9 - 43.0)	33.2 (32.7 - 33.8)	26.4 (25.6 - 27.1)
Cardiovascular diseases	Subarachnoid hem	Male	16-24	553	NR	95.8 (93.8 - 97.2)	94.7 (92.4 - 96.3)	94.3 (91.9 - 95.9)	92.5 (89.9 - 94.5)	89.5 (86.0 - 92.2)	86.3 (80.7 - 90.3)
Cardiovascular diseases	Subarachnoid hem	Male	25-34	895	NR	96.7 (95.4 - 97.7)	95.1 (93.4 - 96.3)	94.4 (92.6 - 95.8)	92.6 (90.5 - 94.2)	90.2 (87.6 - 92.4)	87.9 (84.3 - 90.7)
Cardiovascular diseases	Subarachnoid hem	Male	35-44	1891	NR	94.3 (93.1 - 95.2)	92.2 (90.9 - 93.3)	91.0 (89.5 - 92.2)	87.3 (85.6 - 88.9)	84.3 (82.3 - 86.1)	78.7 (75.8 - 81.3)
Cardiovascular diseases	Subarachnoid hem	Male	45-54	3991	NR	92.2 (91.3 - 93.0)	89.3 (88.3 - 90.2)	86.8 (85.7 - 87.8)	80.9 (79.6 - 82.2)	73.9 (72.2 - 75.5)	65.9 (63.6 - 68.2)
Cardiovascular diseases	Subarachnoid hem	Male	55-64	6737	16.53 (17.14 - 15.74)	88.1 (87.3 - 88.8)	82.2 (81.2 - 83.1)	77.1 (76.1 - 78.2)	65.3 (64.0 - 66.6)	53.5 (52.0 - 55.1)	39.7 (37.6 - 41.8)
Cardiovascular diseases	Subarachnoid hem	Male	65-74	9718	8.94 (9.2 - 8.58)	82.6 (81.9 - 83.4)	73.1 (72.2 - 74.0)	64.8 (63.8 - 65.8)	45.3 (44.1 - 46.5)	27.8 (26.4 - 29.2)	13.9 (12.2 - 15.7)
Cardiovascular diseases	Subarachnoid hem	Male	75-84	11193	3.69 (3.84 - 3.55)	71.1 (70.2 - 71.9)	55.2 (54.2 - 56.1)	41.5 (40.5 - 42.4)	16.5 (15.6 - 17.4)	5.0 (4.3 - 5.7)	1.3 (0.8 - 2.0)
Cardiovascular diseases	Subarachnoid hem	Male	85-95	6043	1.18 (1.28 - 1.1)	53.0 (51.7 - 54.2)	29.5 (28.3 - 30.7)	15.2 (14.2 - 16.2)	1.7 (1.3 - 2.2)	0.2 (0.1 - 0.5)	0.0 (0.0 - 0.0)
Chronic respiratory	Asthma	Male	All	154898	NR	95.6 (95.5 - 95.7)	91.1 (90.9 - 91.2)	87.2 (87.0 - 87.4)	78.6 (78.3 - 78.8)	71.0 (70.7 - 71.3)	64.3 (64.0 - 64.7)
Chronic respiratory	Asthma	Male	16-24	18233	NR	99.9 (99.8 - 99.9)	99.8 (99.7 - 99.8)	99.5 (99.4 - 99.6)	99.1 (98.9 - 99.2)	98.6 (98.3 - 98.7)	98.1 (97.8 - 98.3)
Chronic respiratory	Asthma	Male	25-34	18644	NR	99.8 (99.7 - 99.9)	99.5 (99.4 - 99.6)	99.1 (99.0 - 99.3)	98.5 (98.3 - 98.7)	97.6 (97.3 - 97.8)	96.5 (96.1 - 96.9)
Chronic respiratory	Asthma	Male	35-44	21313	NR	99.6 (99.6 - 99.7)	99.2 (99.0 - 99.3)	98.7 (98.5 - 98.9)	97.3 (97.1 - 97.6)	95.2 (94.8 - 95.5)	92.3 (91.7 - 92.9)

Appendix Table A6: Unadjusted Median Survival and Survival Probabilities by Age, males (Page 9 of 18)

Group	Diagnosis Category	Sex	Age	N	Median Survival (y)	1y (%)	3y (%)	5y (%)	10y (%)	15y (%)	20y (%)
Chronic respiratory	Asthma	Male	45-54	23314	NR	99.1 (99.0 - 99.2)	97.8 (97.6 - 97.9)	96.5 (96.3 - 96.8)	93.3 (92.9 - 93.6)	88.2 (87.7 - 88.8)	81.3 (80.3 - 82.1)
Chronic respiratory	Asthma	Male	55-64	25395	22.62	97.5 (97.3 - 97.7)	94.6 (94.3 - 94.9)	91.9 (91.6 - 92.3)	83.7 (83.2 - 84.3)	73.1 (72.4 - 73.8)	58.8 (57.7 - 59.9)
Chronic respiratory	Asthma	Male	65-74	23652	13.31 (13.47 - 13.11)	94.5 (94.2 - 94.8)	88.1 (87.6 - 88.5)	81.9 (81.3 - 82.4)	64.0 (63.3 - 64.7)	42.7 (41.8 - 43.6)	21.5 (20.4 - 22.7)
Chronic respiratory	Asthma	Male	75-84	17802	6.14 (6.26 - 6.01)	86.3 (85.8 - 86.8)	71.7 (71.0 - 72.4)	58.2 (57.4 - 58.9)	27.2 (26.4 - 28.0)	8.6 (8.0 - 9.3)	1.4 (1.0 - 1.9)
Chronic respiratory	Asthma	Male	85-95	6359	2.28 (2.39 - 2.16)	68.4 (67.2 - 69.5)	41.1 (39.9 - 42.4)	23.0 (21.9 - 24.1)	3.5 (2.9 - 4.1)	0.2 (0.1 - 0.6)	0.0 (0.0 - 0.0)
Chronic respiratory	COPD	Male	All	179456	6.11 (6.16 - 6.06)	81.7 (81.6 - 81.9)	66.9 (66.7 - 67.1)	55.4 (55.1 - 55.6)	35.0 (34.7 - 35.2)	22.4 (22.2 - 22.7)	14.9 (14.6 - 15.2)
Chronic respiratory	COPD	Male	16-24	1011	NR	98.3 (97.3 - 98.9)	96.6 (95.2 - 97.6)	95.9 (94.4 - 97.0)	93.5 (91.5 - 94.9)	91.5 (89.1 - 93.3)	89.8 (86.7 - 92.2)
Chronic respiratory	COPD	Male	25-34	1418	NR	98.4 (97.6 - 99.0)	96.9 (95.9 - 97.7)	95.8 (94.6 - 96.8)	93.5 (92.0 - 94.8)	91.6 (89.8 - 93.2)	88.8 (86.3 - 91.0)
Chronic respiratory	COPD	Male	35-44	3284	NR	98.4 (97.9 - 98.8)	96.3 (95.6 - 96.9)	94.5 (93.7 - 95.3)	90.7 (89.6 - 91.7)	85.3 (83.8 - 86.7)	79.2 (77.1 - 81.2)
Chronic respiratory	COPD	Male	45-54	10397	21.96 (22.42 - 21.1)	95.9 (95.5 - 96.3)	91.4 (90.8 - 91.9)	87.4 (86.7 - 88.1)	77.6 (76.7 - 78.4)	66.5 (65.3 - 67.6)	53.7 (52.2 - 55.2)
Chronic respiratory	COPD	Male	55-64	31102	12.9 (13.06 - 12.68)	91.6 (91.3 - 92.0)	83.7 (83.3 - 84.1)	76.6 (76.1 - 77.1)	59.4 (58.8 - 60.0)	43.2 (42.5 - 43.9)	28.8 (27.9 - 29.6)
Chronic respiratory	COPD	Male	65-74	55926	7.46 (7.56 - 7.37)	85.7 (85.4 - 86.0)	73.2 (72.9 - 73.6)	62.4 (62.0 - 62.8)	37.9 (37.5 - 38.4)	18.7 (18.3 - 19.2)	7.1 (6.6 - 7.6)
Chronic respiratory	COPD	Male	75-84	56449	3.73 (3.78 - 3.68)	76.4 (76.1 - 76.8)	56.3 (55.9 - 56.8)	39.8 (39.4 - 40.3)	13.5 (13.2 - 13.9)	3.0 (2.8 - 3.2)	0.5 (0.4 - 0.7)
Chronic respiratory	COPD	Male	85-95	19403	1.47 (1.51 - 1.43)	58.2 (57.5 - 58.9)	30.5 (29.8 - 31.2)	15.3 (14.7 - 15.8)	1.8 (1.6 - 2.1)	0.1 (0.1 - 0.2)	0.0 (0.0 - 0.0)
Chronic respiratory	Interstitial lung disease	Male	All	38468	7.67 (7.88 - 7.41)	81.4 (81.0 - 81.8)	67.4 (66.9 - 67.9)	58.2 (57.7 - 58.7)	45.2 (44.6 - 45.8)	38.5 (37.9 - 39.1)	33.5 (32.9 - 34.2)
Chronic respiratory	Interstitial lung disease	Male	16-24	315	NR	96.8 (94.1 - 98.3)	96.1 (93.3 - 97.8)	95.4 (92.4 - 97.3)	94.5 (91.2 - 96.6)	93.8 (90.1 - 96.2)	93.8 (90.1 - 96.2)
Chronic respiratory	Interstitial lung disease	Male	25-34	2058	NR	99.3 (98.8 - 99.6)	98.7 (98.1 - 99.1)	98.3 (97.6 - 98.8)	97.4 (96.5 - 98.0)	95.7 (94.6 - 96.6)	94.2 (92.7 - 95.4)
Chronic respiratory	Interstitial lung disease	Male	35-44	3459	NR	99.0 (98.6 - 99.3)	98.2 (97.7 - 98.6)	97.7 (97.1 - 98.1)	96.2 (95.4 - 96.8)	93.8 (92.8 - 94.7)	90.6 (89.1 - 92.0)
Chronic respiratory	Interstitial lung disease	Male	45-54	3894	NR	97.3 (96.8 - 97.8)	94.9 (94.1 - 95.5)	92.9 (92.0 - 93.7)	87.3 (86.1 - 88.5)	82.3 (80.7 - 83.7)	74.5 (72.2 - 76.6)
Chronic respiratory	Interstitial lung disease	Male	55-64	5543	14.84 (15.64 - 14.2)	91.7 (91.0 - 92.4)	84.0 (83.0 - 85.0)	77.4 (76.2 - 78.6)	63.2 (61.7 - 64.6)	49.6 (47.8 - 51.3)	37.6 (35.5 - 39.7)
Chronic respiratory	Interstitial lung disease	Male	65-74	8846	5.82 (6.02 - 5.6)	83.4 (82.6 - 84.2)	67.3 (66.2 - 68.3)	54.3 (53.2 - 55.4)	31.0 (29.8 - 32.2)	16.7 (15.5 - 18.0)	6.9 (5.8 - 8.2)
Chronic respiratory	Interstitial lung disease	Male	75-84	10707	2.59 (2.7 - 2.5)	70.2 (69.3 - 71.0)	45.8 (44.8 - 46.8)	29.3 (28.3 - 30.3)	8.2 (7.4 - 8.9)	2.3 (1.8 - 2.9)	0.5 (0.2 - 0.9)
Chronic respiratory	Interstitial lung disease	Male	85-95	3582	0.91 (0.99 - 0.83)	48.0 (46.4 - 49.7)	20.5 (19.0 - 21.9)	8.7 (7.7 - 9.9)	1.0 (0.6 - 1.6)	0.1 (0.0 - 0.6)	0.1 (0.0 - 0.6)
Chronic respiratory	Pneumoconiosis	Male	All	7307	6.86 (7.14 - 6.53)	82.7 (81.8 - 83.5)	68.6 (67.5 - 69.6)	58.0 (56.8 - 59.2)	39.0 (37.8 - 40.1)	25.9 (24.7 - 27.0)	17.2 (16.1 - 18.4)
Chronic respiratory	Pneumoconiosis	Male	16-24	18	NR	100.0 (100.0 - 100.0)	100.0 (100.0 - 100.0)	100.0 (100.0 - 100.0)	92.3 (56.6 - 98.9)	92.3 (56.6 - 98.9)	92.3 (56.6 - 98.9)
Chronic respiratory	Pneumoconiosis	Male	25-34	20	NR	100.0 (100.0 - 100.0)	100.0 (100.0 - 100.0)	100.0 (100.0 - 100.0)	94.7 (68.1 - 99.2)	94.7 (68.1 - 99.2)	94.7 (68.1 - 99.2)
Chronic respiratory	Pneumoconiosis	Male	35-44	52	NR	100.0 (100.0 - 100.0)	98.0 (86.9 - 99.7)	98.0 (86.9 - 99.7)	95.9 (84.6 - 99.0)	93.6 (81.3 - 97.9)	86.1 (68.6 - 94.3)
Chronic respiratory	Pneumoconiosis	Male	45-54	171	NR	99.4 (95.9 - 99.9)	98.2 (94.5 - 99.4)	97.0 (92.9 - 98.7)	88.8 (82.6 - 92.9)	76.7 (68.5 - 83.0)	71.5 (62.5 - 78.6)
Chronic respiratory	Pneumoconiosis	Male	55-64	1071	20.11 (21.04 - 18.61)	95.8 (94.4 - 96.8)	91.0 (89.1 - 92.5)	86.9 (84.7 - 88.8)	75.9 (73.2 - 78.4)	63.0 (59.9 - 66.0)	50.8 (47.1 - 54.3)
Chronic respiratory	Pneumoconiosis	Male	65-74	2150	10.75 (11.37 - 10.07)	90.2 (88.8 - 91.3)	80.2 (78.4 - 81.8)	72.1 (70.1 - 74.0)	52.5 (50.2 - 54.7)	32.9 (30.6 - 35.3)	14.8 (12.6 - 17.3)
Chronic respiratory	Pneumoconiosis	Male	75-84	2860	4.39 (4.65 - 4.16)	78.9 (77.4 - 80.4)	60.8 (58.9 - 62.6)	45.7 (43.8 - 47.6)	19.1 (17.5 - 20.8)	5.5 (4.4 - 6.6)	0.2 (0.0 - 1.2)
Chronic respiratory	Pneumoconiosis	Male	85-95	948	1.5 (1.82 - 1.29)	57.4 (54.2 - 60.5)	31.4 (28.4 - 34.5)	16.2 (13.7 - 18.8)	2.4 (1.4 - 3.9)	0.2 (0.0 - 0.9)	0.0 (0.0 - 0.0)
Chronic respiratory	Sleep Apnea	Male	All	205146	NR	98.6 (98.6 - 98.7)	96.4 (96.3 - 96.4)	93.8 (93.7 - 93.9)	86.9 (86.8 - 87.1)	78.8 (78.6 - 79.1)	69.5 (69.1 - 69.9)
Chronic respiratory	Sleep Apnea	Male	16-24	3358	NR	99.8 (99.5 - 99.9)	99.3 (99.0 - 99.6)	99.0 (98.5 - 99.3)	98.0 (97.3 - 98.5)	97.0 (96.1 - 97.7)	96.7 (95.7 - 97.5)
Chronic respiratory	Sleep Apnea	Male	25-34	14868	NR	99.8 (99.8 - 99.9)	99.5 (99.4 - 99.6)	99.1 (98.9 - 99.2)	98.3 (98.0 - 98.5)	97.1 (96.7 - 97.5)	95.5 (94.7 - 96.1)
Chronic respiratory	Sleep Apnea	Male	35-44	30536	NR	99.8 (99.8 - 99.9)	99.4 (99.3 - 99.5)	99.0 (98.9 - 99.1)	97.5 (97.3 - 97.7)	95.6 (95.3 - 95.9)	92.6 (92.0 - 93.2)
Chronic respiratory	Sleep Apnea	Male	45-54	50019	NR	99.6 (99.5 - 99.6)	98.8 (98.7 - 98.9)	97.8 (97.7 - 98.0)	95.0 (94.8 - 95.3)	90.8 (90.5 - 91.2)	84.6 (84.0 - 85.2)
Chronic respiratory	Sleep Apnea	Male	55-64	56021	NR	99.0 (99.0 - 99.1)	97.4 (97.2 - 97.5)	95.3 (95.1 - 95.5)	88.9 (88.6 - 89.2)	79.8 (79.4 - 80.3)	66.9 (66.2 - 67.7)
Chronic respiratory	Sleep Apnea	Male	65-74	36594	15.55 (15.75 - 15.35)	97.4 (97.3 - 97.6)	92.9 (92.7 - 93.2)	87.7 (87.4 - 88.1)	72.9 (72.3 - 73.4)	52.7 (51.9 - 53.5)	29.6 (28.3 - 30.9)
Chronic respiratory	Sleep Apnea	Male	75-84	12629	8.34 (8.56 - 8.17)	93.3 (92.8 - 93.7)	82.7 (82.0 - 83.4)	70.6 (69.7 - 71.5)	40.5 (39.4 - 41.7)	15.4 (14.1 - 16.7)	3.6 (2.5 - 4.9)
Chronic respiratory	Sleep Apnea	Male	85-95	1108	3.81 (4.1 - 3.53)	82.5 (80.1 - 84.7)	59.2 (56.0 - 62.3)	38.5 (35.1 - 41.9)	10.4 (7.9 - 13.3)	0.6 (0.1 - 2.8)	0.0 (0.0 - 0.0)
Diabetes & CKD	Acute glomerulonephritis	Male	All	2217	NR	92.8 (91.7 - 93.8)	88.6 (87.1 - 89.9)	84.0 (82.3 - 85.5)	73.9 (71.9 - 75.8)	64.4 (62.0 - 66.7)	54.8 (51.5 - 57.9)
Diabetes & CKD	Acute glomerulonephritis	Male	16-24	272	NR	100.0 (100.0 - 100.0)	100.0 (100.0 - 100.0)	98.8 (96.2 - 99.6)	97.8 (94.7 - 99.1)	97.1 (93.5 - 98.7)	97.1 (93.5 - 98.7)
Diabetes & CKD	Acute glomerulonephritis	Male	25-34	233	NR	98.7 (96.0 - 99.6)	97.8 (94.8 - 99.1)	96.0 (92.4 - 97.9)	94.5 (90.5 - 96.8)	94.5 (90.5 - 96.8)	93.1 (87.5 - 96.2)
Diabetes & CKD	Acute glomerulonephritis	Male	35-44	253	NR	99.6 (97.2 - 99.9)	99.2 (96.8 - 99.8)	98.3 (95.6 - 99.4)	95.1 (91.3 - 97.3)	92.7 (88.2 - 95.6)	80.0 (68.9 - 87.5)
Diabetes & CKD	Acute glomerulonephritis	Male	45-54	319	NR	96.5 (93.8 - 98.1)	94.6 (91.4 - 96.6)	93.2 (89.7 - 95.5)	89.0 (84.8 - 92.1)	84.8 (79.8 - 88.7)	70.5 (61.1 - 78.1)
Diabetes & CKD	Acute glomerulonephritis	Male	55-64	389	19.67	95.8 (93.2 - 97.4)	93.3 (90.2 - 95.4)	89.2 (85.5 - 92.0)	78.0 (73.1 - 82.1)	65.0 (59.1 - 70.4)	48.1 (38.7 - 56.9)
Diabetes & CKD	Acute glomerulonephritis	Male	65-74	399	10.62 (11.95 - 9.35)	90.2 (86.7 - 92.7)	83.5 (79.3 - 86.9)	74.6 (69.7 - 78.8)	53.5 (47.7 - 58.9)	28.5 (22.8 - 34.4)	8.9 (3.8 - 16.8)
Diabetes & CKD	Acute glomerulonephritis	Male	75-84	296	5.0 (5.78 - 4.21)	77.7 (72.3 - 82.2)	63.1 (56.9 - 68.6)	49.8 (43.3 - 55.9)	22.4 (16.9 - 28.5)	5.4 (2.3 - 10.2)	0.0 (0.0 - 0.0)
Diabetes & CKD	Acute glomerulonephritis	Male	85-95	56	1.53 (2.33 - 0.51)	53.7 (39.1 - 66.2)	34.1 (21.1 - 47.4)	24.8 (12.9 - 38.7)	12.4 (3.6 - 26.8)	4.1 (0.3 - 17.0)	4.1 (0.3 - 17.0)

Appendix Table A6: Unadjusted Median Survival and Survival Probabilities by Age, males (Page 10 of 18)

Group	Diagnosis Category	Sex	Age	N	Median Survival (y)	1y (%)	3y (%)	5y (%)	10y (%)	15y (%)	20y (%)
Diabetes & CKD	Chronic kidney disease	Male	All	268722	3.87 (3.91 - 3.83)	70.4 (70.2 - 70.5)	54.9 (54.7 - 55.1)	44.5 (44.3 - 44.7)	29.0 (28.8 - 29.2)	20.9 (20.7 - 21.1)	16.2 (16.0 - 16.5)
Diabetes & CKD	Chronic kidney disease	Male	16-24	4068	NR	97.9 (97.4 - 98.3)	96.7 (96.1 - 97.2)	96.1 (95.5 - 96.7)	94.2 (93.4 - 95.0)	92.8 (91.8 - 93.7)	91.5 (90.1 - 92.6)
Diabetes & CKD	Chronic kidney disease	Male	25-34	5984	NR	96.7 (96.3 - 97.2)	94.7 (94.1 - 95.3)	93.5 (92.9 - 94.2)	90.7 (89.8 - 91.5)	88.0 (86.9 - 89.0)	85.9 (84.6 - 87.1)
Diabetes & CKD	Chronic kidney disease	Male	35-44	8963	NR	94.9 (94.4 - 95.3)	92.2 (91.7 - 92.8)	90.5 (89.8 - 91.1)	86.4 (85.6 - 87.2)	81.0 (80.0 - 82.0)	75.0 (73.6 - 76.3)
Diabetes & CKD	Chronic kidney disease	Male	45-54	15357	20.76 (21.45 - 20.11)	90.2 (89.7 - 90.7)	85.7 (85.1 - 86.2)	81.9 (81.2 - 82.5)	72.4 (71.6 - 73.2)	62.6 (61.7 - 63.6)	51.5 (50.2 - 52.7)
Diabetes & CKD	Chronic kidney disease	Male	55-64	31104	11.03 (11.3 - 10.81)	84.4 (84.0 - 84.8)	76.6 (76.1 - 77.0)	69.5 (69.0 - 70.1)	53.1 (52.4 - 53.7)	38.5 (37.7 - 39.2)	25.9 (25.0 - 26.7)
Diabetes & CKD	Chronic kidney disease	Male	65-74	59887	5.87 (5.95 - 5.79)	77.4 (77.0 - 77.7)	64.8 (64.4 - 65.1)	54.2 (53.8 - 54.7)	32.0 (31.5 - 32.5)	15.1 (14.6 - 15.6)	6.0 (5.5 - 6.5)
Diabetes & CKD	Chronic kidney disease	Male	75-84	86443	2.63 (2.67 - 2.58)	66.5 (66.2 - 66.8)	46.8 (46.5 - 47.2)	32.0 (31.6 - 32.3)	9.9 (9.6 - 10.2)	2.2 (2.0 - 2.4)	0.3 (0.2 - 0.4)
Diabetes & CKD	Chronic kidney disease	Male	85-95	54395	0.88 (0.9 - 0.86)	47.6 (47.2 - 48.1)	22.8 (22.4 - 23.2)	10.0 (9.7 - 10.3)	0.9 (0.8 - 1.1)	0.1 (0.0 - 0.2)	0.0 (0.0 - 0.1)
Diabetes & CKD	Diabetes	Male	All	423694	13.63 (13.7 - 13.56)	91.8 (91.7 - 91.9)	84.2 (84.1 - 84.3)	77.2 (77.1 - 77.3)	60.9 (60.8 - 61.1)	46.2 (46.0 - 46.3)	34.5 (34.3 - 34.8)
Diabetes & CKD	Diabetes	Male	16-24	6327	NR	99.6 (99.5 - 99.8)	99.2 (99.0 - 99.4)	98.8 (98.5 - 99.0)	97.8 (97.3 - 98.1)	96.9 (96.3 - 97.3)	95.6 (94.8 - 96.2)
Diabetes & CKD	Diabetes	Male	25-34	10818	NR	99.4 (99.2 - 99.5)	98.8 (98.6 - 99.0)	98.1 (97.8 - 98.3)	96.5 (96.1 - 96.9)	94.9 (94.3 - 95.3)	92.3 (91.6 - 92.9)
Diabetes & CKD	Diabetes	Male	35-44	23509	NR	99.0 (98.9 - 99.1)	97.9 (97.7 - 98.1)	96.7 (96.4 - 96.9)	93.3 (92.9 - 93.6)	88.6 (88.0 - 89.1)	82.6 (81.9 - 83.3)
Diabetes & CKD	Diabetes	Male	45-54	53511	NR	97.9 (97.8 - 98.0)	95.9 (95.7 - 96.0)	93.8 (93.6 - 94.0)	87.5 (87.2 - 87.8)	78.5 (78.0 - 78.9)	66.8 (66.1 - 67.4)
Diabetes & CKD	Diabetes	Male	55-64	100554	18.58 (18.72 - 18.45)	96.1 (96.0 - 96.2)	92.3 (92.2 - 92.5)	88.6 (88.4 - 88.8)	77.4 (77.1 - 77.6)	62.4 (62.0 - 62.8)	44.6 (44.1 - 45.1)
Diabetes & CKD	Diabetes	Male	65-74	120644	11.7 (11.78 - 11.63)	92.6 (92.5 - 92.8)	85.6 (85.4 - 85.8)	78.6 (78.3 - 78.8)	58.1 (57.8 - 58.4)	34.0 (33.6 - 34.4)	14.3 (13.9 - 14.7)
Diabetes & CKD	Diabetes	Male	75-84	84654	5.82 (5.87 - 5.77)	85.0 (84.7 - 85.2)	69.9 (69.6 - 70.3)	55.7 (55.3 - 56.0)	24.8 (24.4 - 25.1)	6.5 (6.2 - 6.7)	1.0 (0.8 - 1.2)
Diabetes & CKD	Diabetes	Male	85-95	23219	2.26 (2.31 - 2.21)	67.8 (67.2 - 68.4)	41.0 (40.3 - 41.6)	22.6 (22.0 - 23.2)	3.1 (2.9 - 3.4)	0.2 (0.1 - 0.3)	0.0 (0.0 - 0.1)
Diabetes & CKD	Diabetes type 1	Male	All	102331	12.79 (12.91 - 12.65)	90.2 (90.1 - 90.4)	81.1 (80.9 - 81.3)	73.3 (73.0 - 73.6)	57.4 (57.1 - 57.7)	44.8 (44.5 - 45.2)	36.0 (35.6 - 36.3)
Diabetes & CKD	Diabetes type 1	Male	16-24	5113	NR	99.8 (99.7 - 99.9)	99.5 (99.3 - 99.7)	99.2 (98.9 - 99.4)	98.3 (97.8 - 98.6)	97.4 (96.9 - 97.9)	96.2 (95.4 - 96.8)
Diabetes & CKD	Diabetes type 1	Male	25-34	6705	NR	99.6 (99.5 - 99.8)	99.1 (98.8 - 99.3)	98.5 (98.2 - 98.8)	97.3 (96.8 - 97.7)	95.7 (95.1 - 96.3)	93.5 (92.7 - 94.2)
Diabetes & CKD	Diabetes type 1	Male	35-44	8732	NR	99.0 (98.7 - 99.1)	97.9 (97.5 - 98.2)	96.4 (95.9 - 96.7)	92.5 (91.9 - 93.1)	87.9 (87.1 - 88.6)	82.8 (81.8 - 83.7)
Diabetes & CKD	Diabetes type 1	Male	45-54	13258	NR	97.3 (97.0 - 97.6)	94.1 (93.7 - 94.5)	91.2 (90.7 - 91.6)	82.8 (82.2 - 83.5)	72.6 (71.8 - 73.4)	61.2 (60.2 - 62.2)
Diabetes & CKD	Diabetes type 1	Male	55-64	21597	15.48 (15.69 - 15.25)	94.6 (94.3 - 94.9)	88.8 (88.4 - 89.2)	83.4 (82.9 - 83.9)	68.3 (67.6 - 68.9)	51.6 (50.9 - 52.4)	35.1 (34.2 - 35.9)
Diabetes & CKD	Diabetes type 1	Male	65-74	23789	8.89 (9.03 - 8.73)	89.4 (89.0 - 89.8)	78.9 (78.3 - 79.4)	68.6 (68.0 - 69.2)	45.0 (44.3 - 45.7)	23.5 (22.9 - 24.1)	9.1 (8.6 - 9.7)
Diabetes & CKD	Diabetes type 1	Male	75-84	18124	4.09 (4.17 - 3.98)	79.0 (78.4 - 79.6)	59.3 (58.6 - 60.0)	43.1 (42.4 - 43.9)	15.8 (15.3 - 16.4)	3.7 (3.3 - 4.0)	0.6 (0.4 - 0.8)
Diabetes & CKD	Diabetes type 1	Male	85-95	4921	1.59 (1.7 - 1.51)	60.4 (59.0 - 61.8)	32.1 (30.8 - 33.4)	15.4 (14.3 - 16.4)	1.8 (1.4 - 2.2)	0.1 (0.0 - 0.3)	0.0 (0.0 - 0.0)
Diabetes & CKD	Diabetes type 2	Male	All	395070	12.3 (12.36 - 12.23)	91.3 (91.2 - 91.4)	82.9 (82.7 - 83.0)	75.2 (75.1 - 75.4)	57.6 (57.4 - 57.8)	41.7 (41.5 - 41.9)	29.0 (28.7 - 29.2)
Diabetes & CKD	Diabetes type 2	Male	16-24	1859	NR	99.4 (98.9 - 99.7)	98.7 (98.0 - 99.1)	97.8 (97.0 - 98.4)	95.7 (94.5 - 96.6)	93.2 (91.5 - 94.6)	90.6 (87.8 - 92.8)
Diabetes & CKD	Diabetes type 2	Male	25-34	5883	NR	99.3 (99.0 - 99.4)	98.3 (97.9 - 98.6)	96.9 (96.4 - 97.4)	94.6 (93.9 - 95.3)	91.8 (90.8 - 92.6)	87.8 (86.2 - 89.1)
Diabetes & CKD	Diabetes type 2	Male	35-44	18369	NR	98.9 (98.7 - 99.0)	97.6 (97.4 - 97.8)	96.2 (95.8 - 96.4)	92.0 (91.5 - 92.4)	85.9 (85.2 - 86.6)	78.0 (76.9 - 79.0)
Diabetes & CKD	Diabetes type 2	Male	45-54	47904	NR	97.8 (97.7 - 97.9)	95.6 (95.4 - 95.7)	93.3 (93.0 - 93.5)	86.1 (85.7 - 86.4)	76.2 (75.7 - 76.7)	63.3 (62.6 - 64.1)
Diabetes & CKD	Diabetes type 2	Male	55-64	94959	17.93 (18.07 - 17.79)	96.1 (95.9 - 96.2)	92.0 (91.8 - 92.2)	88.0 (87.8 - 88.2)	75.9 (75.6 - 76.2)	60.3 (59.9 - 60.7)	42.3 (41.7 - 42.9)
Diabetes & CKD	Diabetes type 2	Male	65-74	117869	11.38 (11.45 - 11.29)	92.5 (92.4 - 92.7)	85.1 (84.9 - 85.3)	77.7 (77.5 - 78.0)	56.6 (56.2 - 56.9)	32.3 (31.9 - 32.7)	13.2 (12.8 - 13.6)
Diabetes & CKD	Diabetes type 2	Male	75-84	84472	5.67 (5.72 - 5.62)	84.8 (84.5 - 85.0)	69.2 (68.9 - 69.5)	54.6 (54.3 - 55.0)	23.7 (23.4 - 24.1)	6.0 (5.8 - 6.3)	0.9 (0.7 - 1.1)
Diabetes & CKD	Diabetes type 2	Male	85-95	23285	2.21 (2.26 - 2.16)	67.5 (66.9 - 68.1)	40.4 (39.7 - 41.1)	22.0 (21.4 - 22.6)	2.9 (2.6 - 3.2)	0.2 (0.1 - 0.3)	0.0 (0.0 - 0.1)
Digestive diseases	Cirrhosis	Male	All	90886	17.18 (17.48 - 16.9)	87.0 (86.7 - 87.2)	78.9 (78.6 - 79.1)	73.1 (72.8 - 73.4)	61.9 (61.6 - 62.3)	53.2 (52.8 - 53.6)	46.2 (45.7 - 46.7)
Digestive diseases	Cirrhosis	Male	16-24	5184	NR	98.6 (98.3 - 98.9)	96.6 (96.0 - 97.0)	95.1 (94.4 - 95.6)	91.4 (90.5 - 92.2)	87.7 (86.6 - 88.8)	84.3 (82.8 - 85.6)
Digestive diseases	Cirrhosis	Male	25-34	11658	NR	98.3 (98.1 - 98.6)	96.4 (96.0 - 96.7)	94.6 (94.2 - 95.0)	90.2 (89.6 - 90.8)	86.6 (85.8 - 87.3)	82.0 (80.9 - 83.0)
Digestive diseases	Cirrhosis	Male	35-44	14288	NR	97.6 (97.4 - 97.9)	95.3 (94.9 - 95.6)	93.0 (92.6 - 93.4)	87.3 (86.7 - 87.9)	80.7 (79.9 - 81.4)	74.4 (73.4 - 75.3)
Digestive diseases	Cirrhosis	Male	45-54	17289	19.93 (20.35 - 19.29)	92.8 (92.4 - 93.1)	86.9 (86.4 - 87.4)	82.3 (81.7 - 82.9)	70.9 (70.1 - 71.6)	59.9 (59.0 - 60.7)	49.9 (48.9 - 50.9)
Digestive diseases	Cirrhosis	Male	55-64	18152	10.42 (10.7 - 10.1)	85.0 (84.5 - 85.5)	75.1 (74.5 - 75.7)	67.3 (66.6 - 68.0)	51.1 (50.2 - 51.9)	37.8 (36.9 - 38.8)	27.0 (25.9 - 28.1)
Digestive diseases	Cirrhosis	Male	65-74	14956	5.15 (5.36 - 4.96)	75.8 (75.1 - 76.5)	61.2 (60.4 - 62.1)	50.7 (49.9 - 51.6)	31.4 (30.5 - 32.3)	17.7 (16.7 - 18.7)	8.0 (7.0 - 9.1)
Digestive diseases	Cirrhosis	Male	75-84	7595	2.24 (2.38 - 2.13)	63.6 (62.5 - 64.7)	43.5 (42.3 - 44.7)	31.0 (29.8 - 32.2)	12.8 (11.8 - 13.8)	3.8 (3.1 - 4.5)	0.7 (0.4 - 1.4)
Digestive diseases	Cirrhosis	Male	85-95	1738	0.84 (0.97 - 0.68)	47.1 (44.7 - 49.4)	24.2 (22.1 - 26.4)	12.2 (10.6 - 14.1)	2.0 (1.3 - 3.0)	0.3 (0.1 - 1.0)	0.3 (0.1 - 1.0)
Digestive diseases	Crohn's disease	Male	All	25804	NR	97.4 (97.2 - 97.6)	94.4 (94.1 - 94.7)	91.8 (91.5 - 92.2)	85.5 (85.0 - 86.0)	79.0 (78.4 - 79.6)	72.3 (71.5 - 73.1)
Digestive diseases	Crohn's disease	Male	16-24	4269	NR	100.0 (99.8 - 100.0)	99.7 (99.5 - 99.9)	99.4 (99.1 - 99.6)	98.8 (98.4 - 99.1)	98.0 (97.4 - 98.5)	96.7 (95.6 - 97.5)
Digestive diseases	Crohn's disease	Male	25-34	4868	NR	99.8 (99.6 - 99.9)	99.5 (99.2 - 99.7)	99.1 (98.8 - 99.3)	98.1 (97.6 - 98.5)	96.9 (96.3 - 97.5)	94.9 (93.8 - 95.8)
Digestive diseases	Crohn's disease	Male	35-44	4060	NR	99.6 (99.3 - 99.7)	98.9 (98.6 - 99.2)	98.4 (98.0 - 98.8)	96.5 (95.8 - 97.1)	94.2 (93.3 - 95.1)	91.9 (90.6 - 93.0)
Digestive diseases	Crohn's disease	Male	45-54	3846	NR	99.2 (98.9 - 99.4)	98.3 (97.9 - 98.7)	97.1 (96.5 - 97.6)	93.4 (92.5 - 94.3)	88.4 (87.1 - 89.6)	80.8 (78.9 - 82.6)

Appendix Table A6: Unadjusted Median Survival and Survival Probabilities by Age, males (Page 11 of 18)

Group	Diagnosis Category	Sex	Age	N	Median Survival (y)	1y (%)	3y (%)	5y (%)	10y (%)	15y (%)	20y (%)
Digestive diseases	Crohn's disease	Male	55-64	3901	22.46	97.5 (97.0 - 97.9)	93.7 (92.9 - 94.5)	90.5 (89.5 - 91.4)	82.6 (81.2 - 83.9)	72.2 (70.4 - 73.9)	57.9 (55.5 - 60.3)
Digestive diseases	Crohn's disease	Male	65-74	2878	13.07 (13.61 - 12.57)	93.9 (93.0 - 94.7)	87.4 (86.1 - 88.6)	81.1 (79.5 - 82.6)	63.9 (61.8 - 65.9)	41.9 (39.3 - 44.5)	23.3 (20.4 - 26.4)
Digestive diseases	Crohn's disease	Male	75-84	1606	6.15 (6.47 - 5.72)	85.9 (84.1 - 87.6)	69.7 (67.3 - 72.0)	57.1 (54.4 - 59.6)	26.9 (24.3 - 29.6)	9.1 (7.1 - 11.2)	1.7 (0.6 - 3.9)
Digestive diseases	Crohn's disease	Male	85-95	365	2.55 (3.16 - 2.04)	70.5 (65.5 - 74.9)	46.0 (40.6 - 51.1)	30.0 (25.1 - 35.0)	6.5 (3.9 - 10.1)	0.0 (0.0 - 0.0)	0.0 (0.0 - 0.0)
Digestive diseases	Diverticular disease	Male	All	163849	16.61 (16.77 - 16.45)	94.4 (94.3 - 94.5)	87.4 (87.2 - 87.5)	81.0 (80.8 - 81.2)	66.6 (66.3 - 66.9)	53.8 (53.4 - 54.1)	43.1 (42.6 - 43.6)
Digestive diseases	Diverticular disease	Male	16-24	400	NR	100.0 (100.0 - 100.0)	99.7 (98.0 - 100.0)	98.8 (96.9 - 99.6)	97.7 (95.2 - 98.9)	96.6 (93.3 - 98.2)	96.6 (93.3 - 98.2)
Digestive diseases	Diverticular disease	Male	25-34	3237	NR	99.9 (99.7 - 100.0)	99.7 (99.4 - 99.8)	99.2 (98.8 - 99.5)	98.5 (98.0 - 99.0)	97.6 (96.8 - 98.3)	96.6 (95.3 - 97.6)
Digestive diseases	Diverticular disease	Male	35-44	11154	NR	99.8 (99.7 - 99.9)	99.4 (99.2 - 99.5)	99.0 (98.8 - 99.2)	97.6 (97.3 - 97.9)	95.8 (95.3 - 96.3)	93.4 (92.4 - 94.2)
Digestive diseases	Diverticular disease	Male	45-54	20691	NR	99.2 (99.0 - 99.3)	98.1 (97.9 - 98.3)	97.2 (97.0 - 97.5)	94.5 (94.1 - 94.8)	90.4 (89.8 - 90.9)	84.0 (83.0 - 85.0)
Digestive diseases	Diverticular disease	Male	55-64	35047	NR	98.0 (97.8 - 98.1)	95.6 (95.3 - 95.8)	93.2 (92.9 - 93.5)	86.1 (85.6 - 86.5)	76.3 (75.6 - 76.9)	62.6 (61.5 - 63.7)
Digestive diseases	Diverticular disease	Male	65-74	45658	14.28 (14.45 - 14.11)	95.3 (95.1 - 95.5)	89.3 (89.0 - 89.6)	83.5 (83.2 - 83.9)	67.4 (66.8 - 67.9)	46.7 (45.9 - 47.5)	25.7 (24.6 - 26.8)
Digestive diseases	Diverticular disease	Male	75-84	37569	6.94 (7.03 - 6.86)	89.6 (89.3 - 90.0)	75.9 (75.5 - 76.4)	62.7 (62.1 - 63.2)	32.0 (31.4 - 32.6)	10.6 (10.1 - 11.2)	2.1 (1.7 - 2.6)
Digestive diseases	Diverticular disease	Male	85-95	9939	3.12 (3.21 - 3.04)	77.6 (76.7 - 78.4)	51.6 (50.5 - 52.6)	31.8 (30.8 - 32.8)	6.2 (5.6 - 6.9)	0.4 (0.2 - 0.8)	0.1 (0.0 - 0.5)
Digestive diseases	Gallbladder & biliary	Male	All	187541	20.11 (20.33 - 19.88)	91.8 (91.7 - 91.9)	85.2 (85.0 - 85.4)	80.0 (79.8 - 80.2)	68.6 (68.4 - 68.9)	58.8 (58.5 - 59.0)	50.2 (49.8 - 50.6)
Digestive diseases	Gallbladder & biliary	Male	16-24	4676	NR	99.5 (99.3 - 99.7)	99.1 (98.8 - 99.4)	98.8 (98.4 - 99.1)	97.9 (97.4 - 98.3)	97.0 (96.3 - 97.5)	95.5 (94.3 - 96.4)
Digestive diseases	Gallbladder & biliary	Male	25-34	13169	NR	99.6 (99.5 - 99.7)	99.1 (98.9 - 99.2)	98.6 (98.4 - 98.8)	97.5 (97.2 - 97.8)	96.1 (95.6 - 96.5)	94.7 (94.0 - 95.3)
Digestive diseases	Gallbladder & biliary	Male	35-44	22270	NR	99.2 (99.1 - 99.3)	98.6 (98.4 - 98.7)	98.1 (97.9 - 98.3)	96.7 (96.4 - 97.0)	95.0 (94.6 - 95.3)	92.5 (91.9 - 93.0)
Digestive diseases	Gallbladder & biliary	Male	45-54	28842	NR	98.1 (97.9 - 98.2)	96.5 (96.3 - 96.8)	95.4 (95.1 - 95.6)	92.4 (92.0 - 92.7)	88.5 (88.0 - 89.0)	82.4 (81.6 - 83.2)
Digestive diseases	Gallbladder & biliary	Male	55-64	34089	NR	95.3 (95.1 - 95.5)	91.9 (91.6 - 92.2)	89.3 (88.9 - 89.6)	82.2 (81.7 - 82.6)	73.2 (72.6 - 73.8)	61.6 (60.7 - 62.5)
Digestive diseases	Gallbladder & biliary	Male	65-74	38351	13.43 (13.63 - 13.25)	90.8 (90.5 - 91.0)	83.8 (83.4 - 84.2)	78.0 (77.6 - 78.5)	62.4 (61.9 - 63.0)	43.8 (43.1 - 44.5)	24.9 (24.0 - 25.8)
Digestive diseases	Gallbladder & biliary	Male	75-84	33139	6.16 (6.25 - 6.07)	83.7 (83.2 - 84.1)	69.3 (68.8 - 69.8)	56.9 (56.3 - 57.5)	28.8 (28.2 - 29.4)	9.3 (8.8 - 9.8)	1.6 (1.3 - 1.9)
Digestive diseases	Gallbladder & biliary	Male	85-95	12624	2.57 (2.65 - 2.49)	69.7 (68.9 - 70.5)	45.2 (44.3 - 46.1)	27.0 (26.1 - 27.8)	5.4 (4.9 - 5.9)	0.5 (0.4 - 0.8)	0.1 (0.0 - 0.3)
Digestive diseases	Inflammatory bowel	Male	All	103003	NR	94.0 (93.8 - 94.1)	88.4 (88.2 - 88.6)	84.2 (84.0 - 84.5)	75.9 (75.6 - 76.2)	69.0 (68.7 - 69.3)	62.9 (62.4 - 63.3)
Digestive diseases	Inflammatory bowel	Male	16-24	12858	NR	99.8 (99.7 - 99.9)	99.6 (99.5 - 99.7)	99.4 (99.2 - 99.5)	98.8 (98.6 - 99.0)	98.0 (97.7 - 98.3)	97.1 (96.6 - 97.5)
Digestive diseases	Inflammatory bowel	Male	25-34	16178	NR	99.7 (99.6 - 99.8)	99.4 (99.2 - 99.5)	99.1 (98.9 - 99.2)	98.2 (97.9 - 98.4)	97.2 (96.9 - 97.5)	96.0 (95.6 - 96.5)
Digestive diseases	Inflammatory bowel	Male	35-44	13743	NR	99.4 (99.3 - 99.5)	98.6 (98.3 - 98.7)	98.0 (97.7 - 98.2)	96.3 (96.0 - 96.7)	94.0 (93.5 - 94.4)	91.3 (90.6 - 92.0)
Digestive diseases	Inflammatory bowel	Male	45-54	13456	NR	98.3 (98.0 - 98.5)	96.5 (96.2 - 96.9)	95.3 (94.9 - 95.6)	91.4 (90.8 - 91.9)	87.0 (86.3 - 87.6)	80.3 (79.3 - 81.2)
Digestive diseases	Inflammatory bowel	Male	55-64	15324	22.39	95.7 (95.4 - 96.0)	91.3 (90.8 - 91.7)	87.6 (87.1 - 88.2)	79.1 (78.4 - 79.8)	68.7 (67.8 - 69.6)	56.4 (55.2 - 57.5)
Digestive diseases	Inflammatory bowel	Male	65-74	15260	11.75 (11.94 - 11.49)	90.7 (90.2 - 91.1)	81.9 (81.3 - 82.5)	74.8 (74.0 - 75.5)	56.5 (55.6 - 57.5)	37.4 (36.3 - 38.4)	19.7 (18.5 - 20.9)
Digestive diseases	Inflammatory bowel	Male	75-84	12021	4.8 (4.93 - 4.67)	81.0 (80.2 - 81.7)	63.2 (62.3 - 64.1)	48.6 (47.6 - 49.5)	21.4 (20.5 - 22.3)	6.7 (6.0 - 7.4)	1.1 (0.7 - 1.6)
Digestive diseases	Inflammatory bowel	Male	85-95	4040	1.9 (2.05 - 1.78)	65.3 (63.8 - 66.8)	36.6 (35.1 - 38.2)	20.0 (18.7 - 21.4)	3.5 (2.9 - 4.2)	0.1 (0.0 - 0.4)	0.0 (0.0 - 0.0)
Digestive diseases	Pancreatitis	Male	All	56642	14.36 (14.58 - 14.12)	89.3 (89.1 - 89.6)	81.3 (81.0 - 81.7)	74.9 (74.6 - 75.3)	60.8 (60.3 - 61.2)	48.4 (47.8 - 49.0)	38.4 (37.7 - 39.2)
Digestive diseases	Pancreatitis	Male	16-24	1802	NR	99.1 (98.5 - 99.4)	98.8 (98.1 - 99.2)	98.6 (97.9 - 99.0)	97.5 (96.5 - 98.2)	96.6 (95.4 - 97.6)	95.6 (93.6 - 97.0)
Digestive diseases	Pancreatitis	Male	25-34	3133	NR	98.6 (98.2 - 99.0)	97.3 (96.6 - 97.8)	96.3 (95.5 - 96.9)	94.0 (93.0 - 94.9)	91.1 (89.7 - 92.4)	88.5 (86.4 - 90.4)
Digestive diseases	Pancreatitis	Male	35-44	5235	NR	97.4 (96.9 - 97.8)	95.1 (94.5 - 95.7)	93.4 (92.7 - 94.1)	88.9 (87.9 - 89.8)	82.6 (81.2 - 83.9)	75.7 (73.6 - 77.6)
Digestive diseases	Pancreatitis	Male	45-54	8072	NR	95.4 (94.9 - 95.8)	91.9 (91.3 - 92.5)	89.3 (88.5 - 89.9)	81.0 (80.0 - 82.0)	71.9 (70.6 - 73.2)	62.6 (60.8 - 64.4)
Digestive diseases	Pancreatitis	Male	55-64	11036	17.66 (18.13 - 17.06)	92.6 (92.0 - 93.0)	86.3 (85.6 - 86.9)	82.0 (81.3 - 82.8)	69.8 (68.7 - 70.8)	57.3 (56.0 - 58.5)	42.8 (41.1 - 44.5)
Digestive diseases	Pancreatitis	Male	65-74	12915	11.63 (11.92 - 11.26)	88.2 (87.6 - 88.8)	79.6 (78.9 - 80.3)	73.0 (72.2 - 73.8)	56.2 (55.1 - 57.2)	35.0 (33.7 - 36.4)	16.7 (15.1 - 18.3)
Digestive diseases	Pancreatitis	Male	75-84	10593	5.82 (6.05 - 5.62)	82.2 (81.5 - 83.0)	68.4 (67.4 - 69.3)	55.5 (54.4 - 56.5)	26.9 (25.7 - 28.0)	8.0 (7.2 - 9.0)	1.2 (0.7 - 1.8)
Digestive diseases	Pancreatitis	Male	85-95	3744	2.53 (2.69 - 2.36)	67.8 (66.3 - 69.3)	45.4 (43.7 - 47.1)	26.9 (25.4 - 28.5)	4.6 (3.8 - 5.6)	0.7 (0.3 - 1.3)	0.0 (0.0 - 0.0)
Digestive diseases	Ulcerative colitis	Male	All	90137	NR	93.5 (93.4 - 93.7)	87.7 (87.5 - 87.9)	83.3 (83.0 - 83.5)	74.7 (74.4 - 75.0)	67.9 (67.5 - 68.2)	61.8 (61.4 - 62.2)
Digestive diseases	Ulcerative colitis	Male	16-24	10666	NR	99.8 (99.7 - 99.9)	99.6 (99.4 - 99.7)	99.4 (99.2 - 99.5)	98.8 (98.5 - 99.0)	97.9 (97.6 - 98.2)	97.0 (96.5 - 97.5)
Digestive diseases	Ulcerative colitis	Male	25-34	13891	NR	99.6 (99.5 - 99.7)	99.3 (99.1 - 99.4)	99.0 (98.9 - 99.2)	98.1 (97.9 - 98.4)	97.1 (96.8 - 97.5)	96.1 (95.6 - 96.5)
Digestive diseases	Ulcerative colitis	Male	35-44	11808	NR	99.4 (99.2 - 99.5)	98.5 (98.2 - 98.7)	97.9 (97.6 - 98.1)	96.2 (95.8 - 96.5)	93.7 (93.2 - 94.2)	91.0 (90.2 - 91.7)
Digestive diseases	Ulcerative colitis	Male	45-54	11623	NR	98.1 (97.9 - 98.4)	96.2 (95.9 - 96.6)	94.9 (94.5 - 95.3)	91.0 (90.4 - 91.6)	86.7 (85.9 - 87.4)	80.0 (78.9 - 81.0)
Digestive diseases	Ulcerative colitis	Male	55-64	13382	22.46	95.5 (95.1 - 95.8)	91.0 (90.4 - 91.4)	87.2 (86.6 - 87.8)	78.5 (77.7 - 79.2)	68.2 (67.2 - 69.1)	56.2 (54.9 - 57.4)
Digestive diseases	Ulcerative colitis	Male	65-74	13740	11.62 (11.83 - 11.31)	90.4 (89.9 - 90.9)	81.3 (80.7 - 82.0)	74.1 (73.3 - 74.9)	55.8 (54.9 - 56.8)	36.9 (35.8 - 38.1)	19.4 (18.1 - 20.7)
Digestive diseases	Ulcerative colitis	Male	75-84	11117	4.72 (4.85 - 4.59)	80.6 (79.8 - 81.3)	62.8 (61.8 - 63.7)	47.9 (46.9 - 48.9)	20.8 (19.9 - 21.7)	6.6 (5.9 - 7.3)	1.0 (0.6 - 1.5)
Digestive diseases	Ulcerative colitis	Male	85-95	3796	1.88 (2.0 - 1.76)	65.0 (63.4 - 66.5)	36.0 (34.4 - 37.6)	19.3 (17.9 - 20.6)	3.3 (2.7 - 4.1)	0.1 (0.0 - 0.4)	0.0 (0.0 - 0.0)
Digestive diseases	Upper digest diseases	Male	All	417427	21.48 (21.62 - 21.28)	92.6 (92.5 - 92.7)	86.2 (86.0 - 86.3)	80.9 (80.8 - 81.0)	69.9 (69.7 - 70.0)	60.6 (60.4 - 60.7)	52.2 (51.9 - 52.4)

Appendix Table A6: Unadjusted Median Survival and Survival Probabilities by Age, males (Page 12 of 18)

Group	Diagnosis Category	Sex	Age	N	Median Survival (y)	1y (%)	3y (%)	5y (%)	10y (%)	15y (%)	20y (%)
Digestive diseases	Upper digest diseases	Male	16-24	24051	NR	99.8 (99.7 - 99.8)	99.5 (99.4 - 99.6)	99.2 (99.1 - 99.3)	98.6 (98.4 - 98.7)	97.7 (97.4 - 97.9)	96.9 (96.5 - 97.2)
Digestive diseases	Upper digest diseases	Male	25-34	39153	NR	99.7 (99.7 - 99.8)	99.3 (99.2 - 99.4)	99.0 (98.9 - 99.1)	98.1 (97.9 - 98.2)	97.2 (97.0 - 97.4)	95.9 (95.5 - 96.2)
Digestive diseases	Upper digest diseases	Male	35-44	48384	NR	99.3 (99.2 - 99.4)	98.6 (98.5 - 98.7)	97.9 (97.8 - 98.1)	96.3 (96.1 - 96.5)	94.1 (93.8 - 94.4)	91.1 (90.7 - 91.5)
Digestive diseases	Upper digest diseases	Male	45-54	59969	NR	98.1 (98.0 - 98.2)	96.0 (95.9 - 96.2)	94.3 (94.1 - 94.5)	90.0 (89.7 - 90.3)	84.9 (84.5 - 85.3)	78.2 (77.7 - 78.7)
Digestive diseases	Upper digest diseases	Male	55-64	76917	22.03 (22.22 - 21.82)	95.6 (95.4 - 95.7)	91.2 (91.0 - 91.4)	87.5 (87.3 - 87.7)	78.7 (78.4 - 79.0)	68.6 (68.1 - 69.0)	56.0 (55.4 - 56.5)
Digestive diseases	Upper digest diseases	Male	65-74	82717	12.52 (12.64 - 12.42)	91.2 (91.0 - 91.4)	83.2 (82.9 - 83.5)	76.3 (76.0 - 76.6)	59.2 (58.9 - 59.6)	40.4 (40.0 - 40.9)	21.2 (20.6 - 21.8)
Digestive diseases	Upper digest diseases	Male	75-84	64909	5.59 (5.65 - 5.52)	82.4 (82.1 - 82.7)	67.0 (66.7 - 67.4)	53.7 (53.3 - 54.1)	25.9 (25.5 - 26.3)	8.8 (8.5 - 9.2)	1.6 (1.4 - 1.8)
Digestive diseases	Upper digest diseases	Male	85-95	20888	2.23 (2.29 - 2.17)	66.3 (65.6 - 66.9)	41.4 (40.7 - 42.1)	24.1 (23.4 - 24.7)	4.2 (3.8 - 4.5)	0.2 (0.1 - 0.3)	0.1 (0.0 - 0.2)
Digestive diseases	Vascular intestinal	Male	All	14450	5.02 (5.22 - 4.82)	71.7 (71.0 - 72.4)	60.1 (59.2 - 60.9)	50.1 (49.2 - 51.0)	32.9 (31.9 - 33.9)	22.6 (21.5 - 23.6)	17.1 (15.9 - 18.4)
Digestive diseases	Vascular intestinal	Male	16-24	139	NR	97.8 (93.4 - 99.3)	96.3 (91.3 - 98.4)	95.4 (90.0 - 97.9)	91.6 (84.0 - 95.7)	88.0 (78.5 - 93.5)	85.7 (74.9 - 92.1)
Digestive diseases	Vascular intestinal	Male	25-34	224	NR	95.5 (91.7 - 97.5)	94.0 (89.8 - 96.5)	92.8 (88.3 - 95.6)	89.5 (83.8 - 93.3)	89.5 (83.8 - 93.3)	87.9 (80.9 - 92.4)
Digestive diseases	Vascular intestinal	Male	35-44	379	NR	92.8 (89.7 - 95.0)	90.7 (87.2 - 93.3)	89.3 (85.6 - 92.1)	86.3 (82.0 - 89.7)	81.9 (76.5 - 86.2)	78.5 (71.7 - 83.9)
Digestive diseases	Vascular intestinal	Male	45-54	767	NR	85.5 (82.7 - 87.8)	81.3 (78.3 - 83.9)	77.8 (74.5 - 80.7)	72.1 (68.2 - 75.6)	65.1 (60.2 - 69.6)	58.6 (51.9 - 64.6)
Digestive diseases	Vascular intestinal	Male	55-64	1973	12.24 (13.3 - 10.93)	81.0 (79.2 - 82.7)	73.6 (71.6 - 75.6)	68.3 (66.1 - 70.4)	55.2 (52.6 - 57.7)	42.8 (39.7 - 45.8)	30.8 (26.9 - 34.8)
Digestive diseases	Vascular intestinal	Male	65-74	4205	6.51 (7.0 - 6.05)	76.1 (74.8 - 77.4)	65.8 (64.2 - 67.2)	56.5 (54.8 - 58.1)	37.1 (35.2 - 39.0)	20.0 (17.9 - 22.3)	9.1 (6.5 - 12.1)
Digestive diseases	Vascular intestinal	Male	75-84	4936	3.33 (3.51 - 3.15)	66.9 (65.6 - 68.3)	52.6 (51.1 - 54.1)	38.5 (36.9 - 40.0)	13.9 (12.5 - 15.3)	3.3 (2.4 - 4.4)	0.7 (0.2 - 2.0)
Digestive diseases	Vascular intestinal	Male	85-95	1790	0.97 (1.17 - 0.82)	49.5 (47.2 - 51.8)	29.6 (27.3 - 31.8)	15.3 (13.4 - 17.2)	2.5 (1.7 - 3.7)	0.2 (0.0 - 1.0)	0.2 (0.0 - 1.0)
Mental disorders & substance use	ADHD	Male	All	96400	NR	99.5 (99.5 - 99.5)	98.5 (98.4 - 98.6)	97.4 (97.3 - 97.5)	94.5 (94.3 - 94.7)	91.1 (90.7 - 91.4)	86.9 (86.0 - 87.7)
Mental disorders & substance use	ADHD	Male	16-24	37572	NR	99.8 (99.7 - 99.8)	99.1 (99.0 - 99.2)	98.5 (98.4 - 98.7)	97.0 (96.8 - 97.2)	95.3 (94.9 - 95.6)	93.6 (92.9 - 94.3)
Mental disorders & substance use	ADHD	Male	25-34	27917	NR	99.5 (99.4 - 99.5)	98.5 (98.3 - 98.6)	97.5 (97.3 - 97.7)	95.0 (94.6 - 95.3)	92.1 (91.5 - 92.7)	85.6 (82.7 - 88.0)
Mental disorders & substance use	ADHD	Male	35-44	17812	NR	99.4 (99.2 - 99.5)	98.2 (98.0 - 98.4)	97.0 (96.7 - 97.2)	93.4 (92.9 - 93.9)	88.7 (87.8 - 89.5)	81.3 (78.4 - 83.9)
Mental disorders & substance use	ADHD	Male	45-54	9722	NR	99.2 (99.0 - 99.4)	97.7 (97.3 - 98.0)	95.6 (95.1 - 96.0)	89.7 (88.8 - 90.4)	81.9 (80.4 - 83.3)	72.6 (67.5 - 77.0)
Mental disorders & substance use	ADHD	Male	55-64	2920	20.61	98.9 (98.4 - 99.2)	95.9 (95.1 - 96.7)	93.1 (91.9 - 94.0)	82.3 (80.3 - 84.2)	70.2 (66.4 - 73.7)	63.1 (56.1 - 69.3)
Mental disorders & substance use	ADHD	Male	65-74	383	15.05	95.9 (93.3 - 97.5)	90.9 (87.3 - 93.5)	84.1 (79.4 - 87.8)	64.7 (57.5 - 71.0)	52.0 (41.5 - 61.5)	46.8 (33.2 - 59.3)
Mental disorders & substance use	ADHD	Male	75-84	64	8.47 (9.35 - 6.0)	91.9 (81.7 - 96.6)	86.6 (74.9 - 93.1)	74.6 (59.8 - 84.6)	30.5 (13.5 - 49.4)	11.4 (1.0 - 35.8)	11.4 (1.0 - 35.8)
Mental disorders & substance use	ADHD	Male	85-95	10	2.38 (7.89 - 0.2)	88.9 (43.3 - 98.4)	44.4 (13.6 - 71.9)	22.2 (3.4 - 51.3)	11.1 (0.6 - 38.8)	0.0 (0.0 - 0.0)	0.0 (0.0 - 0.0)
Mental disorders & substance use	Alcohol use disorders	Male	All	237330	NR	96.0 (95.9 - 96.0)	91.2 (91.1 - 91.3)	86.7 (86.5 - 86.8)	76.4 (76.2 - 76.6)	66.8 (66.5 - 67.0)	57.3 (56.9 - 57.6)
Mental disorders & substance use	Alcohol use disorders	Male	16-24	47738	NR	99.6 (99.6 - 99.7)	98.9 (98.8 - 99.0)	98.2 (98.1 - 98.3)	96.6 (96.4 - 96.8)	95.1 (94.9 - 95.4)	93.9 (93.5 - 94.2)
Mental disorders & substance use	Alcohol use disorders	Male	25-34	32425	NR	99.0 (98.9 - 99.1)	97.7 (97.6 - 97.9)	96.6 (96.4 - 96.8)	93.8 (93.5 - 94.1)	90.3 (89.8 - 90.7)	86.8 (86.1 - 87.5)
Mental disorders & substance use	Alcohol use disorders	Male	35-44	32604	NR	98.7 (98.6 - 98.8)	96.8 (96.6 - 97.0)	94.8 (94.6 - 95.1)	89.9 (89.5 - 90.3)	83.6 (83.1 - 84.1)	74.8 (74.0 - 75.6)
Mental disorders & substance use	Alcohol use disorders	Male	45-54	40335	21.57 (21.89 - 21.26)	97.4 (97.3 - 97.6)	93.8 (93.5 - 94.0)	90.3 (90.0 - 90.6)	80.6 (80.1 - 81.0)	69.0 (68.4 - 69.5)	54.9 (54.0 - 55.8)
Mental disorders & substance use	Alcohol use disorders	Male	55-64	40553	14.01 (14.21 - 13.82)	94.6 (94.4 - 94.8)	87.6 (87.2 - 87.9)	80.7 (80.3 - 81.1)	63.5 (63.0 - 64.1)	46.8 (46.1 - 47.4)	30.7 (29.8 - 31.5)
Mental disorders & substance use	Alcohol use disorders	Male	65-74	30129	8.74 (8.88 - 8.61)	89.8 (89.4 - 90.1)	79.0 (78.6 - 79.5)	68.5 (68.0 - 69.1)	44.0 (43.3 - 44.7)	22.8 (22.1 - 23.6)	8.7 (7.9 - 9.6)
Mental disorders & substance use	Alcohol use disorders	Male	75-84	11885	4.8 (4.94 - 4.68)	83.4 (82.7 - 84.1)	65.3 (64.4 - 66.2)	48.6 (47.6 - 49.6)	18.3 (17.4 - 19.3)	3.2 (2.7 - 3.9)	0.4 (0.1 - 1.0)
Mental disorders & substance use	Alcohol use disorders	Male	85-95	1642	2.71 (2.9 - 2.5)	73.9 (71.6 - 76.0)	46.2 (43.6 - 48.7)	27.4 (25.0 - 29.8)	2.7 (1.7 - 4.0)	0.0 (0.0 - 0.0)	0.0 (0.0 - 0.0)
Mental disorders & substance use	Anorexia nervosa	Male	All	1080	NR	97.5 (96.3 - 98.2)	94.8 (93.2 - 96.0)	93.3 (91.5 - 94.7)	90.1 (87.8 - 91.9)	87.4 (84.6 - 89.8)	85.5 (81.5 - 88.6)
Mental disorders & substance use	Anorexia nervosa	Male	16-24	679	NR	99.9 (98.9 - 100.0)	99.7 (98.7 - 99.9)	99.7 (98.7 - 99.9)	98.4 (96.7 - 99.3)	97.5 (95.0 - 98.7)	97.5 (95.0 - 98.7)
Mental disorders & substance use	Anorexia nervosa	Male	25-34	188	NR	99.4 (96.1 - 99.9)	97.6 (93.7 - 99.1)	96.9 (92.8 - 98.7)	96.9 (92.8 - 98.7)	93.1 (84.1 - 97.1)	93.1 (84.1 - 97.1)
Mental disorders & substance use	Anorexia nervosa	Male	35-44	66	NR	98.5 (89.7 - 99.8)	95.1 (85.7 - 98.4)	93.3 (83.0 - 97.4)	81.9 (65.9 - 90.9)	81.9 (65.9 - 90.9)	81.9 (65.9 - 90.9)
Mental disorders & substance use	Anorexia nervosa	Male	45-54	62	NR	95.2 (85.7 - 98.4)	91.9 (81.6 - 96.5)	90.1 (79.4 - 95.5)	81.7 (68.3 - 89.8)	68.5 (52.6 - 80.1)	68.5 (52.6 - 80.1)
Mental disorders & substance use	Anorexia nervosa	Male	55-64	32	7.0 (16.38 - 3.07)	90.4 (73.1 - 96.8)	75.1 (54.4 - 87.4)	62.0 (40.3 - 77.8)	42.4 (22.0 - 61.4)	42.4 (22.0 - 61.4)	14.1 (1.0 - 43.5)
Mental disorders & substance use	Anorexia nervosa	Male	65-74	28	2.44 (8.72 - 1.44)	71.4 (50.9 - 84.6)	46.1 (26.0 - 64.0)	31.1 (13.7 - 50.4)	15.6 (3.1 - 36.9)	0.0 (0.0 - 0.0)	0.0 (0.0 - 0.0)
Mental disorders & substance use	Anorexia nervosa	Male	75-84	17	2.03 (3.47 - 0.62)	75.6 (47.3 - 90.1)	31.5 (11.5 - 54.0)	15.8 (2.8 - 38.5)	7.9 (0.5 - 29.3)	7.9 (0.5 - 29.3)	0.0 (0.0 - 0.0)
Mental disorders & substance use	Anorexia nervosa	Male	85-95	7	0.44 (0.83 - 0.05)	14.3 (0.7 - 46.5)	14.3 (0.7 - 46.5)	0.0 (0.0 - 0.0)	0.0 (0.0 - 0.0)	0.0 (0.0 - 0.0)	0.0 (0.0 - 0.0)
Mental disorders & substance use	Anxiety disorders	Male	All	353038	NR	96.9 (96.8 - 96.9)	94.0 (94.0 - 94.1)	91.7 (91.6 - 91.8)	86.3 (86.2 - 86.4)	80.9 (80.8 - 81.1)	75.1 (74.9 - 75.4)
Mental disorders & substance use	Anxiety disorders	Male	16-24	79101	NR	99.6 (99.5 - 99.6)	98.9 (98.9 - 99.0)	98.2 (98.1 - 98.3)	96.6 (96.4 - 96.7)	94.9 (94.7 - 95.1)	93.4 (93.0 - 93.7)
Mental disorders & substance use	Anxiety disorders	Male	25-34	79308	NR	99.4 (99.4 - 99.5)	98.7 (98.6 - 98.7)	98.0 (97.8 - 98.1)	96.1 (96.0 - 96.3)	94.1 (93.8 - 94.3)	91.7 (91.3 - 92.1)
Mental disorders & substance use	Anxiety disorders	Male	35-44	64479	NR	99.1 (99.1 - 99.2)	98.1 (98.0 - 98.2)	97.2 (97.1 - 97.4)	94.7 (94.5 - 94.9)	91.5 (91.2 - 91.8)	87.1 (86.6 - 87.5)
Mental disorders & substance use	Anxiety disorders	Male	45-54	54709	NR	98.3 (98.2 - 98.4)	96.4 (96.3 - 96.6)	94.7 (94.5 - 94.9)	89.7 (89.4 - 90.0)	83.3 (82.8 - 83.7)	75.2 (74.5 - 75.8)
Mental disorders & substance use	Anxiety disorders	Male	55-64	36918	20.75 (21.04 - 20.34)	95.9 (95.7 - 96.1)	91.8 (91.5 - 92.1)	88.1 (87.8 - 88.5)	78.1 (77.6 - 78.6)	66.3 (65.6 - 67.0)	52.1 (51.0 - 53.1)

Appendix Table A6: Unadjusted Median Survival and Survival Probabilities by Age, males (Page 13 of 18)

Group	Diagnosis Category	Sex	Age	N	Median Survival (y)	1y (%)	3y (%)	5y (%)	10y (%)	15y (%)	20y (%)
Mental disorders & substance use	Anxiety disorders	Male	65-74	19476	10.26 (10.47 - 10.03)	88.1 (87.6 - 88.5)	78.7 (78.1 - 79.3)	70.4 (69.7 - 71.0)	51.0 (50.2 - 51.8)	31.7 (30.7 - 32.7)	14.7 (13.4 - 16.0)
Mental disorders & substance use	Anxiety disorders	Male	75-84	13570	4.47 (4.61 - 4.34)	78.0 (77.2 - 78.7)	60.4 (59.5 - 61.2)	46.3 (45.4 - 47.2)	19.1 (18.3 - 20.0)	5.3 (4.7 - 6.0)	1.1 (0.7 - 1.7)
Mental disorders & substance use	Anxiety disorders	Male	85-95	5326	1.93 (2.03 - 1.82)	63.4 (62.1 - 64.7)	36.2 (34.9 - 37.6)	20.6 (19.4 - 21.8)	3.2 (2.6 - 3.9)	0.1 (0.0 - 0.4)	0.1 (0.0 - 0.4)
Mental disorders & substance use	Bipolar disorder	Male	All	39801	NR	97.7 (97.5 - 97.8)	94.6 (94.4 - 94.8)	91.7 (91.4 - 91.9)	84.2 (83.8 - 84.6)	75.8 (75.2 - 76.3)	65.3 (64.4 - 66.3)
Mental disorders & substance use	Bipolar disorder	Male	16-24	6001	NR	99.3 (99.1 - 99.5)	98.4 (98.0 - 98.7)	97.1 (96.7 - 97.6)	94.8 (94.1 - 95.4)	93.1 (92.2 - 93.9)	90.3 (88.2 - 92.1)
Mental disorders & substance use	Bipolar disorder	Male	25-34	9089	NR	99.2 (99.0 - 99.4)	97.8 (97.5 - 98.1)	96.6 (96.2 - 97.0)	94.2 (93.6 - 94.8)	91.6 (90.8 - 92.4)	89.3 (88.0 - 90.4)
Mental disorders & substance use	Bipolar disorder	Male	35-44	7933	NR	99.0 (98.8 - 99.2)	97.6 (97.3 - 98.0)	96.4 (96.0 - 96.8)	93.1 (92.5 - 93.7)	88.6 (87.6 - 89.5)	83.0 (81.3 - 84.6)
Mental disorders & substance use	Bipolar disorder	Male	45-54	6981	NR	98.5 (98.2 - 98.7)	96.5 (96.1 - 96.9)	94.3 (93.7 - 94.8)	88.1 (87.2 - 88.9)	80.7 (79.4 - 81.8)	70.8 (68.8 - 72.7)
Mental disorders & substance use	Bipolar disorder	Male	55-64	5494	18.54 (19.16 - 18.16)	97.0 (96.5 - 97.4)	92.5 (91.8 - 93.2)	88.3 (87.4 - 89.1)	76.8 (75.5 - 77.9)	62.6 (61.0 - 64.1)	45.6 (43.3 - 47.8)
Mental disorders & substance use	Bipolar disorder	Male	65-74	2744	10.9 (11.37 - 10.45)	93.2 (92.1 - 94.0)	84.8 (83.4 - 86.1)	77.4 (75.7 - 79.0)	54.7 (52.6 - 56.7)	31.5 (29.2 - 33.8)	13.4 (11.2 - 15.9)
Mental disorders & substance use	Bipolar disorder	Male	75-84	1280	5.08 (5.59 - 4.59)	84.7 (82.6 - 86.5)	66.9 (64.1 - 69.4)	50.4 (47.5 - 53.2)	19.8 (17.4 - 22.3)	6.8 (5.1 - 8.7)	0.0 (0.0 - 0.0)
Mental disorders & substance use	Bipolar disorder	Male	85-95	279	2.17 (2.63 - 1.86)	70.6 (64.9 - 75.6)	40.6 (34.7 - 46.5)	21.9 (17.1 - 27.2)	3.1 (1.3 - 6.0)	0.0 (0.0 - 0.0)	0.0 (0.0 - 0.0)
Mental disorders & substance use	Depressive disorders	Male	All	278186	NR	95.3 (95.2 - 95.4)	90.7 (90.6 - 90.8)	86.8 (86.7 - 87.0)	79.0 (78.9 - 79.2)	72.2 (72.0 - 72.4)	65.3 (65.0 - 65.6)
Mental disorders & substance use	Depressive disorders	Male	16-24	56423	NR	99.5 (99.4 - 99.6)	98.8 (98.8 - 98.9)	98.1 (98.0 - 98.3)	96.5 (96.3 - 96.6)	94.8 (94.6 - 95.1)	93.2 (92.7 - 93.6)
Mental disorders & substance use	Depressive disorders	Male	25-34	52898	NR	99.3 (99.2 - 99.4)	98.4 (98.2 - 98.5)	97.6 (97.5 - 97.8)	95.8 (95.6 - 96.0)	93.8 (93.5 - 94.1)	91.3 (90.9 - 91.8)
Mental disorders & substance use	Depressive disorders	Male	35-44	44028	NR	99.0 (98.9 - 99.1)	97.9 (97.7 - 98.0)	96.8 (96.6 - 97.0)	94.0 (93.8 - 94.3)	90.5 (90.1 - 90.8)	85.6 (85.1 - 86.2)
Mental disorders & substance use	Depressive disorders	Male	45-54	41502	NR	98.2 (98.1 - 98.4)	96.1 (96.0 - 96.3)	94.1 (93.9 - 94.3)	88.6 (88.3 - 89.0)	81.9 (81.5 - 82.4)	74.0 (73.3 - 74.7)
Mental disorders & substance use	Depressive disorders	Male	55-64	32447	19.76 (19.98 - 19.43)	95.9 (95.6 - 96.1)	91.3 (91.0 - 91.6)	87.0 (86.7 - 87.4)	76.0 (75.4 - 76.5)	63.4 (62.7 - 64.1)	49.0 (48.0 - 49.9)
Mental disorders & substance use	Depressive disorders	Male	65-74	22010	9.04 (9.2 - 8.85)	89.0 (88.5 - 89.4)	78.1 (77.5 - 78.6)	68.1 (67.4 - 68.7)	46.0 (45.2 - 46.7)	25.8 (25.0 - 26.6)	10.6 (9.8 - 11.5)
Mental disorders & substance use	Depressive disorders	Male	75-84	20411	3.97 (4.04 - 3.88)	78.6 (78.1 - 79.2)	58.4 (57.7 - 59.1)	42.0 (41.2 - 42.7)	14.6 (14.0 - 15.2)	3.5 (3.1 - 3.9)	0.5 (0.3 - 0.7)
Mental disorders & substance use	Depressive disorders	Male	85-95	8318	1.71 (1.79 - 1.65)	62.6 (61.6 - 63.7)	33.1 (32.1 - 34.2)	15.8 (15.0 - 16.6)	1.8 (1.5 - 2.2)	0.1 (0.0 - 0.3)	0.0 (0.0 - 0.0)
Mental disorders & substance use	Drug use disorders	Male	All	111167	NR	97.1 (97.0 - 97.2)	93.6 (93.5 - 93.8)	90.6 (90.4 - 90.8)	83.5 (83.2 - 83.8)	76.0 (75.7 - 76.4)	68.4 (67.9 - 68.9)
Mental disorders & substance use	Drug use disorders	Male	16-24	39728	NR	99.1 (99.0 - 99.2)	97.5 (97.4 - 97.7)	95.9 (95.7 - 96.1)	92.5 (92.2 - 92.8)	88.7 (88.3 - 89.2)	85.6 (84.9 - 86.3)
Mental disorders & substance use	Drug use disorders	Male	25-34	28626	NR	98.5 (98.4 - 98.7)	96.6 (96.4 - 96.8)	94.9 (94.7 - 95.2)	90.8 (90.4 - 91.2)	86.5 (85.9 - 87.1)	81.9 (81.0 - 82.7)
Mental disorders & substance use	Drug use disorders	Male	35-44	15776	NR	98.2 (98.0 - 98.4)	95.6 (95.2 - 95.9)	93.3 (92.9 - 93.7)	87.2 (86.6 - 87.8)	79.9 (79.0 - 80.7)	71.3 (70.1 - 72.5)
Mental disorders & substance use	Drug use disorders	Male	45-54	12097	20.5 (21.23 - 19.94)	96.4 (96.1 - 96.8)	91.8 (91.2 - 92.2)	87.8 (87.2 - 88.4)	77.0 (76.1 - 77.8)	64.7 (63.6 - 65.8)	51.4 (49.8 - 53.0)
Mental disorders & substance use	Drug use disorders	Male	55-64	7775	14.27 (14.7 - 13.86)	93.4 (92.8 - 94.0)	85.9 (85.1 - 86.7)	79.4 (78.5 - 80.4)	63.1 (61.9 - 64.3)	47.4 (45.9 - 49.0)	31.6 (29.5 - 33.7)
Mental disorders & substance use	Drug use disorders	Male	65-74	4293	8.82 (9.26 - 8.43)	88.7 (87.7 - 89.6)	76.5 (75.2 - 77.8)	66.3 (64.8 - 67.8)	45.9 (44.1 - 47.7)	26.9 (25.0 - 28.9)	11.7 (9.5 - 14.1)
Mental disorders & substance use	Drug use disorders	Male	75-84	2092	4.34 (4.65 - 4.0)	79.4 (77.6 - 81.1)	59.9 (57.7 - 62.1)	45.4 (43.0 - 47.7)	16.7 (14.7 - 18.9)	5.0 (3.5 - 6.8)	1.1 (0.4 - 2.6)
Mental disorders & substance use	Drug use disorders	Male	85-95	758	1.82 (2.04 - 1.58)	65.7 (62.2 - 69.0)	34.5 (30.9 - 38.0)	17.4 (14.6 - 20.5)	3.2 (1.8 - 5.3)	0.0 (0.0 - 0.0)	0.0 (0.0 - 0.0)
Mental disorders & substance use	Dysthymia	Male	All	13090	NR	98.5 (98.3 - 98.7)	96.6 (96.2 - 96.9)	94.5 (94.0 - 94.9)	88.9 (88.3 - 89.5)	82.5 (81.6 - 83.4)	75.4 (73.9 - 76.8)
Mental disorders & substance use	Dysthymia	Male	16-24	2485	NR	99.5 (99.1 - 99.7)	98.8 (98.2 - 99.1)	98.2 (97.6 - 98.7)	96.4 (95.4 - 97.1)	94.8 (93.4 - 95.9)	92.7 (90.6 - 94.4)
Mental disorders & substance use	Dysthymia	Male	25-34	3149	NR	99.5 (99.1 - 99.7)	98.4 (97.9 - 98.8)	97.8 (97.1 - 98.3)	96.1 (95.2 - 96.9)	94.2 (92.9 - 95.2)	91.4 (88.6 - 93.4)
Mental disorders & substance use	Dysthymia	Male	35-44	2403	NR	99.0 (98.5 - 99.4)	97.8 (97.1 - 98.3)	96.7 (95.9 - 97.4)	94.0 (92.8 - 94.9)	90.5 (88.9 - 91.9)	84.0 (80.9 - 86.6)
Mental disorders & substance use	Dysthymia	Male	45-54	2413	NR	99.3 (98.9 - 99.6)	97.4 (96.6 - 98.0)	95.7 (94.8 - 96.5)	90.4 (89.0 - 91.6)	83.2 (81.2 - 85.0)	75.9 (72.8 - 78.6)
Mental disorders & substance use	Dysthymia	Male	55-64	1732	21.18	98.2 (97.4 - 98.7)	95.0 (93.9 - 96.0)	91.5 (90.0 - 92.7)	82.2 (80.0 - 84.1)	70.7 (67.9 - 73.4)	57.0 (52.5 - 61.3)
Mental disorders & substance use	Dysthymia	Male	65-74	583	11.96 (12.83 - 10.79)	95.0 (92.8 - 96.5)	89.7 (86.8 - 91.9)	81.0 (77.4 - 84.1)	59.8 (55.1 - 64.1)	36.5 (31.3 - 41.8)	21.3 (15.5 - 27.8)
Mental disorders & substance use	Dysthymia	Male	75-84	259	6.05 (6.81 - 5.45)	84.3 (79.2 - 88.2)	73.5 (67.6 - 78.6)	59.2 (52.7 - 65.2)	26.0 (20.0 - 32.4)	5.0 (1.8 - 10.7)	3.3 (0.8 - 9.1)
Mental disorders & substance use	Dysthymia	Male	85-95	66	3.04 (3.57 - 1.36)	69.7 (57.1 - 79.3)	50.4 (37.6 - 61.9)	25.2 (15.2 - 36.6)	5.6 (1.5 - 13.8)	0.0 (0.0 - 0.0)	0.0 (0.0 - 0.0)
Mental disorders & substance use	Major depression	Male	All	274828	NR	95.2 (95.2 - 95.3)	90.6 (90.5 - 90.7)	86.7 (86.6 - 86.8)	78.9 (78.7 - 79.0)	72.0 (71.8 - 72.2)	65.1 (64.8 - 65.4)
Mental disorders & substance use	Major depression	Male	16-24	55617	NR	99.5 (99.4 - 99.6)	98.8 (98.7 - 98.9)	98.1 (98.0 - 98.3)	96.4 (96.3 - 96.6)	94.8 (94.5 - 95.1)	93.1 (92.7 - 93.6)
Mental disorders & substance use	Major depression	Male	25-34	52099	NR	99.3 (99.2 - 99.4)	98.3 (98.2 - 98.5)	97.6 (97.5 - 97.8)	95.8 (95.6 - 96.0)	93.8 (93.5 - 94.0)	91.3 (90.8 - 91.8)
Mental disorders & substance use	Major depression	Male	35-44	43374	NR	99.0 (98.9 - 99.1)	97.9 (97.7 - 98.0)	96.8 (96.6 - 97.0)	94.0 (93.7 - 94.2)	90.4 (90.1 - 90.8)	85.7 (85.1 - 86.3)
Mental disorders & substance use	Major depression	Male	45-54	40915	NR	98.2 (98.1 - 98.3)	96.1 (95.9 - 96.3)	94.0 (93.8 - 94.3)	88.6 (88.2 - 88.9)	81.9 (81.4 - 82.3)	73.9 (73.2 - 74.6)
Mental disorders & substance use	Major depression	Male	55-64	32121	19.69 (19.92 - 19.29)	95.8 (95.6 - 96.0)	91.3 (90.9 - 91.6)	86.9 (86.5 - 87.3)	75.8 (75.2 - 76.3)	63.2 (62.5 - 63.9)	48.7 (47.7 - 49.7)
Mental disorders & substance use	Major depression	Male	65-74	21920	9.02 (9.19 - 8.84)	88.9 (88.5 - 89.4)	78.0 (77.5 - 78.6)	68.0 (67.4 - 68.6)	45.9 (45.2 - 46.7)	25.7 (24.9 - 26.5)	10.5 (9.6 - 11.4)
Mental disorders & substance use	Major depression	Male	75-84	20343	3.95 (4.03 - 3.88)	78.6 (78.0 - 79.2)	58.4 (57.7 - 59.1)	41.9 (41.2 - 42.6)	14.6 (14.0 - 15.1)	3.5 (3.1 - 3.8)	0.5 (0.3 - 0.7)
Mental disorders & substance use	Major depression	Male	85-95	8290	1.71 (1.78 - 1.65)	62.7 (61.6 - 63.7)	33.0 (32.0 - 34.1)	15.8 (15.0 - 16.6)	1.8 (1.5 - 2.2)	0.1 (0.0 - 0.3)	0.0 (0.0 - 0.0)
Mental disorders & substance use	Opioid use disorders	Male	All	27831	NR	95.3 (95.0 - 95.5)	90.1 (89.7 - 90.4)	86.0 (85.6 - 86.4)	76.6 (76.0 - 77.2)	67.8 (67.0 - 68.6)	59.9 (58.8 - 61.0)
Mental disorders & substance use	Opioid use disorders	Male	16-24	6230	NR	97.8 (97.4 - 98.2)	94.4 (93.8 - 94.9)	91.6 (90.8 - 92.3)	84.9 (83.8 - 86.0)	78.8 (77.2 - 80.3)	74.2 (72.1 - 76.2)

Appendix Table A6: Unadjusted Median Survival and Survival Probabilities by Age, males (Page 14 of 18)

Group	Diagnosis Category	Sex	Age	N	Median Survival (y)	1y (%)	3y (%)	5y (%)	10y (%)	15y (%)	20y (%)
Mental disorders & substance use	Opioid use disorders	Male	25-34	8407	NR	97.3 (96.9 - 97.6)	93.9 (93.3 - 94.4)	90.9 (90.2 - 91.5)	84.3 (83.4 - 85.2)	78.3 (77.1 - 79.5)	72.5 (70.7 - 74.3)
Mental disorders & substance use	Opioid use disorders	Male	35-44	4915	NR	97.3 (96.8 - 97.7)	94.0 (93.3 - 94.7)	90.7 (89.8 - 91.5)	83.1 (81.9 - 84.3)	74.0 (72.3 - 75.6)	65.8 (63.4 - 68.0)
Mental disorders & substance use	Opioid use disorders	Male	45-54	3849	20.31 (21.25 - 19.14)	95.9 (95.3 - 96.5)	90.7 (89.7 - 91.6)	86.3 (85.1 - 87.4)	74.3 (72.6 - 75.8)	62.3 (60.2 - 64.3)	51.1 (47.9 - 54.2)
Mental disorders & substance use	Opioid use disorders	Male	55-64	2338	13.73 (14.71 - 12.78)	92.4 (91.2 - 93.4)	83.9 (82.3 - 85.4)	77.0 (75.2 - 78.8)	60.9 (58.5 - 63.2)	45.3 (42.2 - 48.3)	23.1 (17.9 - 28.6)
Mental disorders & substance use	Opioid use disorders	Male	65-74	1236	7.17 (7.75 - 6.58)	85.6 (83.5 - 87.4)	70.6 (67.9 - 73.2)	60.9 (57.9 - 63.7)	39.7 (36.3 - 43.1)	21.8 (17.9 - 25.9)	4.9 (1.7 - 10.9)
Mental disorders & substance use	Opioid use disorders	Male	75-84	607	2.58 (3.17 - 2.08)	69.0 (65.0 - 72.5)	46.3 (42.0 - 50.4)	34.8 (30.6 - 38.9)	11.7 (8.6 - 15.4)	1.4 (0.2 - 5.9)	0.0 (0.0 - 0.0)
Mental disorders & substance use	Opioid use disorders	Male	85-95	243	1.2 (1.39 - 0.94)	54.6 (48.1 - 60.7)	21.7 (16.6 - 27.4)	10.9 (7.1 - 15.6)	0.0 (0.0 - 0.0)	0.0 (0.0 - 0.0)	0.0 (0.0 - 0.0)
Mental disorders & substance use	Schizophrenia	Male	All	22480	NR	96.1 (95.8 - 96.3)	91.7 (91.3 - 92.1)	87.9 (87.5 - 88.3)	78.4 (77.8 - 79.0)	68.7 (68.0 - 69.4)	58.3 (57.4 - 59.2)
Mental disorders & substance use	Schizophrenia	Male	16-24	2767	NR	99.1 (98.6 - 99.4)	97.5 (96.8 - 98.0)	96.2 (95.4 - 96.9)	93.6 (92.5 - 94.5)	91.1 (89.7 - 92.3)	87.1 (84.8 - 89.0)
Mental disorders & substance use	Schizophrenia	Male	25-34	5082	NR	99.1 (98.8 - 99.3)	97.4 (96.9 - 97.8)	96.2 (95.6 - 96.7)	93.1 (92.3 - 93.8)	89.7 (88.6 - 90.7)	86.0 (84.4 - 87.4)
Mental disorders & substance use	Schizophrenia	Male	35-44	4716	NR	98.7 (98.3 - 99.0)	96.9 (96.3 - 97.3)	95.5 (94.8 - 96.1)	91.2 (90.3 - 92.0)	85.5 (84.4 - 86.6)	78.5 (76.9 - 80.0)
Mental disorders & substance use	Schizophrenia	Male	45-54	4365	21.56 (22.36 - 20.94)	97.5 (97.0 - 97.9)	93.9 (93.1 - 94.5)	91.0 (90.1 - 91.9)	81.6 (80.3 - 82.7)	70.1 (68.6 - 71.6)	54.5 (52.4 - 56.5)
Mental disorders & substance use	Schizophrenia	Male	55-64	3126	13.45 (13.89 - 12.97)	94.2 (93.3 - 95.0)	87.6 (86.4 - 88.8)	81.5 (80.1 - 82.8)	63.6 (61.8 - 65.3)	44.0 (42.0 - 45.9)	25.6 (23.5 - 27.9)
Mental disorders & substance use	Schizophrenia	Male	65-74	1563	7.11 (7.44 - 6.61)	87.5 (85.8 - 89.1)	75.2 (72.9 - 77.3)	61.4 (58.9 - 63.9)	35.3 (32.7 - 37.8)	16.3 (14.2 - 18.6)	4.8 (3.2 - 6.9)
Mental disorders & substance use	Schizophrenia	Male	75-84	732	2.96 (3.4 - 2.65)	72.9 (69.5 - 75.9)	49.6 (45.9 - 53.2)	33.7 (30.2 - 37.2)	10.9 (8.6 - 13.5)	2.7 (1.5 - 4.4)	1.0 (0.3 - 2.5)
Mental disorders & substance use	Schizophrenia	Male	85-95	129	1.48 (1.81 - 0.65)	54.1 (45.1 - 62.2)	27.4 (20.0 - 35.3)	13.3 (8.1 - 19.8)	0.8 (0.1 - 3.9)	0.8 (0.1 - 3.9)	0.8 (0.1 - 3.9)
Mental disorders & substance use	Self-harm	Male	All	59401	NR	94.2 (94.1 - 94.4)	89.3 (89.0 - 89.5)	85.1 (84.8 - 85.4)	77.1 (76.7 - 77.4)	70.0 (69.6 - 70.5)	63.4 (62.8 - 64.0)
Mental disorders & substance use	Self-harm	Male	16-24	16348	NR	98.4 (98.2 - 98.6)	96.5 (96.2 - 96.7)	94.8 (94.4 - 95.1)	91.5 (91.0 - 92.0)	88.5 (87.9 - 89.1)	86.0 (85.1 - 86.8)
Mental disorders & substance use	Self-harm	Male	25-34	12909	NR	96.9 (96.6 - 97.2)	94.0 (93.6 - 94.4)	91.9 (91.4 - 92.4)	87.4 (86.8 - 88.1)	83.2 (82.4 - 84.0)	79.5 (78.4 - 80.6)
Mental disorders & substance use	Self-harm	Male	35-44	9352	NR	96.2 (95.8 - 96.6)	93.0 (92.4 - 93.5)	90.4 (89.7 - 91.0)	84.4 (83.5 - 85.2)	78.7 (77.7 - 79.7)	72.0 (70.6 - 73.3)
Mental disorders & substance use	Self-harm	Male	45-54	8252	21.72 (22.74 - 20.83)	94.5 (94.0 - 95.0)	89.6 (88.9 - 90.3)	85.7 (84.9 - 86.5)	76.1 (75.1 - 77.1)	65.4 (64.1 - 66.7)	53.7 (51.9 - 55.5)
Mental disorders & substance use	Self-harm	Male	55-64	5644	14.76 (15.41 - 14.19)	91.9 (91.2 - 92.6)	84.9 (83.9 - 85.8)	78.4 (77.2 - 79.5)	63.7 (62.3 - 65.1)	49.5 (47.8 - 51.2)	36.1 (34.0 - 38.3)
Mental disorders & substance use	Self-harm	Male	65-74	3362	7.66 (8.05 - 7.26)	85.6 (84.3 - 86.7)	73.3 (71.7 - 74.8)	61.6 (59.9 - 63.4)	39.9 (37.9 - 41.8)	22.4 (20.4 - 24.4)	9.6 (7.7 - 11.8)
Mental disorders & substance use	Self-harm	Male	75-84	2353	3.85 (4.09 - 3.66)	76.9 (75.1 - 78.5)	57.9 (55.8 - 60.0)	40.8 (38.6 - 42.9)	14.4 (12.6 - 16.2)	2.5 (1.7 - 3.6)	0.4 (0.1 - 1.3)
Mental disorders & substance use	Self-harm	Male	85-95	1139	1.75 (1.94 - 1.46)	60.9 (58.0 - 63.7)	34.0 (31.2 - 36.9)	17.5 (15.2 - 20.0)	1.5 (0.7 - 2.9)	0.0 (0.0 - 0.0)	0.0 (0.0 - 0.0)
Musculoskeletal disorders	Gout	Male	All	93093	9.22 (9.35 - 9.1)	88.3 (88.1 - 88.5)	75.7 (75.4 - 76.0)	65.7 (65.4 - 66.0)	47.5 (47.2 - 47.9)	35.1 (34.6 - 35.5)	26.9 (26.3 - 27.4)
Musculoskeletal disorders	Gout	Male	16-24	290	NR	99.6 (97.5 - 100.0)	99.3 (97.2 - 99.8)	98.9 (96.6 - 99.6)	97.2 (93.6 - 98.7)	92.4 (86.1 - 95.9)	92.4 (86.1 - 95.9)
Musculoskeletal disorders	Gout	Male	25-34	1776	NR	99.5 (99.1 - 99.8)	99.0 (98.4 - 99.4)	98.4 (97.6 - 98.9)	96.7 (95.5 - 97.6)	94.9 (93.1 - 96.2)	92.1 (89.3 - 94.1)
Musculoskeletal disorders	Gout	Male	35-44	4486	NR	99.4 (99.2 - 99.6)	98.6 (98.2 - 98.9)	97.6 (97.1 - 98.1)	95.2 (94.4 - 95.8)	90.9 (89.6 - 92.0)	86.1 (84.1 - 87.8)
Musculoskeletal disorders	Gout	Male	45-54	9048	NR	98.5 (98.3 - 98.8)	96.5 (96.1 - 96.9)	94.5 (94.0 - 95.0)	88.8 (88.0 - 89.5)	80.7 (79.5 - 81.9)	71.9 (70.1 - 73.7)
Musculoskeletal disorders	Gout	Male	55-64	15598	18.16 (18.62 - 17.77)	97.2 (96.9 - 97.4)	92.6 (92.2 - 93.0)	87.9 (87.4 - 88.5)	75.5 (74.7 - 76.3)	60.4 (59.2 - 61.5)	44.4 (42.7 - 46.0)
Musculoskeletal disorders	Gout	Male	65-74	23706	10.37 (10.58 - 10.19)	92.9 (92.6 - 93.2)	83.5 (83.0 - 84.0)	74.7 (74.1 - 75.3)	51.7 (50.9 - 52.5)	30.1 (29.1 - 31.1)	13.4 (12.2 - 14.7)
Musculoskeletal disorders	Gout	Male	75-84	25606	4.77 (4.85 - 4.7)	83.7 (83.2 - 84.1)	64.5 (63.9 - 65.1)	48.2 (47.5 - 48.8)	19.0 (18.4 - 19.6)	4.6 (4.1 - 5.1)	0.5 (0.3 - 0.9)
Musculoskeletal disorders	Gout	Male	85-95	12179	1.91 (1.97 - 1.86)	65.4 (64.6 - 66.3)	35.9 (35.0 - 36.8)	17.9 (17.2 - 18.7)	2.3 (2.0 - 2.7)	0.1 (0.0 - 0.3)	0.0 (0.0 - 0.2)
Musculoskeletal disorders	Low back pain	Male	All	263321	NR	97.5 (97.5 - 97.6)	94.3 (94.2 - 94.4)	91.2 (91.1 - 91.3)	84.0 (83.8 - 84.1)	77.0 (76.8 - 77.3)	70.5 (70.2 - 70.8)
Musculoskeletal disorders	Low back pain	Male	16-24	13741	NR	99.8 (99.7 - 99.9)	99.5 (99.3 - 99.6)	99.1 (98.9 - 99.3)	98.4 (98.1 - 98.6)	97.6 (97.2 - 97.9)	96.9 (96.4 - 97.3)
Musculoskeletal disorders	Low back pain	Male	25-34	35694	NR	99.8 (99.8 - 99.9)	99.5 (99.5 - 99.6)	99.3 (99.2 - 99.4)	98.5 (98.4 - 98.7)	97.7 (97.5 - 97.9)	96.8 (96.4 - 97.0)
Musculoskeletal disorders	Low back pain	Male	35-44	51480	NR	99.8 (99.7 - 99.8)	99.4 (99.3 - 99.5)	99.0 (98.9 - 99.0)	97.9 (97.8 - 98.1)	96.2 (96.0 - 96.4)	94.1 (93.8 - 94.4)
Musculoskeletal disorders	Low back pain	Male	45-54	51680	NR	99.4 (99.3 - 99.5)	98.6 (98.5 - 98.7)	97.8 (97.6 - 97.9)	95.0 (94.8 - 95.3)	91.0 (90.6 - 91.3)	85.3 (84.8 - 85.9)
Musculoskeletal disorders	Low back pain	Male	55-64	47114	NR	98.4 (98.3 - 98.5)	96.3 (96.1 - 96.4)	94.1 (93.9 - 94.3)	87.3 (86.9 - 87.6)	77.8 (77.2 - 78.3)	64.7 (63.9 - 65.5)
Musculoskeletal disorders	Low back pain	Male	65-74	33725	14.31 (14.49 - 14.17)	95.7 (95.4 - 95.9)	90.1 (89.8 - 90.4)	84.1 (83.6 - 84.5)	67.4 (66.8 - 68.0)	47.1 (46.3 - 47.9)	25.6 (24.5 - 26.7)
Musculoskeletal disorders	Low back pain	Male	75-84	22887	6.99 (7.1 - 6.87)	90.4 (90.0 - 90.7)	77.1 (76.5 - 77.6)	63.6 (62.9 - 64.3)	31.5 (30.8 - 32.3)	10.1 (9.4 - 10.7)	2.3 (1.9 - 2.9)
Musculoskeletal disorders	Low back pain	Male	85-95	6811	3.02 (3.12 - 2.91)	77.3 (76.2 - 78.3)	50.3 (49.0 - 51.5)	30.4 (29.3 - 31.6)	5.0 (4.4 - 5.8)	0.4 (0.2 - 0.8)	0.1 (0.0 - 0.4)
Musculoskeletal disorders	Lupus	Male	All	3445	20.95	96.0 (95.3 - 96.6)	90.1 (89.0 - 91.1)	84.8 (83.5 - 86.0)	73.0 (71.4 - 74.6)	62.7 (60.7 - 64.6)	51.8 (49.3 - 54.1)
Musculoskeletal disorders	Lupus	Male	16-24	175	NR	99.4 (95.9 - 99.9)	98.8 (95.3 - 99.7)	98.1 (94.4 - 99.4)	96.6 (91.9 - 98.6)	95.2 (89.0 - 97.9)	95.2 (89.0 - 97.9)
Musculoskeletal disorders	Lupus	Male	25-34	295	NR	100.0 (100.0 - 100.0)	99.6 (97.5 - 99.9)	99.3 (97.0 - 99.8)	98.4 (95.9 - 99.4)	95.9 (91.7 - 98.0)	95.9 (91.7 - 98.0)
Musculoskeletal disorders	Lupus	Male	35-44	395	NR	99.2 (97.6 - 99.7)	98.4 (96.5 - 99.3)	98.1 (96.2 - 99.1)	96.2 (93.5 - 97.8)	95.0 (92.0 - 97.0)	91.2 (86.5 - 94.3)
Musculoskeletal disorders	Lupus	Male	45-54	554	NR	98.9 (97.6 - 99.5)	96.8 (94.9 - 98.0)	94.6 (92.2 - 96.2)	89.5 (86.3 - 91.9)	81.7 (77.4 - 85.3)	68.5 (62.4 - 73.9)
Musculoskeletal disorders	Lupus	Male	55-64	809	19.46 (21.2 - 18.64)	97.0 (95.6 - 98.0)	93.3 (91.3 - 94.8)	89.0 (86.5 - 91.0)	77.6 (74.3 - 80.6)	65.6 (61.6 - 69.3)	48.4 (43.3 - 53.3)
Musculoskeletal disorders	Lupus	Male	65-74	737	11.74 (12.98 - 10.48)	95.7 (94.0 - 97.0)	86.1 (83.3 - 88.5)	76.7 (73.3 - 79.8)	56.2 (52.0 - 60.2)	37.0 (32.4 - 41.6)	20.0 (15.3 - 25.2)

Appendix Table A6: Unadjusted Median Survival and Survival Probabilities by Age, males (Page 15 of 18)

Group	Diagnosis Category	Sex	Age	N	Median Survival (y)	1y (%)	3y (%)	5y (%)	10y (%)	15y (%)	20y (%)
Musculoskeletal disorders	Lupus	Male	75-84	401	5.92 (6.68 - 5.38)	86.8 (83.1 - 89.8)	71.9 (67.0 - 76.2)	59.4 (54.0 - 64.3)	26.6 (21.4 - 32.1)	6.1 (3.2 - 10.3)	1.5 (0.3 - 4.8)
Musculoskeletal disorders	Lupus	Male	85-95	79	2.08 (3.04 - 1.72)	74.1 (62.8 - 82.5)	40.2 (29.1 - 51.1)	18.1 (10.1 - 28.0)	0.0 (0.0 - 0.0)	0.0 (0.0 - 0.0)	0.0 (0.0 - 0.0)
Musculoskeletal disorders	Neck pain	Male	All	109300	NR	98.4 (98.4 - 98.5)	96.3 (96.2 - 96.5)	94.3 (94.1 - 94.4)	88.6 (88.4 - 88.8)	82.4 (82.0 - 82.7)	75.3 (74.8 - 75.8)
Musculoskeletal disorders	Neck pain	Male	16-24	6367	NR	99.9 (99.8 - 99.9)	99.7 (99.5 - 99.8)	99.4 (99.1 - 99.5)	98.5 (98.1 - 98.9)	97.9 (97.4 - 98.3)	96.4 (95.4 - 97.3)
Musculoskeletal disorders	Neck pain	Male	25-34	12318	NR	99.8 (99.7 - 99.9)	99.6 (99.5 - 99.7)	99.4 (99.2 - 99.5)	98.7 (98.4 - 98.9)	97.6 (97.2 - 98.0)	96.9 (96.3 - 97.3)
Musculoskeletal disorders	Neck pain	Male	35-44	20837	NR	99.8 (99.7 - 99.9)	99.5 (99.4 - 99.6)	99.2 (99.0 - 99.3)	98.0 (97.7 - 98.2)	96.4 (96.1 - 96.7)	93.3 (92.6 - 93.9)
Musculoskeletal disorders	Neck pain	Male	45-54	27230	NR	99.6 (99.5 - 99.7)	98.8 (98.7 - 98.9)	98.1 (97.9 - 98.3)	95.7 (95.4 - 96.0)	91.8 (91.4 - 92.3)	85.4 (84.5 - 86.2)
Musculoskeletal disorders	Neck pain	Male	55-64	23033	NR	98.9 (98.8 - 99.1)	97.3 (97.1 - 97.5)	95.5 (95.2 - 95.7)	89.6 (89.2 - 90.1)	80.7 (80.0 - 81.5)	68.3 (67.1 - 69.5)
Musculoskeletal disorders	Neck pain	Male	65-74	11566	15.03 (15.45 - 14.63)	96.7 (96.3 - 97.0)	92.2 (91.7 - 92.7)	87.0 (86.4 - 87.7)	71.2 (70.2 - 72.2)	50.1 (48.6 - 51.6)	27.2 (25.0 - 29.4)
Musculoskeletal disorders	Neck pain	Male	75-84	6283	7.9 (8.1 - 7.65)	91.5 (90.8 - 92.2)	80.0 (78.9 - 81.0)	68.3 (67.0 - 69.5)	36.1 (34.5 - 37.6)	12.8 (11.3 - 14.4)	2.7 (1.6 - 4.2)
Musculoskeletal disorders	Neck pain	Male	85-95	1618	3.32 (3.55 - 3.02)	78.9 (76.8 - 80.9)	53.2 (50.6 - 55.7)	33.8 (31.3 - 36.4)	6.1 (4.6 - 7.8)	0.9 (0.3 - 2.5)	0.9 (0.3 - 2.5)
Musculoskeletal disorders	Osteoarthritis	Male	All	365672	20.74 (20.86 - 20.63)	98.3 (98.2 - 98.3)	94.8 (94.7 - 94.8)	90.7 (90.6 - 90.8)	78.7 (78.5 - 78.8)	65.3 (65.1 - 65.5)	51.8 (51.5 - 52.1)
Musculoskeletal disorders	Osteoarthritis	Male	16-24	2262	NR	100.0 (100.0 - 100.0)	99.8 (99.4 - 99.9)	99.6 (99.2 - 99.8)	99.5 (99.0 - 99.7)	98.9 (98.2 - 99.3)	98.9 (98.2 - 99.3)
Musculoskeletal disorders	Osteoarthritis	Male	25-34	7367	NR	99.9 (99.8 - 100.0)	99.8 (99.6 - 99.8)	99.6 (99.4 - 99.7)	99.1 (98.8 - 99.3)	98.3 (97.9 - 98.6)	97.7 (97.1 - 98.1)
Musculoskeletal disorders	Osteoarthritis	Male	35-44	22857	NR	99.9 (99.8 - 99.9)	99.6 (99.5 - 99.7)	99.2 (99.1 - 99.3)	98.2 (98.0 - 98.4)	96.7 (96.5 - 97.0)	94.4 (93.8 - 94.8)
Musculoskeletal disorders	Osteoarthritis	Male	45-54	59020	NR	99.8 (99.7 - 99.8)	99.2 (99.1 - 99.2)	98.4 (98.3 - 98.5)	96.0 (95.8 - 96.2)	92.3 (92.1 - 92.6)	86.5 (86.0 - 87.0)
Musculoskeletal disorders	Osteoarthritis	Male	55-64	101451	NR	99.5 (99.4 - 99.5)	98.2 (98.1 - 98.3)	96.5 (96.4 - 96.6)	90.7 (90.5 - 90.9)	81.7 (81.4 - 82.0)	67.4 (66.9 - 67.9)
Musculoskeletal disorders	Osteoarthritis	Male	65-74	100952	15.93 (16.01 - 15.84)	98.7 (98.6 - 98.8)	95.5 (95.4 - 95.6)	91.3 (91.2 - 91.5)	76.8 (76.5 - 77.2)	54.8 (54.4 - 55.3)	28.8 (28.2 - 29.5)
Musculoskeletal disorders	Osteoarthritis	Male	75-84	59712	9.03 (9.1 - 8.96)	95.9 (95.8 - 96.1)	87.2 (86.9 - 87.5)	76.1 (75.7 - 76.5)	43.4 (42.9 - 43.9)	15.0 (14.5 - 15.4)	2.5 (2.2 - 2.8)
Musculoskeletal disorders	Osteoarthritis	Male	85-95	11826	4.17 (4.27 - 4.07)	84.6 (83.9 - 85.2)	62.0 (61.1 - 62.9)	42.3 (41.3 - 43.2)	9.8 (9.2 - 10.5)	0.9 (0.7 - 1.2)	0.0 (0.0 - 0.0)
Musculoskeletal disorders	Rheumatoid arthritis	Male	All	38292	17.38 (17.69 - 17.1)	95.9 (95.7 - 96.1)	90.0 (89.7 - 90.3)	84.1 (83.7 - 84.5)	69.2 (68.7 - 69.7)	55.7 (55.1 - 56.4)	43.7 (42.9 - 44.4)
Musculoskeletal disorders	Rheumatoid arthritis	Male	16-24	1007	NR	99.8 (99.2 - 99.9)	99.4 (98.6 - 99.7)	99.1 (98.3 - 99.6)	98.5 (97.4 - 99.2)	98.2 (96.9 - 98.9)	97.4 (95.8 - 98.4)
Musculoskeletal disorders	Rheumatoid arthritis	Male	25-34	1607	NR	99.9 (99.5 - 100.0)	99.8 (99.4 - 99.9)	99.4 (98.9 - 99.7)	98.8 (98.0 - 99.3)	97.2 (96.1 - 98.1)	95.3 (93.5 - 96.6)
Musculoskeletal disorders	Rheumatoid arthritis	Male	35-44	2863	NR	99.9 (99.6 - 99.9)	99.3 (98.9 - 99.5)	98.6 (98.1 - 99.0)	96.8 (96.0 - 97.4)	94.1 (92.9 - 95.1)	90.9 (89.2 - 92.3)
Musculoskeletal disorders	Rheumatoid arthritis	Male	45-54	5150	NR	99.3 (99.1 - 99.5)	98.0 (97.6 - 98.4)	97.0 (96.5 - 97.5)	92.3 (91.5 - 93.1)	86.3 (85.1 - 87.4)	77.0 (75.2 - 78.6)
Musculoskeletal disorders	Rheumatoid arthritis	Male	55-64	9015	20.74 (21.13 - 20.17)	98.5 (98.2 - 98.7)	96.1 (95.7 - 96.5)	92.9 (92.4 - 93.5)	83.7 (82.8 - 84.5)	70.7 (69.5 - 71.8)	52.3 (50.6 - 53.8)
Musculoskeletal disorders	Rheumatoid arthritis	Male	65-74	10171	12.65 (12.95 - 12.38)	96.7 (96.3 - 97.0)	90.3 (89.7 - 90.9)	83.6 (82.8 - 84.4)	63.0 (61.8 - 64.0)	38.7 (37.4 - 40.0)	17.1 (15.7 - 18.5)
Musculoskeletal disorders	Rheumatoid arthritis	Male	75-84	7058	6.65 (6.81 - 6.49)	90.7 (90.0 - 91.4)	76.6 (75.6 - 77.6)	62.5 (61.3 - 63.7)	28.2 (27.0 - 29.5)	8.0 (7.1 - 9.0)	1.4 (0.9 - 2.1)
Musculoskeletal disorders	Rheumatoid arthritis	Male	85-95	1400	2.72 (2.94 - 2.48)	73.4 (71.0 - 75.7)	46.6 (43.8 - 49.3)	28.6 (26.1 - 31.2)	4.2 (3.0 - 5.7)	0.2 (0.0 - 1.0)	0.2 (0.0 - 1.0)
Neurological disorders	Alzheimer's disease	Male	All	53189	3.53 (3.57 - 3.48)	80.7 (80.3 - 81.0)	56.0 (55.6 - 56.5)	35.4 (35.0 - 35.9)	8.8 (8.5 - 9.1)	2.4 (2.2 - 2.6)	1.1 (0.9 - 1.2)
Neurological disorders	Alzheimer's disease	Male	16-24	8	NR	100.0 (100.0 - 100.0)	100.0 (100.0 - 100.0)	80.0 (20.4 - 96.9)	80.0 (20.4 - 96.9)	80.0 (20.4 - 96.9)	80.0 (20.4 - 96.9)
Neurological disorders	Alzheimer's disease	Male	25-34	16	NR	100.0 (100.0 - 100.0)	100.0 (100.0 - 100.0)	100.0 (100.0 - 100.0)	100.0 (100.0 - 100.0)	100.0 (100.0 - 100.0)	100.0 (100.0 - 100.0)
Neurological disorders	Alzheimer's disease	Male	35-44	41	NR	100.0 (100.0 - 100.0)	91.9 (76.9 - 97.3)	80.3 (63.0 - 90.1)	59.7 (40.5 - 74.5)	51.3 (31.9 - 67.7)	51.3 (31.9 - 67.7)
Neurological disorders	Alzheimer's disease	Male	45-54	356	8.69 (9.88 - 7.81)	96.0 (93.3 - 97.6)	87.8 (83.7 - 90.9)	75.7 (70.4 - 80.2)	43.3 (36.8 - 49.6)	28.6 (22.4 - 35.2)	21.9 (15.8 - 28.6)
Neurological disorders	Alzheimer's disease	Male	55-64	2494	8.06 (8.31 - 7.8)	96.4 (95.6 - 97.1)	89.2 (87.8 - 90.4)	75.6 (73.7 - 77.4)	35.6 (33.3 - 38.0)	14.3 (12.3 - 16.4)	8.0 (6.2 - 10.0)
Neurological disorders	Alzheimer's disease	Male	65-74	11036	5.62 (5.73 - 5.49)	92.0 (91.5 - 92.5)	75.5 (74.6 - 76.3)	55.3 (54.2 - 56.3)	17.7 (16.8 - 18.6)	4.6 (4.0 - 5.2)	1.2 (0.8 - 1.7)
Neurological disorders	Alzheimer's disease	Male	75-84	26245	3.5 (3.56 - 3.45)	82.3 (81.8 - 82.7)	56.3 (55.7 - 56.9)	33.4 (32.8 - 34.1)	5.6 (5.3 - 6.0)	0.8 (0.6 - 1.0)	0.2 (0.1 - 0.3)
Neurological disorders	Alzheimer's disease	Male	85-95	12793	1.75 (1.81 - 1.71)	64.6 (63.8 - 65.5)	31.6 (30.8 - 32.5)	13.1 (12.4 - 13.7)	0.6 (0.5 - 0.8)	0.0 (0.0 - 0.1)	0.0 (0.0 - 0.1)
Neurological disorders	Alzheimer's disease and other dementias	Male	All	155265	2.4 (2.42 - 2.38)	69.3 (69.1 - 69.6)	43.1 (42.8 - 43.3)	25.5 (25.3 - 25.7)	6.9 (6.7 - 7.1)	2.5 (2.4 - 2.6)	1.4 (1.3 - 1.5)
Neurological disorders	Alzheimer's disease and other dementias	Male	16-24	88	NR	98.8 (92.0 - 99.8)	96.5 (89.5 - 98.9)	93.9 (86.0 - 97.4)	87.8 (77.5 - 93.5)	87.8 (77.5 - 93.5)	84.1 (70.6 - 91.7)
Neurological disorders	Alzheimer's disease and other dementias	Male	25-34	183	NR	98.3 (94.9 - 99.5)	97.1 (93.3 - 98.8)	95.3 (90.8 - 97.6)	90.4 (84.6 - 94.1)	88.0 (81.0 - 92.5)	83.7 (73.7 - 90.2)
Neurological disorders	Alzheimer's disease and other dementias	Male	35-44	336	NR	98.2 (96.0 - 99.2)	91.8 (88.2 - 94.3)	87.3 (83.0 - 90.6)	78.8 (73.6 - 83.2)	67.9 (61.5 - 73.4)	61.0 (53.5 - 67.6)
Neurological disorders	Alzheimer's disease and other dementias	Male	45-54	1352	11.1 (12.16 - 10.27)	91.7 (90.1 - 93.0)	82.4 (80.2 - 84.3)	74.1 (71.5 - 76.4)	53.7 (50.7 - 56.7)	40.6 (37.3 - 43.8)	31.6 (27.9 - 35.3)
Neurological disorders	Alzheimer's disease and other dementias	Male	55-64	6260	7.78 (7.99 - 7.57)	91.9 (91.2 - 92.6)	81.0 (80.0 - 82.0)	67.9 (66.6 - 69.1)	36.1 (34.7 - 37.6)	17.9 (16.6 - 19.2)	10.1 (8.8 - 11.5)
Neurological disorders	Alzheimer's disease and other dementias	Male	65-74	26192	4.67 (4.74 - 4.61)	85.9 (85.5 - 86.3)	66.7 (66.1 - 67.3)	46.9 (46.2 - 47.5)	15.1 (14.6 - 15.7)	4.1 (3.7 - 4.4)	1.0 (0.8 - 1.3)
Neurological disorders	Alzheimer's disease and other dementias	Male	75-84	70148	2.62 (2.65 - 2.6)	72.9 (72.6 - 73.2)	45.2 (44.9 - 45.6)	24.9 (24.6 - 25.3)	3.9 (3.8 - 4.1)	0.5 (0.5 - 0.6)	0.1 (0.1 - 0.2)
Neurological disorders	Alzheimer's disease and other dementias	Male	85-95	49221	1.14 (1.16 - 1.12)	52.7 (52.3 - 53.2)	21.7 (21.3 - 22.1)	7.8 (7.5 - 8.0)	0.3 (0.3 - 0.4)	0.0 (0.0 - 0.1)	0.0 (0.0 - 0.1)
Neurological disorders	Dementia as a sign with others	Male	All	48891	3.61 (3.65 - 3.57)	82.3 (82.0 - 82.6)	57.4 (56.9 - 57.8)	35.8 (35.3 - 36.3)	8.7 (8.4 - 9.0)	2.4 (2.2 - 2.6)	1.1 (0.9 - 1.3)
Neurological disorders	Dementia as a sign with others	Male	16-24	8	NR	87.5 (38.7 - 98.1)	87.5 (38.7 - 98.1)	87.5 (38.7 - 98.1)	87.5 (38.7 - 98.1)	87.5 (38.7 - 98.1)	87.5 (38.7 - 98.1)
Neurological disorders	Dementia as a sign with others	Male	25-34	16	NR	100.0 (100.0 - 100.0)	100.0 (100.0 - 100.0)	100.0 (100.0 - 100.0)	91.7 (53.9 - 98.8)	91.7 (53.9 - 98.8)	68.8 (16.3 - 92.6)

Appendix Table A6: Unadjusted Median Survival and Survival Probabilities by Age, males (Page 16 of 18)

Group	Diagnosis Category	Sex	Age	N	Median Survival (y)	1y (%)	3y (%)	5y (%)	10y (%)	15y (%)	20y (%)
Neurological disorders	Dementia as a sign with others	Male	35-44	54	9.58	88.7 (76.6 - 94.8)	78.7 (64.8 - 87.6)	65.7 (50.6 - 77.2)	48.3 (32.9 - 62.1)	45.4 (30.1 - 59.5)	45.4 (30.1 - 59.5)
Neurological disorders	Dementia as a sign with others	Male	45-54	439	8.18 (9.43 - 7.47)	93.1 (90.2 - 95.1)	83.1 (79.1 - 86.4)	71.8 (66.9 - 76.0)	42.2 (36.5 - 47.8)	26.6 (21.3 - 32.3)	21.4 (16.0 - 27.4)
Neurological disorders	Dementia as a sign with others	Male	55-64	2658	7.76 (7.98 - 7.49)	96.4 (95.6 - 97.1)	87.0 (85.6 - 88.3)	72.0 (70.1 - 73.8)	32.0 (29.8 - 34.2)	12.5 (10.7 - 14.5)	7.1 (5.4 - 9.0)
Neurological disorders	Dementia as a sign with others	Male	65-74	11069	5.3 (5.42 - 5.18)	92.2 (91.7 - 92.7)	74.6 (73.7 - 75.4)	52.7 (51.7 - 53.8)	15.6 (14.8 - 16.5)	3.9 (3.3 - 4.4)	0.9 (0.6 - 1.4)
Neurological disorders	Dementia as a sign with others	Male	75-84	23744	3.49 (3.55 - 3.44)	83.4 (82.9 - 83.9)	56.6 (56.0 - 57.3)	33.1 (32.4 - 33.8)	5.4 (5.0 - 5.8)	0.7 (0.5 - 0.9)	0.2 (0.1 - 0.3)
Neurological disorders	Dementia as a sign with others	Male	85-95	10742	1.85 (1.91 - 1.8)	66.3 (65.4 - 67.2)	33.1 (32.2 - 34.1)	13.7 (13.0 - 14.5)	0.6 (0.5 - 0.9)	0.0 (0.0 - 0.1)	0.0 (0.0 - 0.1)
Neurological disorders	Idiopathic epilepsy	Male	All	78915	12.87 (13.12 - 12.64)	86.3 (86.1 - 86.6)	75.7 (75.4 - 76.0)	68.4 (68.1 - 68.7)	55.6 (55.2 - 55.9)	46.6 (46.2 - 47.0)	40.2 (39.7 - 40.6)
Neurological disorders	Idiopathic epilepsy	Male	16-24	7585	NR	98.9 (98.7 - 99.2)	97.6 (97.2 - 97.9)	96.7 (96.2 - 97.1)	94.4 (93.9 - 95.0)	92.5 (91.8 - 93.2)	90.3 (89.4 - 91.2)
Neurological disorders	Idiopathic epilepsy	Male	25-34	7500	NR	98.1 (97.7 - 98.4)	95.8 (95.3 - 96.3)	93.6 (93.0 - 94.2)	90.1 (89.3 - 90.8)	87.2 (86.3 - 88.0)	84.7 (83.7 - 85.7)
Neurological disorders	Idiopathic epilepsy	Male	35-44	7670	NR	96.4 (95.9 - 96.8)	92.7 (92.0 - 93.2)	90.2 (89.5 - 90.9)	84.3 (83.4 - 85.1)	78.7 (77.7 - 79.7)	73.0 (71.8 - 74.2)
Neurological disorders	Idiopathic epilepsy	Male	45-54	10134	21.33 (22.03 - 20.69)	92.8 (92.3 - 93.3)	85.8 (85.1 - 86.5)	81.5 (80.7 - 82.3)	72.1 (71.2 - 73.0)	63.0 (61.9 - 64.0)	52.6 (51.4 - 53.9)
Neurological disorders	Idiopathic epilepsy	Male	55-64	13008	12.67 (13.06 - 12.32)	87.7 (87.1 - 88.2)	77.7 (77.0 - 78.5)	71.1 (70.3 - 71.9)	57.1 (56.1 - 58.0)	43.7 (42.7 - 44.7)	31.9 (30.7 - 33.0)
Neurological disorders	Idiopathic epilepsy	Male	65-74	14905	7.17 (7.37 - 7.01)	82.5 (81.9 - 83.1)	69.7 (69.0 - 70.5)	59.7 (58.9 - 60.6)	38.5 (37.6 - 39.4)	20.9 (20.0 - 21.8)	9.7 (8.8 - 10.6)
Neurological disorders	Idiopathic epilepsy	Male	75-84	13321	3.55 (3.65 - 3.46)	74.7 (73.9 - 75.4)	55.0 (54.1 - 55.9)	39.5 (38.6 - 40.4)	15.2 (14.5 - 16.0)	4.6 (4.0 - 5.1)	0.8 (0.5 - 1.2)
Neurological disorders	Idiopathic epilepsy	Male	85-95	4704	1.46 (1.54 - 1.38)	58.5 (57.1 - 59.9)	29.8 (28.4 - 31.2)	15.2 (14.1 - 16.4)	2.6 (2.1 - 3.2)	0.2 (0.0 - 0.4)	0.0 (0.0 - 0.0)
Neurological disorders	Motor neuron disease	Male	All	5938	1.76 (1.85 - 1.68)	64.5 (63.3 - 65.7)	38.4 (37.1 - 39.6)	29.7 (28.5 - 31.0)	21.0 (19.9 - 22.2)	15.7 (14.6 - 16.9)	12.5 (11.4 - 13.7)
Neurological disorders	Motor neuron disease	Male	16-24	85	NR	98.8 (91.9 - 99.8)	98.8 (91.9 - 99.8)	97.4 (90.0 - 99.4)	92.2 (82.1 - 96.7)	81.3 (67.5 - 89.6)	81.3 (67.5 - 89.6)
Neurological disorders	Motor neuron disease	Male	25-34	122	NR	96.7 (91.3 - 98.7)	92.2 (85.5 - 95.9)	88.4 (80.9 - 93.1)	78.3 (68.7 - 85.2)	72.3 (61.6 - 80.5)	64.4 (52.1 - 74.3)
Neurological disorders	Motor neuron disease	Male	35-44	242	12.62	92.5 (88.3 - 95.2)	75.0 (68.9 - 80.1)	63.0 (56.4 - 69.0)	55.3 (48.4 - 61.7)	47.6 (40.3 - 54.5)	46.4 (38.9 - 53.5)
Neurological disorders	Motor neuron disease	Male	45-54	589	5.04 (6.69 - 4.12)	88.6 (85.7 - 90.9)	61.8 (57.6 - 65.8)	50.0 (45.7 - 54.2)	41.0 (36.7 - 45.4)	37.3 (32.8 - 41.7)	30.8 (25.8 - 35.9)
Neurological disorders	Motor neuron disease	Male	55-64	1340	2.21 (2.5 - 1.99)	75.2 (72.7 - 77.4)	42.6 (39.8 - 45.3)	32.7 (30.0 - 35.3)	24.1 (21.6 - 26.6)	18.3 (16.0 - 20.8)	13.5 (11.1 - 16.2)
Neurological disorders	Motor neuron disease	Male	65-74	1899	1.43 (1.56 - 1.33)	60.2 (57.9 - 62.4)	31.6 (29.5 - 33.8)	23.2 (21.2 - 25.2)	14.1 (12.4 - 15.9)	7.3 (5.9 - 9.0)	3.9 (2.6 - 5.5)
Neurological disorders	Motor neuron disease	Male	75-84	1396	0.87 (0.98 - 0.77)	46.4 (43.8 - 49.1)	22.9 (20.6 - 25.3)	16.0 (13.9 - 18.1)	6.5 (5.1 - 8.2)	1.9 (1.0 - 3.2)	0.4 (0.1 - 1.4)
Neurological disorders	Motor neuron disease	Male	85-95	264	0.45 (0.61 - 0.35)	31.3 (25.7 - 37.0)	14.5 (10.4 - 19.2)	7.2 (4.4 - 11.1)	0.0 (0.0 - 0.0)	0.0 (0.0 - 0.0)	0.0 (0.0 - 0.0)
Neurological disorders	Multiple sclerosis	Male	All	8809	NR	98.0 (97.6 - 98.2)	95.7 (95.3 - 96.1)	93.4 (92.9 - 94.0)	86.5 (85.7 - 87.3)	77.6 (76.5 - 78.7)	67.2 (65.8 - 68.7)
Neurological disorders	Multiple sclerosis	Male	16-24	646	NR	99.8 (98.9 - 100.0)	99.8 (98.9 - 100.0)	99.7 (98.6 - 99.9)	98.9 (97.3 - 99.5)	97.6 (95.1 - 98.9)	97.0 (94.0 - 98.5)
Neurological disorders	Multiple sclerosis	Male	25-34	1834	NR	99.8 (99.5 - 99.9)	99.5 (99.1 - 99.8)	99.3 (98.8 - 99.6)	98.7 (97.9 - 99.1)	97.1 (95.8 - 98.0)	94.2 (92.1 - 95.8)
Neurological disorders	Multiple sclerosis	Male	35-44	1928	NR	100.0 (100.0 - 100.0)	99.6 (99.1 - 99.8)	98.8 (98.2 - 99.2)	96.7 (95.7 - 97.5)	92.9 (91.3 - 94.2)	87.6 (85.2 - 89.7)
Neurological disorders	Multiple sclerosis	Male	45-54	1861	NR	98.6 (97.9 - 99.0)	97.9 (97.1 - 98.5)	96.4 (95.4 - 97.2)	91.2 (89.7 - 92.5)	83.4 (81.3 - 85.3)	71.1 (68.1 - 73.8)
Neurological disorders	Multiple sclerosis	Male	55-64	1491	19.42 (20.16 - 17.83)	97.6 (96.7 - 98.3)	94.8 (93.5 - 95.8)	91.6 (90.0 - 92.9)	80.7 (78.4 - 82.8)	64.6 (61.6 - 67.4)	47.2 (43.6 - 50.7)
Neurological disorders	Multiple sclerosis	Male	65-74	721	10.93 (11.62 - 9.86)	94.0 (92.0 - 95.5)	84.8 (81.9 - 87.3)	77.8 (74.5 - 80.8)	53.4 (49.2 - 57.3)	28.5 (24.4 - 32.8)	10.8 (7.5 - 14.7)
Neurological disorders	Multiple sclerosis	Male	75-84	281	5.0 (6.24 - 4.28)	81.0 (75.8 - 85.1)	64.0 (58.0 - 69.4)	49.6 (43.5 - 55.4)	22.6 (17.4 - 28.2)	8.8 (5.3 - 13.3)	1.3 (0.1 - 5.4)
Neurological disorders	Multiple sclerosis	Male	85-95	47	1.78 (3.49 - 1.09)	65.8 (50.4 - 77.5)	39.7 (25.3 - 53.7)	20.2 (9.3 - 34.0)	0.0 (0.0 - 0.0)	0.0 (0.0 - 0.0)	0.0 (0.0 - 0.0)
Neurological disorders	Parkinson's disease	Male	All	39160	6.45 (6.54 - 6.34)	88.9 (88.6 - 89.2)	74.0 (73.5 - 74.4)	59.3 (58.8 - 59.8)	31.4 (30.8 - 31.9)	15.6 (15.1 - 16.1)	7.4 (6.9 - 7.9)
Neurological disorders	Parkinson's disease	Male	16-24	21	NR	100.0 (100.0 - 100.0)	100.0 (100.0 - 100.0)	100.0 (100.0 - 100.0)	77.9 (35.4 - 94.2)	77.9 (35.4 - 94.2)	77.9 (35.4 - 94.2)
Neurological disorders	Parkinson's disease	Male	25-34	81	NR	100.0 (100.0 - 100.0)	100.0 (100.0 - 100.0)	100.0 (100.0 - 100.0)	100.0 (100.0 - 100.0)	100.0 (100.0 - 100.0)	96.2 (75.7 - 99.4)
Neurological disorders	Parkinson's disease	Male	35-44	381	NR	99.7 (98.1 - 100.0)	98.9 (97.1 - 99.6)	98.9 (97.1 - 99.6)	94.5 (91.2 - 96.6)	89.6 (85.0 - 92.9)	86.6 (81.0 - 90.7)
Neurological disorders	Parkinson's disease	Male	45-54	1589	20.38 (21.1 - 19.38)	99.4 (98.9 - 99.7)	98.3 (97.5 - 98.8)	96.4 (95.3 - 97.3)	87.6 (85.5 - 89.4)	75.3 (72.3 - 78.1)	52.5 (48.1 - 56.8)
Neurological disorders	Parkinson's disease	Male	55-64	5401	13.91 (14.23 - 13.66)	98.1 (97.7 - 98.4)	94.6 (93.9 - 95.1)	89.3 (88.4 - 90.2)	71.5 (70.1 - 72.9)	43.7 (41.9 - 45.6)	18.4 (16.5 - 20.3)
Neurological disorders	Parkinson's disease	Male	65-74	13005	8.22 (8.37 - 8.07)	94.6 (94.2 - 95.0)	85.0 (84.3 - 85.6)	72.4 (71.6 - 73.2)	38.0 (37.0 - 39.1)	12.3 (11.5 - 13.2)	2.4 (1.8 - 3.0)
Neurological disorders	Parkinson's disease	Male	75-84	14856	4.24 (4.33 - 4.17)	85.0 (84.4 - 85.6)	63.5 (62.7 - 64.4)	42.4 (41.6 - 43.3)	8.9 (8.4 - 9.5)	1.0 (0.7 - 1.3)	0.2 (0.1 - 0.4)
Neurological disorders	Parkinson's disease	Male	85-95	3786	1.89 (2.0 - 1.82)	66.1 (64.6 - 67.6)	33.4 (31.8 - 35.0)	14.5 (13.3 - 15.7)	0.7 (0.5 - 1.2)	0.0 (0.0 - 0.0)	0.0 (0.0 - 0.0)
Neurological disorders	Unspecified dementia	Male	All	90859	1.56 (1.59 - 1.54)	59.3 (59.0 - 59.6)	31.9 (31.6 - 32.2)	16.6 (16.3 - 16.9)	3.9 (3.7 - 4.0)	1.4 (1.3 - 1.5)	0.7 (0.6 - 0.8)
Neurological disorders	Unspecified dementia	Male	16-24	16	NR	100.0 (100.0 - 100.0)	100.0 (100.0 - 100.0)	100.0 (100.0 - 100.0)	100.0 (100.0 - 100.0)	100.0 (100.0 - 100.0)	100.0 (100.0 - 100.0)
Neurological disorders	Unspecified dementia	Male	25-34	26	22.36	96.2 (75.7 - 99.4)	92.3 (72.6 - 98.0)	92.3 (72.6 - 98.0)	82.0 (58.2 - 93.0)	82.0 (58.2 - 93.0)	61.5 (18.8 - 86.8)
Neurological disorders	Unspecified dementia	Male	35-44	74	NR	95.9 (87.8 - 98.7)	91.5 (82.1 - 96.1)	85.4 (74.5 - 91.9)	75.8 (62.7 - 84.8)	61.1 (45.9 - 73.3)	55.6 (38.0 - 70.0)
Neurological disorders	Unspecified dementia	Male	45-54	515	10.28 (12.83 - 8.45)	86.4 (83.2 - 89.1)	76.4 (72.4 - 79.9)	67.4 (63.0 - 71.4)	51.1 (46.3 - 55.7)	39.8 (34.7 - 44.9)	32.2 (26.5 - 38.0)
Neurological disorders	Unspecified dementia	Male	55-64	2540	6.23 (6.68 - 5.88)	86.6 (85.2 - 87.8)	72.5 (70.7 - 74.2)	58.5 (56.5 - 60.5)	30.8 (28.8 - 32.9)	16.5 (14.6 - 18.5)	8.6 (6.7 - 10.8)
Neurological disorders	Unspecified dementia	Male	65-74	12122	3.56 (3.65 - 3.47)	77.7 (76.9 - 78.4)	55.6 (54.7 - 56.5)	36.4 (35.5 - 37.3)	10.4 (9.8 - 11.1)	2.7 (2.3 - 3.2)	0.7 (0.4 - 1.1)
Neurological disorders	Unspecified dementia	Male	75-84	39647	1.84 (1.87 - 1.8)	63.6 (63.1 - 64.1)	34.9 (34.4 - 35.4)	17.1 (16.7 - 17.5)	2.4 (2.2 - 2.6)	0.3 (0.2 - 0.4)	0.1 (0.0 - 0.1)

Appendix Table A6: Unadjusted Median Survival and Survival Probabilities by Age, males (Page 17 of 18)

Group	Diagnosis Category	Sex	Age	N	Median Survival (y)	1y (%)	3y (%)	5y (%)	10y (%)	15y (%)	20y (%)
Neurological disorders	Unspecified dementia	Male	85-95	34689	0.85 (0.87 - 0.83)	46.6 (46.0 - 47.1)	17.1 (16.7 - 17.5)	5.4 (5.1 - 5.6)	0.2 (0.2 - 0.3)	0.0 (0.0 - 0.1)	0.0 (0.0 - 0.1)
Neurological disorders	Vascular dementia	Male	All	38164	2.28 (2.32 - 2.25)	69.1 (68.6 - 69.5)	41.3 (40.8 - 41.8)	23.9 (23.4 - 24.3)	6.3 (6.0 - 6.6)	2.3 (2.1 - 2.5)	1.2 (1.0 - 1.4)
Neurological disorders	Vascular dementia	Male	16-24	13	NR	100.0 (100.0 - 100.0)	92.3 (56.6 - 98.9)	92.3 (56.6 - 98.9)	84.6 (51.2 - 95.9)	84.6 (51.2 - 95.9)	84.6 (51.2 - 95.9)
Neurological disorders	Vascular dementia	Male	25-34	31	NR	96.8 (79.2 - 99.5)	93.5 (76.6 - 98.3)	90.3 (72.9 - 96.8)	90.3 (72.9 - 96.8)	82.8 (57.0 - 93.9)	82.8 (57.0 - 93.9)
Neurological disorders	Vascular dementia	Male	35-44	56	NR	100.0 (100.0 - 100.0)	98.1 (87.1 - 99.7)	94.0 (82.6 - 98.0)	83.0 (68.8 - 91.1)	71.3 (54.3 - 82.9)	63.9 (45.7 - 77.4)
Neurological disorders	Vascular dementia	Male	45-54	239	12.07 (15.4 - 9.81)	90.7 (86.3 - 93.8)	80.0 (74.3 - 84.6)	72.5 (66.1 - 77.8)	56.8 (49.6 - 63.4)	44.0 (36.3 - 51.5)	33.2 (24.6 - 42.1)
Neurological disorders	Vascular dementia	Male	55-64	1353	7.52 (8.12 - 7.04)	89.9 (88.2 - 91.4)	77.6 (75.2 - 79.8)	64.0 (61.2 - 66.6)	37.7 (34.8 - 40.7)	20.7 (17.9 - 23.6)	12.5 (9.6 - 15.8)
Neurological disorders	Vascular dementia	Male	65-74	6758	4.16 (4.28 - 4.02)	82.9 (82.0 - 83.8)	61.3 (60.1 - 62.5)	42.5 (41.3 - 43.8)	14.0 (13.0 - 15.0)	3.6 (3.0 - 4.2)	0.9 (0.6 - 1.5)
Neurological disorders	Vascular dementia	Male	75-84	18510	2.39 (2.46 - 2.34)	71.4 (70.7 - 72.0)	42.4 (41.7 - 43.2)	22.6 (22.0 - 23.3)	3.2 (2.9 - 3.6)	0.5 (0.4 - 0.7)	0.1 (0.0 - 0.3)
Neurological disorders	Vascular dementia	Male	85-95	10987	1.19 (1.23 - 1.14)	54.0 (53.0 - 54.9)	21.5 (20.7 - 22.4)	7.6 (7.0 - 8.1)	0.3 (0.2 - 0.5)	0.1 (0.0 - 0.2)	0.1 (0.0 - 0.2)
Other	Decubitus ulcer	Male	All	36588	1.93 (1.99 - 1.87)	59.6 (59.1 - 60.1)	42.0 (41.5 - 42.5)	31.2 (30.7 - 31.8)	17.9 (17.4 - 18.4)	11.9 (11.4 - 12.4)	8.7 (8.2 - 9.3)
Other	Decubitus ulcer	Male	16-24	470	NR	96.3 (94.2 - 97.7)	91.6 (88.5 - 93.8)	88.7 (85.2 - 91.3)	86.6 (82.8 - 89.6)	83.6 (79.0 - 87.2)	78.2 (71.6 - 83.4)
Other	Decubitus ulcer	Male	25-34	668	NR	94.3 (92.2 - 95.9)	89.0 (86.3 - 91.2)	85.8 (82.7 - 88.4)	80.0 (76.2 - 83.2)	75.0 (70.6 - 78.9)	68.8 (63.2 - 73.7)
Other	Decubitus ulcer	Male	35-44	934	NR	91.3 (89.3 - 92.9)	86.5 (84.1 - 88.6)	83.3 (80.6 - 85.6)	76.7 (73.5 - 79.6)	65.0 (60.9 - 68.9)	55.1 (49.3 - 60.4)
Other	Decubitus ulcer	Male	45-54	1768	11.67 (12.33 - 10.8)	84.1 (82.3 - 85.7)	77.2 (75.1 - 79.1)	70.3 (68.0 - 72.5)	55.3 (52.6 - 58.0)	40.8 (37.6 - 44.0)	30.0 (26.0 - 34.1)
Other	Decubitus ulcer	Male	55-64	4137	5.93 (6.44 - 5.59)	76.7 (75.4 - 78.0)	63.9 (62.4 - 65.4)	54.7 (53.0 - 56.3)	35.4 (33.6 - 37.2)	21.8 (20.0 - 23.6)	12.5 (10.4 - 14.7)
Other	Decubitus ulcer	Male	65-74	8659	2.85 (3.02 - 2.7)	65.8 (64.7 - 66.8)	49.0 (47.9 - 50.1)	36.8 (35.7 - 38.0)	17.2 (16.1 - 18.2)	7.5 (6.6 - 8.5)	2.3 (1.4 - 3.6)
Other	Decubitus ulcer	Male	75-84	11660	1.27 (1.35 - 1.21)	53.9 (52.9 - 54.8)	32.6 (31.7 - 33.6)	19.2 (18.4 - 20.0)	4.9 (4.3 - 5.5)	0.9 (0.6 - 1.3)	0.2 (0.1 - 0.5)
Other	Decubitus ulcer	Male	85-95	7870	0.51 (0.55 - 0.47)	39.2 (38.1 - 40.3)	17.0 (16.1 - 17.9)	6.9 (6.3 - 7.6)	0.5 (0.3 - 0.8)	0.1 (0.0 - 0.4)	0.1 (0.0 - 0.4)
Other	Endo/metab/blood/immune	Male	All	677544	17.34 (17.42 - 17.26)	92.3 (92.2 - 92.4)	85.9 (85.8 - 86.0)	80.5 (80.4 - 80.6)	67.6 (67.5 - 67.8)	55.4 (55.3 - 55.6)	44.2 (44.0 - 44.4)
Other	Endo/metab/blood/immune	Male	16-24	21392	NR	99.4 (99.3 - 99.5)	98.7 (98.6 - 98.9)	98.3 (98.2 - 98.5)	97.4 (97.2 - 97.7)	96.3 (96.0 - 96.6)	95.0 (94.5 - 95.5)
Other	Endo/metab/blood/immune	Male	25-34	31928	NR	99.2 (99.1 - 99.3)	98.4 (98.3 - 98.5)	97.7 (97.6 - 97.9)	96.3 (96.0 - 96.5)	94.5 (94.1 - 94.8)	92.1 (91.6 - 92.6)
Other	Endo/metab/blood/immune	Male	35-44	55509	NR	98.8 (98.7 - 98.9)	97.8 (97.6 - 97.9)	96.9 (96.8 - 97.1)	94.4 (94.2 - 94.6)	90.9 (90.5 - 91.2)	86.0 (85.5 - 86.5)
Other	Endo/metab/blood/immune	Male	45-54	96157	NR	97.9 (97.8 - 97.9)	95.9 (95.8 - 96.1)	94.3 (94.2 - 94.5)	89.5 (89.3 - 89.8)	83.0 (82.7 - 83.3)	73.7 (73.2 - 74.2)
Other	Endo/metab/blood/immune	Male	55-64	150999	20.89 (21.02 - 20.7)	95.9 (95.8 - 96.0)	92.4 (92.3 - 92.6)	89.2 (89.1 - 89.4)	80.1 (79.8 - 80.3)	68.2 (67.9 - 68.5)	52.7 (52.3 - 53.2)
Other	Endo/metab/blood/immune	Male	65-74	170873	12.85 (12.92 - 12.78)	92.0 (91.9 - 92.2)	85.4 (85.2 - 85.5)	79.2 (79.0 - 79.4)	61.6 (61.4 - 61.9)	40.6 (40.2 - 40.9)	19.9 (19.4 - 20.3)
Other	Endo/metab/blood/immune	Male	75-84	117106	6.29 (6.34 - 6.24)	84.5 (84.3 - 84.7)	70.8 (70.5 - 71.0)	58.1 (57.8 - 58.4)	29.1 (28.7 - 29.4)	9.7 (9.4 - 10.0)	1.7 (1.5 - 1.9)
Other	Endo/metab/blood/immune	Male	85-95	32742	2.23 (2.28 - 2.18)	66.6 (66.1 - 67.2)	41.6 (41.0 - 42.2)	23.9 (23.4 - 24.4)	4.2 (3.9 - 4.5)	0.3 (0.2 - 0.4)	0.1 (0.0 - 0.2)
Other	HIV/AIDS	Male	All	23165	NR	96.4 (96.1 - 96.6)	93.2 (92.8 - 93.5)	90.7 (90.3 - 91.1)	86.2 (85.7 - 86.7)	82.1 (81.5 - 82.6)	76.7 (75.9 - 77.5)
Other	HIV/AIDS	Male	16-24	3845	NR	99.6 (99.4 - 99.8)	99.0 (98.6 - 99.3)	98.5 (98.1 - 98.9)	97.8 (97.2 - 98.2)	96.9 (96.1 - 97.5)	95.5 (94.4 - 96.4)
Other	HIV/AIDS	Male	25-34	5945	NR	99.6 (99.4 - 99.7)	99.2 (99.0 - 99.4)	98.8 (98.5 - 99.1)	97.6 (97.2 - 98.0)	96.8 (96.2 - 97.3)	95.1 (94.2 - 95.9)
Other	HIV/AIDS	Male	35-44	4496	NR	98.9 (98.6 - 99.2)	97.7 (97.2 - 98.1)	96.9 (96.4 - 97.4)	94.9 (94.2 - 95.6)	92.7 (91.8 - 93.5)	88.9 (87.4 - 90.1)
Other	HIV/AIDS	Male	45-54	3224	NR	97.9 (97.3 - 98.3)	95.1 (94.3 - 95.8)	93.0 (92.0 - 93.8)	88.3 (87.0 - 89.4)	82.1 (80.5 - 83.6)	73.1 (70.6 - 75.5)
Other	HIV/AIDS	Male	55-64	2426	20.2 (21.7 - 19.17)	94.0 (93.0 - 94.9)	88.5 (87.1 - 89.7)	83.6 (82.0 - 85.1)	73.2 (71.2 - 75.1)	63.2 (60.8 - 65.5)	50.8 (47.6 - 53.9)
Other	HIV/AIDS	Male	65-74	1971	10.39 (11.59 - 9.62)	88.4 (86.9 - 89.8)	77.7 (75.7 - 79.5)	68.2 (65.9 - 70.4)	51.7 (49.0 - 54.3)	35.1 (31.9 - 38.3)	19.6 (15.8 - 23.6)
Other	HIV/AIDS	Male	75-84	1025	4.38 (4.8 - 3.87)	76.0 (73.2 - 78.5)	59.3 (56.1 - 62.4)	45.6 (42.2 - 48.9)	22.4 (19.2 - 25.8)	8.2 (5.7 - 11.2)	1.4 (0.2 - 5.6)
Other	HIV/AIDS	Male	85-95	227	1.97 (2.39 - 1.53)	66.8 (60.2 - 72.6)	39.2 (32.5 - 45.7)	23.6 (17.8 - 29.9)	4.6 (2.0 - 8.9)	0.0 (0.0 - 0.0)	0.0 (0.0 - 0.0)
Other	Hemoglobinopathies	Male	All	281656	3.78 (3.82 - 3.75)	71.2 (71.0 - 71.3)	54.5 (54.3 - 54.7)	44.2 (44.0 - 44.4)	28.8 (28.6 - 29.0)	20.3 (20.0 - 20.5)	15.2 (15.0 - 15.5)
Other	Hemoglobinopathies	Male	16-24	4303	NR	97.0 (96.5 - 97.5)	94.6 (93.8 - 95.2)	93.6 (92.8 - 94.3)	91.9 (91.0 - 92.8)	90.6 (89.5 - 91.6)	89.4 (87.9 - 90.7)
Other	Hemoglobinopathies	Male	25-34	5750	NR	95.5 (94.9 - 96.0)	92.7 (91.9 - 93.3)	91.0 (90.2 - 91.7)	87.7 (86.7 - 88.6)	85.4 (84.2 - 86.6)	83.0 (81.4 - 84.5)
Other	Hemoglobinopathies	Male	35-44	8721	NR	92.0 (91.4 - 92.6)	87.5 (86.7 - 88.2)	84.9 (84.1 - 85.6)	79.7 (78.7 - 80.6)	75.3 (74.2 - 76.4)	70.1 (68.5 - 71.6)
Other	Hemoglobinopathies	Male	45-54	16813	18.94 (19.8 - 17.9)	85.8 (85.3 - 86.3)	78.8 (78.1 - 79.4)	74.1 (73.4 - 74.8)	64.6 (63.8 - 65.4)	55.9 (54.9 - 56.8)	48.6 (47.3 - 49.9)
Other	Hemoglobinopathies	Male	55-64	37813	9.82 (10.05 - 9.57)	79.9 (79.5 - 80.3)	69.5 (69.0 - 70.0)	62.7 (62.2 - 63.2)	49.6 (49.0 - 50.1)	38.2 (37.5 - 38.9)	28.1 (27.3 - 29.0)
Other	Hemoglobinopathies	Male	65-74	69562	5.21 (5.29 - 5.11)	74.3 (74.0 - 74.7)	60.3 (60.0 - 60.7)	50.8 (50.4 - 51.2)	32.7 (32.2 - 33.1)	18.5 (18.1 - 19.0)	8.4 (7.8 - 8.9)
Other	Hemoglobinopathies	Male	75-84	88112	2.65 (2.7 - 2.61)	67.5 (67.2 - 67.8)	47.2 (46.8 - 47.5)	33.4 (33.1 - 33.7)	12.2 (11.9 - 12.5)	2.9 (2.7 - 3.1)	0.5 (0.4 - 0.6)
Other	Hemoglobinopathies	Male	85-95	48578	1.2 (1.23 - 1.17)	53.9 (53.4 - 54.3)	26.8 (26.4 - 27.2)	12.9 (12.5 - 13.2)	1.4 (1.3 - 1.5)	0.1 (0.1 - 0.2)	0.0 (0.0 - 0.1)
Other	Psoriasis	Male	All	110597	NR	98.1 (98.0 - 98.1)	94.9 (94.8 - 95.0)	91.8 (91.6 - 91.9)	83.6 (83.3 - 83.8)	75.1 (74.8 - 75.4)	66.6 (66.1 - 67.0)
Other	Psoriasis	Male	16-24	7498	NR	100.0 (99.9 - 100.0)	99.8 (99.7 - 99.9)	99.7 (99.5 - 99.8)	99.1 (98.9 - 99.4)	98.6 (98.2 - 98.9)	97.9 (97.4 - 98.4)
Other	Psoriasis	Male	25-34	13493	NR	99.9 (99.9 - 100.0)	99.7 (99.6 - 99.8)	99.6 (99.5 - 99.7)	98.9 (98.7 - 99.1)	98.3 (98.0 - 98.6)	97.3 (96.8 - 97.7)
Other	Psoriasis	Male	35-44	17409	NR	99.8 (99.7 - 99.9)	99.4 (99.3 - 99.5)	99.0 (98.8 - 99.1)	97.6 (97.3 - 97.8)	95.6 (95.2 - 96.0)	92.7 (92.1 - 93.3)

Appendix Table A6: Unadjusted Median Survival and Survival Probabilities by Age, males (Page 18 of 18)

Group	Diagnosis Category	Sex	Age	N	Median Survival (y)	1y (%)	3y (%)	5y (%)	10y (%)	15y (%)	20y (%)
Other	Psoriasis	Male	45-54	19979	NR	99.5 (99.4 - 99.6)	98.5 (98.3 - 98.7)	97.4 (97.2 - 97.6)	94.2 (93.8 - 94.5)	89.2 (88.6 - 89.7)	82.3 (81.5 - 83.1)
Other	Psoriasis	Male	55-64	22790	NR	98.7 (98.5 - 98.8)	96.2 (95.9 - 96.4)	93.5 (93.1 - 93.8)	85.5 (85.0 - 86.0)	74.7 (74.0 - 75.4)	59.8 (58.7 - 60.8)
Other	Psoriasis	Male	65-74	18495	14.21 (14.42 - 14.04)	97.0 (96.7 - 97.2)	91.4 (91.0 - 91.8)	85.8 (85.3 - 86.4)	68.5 (67.7 - 69.3)	46.3 (45.2 - 47.3)	24.2 (22.9 - 25.5)
Other	Psoriasis	Male	75-84	9024	7.49 (7.7 - 7.28)	91.8 (91.2 - 92.4)	79.5 (78.7 - 80.4)	66.2 (65.1 - 67.2)	34.0 (32.8 - 35.2)	11.9 (10.9 - 13.0)	2.1 (1.4 - 2.9)
Other	Psoriasis	Male	85-95	1870	3.26 (3.46 - 3.03)	78.8 (76.9 - 80.6)	52.7 (50.3 - 55.0)	33.3 (31.0 - 35.6)	6.0 (4.7 - 7.5)	0.0 (0.0 - 0.0)	0.0 (0.0 - 0.0)
Other	Tuberculosis	Male	All	18337	NR	93.1 (92.7 - 93.5)	87.9 (87.4 - 88.4)	83.7 (83.1 - 84.2)	75.1 (74.4 - 75.8)	68.0 (67.1 - 68.8)	61.2 (60.1 - 62.3)
Other	Tuberculosis	Male	16-24	2113	NR	99.9 (99.6 - 100.0)	99.7 (99.3 - 99.8)	99.4 (98.9 - 99.7)	98.6 (97.9 - 99.1)	98.1 (97.3 - 98.7)	97.6 (96.3 - 98.4)
Other	Tuberculosis	Male	25-34	3057	NR	99.6 (99.3 - 99.8)	99.3 (99.0 - 99.6)	99.2 (98.8 - 99.5)	98.5 (98.0 - 98.9)	97.7 (96.9 - 98.3)	96.4 (95.1 - 97.4)
Other	Tuberculosis	Male	35-44	3047	NR	99.5 (99.2 - 99.7)	99.0 (98.5 - 99.3)	98.2 (97.7 - 98.7)	96.7 (95.9 - 97.3)	94.3 (93.2 - 95.3)	91.1 (89.3 - 92.7)
Other	Tuberculosis	Male	45-54	2671	NR	97.8 (97.2 - 98.3)	96.0 (95.1 - 96.7)	94.0 (93.0 - 94.9)	90.1 (88.8 - 91.3)	85.5 (83.7 - 87.0)	77.5 (74.6 - 80.2)
Other	Tuberculosis	Male	55-64	2452	20.7 (21.47 - 19.07)	95.2 (94.2 - 95.9)	90.7 (89.5 - 91.8)	86.1 (84.6 - 87.5)	74.9 (73.0 - 76.7)	63.8 (61.4 - 66.1)	50.7 (47.3 - 54.1)
Other	Tuberculosis	Male	65-74	2164	10.38 (11.09 - 9.68)	88.6 (87.2 - 89.9)	79.6 (77.8 - 81.2)	71.7 (69.7 - 73.5)	51.4 (49.0 - 53.7)	32.6 (30.0 - 35.2)	17.2 (14.4 - 20.2)
Other	Tuberculosis	Male	75-84	2059	4.08 (4.45 - 3.85)	77.4 (75.5 - 79.1)	58.9 (56.7 - 61.0)	44.3 (42.0 - 46.5)	18.6 (16.8 - 20.6)	4.7 (3.6 - 6.0)	0.7 (0.3 - 1.6)
Other	Tuberculosis	Male	85-95	759	1.43 (1.69 - 1.18)	55.4 (51.8 - 58.9)	31.6 (28.3 - 35.0)	17.7 (15.0 - 20.5)	2.8 (1.7 - 4.4)	0.0 (0.0 - 0.0)	0.0 (0.0 - 0.0)
Other	Urinary tract infections and interstitial nephritis	Male	All	446658	8.11 (8.18 - 8.05)	82.8 (82.7 - 82.9)	69.0 (68.8 - 69.1)	59.7 (59.5 - 59.8)	45.8 (45.6 - 45.9)	37.5 (37.4 - 37.7)	31.7 (31.4 - 31.9)
Other	Urinary tract infections and interstitial nephritis	Male	16-24	20376	NR	99.6 (99.6 - 99.7)	99.4 (99.2 - 99.5)	99.1 (98.9 - 99.2)	98.5 (98.3 - 98.7)	98.0 (97.8 - 98.2)	97.5 (97.2 - 97.8)
Other	Urinary tract infections and interstitial nephritis	Male	25-34	27809	NR	99.6 (99.5 - 99.7)	99.1 (99.0 - 99.3)	98.8 (98.7 - 99.0)	98.1 (97.9 - 98.2)	97.3 (97.1 - 97.5)	96.2 (95.8 - 96.5)
Other	Urinary tract infections and interstitial nephritis	Male	35-44	20401	NR	98.8 (98.6 - 98.9)	97.7 (97.5 - 97.9)	96.8 (96.5 - 97.0)	94.6 (94.3 - 94.9)	91.8 (91.3 - 92.2)	88.0 (87.2 - 88.7)
Other	Urinary tract infections and interstitial nephritis	Male	45-54	27688	NR	96.7 (96.5 - 96.9)	93.8 (93.5 - 94.1)	91.4 (91.1 - 91.7)	85.7 (85.3 - 86.2)	79.1 (78.4 - 79.7)	71.5 (70.6 - 72.4)
Other	Urinary tract infections and interstitial nephritis	Male	55-64	53699	19.06 (19.33 - 18.77)	93.2 (93.0 - 93.4)	87.5 (87.2 - 87.8)	82.9 (82.5 - 83.2)	71.9 (71.5 - 72.4)	60.0 (59.5 - 60.6)	47.6 (46.8 - 48.4)
Other	Urinary tract infections and interstitial nephritis	Male	65-74	96491	9.51 (9.62 - 9.41)	87.1 (86.9 - 87.4)	76.2 (75.9 - 76.5)	67.4 (67.1 - 67.7)	48.1 (47.8 - 48.5)	30.2 (29.8 - 30.7)	15.0 (14.5 - 15.5)
Other	Urinary tract infections and interstitial nephritis	Male	75-84	125153	3.69 (3.73 - 3.65)	76.5 (76.3 - 76.8)	55.8 (55.5 - 56.1)	40.4 (40.1 - 40.7)	16.1 (15.9 - 16.4)	4.7 (4.5 - 4.9)	0.9 (0.8 - 1.0)
Other	Urinary tract infections and interstitial nephritis	Male	85-95	72066	1.58 (1.6 - 1.55)	60.5 (60.2 - 60.9)	31.2 (30.9 - 31.6)	15.4 (15.1 - 15.7)	2.0 (1.8 - 2.1)	0.2 (0.1 - 0.2)	0.0 (0.0 - 0.1)

Appendix Table A7: Unadjusted Median Survival and Survival Probabilities by Age, females (Page 1 of 18)

Group	Diagnosis Category	Sex	Age	N	Median Survival (y)	1y (%)	3y (%)	5y (%)	10y (%)	15y (%)	20y (%)
Cancers	Bladder cancer	Female	All	18444	7.73 (7.98 - 7.47)	79.6 (79.0 - 80.2)	66.2 (65.5 - 66.9)	58.8 (58.0 - 59.5)	43.2 (42.4 - 44.0)	30.4 (29.5 - 31.3)	21.3 (20.3 - 22.4)
Cancers	Bladder cancer	Female	16-24	27	NR	100.0 (100.0 - 100.0)	95.8 (73.9 - 99.4)	91.7 (70.6 - 97.8)	91.7 (70.6 - 97.8)	91.7 (70.6 - 97.8)	91.7 (70.6 - 97.8)
Cancers	Bladder cancer	Female	25-34	93	NR	98.9 (92.5 - 99.8)	96.6 (89.9 - 98.9)	95.4 (88.1 - 98.2)	92.5 (83.8 - 96.6)	92.5 (83.8 - 96.6)	92.5 (83.8 - 96.6)
Cancers	Bladder cancer	Female	35-44	253	NR	88.7 (84.1 - 92.1)	80.8 (75.3 - 85.3)	80.0 (74.3 - 84.5)	77.1 (71.0 - 82.0)	74.6 (68.1 - 80.0)	70.7 (62.1 - 77.7)
Cancers	Bladder cancer	Female	45-54	883	NR	91.2 (89.1 - 92.9)	83.3 (80.5 - 85.6)	82.0 (79.2 - 84.4)	78.3 (75.2 - 81.1)	74.1 (70.6 - 77.3)	70.3 (66.1 - 74.0)
Cancers	Bladder cancer	Female	55-64	2807	20.17 (21.01 - 19.0)	90.2 (89.1 - 91.3)	81.6 (80.1 - 83.0)	77.9 (76.2 - 79.4)	70.2 (68.3 - 72.0)	60.4 (58.1 - 62.6)	50.3 (47.4 - 53.2)
Cancers	Bladder cancer	Female	65-74	5272	11.87 (12.44 - 11.35)	86.2 (85.2 - 87.1)	75.4 (74.2 - 76.6)	69.5 (68.2 - 70.8)	55.8 (54.2 - 57.4)	38.3 (36.4 - 40.2)	21.1 (18.9 - 23.4)
Cancers	Bladder cancer	Female	75-84	6178	5.42 (5.74 - 5.15)	77.4 (76.4 - 78.5)	62.0 (60.7 - 63.2)	52.1 (50.7 - 53.4)	28.0 (26.7 - 29.4)	11.1 (9.9 - 12.3)	3.3 (2.5 - 4.3)
Cancers	Bladder cancer	Female	85-95	2824	1.52 (1.7 - 1.4)	57.8 (55.9 - 59.6)	36.3 (34.4 - 38.1)	24.3 (22.6 - 26.0)	6.4 (5.3 - 7.7)	1.2 (0.6 - 1.9)	0.0 (0.0 - 0.0)
Cancers	Brain cancer	Female	All	12920	1.12 (1.17 - 1.07)	52.2 (51.4 - 53.1)	35.3 (34.5 - 36.2)	30.3 (29.5 - 31.2)	23.9 (23.1 - 24.7)	20.0 (19.2 - 20.8)	16.9 (16.0 - 17.7)
Cancers	Brain cancer	Female	16-24	397	NR	93.9 (91.0 - 95.9)	86.7 (82.9 - 89.7)	82.5 (78.3 - 86.0)	77.8 (73.2 - 81.8)	73.0 (67.9 - 77.5)	72.4 (67.2 - 77.0)
Cancers	Brain cancer	Female	25-34	684	NR	92.3 (90.0 - 94.1)	83.9 (80.9 - 86.5)	78.4 (75.0 - 81.4)	68.3 (64.4 - 71.9)	63.7 (59.4 - 67.6)	59.7 (54.9 - 64.1)
Cancers	Brain cancer	Female	35-44	1009	16.2 (17.95 - 14.11)	86.3 (84.0 - 88.3)	72.1 (69.1 - 74.8)	66.0 (62.9 - 69.0)	58.2 (54.8 - 61.4)	51.9 (48.2 - 55.4)	44.4 (40.0 - 48.7)
Cancers	Brain cancer	Female	45-54	1648	3.02 (3.61 - 2.63)	72.0 (69.8 - 74.1)	50.2 (47.7 - 52.6)	43.6 (41.1 - 46.1)	35.3 (32.8 - 37.8)	31.0 (28.4 - 33.6)	26.2 (23.2 - 29.2)
Cancers	Brain cancer	Female	55-64	2597	1.4 (1.48 - 1.29)	59.5 (57.6 - 61.4)	33.7 (31.8 - 35.6)	27.9 (26.1 - 29.7)	21.4 (19.7 - 23.1)	17.5 (15.8 - 19.2)	13.4 (11.6 - 15.3)
Cancers	Brain cancer	Female	65-74	3166	0.76 (0.81 - 0.71)	42.5 (40.7 - 44.2)	23.5 (22.0 - 25.0)	18.7 (17.3 - 20.1)	12.3 (11.0 - 13.6)	8.2 (7.0 - 9.5)	4.1 (2.9 - 5.7)
Cancers	Brain cancer	Female	75-84	2522	0.35 (0.38 - 0.33)	25.5 (23.8 - 27.2)	15.4 (14.0 - 16.9)	11.6 (10.3 - 13.0)	5.2 (4.2 - 6.3)	1.8 (1.2 - 2.7)	0.7 (0.3 - 1.5)
Cancers	Brain cancer	Female	85-95	863	0.22 (0.25 - 0.2)	16.8 (14.4 - 19.4)	7.9 (6.1 - 9.9)	4.4 (3.0 - 6.0)	1.6 (0.8 - 2.8)	0.0 (0.0 - 0.0)	0.0 (0.0 - 0.0)
Cancers	Breast cancer	Female	All	186582	18.22 (18.38 - 18.06)	95.2 (95.1 - 95.3)	87.7 (87.5 - 87.8)	81.3 (81.1 - 81.5)	68.5 (68.3 - 68.8)	57.0 (56.7 - 57.3)	46.1 (45.8 - 46.5)
Cancers	Breast cancer	Female	16-24	209	NR	98.6 (95.6 - 99.5)	97.5 (94.2 - 99.0)	96.4 (92.6 - 98.3)	93.1 (88.0 - 96.0)	91.8 (85.9 - 95.3)	89.1 (79.9 - 94.2)
Cancers	Breast cancer	Female	25-34	2714	NR	98.3 (97.8 - 98.8)	91.7 (90.5 - 92.7)	87.1 (85.7 - 88.4)	80.9 (79.2 - 82.5)	78.1 (76.1 - 79.9)	73.7 (71.1 - 76.1)
Cancers	Breast cancer	Female	35-44	13707	NR	99.1 (98.9 - 99.3)	95.7 (95.3 - 96.0)	92.4 (92.0 - 92.9)	86.2 (85.5 - 86.8)	81.7 (80.8 - 82.5)	77.3 (76.2 - 78.4)
Cancers	Breast cancer	Female	45-54	33573	NR	98.8 (98.7 - 99.0)	95.8 (95.5 - 96.0)	93.0 (92.7 - 93.3)	87.4 (87.0 - 87.8)	81.7 (81.2 - 82.2)	75.1 (74.3 - 75.8)
Cancers	Breast cancer	Female	55-64	45674	NR	97.9 (97.8 - 98.0)	93.8 (93.6 - 94.0)	90.1 (89.8 - 90.4)	81.9 (81.5 - 82.3)	73.2 (72.7 - 73.7)	62.3 (61.6 - 63.0)
Cancers	Breast cancer	Female	65-74	49302	15.93 (16.09 - 15.78)	96.7 (96.6 - 96.9)	91.2 (90.9 - 91.5)	85.8 (85.5 - 86.1)	71.8 (71.3 - 72.3)	53.7 (53.1 - 54.4)	32.8 (32.0 - 33.7)
Cancers	Breast cancer	Female	75-84	27756	7.12 (7.24 - 7.0)	89.6 (89.3 - 90.0)	74.5 (73.9 - 75.0)	62.2 (61.6 - 62.8)	35.4 (34.7 - 36.1)	14.5 (13.9 - 15.1)	3.9 (3.5 - 4.4)
Cancers	Breast cancer	Female	85-95	13126	3.24 (3.32 - 3.16)	79.4 (78.7 - 80.1)	52.5 (51.6 - 53.4)	32.7 (31.9 - 33.6)	8.1 (7.5 - 8.6)	0.9 (0.7 - 1.2)	0.2 (0.0 - 0.4)
Cancers	Cervical cancer	Female	All	15585	19.21 (20.01 - 18.35)	86.4 (85.9 - 87.0)	74.9 (74.2 - 75.6)	69.4 (68.7 - 70.2)	61.6 (60.7 - 62.4)	55.7 (54.7 - 56.6)	48.9 (47.8 - 50.0)
Cancers	Cervical cancer	Female	16-24	163	NR	96.3 (91.9 - 98.3)	88.7 (82.7 - 92.8)	85.2 (78.5 - 89.9)	85.2 (78.5 - 89.9)	85.2 (78.5 - 89.9)	82.5 (73.5 - 88.7)
Cancers	Cervical cancer	Female	25-34	2014	NR	98.4 (97.7 - 98.8)	94.8 (93.7 - 95.7)	93.0 (91.7 - 94.1)	91.0 (89.5 - 92.3)	89.9 (88.2 - 91.3)	89.0 (87.1 - 90.6)
Cancers	Cervical cancer	Female	35-44	3105	NR	97.6 (97.0 - 98.1)	92.0 (91.0 - 92.9)	89.7 (88.6 - 90.8)	86.8 (85.4 - 88.0)	85.3 (83.8 - 86.6)	83.9 (82.2 - 85.5)
Cancers	Cervical cancer	Female	45-54	2699	NR	94.5 (93.6 - 95.3)	87.0 (85.6 - 88.2)	82.9 (81.3 - 84.3)	78.2 (76.5 - 79.8)	74.6 (72.6 - 76.4)	68.1 (65.7 - 70.4)
Cancers	Cervical cancer	Female	55-64	2464	16.09 (17.3 - 14.96)	88.3 (86.9 - 89.5)	75.8 (74.0 - 77.5)	69.8 (67.9 - 71.7)	60.7 (58.6 - 62.7)	52.2 (49.9 - 54.5)	44.2 (41.6 - 46.8)
Cancers	Cervical cancer	Female	65-74	2301	6.24 (7.08 - 5.64)	79.2 (77.5 - 80.8)	61.4 (59.3 - 63.4)	53.6 (51.5 - 55.7)	41.2 (39.0 - 43.5)	31.3 (29.0 - 33.7)	15.4 (13.0 - 18.0)
Cancers	Cervical cancer	Female	75-84	2080	2.39 (2.72 - 2.16)	66.3 (64.2 - 68.3)	46.0 (43.8 - 48.2)	35.1 (32.9 - 37.3)	17.8 (15.9 - 19.7)	7.1 (5.7 - 8.6)	1.1 (0.5 - 2.2)
Cancers	Cervical cancer	Female	85-95	737	0.98 (1.09 - 0.82)	49.3 (45.6 - 52.9)	24.5 (21.4 - 27.7)	13.4 (10.9 - 16.2)	3.3 (2.0 - 5.0)	0.2 (0.0 - 1.3)	0.0 (0.0 - 0.0)
Cancers	Colorectal cancer	Female	All	77291	5.87 (5.99 - 5.76)	76.9 (76.6 - 77.2)	61.3 (60.9 - 61.6)	52.9 (52.6 - 53.3)	38.9 (38.5 - 39.3)	28.3 (27.9 - 28.7)	19.4 (18.9 - 20.0)
Cancers	Colorectal cancer	Female	16-24	172	NR	95.9 (91.5 - 98.0)	90.9 (85.4 - 94.4)	88.2 (82.1 - 92.3)	87.5 (81.2 - 91.7)	87.5 (81.2 - 91.7)	87.5 (81.2 - 91.7)
Cancers	Colorectal cancer	Female	25-34	525	NR	91.0 (88.2 - 93.2)	80.8 (77.1 - 84.0)	76.9 (72.9 - 80.5)	74.5 (70.1 - 78.2)	72.4 (67.6 - 76.5)	69.6 (63.9 - 74.6)
Cancers	Colorectal cancer	Female	35-44	1622	NR	90.0 (88.4 - 91.4)	75.9 (73.6 - 77.9)	70.2 (67.7 - 72.4)	64.8 (62.2 - 67.3)	62.4 (59.7 - 65.1)	60.3 (57.0 - 63.4)
Cancers	Colorectal cancer	Female	45-54	4981	NR	89.0 (88.1 - 89.8)	76.2 (75.0 - 77.5)	70.3 (68.9 - 71.6)	64.3 (62.8 - 65.7)	59.9 (58.2 - 61.5)	56.0 (54.0 - 58.0)
Cancers	Colorectal cancer	Female	55-64	11646	16.63 (17.43 - 15.93)	87.0 (86.4 - 87.6)	73.5 (72.6 - 74.3)	67.3 (66.4 - 68.2)	59.2 (58.2 - 60.2)	52.7 (51.6 - 53.8)	44.7 (43.3 - 46.1)
Cancers	Colorectal cancer	Female	65-74	20998	9.79 (10.08 - 9.4)	82.3 (81.7 - 82.8)	68.5 (67.8 - 69.1)	61.4 (60.7 - 62.1)	49.4 (48.6 - 50.1)	37.0 (36.1 - 37.9)	21.1 (20.0 - 22.2)
Cancers	Colorectal cancer	Female	75-84	25134	4.53 (4.69 - 4.39)	74.2 (73.6 - 74.7)	57.5 (56.9 - 58.2)	47.9 (47.3 - 48.6)	27.9 (27.2 - 28.6)	11.9 (11.3 - 12.5)	2.7 (2.2 - 3.2)
Cancers	Colorectal cancer	Female	85-95	11857	1.48 (1.57 - 1.4)	57.1 (56.2 - 58.0)	36.1 (35.2 - 37.0)	24.2 (23.3 - 25.0)	6.9 (6.4 - 7.5)	1.2 (0.9 - 1.6)	0.0 (0.0 - 0.0)
Cancers	Esophageal cancer	Female	All	4232	0.7 (0.74 - 0.66)	40.3 (38.8 - 41.8)	22.0 (20.7 - 23.3)	16.6 (15.4 - 17.8)	11.9 (10.9 - 13.1)	8.7 (7.7 - 9.9)	4.4 (3.2 - 5.9)
Cancers	Esophageal cancer	Female	16-24	censored	censored	censored	censored	censored	censored	censored	censored
Cancers	Esophageal cancer	Female	25-34	16	NR	75.0 (46.3 - 89.8)	68.8 (40.5 - 85.6)	68.8 (40.5 - 85.6)	68.8 (40.5 - 85.6)	68.8 (40.5 - 85.6)	68.8 (40.5 - 85.6)
Cancers	Esophageal cancer	Female	35-44	54	5.23	66.5 (52.2 - 77.4)	57.0 (42.7 - 69.0)	52.9 (38.7 - 65.3)	46.4 (32.3 - 59.3)	40.0 (25.7 - 53.9)	40.0 (25.7 - 53.9)
Cancers	Esophageal cancer	Female	45-54	226	1.9 (3.04 - 1.3)	62.7 (56.0 - 68.6)	44.4 (37.8 - 50.8)	36.1 (29.7 - 42.6)	31.5 (25.1 - 38.1)	26.6 (19.8 - 33.8)	23.9 (16.3 - 32.3)

Appendix Table A7: Unadjusted Median Survival and Survival Probabilities by Age, females (Page 2 of 18)

Group	Diagnosis Category	Sex	Age	N	Median Survival (y)	1y (%)	3y (%)	5y (%)	10y (%)	15y (%)	20y (%)
Cancers	Esophageal cancer	Female	55-64	715	1.08 (1.22 - 0.91)	51.1 (47.3 - 54.7)	28.6 (25.2 - 32.1)	23.4 (20.2 - 26.7)	18.0 (15.0 - 21.3)	14.6 (11.6 - 17.9)	7.8 (4.3 - 12.6)
Cancers	Esophageal cancer	Female	65-74	1263	0.81 (0.9 - 0.74)	44.3 (41.6 - 47.1)	25.3 (22.8 - 27.8)	19.2 (17.0 - 21.6)	13.3 (11.1 - 15.6)	10.2 (8.0 - 12.8)	3.2 (1.4 - 6.3)
Cancers	Esophageal cancer	Female	75-84	1308	0.59 (0.66 - 0.56)	36.4 (33.7 - 39.0)	16.5 (14.4 - 18.7)	11.0 (9.3 - 13.0)	6.5 (5.1 - 8.2)	2.2 (1.2 - 3.7)	0.0 (0.0 - 0.0)
Cancers	Esophageal cancer	Female	85-95	621	0.34 (0.37 - 0.3)	18.0 (15.0 - 21.1)	6.6 (4.8 - 8.8)	3.1 (1.9 - 4.9)	0.0 (0.0 - 0.0)	0.0 (0.0 - 0.0)	0.0 (0.0 - 0.0)
Cancers	Gallbladder cancer	Female	All	9926	0.42 (0.44 - 0.4)	31.1 (30.2 - 32.1)	14.8 (14.0 - 15.5)	11.4 (10.8 - 12.1)	7.8 (7.2 - 8.4)	6.3 (5.7 - 6.9)	5.3 (4.7 - 6.0)
Cancers	Gallbladder cancer	Female	16-24	18	NR	82.6 (55.2 - 94.1)	64.9 (38.0 - 82.5)	64.9 (38.0 - 82.5)	58.4 (31.9 - 77.7)	58.4 (31.9 - 77.7)	58.4 (31.9 - 77.7)
Cancers	Gallbladder cancer	Female	25-34	43	7.6	67.4 (51.3 - 79.3)	58.1 (42.1 - 71.2)	50.9 (35.1 - 64.6)	48.2 (32.6 - 62.2)	45.2 (29.6 - 59.5)	45.2 (29.6 - 59.5)
Cancers	Gallbladder cancer	Female	35-44	128	1.55 (2.2 - 1.01)	59.7 (50.6 - 67.7)	36.3 (27.8 - 44.8)	34.0 (25.6 - 42.6)	32.8 (24.4 - 41.5)	32.8 (24.4 - 41.5)	29.2 (19.5 - 39.5)
Cancers	Gallbladder cancer	Female	45-54	489	0.94 (1.16 - 0.8)	48.6 (44.1 - 53.0)	28.3 (24.3 - 32.5)	24.4 (20.5 - 28.5)	21.8 (18.1 - 25.8)	20.9 (17.1 - 25.0)	20.2 (16.4 - 24.4)
Cancers	Gallbladder cancer	Female	55-64	1400	0.72 (0.82 - 0.65)	43.0 (40.3 - 45.6)	21.8 (19.6 - 24.1)	17.9 (15.8 - 20.1)	13.3 (11.4 - 15.3)	11.6 (9.8 - 13.6)	10.7 (8.8 - 12.8)
Cancers	Gallbladder cancer	Female	65-74	2927	0.56 (0.61 - 0.52)	36.4 (34.7 - 38.2)	16.5 (15.2 - 17.9)	12.5 (11.2 - 13.8)	8.3 (7.1 - 9.5)	6.1 (5.0 - 7.4)	3.8 (2.6 - 5.3)
Cancers	Gallbladder cancer	Female	75-84	3422	0.31 (0.34 - 0.29)	24.8 (23.3 - 26.3)	11.1 (10.0 - 12.3)	8.0 (7.0 - 9.0)	4.1 (3.3 - 5.0)	2.1 (1.4 - 2.9)	1.3 (0.7 - 2.2)
Cancers	Gallbladder cancer	Female	85-95	1453	0.16 (0.17 - 0.14)	14.4 (12.6 - 16.3)	4.5 (3.5 - 5.8)	2.6 (1.9 - 3.7)	0.4 (0.1 - 1.1)	0.0 (0.0 - 0.0)	0.0 (0.0 - 0.0)
Cancers	Hodgkin lymphoma	Female	All	3803	22.97	89.4 (88.3 - 90.3)	81.8 (80.5 - 83.0)	77.6 (76.2 - 79.0)	69.8 (68.2 - 71.4)	62.2 (60.4 - 64.0)	54.9 (52.8 - 57.0)
Cancers	Hodgkin lymphoma	Female	16-24	511	NR	99.6 (98.4 - 99.9)	99.0 (97.6 - 99.6)	98.5 (96.9 - 99.3)	97.7 (95.7 - 98.8)	96.8 (94.3 - 98.2)	96.2 (93.1 - 97.9)
Cancers	Hodgkin lymphoma	Female	25-34	623	NR	99.8 (98.9 - 100.0)	99.1 (98.0 - 99.6)	98.4 (97.0 - 99.2)	97.1 (95.2 - 98.3)	95.2 (92.6 - 96.9)	92.8 (89.2 - 95.2)
Cancers	Hodgkin lymphoma	Female	35-44	467	NR	99.3 (98.0 - 99.8)	97.5 (95.5 - 98.6)	96.7 (94.6 - 98.1)	92.9 (89.8 - 95.1)	88.9 (85.0 - 91.9)	83.8 (78.7 - 87.8)
Cancers	Hodgkin lymphoma	Female	45-54	379	NR	94.1 (91.2 - 96.1)	91.6 (88.3 - 94.0)	88.9 (85.2 - 91.7)	84.7 (80.4 - 88.2)	76.4 (70.9 - 81.0)	62.6 (55.3 - 69.1)
Cancers	Hodgkin lymphoma	Female	55-64	491	18.41 (22.68 - 16.9)	91.7 (88.9 - 93.8)	83.8 (80.2 - 86.9)	78.8 (74.7 - 82.2)	69.5 (64.9 - 73.7)	60.4 (55.2 - 65.2)	46.6 (40.4 - 52.6)
Cancers	Hodgkin lymphoma	Female	65-74	604	10.12 (11.47 - 9.15)	87.0 (84.0 - 89.4)	72.6 (68.7 - 76.0)	66.1 (62.0 - 69.9)	50.7 (46.1 - 55.1)	34.2 (29.3 - 39.2)	18.8 (13.8 - 24.4)
Cancers	Hodgkin lymphoma	Female	75-84	540	3.25 (3.99 - 2.29)	67.7 (63.6 - 71.5)	50.8 (46.4 - 55.1)	41.3 (36.9 - 45.6)	23.5 (19.4 - 27.8)	6.8 (4.1 - 10.4)	1.1 (0.1 - 4.9)
Cancers	Hodgkin lymphoma	Female	85-95	182	1.36 (1.94 - 0.99)	57.4 (49.8 - 64.3)	33.4 (26.4 - 40.6)	18.2 (12.5 - 24.6)	2.9 (0.8 - 7.4)	0.0 (0.0 - 0.0)	0.0 (0.0 - 0.0)
Cancers	Kidney cancer	Female	All	15306	5.68 (5.97 - 5.42)	73.6 (72.9 - 74.3)	59.6 (58.8 - 60.4)	52.2 (51.4 - 53.1)	38.1 (37.2 - 38.9)	27.4 (26.5 - 28.4)	18.9 (17.8 - 20.0)
Cancers	Kidney cancer	Female	16-24	29	NR	100.0 (100.0 - 100.0)	88.7 (69.0 - 96.2)	84.9 (64.5 - 94.0)	80.8 (59.9 - 91.6)	76.1 (54.0 - 88.6)	76.1 (54.0 - 88.6)
Cancers	Kidney cancer	Female	25-34	121	NR	90.8 (84.0 - 94.8)	85.2 (77.2 - 90.5)	83.2 (74.8 - 88.9)	82.0 (73.5 - 88.1)	78.5 (68.6 - 85.6)	78.5 (68.6 - 85.6)
Cancers	Kidney cancer	Female	35-44	365	NR	91.8 (88.5 - 94.3)	87.3 (83.3 - 90.4)	84.6 (80.3 - 88.1)	80.8 (75.9 - 84.8)	77.8 (72.4 - 82.4)	73.0 (65.6 - 79.1)
Cancers	Kidney cancer	Female	45-54	1105	NR	89.3 (87.3 - 91.0)	81.0 (78.4 - 83.2)	76.4 (73.6 - 78.9)	70.8 (67.7 - 73.6)	65.5 (61.9 - 68.8)	57.0 (51.9 - 61.8)
Cancers	Kidney cancer	Female	55-64	2576	13.63 (14.97 - 12.31)	83.9 (82.4 - 85.3)	72.6 (70.8 - 74.3)	67.6 (65.6 - 69.4)	57.9 (55.8 - 60.0)	47.4 (45.0 - 49.8)	36.5 (33.4 - 39.6)
Cancers	Kidney cancer	Female	65-74	4532	8.19 (8.67 - 7.61)	78.9 (77.7 - 80.1)	66.5 (65.0 - 67.9)	59.7 (58.2 - 61.2)	44.6 (42.9 - 46.3)	31.0 (29.1 - 32.9)	16.7 (14.4 - 19.1)
Cancers	Kidney cancer	Female	75-84	4784	3.23 (3.49 - 2.97)	67.4 (66.1 - 68.7)	51.3 (49.8 - 52.7)	41.2 (39.7 - 42.7)	21.1 (19.7 - 22.5)	7.6 (6.4 - 8.8)	1.7 (1.0 - 2.6)
Cancers	Kidney cancer	Female	85-95	1753	0.87 (0.96 - 0.76)	47.1 (44.7 - 49.4)	25.2 (23.1 - 27.3)	16.2 (14.4 - 18.1)	3.5 (2.6 - 4.7)	0.8 (0.4 - 1.6)	0.0 (0.0 - 0.0)
Cancers	Larynx cancer	Female	All	1219	6.86 (8.06 - 5.98)	79.5 (77.1 - 81.7)	66.3 (63.5 - 68.9)	56.6 (53.6 - 59.4)	39.6 (36.6 - 42.7)	29.3 (26.2 - 32.3)	20.2 (17.0 - 23.6)
Cancers	Larynx cancer	Female	16-24	5	NR	100.0 (100.0 - 100.0)	100.0 (100.0 - 100.0)	100.0 (100.0 - 100.0)	100.0 (100.0 - 100.0)	100.0 (100.0 - 100.0)	66.7 (5.4 - 94.5)
Cancers	Larynx cancer	Female	25-34	23	NR	91.1 (68.8 - 97.7)	91.1 (68.8 - 97.7)	91.1 (68.8 - 97.7)	91.1 (68.8 - 97.7)	91.1 (68.8 - 97.7)	91.1 (68.8 - 97.7)
Cancers	Larynx cancer	Female	35-44	44	NR	90.8 (77.3 - 96.4)	83.7 (68.8 - 91.9)	83.7 (68.8 - 91.9)	81.0 (65.5 - 90.0)	77.6 (61.0 - 87.8)	77.6 (61.0 - 87.8)
Cancers	Larynx cancer	Female	45-54	108	NR	90.7 (83.5 - 94.9)	84.2 (75.7 - 89.8)	81.2 (72.4 - 87.5)	69.9 (59.7 - 78.0)	66.6 (55.8 - 75.4)	57.9 (45.3 - 68.6)
Cancers	Larynx cancer	Female	55-64	287	10.0 (11.51 - 7.98)	83.9 (79.2 - 87.7)	72.6 (67.0 - 77.4)	63.7 (57.7 - 69.0)	49.6 (43.2 - 55.6)	39.3 (32.8 - 45.7)	23.9 (16.6 - 32.0)
Cancers	Larynx cancer	Female	65-74	405	6.97 (8.36 - 5.98)	85.2 (81.3 - 88.3)	68.0 (63.1 - 72.5)	58.0 (52.7 - 62.9)	35.6 (30.2 - 41.0)	20.0 (15.1 - 25.5)	9.0 (5.0 - 14.5)
Cancers	Larynx cancer	Female	75-84	264	3.15 (3.86 - 2.5)	66.1 (59.9 - 71.5)	52.0 (45.6 - 58.0)	36.3 (30.1 - 42.5)	17.7 (12.8 - 23.3)	5.9 (2.9 - 10.4)	2.2 (0.6 - 5.7)
Cancers	Larynx cancer	Female	85-95	81	1.61 (2.93 - 0.49)	52.8 (41.3 - 63.0)	39.3 (28.4 - 49.9)	26.3 (16.8 - 36.6)	4.3 (1.0 - 11.8)	0.0 (0.0 - 0.0)	0.0 (0.0 - 0.0)
Cancers	Leukemia	Female	All	18915	5.24 (5.46 - 5.04)	72.8 (72.2 - 73.4)	58.9 (58.2 - 59.7)	50.9 (50.1 - 51.6)	37.0 (36.2 - 37.8)	27.7 (26.8 - 28.5)	21.9 (21.0 - 22.8)
Cancers	Leukemia	Female	16-24	387	NR	94.3 (91.4 - 96.2)	88.9 (85.2 - 91.6)	85.6 (81.6 - 88.8)	82.7 (78.3 - 86.2)	81.8 (77.2 - 85.5)	81.0 (76.3 - 84.9)
Cancers	Leukemia	Female	25-34	498	NR	92.4 (89.7 - 94.5)	85.8 (82.3 - 88.6)	83.9 (80.3 - 86.9)	81.0 (77.0 - 84.4)	79.1 (74.8 - 82.7)	75.5 (70.4 - 79.8)
Cancers	Leukemia	Female	35-44	708	NR	90.4 (88.0 - 92.4)	81.7 (78.6 - 84.5)	77.5 (74.1 - 80.5)	72.8 (69.1 - 76.2)	69.8 (65.8 - 73.5)	65.7 (60.8 - 70.1)
Cancers	Leukemia	Female	45-54	1416	NR	90.1 (88.4 - 91.5)	82.0 (79.8 - 84.0)	78.8 (76.5 - 81.0)	72.6 (70.0 - 75.1)	66.1 (63.0 - 69.0)	60.4 (56.8 - 63.9)
Cancers	Leukemia	Female	55-64	2814	14.04 (15.14 - 13.13)	85.7 (84.3 - 86.9)	75.6 (73.9 - 77.2)	69.7 (67.9 - 71.4)	59.7 (57.7 - 61.7)	47.9 (45.6 - 50.2)	39.0 (36.3 - 41.8)
Cancers	Leukemia	Female	65-74	4750	7.29 (7.8 - 6.79)	78.7 (77.5 - 79.8)	65.1 (63.7 - 66.4)	57.1 (55.6 - 58.6)	42.2 (40.6 - 43.9)	27.2 (25.4 - 29.0)	14.9 (13.0 - 17.0)
Cancers	Leukemia	Female	75-84	5467	2.76 (2.95 - 2.54)	65.0 (63.7 - 66.3)	48.4 (47.0 - 49.8)	37.7 (36.4 - 39.1)	16.1 (15.0 - 17.4)	5.1 (4.3 - 6.0)	0.8 (0.3 - 1.5)
Cancers	Leukemia	Female	85-95	2757	0.84 (0.94 - 0.71)	47.1 (45.2 - 48.9)	27.1 (25.4 - 28.9)	15.7 (14.3 - 17.2)	2.5 (1.8 - 3.3)	0.1 (0.0 - 0.6)	0.1 (0.0 - 0.6)
Cancers	Lip oral cavity cancer	Female	All	14491	11.14 (11.59 - 10.8)	87.6 (87.1 - 88.1)	75.7 (75.0 - 76.4)	67.9 (67.1 - 68.7)	52.8 (51.8 - 53.7)	41.8 (40.7 - 42.8)	33.1 (31.8 - 34.3)

Appendix Table A7: Unadjusted Median Survival and Survival Probabilities by Age, females (Page 3 of 18)

Group	Diagnosis Category	Sex	Age	N	Median Survival (y)	1y (%)	3y (%)	5y (%)	10y (%)	15y (%)	20y (%)
Cancers	Lip oral cavity cancer	Female	16-24	144	NR	100.0 (100.0 - 100.0)	96.3 (91.3 - 98.4)	95.4 (90.1 - 97.9)	94.4 (88.5 - 97.3)	93.1 (86.3 - 96.5)	93.1 (86.3 - 96.5)
Cancers	Lip oral cavity cancer	Female	25-34	345	NR	97.3 (94.9 - 98.6)	93.6 (90.4 - 95.8)	92.3 (88.8 - 94.8)	91.2 (87.4 - 93.8)	89.6 (85.4 - 92.6)	89.6 (85.4 - 92.6)
Cancers	Lip oral cavity cancer	Female	35-44	667	NR	97.3 (95.7 - 98.3)	92.5 (90.1 - 94.3)	91.0 (88.4 - 92.9)	87.0 (84.0 - 89.5)	84.1 (80.6 - 87.0)	81.4 (77.2 - 85.0)
Cancers	Lip oral cavity cancer	Female	45-54	1397	NR	94.9 (93.6 - 96.0)	88.2 (86.3 - 89.8)	85.6 (83.6 - 87.4)	80.0 (77.6 - 82.2)	73.8 (70.9 - 76.5)	67.8 (64.2 - 71.2)
Cancers	Lip oral cavity cancer	Female	55-64	2636	21.5	92.3 (91.2 - 93.3)	84.1 (82.6 - 85.5)	79.7 (78.0 - 81.2)	70.2 (68.2 - 72.1)	62.6 (60.3 - 64.8)	52.8 (49.8 - 55.8)
Cancers	Lip oral cavity cancer	Female	65-74	3816	12.86 (13.49 - 12.14)	89.7 (88.7 - 90.6)	79.9 (78.6 - 81.2)	73.1 (71.6 - 74.6)	58.4 (56.5 - 60.2)	43.4 (41.1 - 45.6)	25.4 (22.5 - 28.4)
Cancers	Lip oral cavity cancer	Female	75-84	3511	6.57 (6.89 - 6.19)	85.3 (84.1 - 86.4)	70.1 (68.4 - 71.6)	58.9 (57.1 - 60.7)	31.7 (29.8 - 33.7)	12.9 (11.3 - 14.7)	2.6 (1.5 - 4.1)
Cancers	Lip oral cavity cancer	Female	85-95	1868	2.75 (3.02 - 2.53)	71.4 (69.2 - 73.4)	47.8 (45.4 - 50.1)	30.9 (28.7 - 33.2)	8.7 (7.1 - 10.4)	1.1 (0.5 - 2.1)	0.0 (0.0 - 0.0)
Cancers	Liver cancer	Female	All	5703	0.64 (0.69 - 0.59)	41.6 (40.3 - 42.9)	23.0 (21.8 - 24.1)	17.2 (16.2 - 18.3)	11.8 (10.9 - 12.9)	9.2 (8.3 - 10.3)	8.2 (7.1 - 9.3)
Cancers	Liver cancer	Female	16-24	32	NR	78.1 (59.5 - 88.9)	62.5 (43.5 - 76.7)	62.5 (43.5 - 76.7)	62.5 (43.5 - 76.7)	62.5 (43.5 - 76.7)	62.5 (43.5 - 76.7)
Cancers	Liver cancer	Female	25-34	60	5.49	68.5 (54.7 - 78.8)	59.2 (45.2 - 70.8)	51.1 (37.1 - 63.5)	44.4 (30.6 - 57.3)	44.4 (30.6 - 57.3)	38.8 (23.5 - 53.9)
Cancers	Liver cancer	Female	35-44	129	1.89 (5.99 - 0.91)	57.7 (48.7 - 65.8)	46.3 (37.4 - 54.7)	43.5 (34.6 - 52.0)	35.6 (26.8 - 44.5)	35.6 (26.8 - 44.5)	35.6 (26.8 - 44.5)
Cancers	Liver cancer	Female	45-54	393	1.3 (1.7 - 1.06)	56.7 (51.6 - 61.5)	36.4 (31.4 - 41.3)	30.1 (25.3 - 35.0)	25.6 (20.9 - 30.5)	23.8 (19.1 - 28.8)	22.6 (17.7 - 28.0)
Cancers	Liver cancer	Female	55-64	997	0.99 (1.16 - 0.84)	49.8 (46.7 - 52.9)	29.3 (26.4 - 32.2)	24.2 (21.4 - 27.1)	18.3 (15.7 - 21.1)	13.8 (11.1 - 16.8)	12.1 (9.3 - 15.2)
Cancers	Liver cancer	Female	65-74	1803	0.75 (0.85 - 0.65)	44.4 (42.0 - 46.7)	23.1 (21.1 - 25.2)	16.7 (14.9 - 18.7)	9.6 (7.9 - 11.5)	6.4 (4.7 - 8.4)	4.6 (3.0 - 6.8)
Cancers	Liver cancer	Female	75-84	1739	0.46 (0.52 - 0.41)	34.7 (32.5 - 37.0)	17.3 (15.5 - 19.3)	10.7 (9.1 - 12.5)	5.1 (3.9 - 6.7)	1.5 (0.6 - 2.9)	0.9 (0.3 - 2.2)
Cancers	Liver cancer	Female	85-95	538	0.21 (0.25 - 0.18)	19.4 (16.2 - 22.9)	6.4 (4.5 - 8.9)	2.2 (1.1 - 4.0)	0.8 (0.2 - 2.4)	0.0 (0.0 - 0.0)	0.0 (0.0 - 0.0)
Cancers	Lung cancer	Female	All	54909	0.87 (0.89 - 0.85)	47.0 (46.6 - 47.4)	27.5 (27.1 - 27.9)	21.2 (20.9 - 21.6)	13.7 (13.3 - 14.0)	9.3 (9.0 - 9.7)	6.6 (6.2 - 7.0)
Cancers	Lung cancer	Female	16-24	63	NR	91.9 (81.7 - 96.6)	83.9 (72.1 - 91.0)	82.2 (70.1 - 89.7)	80.4 (68.1 - 88.4)	80.4 (68.1 - 88.4)	76.9 (62.7 - 86.3)
Cancers	Lung cancer	Female	25-34	190	NR	81.8 (75.5 - 86.6)	70.7 (63.5 - 76.7)	68.3 (60.9 - 74.5)	65.0 (57.5 - 71.5)	63.0 (55.2 - 69.8)	60.6 (51.6 - 68.4)
Cancers	Lung cancer	Female	35-44	619	2.65 (4.5 - 2.05)	68.2 (64.3 - 71.7)	48.8 (44.7 - 52.7)	44.8 (40.8 - 48.8)	39.9 (35.7 - 44.0)	38.3 (34.0 - 42.4)	37.8 (33.5 - 42.0)
Cancers	Lung cancer	Female	45-54	2999	1.38 (1.49 - 1.26)	56.9 (55.1 - 58.6)	35.9 (34.1 - 37.6)	31.0 (29.3 - 32.7)	25.4 (23.7 - 27.1)	21.2 (19.5 - 22.9)	17.6 (15.8 - 19.6)
Cancers	Lung cancer	Female	55-64	10489	1.13 (1.19 - 1.09)	53.1 (52.1 - 54.0)	32.1 (31.1 - 33.0)	25.9 (25.1 - 26.8)	18.9 (18.1 - 19.8)	14.1 (13.3 - 15.0)	10.1 (9.1 - 11.0)
Cancers	Lung cancer	Female	65-74	19504	1.03 (1.06 - 1.0)	50.8 (50.1 - 51.5)	30.4 (29.8 - 31.1)	23.5 (22.9 - 24.2)	14.2 (13.6 - 14.8)	8.2 (7.6 - 8.9)	3.6 (2.9 - 4.4)
Cancers	Lung cancer	Female	75-84	16494	0.66 (0.69 - 0.64)	41.7 (40.9 - 42.4)	22.7 (22.0 - 23.3)	15.7 (15.1 - 16.3)	6.9 (6.4 - 7.5)	2.1 (1.7 - 2.6)	0.7 (0.4 - 1.2)
Cancers	Lung cancer	Female	85-95	4447	0.26 (0.28 - 0.24)	24.2 (23.0 - 25.5)	10.1 (9.2 - 11.1)	5.5 (4.7 - 6.3)	1.1 (0.7 - 1.7)	0.1 (0.0 - 0.7)	0.1 (0.0 - 0.7)
Cancers	Lymphoma	Female	All	22691	8.63 (8.92 - 8.36)	80.4 (79.8 - 80.9)	68.8 (68.1 - 69.4)	61.0 (60.3 - 61.7)	46.7 (45.9 - 47.4)	35.9 (35.1 - 36.7)	28.4 (27.5 - 29.4)
Cancers	Lymphoma	Female	16-24	324	NR	96.3 (93.5 - 97.9)	92.1 (88.5 - 94.6)	91.4 (87.7 - 94.0)	89.3 (85.2 - 92.4)	87.7 (83.2 - 91.1)	87.0 (82.2 - 90.6)
Cancers	Lymphoma	Female	25-34	645	NR	97.2 (95.5 - 98.2)	93.6 (91.4 - 95.3)	92.5 (90.1 - 94.4)	91.5 (88.9 - 93.5)	90.6 (87.8 - 92.8)	89.2 (85.7 - 91.9)
Cancers	Lymphoma	Female	35-44	934	NR	95.2 (93.6 - 96.4)	90.1 (87.9 - 91.9)	88.7 (86.4 - 90.6)	86.2 (83.6 - 88.4)	83.3 (80.3 - 85.9)	80.4 (76.8 - 83.5)
Cancers	Lymphoma	Female	45-54	1942	NR	93.7 (92.5 - 94.7)	88.0 (86.4 - 89.4)	84.9 (83.2 - 86.5)	79.5 (77.5 - 81.4)	73.9 (71.5 - 76.2)	66.8 (63.7 - 69.7)
Cancers	Lymphoma	Female	55-64	3829	19.04 (19.89 - 17.67)	90.6 (89.6 - 91.5)	82.4 (81.1 - 83.6)	77.7 (76.3 - 79.0)	68.0 (66.3 - 69.6)	56.7 (54.7 - 58.6)	47.4 (45.1 - 49.7)
Cancers	Lymphoma	Female	65-74	6052	9.95 (10.46 - 9.47)	84.2 (83.2 - 85.1)	73.5 (72.3 - 74.6)	65.9 (64.6 - 67.1)	49.9 (48.5 - 51.4)	33.3 (31.7 - 35.0)	18.1 (16.2 - 20.0)
Cancers	Lymphoma	Female	75-84	6397	4.25 (4.49 - 4.03)	72.6 (71.5 - 73.7)	57.5 (56.3 - 58.8)	46.1 (44.8 - 47.4)	22.0 (20.8 - 23.3)	7.8 (6.8 - 8.8)	2.0 (1.4 - 2.9)
Cancers	Lymphoma	Female	85-95	2488	1.25 (1.4 - 1.1)	53.8 (51.8 - 55.7)	33.5 (31.6 - 35.5)	20.4 (18.7 - 22.2)	5.4 (4.3 - 6.6)	0.7 (0.3 - 1.4)	0.5 (0.1 - 1.2)
Cancers	Malignant neoplasm of bone & articular cartilage	Female	All	3489	2.69 (3.03 - 2.34)	64.4 (62.7 - 65.9)	48.3 (46.6 - 50.0)	41.1 (39.4 - 42.7)	31.7 (30.1 - 33.4)	26.8 (25.1 - 28.4)	22.6 (20.8 - 24.4)
Cancers	Malignant neoplasm of bone & articular cartilage	Female	16-24	152	NR	92.0 (86.3 - 95.4)	80.0 (72.5 - 85.7)	74.7 (66.7 - 81.1)	71.4 (63.1 - 78.2)	70.4 (61.9 - 77.3)	66.4 (56.5 - 74.6)
Cancers	Malignant neoplasm of bone & articular cartilage	Female	25-34	173	NR	91.3 (85.9 - 94.6)	80.5 (73.7 - 85.8)	76.1 (68.8 - 81.9)	73.0 (65.4 - 79.2)	72.1 (64.3 - 78.4)	72.1 (64.3 - 78.4)
Cancers	Malignant neoplasm of bone & articular cartilage	Female	35-44	258	NR	84.3 (79.2 - 88.2)	71.7 (65.7 - 76.9)	67.8 (61.6 - 73.3)	60.9 (54.3 - 66.8)	59.5 (52.7 - 65.6)	57.1 (49.8 - 63.7)
Cancers	Malignant neoplasm of bone & articular cartilage	Female	45-54	356	14.46 (22.11 - 8.61)	75.6 (70.8 - 79.8)	64.6 (59.3 - 69.4)	59.7 (54.3 - 64.7)	52.9 (47.2 - 58.2)	49.7 (43.9 - 55.3)	45.3 (38.8 - 51.6)
Cancers	Malignant neoplasm of bone & articular cartilage	Female	55-64	638	4.13 (5.62 - 3.16)	70.9 (67.2 - 74.3)	54.3 (50.3 - 58.1)	47.9 (43.8 - 51.8)	37.6 (33.6 - 41.6)	32.0 (28.0 - 36.1)	24.8 (20.3 - 29.6)
Cancers	Malignant neoplasm of bone & articular cartilage	Female	65-74	819	2.13 (2.6 - 1.66)	61.5 (58.1 - 64.8)	43.8 (40.3 - 47.2)	36.3 (32.9 - 39.7)	24.4 (21.2 - 27.7)	14.3 (11.3 - 17.5)	7.5 (4.8 - 10.9)
Cancers	Malignant neoplasm of bone & articular cartilage	Female	75-84	784	0.93 (1.17 - 0.75)	49.1 (45.6 - 52.6)	31.1 (27.8 - 34.5)	20.2 (17.4 - 23.3)	10.1 (7.9 - 12.6)	5.0 (3.3 - 7.2)	1.5 (0.4 - 3.9)
Cancers	Malignant neoplasm of bone & articular cartilage	Female	85-95	296	0.51 (0.71 - 0.38)	38.0 (32.5 - 43.6)	18.9 (14.6 - 23.7)	11.1 (7.7 - 15.2)	0.5 (0.1 - 2.7)	0.5 (0.1 - 2.7)	0.0 (0.0 - 0.0)
Cancers	Melanoma	Female	All	46092	22.18	94.5 (94.3 - 94.8)	87.4 (87.1 - 87.7)	82.2 (81.8 - 82.5)	71.2 (70.7 - 71.7)	61.4 (60.7 - 62.0)	53.2 (52.4 - 54.1)
Cancers	Melanoma	Female	16-24	628	NR	99.2 (98.1 - 99.7)	98.5 (97.2 - 99.2)	97.1 (95.3 - 98.2)	96.2 (94.2 - 97.5)	95.7 (93.5 - 97.2)	93.6 (89.1 - 96.3)
Cancers	Melanoma	Female	25-34	2407	NR	99.0 (98.5 - 99.3)	97.4 (96.6 - 97.9)	96.4 (95.5 - 97.1)	95.1 (94.0 - 95.9)	93.8 (92.5 - 94.9)	93.0 (91.4 - 94.3)
Cancers	Melanoma	Female	35-44	4636	NR	98.5 (98.1 - 98.8)	96.3 (95.7 - 96.9)	94.9 (94.2 - 95.6)	92.5 (91.7 - 93.3)	90.3 (89.2 - 91.4)	88.8 (87.3 - 90.1)
Cancers	Melanoma	Female	45-54	6969	NR	97.6 (97.2 - 97.9)	95.4 (94.8 - 95.9)	93.5 (92.8 - 94.1)	90.0 (89.1 - 90.7)	86.2 (85.0 - 87.3)	81.8 (80.0 - 83.4)
Cancers	Melanoma	Female	55-64	8545	NR	96.3 (95.9 - 96.7)	92.2 (91.6 - 92.8)	89.4 (88.7 - 90.1)	83.4 (82.5 - 84.3)	77.4 (76.1 - 78.5)	68.7 (66.8 - 70.4)

Appendix Table A7: Unadjusted Median Survival and Survival Probabilities by Age, females (Page 4 of 18)

Group	Diagnosis Category	Sex	Age	N	Median Survival (y)	1y (%)	3y (%)	5y (%)	10y (%)	15y (%)	20y (%)
Cancers	Melanoma	Female	65-74	10300	17.17 (17.58 - 16.65)	95.0 (94.6 - 95.4)	89.0 (88.3 - 89.6)	84.4 (83.6 - 85.1)	73.0 (71.9 - 74.1)	57.3 (55.7 - 58.9)	37.5 (35.0 - 39.9)
Cancers	Melanoma	Female	75-84	8646	8.98 (9.23 - 8.74)	91.9 (91.3 - 92.5)	80.2 (79.2 - 81.0)	70.7 (69.6 - 71.8)	45.2 (43.8 - 46.6)	19.6 (18.1 - 21.2)	6.3 (5.0 - 7.8)
Cancers	Melanoma	Female	85-95	3754	3.65 (3.83 - 3.48)	82.3 (81.0 - 83.5)	56.4 (54.7 - 58.0)	38.2 (36.5 - 40.0)	10.8 (9.5 - 12.1)	1.0 (0.5 - 1.8)	0.1 (0.0 - 0.8)
Cancers	Myeloma	Female	All	12665	4.78 (4.93 - 4.61)	81.8 (81.1 - 82.5)	62.9 (62.0 - 63.7)	48.6 (47.7 - 49.5)	29.2 (28.3 - 30.2)	19.4 (18.4 - 20.4)	13.7 (12.6 - 14.8)
Cancers	Myeloma	Female	16-24	46	NR	93.3 (80.7 - 97.8)	88.9 (75.3 - 95.2)	86.5 (72.3 - 93.7)	86.5 (72.3 - 93.7)	86.5 (72.3 - 93.7)	86.5 (72.3 - 93.7)
Cancers	Myeloma	Female	25-34	85	NR	94.0 (86.3 - 97.5)	89.0 (80.0 - 94.1)	83.5 (73.2 - 90.1)	83.5 (73.2 - 90.1)	81.0 (69.4 - 88.5)	81.0 (69.4 - 88.5)
Cancers	Myeloma	Female	35-44	264	NR	95.4 (92.0 - 97.3)	90.5 (86.1 - 93.5)	85.5 (80.4 - 89.4)	76.0 (69.5 - 81.3)	69.1 (61.4 - 75.6)	62.9 (51.1 - 72.5)
Cancers	Myeloma	Female	45-54	780	16.17 (19.77 - 14.16)	94.4 (92.5 - 95.8)	86.6 (83.9 - 88.9)	78.9 (75.6 - 81.7)	65.4 (61.3 - 69.1)	53.4 (48.6 - 58.0)	43.0 (37.0 - 48.8)
Cancers	Myeloma	Female	55-64	1991	11.07 (12.41 - 10.13)	91.8 (90.5 - 92.9)	81.5 (79.7 - 83.2)	70.9 (68.7 - 72.9)	53.0 (50.4 - 55.5)	41.4 (38.6 - 44.3)	31.4 (28.0 - 34.9)
Cancers	Myeloma	Female	65-74	3640	6.01 (6.37 - 5.7)	86.5 (85.4 - 87.6)	69.7 (68.1 - 71.2)	55.3 (53.5 - 57.0)	31.5 (29.6 - 33.4)	17.1 (15.2 - 19.1)	7.9 (6.0 - 10.1)
Cancers	Myeloma	Female	75-84	4164	3.45 (3.58 - 3.28)	78.2 (76.9 - 79.5)	54.6 (53.0 - 56.1)	35.8 (34.2 - 37.4)	12.4 (11.1 - 13.8)	3.1 (2.3 - 4.1)	0.9 (0.5 - 1.7)
Cancers	Myeloma	Female	85-95	1663	1.49 (1.62 - 1.33)	59.9 (57.5 - 62.3)	28.9 (26.6 - 31.2)	14.7 (12.9 - 16.7)	2.9 (2.0 - 4.1)	0.4 (0.1 - 1.3)	0.4 (0.1 - 1.3)
Cancers	Nasopharynx cancer	Female	All	653	10.08 (12.11 - 8.19)	83.1 (80.0 - 85.8)	70.9 (67.2 - 74.3)	62.1 (58.1 - 65.8)	50.1 (45.9 - 54.2)	39.3 (34.7 - 43.7)	28.7 (23.4 - 34.2)
Cancers	Nasopharynx cancer	Female	16-24	6	NR	100.0 (100.0 - 100.0)	83.3 (27.3 - 97.5)	83.3 (27.3 - 97.5)	83.3 (27.3 - 97.5)	83.3 (27.3 - 97.5)	83.3 (27.3 - 97.5)
Cancers	Nasopharynx cancer	Female	25-34	43	NR	97.6 (83.9 - 99.7)	86.7 (71.0 - 94.3)	86.7 (71.0 - 94.3)	83.0 (65.5 - 92.1)	78.1 (58.2 - 89.3)	71.6 (48.5 - 85.7)
Cancers	Nasopharynx cancer	Female	35-44	47	NR	93.6 (81.5 - 97.9)	89.2 (76.0 - 95.4)	86.7 (72.6 - 93.8)	81.1 (65.5 - 90.2)	81.1 (65.5 - 90.2)	81.1 (65.5 - 90.2)
Cancers	Nasopharynx cancer	Female	45-54	93	NR	88.9 (80.3 - 93.9)	84.1 (74.6 - 90.3)	81.5 (71.5 - 88.2)	76.2 (64.8 - 84.3)	65.4 (51.9 - 76.0)	61.0 (45.6 - 73.3)
Cancers	Nasopharynx cancer	Female	55-64	165	12.69 (16.36 - 9.33)	85.2 (78.8 - 89.9)	75.6 (68.1 - 81.5)	65.1 (56.9 - 72.1)	54.1 (45.3 - 62.0)	43.7 (34.4 - 52.6)	27.2 (16.6 - 39.0)
Cancers	Nasopharynx cancer	Female	65-74	143	10.82 (12.72 - 7.16)	83.9 (76.7 - 89.0)	71.5 (63.2 - 78.2)	61.4 (52.6 - 69.0)	50.3 (41.2 - 58.8)	33.0 (23.5 - 42.7)	6.2 (0.6 - 21.4)
Cancers	Nasopharynx cancer	Female	75-84	110	3.5 (4.42 - 2.03)	73.6 (64.3 - 80.9)	53.3 (43.5 - 62.1)	40.4 (31.0 - 49.6)	18.3 (11.2 - 26.8)	5.3 (1.8 - 11.7)	0.0 (0.0 - 0.0)
Cancers	Nasopharynx cancer	Female	85-95	44	1.7 (2.73 - 0.72)	58.4 (42.4 - 71.4)	34.8 (21.0 - 48.9)	19.9 (9.4 - 33.1)	0.0 (0.0 - 0.0)	0.0 (0.0 - 0.0)	0.0 (0.0 - 0.0)
Cancers	Other pharynx cancer	Female	All	3394	10.12 (10.98 - 9.34)	80.9 (79.5 - 82.2)	69.0 (67.3 - 70.5)	61.8 (60.1 - 63.5)	50.4 (48.5 - 52.2)	40.5 (38.4 - 42.6)	33.7 (31.2 - 36.1)
Cancers	Other pharynx cancer	Female	16-24	30	NR	100.0 (100.0 - 100.0)	100.0 (100.0 - 100.0)	100.0 (100.0 - 100.0)	100.0 (100.0 - 100.0)	100.0 (100.0 - 100.0)	100.0 (100.0 - 100.0)
Cancers	Other pharynx cancer	Female	25-34	68	NR	98.5 (89.9 - 99.8)	94.0 (84.7 - 97.7)	94.0 (84.7 - 97.7)	92.3 (82.4 - 96.7)	92.3 (82.4 - 96.7)	92.3 (82.4 - 96.7)
Cancers	Other pharynx cancer	Female	35-44	177	NR	97.2 (93.3 - 98.8)	86.0 (79.8 - 90.4)	84.1 (77.7 - 88.8)	79.5 (72.3 - 85.0)	72.8 (64.5 - 79.5)	68.3 (58.6 - 76.1)
Cancers	Other pharynx cancer	Female	45-54	515	NR	91.1 (88.3 - 93.3)	86.6 (83.3 - 89.3)	82.7 (79.0 - 85.8)	76.7 (72.4 - 80.4)	69.9 (64.8 - 74.4)	66.3 (60.4 - 71.5)
Cancers	Other pharynx cancer	Female	55-64	914	14.56 (16.71 - 11.86)	85.6 (83.1 - 87.7)	74.8 (71.7 - 77.6)	67.3 (64.0 - 70.4)	57.9 (54.3 - 61.4)	49.6 (45.4 - 53.6)	40.0 (35.0 - 45.0)
Cancers	Other pharynx cancer	Female	65-74	876	7.99 (9.01 - 6.93)	79.6 (76.7 - 82.1)	64.8 (61.5 - 68.0)	58.7 (55.2 - 62.0)	43.4 (39.5 - 47.3)	24.9 (20.5 - 29.4)	12.0 (7.5 - 17.6)
Cancers	Other pharynx cancer	Female	75-84	625	3.58 (4.1 - 2.73)	68.1 (64.3 - 71.6)	53.0 (48.9 - 57.0)	39.6 (35.5 - 43.7)	20.9 (17.2 - 24.9)	9.4 (6.5 - 13.0)	1.7 (0.2 - 6.5)
Cancers	Other pharynx cancer	Female	85-95	185	1.19 (1.82 - 0.83)	54.6 (47.1 - 61.5)	33.7 (26.8 - 40.8)	24.3 (18.1 - 31.0)	6.9 (3.1 - 12.9)	1.7 (0.2 - 7.6)	1.7 (0.2 - 7.6)
Cancers	Ovarian cancer	Female	All	26767	4.29 (4.43 - 4.17)	78.8 (78.3 - 79.3)	57.7 (57.0 - 58.3)	47.1 (46.4 - 47.7)	35.7 (35.0 - 36.3)	29.5 (28.8 - 30.1)	24.1 (23.5 - 24.8)
Cancers	Ovarian cancer	Female	16-24	252	NR	95.1 (91.5 - 97.2)	91.5 (87.1 - 94.4)	91.0 (86.5 - 94.0)	87.4 (82.1 - 91.3)	86.5 (80.8 - 90.6)	86.5 (80.8 - 90.6)
Cancers	Ovarian cancer	Female	25-34	610	NR	95.0 (92.9 - 96.5)	87.5 (84.5 - 90.0)	84.0 (80.7 - 86.8)	80.6 (77.0 - 83.8)	78.2 (74.3 - 81.6)	75.2 (70.6 - 79.1)
Cancers	Ovarian cancer	Female	35-44	1413	NR	92.3 (90.8 - 93.6)	81.2 (79.0 - 83.1)	73.8 (71.3 - 76.1)	65.2 (62.5 - 67.8)	63.1 (60.3 - 65.8)	61.0 (58.0 - 63.9)
Cancers	Ovarian cancer	Female	45-54	3643	17.36 (19.76 - 15.14)	92.2 (91.2 - 93.0)	75.9 (74.5 - 77.3)	67.3 (65.7 - 68.9)	56.5 (54.7 - 58.2)	52.0 (50.2 - 53.8)	47.8 (45.8 - 49.8)
Cancers	Ovarian cancer	Female	55-64	6297	6.32 (6.84 - 5.9)	86.4 (85.5 - 87.2)	66.0 (64.8 - 67.2)	54.6 (53.3 - 55.9)	43.2 (41.9 - 44.6)	37.7 (36.3 - 39.1)	31.8 (30.3 - 33.3)
Cancers	Ovarian cancer	Female	65-74	7379	3.6 (3.77 - 3.47)	80.2 (79.3 - 81.1)	55.1 (53.9 - 56.2)	42.5 (41.3 - 43.7)	30.2 (29.0 - 31.3)	21.3 (20.2 - 22.5)	12.5 (11.3 - 13.8)
Cancers	Ovarian cancer	Female	75-84	5556	1.92 (2.02 - 1.82)	65.0 (63.7 - 66.2)	39.4 (38.1 - 40.7)	28.4 (27.1 - 29.6)	14.9 (13.8 - 16.0)	6.6 (5.8 - 7.5)	2.0 (1.4 - 2.7)
Cancers	Ovarian cancer	Female	85-95	1586	0.61 (0.68 - 0.51)	39.5 (37.1 - 41.9)	20.8 (18.8 - 22.9)	11.9 (10.3 - 13.6)	2.6 (1.8 - 3.6)	0.6 (0.3 - 1.3)	0.0 (0.0 - 0.0)
Cancers	Pancreatic cancer	Female	All	23669	0.39 (0.4 - 0.38)	28.7 (28.1 - 29.2)	13.1 (12.7 - 13.6)	10.0 (9.6 - 10.5)	6.9 (6.6 - 7.3)	5.3 (4.9 - 5.7)	4.1 (3.6 - 4.5)
Cancers	Pancreatic cancer	Female	16-24	32	NR	100.0 (100.0 - 100.0)	100.0 (100.0 - 100.0)	100.0 (100.0 - 100.0)	88.7 (61.4 - 97.1)	88.7 (61.4 - 97.1)	88.7 (61.4 - 97.1)
Cancers	Pancreatic cancer	Female	25-34	100	NR	85.1 (76.1 - 90.9)	76.1 (66.0 - 83.6)	72.3 (61.8 - 80.4)	63.7 (51.8 - 73.4)	63.7 (51.8 - 73.4)	63.7 (51.8 - 73.4)
Cancers	Pancreatic cancer	Female	35-44	318	4.28 (11.39 - 2.38)	69.9 (64.5 - 74.7)	52.5 (46.6 - 58.0)	48.4 (42.5 - 54.0)	44.7 (38.7 - 50.6)	40.5 (33.8 - 47.2)	38.8 (31.5 - 46.0)
Cancers	Pancreatic cancer	Female	45-54	1134	1.12 (1.27 - 1.0)	53.1 (50.1 - 55.9)	30.6 (27.9 - 33.4)	26.7 (24.0 - 29.5)	21.9 (19.3 - 24.7)	19.9 (17.2 - 22.7)	16.8 (13.7 - 20.2)
Cancers	Pancreatic cancer	Female	55-64	3450	0.74 (0.78 - 0.69)	41.0 (39.3 - 42.7)	18.9 (17.6 - 20.3)	15.0 (13.7 - 16.3)	11.3 (10.1 - 12.5)	9.4 (8.2 - 10.7)	7.7 (6.4 - 9.2)
Cancers	Pancreatic cancer	Female	65-74	6867	0.55 (0.58 - 0.52)	34.1 (33.0 - 35.3)	14.4 (13.5 - 15.3)	10.6 (9.9 - 11.4)	6.8 (6.1 - 7.6)	4.7 (3.9 - 5.5)	3.0 (2.2 - 3.9)
Cancers	Pancreatic cancer	Female	75-84	7904	0.29 (0.3 - 0.27)	21.3 (20.4 - 22.2)	9.3 (8.7 - 10.0)	6.3 (5.7 - 6.9)	3.2 (2.7 - 3.8)	1.4 (1.0 - 1.8)	0.2 (0.0 - 0.9)
Cancers	Pancreatic cancer	Female	85-95	3733	0.16 (0.17 - 0.15)	10.6 (9.6 - 11.6)	3.0 (2.4 - 3.6)	1.8 (1.4 - 2.3)	0.5 (0.3 - 0.8)	0.0 (0.0 - 0.0)	0.0 (0.0 - 0.0)
Cancers	Soft tissue and other extraosseous sarcomas	Female	All	9741	6.42 (6.86 - 5.97)	76.7 (75.9 - 77.6)	61.5 (60.5 - 62.5)	53.5 (52.4 - 54.5)	42.6 (41.5 - 43.6)	34.7 (33.6 - 35.9)	28.9 (27.6 - 30.2)
Cancers	Soft tissue and other extraosseous sarcomas	Female	16-24	258	NR	91.3 (87.0 - 94.2)	81.9 (76.4 - 86.2)	77.7 (71.8 - 82.5)	74.8 (68.6 - 80.0)	74.8 (68.6 - 80.0)	71.5 (63.6 - 77.9)

Group	Diagnosis Category	Sex	Age	N	Median Survival (y)	1y (%)	3y (%)	5y (%)	10y (%)	15y (%)	20y (%)
Cancers	Soft tissue and other extraosseous sarcomas	Female	25-34	406	NR	88.6 (85.0 - 91.3)	83.9 (79.8 - 87.1)	80.7 (76.4 - 84.3)	77.3 (72.7 - 81.3)	76.1 (71.3 - 80.2)	74.8 (69.7 - 79.2)
Cancers	Soft tissue and other extraosseous sarcomas	Female	35-44	705	NR	90.1 (87.6 - 92.1)	80.8 (77.7 - 83.6)	75.4 (71.9 - 78.5)	69.9 (66.1 - 73.4)	65.4 (61.2 - 69.3)	62.4 (57.7 - 66.7)
Cancers	Soft tissue and other extraosseous sarcomas	Female	45-54	1159	NR	87.1 (85.0 - 88.9)	75.9 (73.3 - 78.4)	70.0 (67.1 - 72.7)	64.4 (61.4 - 67.3)	58.5 (55.2 - 61.8)	53.7 (49.8 - 57.4)
Cancers	Soft tissue and other extraosseous sarcomas	Female	55-64	1852	9.48 (11.75 - 7.73)	80.2 (78.3 - 81.9)	64.7 (62.4 - 66.9)	58.1 (55.7 - 60.4)	49.4 (46.9 - 51.8)	42.8 (40.1 - 45.4)	37.9 (35.0 - 40.9)
Cancers	Soft tissue and other extraosseous sarcomas	Female	65-74	2322	5.46 (6.21 - 4.72)	75.9 (74.1 - 77.6)	59.4 (57.3 - 61.4)	51.2 (49.1 - 53.3)	38.7 (36.5 - 40.9)	28.2 (25.8 - 30.6)	16.7 (14.1 - 19.6)
Cancers	Soft tissue and other extraosseous sarcomas	Female	75-84	2085	3.28 (3.58 - 2.89)	68.5 (66.4 - 70.5)	51.6 (49.3 - 53.8)	41.4 (39.1 - 43.6)	23.7 (21.6 - 26.0)	10.4 (8.6 - 12.4)	2.9 (1.7 - 4.6)
Cancers	Soft tissue and other extraosseous sarcomas	Female	85-95	915	1.57 (1.84 - 1.34)	59.1 (55.8 - 62.2)	36.0 (32.8 - 39.2)	22.2 (19.4 - 25.1)	6.9 (5.2 - 9.0)	1.2 (0.5 - 2.5)	0.6 (0.1 - 1.8)
Cancers	Stomach cancer	Female	All	9664	0.94 (0.99 - 0.89)	48.9 (47.9 - 49.9)	30.8 (29.9 - 31.8)	25.2 (24.3 - 26.1)	17.9 (17.1 - 18.8)	12.2 (11.4 - 13.1)	8.9 (8.0 - 9.9)
Cancers	Stomach cancer	Female	16-24	15	NR	86.7 (56.4 - 96.5)	63.0 (32.3 - 82.8)	63.0 (32.3 - 82.8)	63.0 (32.3 - 82.8)	63.0 (32.3 - 82.8)	63.0 (32.3 - 82.8)
Cancers	Stomach cancer	Female	25-34	85	1.34 (5.08 - 0.9)	59.4 (47.9 - 69.2)	41.5 (30.6 - 52.0)	40.2 (29.4 - 50.7)	38.6 (27.9 - 49.2)	38.6 (27.9 - 49.2)	38.6 (27.9 - 49.2)
Cancers	Stomach cancer	Female	35-44	267	2.59 (5.18 - 1.81)	66.6 (60.5 - 71.9)	47.2 (40.9 - 53.2)	44.5 (38.3 - 50.6)	41.4 (35.1 - 47.5)	37.1 (30.4 - 43.7)	37.1 (30.4 - 43.7)
Cancers	Stomach cancer	Female	45-54	657	2.15 (2.61 - 1.76)	67.2 (63.4 - 70.7)	44.5 (40.5 - 48.4)	38.7 (34.8 - 42.6)	35.1 (31.2 - 39.1)	32.0 (27.9 - 36.1)	30.7 (26.1 - 35.4)
Cancers	Stomach cancer	Female	55-64	1452	1.79 (2.08 - 1.59)	61.3 (58.7 - 63.8)	41.5 (38.9 - 44.2)	36.2 (33.6 - 38.8)	29.7 (27.1 - 32.3)	24.6 (22.0 - 27.3)	20.7 (17.8 - 23.7)
Cancers	Stomach cancer	Female	65-74	2546	1.34 (1.46 - 1.23)	55.1 (53.1 - 57.0)	36.1 (34.2 - 38.0)	30.4 (28.6 - 32.3)	22.5 (20.7 - 24.4)	14.3 (12.4 - 16.4)	8.2 (6.1 - 10.8)
Cancers	Stomach cancer	Female	75-84	3148	0.67 (0.74 - 0.61)	42.3 (40.5 - 44.0)	25.7 (24.1 - 27.3)	19.6 (18.2 - 21.1)	10.2 (9.0 - 11.5)	3.3 (2.5 - 4.3)	0.5 (0.2 - 1.2)
Cancers	Stomach cancer	Female	85-95	1453	0.37 (0.4 - 0.32)	28.4 (26.1 - 30.7)	12.6 (10.9 - 14.4)	6.9 (5.6 - 8.4)	1.7 (1.0 - 2.7)	0.1 (0.0 - 0.7)	0.0 (0.0 - 0.0)
Cancers	Thyroid cancer	Female	All	10425	NR	90.6 (90.1 - 91.2)	86.8 (86.1 - 87.4)	83.7 (83.0 - 84.4)	76.7 (75.7 - 77.6)	69.6 (68.5 - 70.7)	62.5 (61.0 - 63.9)
Cancers	Thyroid cancer	Female	16-24	401	NR	99.7 (98.2 - 100.0)	99.5 (97.9 - 99.9)	99.1 (97.3 - 99.7)	97.9 (95.3 - 99.1)	97.9 (95.3 - 99.1)	97.9 (95.3 - 99.1)
Cancers	Thyroid cancer	Female	25-34	1258	NR	99.6 (99.0 - 99.8)	99.6 (99.0 - 99.8)	99.5 (98.9 - 99.8)	99.0 (98.0 - 99.4)	98.8 (97.7 - 99.3)	96.7 (94.0 - 98.2)
Cancers	Thyroid cancer	Female	35-44	1749	NR	99.5 (99.0 - 99.7)	98.8 (98.2 - 99.2)	98.5 (97.8 - 99.0)	97.9 (97.0 - 98.5)	96.4 (95.1 - 97.4)	95.1 (93.2 - 96.4)
Cancers	Thyroid cancer	Female	45-54	1863	NR	96.8 (95.9 - 97.5)	95.8 (94.7 - 96.6)	94.6 (93.4 - 95.6)	91.7 (90.1 - 93.0)	88.8 (86.8 - 90.5)	84.5 (81.8 - 86.8)
Cancers	Thyroid cancer	Female	55-64	1756	NR	94.0 (92.7 - 95.0)	90.3 (88.7 - 91.6)	87.7 (86.0 - 89.2)	81.3 (79.2 - 83.3)	74.4 (71.8 - 76.9)	66.2 (62.8 - 69.4)
Cancers	Thyroid cancer	Female	65-74	1703	14.02 (14.97 - 12.83)	84.2 (82.4 - 85.9)	77.9 (75.8 - 79.9)	73.5 (71.2 - 75.7)	61.6 (58.9 - 64.3)	46.5 (43.0 - 49.8)	29.3 (25.5 - 33.2)
Cancers	Thyroid cancer	Female	75-84	1289	6.18 (6.95 - 5.39)	75.2 (72.7 - 77.5)	63.8 (61.0 - 66.5)	54.1 (51.2 - 57.0)	32.9 (29.8 - 36.1)	13.0 (10.3 - 16.0)	2.9 (1.5 - 5.1)
Cancers	Thyroid cancer	Female	85-95	389	1.1 (1.83 - 0.62)	50.6 (45.5 - 55.5)	37.3 (32.4 - 42.2)	25.3 (20.9 - 30.0)	6.9 (4.2 - 10.4)	1.8 (0.6 - 4.5)	1.8 (0.6 - 4.5)
Cancers	Uterine cancer	Female	All	39339	13.09 (13.32 - 12.84)	89.2 (88.9 - 89.5)	77.4 (77.0 - 77.8)	70.2 (69.8 - 70.7)	57.2 (56.7 - 57.8)	45.2 (44.6 - 45.8)	33.3 (32.6 - 34.1)
Cancers	Uterine cancer	Female	16-24	31	NR	83.9 (65.5 - 92.9)	73.8 (54.3 - 85.9)	73.8 (54.3 - 85.9)	73.8 (54.3 - 85.9)	73.8 (54.3 - 85.9)	73.8 (54.3 - 85.9)
Cancers	Uterine cancer	Female	25-34	261	NR	94.9 (91.4 - 97.0)	88.6 (83.9 - 92.0)	86.3 (81.2 - 90.1)	83.1 (77.4 - 87.5)	81.5 (75.4 - 86.3)	81.5 (75.4 - 86.3)
Cancers	Uterine cancer	Female	35-44	938	NR	95.0 (93.3 - 96.2)	90.4 (88.2 - 92.1)	87.3 (84.9 - 89.4)	81.6 (78.6 - 84.1)	77.7 (74.3 - 80.7)	75.7 (72.0 - 79.1)
Cancers	Uterine cancer	Female	45-54	3427	NR	94.8 (94.0 - 95.5)	88.8 (87.7 - 89.9)	85.6 (84.3 - 86.8)	81.9 (80.4 - 83.2)	77.8 (76.1 - 79.4)	72.3 (70.1 - 74.4)
Cancers	Uterine cancer	Female	55-64	8972	NR	94.4 (93.9 - 94.8)	87.4 (86.6 - 88.1)	83.1 (82.3 - 83.9)	76.4 (75.4 - 77.3)	69.2 (68.0 - 70.3)	58.8 (57.3 - 60.3)
Cancers	Uterine cancer	Female	65-74	12452	14.72 (14.94 - 14.41)	92.1 (91.6 - 92.5)	81.2 (80.5 - 81.9)	75.2 (74.4 - 75.9)	63.1 (62.2 - 64.1)	48.5 (47.4 - 49.7)	28.9 (27.4 - 30.4)
Cancers	Uterine cancer	Female	75-84	10029	6.82 (7.06 - 6.51)	84.7 (84.0 - 85.4)	68.8 (67.8 - 69.7)	58.0 (56.9 - 59.0)	35.4 (34.3 - 36.5)	15.4 (14.5 - 16.5)	3.9 (3.2 - 4.7)
Cancers	Uterine cancer	Female	85-95	3152	2.43 (2.66 - 2.24)	69.9 (68.2 - 71.5)	45.3 (43.4 - 47.0)	30.4 (28.7 - 32.1)	9.1 (7.9 - 10.4)	1.4 (0.9 - 2.2)	0.0 (0.0 - 0.0)
Cardiovascular diseases	Acute myocardial infarction	Female	All	178656	5.22 (5.28 - 5.18)	74.3 (74.1 - 74.5)	61.3 (61.0 - 61.5)	51.0 (50.8 - 51.3)	32.6 (32.4 - 32.8)	20.6 (20.4 - 20.9)	12.6 (12.3 - 12.9)
Cardiovascular diseases	Acute myocardial infarction	Female	16-24	64	NR	93.8 (84.2 - 97.6)	90.4 (79.9 - 95.6)	90.4 (79.9 - 95.6)	88.3 (76.9 - 94.3)	84.6 (70.3 - 92.4)	84.6 (70.3 - 92.4)
Cardiovascular diseases	Acute myocardial infarction	Female	25-34	301	NR	94.3 (91.0 - 96.4)	93.6 (90.2 - 95.9)	93.6 (90.2 - 95.9)	90.0 (85.4 - 93.1)	86.8 (81.5 - 90.7)	82.0 (74.7 - 87.4)
Cardiovascular diseases	Acute myocardial infarction	Female	35-44	1731	NR	96.4 (95.4 - 97.2)	95.5 (94.4 - 96.4)	93.7 (92.5 - 94.8)	90.4 (88.8 - 91.8)	86.1 (84.0 - 88.0)	79.6 (76.4 - 82.4)
Cardiovascular diseases	Acute myocardial infarction	Female	45-54	7453	NR	95.5 (95.0 - 95.9)	93.1 (92.5 - 93.7)	91.2 (90.5 - 91.8)	86.3 (85.4 - 87.1)	79.3 (78.1 - 80.4)	68.9 (67.1 - 70.6)
Cardiovascular diseases	Acute myocardial infarction	Female	55-64	18462	19.28 (19.57 - 18.81)	92.8 (92.4 - 93.2)	89.0 (88.5 - 89.4)	85.5 (85.0 - 86.0)	75.2 (74.5 - 75.9)	62.6 (61.7 - 63.5)	47.4 (46.2 - 48.7)
Cardiovascular diseases	Acute myocardial infarction	Female	65-74	36746	11.42 (11.6 - 11.26)	87.2 (86.8 - 87.5)	80.0 (79.6 - 80.4)	73.5 (73.0 - 74.0)	55.4 (54.9 - 56.0)	35.9 (35.2 - 36.5)	17.5 (16.8 - 18.2)
Cardiovascular diseases	Acute myocardial infarction	Female	75-84	60651	4.8 (4.87 - 4.73)	74.8 (74.5 - 75.2)	61.1 (60.7 - 61.5)	48.8 (48.3 - 49.2)	23.5 (23.1 - 23.9)	7.8 (7.5 - 8.1)	1.6 (1.4 - 1.8)
Cardiovascular diseases	Acute myocardial infarction	Female	85-95	50189	1.45 (1.49 - 1.41)	55.6 (55.2 - 56.1)	34.4 (34.0 - 34.9)	20.2 (19.8 - 20.6)	3.7 (3.6 - 3.9)	0.4 (0.3 - 0.4)	0.1 (0.0 - 0.1)
Cardiovascular diseases	Angina	Female	All	185543	10.5 (10.58 - 10.44)	90.4 (90.3 - 90.6)	80.5 (80.3 - 80.7)	71.4 (71.2 - 71.6)	51.7 (51.5 - 52.0)	36.3 (36.0 - 36.6)	24.4 (24.1 - 24.7)
Cardiovascular diseases	Angina	Female	16-24	75	NR	98.6 (90.8 - 99.8)	98.6 (90.8 - 99.8)	98.6 (90.8 - 99.8)	95.6 (87.0 - 98.6)	95.6 (87.0 - 98.6)	95.6 (87.0 - 98.6)
Cardiovascular diseases	Angina	Female	25-34	239	NR	98.3 (95.6 - 99.4)	97.8 (94.9 - 99.1)	97.8 (94.9 - 99.1)	94.2 (90.0 - 96.7)	93.0 (88.4 - 95.8)	91.3 (86.0 - 94.7)
Cardiovascular diseases	Angina	Female	35-44	2261	NR	99.5 (99.1 - 99.7)	98.6 (98.0 - 99.0)	97.8 (97.1 - 98.3)	95.4 (94.4 - 96.3)	91.7 (90.3 - 92.9)	85.9 (83.8 - 87.8)
Cardiovascular diseases	Angina	Female	45-54	11931	NR	99.0 (98.9 - 99.2)	97.7 (97.5 - 98.0)	96.7 (96.4 - 97.0)	92.9 (92.4 - 93.4)	87.5 (86.8 - 88.2)	79.9 (78.9 - 80.9)
Cardiovascular diseases	Angina	Female	55-64	28896	NR	98.4 (98.2 - 98.5)	96.2 (96.0 - 96.5)	93.8 (93.5 - 94.0)	86.6 (86.2 - 87.0)	76.8 (76.2 - 77.3)	62.5 (61.7 - 63.3)
Cardiovascular diseases	Angina	Female	65-74	46965	15.19 (15.33 - 15.07)	96.2 (96.0 - 96.4)	91.8 (91.6 - 92.1)	86.8 (86.5 - 87.1)	71.4 (71.0 - 71.9)	50.9 (50.3 - 51.4)	27.6 (26.9 - 28.2)

Appendix Table A7: Unadjusted Median Survival and Survival Probabilities by Age, females (Page 6 of 18)

Group	Diagnosis Category	Sex	Age	N	Median Survival (y)	1y (%)	3y (%)	5y (%)	10y (%)	15y (%)	20y (%)
Cardiovascular diseases	Angina	Female	75-84	58224	7.81 (7.89 - 7.74)	90.3 (90.1 - 90.6)	79.0 (78.6 - 79.3)	67.3 (66.9 - 67.7)	37.1 (36.7 - 37.5)	13.8 (13.5 - 14.2)	3.0 (2.8 - 3.3)
Cardiovascular diseases	Angina	Female	85-95	34981	3.1 (3.14 - 3.05)	74.8 (74.3 - 75.2)	51.0 (50.5 - 51.5)	32.4 (31.9 - 32.9)	7.1 (6.8 - 7.4)	0.8 (0.7 - 1.0)	0.1 (0.0 - 0.2)
Cardiovascular diseases	Aortic aneurysm	Female	All	30991	6.5 (6.64 - 6.38)	81.6 (81.1 - 82.0)	68.6 (68.0 - 69.1)	57.2 (56.6 - 57.8)	35.8 (35.1 - 36.4)	21.6 (20.9 - 22.3)	13.6 (12.7 - 14.4)
Cardiovascular diseases	Aortic aneurysm	Female	16-24	113	NR	99.1 (93.9 - 99.9)	99.1 (93.9 - 99.9)	99.1 (93.9 - 99.9)	97.3 (88.9 - 99.4)	97.3 (88.9 - 99.4)	97.3 (88.9 - 99.4)
Cardiovascular diseases	Aortic aneurysm	Female	25-34	300	NR	98.3 (96.0 - 99.3)	97.9 (95.4 - 99.1)	96.4 (93.1 - 98.1)	92.2 (87.0 - 95.3)	92.2 (87.0 - 95.3)	85.9 (73.3 - 92.8)
Cardiovascular diseases	Aortic aneurysm	Female	35-44	564	NR	98.4 (96.9 - 99.1)	97.0 (95.1 - 98.2)	96.0 (93.7 - 97.4)	93.6 (90.6 - 95.7)	90.1 (85.7 - 93.2)	75.8 (64.7 - 83.8)
Cardiovascular diseases	Aortic aneurysm	Female	45-54	1236	NR	97.0 (95.9 - 97.8)	94.4 (92.9 - 95.6)	93.0 (91.2 - 94.4)	87.1 (84.5 - 89.3)	79.9 (76.0 - 83.2)	66.0 (59.2 - 72.0)
Cardiovascular diseases	Aortic aneurysm	Female	55-64	3595	16.31 (17.13 - 15.63)	94.4 (93.5 - 95.1)	90.3 (89.2 - 91.3)	85.2 (83.9 - 86.4)	71.5 (69.6 - 73.3)	54.5 (52.0 - 57.0)	40.5 (37.0 - 43.9)
Cardiovascular diseases	Aortic aneurysm	Female	65-74	9200	9.55 (9.75 - 9.25)	89.5 (88.8 - 90.1)	80.4 (79.5 - 81.2)	71.2 (70.2 - 72.2)	47.5 (46.2 - 48.8)	25.5 (24.0 - 27.0)	11.9 (10.3 - 13.6)
Cardiovascular diseases	Aortic aneurysm	Female	75-84	11384	4.58 (4.73 - 4.41)	78.3 (77.5 - 79.0)	62.2 (61.3 - 63.1)	47.0 (46.0 - 48.0)	18.9 (18.0 - 19.9)	5.2 (4.5 - 6.0)	0.8 (0.4 - 1.4)
Cardiovascular diseases	Aortic aneurysm	Female	85-95	4405	1.5 (1.61 - 1.38)	57.1 (55.6 - 58.5)	32.0 (30.5 - 33.4)	16.8 (15.6 - 18.0)	2.3 (1.7 - 3.0)	0.3 (0.1 - 0.8)	0.3 (0.1 - 0.8)
Cardiovascular diseases	Atrial fibrillation	Female	All	366575	6.2 (6.24 - 6.17)	82.0 (81.9 - 82.1)	68.0 (67.8 - 68.1)	56.1 (56.0 - 56.3)	34.6 (34.4 - 34.7)	21.1 (21.0 - 21.3)	12.9 (12.7 - 13.1)
Cardiovascular diseases	Atrial fibrillation	Female	16-24	471	NR	97.7 (95.8 - 98.7)	97.0 (94.9 - 98.2)	96.7 (94.6 - 98.0)	95.3 (92.7 - 96.9)	94.0 (90.9 - 96.0)	94.0 (90.9 - 96.0)
Cardiovascular diseases	Atrial fibrillation	Female	25-34	1272	NR	98.9 (98.1 - 99.3)	98.2 (97.3 - 98.8)	97.6 (96.5 - 98.3)	96.2 (94.8 - 97.2)	94.9 (93.2 - 96.1)	94.2 (92.2 - 95.7)
Cardiovascular diseases	Atrial fibrillation	Female	35-44	2853	NR	98.6 (98.1 - 99.0)	97.1 (96.4 - 97.7)	95.8 (95.0 - 96.5)	93.6 (92.5 - 94.5)	91.1 (89.7 - 92.2)	87.2 (85.0 - 89.0)
Cardiovascular diseases	Atrial fibrillation	Female	45-54	9200	NR	97.6 (97.3 - 97.9)	95.5 (95.1 - 96.0)	93.8 (93.3 - 94.3)	89.2 (88.4 - 89.9)	83.4 (82.4 - 84.4)	76.2 (74.7 - 77.7)
Cardiovascular diseases	Atrial fibrillation	Female	55-64	29781	21.45 (21.7 - 21.08)	95.4 (95.1 - 95.6)	91.8 (91.5 - 92.1)	88.5 (88.2 - 88.9)	79.8 (79.3 - 80.4)	68.6 (67.9 - 69.3)	54.8 (53.8 - 55.8)
Cardiovascular diseases	Atrial fibrillation	Female	65-74	77967	12.92 (13.03 - 12.82)	92.0 (91.8 - 92.2)	85.4 (85.2 - 85.7)	79.2 (78.9 - 79.5)	61.9 (61.5 - 62.3)	41.1 (40.6 - 41.6)	20.2 (19.6 - 20.8)
Cardiovascular diseases	Atrial fibrillation	Female	75-84	135251	6.2 (6.24 - 6.15)	84.8 (84.6 - 85.0)	71.2 (71.0 - 71.5)	57.9 (57.6 - 58.1)	27.6 (27.3 - 27.8)	8.6 (8.3 - 8.8)	1.6 (1.4 - 1.7)
Cardiovascular diseases	Atrial fibrillation	Female	85-95	102900	2.37 (2.4 - 2.35)	67.4 (67.1 - 67.7)	43.1 (42.8 - 43.4)	25.2 (24.9 - 25.5)	4.2 (4.1 - 4.4)	0.4 (0.3 - 0.4)	0.1 (0.0 - 0.1)
Cardiovascular diseases	Cardiomyopathy	Female	All	30040	13.31 (13.58 - 13.0)	90.4 (90.1 - 90.8)	82.0 (81.6 - 82.5)	74.6 (74.1 - 75.1)	58.8 (58.1 - 59.5)	45.8 (44.9 - 46.6)	36.3 (35.3 - 37.4)
Cardiovascular diseases	Cardiomyopathy	Female	16-24	1067	NR	98.9 (98.0 - 99.4)	98.3 (97.3 - 98.9)	97.8 (96.7 - 98.6)	96.3 (94.7 - 97.5)	94.8 (92.6 - 96.4)	92.5 (88.6 - 95.1)
Cardiovascular diseases	Cardiomyopathy	Female	25-34	1314	NR	98.7 (97.9 - 99.2)	97.9 (96.9 - 98.6)	97.1 (96.0 - 98.0)	95.4 (93.8 - 96.5)	93.2 (91.1 - 94.9)	92.0 (89.3 - 94.0)
Cardiovascular diseases	Cardiomyopathy	Female	35-44	1715	NR	97.4 (96.5 - 98.1)	96.0 (94.9 - 96.8)	94.6 (93.4 - 95.6)	91.4 (89.8 - 92.8)	87.0 (84.7 - 88.9)	81.1 (77.6 - 84.0)
Cardiovascular diseases	Cardiomyopathy	Female	45-54	3106	22.94	96.3 (95.6 - 96.9)	93.3 (92.3 - 94.1)	91.1 (90.0 - 92.1)	84.1 (82.5 - 85.6)	74.3 (72.0 - 76.4)	65.3 (62.1 - 68.3)
Cardiovascular diseases	Cardiomyopathy	Female	55-64	5428	19.78 (20.63 - 18.88)	95.1 (94.5 - 95.6)	90.6 (89.8 - 91.4)	86.7 (85.7 - 87.6)	77.0 (75.6 - 78.3)	63.2 (61.2 - 65.0)	49.7 (47.1 - 52.3)
Cardiovascular diseases	Cardiomyopathy	Female	65-74	7567	11.9 (12.38 - 11.56)	92.6 (91.9 - 93.1)	84.9 (84.1 - 85.8)	77.8 (76.8 - 78.8)	58.0 (56.5 - 59.4)	37.3 (35.5 - 39.2)	19.1 (16.9 - 21.5)
Cardiovascular diseases	Cardiomyopathy	Female	75-84	7057	6.38 (6.59 - 6.16)	86.1 (85.3 - 86.9)	72.6 (71.4 - 73.6)	59.1 (57.8 - 60.4)	29.8 (28.3 - 31.2)	10.5 (9.2 - 11.8)	2.4 (1.4 - 3.8)
Cardiovascular diseases	Cardiomyopathy	Female	85-95	2702	2.65 (2.82 - 2.46)	69.2 (67.4 - 70.9)	46.0 (44.0 - 48.0)	28.0 (26.2 - 30.0)	4.9 (3.9 - 6.2)	0.6 (0.2 - 1.5)	0.0 (0.0 - 0.0)
Cardiovascular diseases	Chronic ischemic heart disease	Female	All	217230	7.45 (7.5 - 7.39)	84.1 (84.0 - 84.3)	71.5 (71.3 - 71.7)	61.0 (60.8 - 61.2)	40.5 (40.3 - 40.8)	26.2 (25.9 - 26.4)	16.4 (16.1 - 16.6)
Cardiovascular diseases	Chronic ischemic heart disease	Female	16-24	64	NR	95.3 (86.1 - 98.5)	91.9 (81.5 - 96.5)	91.9 (81.5 - 96.5)	89.9 (78.7 - 95.3)	89.9 (78.7 - 95.3)	89.9 (78.7 - 95.3)
Cardiovascular diseases	Chronic ischemic heart disease	Female	25-34	327	NR	97.8 (95.5 - 99.0)	95.8 (92.9 - 97.5)	95.8 (92.9 - 97.5)	92.0 (87.8 - 94.8)	87.5 (82.1 - 91.4)	82.6 (75.0 - 88.1)
Cardiovascular diseases	Chronic ischemic heart disease	Female	35-44	2085	NR	98.2 (97.6 - 98.7)	97.1 (96.2 - 97.7)	95.3 (94.2 - 96.1)	91.7 (90.3 - 92.9)	86.9 (85.0 - 88.6)	80.2 (77.4 - 82.7)
Cardiovascular diseases	Chronic ischemic heart disease	Female	45-54	9813	NR	98.1 (97.8 - 98.3)	96.1 (95.7 - 96.5)	94.2 (93.7 - 94.6)	88.3 (87.6 - 89.0)	80.6 (79.6 - 81.5)	70.6 (69.1 - 72.0)
Cardiovascular diseases	Chronic ischemic heart disease	Female	55-64	26665	20.07 (20.37 - 19.77)	96.7 (96.4 - 96.9)	93.1 (92.8 - 93.4)	89.6 (89.2 - 90.0)	79.3 (78.8 - 79.9)	66.6 (65.9 - 67.3)	50.3 (49.3 - 51.3)
Cardiovascular diseases	Chronic ischemic heart disease	Female	65-74	51723	12.83 (12.96 - 12.7)	93.3 (93.1 - 93.5)	86.7 (86.4 - 87.0)	80.2 (79.9 - 80.6)	61.8 (61.3 - 62.3)	40.8 (40.2 - 41.3)	20.4 (19.8 - 21.1)
Cardiovascular diseases	Chronic ischemic heart disease	Female	75-84	72962	6.36 (6.42 - 6.29)	84.7 (84.4 - 85.0)	71.2 (70.8 - 71.5)	58.4 (58.1 - 58.8)	29.4 (29.0 - 29.8)	9.8 (9.5 - 10.1)	2.1 (1.9 - 2.3)
Cardiovascular diseases	Chronic ischemic heart disease	Female	85-95	50390	2.27 (2.31 - 2.23)	66.3 (65.9 - 66.7)	42.1 (41.7 - 42.6)	25.3 (24.9 - 25.7)	4.9 (4.6 - 5.1)	0.5 (0.4 - 0.6)	0.1 (0.0 - 0.1)
Cardiovascular diseases	Endocarditis	Female	All	8409	4.44 (4.66 - 4.24)	70.1 (69.1 - 71.1)	57.3 (56.2 - 58.4)	47.5 (46.4 - 48.6)	30.9 (29.8 - 32.0)	21.9 (20.8 - 23.1)	16.7 (15.4 - 18.0)
Cardiovascular diseases	Endocarditis	Female	16-24	140	NR	95.7 (90.7 - 98.0)	95.0 (89.8 - 97.6)	92.6 (86.6 - 95.9)	87.3 (79.7 - 92.2)	80.3 (69.6 - 87.5)	80.3 (69.6 - 87.5)
Cardiovascular diseases	Endocarditis	Female	25-34	311	NR	94.5 (91.3 - 96.5)	90.9 (87.1 - 93.7)	86.9 (82.4 - 90.4)	81.7 (76.2 - 86.1)	79.8 (73.6 - 84.6)	76.0 (67.7 - 82.5)
Cardiovascular diseases	Endocarditis	Female	35-44	352	NR	91.4 (87.9 - 93.9)	87.3 (83.2 - 90.4)	83.7 (79.2 - 87.3)	76.9 (71.5 - 81.4)	72.6 (66.5 - 77.7)	66.7 (58.8 - 73.3)
Cardiovascular diseases	Endocarditis	Female	45-54	555	19.59	85.4 (82.2 - 88.1)	79.0 (75.3 - 82.2)	73.9 (69.8 - 77.4)	64.8 (60.3 - 69.0)	56.2 (50.9 - 61.1)	48.3 (41.8 - 54.5)
Cardiovascular diseases	Endocarditis	Female	55-64	929	11.81 (13.72 - 9.72)	80.6 (77.9 - 83.0)	71.8 (68.7 - 74.6)	65.8 (62.6 - 68.9)	52.6 (48.9 - 56.1)	43.6 (39.6 - 47.5)	32.4 (27.6 - 37.3)
Cardiovascular diseases	Endocarditis	Female	65-74	1706	6.71 (7.53 - 6.1)	74.3 (72.1 - 76.3)	64.6 (62.3 - 66.9)	56.7 (54.2 - 59.1)	36.4 (33.7 - 39.1)	22.3 (19.6 - 25.1)	10.8 (8.0 - 14.0)
Cardiovascular diseases	Endocarditis	Female	75-84	2713	3.09 (3.34 - 2.83)	66.2 (64.3 - 67.9)	51.0 (49.0 - 52.9)	38.0 (36.0 - 39.9)	14.7 (13.1 - 16.4)	3.7 (2.7 - 4.9)	1.7 (1.0 - 2.9)
Cardiovascular diseases	Endocarditis	Female	85-95	1639	1.06 (1.22 - 0.85)	50.6 (48.1 - 53.0)	29.9 (27.6 - 32.2)	15.8 (13.9 - 17.7)	2.6 (1.7 - 3.7)	0.2 (0.0 - 0.8)	0.1 (0.0 - 0.6)
Cardiovascular diseases	Heart failure	Female	All	338102	3.27 (3.3 - 3.25)	71.2 (71.1 - 71.4)	52.1 (51.9 - 52.3)	38.2 (38.0 - 38.3)	18.1 (17.9 - 18.3)	9.1 (8.9 - 9.2)	4.7 (4.6 - 4.9)
Cardiovascular diseases	Heart failure	Female	16-24	428	NR	91.4 (88.3 - 93.7)	88.6 (85.1 - 91.4)	85.8 (81.9 - 89.0)	80.9 (76.2 - 84.7)	77.1 (71.4 - 81.8)	75.7 (69.3 - 80.9)
Cardiovascular diseases	Heart failure	Female	25-34	1232	NR	93.8 (92.3 - 95.1)	90.7 (88.8 - 92.2)	89.3 (87.3 - 90.9)	84.8 (82.4 - 87.0)	82.3 (79.5 - 84.8)	77.9 (73.9 - 81.3)

Appendix Table A7: Unadjusted Median Survival and Survival Probabilities by Age, females (Page 7 of 18)

Group	Diagnosis Category	Sex	Age	N	Median Survival (y)	1y (%)	3y (%)	5y (%)	10y (%)	15y (%)	20y (%)
Cardiovascular diseases	Heart failure	Female	35-44	2279	NR	92.6 (91.4 - 93.6)	88.7 (87.3 - 89.9)	85.4 (83.9 - 86.9)	79.7 (77.8 - 81.5)	72.9 (70.4 - 75.1)	65.4 (62.2 - 68.3)
Cardiovascular diseases	Heart failure	Female	45-54	6536	18.86 (19.72 - 17.91)	90.4 (89.6 - 91.1)	84.6 (83.7 - 85.5)	79.5 (78.4 - 80.5)	69.2 (67.9 - 70.4)	57.6 (56.0 - 59.2)	47.3 (45.2 - 49.3)
Cardiovascular diseases	Heart failure	Female	55-64	18917	11.96 (12.24 - 11.68)	87.1 (86.6 - 87.6)	78.1 (77.4 - 78.7)	70.8 (70.1 - 71.5)	55.7 (54.9 - 56.5)	41.8 (40.9 - 42.7)	27.9 (26.8 - 29.1)
Cardiovascular diseases	Heart failure	Female	65-74	52884	7.37 (7.47 - 7.26)	82.9 (82.6 - 83.2)	70.9 (70.5 - 71.3)	61.0 (60.5 - 61.4)	38.9 (38.4 - 39.4)	21.4 (21.0 - 21.9)	8.8 (8.3 - 9.3)
Cardiovascular diseases	Heart failure	Female	75-84	121890	4.0 (4.04 - 3.96)	75.6 (75.4 - 75.9)	57.8 (57.5 - 58.1)	42.6 (42.3 - 42.9)	16.2 (16.0 - 16.5)	4.2 (4.0 - 4.4)	0.8 (0.7 - 0.9)
Cardiovascular diseases	Heart failure	Female	85-95	124474	1.69 (1.71 - 1.67)	60.0 (59.7 - 60.3)	34.7 (34.4 - 34.9)	18.2 (18.0 - 18.4)	2.5 (2.4 - 2.6)	0.2 (0.2 - 0.2)	0.0 (0.0 - 0.1)
Cardiovascular diseases	Hypertension	Female	All	1017737	12.74 (12.78 - 12.71)	92.4 (92.3 - 92.4)	84.5 (84.5 - 84.6)	76.8 (76.7 - 76.9)	58.8 (58.7 - 58.9)	43.5 (43.3 - 43.6)	31.3 (31.1 - 31.5)
Cardiovascular diseases	Hypertension	Female	16-24	3064	NR	99.4 (99.0 - 99.6)	99.1 (98.7 - 99.4)	98.8 (98.3 - 99.1)	98.1 (97.4 - 98.6)	97.1 (96.1 - 97.8)	96.0 (94.7 - 97.0)
Cardiovascular diseases	Hypertension	Female	25-34	11336	NR	99.7 (99.5 - 99.8)	99.2 (99.0 - 99.3)	98.8 (98.5 - 99.0)	97.8 (97.4 - 98.1)	96.0 (95.4 - 96.5)	93.8 (92.9 - 94.6)
Cardiovascular diseases	Hypertension	Female	35-44	29667	NR	99.4 (99.3 - 99.5)	98.7 (98.5 - 98.8)	97.9 (97.8 - 98.1)	96.0 (95.8 - 96.3)	93.3 (92.9 - 93.6)	89.1 (88.4 - 89.8)
Cardiovascular diseases	Hypertension	Female	45-54	90330	NR	98.9 (98.9 - 99.0)	97.8 (97.7 - 97.8)	96.6 (96.5 - 96.7)	93.3 (93.1 - 93.5)	88.2 (87.9 - 88.5)	80.7 (80.2 - 81.2)
Cardiovascular diseases	Hypertension	Female	55-64	181854	NR	97.8 (97.7 - 97.8)	95.4 (95.3 - 95.5)	93.1 (93.0 - 93.2)	86.3 (86.1 - 86.5)	76.7 (76.4 - 76.9)	62.9 (62.5 - 63.4)
Cardiovascular diseases	Hypertension	Female	65-74	269059	15.34 (15.4 - 15.27)	95.5 (95.4 - 95.6)	90.9 (90.8 - 91.1)	86.3 (86.2 - 86.4)	71.7 (71.5 - 71.9)	51.5 (51.3 - 51.8)	28.2 (27.8 - 28.6)
Cardiovascular diseases	Hypertension	Female	75-84	281544	8.26 (8.29 - 8.22)	90.7 (90.6 - 90.8)	80.7 (80.5 - 80.8)	69.6 (69.5 - 69.8)	39.5 (39.3 - 39.8)	15.2 (15.0 - 15.4)	3.3 (3.1 - 3.4)
Cardiovascular diseases	Hypertension	Female	85-95	143666	3.65 (3.68 - 3.62)	78.3 (78.1 - 78.5)	56.7 (56.4 - 56.9)	37.6 (37.3 - 37.8)	8.8 (8.6 - 9.0)	1.0 (0.9 - 1.1)	0.1 (0.1 - 0.2)
Cardiovascular diseases	Hypertensive heart disease	Female	All	30921	7.34 (7.51 - 7.2)	85.4 (85.0 - 85.8)	72.1 (71.6 - 72.6)	60.9 (60.3 - 61.4)	40.0 (39.4 - 40.6)	25.7 (25.2 - 26.3)	16.3 (15.7 - 16.9)
Cardiovascular diseases	Hypertensive heart disease	Female	16-24	27	NR	96.3 (76.5 - 99.5)	96.3 (76.5 - 99.5)	92.3 (72.5 - 98.0)	92.3 (72.5 - 98.0)	92.3 (72.5 - 98.0)	92.3 (72.5 - 98.0)
Cardiovascular diseases	Hypertensive heart disease	Female	25-34	106	NR	98.1 (92.7 - 99.5)	98.1 (92.7 - 99.5)	98.1 (92.7 - 99.5)	98.1 (92.7 - 99.5)	92.3 (83.3 - 96.5)	92.3 (83.3 - 96.5)
Cardiovascular diseases	Hypertensive heart disease	Female	35-44	331	NR	99.1 (97.2 - 99.7)	96.8 (94.2 - 98.3)	94.8 (91.7 - 96.8)	91.1 (87.2 - 93.9)	85.2 (80.1 - 89.0)	77.5 (70.8 - 82.9)
Cardiovascular diseases	Hypertensive heart disease	Female	45-54	1339	NR	98.1 (97.2 - 98.7)	95.8 (94.5 - 96.7)	94.4 (93.0 - 95.5)	87.0 (85.0 - 88.8)	80.9 (78.5 - 83.1)	70.6 (67.3 - 73.7)
Cardiovascular diseases	Hypertensive heart disease	Female	55-64	3748	20.46 (21.27 - 19.76)	96.5 (95.9 - 97.1)	92.4 (91.5 - 93.2)	88.2 (87.1 - 89.2)	77.7 (76.2 - 79.0)	66.2 (64.5 - 67.8)	51.7 (49.6 - 53.8)
Cardiovascular diseases	Hypertensive heart disease	Female	65-74	7454	12.29 (12.64 - 11.97)	93.4 (92.8 - 93.9)	86.2 (85.4 - 87.0)	79.4 (78.4 - 80.3)	60.2 (59.0 - 61.4)	38.3 (37.0 - 39.5)	18.6 (17.3 - 20.0)
Cardiovascular diseases	Hypertensive heart disease	Female	75-84	11182	5.98 (6.12 - 5.83)	85.7 (85.1 - 86.4)	71.0 (70.1 - 71.8)	56.4 (55.5 - 57.4)	26.8 (25.9 - 27.7)	8.4 (7.8 - 9.0)	1.4 (1.1 - 1.9)
Cardiovascular diseases	Hypertensive heart disease	Female	85-95	6439	2.24 (2.35 - 2.14)	67.4 (66.2 - 68.5)	41.5 (40.2 - 42.7)	23.8 (22.8 - 24.9)	4.5 (3.9 - 5.0)	0.4 (0.2 - 0.6)	0.1 (0.0 - 0.3)
Cardiovascular diseases	Intracerebral hem	Female	All	42614	3.46 (3.56 - 3.37)	63.3 (62.9 - 63.8)	52.4 (51.9 - 52.9)	43.2 (42.7 - 43.7)	27.4 (27.0 - 27.9)	18.9 (18.4 - 19.4)	13.8 (13.2 - 14.3)
Cardiovascular diseases	Intracerebral hem	Female	16-24	339	NR	91.4 (87.9 - 94.0)	89.9 (86.1 - 92.6)	89.5 (85.7 - 92.4)	88.1 (83.9 - 91.2)	86.0 (81.2 - 89.7)	86.0 (81.2 - 89.7)
Cardiovascular diseases	Intracerebral hem	Female	25-34	679	NR	93.3 (91.2 - 95.0)	92.0 (89.7 - 93.8)	91.1 (88.6 - 93.0)	89.0 (86.2 - 91.3)	86.5 (83.1 - 89.3)	85.2 (81.2 - 88.4)
Cardiovascular diseases	Intracerebral hem	Female	35-44	1032	NR	85.5 (83.2 - 87.5)	83.6 (81.2 - 85.7)	82.3 (79.8 - 84.5)	79.4 (76.7 - 81.9)	75.1 (71.8 - 78.0)	70.3 (66.2 - 74.1)
Cardiovascular diseases	Intracerebral hem	Female	45-54	2605	NR	84.2 (82.7 - 85.5)	81.0 (79.5 - 82.5)	78.8 (77.1 - 80.3)	73.3 (71.5 - 75.1)	67.0 (64.8 - 69.1)	60.0 (57.1 - 62.8)
Cardiovascular diseases	Intracerebral hem	Female	55-64	4875	14.38 (15.16 - 13.49)	78.6 (77.4 - 79.7)	74.1 (72.8 - 75.3)	69.8 (68.4 - 71.1)	59.3 (57.8 - 60.8)	48.7 (46.9 - 50.4)	35.0 (32.7 - 37.3)
Cardiovascular diseases	Intracerebral hem	Female	65-74	8311	6.68 (7.0 - 6.32)	71.4 (70.4 - 72.4)	63.3 (62.2 - 64.3)	56.1 (55.0 - 57.2)	37.4 (36.2 - 38.6)	21.3 (20.1 - 22.6)	10.2 (9.0 - 11.6)
Cardiovascular diseases	Intracerebral hem	Female	75-84	13887	2.53 (2.66 - 2.38)	59.7 (58.9 - 60.5)	47.1 (46.2 - 47.9)	35.2 (34.4 - 36.0)	13.4 (12.7 - 14.1)	3.3 (2.9 - 3.8)	0.8 (0.5 - 1.1)
Cardiovascular diseases	Intracerebral hem	Female	85-95	10341	0.61 (0.68 - 0.53)	45.8 (44.8 - 46.7)	28.4 (27.5 - 29.3)	15.4 (14.7 - 16.2)	1.8 (1.5 - 2.2)	0.1 (0.1 - 0.3)	0.1 (0.1 - 0.3)
Cardiovascular diseases	Ischemic heart disease	Female	All	339927	8.33 (8.38 - 8.28)	83.4 (83.2 - 83.5)	72.5 (72.3 - 72.6)	63.1 (63.0 - 63.3)	44.5 (44.3 - 44.6)	30.6 (30.4 - 30.8)	20.4 (20.2 - 20.6)
Cardiovascular diseases	Ischemic heart disease	Female	16-24	1033	NR	99.5 (98.8 - 99.8)	99.2 (98.3 - 99.6)	98.8 (97.8 - 99.3)	98.1 (96.9 - 98.9)	97.5 (95.3 - 98.7)	97.5 (95.3 - 98.7)
Cardiovascular diseases	Ischemic heart disease	Female	25-34	3647	NR	99.4 (99.0 - 99.6)	99.1 (98.7 - 99.4)	99.1 (98.7 - 99.4)	97.7 (96.8 - 98.4)	96.1 (94.6 - 97.2)	93.7 (90.8 - 95.7)
Cardiovascular diseases	Ischemic heart disease	Female	35-44	5457	NR	98.4 (98.0 - 98.7)	97.6 (97.2 - 98.0)	96.6 (96.1 - 97.1)	94.3 (93.5 - 94.9)	90.7 (89.6 - 91.6)	85.2 (83.5 - 86.7)
Cardiovascular diseases	Ischemic heart disease	Female	45-54	19080	NR	97.7 (97.5 - 97.9)	96.1 (95.8 - 96.3)	94.7 (94.4 - 95.1)	90.6 (90.2 - 91.1)	85.1 (84.5 - 85.7)	77.1 (76.2 - 78.0)
Cardiovascular diseases	Ischemic heart disease	Female	55-64	45255	22.62 (22.94 - 22.3)	96.2 (96.0 - 96.4)	93.5 (93.2 - 93.7)	90.7 (90.4 - 91.0)	82.8 (82.5 - 83.2)	72.7 (72.2 - 73.2)	58.5 (57.8 - 59.2)
Cardiovascular diseases	Ischemic heart disease	Female	65-74	77951	13.99 (14.09 - 13.9)	92.4 (92.2 - 92.6)	86.9 (86.7 - 87.1)	81.5 (81.2 - 81.8)	65.5 (65.2 - 65.9)	45.8 (45.3 - 46.2)	24.5 (24.0 - 25.0)
Cardiovascular diseases	Ischemic heart disease	Female	75-84	107584	6.47 (6.53 - 6.42)	82.7 (82.4 - 82.9)	70.4 (70.1 - 70.7)	58.5 (58.2 - 58.8)	30.9 (30.6 - 31.2)	11.2 (11.0 - 11.4)	2.4 (2.3 - 2.6)
Cardiovascular diseases	Ischemic heart disease	Female	85-95	75305	2.2 (2.23 - 2.17)	63.9 (63.6 - 64.3)	42.0 (41.7 - 42.4)	25.9 (25.6 - 26.3)	5.3 (5.1 - 5.5)	0.6 (0.5 - 0.7)	0.1 (0.1 - 0.1)
Cardiovascular diseases	Ischemic stroke	Female	All	324516	7.36 (7.41 - 7.32)	82.6 (82.5 - 82.8)	70.9 (70.8 - 71.1)	60.5 (60.3 - 60.6)	40.4 (40.2 - 40.6)	26.9 (26.7 - 27.1)	17.7 (17.5 - 18.0)
Cardiovascular diseases	Ischemic stroke	Female	16-24	949	NR	96.8 (95.5 - 97.8)	96.6 (95.2 - 97.6)	95.9 (94.4 - 97.0)	95.4 (93.8 - 96.6)	94.9 (93.1 - 96.3)	94.5 (92.4 - 96.0)
Cardiovascular diseases	Ischemic stroke	Female	25-34	2466	NR	97.3 (96.6 - 97.9)	96.5 (95.7 - 97.2)	95.9 (95.0 - 96.6)	94.3 (93.2 - 95.3)	92.9 (91.5 - 94.1)	92.0 (90.3 - 93.3)
Cardiovascular diseases	Ischemic stroke	Female	35-44	5507	NR	97.4 (96.9 - 97.8)	96.3 (95.8 - 96.8)	95.7 (95.1 - 96.2)	93.3 (92.5 - 94.0)	90.2 (89.1 - 91.2)	85.2 (83.4 - 86.8)
Cardiovascular diseases	Ischemic stroke	Female	45-54	15035	NR	96.8 (96.5 - 97.1)	95.1 (94.7 - 95.4)	93.5 (93.0 - 93.9)	88.7 (88.1 - 89.3)	82.6 (81.8 - 83.4)	75.0 (73.8 - 76.2)
Cardiovascular diseases	Ischemic stroke	Female	55-64	36868	21.44 (21.78 - 21.1)	95.3 (95.1 - 95.5)	92.2 (91.9 - 92.5)	89.2 (88.9 - 89.5)	80.7 (80.3 - 81.2)	69.6 (69.0 - 70.2)	54.9 (54.0 - 55.8)
Cardiovascular diseases	Ischemic stroke	Female	65-74	72414	13.13 (13.25 - 13.03)	92.0 (91.8 - 92.2)	86.2 (85.9 - 86.5)	80.3 (80.0 - 80.6)	62.7 (62.2 - 63.1)	42.1 (41.6 - 42.6)	22.1 (21.4 - 22.7)
Cardiovascular diseases	Ischemic stroke	Female	75-84	109263	6.13 (6.18 - 6.08)	83.3 (83.1 - 83.5)	70.4 (70.1 - 70.7)	57.3 (57.0 - 57.6)	28.3 (28.0 - 28.6)	9.8 (9.5 - 10.0)	2.2 (2.0 - 2.4)

Appendix Table A7: Unadjusted Median Survival and Survival Probabilities by Age, females (Page 8 of 18)

Group	Diagnosis Category	Sex	Age	N	Median Survival (y)	1y (%)	3y (%)	5y (%)	10y (%)	15y (%)	20y (%)
Cardiovascular diseases	Ischemic stroke	Female	85-95	77635	2.28 (2.32 - 2.25)	64.8 (64.4 - 65.1)	42.8 (42.4 - 43.1)	25.6 (25.3 - 26.0)	5.0 (4.8 - 5.2)	0.6 (0.5 - 0.7)	0.1 (0.0 - 0.1)
Cardiovascular diseases	Nonrheum valv diseases	Female	All	127061	7.86 (7.94 - 7.79)	87.5 (87.4 - 87.7)	74.5 (74.3 - 74.8)	63.4 (63.1 - 63.7)	41.7 (41.4 - 42.0)	27.4 (27.1 - 27.8)	18.1 (17.7 - 18.5)
Cardiovascular diseases	Nonrheum valv diseases	Female	16-24	1424	NR	99.6 (99.1 - 99.8)	99.2 (98.5 - 99.5)	98.9 (98.2 - 99.4)	97.8 (96.7 - 98.6)	96.5 (94.9 - 97.6)	96.2 (94.4 - 97.4)
Cardiovascular diseases	Nonrheum valv diseases	Female	25-34	2023	NR	99.4 (99.0 - 99.7)	98.9 (98.3 - 99.2)	98.3 (97.6 - 98.8)	97.5 (96.5 - 98.1)	97.2 (96.2 - 97.9)	95.2 (93.3 - 96.6)
Cardiovascular diseases	Nonrheum valv diseases	Female	35-44	2707	NR	99.2 (98.7 - 99.5)	97.7 (97.1 - 98.3)	96.5 (95.7 - 97.2)	94.0 (92.9 - 94.9)	91.1 (89.6 - 92.4)	84.9 (82.4 - 87.0)
Cardiovascular diseases	Nonrheum valv diseases	Female	45-54	4857	NR	98.3 (97.9 - 98.6)	95.7 (95.1 - 96.3)	93.8 (93.0 - 94.4)	88.1 (87.0 - 89.1)	81.4 (79.9 - 82.8)	73.5 (71.4 - 75.5)
Cardiovascular diseases	Nonrheum valv diseases	Female	55-64	11689	20.76 (21.25 - 20.14)	96.8 (96.4 - 97.1)	92.9 (92.4 - 93.4)	89.4 (88.8 - 90.0)	79.7 (78.8 - 80.5)	68.7 (67.6 - 69.9)	52.2 (50.5 - 53.9)
Cardiovascular diseases	Nonrheum valv diseases	Female	65-74	28437	12.87 (13.05 - 12.69)	94.4 (94.1 - 94.6)	88.1 (87.7 - 88.5)	81.5 (81.0 - 82.0)	62.5 (61.8 - 63.2)	40.2 (39.3 - 41.0)	18.6 (17.6 - 19.6)
Cardiovascular diseases	Nonrheum valv diseases	Female	75-84	46968	6.66 (6.73 - 6.59)	88.7 (88.4 - 89.0)	75.1 (74.7 - 75.5)	61.3 (60.8 - 61.8)	29.3 (28.8 - 29.8)	9.3 (8.9 - 9.7)	1.7 (1.5 - 2.0)
Cardiovascular diseases	Nonrheum valv diseases	Female	85-95	27695	2.6 (2.65 - 2.55)	71.8 (71.3 - 72.4)	45.0 (44.4 - 45.6)	26.0 (25.5 - 26.6)	4.6 (4.2 - 4.9)	0.3 (0.2 - 0.5)	0.1 (0.0 - 0.2)
Cardiovascular diseases	PAH	Female	All	7861	2.42 (2.53 - 2.32)	67.2 (66.2 - 68.3)	44.5 (43.3 - 45.6)	31.5 (30.4 - 32.6)	16.9 (15.9 - 18.0)	10.1 (9.1 - 11.2)	6.5 (5.5 - 7.6)
Cardiovascular diseases	PAH	Female	16-24	49	NR	85.6 (72.2 - 92.9)	83.4 (69.4 - 91.3)	83.4 (69.4 - 91.3)	79.6 (63.6 - 89.1)	79.6 (63.6 - 89.1)	58.0 (27.5 - 79.5)
Cardiovascular diseases	PAH	Female	25-34	105	NR	96.2 (90.1 - 98.5)	94.0 (87.2 - 97.3)	88.8 (80.1 - 93.9)	83.4 (72.5 - 90.2)	72.9 (58.4 - 83.1)	61.2 (40.6 - 76.6)
Cardiovascular diseases	PAH	Female	35-44	128	NR	94.4 (88.7 - 97.3)	89.9 (82.9 - 94.2)	84.0 (75.7 - 89.7)	72.3 (62.1 - 80.2)	67.2 (56.1 - 76.1)	59.3 (44.4 - 71.4)
Cardiovascular diseases	PAH	Female	45-54	245	11.56 (16.55 - 7.21)	85.0 (79.8 - 88.9)	74.9 (68.7 - 80.0)	65.3 (58.4 - 71.3)	52.8 (45.2 - 59.8)	43.4 (35.2 - 51.2)	35.6 (26.5 - 44.8)
Cardiovascular diseases	PAH	Female	55-64	606	4.81 (5.95 - 3.96)	78.9 (75.4 - 82.0)	60.4 (56.2 - 64.4)	48.6 (44.2 - 52.8)	36.8 (32.3 - 41.2)	24.0 (19.4 - 29.0)	15.2 (10.3 - 21.1)
Cardiovascular diseases	PAH	Female	65-74	1612	3.38 (3.74 - 3.09)	74.4 (72.2 - 76.5)	53.2 (50.6 - 55.7)	39.6 (36.9 - 42.2)	21.5 (19.0 - 24.1)	10.7 (8.4 - 13.3)	3.9 (2.0 - 6.7)
Cardiovascular diseases	PAH	Female	75-84	3128	2.29 (2.43 - 2.12)	66.2 (64.4 - 67.8)	42.5 (40.7 - 44.4)	28.5 (26.8 - 30.3)	10.2 (8.7 - 11.7)	2.9 (2.0 - 4.2)	0.5 (0.1 - 1.5)
Cardiovascular diseases	PAH	Female	85-95	1928	1.15 (1.3 - 1.08)	54.0 (51.7 - 56.2)	24.8 (22.7 - 26.9)	11.4 (9.8 - 13.1)	1.3 (0.7 - 2.3)	0.0 (0.0 - 0.0)	0.0 (0.0 - 0.0)
Cardiovascular diseases	Peripheral artery	Female	All	94667	6.64 (6.72 - 6.55)	81.8 (81.6 - 82.1)	68.3 (68.0 - 68.6)	57.5 (57.2 - 57.9)	37.2 (36.9 - 37.6)	24.0 (23.6 - 24.3)	15.5 (15.1 - 15.9)
Cardiovascular diseases	Peripheral artery	Female	16-24	1386	NR	99.9 (99.5 - 100.0)	99.9 (99.5 - 100.0)	99.8 (99.4 - 100.0)	99.5 (98.9 - 99.8)	99.5 (98.9 - 99.8)	98.9 (97.4 - 99.6)
Cardiovascular diseases	Peripheral artery	Female	25-34	1663	NR	99.6 (99.1 - 99.8)	99.1 (98.5 - 99.5)	98.5 (97.7 - 99.0)	97.7 (96.7 - 98.4)	97.3 (96.1 - 98.1)	95.5 (93.1 - 97.0)
Cardiovascular diseases	Peripheral artery	Female	35-44	2748	NR	99.2 (98.7 - 99.5)	98.0 (97.3 - 98.4)	97.0 (96.2 - 97.6)	94.6 (93.5 - 95.5)	91.7 (90.2 - 93.0)	87.3 (84.8 - 89.3)
Cardiovascular diseases	Peripheral artery	Female	45-54	4584	NR	98.0 (97.6 - 98.4)	95.4 (94.7 - 96.0)	92.8 (91.9 - 93.5)	85.3 (84.1 - 86.4)	76.6 (74.9 - 78.1)	66.3 (64.0 - 68.4)
Cardiovascular diseases	Peripheral artery	Female	55-64	9938	16.35 (16.8 - 15.94)	95.1 (94.7 - 95.5)	89.6 (89.0 - 90.2)	84.3 (83.5 - 85.0)	70.2 (69.2 - 71.2)	54.5 (53.3 - 55.7)	37.6 (36.1 - 39.1)
Cardiovascular diseases	Peripheral artery	Female	65-74	21870	10.01 (10.18 - 9.85)	91.5 (91.1 - 91.9)	82.3 (81.8 - 82.8)	73.6 (73.0 - 74.2)	50.1 (49.3 - 50.9)	28.8 (27.9 - 29.6)	12.0 (11.2 - 12.9)
Cardiovascular diseases	Peripheral artery	Female	75-84	30358	5.26 (5.35 - 5.16)	82.0 (81.5 - 82.4)	65.9 (65.4 - 66.5)	51.8 (51.2 - 52.3)	23.1 (22.5 - 23.7)	6.7 (6.3 - 7.1)	1.0 (0.8 - 1.2)
Cardiovascular diseases	Peripheral artery	Female	85-95	20813	1.73 (1.78 - 1.67)	59.4 (58.7 - 60.1)	35.8 (35.1 - 36.5)	20.5 (19.9 - 21.1)	3.7 (3.4 - 4.0)	0.4 (0.3 - 0.6)	0.0 (0.0 - 0.1)
Cardiovascular diseases	Rheumatic heart disease	Female	All	18087	5.89 (6.05 - 5.72)	81.2 (80.7 - 81.8)	65.8 (65.1 - 66.6)	54.3 (53.5 - 55.1)	36.0 (35.1 - 36.9)	25.1 (24.1 - 26.0)	18.8 (17.8 - 19.8)
Cardiovascular diseases	Rheumatic heart disease	Female	16-24	181	NR	97.8 (94.2 - 99.2)	97.2 (93.4 - 98.8)	96.5 (92.4 - 98.4)	95.6 (91.0 - 97.9)	93.2 (86.9 - 96.6)	93.2 (86.9 - 96.6)
Cardiovascular diseases	Rheumatic heart disease	Female	25-34	443	NR	98.6 (97.0 - 99.4)	96.9 (94.7 - 98.2)	95.6 (93.1 - 97.2)	92.5 (89.1 - 94.9)	92.0 (88.4 - 94.5)	91.1 (86.9 - 94.0)
Cardiovascular diseases	Rheumatic heart disease	Female	35-44	569	NR	98.4 (97.0 - 99.2)	97.6 (96.0 - 98.6)	96.1 (94.1 - 97.5)	93.1 (90.2 - 95.1)	89.0 (85.2 - 91.9)	82.0 (76.2 - 86.5)
Cardiovascular diseases	Rheumatic heart disease	Female	45-54	771	NR	95.5 (93.8 - 96.8)	92.4 (90.2 - 94.1)	88.8 (86.1 - 90.9)	82.7 (79.3 - 85.5)	75.1 (70.8 - 78.9)	67.4 (61.8 - 72.3)
Cardiovascular diseases	Rheumatic heart disease	Female	55-64	1464	17.15 (18.96 - 15.45)	92.4 (90.9 - 93.7)	85.4 (83.4 - 87.2)	80.2 (77.9 - 82.3)	67.7 (64.7 - 70.4)	55.0 (51.4 - 58.4)	42.5 (38.1 - 46.8)
Cardiovascular diseases	Rheumatic heart disease	Female	65-74	3452	9.91 (10.36 - 9.33)	88.4 (87.3 - 89.4)	78.1 (76.6 - 79.5)	68.5 (66.7 - 70.2)	49.6 (47.5 - 51.7)	28.9 (26.4 - 31.4)	12.4 (10.1 - 15.0)
Cardiovascular diseases	Rheumatic heart disease	Female	75-84	6802	5.04 (5.22 - 4.86)	81.8 (80.8 - 82.7)	65.1 (63.8 - 66.3)	50.3 (48.9 - 51.7)	21.7 (20.3 - 23.2)	5.8 (4.8 - 7.0)	1.2 (0.6 - 2.0)
Cardiovascular diseases	Rheumatic heart disease	Female	85-95	4219	1.96 (2.07 - 1.85)	64.3 (62.8 - 65.8)	36.5 (34.9 - 38.1)	19.9 (18.5 - 21.4)	2.9 (2.2 - 3.8)	0.1 (0.0 - 0.8)	0.1 (0.0 - 0.8)
Cardiovascular diseases	Stroke	Female	All	382796	7.19 (7.23 - 7.14)	80.7 (80.6 - 80.8)	69.4 (69.3 - 69.6)	59.4 (59.2 - 59.5)	40.2 (40.0 - 40.3)	27.3 (27.1 - 27.5)	18.6 (18.4 - 18.8)
Cardiovascular diseases	Stroke	Female	16-24	1649	NR	96.3 (95.3 - 97.1)	95.4 (94.2 - 96.3)	94.9 (93.7 - 95.9)	94.2 (92.9 - 95.3)	93.3 (91.7 - 94.5)	92.5 (90.6 - 94.1)
Cardiovascular diseases	Stroke	Female	25-34	3972	NR	96.6 (96.0 - 97.1)	95.8 (95.1 - 96.4)	95.3 (94.5 - 95.9)	93.7 (92.8 - 94.5)	92.2 (91.2 - 93.2)	90.4 (88.8 - 91.7)
Cardiovascular diseases	Stroke	Female	35-44	8052	NR	95.6 (95.1 - 96.0)	94.5 (94.0 - 95.0)	93.8 (93.2 - 94.3)	91.4 (90.7 - 92.1)	88.5 (87.6 - 89.3)	84.2 (82.9 - 85.5)
Cardiovascular diseases	Stroke	Female	45-54	20350	NR	94.6 (94.3 - 94.9)	92.8 (92.4 - 93.2)	91.1 (90.7 - 91.5)	86.7 (86.1 - 87.2)	81.0 (80.3 - 81.6)	73.3 (72.2 - 74.3)
Cardiovascular diseases	Stroke	Female	55-64	44798	21.01 (21.31 - 20.76)	93.1 (92.9 - 93.4)	90.1 (89.8 - 90.4)	87.1 (86.8 - 87.4)	78.6 (78.2 - 79.0)	67.8 (67.3 - 68.4)	53.4 (52.6 - 54.2)
Cardiovascular diseases	Stroke	Female	65-74	83487	12.75 (12.84 - 12.64)	89.9 (89.7 - 90.2)	84.0 (83.8 - 84.3)	78.2 (77.9 - 78.4)	60.7 (60.3 - 61.1)	40.7 (40.2 - 41.1)	21.2 (20.6 - 21.8)
Cardiovascular diseases	Stroke	Female	75-84	125275	5.86 (5.9 - 5.82)	81.0 (80.7 - 81.2)	68.4 (68.1 - 68.6)	55.4 (55.1 - 55.7)	27.0 (26.8 - 27.3)	9.2 (9.0 - 9.5)	2.1 (1.9 - 2.2)
Cardiovascular diseases	Stroke	Female	85-95	90000	2.1 (2.13 - 2.07)	62.4 (62.1 - 62.8)	41.1 (40.8 - 41.4)	24.5 (24.2 - 24.8)	4.7 (4.5 - 4.9)	0.5 (0.5 - 0.6)	0.1 (0.0 - 0.1)
Cardiovascular diseases	Subarachnoid hem	Female	All	34299	9.6 (9.86 - 9.32)	78.9 (78.5 - 79.4)	70.0 (69.5 - 70.5)	62.5 (61.9 - 63.0)	49.1 (48.5 - 49.7)	40.1 (39.4 - 40.8)	32.6 (31.8 - 33.4)
Cardiovascular diseases	Subarachnoid hem	Female	16-24	364	NR	98.1 (96.0 - 99.1)	96.2 (93.6 - 97.8)	96.2 (93.6 - 97.8)	95.9 (93.1 - 97.5)	94.9 (91.7 - 96.9)	93.8 (89.6 - 96.3)
Cardiovascular diseases	Subarachnoid hem	Female	25-34	930	NR	96.8 (95.4 - 97.7)	96.1 (94.6 - 97.1)	95.8 (94.3 - 96.9)	94.6 (92.8 - 96.0)	93.2 (91.0 - 94.9)	90.2 (86.4 - 93.0)
Cardiovascular diseases	Subarachnoid hem	Female	35-44	2040	NR	93.8 (92.7 - 94.8)	92.9 (91.7 - 93.9)	92.1 (90.8 - 93.2)	90.3 (88.9 - 91.6)	88.1 (86.4 - 89.6)	85.3 (83.0 - 87.4)

Appendix Table A7: Unadjusted Median Survival and Survival Probabilities by Age, females (Page 9 of 18)

Group	Diagnosis Category	Sex	Age	N	Median Survival (y)	1y (%)	3y (%)	5y (%)	10y (%)	15y (%)	20y (%)
Cardiovascular diseases	Subarachnoid hem	Female	45-54	4183	NR	91.8 (90.9 - 92.5)	89.9 (89.0 - 90.8)	88.3 (87.3 - 89.2)	84.5 (83.3 - 85.7)	79.4 (77.9 - 80.8)	71.5 (69.3 - 73.6)
Cardiovascular diseases	Subarachnoid hem	Female	55-64	5912	19.38 (19.98 - 18.62)	87.7 (86.9 - 88.5)	84.5 (83.6 - 85.4)	81.4 (80.3 - 82.4)	72.4 (71.1 - 73.6)	61.6 (60.0 - 63.2)	47.6 (45.4 - 49.8)
Cardiovascular diseases	Subarachnoid hem	Female	65-74	7192	10.54 (11.0 - 10.2)	82.3 (81.4 - 83.2)	75.7 (74.6 - 76.6)	69.1 (68.0 - 70.2)	52.1 (50.7 - 53.5)	35.4 (33.7 - 37.0)	19.4 (17.5 - 21.5)
Cardiovascular diseases	Subarachnoid hem	Female	75-84	8141	4.34 (4.51 - 4.15)	72.1 (71.1 - 73.0)	58.2 (57.1 - 59.3)	45.5 (44.3 - 46.6)	20.3 (19.2 - 21.4)	6.8 (6.0 - 7.8)	1.8 (1.1 - 2.6)
Cardiovascular diseases	Subarachnoid hem	Female	85-95	5223	1.58 (1.68 - 1.46)	56.8 (55.4 - 58.1)	35.3 (33.9 - 36.6)	19.5 (18.4 - 20.7)	3.7 (3.1 - 4.4)	0.3 (0.1 - 0.7)	0.1 (0.0 - 0.5)
Chronic respiratory	Asthma	Female	All	245647	NR	95.8 (95.7 - 95.9)	91.5 (91.4 - 91.6)	87.6 (87.5 - 87.8)	78.7 (78.5 - 78.9)	70.7 (70.5 - 71.0)	64.0 (63.7 - 64.3)
Chronic respiratory	Asthma	Female	16-24	29003	NR	99.9 (99.9 - 99.9)	99.8 (99.8 - 99.9)	99.7 (99.7 - 99.8)	99.5 (99.4 - 99.5)	99.2 (99.1 - 99.3)	98.8 (98.6 - 99.0)
Chronic respiratory	Asthma	Female	25-34	29291	NR	99.8 (99.8 - 99.9)	99.7 (99.6 - 99.7)	99.5 (99.4 - 99.6)	99.0 (98.9 - 99.1)	98.4 (98.2 - 98.6)	97.4 (97.1 - 97.7)
Chronic respiratory	Asthma	Female	35-44	31982	NR	99.7 (99.7 - 99.8)	99.4 (99.3 - 99.4)	99.0 (98.8 - 99.1)	97.8 (97.6 - 98.0)	96.1 (95.8 - 96.4)	93.8 (93.4 - 94.3)
Chronic respiratory	Asthma	Female	45-54	34256	NR	99.3 (99.2 - 99.3)	98.2 (98.1 - 98.4)	97.3 (97.1 - 97.5)	94.6 (94.3 - 94.9)	90.5 (90.1 - 90.9)	84.9 (84.2 - 85.5)
Chronic respiratory	Asthma	Female	55-64	36259	NR	97.8 (97.7 - 98.0)	95.4 (95.2 - 95.6)	93.1 (92.9 - 93.4)	86.6 (86.2 - 87.0)	76.9 (76.4 - 77.5)	64.0 (63.2 - 64.9)
Chronic respiratory	Asthma	Female	65-74	37388	15.0 (15.21 - 14.87)	95.6 (95.4 - 95.8)	90.5 (90.2 - 90.8)	85.4 (85.0 - 85.7)	69.7 (69.2 - 70.3)	50.0 (49.3 - 50.7)	28.0 (27.0 - 29.0)
Chronic respiratory	Asthma	Female	75-84	32079	7.73 (7.84 - 7.62)	89.3 (88.9 - 89.6)	77.9 (77.4 - 78.3)	66.4 (65.8 - 67.0)	36.3 (35.7 - 37.0)	13.7 (13.2 - 14.3)	3.5 (3.1 - 4.0)
Chronic respiratory	Asthma	Female	85-95	14667	3.1 (3.19 - 3.01)	74.6 (73.9 - 75.3)	50.9 (50.1 - 51.8)	32.5 (31.7 - 33.4)	7.3 (6.8 - 7.8)	0.8 (0.6 - 1.1)	0.1 (0.0 - 0.3)
Chronic respiratory	COPD	Female	All	194003	7.99 (8.06 - 7.93)	85.5 (85.4 - 85.7)	73.3 (73.1 - 73.5)	63.0 (62.8 - 63.3)	42.8 (42.5 - 43.0)	29.0 (28.8 - 29.3)	19.9 (19.6 - 20.2)
Chronic respiratory	COPD	Female	16-24	1045	NR	99.3 (98.6 - 99.7)	98.6 (97.6 - 99.2)	97.8 (96.7 - 98.6)	96.2 (94.7 - 97.3)	94.8 (93.0 - 96.2)	94.8 (93.0 - 96.2)
Chronic respiratory	COPD	Female	25-34	1768	NR	99.2 (98.7 - 99.5)	98.2 (97.4 - 98.7)	97.4 (96.5 - 98.0)	95.8 (94.7 - 96.7)	94.1 (92.6 - 95.2)	91.6 (89.5 - 93.2)
Chronic respiratory	COPD	Female	35-44	4146	NR	98.7 (98.3 - 99.0)	97.2 (96.6 - 97.6)	95.7 (95.0 - 96.3)	92.0 (91.1 - 92.8)	87.0 (85.8 - 88.2)	82.3 (80.7 - 83.9)
Chronic respiratory	COPD	Female	45-54	12808	NR	96.9 (96.6 - 97.2)	93.7 (93.2 - 94.1)	91.0 (90.5 - 91.5)	82.3 (81.5 - 83.0)	72.1 (71.1 - 73.0)	60.1 (58.8 - 61.4)
Chronic respiratory	COPD	Female	55-64	34925	15.35 (15.56 - 15.13)	93.9 (93.6 - 94.1)	87.9 (87.5 - 88.2)	82.3 (81.8 - 82.7)	67.5 (67.0 - 68.1)	51.0 (50.4 - 51.7)	35.1 (34.3 - 36.0)
Chronic respiratory	COPD	Female	65-74	58279	9.23 (9.33 - 9.13)	89.0 (88.7 - 89.2)	79.1 (78.8 - 79.5)	69.8 (69.4 - 70.2)	46.5 (46.0 - 47.0)	26.1 (25.5 - 26.6)	11.5 (10.9 - 12.1)
Chronic respiratory	COPD	Female	75-84	57250	4.94 (5.0 - 4.88)	81.0 (80.7 - 81.3)	64.5 (64.1 - 64.9)	49.5 (49.1 - 50.0)	20.5 (20.1 - 20.9)	6.3 (6.0 - 6.6)	1.3 (1.1 - 1.6)
Chronic respiratory	COPD	Female	85-95	22916	2.13 (2.19 - 2.08)	66.2 (65.6 - 66.8)	39.8 (39.1 - 40.5)	22.7 (22.1 - 23.3)	4.0 (3.6 - 4.3)	0.3 (0.2 - 0.5)	0.1 (0.0 - 0.2)
Chronic respiratory	Interstitial lung disease	Female	All	27572	11.57 (11.93 - 11.2)	85.7 (85.3 - 86.1)	74.4 (73.8 - 74.9)	66.5 (65.9 - 67.1)	53.2 (52.5 - 53.8)	44.2 (43.5 - 44.9)	37.4 (36.5 - 38.2)
Chronic respiratory	Interstitial lung disease	Female	16-24	309	NR	98.7 (96.6 - 99.5)	98.0 (95.7 - 99.1)	97.3 (94.6 - 98.6)	96.8 (93.9 - 98.3)	94.2 (90.2 - 96.6)	90.4 (83.5 - 94.5)
Chronic respiratory	Interstitial lung disease	Female	25-34	1322	NR	98.5 (97.7 - 99.1)	97.7 (96.7 - 98.4)	97.2 (96.2 - 98.0)	96.5 (95.3 - 97.4)	95.6 (94.1 - 96.6)	95.0 (93.4 - 96.3)
Chronic respiratory	Interstitial lung disease	Female	35-44	1995	NR	98.6 (98.0 - 99.0)	97.4 (96.5 - 98.0)	96.5 (95.5 - 97.2)	93.7 (92.4 - 94.7)	91.1 (89.6 - 92.5)	88.7 (86.7 - 90.4)
Chronic respiratory	Interstitial lung disease	Female	45-54	2995	NR	97.2 (96.6 - 97.8)	94.2 (93.3 - 95.0)	92.7 (91.7 - 93.6)	88.1 (86.8 - 89.3)	82.6 (80.9 - 84.3)	76.7 (74.4 - 78.9)
Chronic respiratory	Interstitial lung disease	Female	55-64	4748	20.74 (21.97 - 19.75)	94.5 (93.8 - 95.1)	89.3 (88.4 - 90.2)	85.0 (83.9 - 86.0)	74.5 (73.1 - 75.9)	64.9 (63.1 - 66.6)	52.0 (49.7 - 54.3)
Chronic respiratory	Interstitial lung disease	Female	65-74	6489	9.55 (9.92 - 9.2)	87.8 (87.0 - 88.6)	76.5 (75.4 - 77.6)	67.5 (66.3 - 68.7)	48.3 (46.8 - 49.7)	30.2 (28.5 - 31.9)	15.4 (13.6 - 17.3)
Chronic respiratory	Interstitial lung disease	Female	75-84	6897	4.01 (4.21 - 3.9)	77.7 (76.7 - 78.7)	58.3 (57.1 - 59.5)	43.7 (42.4 - 45.0)	18.2 (17.1 - 19.5)	5.6 (4.8 - 6.5)	1.5 (0.9 - 2.3)
Chronic respiratory	Interstitial lung disease	Female	85-95	2740	1.3 (1.4 - 1.21)	56.4 (54.5 - 58.3)	29.6 (27.8 - 31.4)	14.9 (13.5 - 16.4)	2.3 (1.6 - 3.2)	0.0 (0.0 - 0.0)	0.0 (0.0 - 0.0)
Chronic respiratory	Pneumoconiosis	Female	All	678	10.68 (12.22 - 9.18)	84.6 (81.6 - 87.1)	75.3 (71.8 - 78.4)	67.3 (63.6 - 70.8)	51.8 (47.7 - 55.6)	43.0 (38.9 - 47.0)	36.4 (32.0 - 40.8)
Chronic respiratory	Pneumoconiosis	Female	16-24	21	NR	100.0 (100.0 - 100.0)	100.0 (100.0 - 100.0)	100.0 (100.0 - 100.0)	94.1 (65.0 - 99.1)	94.1 (65.0 - 99.1)	94.1 (65.0 - 99.1)
Chronic respiratory	Pneumoconiosis	Female	25-34	29	NR	96.6 (77.9 - 99.5)	96.6 (77.9 - 99.5)	96.6 (77.9 - 99.5)	92.7 (73.7 - 98.1)	92.7 (73.7 - 98.1)	92.7 (73.7 - 98.1)
Chronic respiratory	Pneumoconiosis	Female	35-44	42	NR	97.5 (83.5 - 99.6)	92.4 (78.2 - 97.5)	89.7 (74.7 - 96.0)	83.4 (66.4 - 92.2)	83.4 (66.4 - 92.2)	83.4 (66.4 - 92.2)
Chronic respiratory	Pneumoconiosis	Female	45-54	58	22.3 (22.3 - 20.53)	94.8 (84.7 - 98.3)	93.0 (82.4 - 97.3)	93.0 (82.4 - 97.3)	84.7 (71.6 - 92.1)	80.1 (66.0 - 88.9)	75.4 (58.3 - 86.3)
Chronic respiratory	Pneumoconiosis	Female	55-64	125	NR	94.4 (88.6 - 97.3)	91.9 (85.4 - 95.5)	86.7 (79.2 - 91.7)	71.2 (61.9 - 78.6)	64.8 (55.0 - 73.0)	60.0 (49.4 - 69.1)
Chronic respiratory	Pneumoconiosis	Female	65-74	129	12.26 (16.95 - 10.37)	86.8 (79.6 - 91.6)	80.4 (72.4 - 86.3)	77.2 (68.9 - 83.6)	63.1 (53.7 - 71.0)	45.0 (35.4 - 54.1)	25.6 (15.8 - 36.6)
Chronic respiratory	Pneumoconiosis	Female	75-84	193	4.69 (6.2 - 3.9)	76.2 (69.5 - 81.6)	63.4 (56.2 - 69.8)	48.7 (41.3 - 55.6)	25.5 (19.3 - 32.2)	12.5 (7.6 - 18.7)	3.5 (0.4 - 12.7)
Chronic respiratory	Pneumoconiosis	Female	85-95	79	1.92 (2.61 - 1.39)	65.7 (54.1 - 75.1)	35.5 (25.0 - 46.1)	18.9 (11.0 - 28.6)	0.0 (0.0 - 0.0)	0.0 (0.0 - 0.0)	0.0 (0.0 - 0.0)
Chronic respiratory	Sleep Apnea	Female	All	88363	NR	98.6 (98.5 - 98.7)	96.4 (96.3 - 96.6)	94.0 (93.9 - 94.2)	87.2 (87.0 - 87.5)	78.9 (78.5 - 79.3)	68.7 (68.0 - 69.4)
Chronic respiratory	Sleep Apnea	Female	16-24	1277	NR	99.7 (99.2 - 99.9)	99.3 (98.7 - 99.7)	98.7 (97.9 - 99.2)	97.8 (96.5 - 98.6)	96.4 (94.5 - 97.7)	94.6 (91.5 - 96.7)
Chronic respiratory	Sleep Apnea	Female	25-34	3453	NR	99.6 (99.4 - 99.8)	99.3 (98.9 - 99.5)	98.9 (98.5 - 99.2)	98.0 (97.3 - 98.5)	96.3 (95.1 - 97.2)	93.0 (90.7 - 94.7)
Chronic respiratory	Sleep Apnea	Female	35-44	7925	NR	99.7 (99.6 - 99.8)	99.3 (99.1 - 99.5)	98.6 (98.3 - 98.9)	96.9 (96.4 - 97.3)	94.2 (93.4 - 94.9)	90.7 (89.2 - 91.9)
Chronic respiratory	Sleep Apnea	Female	45-54	18737	NR	99.6 (99.5 - 99.7)	99.0 (98.9 - 99.2)	98.2 (98.0 - 98.4)	95.7 (95.4 - 96.1)	92.2 (91.6 - 92.8)	86.6 (85.5 - 87.7)
Chronic respiratory	Sleep Apnea	Female	55-64	28221	NR	99.3 (99.2 - 99.4)	97.9 (97.7 - 98.1)	96.4 (96.2 - 96.7)	91.6 (91.2 - 92.0)	84.6 (84.0 - 85.2)	73.9 (72.7 - 75.1)
Chronic respiratory	Sleep Apnea	Female	65-74	20279	17.99 (18.29 - 17.74)	98.1 (97.9 - 98.3)	94.8 (94.5 - 95.2)	91.1 (90.6 - 91.5)	79.9 (79.2 - 80.6)	63.8 (62.7 - 64.9)	40.8 (38.6 - 42.9)
Chronic respiratory	Sleep Apnea	Female	75-84	7678	10.48 (10.81 - 10.18)	94.6 (94.1 - 95.1)	86.7 (85.8 - 87.5)	77.9 (76.8 - 78.9)	52.7 (51.1 - 54.2)	25.0 (22.9 - 27.1)	9.3 (7.0 - 12.1)
Chronic respiratory	Sleep Apnea	Female	85-95	782	4.73 (5.25 - 4.11)	81.9 (79.0 - 84.5)	65.4 (61.7 - 68.9)	48.0 (43.8 - 52.1)	14.8 (11.1 - 19.1)	2.9 (0.7 - 7.8)	0.0 (0.0 - 0.0)

Appendix Table A7: Unadjusted Median Survival and Survival Probabilities by Age, females (Page 10 of 18)

Group	Diagnosis Category	Sex	Age	N	Median Survival (y)	1y (%)	3y (%)	5y (%)	10y (%)	15y (%)	20y (%)
Diabetes & CKD	Acute glomerulonephritis	Female	All	2028	NR	95.2 (94.1 - 96.0)	91.1 (89.8 - 92.3)	87.3 (85.8 - 88.8)	78.8 (76.8 - 80.6)	70.3 (67.9 - 72.5)	61.5 (58.0 - 64.8)
Diabetes & CKD	Acute glomerulonephritis	Female	16-24	257	NR	100.0 (100.0 - 100.0)	100.0 (100.0 - 100.0)	100.0 (100.0 - 100.0)	99.5 (96.5 - 99.9)	99.5 (96.5 - 99.9)	96.9 (84.4 - 99.4)
Diabetes & CKD	Acute glomerulonephritis	Female	25-34	216	NR	100.0 (100.0 - 100.0)	100.0 (100.0 - 100.0)	100.0 (100.0 - 100.0)	98.8 (95.4 - 99.7)	98.0 (93.7 - 99.4)	98.0 (93.7 - 99.4)
Diabetes & CKD	Acute glomerulonephritis	Female	35-44	254	NR	99.6 (97.2 - 99.9)	99.2 (96.8 - 99.8)	98.3 (95.6 - 99.4)	96.9 (93.7 - 98.5)	95.5 (91.4 - 97.7)	92.3 (84.6 - 96.3)
Diabetes & CKD	Acute glomerulonephritis	Female	45-54	271	NR	98.5 (96.0 - 99.4)	97.3 (94.5 - 98.7)	97.3 (94.5 - 98.7)	96.5 (93.3 - 98.1)	92.7 (88.2 - 95.5)	85.5 (73.8 - 92.2)
Diabetes & CKD	Acute glomerulonephritis	Female	55-64	329	21.91	97.2 (94.7 - 98.5)	94.3 (91.1 - 96.4)	91.2 (87.5 - 93.9)	82.7 (77.8 - 86.6)	76.4 (70.8 - 81.2)	56.0 (44.1 - 66.4)
Diabetes & CKD	Acute glomerulonephritis	Female	65-74	358	14.08 (15.17 - 12.38)	94.5 (91.5 - 96.5)	88.4 (84.5 - 91.4)	82.1 (77.5 - 85.9)	65.1 (59.2 - 70.4)	46.8 (40.1 - 53.1)	23.3 (14.1 - 33.9)
Diabetes & CKD	Acute glomerulonephritis	Female	75-84	276	6.48 (7.5 - 5.23)	83.4 (78.3 - 87.4)	69.8 (63.8 - 75.1)	57.5 (51.0 - 63.5)	32.2 (26.1 - 38.5)	11.7 (7.5 - 16.9)	5.6 (2.3 - 11.2)
Diabetes & CKD	Acute glomerulonephritis	Female	85-95	67	3.23 (4.55 - 1.42)	70.8 (58.1 - 80.3)	52.1 (38.9 - 63.7)	33.2 (21.4 - 45.5)	14.8 (6.7 - 25.9)	2.5 (0.2 - 11.0)	0.0 (0.0 - 0.0)
Diabetes & CKD	Chronic kidney disease	Female	All	187776	3.67 (3.72 - 3.63)	68.8 (68.6 - 69.0)	53.8 (53.6 - 54.1)	43.4 (43.2 - 43.7)	28.7 (28.5 - 29.0)	21.8 (21.6 - 22.1)	17.7 (17.4 - 18.0)
Diabetes & CKD	Chronic kidney disease	Female	16-24	3297	NR	97.9 (97.4 - 98.4)	97.0 (96.3 - 97.5)	96.1 (95.4 - 96.8)	95.3 (94.5 - 96.1)	94.2 (93.1 - 95.0)	92.7 (91.3 - 93.9)
Diabetes & CKD	Chronic kidney disease	Female	25-34	4922	NR	97.8 (97.3 - 98.2)	96.9 (96.3 - 97.3)	96.2 (95.6 - 96.7)	94.4 (93.6 - 95.0)	92.0 (91.0 - 92.9)	88.7 (87.2 - 90.0)
Diabetes & CKD	Chronic kidney disease	Female	35-44	6222	NR	95.6 (95.1 - 96.1)	94.1 (93.4 - 94.6)	92.9 (92.3 - 93.6)	89.2 (88.3 - 90.0)	85.0 (83.9 - 86.1)	79.3 (77.8 - 80.7)
Diabetes & CKD	Chronic kidney disease	Female	45-54	9638	NR	90.7 (90.1 - 91.3)	87.0 (86.3 - 87.6)	84.1 (83.3 - 84.8)	76.1 (75.1 - 77.0)	68.2 (67.0 - 69.4)	59.5 (58.0 - 60.9)
Diabetes & CKD	Chronic kidney disease	Female	55-64	16380	12.63 (13.01 - 12.2)	84.4 (83.9 - 85.0)	77.1 (76.5 - 77.8)	71.1 (70.3 - 71.8)	57.1 (56.2 - 58.0)	43.9 (42.9 - 44.9)	31.5 (30.3 - 32.7)
Diabetes & CKD	Chronic kidney disease	Female	65-74	32743	6.32 (6.44 - 6.19)	77.1 (76.6 - 77.5)	65.5 (65.0 - 66.1)	55.8 (55.2 - 56.3)	34.5 (33.9 - 35.2)	18.9 (18.3 - 19.7)	8.3 (7.6 - 9.1)
Diabetes & CKD	Chronic kidney disease	Female	75-84	57170	2.99 (3.04 - 2.94)	67.6 (67.3 - 68.0)	49.9 (49.4 - 50.3)	35.8 (35.4 - 36.2)	12.8 (12.4 - 13.1)	3.1 (2.8 - 3.4)	0.5 (0.3 - 0.7)
Diabetes & CKD	Chronic kidney disease	Female	85-95	53100	1.04 (1.07 - 1.02)	50.7 (50.3 - 51.1)	28.0 (27.6 - 28.4)	14.1 (13.7 - 14.4)	1.9 (1.7 - 2.0)	0.2 (0.1 - 0.3)	0.1 (0.0 - 0.2)
Diabetes & CKD	Diabetes	Female	All	325958	12.44 (12.51 - 12.36)	90.7 (90.6 - 90.8)	82.5 (82.3 - 82.6)	74.8 (74.7 - 75.0)	57.5 (57.3 - 57.7)	42.8 (42.6 - 43.1)	32.0 (31.8 - 32.3)
Diabetes & CKD	Diabetes	Female	16-24	4989	NR	99.4 (99.2 - 99.6)	99.2 (98.9 - 99.4)	98.9 (98.6 - 99.2)	98.2 (97.8 - 98.6)	97.8 (97.2 - 98.2)	97.1 (96.4 - 97.7)
Diabetes & CKD	Diabetes	Female	25-34	9250	NR	99.5 (99.3 - 99.6)	99.0 (98.8 - 99.2)	98.5 (98.3 - 98.8)	97.5 (97.1 - 97.8)	96.2 (95.7 - 96.7)	94.8 (94.1 - 95.3)
Diabetes & CKD	Diabetes	Female	35-44	15736	NR	99.0 (98.8 - 99.1)	98.2 (98.0 - 98.4)	97.4 (97.1 - 97.6)	95.1 (94.7 - 95.4)	91.8 (91.2 - 92.3)	87.2 (86.4 - 87.9)
Diabetes & CKD	Diabetes	Female	45-54	31110	NR	97.9 (97.7 - 98.0)	96.0 (95.8 - 96.3)	94.4 (94.2 - 94.7)	89.7 (89.3 - 90.0)	82.7 (82.2 - 83.2)	73.3 (72.5 - 74.0)
Diabetes & CKD	Diabetes	Female	55-64	59454	20.58 (20.76 - 20.39)	96.1 (96.0 - 96.3)	92.9 (92.7 - 93.1)	89.8 (89.5 - 90.0)	80.7 (80.4 - 81.1)	68.2 (67.7 - 68.7)	51.9 (51.3 - 52.6)
Diabetes & CKD	Diabetes	Female	65-74	84927	13.16 (13.26 - 13.05)	93.1 (92.9 - 93.3)	87.2 (86.9 - 87.4)	81.4 (81.1 - 81.6)	63.6 (63.2 - 64.0)	40.8 (40.4 - 41.3)	19.2 (18.7 - 19.7)
Diabetes & CKD	Diabetes	Female	75-84	83512	6.99 (7.05 - 6.94)	87.3 (87.0 - 87.5)	75.1 (74.8 - 75.4)	62.4 (62.0 - 62.7)	31.6 (31.2 - 32.0)	10.1 (9.9 - 10.4)	1.9 (1.7 - 2.1)
Diabetes & CKD	Diabetes	Female	85-95	35545	2.81 (2.85 - 2.76)	71.7 (71.3 - 72.2)	48.0 (47.4 - 48.5)	29.2 (28.7 - 29.7)	5.4 (5.1 - 5.7)	0.4 (0.3 - 0.6)	0.1 (0.1 - 0.2)
Diabetes & CKD	Diabetes type 1	Female	All	77808	11.98 (12.12 - 11.83)	89.5 (89.3 - 89.7)	80.0 (79.7 - 80.3)	71.9 (71.6 - 72.2)	55.4 (55.0 - 55.7)	43.0 (42.6 - 43.4)	34.4 (34.0 - 34.8)
Diabetes & CKD	Diabetes type 1	Female	16-24	3528	NR	99.7 (99.4 - 99.8)	99.6 (99.3 - 99.7)	99.4 (99.0 - 99.6)	98.8 (98.3 - 99.1)	98.5 (98.0 - 98.9)	97.8 (97.1 - 98.4)
Diabetes & CKD	Diabetes type 1	Female	25-34	4807	NR	99.7 (99.5 - 99.8)	99.4 (99.2 - 99.6)	98.9 (98.5 - 99.2)	98.0 (97.5 - 98.4)	96.7 (96.0 - 97.2)	95.1 (94.3 - 95.8)
Diabetes & CKD	Diabetes type 1	Female	35-44	5632	NR	99.0 (98.7 - 99.2)	98.2 (97.8 - 98.5)	97.4 (96.9 - 97.8)	94.8 (94.2 - 95.4)	91.3 (90.5 - 92.1)	86.8 (85.7 - 87.8)
Diabetes & CKD	Diabetes type 1	Female	45-54	8172	NR	98.0 (97.7 - 98.3)	95.6 (95.2 - 96.0)	93.4 (92.9 - 94.0)	87.2 (86.4 - 87.9)	79.5 (78.5 - 80.4)	69.8 (68.5 - 71.0)
Diabetes & CKD	Diabetes type 1	Female	55-64	12769	18.09 (18.44 - 17.76)	94.8 (94.4 - 95.2)	90.6 (90.1 - 91.1)	86.4 (85.8 - 87.0)	75.1 (74.3 - 75.8)	60.2 (59.2 - 61.1)	43.1 (42.0 - 44.3)
Diabetes & CKD	Diabetes type 1	Female	65-74	17001	10.69 (10.88 - 10.51)	90.6 (90.1 - 91.0)	82.1 (81.6 - 82.7)	74.1 (73.5 - 74.8)	52.9 (52.1 - 53.7)	30.7 (29.8 - 31.5)	12.7 (12.0 - 13.5)
Diabetes & CKD	Diabetes type 1	Female	75-84	18119	5.26 (5.38 - 5.15)	83.2 (82.6 - 83.7)	66.6 (65.9 - 67.3)	51.8 (51.0 - 52.5)	21.6 (20.9 - 22.2)	5.9 (5.5 - 6.3)	0.9 (0.7 - 1.2)
Diabetes & CKD	Diabetes type 1	Female	85-95	7518	2.15 (2.25 - 2.07)	67.3 (66.2 - 68.3)	39.8 (38.7 - 40.9)	21.8 (20.8 - 22.7)	3.2 (2.8 - 3.6)	0.4 (0.2 - 0.6)	0.1 (0.0 - 0.3)
Diabetes & CKD	Diabetes type 2	Female	All	302269	11.24 (11.32 - 11.18)	90.2 (90.1 - 90.3)	81.2 (81.1 - 81.4)	72.9 (72.7 - 73.1)	54.2 (54.0 - 54.4)	38.5 (38.3 - 38.8)	26.6 (26.3 - 26.9)
Diabetes & CKD	Diabetes type 2	Female	16-24	1998	NR	99.2 (98.7 - 99.5)	98.7 (98.1 - 99.1)	98.2 (97.4 - 98.7)	96.7 (95.7 - 97.5)	95.4 (94.1 - 96.4)	94.5 (92.6 - 96.0)
Diabetes & CKD	Diabetes type 2	Female	25-34	5436	NR	99.4 (99.1 - 99.5)	98.6 (98.2 - 98.9)	97.9 (97.4 - 98.2)	96.3 (95.6 - 96.8)	94.2 (93.3 - 95.0)	90.6 (89.1 - 91.9)
Diabetes & CKD	Diabetes type 2	Female	35-44	12194	NR	98.9 (98.7 - 99.1)	98.0 (97.7 - 98.2)	96.9 (96.6 - 97.2)	93.9 (93.4 - 94.4)	89.4 (88.7 - 90.1)	82.8 (81.6 - 83.9)
Diabetes & CKD	Diabetes type 2	Female	45-54	27378	NR	97.9 (97.7 - 98.0)	95.9 (95.6 - 96.1)	94.1 (93.8 - 94.4)	88.6 (88.2 - 89.1)	80.6 (80.0 - 81.3)	69.2 (68.3 - 70.2)
Diabetes & CKD	Diabetes type 2	Female	55-64	55100	19.83 (20.12 - 19.66)	96.3 (96.1 - 96.5)	92.8 (92.5 - 93.0)	89.4 (89.1 - 89.6)	79.6 (79.2 - 79.9)	66.3 (65.8 - 66.8)	49.5 (48.8 - 50.2)
Diabetes & CKD	Diabetes type 2	Female	65-74	81414	12.78 (12.88 - 12.67)	93.3 (93.1 - 93.5)	87.1 (86.8 - 87.3)	80.9 (80.6 - 81.2)	62.3 (61.9 - 62.7)	39.2 (38.7 - 39.6)	17.9 (17.3 - 18.4)
Diabetes & CKD	Diabetes type 2	Female	75-84	81937	6.78 (6.84 - 6.72)	87.0 (86.8 - 87.3)	74.3 (74.0 - 74.6)	61.2 (60.9 - 61.6)	30.2 (29.8 - 30.6)	9.4 (9.1 - 9.7)	1.8 (1.6 - 2.0)
Diabetes & CKD	Diabetes type 2	Female	85-95	35479	2.78 (2.83 - 2.73)	71.5 (71.0 - 72.0)	47.6 (47.1 - 48.2)	28.5 (28.0 - 29.0)	5.2 (4.9 - 5.4)	0.4 (0.3 - 0.5)	0.1 (0.0 - 0.2)
Digestive diseases	Cirrhosis	Female	All	67539	NR	90.8 (90.6 - 91.0)	84.9 (84.7 - 85.2)	80.4 (80.1 - 80.7)	71.0 (70.6 - 71.3)	62.9 (62.5 - 63.4)	55.5 (54.9 - 56.0)
Digestive diseases	Cirrhosis	Female	16-24	4301	NR	99.2 (98.9 - 99.4)	98.1 (97.6 - 98.5)	97.4 (96.8 - 97.8)	96.0 (95.3 - 96.6)	94.4 (93.5 - 95.1)	92.4 (91.2 - 93.4)
Digestive diseases	Cirrhosis	Female	25-34	8304	NR	99.3 (99.1 - 99.5)	98.3 (98.0 - 98.6)	97.5 (97.1 - 97.8)	95.6 (95.1 - 96.1)	94.2 (93.6 - 94.8)	92.0 (91.2 - 92.8)
Digestive diseases	Cirrhosis	Female	35-44	8690	NR	98.2 (97.9 - 98.5)	97.0 (96.6 - 97.4)	95.7 (95.2 - 96.1)	92.2 (91.6 - 92.8)	87.9 (87.1 - 88.7)	83.4 (82.3 - 84.4)
Digestive diseases	Cirrhosis	Female	45-54	11369	NR	95.4 (95.0 - 95.8)	92.1 (91.6 - 92.6)	89.2 (88.6 - 89.8)	81.9 (81.1 - 82.7)	74.6 (73.6 - 75.5)	65.5 (64.2 - 66.7)

Appendix Table A7: Unadjusted Median Survival and Survival Probabilities by Age, females (Page 11 of 18)

Group	Diagnosis Category	Sex	Age	N	Median Survival (y)	1y (%)	3y (%)	5y (%)	10y (%)	15y (%)	20y (%)
Digestive diseases	Cirrhosis	Female	55-64	13709	18.52 (18.95 - 18.1)	91.7 (91.2 - 92.2)	85.8 (85.1 - 86.3)	80.8 (80.1 - 81.5)	69.7 (68.8 - 70.6)	58.3 (57.2 - 59.3)	45.8 (44.4 - 47.1)
Digestive diseases	Cirrhosis	Female	65-74	12054	10.43 (10.74 - 10.13)	85.5 (84.8 - 86.1)	76.0 (75.2 - 76.8)	68.5 (67.6 - 69.3)	51.4 (50.3 - 52.5)	34.5 (33.2 - 35.8)	18.7 (17.2 - 20.2)
Digestive diseases	Cirrhosis	Female	75-84	7106	4.82 (5.07 - 4.61)	76.0 (75.0 - 77.0)	60.9 (59.7 - 62.1)	49.1 (47.9 - 50.4)	24.8 (23.5 - 26.1)	9.2 (8.2 - 10.3)	2.3 (1.6 - 3.1)
Digestive diseases	Cirrhosis	Female	85-95	1961	1.48 (1.7 - 1.29)	56.1 (53.9 - 58.3)	35.1 (32.9 - 37.3)	22.2 (20.3 - 24.3)	6.0 (4.8 - 7.4)	0.6 (0.2 - 1.5)	0.0 (0.0 - 0.0)
Digestive diseases	Crohn's disease	Female	All	27809	NR	97.6 (97.5 - 97.8)	95.0 (94.7 - 95.3)	92.5 (92.1 - 92.8)	86.5 (86.0 - 86.9)	80.6 (80.0 - 81.2)	74.4 (73.6 - 75.1)
Digestive diseases	Crohn's disease	Female	16-24	4675	NR	100.0 (99.8 - 100.0)	99.8 (99.6 - 99.9)	99.7 (99.5 - 99.8)	99.4 (99.0 - 99.6)	99.0 (98.5 - 99.3)	98.6 (97.9 - 99.1)
Digestive diseases	Crohn's disease	Female	25-34	4994	NR	100.0 (99.8 - 100.0)	99.8 (99.6 - 99.9)	99.6 (99.3 - 99.7)	99.0 (98.7 - 99.3)	98.4 (97.9 - 98.8)	97.5 (96.7 - 98.1)
Digestive diseases	Crohn's disease	Female	35-44	4201	NR	99.8 (99.6 - 99.9)	99.4 (99.1 - 99.6)	98.9 (98.5 - 99.2)	97.5 (96.9 - 98.0)	96.1 (95.3 - 96.8)	94.5 (93.4 - 95.4)
Digestive diseases	Crohn's disease	Female	45-54	4301	NR	99.1 (98.8 - 99.4)	98.1 (97.6 - 98.5)	96.9 (96.3 - 97.4)	93.4 (92.5 - 94.2)	89.4 (88.3 - 90.5)	83.5 (81.9 - 85.0)
Digestive diseases	Crohn's disease	Female	55-64	3984	NR	98.2 (97.8 - 98.6)	95.7 (95.0 - 96.3)	93.4 (92.6 - 94.2)	86.8 (85.6 - 88.0)	78.4 (76.8 - 79.9)	66.4 (64.1 - 68.5)
Digestive diseases	Crohn's disease	Female	65-74	3100	14.89 (15.41 - 14.35)	95.4 (94.6 - 96.0)	89.9 (88.8 - 90.9)	84.3 (82.9 - 85.6)	68.4 (66.4 - 70.2)	49.8 (47.4 - 52.2)	28.0 (25.2 - 30.8)
Digestive diseases	Crohn's disease	Female	75-84	1928	7.15 (7.7 - 6.7)	88.2 (86.7 - 89.6)	76.1 (74.1 - 78.0)	63.3 (60.9 - 65.5)	34.8 (32.3 - 37.4)	12.5 (10.4 - 14.7)	3.9 (2.4 - 5.8)
Digestive diseases	Crohn's disease	Female	85-95	603	3.05 (3.44 - 2.58)	74.2 (70.5 - 77.5)	50.2 (46.0 - 54.2)	32.1 (28.2 - 36.1)	7.6 (5.4 - 10.4)	0.7 (0.1 - 2.1)	0.7 (0.1 - 2.1)
Digestive diseases	Diverticular disease	Female	All	212472	17.26 (17.4 - 17.12)	95.2 (95.1 - 95.2)	89.5 (89.3 - 89.6)	83.7 (83.5 - 83.9)	69.3 (69.1 - 69.6)	55.6 (55.3 - 55.9)	43.4 (43.0 - 43.9)
Digestive diseases	Diverticular disease	Female	16-24	258	NR	99.6 (97.1 - 99.9)	99.2 (96.7 - 99.8)	98.7 (96.0 - 99.6)	98.2 (95.1 - 99.3)	98.2 (95.1 - 99.3)	96.8 (91.6 - 98.8)
Digestive diseases	Diverticular disease	Female	25-34	2052	NR	99.7 (99.3 - 99.9)	99.6 (99.2 - 99.8)	99.1 (98.6 - 99.5)	98.5 (97.7 - 99.0)	97.4 (96.2 - 98.2)	96.7 (95.2 - 97.7)
Digestive diseases	Diverticular disease	Female	35-44	8710	NR	99.7 (99.5 - 99.8)	99.4 (99.3 - 99.6)	99.1 (98.8 - 99.3)	97.9 (97.5 - 98.2)	96.5 (95.9 - 96.9)	94.2 (93.2 - 95.0)
Digestive diseases	Diverticular disease	Female	45-54	23552	NR	99.5 (99.3 - 99.5)	98.6 (98.5 - 98.8)	98.0 (97.8 - 98.2)	95.8 (95.5 - 96.1)	93.0 (92.5 - 93.4)	88.2 (87.4 - 89.0)
Digestive diseases	Diverticular disease	Female	55-64	46120	NR	98.7 (98.6 - 98.8)	97.0 (96.9 - 97.2)	95.5 (95.3 - 95.7)	90.7 (90.4 - 91.0)	83.4 (82.9 - 83.9)	72.5 (71.7 - 73.4)
Digestive diseases	Diverticular disease	Female	65-74	60190	16.98 (17.15 - 16.83)	96.9 (96.8 - 97.1)	93.1 (92.9 - 93.3)	89.2 (88.9 - 89.4)	76.7 (76.3 - 77.1)	58.8 (58.2 - 59.4)	35.8 (34.9 - 36.8)
Digestive diseases	Diverticular disease	Female	75-84	52699	8.99 (9.08 - 8.88)	92.5 (92.3 - 92.7)	83.0 (82.7 - 83.3)	72.3 (71.9 - 72.8)	44.1 (43.5 - 44.6)	18.6 (18.1 - 19.1)	4.8 (4.4 - 5.3)
Digestive diseases	Diverticular disease	Female	85-95	18395	4.13 (4.23 - 4.03)	80.9 (80.3 - 81.4)	60.6 (59.9 - 61.3)	42.1 (41.3 - 42.8)	10.9 (10.4 - 11.5)	1.6 (1.3 - 1.9)	0.1 (0.0 - 0.3)
Digestive diseases	Gallbladder & biliary	Female	All	312018	NR	94.7 (94.6 - 94.8)	90.7 (90.6 - 90.8)	87.5 (87.3 - 87.6)	80.3 (80.2 - 80.5)	73.6 (73.4 - 73.8)	66.8 (66.5 - 67.0)
Digestive diseases	Gallbladder & biliary	Female	16-24	15986	NR	99.9 (99.8 - 99.9)	99.7 (99.6 - 99.8)	99.5 (99.4 - 99.6)	99.2 (99.0 - 99.3)	98.7 (98.4 - 98.9)	98.2 (97.8 - 98.5)
Digestive diseases	Gallbladder & biliary	Female	25-34	44319	NR	99.8 (99.8 - 99.9)	99.7 (99.7 - 99.8)	99.6 (99.5 - 99.6)	99.2 (99.1 - 99.3)	98.7 (98.5 - 98.8)	97.9 (97.7 - 98.2)
Digestive diseases	Gallbladder & biliary	Female	35-44	48947	NR	99.6 (99.5 - 99.7)	99.3 (99.3 - 99.4)	99.1 (99.0 - 99.2)	98.2 (98.1 - 98.4)	97.1 (96.9 - 97.3)	95.2 (94.9 - 95.5)
Digestive diseases	Gallbladder & biliary	Female	45-54	49952	NR	98.9 (98.8 - 99.0)	98.1 (97.9 - 98.2)	97.4 (97.3 - 97.6)	95.6 (95.4 - 95.8)	92.9 (92.6 - 93.2)	88.9 (88.4 - 89.3)
Digestive diseases	Gallbladder & biliary	Female	55-64	51523	NR	97.2 (97.0 - 97.3)	95.1 (94.9 - 95.3)	93.7 (93.4 - 93.9)	89.1 (88.8 - 89.4)	82.7 (82.3 - 83.1)	73.6 (73.0 - 74.3)
Digestive diseases	Gallbladder & biliary	Female	65-74	45270	16.76 (16.94 - 16.57)	93.1 (92.8 - 93.3)	88.1 (87.8 - 88.4)	84.1 (83.7 - 84.4)	72.6 (72.2 - 73.1)	56.7 (56.0 - 57.3)	36.6 (35.7 - 37.4)
Digestive diseases	Gallbladder & biliary	Female	75-84	36125	7.67 (7.78 - 7.56)	85.4 (85.1 - 85.8)	74.0 (73.5 - 74.4)	63.6 (63.1 - 64.1)	38.4 (37.8 - 39.0)	17.0 (16.4 - 17.6)	4.3 (3.9 - 4.8)
Digestive diseases	Gallbladder & biliary	Female	85-95	18877	2.89 (2.97 - 2.82)	71.1 (70.4 - 71.7)	49.0 (48.3 - 49.7)	31.8 (31.1 - 32.5)	7.7 (7.2 - 8.2)	1.0 (0.8 - 1.2)	0.1 (0.0 - 0.2)
Digestive diseases	Inflammatory bowel	Female	All	132174	NR	94.6 (94.5 - 94.7)	89.5 (89.4 - 89.7)	85.2 (85.0 - 85.4)	76.1 (75.8 - 76.4)	68.6 (68.3 - 68.9)	62.2 (61.9 - 62.6)
Digestive diseases	Inflammatory bowel	Female	16-24	14936	NR	99.9 (99.8 - 99.9)	99.7 (99.6 - 99.8)	99.6 (99.5 - 99.7)	99.3 (99.1 - 99.4)	98.9 (98.6 - 99.0)	98.6 (98.3 - 98.8)
Digestive diseases	Inflammatory bowel	Female	25-34	18371	NR	99.8 (99.7 - 99.9)	99.6 (99.5 - 99.7)	99.5 (99.3 - 99.6)	99.0 (98.8 - 99.2)	98.3 (98.1 - 98.5)	97.5 (97.2 - 97.9)
Digestive diseases	Inflammatory bowel	Female	35-44	15641	NR	99.6 (99.4 - 99.7)	99.1 (98.9 - 99.2)	98.4 (98.2 - 98.6)	97.3 (97.0 - 97.5)	95.7 (95.3 - 96.1)	93.6 (93.0 - 94.1)
Digestive diseases	Inflammatory bowel	Female	45-54	16956	NR	98.8 (98.6 - 99.0)	97.5 (97.2 - 97.7)	96.3 (96.0 - 96.6)	93.2 (92.7 - 93.6)	89.8 (89.2 - 90.3)	84.9 (84.1 - 85.6)
Digestive diseases	Inflammatory bowel	Female	55-64	19483	NR	96.9 (96.7 - 97.2)	93.9 (93.5 - 94.2)	91.6 (91.2 - 92.0)	84.7 (84.1 - 85.2)	76.0 (75.3 - 76.8)	64.5 (63.4 - 65.5)
Digestive diseases	Inflammatory bowel	Female	65-74	19876	14.36 (14.57 - 14.14)	93.4 (93.1 - 93.8)	87.2 (86.7 - 87.7)	81.6 (81.0 - 82.2)	65.9 (65.1 - 66.6)	47.6 (46.6 - 48.5)	27.5 (26.3 - 28.7)
Digestive diseases	Inflammatory bowel	Female	75-84	18121	6.75 (6.88 - 6.62)	86.3 (85.8 - 86.8)	72.9 (72.2 - 73.6)	60.2 (59.5 - 61.0)	32.0 (31.2 - 32.9)	11.4 (10.8 - 12.1)	3.2 (2.7 - 3.7)
Digestive diseases	Inflammatory bowel	Female	85-95	8434	2.94 (3.03 - 2.82)	72.9 (72.0 - 73.9)	49.2 (48.1 - 50.3)	30.4 (29.3 - 31.4)	6.5 (5.9 - 7.1)	0.8 (0.5 - 1.1)	0.0 (0.0 - 0.0)
Digestive diseases	Pancreatitis	Female	All	53594	17.38 (17.72 - 17.02)	89.4 (89.1 - 89.6)	82.2 (81.9 - 82.5)	76.5 (76.1 - 76.9)	64.3 (63.8 - 64.8)	54.1 (53.5 - 54.7)	45.4 (44.6 - 46.1)
Digestive diseases	Pancreatitis	Female	16-24	2207	NR	99.5 (99.1 - 99.8)	98.9 (98.4 - 99.3)	98.7 (98.1 - 99.1)	98.0 (97.2 - 98.5)	97.4 (96.4 - 98.1)	96.6 (95.2 - 97.6)
Digestive diseases	Pancreatitis	Female	25-34	4477	NR	99.7 (99.4 - 99.8)	99.2 (98.8 - 99.4)	98.8 (98.4 - 99.1)	97.8 (97.2 - 98.2)	97.2 (96.5 - 97.8)	96.2 (95.1 - 97.1)
Digestive diseases	Pancreatitis	Female	35-44	4821	NR	98.9 (98.5 - 99.1)	97.4 (96.9 - 97.8)	96.5 (95.9 - 97.0)	94.5 (93.7 - 95.2)	92.2 (91.1 - 93.1)	89.1 (87.5 - 90.5)
Digestive diseases	Pancreatitis	Female	45-54	6535	NR	96.6 (96.1 - 97.0)	93.6 (92.9 - 94.2)	91.7 (90.9 - 92.4)	86.8 (85.8 - 87.8)	81.5 (80.2 - 82.8)	73.6 (71.6 - 75.4)
Digestive diseases	Pancreatitis	Female	55-64	8649	22.7	93.9 (93.4 - 94.4)	89.2 (88.5 - 89.9)	86.2 (85.4 - 87.0)	77.8 (76.7 - 78.8)	69.3 (67.9 - 70.6)	57.8 (55.8 - 59.6)
Digestive diseases	Pancreatitis	Female	65-74	10526	14.01 (14.48 - 13.61)	89.6 (88.9 - 90.1)	82.2 (81.5 - 83.0)	76.9 (76.0 - 77.7)	63.6 (62.5 - 64.7)	46.7 (45.2 - 48.2)	27.5 (25.6 - 29.5)
Digestive diseases	Pancreatitis	Female	75-84	10595	7.13 (7.32 - 6.87)	82.5 (81.8 - 83.2)	71.4 (70.5 - 72.3)	60.6 (59.6 - 61.6)	35.2 (34.1 - 36.4)	13.0 (12.0 - 14.1)	2.7 (2.0 - 3.7)
Digestive diseases	Pancreatitis	Female	85-95	5470	2.68 (2.87 - 2.53)	67.5 (66.2 - 68.7)	47.5 (46.1 - 48.8)	31.6 (30.3 - 32.9)	7.3 (6.5 - 8.3)	0.6 (0.3 - 1.1)	0.0 (0.0 - 0.0)
Digestive diseases	Ulcerative colitis	Female	All	117795	NR	94.2 (94.1 - 94.4)	88.9 (88.7 - 89.1)	84.4 (84.1 - 84.6)	74.9 (74.6 - 75.2)	67.2 (66.9 - 67.5)	60.9 (60.5 - 61.3)

Appendix Table A7: Unadjusted Median Survival and Survival Probabilities by Age, females (Page 12 of 18)

Group	Diagnosis Category	Sex	Age	N	Median Survival (y)	1y (%)	3y (%)	5y (%)	10y (%)	15y (%)	20y (%)
Digestive diseases	Ulcerative colitis	Female	16-24	12255	NR	99.9 (99.8 - 99.9)	99.7 (99.5 - 99.8)	99.6 (99.4 - 99.7)	99.3 (99.1 - 99.4)	98.8 (98.5 - 99.0)	98.5 (98.2 - 98.8)
Digestive diseases	Ulcerative colitis	Female	25-34	15946	NR	99.8 (99.7 - 99.9)	99.6 (99.4 - 99.7)	99.4 (99.3 - 99.5)	98.9 (98.8 - 99.1)	98.2 (97.9 - 98.5)	97.4 (97.0 - 97.8)
Digestive diseases	Ulcerative colitis	Female	35-44	13634	NR	99.5 (99.4 - 99.6)	99.0 (98.8 - 99.2)	98.4 (98.1 - 98.6)	97.2 (96.9 - 97.5)	95.6 (95.2 - 96.0)	93.4 (92.7 - 93.9)
Digestive diseases	Ulcerative colitis	Female	45-54	14738	NR	98.8 (98.6 - 98.9)	97.3 (97.0 - 97.6)	96.2 (95.8 - 96.5)	93.1 (92.6 - 93.5)	89.9 (89.3 - 90.5)	85.1 (84.3 - 85.9)
Digestive diseases	Ulcerative colitis	Female	55-64	17465	NR	96.8 (96.5 - 97.0)	93.6 (93.2 - 94.0)	91.3 (90.8 - 91.7)	84.3 (83.7 - 84.9)	75.7 (74.9 - 76.5)	64.3 (63.2 - 65.4)
Digestive diseases	Ulcerative colitis	Female	65-74	18319	14.27 (14.53 - 14.02)	93.3 (92.9 - 93.6)	86.9 (86.4 - 87.4)	81.3 (80.7 - 81.9)	65.5 (64.7 - 66.3)	47.1 (46.1 - 48.1)	27.3 (26.1 - 28.6)
Digestive diseases	Ulcerative colitis	Female	75-84	17054	6.72 (6.86 - 6.58)	86.2 (85.6 - 86.7)	72.7 (72.0 - 73.4)	60.0 (59.2 - 60.8)	31.8 (31.0 - 32.6)	11.4 (10.7 - 12.1)	3.1 (2.7 - 3.7)
Digestive diseases	Ulcerative colitis	Female	85-95	8049	2.95 (3.03 - 2.82)	73.0 (72.0 - 74.0)	49.2 (48.1 - 50.3)	30.3 (29.2 - 31.3)	6.4 (5.8 - 7.1)	0.8 (0.6 - 1.1)	0.0 (0.0 - 0.0)
Digestive diseases	Upper digest diseases	Female	All	497471	NR	94.7 (94.6 - 94.8)	90.0 (89.9 - 90.1)	85.8 (85.7 - 85.9)	76.1 (76.0 - 76.3)	67.3 (67.1 - 67.5)	59.2 (59.0 - 59.4)
Digestive diseases	Upper digest diseases	Female	16-24	43686	NR	99.9 (99.9 - 100.0)	99.8 (99.8 - 99.8)	99.7 (99.7 - 99.8)	99.4 (99.4 - 99.5)	99.1 (99.0 - 99.2)	98.9 (98.7 - 99.0)
Digestive diseases	Upper digest diseases	Female	25-34	53010	NR	99.8 (99.8 - 99.9)	99.7 (99.6 - 99.7)	99.5 (99.4 - 99.6)	99.1 (99.0 - 99.1)	98.5 (98.4 - 98.6)	97.7 (97.5 - 97.9)
Digestive diseases	Upper digest diseases	Female	35-44	56197	NR	99.6 (99.6 - 99.7)	99.2 (99.1 - 99.3)	98.8 (98.7 - 98.9)	97.6 (97.5 - 97.8)	96.2 (96.0 - 96.4)	94.2 (93.9 - 94.5)
Digestive diseases	Upper digest diseases	Female	45-54	67975	NR	98.9 (98.9 - 99.0)	97.8 (97.7 - 97.9)	96.9 (96.8 - 97.0)	94.4 (94.2 - 94.6)	91.2 (90.9 - 91.4)	86.2 (85.8 - 86.6)
Digestive diseases	Upper digest diseases	Female	55-64	81868	NR	97.4 (97.3 - 97.5)	95.0 (94.9 - 95.2)	92.9 (92.7 - 93.1)	87.2 (87.0 - 87.5)	79.9 (79.5 - 80.2)	69.3 (68.8 - 69.8)
Digestive diseases	Upper digest diseases	Female	65-74	86708	15.97 (16.09 - 15.84)	94.4 (94.2 - 94.6)	89.2 (89.0 - 89.5)	84.5 (84.3 - 84.8)	71.2 (70.8 - 71.5)	53.8 (53.3 - 54.2)	33.1 (32.4 - 33.7)
Digestive diseases	Upper digest diseases	Female	75-84	75115	7.58 (7.65 - 7.5)	87.5 (87.2 - 87.7)	76.0 (75.7 - 76.4)	64.8 (64.4 - 65.1)	37.0 (36.6 - 37.4)	15.2 (14.8 - 15.6)	3.9 (3.6 - 4.2)
Digestive diseases	Upper digest diseases	Female	85-95	31827	3.19 (3.25 - 3.13)	73.6 (73.1 - 74.1)	51.8 (51.3 - 52.4)	34.3 (33.8 - 34.9)	8.4 (8.0 - 8.7)	1.0 (0.8 - 1.1)	0.1 (0.0 - 0.2)
Digestive diseases	Vascular intestinal	Female	All	16980	5.36 (5.59 - 5.16)	69.4 (68.7 - 70.1)	59.9 (59.2 - 60.7)	51.4 (50.6 - 52.2)	35.2 (34.3 - 36.1)	25.0 (24.0 - 26.0)	18.8 (17.7 - 20.0)
Digestive diseases	Vascular intestinal	Female	16-24	147	NR	98.6 (94.7 - 99.7)	95.6 (90.4 - 98.0)	93.8 (88.0 - 96.9)	93.8 (88.0 - 96.9)	90.8 (82.9 - 95.1)	90.8 (82.9 - 95.1)
Digestive diseases	Vascular intestinal	Female	25-34	246	NR	95.1 (91.5 - 97.2)	93.7 (89.7 - 96.1)	92.1 (87.7 - 95.0)	90.2 (85.3 - 93.5)	89.3 (84.0 - 92.9)	89.3 (84.0 - 92.9)
Digestive diseases	Vascular intestinal	Female	35-44	432	NR	94.4 (91.7 - 96.2)	93.3 (90.5 - 95.3)	91.8 (88.7 - 94.1)	89.6 (85.9 - 92.4)	86.7 (82.2 - 90.2)	77.5 (69.2 - 83.9)
Digestive diseases	Vascular intestinal	Female	45-54	790	NR	89.5 (87.1 - 91.5)	86.1 (83.5 - 88.4)	82.8 (79.8 - 85.3)	76.4 (72.8 - 79.6)	70.4 (66.0 - 74.3)	63.2 (57.4 - 68.4)
Digestive diseases	Vascular intestinal	Female	55-64	1921	17.03 (18.16 - 14.85)	84.4 (82.7 - 86.0)	79.4 (77.5 - 81.2)	75.3 (73.2 - 77.2)	63.5 (60.9 - 66.0)	53.0 (49.8 - 56.1)	44.1 (39.9 - 48.2)
Digestive diseases	Vascular intestinal	Female	65-74	4184	8.56 (9.05 - 7.89)	77.4 (76.1 - 78.6)	69.2 (67.7 - 70.6)	61.6 (60.0 - 63.1)	44.1 (42.2 - 46.1)	29.6 (27.3 - 31.9)	16.4 (13.6 - 19.5)
Digestive diseases	Vascular intestinal	Female	75-84	5899	4.02 (4.21 - 3.76)	66.5 (65.3 - 67.7)	55.2 (53.9 - 56.5)	44.3 (42.9 - 45.7)	21.9 (20.5 - 23.3)	7.0 (5.9 - 8.3)	1.5 (0.8 - 2.6)
Digestive diseases	Vascular intestinal	Female	85-95	3212	0.54 (0.7 - 0.38)	45.8 (44.1 - 47.6)	31.6 (29.9 - 33.3)	19.3 (17.8 - 20.8)	3.8 (3.0 - 4.9)	0.5 (0.2 - 1.1)	0.2 (0.0 - 1.0)
Mental disorders & substance use	ADHD	Female	All	102191	NR	99.8 (99.8 - 99.8)	99.4 (99.4 - 99.5)	99.0 (98.9 - 99.0)	97.6 (97.5 - 97.8)	95.8 (95.5 - 96.1)	92.9 (91.9 - 93.7)
Mental disorders & substance use	ADHD	Female	16-24	45129	NR	99.9 (99.9 - 99.9)	99.7 (99.7 - 99.8)	99.5 (99.4 - 99.6)	98.8 (98.7 - 99.0)	97.9 (97.6 - 98.2)	97.1 (96.4 - 97.6)
Mental disorders & substance use	ADHD	Female	25-34	26910	NR	99.8 (99.7 - 99.9)	99.5 (99.4 - 99.6)	99.1 (99.0 - 99.2)	98.1 (97.9 - 98.3)	97.0 (96.6 - 97.4)	94.6 (92.8 - 95.9)
Mental disorders & substance use	ADHD	Female	35-44	17795	NR	99.8 (99.7 - 99.9)	99.3 (99.2 - 99.5)	98.6 (98.4 - 98.8)	97.0 (96.7 - 97.4)	94.7 (94.0 - 95.4)	90.9 (87.9 - 93.1)
Mental disorders & substance use	ADHD	Female	45-54	9351	NR	99.7 (99.5 - 99.8)	98.8 (98.5 - 99.0)	97.9 (97.5 - 98.2)	95.1 (94.5 - 95.7)	91.0 (89.7 - 92.1)	77.3 (67.3 - 84.6)
Mental disorders & substance use	ADHD	Female	55-64	2610	NR	99.1 (98.6 - 99.4)	97.7 (97.0 - 98.2)	96.6 (95.7 - 97.3)	90.9 (89.2 - 92.4)	81.7 (77.9 - 85.0)	65.9 (51.8 - 76.7)
Mental disorders & substance use	ADHD	Female	65-74	318	NR	97.0 (94.4 - 98.4)	94.0 (90.5 - 96.2)	90.2 (85.7 - 93.3)	82.1 (75.4 - 87.1)	60.5 (46.2 - 72.1)	53.8 (35.6 - 68.9)
Mental disorders & substance use	ADHD	Female	75-84	59	8.16 (9.03 - 5.41)	93.0 (82.5 - 97.3)	77.4 (63.6 - 86.6)	66.7 (51.9 - 77.9)	26.0 (10.8 - 44.3)	19.5 (6.0 - 38.6)	19.5 (6.0 - 38.6)
Mental disorders & substance use	ADHD	Female	85-95	19	4.49 (5.65 - 0.67)	73.3 (47.2 - 87.9)	60.0 (33.4 - 78.8)	40.0 (17.0 - 62.3)	10.0 (0.8 - 33.4)	10.0 (0.8 - 33.4)	10.0 (0.8 - 33.4)
Mental disorders & substance use	Alcohol use disorders	Female	All	131978	NR	97.6 (97.5 - 97.7)	94.7 (94.6 - 94.8)	92.0 (91.9 - 92.2)	85.2 (85.0 - 85.4)	78.3 (78.0 - 78.6)	71.0 (70.5 - 71.4)
Mental disorders & substance use	Alcohol use disorders	Female	16-24	42009	NR	99.8 (99.8 - 99.9)	99.6 (99.5 - 99.6)	99.3 (99.2 - 99.4)	98.7 (98.6 - 98.9)	98.0 (97.8 - 98.2)	97.2 (96.9 - 97.5)
Mental disorders & substance use	Alcohol use disorders	Female	25-34	17328	NR	99.5 (99.4 - 99.6)	98.8 (98.7 - 99.0)	98.2 (97.9 - 98.4)	96.3 (95.9 - 96.6)	94.3 (93.8 - 94.8)	91.6 (90.8 - 92.4)
Mental disorders & substance use	Alcohol use disorders	Female	35-44	16788	NR	99.2 (99.0 - 99.3)	97.8 (97.6 - 98.1)	96.5 (96.2 - 96.8)	92.8 (92.4 - 93.3)	88.1 (87.5 - 88.7)	81.6 (80.5 - 82.6)
Mental disorders & substance use	Alcohol use disorders	Female	45-54	20459	NR	98.3 (98.1 - 98.5)	96.0 (95.8 - 96.3)	93.7 (93.3 - 94.0)	86.7 (86.1 - 87.2)	77.1 (76.3 - 77.8)	65.4 (64.1 - 66.6)
Mental disorders & substance use	Alcohol use disorders	Female	55-64	17158	16.87 (17.23 - 16.54)	95.8 (95.4 - 96.1)	90.4 (90.0 - 90.9)	85.2 (84.7 - 85.8)	71.6 (70.8 - 72.4)	55.9 (54.9 - 56.9)	39.2 (37.8 - 40.7)
Mental disorders & substance use	Alcohol use disorders	Female	65-74	12170	10.74 (10.98 - 10.44)	91.8 (91.3 - 92.3)	83.0 (82.3 - 83.7)	74.6 (73.8 - 75.5)	52.7 (51.5 - 53.8)	31.4 (30.1 - 32.8)	14.1 (12.4 - 15.9)
Mental disorders & substance use	Alcohol use disorders	Female	75-84	5084	6.36 (6.54 - 6.12)	88.0 (87.1 - 88.9)	73.6 (72.2 - 74.8)	59.6 (58.0 - 61.1)	26.4 (24.7 - 28.1)	9.1 (7.7 - 10.7)	0.7 (0.1 - 2.8)
Mental disorders & substance use	Alcohol use disorders	Female	85-95	962	3.62 (3.95 - 3.27)	78.5 (75.7 - 81.0)	55.7 (52.3 - 59.0)	35.7 (32.3 - 39.0)	7.4 (5.3 - 9.9)	0.4 (0.0 - 2.1)	0.0 (0.0 - 0.0)
Mental disorders & substance use	Anorexia nervosa	Female	All	16312	NR	99.4 (99.2 - 99.5)	98.6 (98.5 - 98.8)	98.1 (97.9 - 98.3)	96.4 (96.1 - 96.8)	95.0 (94.6 - 95.5)	93.0 (92.2 - 93.7)
Mental disorders & substance use	Anorexia nervosa	Female	16-24	11565	NR	99.9 (99.8 - 99.9)	99.6 (99.5 - 99.7)	99.4 (99.2 - 99.5)	98.7 (98.4 - 98.9)	98.0 (97.7 - 98.4)	97.2 (96.6 - 97.7)
Mental disorders & substance use	Anorexia nervosa	Female	25-34	2838	NR	99.8 (99.6 - 99.9)	99.3 (98.9 - 99.6)	99.0 (98.5 - 99.3)	97.8 (97.1 - 98.4)	96.4 (95.3 - 97.3)	94.5 (92.9 - 95.8)
Mental disorders & substance use	Anorexia nervosa	Female	35-44	1028	NR	99.0 (98.1 - 99.5)	97.9 (96.8 - 98.6)	97.0 (95.6 - 97.9)	93.8 (91.9 - 95.3)	90.9 (88.4 - 93.0)	86.2 (82.1 - 89.5)
Mental disorders & substance use	Anorexia nervosa	Female	45-54	436	NR	99.1 (97.5 - 99.6)	96.5 (94.2 - 97.9)	94.9 (92.1 - 96.7)	88.6 (84.5 - 91.6)	82.6 (77.2 - 86.8)	71.8 (62.9 - 78.9)
Mental disorders & substance use	Anorexia nervosa	Female	55-64	248	17.74	91.3 (87.0 - 94.2)	83.2 (77.8 - 87.5)	79.8 (74.0 - 84.5)	70.3 (63.4 - 76.2)	60.8 (52.8 - 67.8)	42.5 (32.1 - 52.5)

Appendix Table A7: Unadjusted Median Survival and Survival Probabilities by Age, females (Page 13 of 18)

Group	Diagnosis Category	Sex	Age	N	Median Survival (y)	1y (%)	3y (%)	5y (%)	10y (%)	15y (%)	20y (%)
Mental disorders & substance use	Anorexia nervosa	Female	65-74	115	6.98 (8.47 - 5.09)	82.4 (74.1 - 88.3)	69.8 (60.4 - 77.4)	60.7 (50.7 - 69.2)	28.7 (18.5 - 39.7)	21.6 (12.0 - 33.0)	21.6 (12.0 - 33.0)
Mental disorders & substance use	Anorexia nervosa	Female	75-84	60	4.57 (5.56 - 1.69)	70.0 (56.7 - 79.9)	56.6 (43.1 - 68.0)	46.5 (33.0 - 58.8)	10.2 (3.1 - 22.4)	0.0 (0.0 - 0.0)	0.0 (0.0 - 0.0)
Mental disorders & substance use	Anorexia nervosa	Female	85-95	22	1.58 (4.21 - 0.69)	59.1 (36.1 - 76.2)	45.5 (24.4 - 64.3)	22.7 (8.3 - 41.4)	4.5 (0.3 - 18.9)	0.0 (0.0 - 0.0)	0.0 (0.0 - 0.0)
Mental disorders & substance use	Anxiety disorders	Female	All	559402	NR	97.6 (97.6 - 97.6)	95.2 (95.2 - 95.3)	93.1 (93.1 - 93.2)	88.4 (88.3 - 88.5)	83.9 (83.8 - 84.1)	78.9 (78.7 - 79.1)
Mental disorders & substance use	Anxiety disorders	Female	16-24	139635	NR	99.9 (99.9 - 99.9)	99.7 (99.6 - 99.7)	99.5 (99.4 - 99.5)	99.0 (98.9 - 99.0)	98.3 (98.2 - 98.4)	97.6 (97.4 - 97.8)
Mental disorders & substance use	Anxiety disorders	Female	25-34	114600	NR	99.8 (99.8 - 99.8)	99.6 (99.6 - 99.6)	99.4 (99.3 - 99.4)	98.6 (98.6 - 98.7)	97.8 (97.7 - 97.9)	96.4 (96.1 - 96.6)
Mental disorders & substance use	Anxiety disorders	Female	35-44	98057	NR	99.6 (99.6 - 99.7)	99.3 (99.2 - 99.3)	98.8 (98.7 - 98.9)	97.5 (97.4 - 97.6)	95.7 (95.6 - 95.9)	93.0 (92.7 - 93.3)
Mental disorders & substance use	Anxiety disorders	Female	45-54	78867	NR	99.1 (99.0 - 99.2)	98.0 (97.9 - 98.1)	97.1 (96.9 - 97.2)	94.2 (94.0 - 94.4)	89.9 (89.6 - 90.2)	83.3 (82.8 - 83.8)
Mental disorders & substance use	Anxiety disorders	Female	55-64	52462	NR	97.3 (97.1 - 97.4)	94.5 (94.3 - 94.7)	92.1 (91.8 - 92.3)	85.4 (85.0 - 85.7)	76.1 (75.6 - 76.6)	63.4 (62.5 - 64.2)
Mental disorders & substance use	Anxiety disorders	Female	65-74	31301	13.34 (13.55 - 13.15)	92.1 (91.8 - 92.4)	85.1 (84.6 - 85.5)	78.5 (78.0 - 78.9)	61.9 (61.3 - 62.5)	43.7 (42.9 - 44.5)	24.2 (23.1 - 25.3)
Mental disorders & substance use	Anxiety disorders	Female	75-84	28473	6.57 (6.68 - 6.47)	86.5 (86.1 - 86.9)	72.9 (72.3 - 73.4)	59.7 (59.1 - 60.3)	30.7 (30.1 - 31.4)	11.3 (10.7 - 11.9)	2.7 (2.2 - 3.2)
Mental disorders & substance use	Anxiety disorders	Female	85-95	15199	3.02 (3.09 - 2.95)	74.7 (74.0 - 75.4)	50.2 (49.3 - 51.0)	31.2 (30.4 - 32.0)	6.5 (6.0 - 7.0)	0.7 (0.5 - 1.0)	0.1 (0.0 - 0.3)
Mental disorders & substance use	Bipolar disorder	Female	All	65259	NR	98.6 (98.5 - 98.6)	96.6 (96.5 - 96.8)	94.6 (94.4 - 94.8)	89.2 (88.9 - 89.5)	82.2 (81.8 - 82.6)	72.1 (71.4 - 72.9)
Mental disorders & substance use	Bipolar disorder	Female	16-24	14202	NR	99.7 (99.6 - 99.8)	99.5 (99.3 - 99.6)	99.1 (98.9 - 99.3)	98.3 (98.0 - 98.5)	97.3 (96.9 - 97.7)	95.4 (94.2 - 96.5)
Mental disorders & substance use	Bipolar disorder	Female	25-34	15631	NR	99.8 (99.7 - 99.8)	99.3 (99.1 - 99.4)	98.8 (98.6 - 98.9)	97.7 (97.4 - 97.9)	96.3 (95.8 - 96.7)	94.9 (94.2 - 95.6)
Mental disorders & substance use	Bipolar disorder	Female	35-44	12393	NR	99.6 (99.4 - 99.7)	99.0 (98.8 - 99.2)	98.4 (98.1 - 98.6)	96.8 (96.5 - 97.1)	94.3 (93.7 - 94.8)	90.5 (89.4 - 91.6)
Mental disorders & substance use	Bipolar disorder	Female	45-54	9624	NR	99.1 (98.9 - 99.3)	98.0 (97.7 - 98.3)	96.7 (96.3 - 97.0)	93.0 (92.5 - 93.6)	87.2 (86.3 - 88.0)	77.8 (76.1 - 79.4)
Mental disorders & substance use	Bipolar disorder	Female	55-64	6456	20.64 (21.39 - 20.15)	98.0 (97.6 - 98.3)	94.6 (94.0 - 95.1)	92.1 (91.4 - 92.7)	83.8 (82.8 - 84.8)	71.7 (70.3 - 73.0)	53.0 (50.8 - 55.1)
Mental disorders & substance use	Bipolar disorder	Female	65-74	3827	12.81 (13.24 - 12.35)	95.6 (94.9 - 96.2)	89.4 (88.3 - 90.3)	82.9 (81.6 - 84.1)	63.1 (61.5 - 64.8)	38.7 (36.8 - 40.7)	17.7 (15.6 - 20.0)
Mental disorders & substance use	Bipolar disorder	Female	75-84	2410	6.63 (6.91 - 6.35)	89.3 (87.9 - 90.4)	76.8 (75.0 - 78.4)	61.6 (59.6 - 63.6)	27.7 (25.8 - 29.7)	7.6 (6.3 - 9.1)	1.4 (0.7 - 2.5)
Mental disorders & substance use	Bipolar disorder	Female	85-95	702	3.18 (3.4 - 2.74)	76.9 (73.6 - 79.9)	51.6 (47.8 - 55.3)	29.7 (26.2 - 33.3)	4.3 (2.8 - 6.3)	0.5 (0.1 - 1.5)	0.0 (0.0 - 0.0)
Mental disorders & substance use	Depressive disorders	Female	All	412237	NR	96.6 (96.5 - 96.6)	92.8 (92.8 - 92.9)	89.4 (89.3 - 89.5)	82.1 (82.0 - 82.2)	76.1 (75.9 - 76.2)	70.2 (69.9 - 70.4)
Mental disorders & substance use	Depressive disorders	Female	16-24	92756	NR	99.9 (99.8 - 99.9)	99.6 (99.6 - 99.7)	99.4 (99.4 - 99.5)	98.8 (98.8 - 98.9)	98.2 (98.1 - 98.3)	97.6 (97.4 - 97.7)
Mental disorders & substance use	Depressive disorders	Female	25-34	75806	NR	99.8 (99.8 - 99.8)	99.5 (99.5 - 99.6)	99.3 (99.2 - 99.3)	98.5 (98.4 - 98.6)	97.6 (97.4 - 97.7)	96.2 (95.9 - 96.5)
Mental disorders & substance use	Depressive disorders	Female	35-44	63832	NR	99.7 (99.6 - 99.7)	99.2 (99.1 - 99.3)	98.7 (98.6 - 98.8)	97.2 (97.1 - 97.4)	95.4 (95.2 - 95.6)	92.5 (92.1 - 92.8)
Mental disorders & substance use	Depressive disorders	Female	45-54	54475	NR	99.1 (99.0 - 99.2)	97.9 (97.8 - 98.1)	96.8 (96.6 - 97.0)	93.6 (93.3 - 93.8)	89.3 (89.0 - 89.7)	83.2 (82.6 - 83.7)
Mental disorders & substance use	Depressive disorders	Female	55-64	40394	NR	97.4 (97.3 - 97.6)	94.5 (94.3 - 94.8)	91.8 (91.5 - 92.0)	84.2 (83.8 - 84.6)	74.4 (73.9 - 74.9)	61.3 (60.5 - 62.2)
Mental disorders & substance use	Depressive disorders	Female	65-74	30706	11.98 (12.11 - 11.85)	92.7 (92.4 - 93.0)	84.8 (84.4 - 85.2)	77.1 (76.6 - 77.6)	58.0 (57.4 - 58.6)	38.0 (37.3 - 38.7)	19.9 (19.0 - 20.7)
Mental disorders & substance use	Depressive disorders	Female	75-84	34979	5.86 (5.94 - 5.79)	86.3 (85.9 - 86.7)	71.3 (70.8 - 71.7)	56.3 (55.7 - 56.8)	25.5 (25.0 - 26.0)	8.3 (7.9 - 8.7)	1.7 (1.5 - 2.0)
Mental disorders & substance use	Depressive disorders	Female	85-95	18630	2.9 (2.96 - 2.83)	74.2 (73.5 - 74.8)	48.8 (48.1 - 49.6)	29.3 (28.6 - 29.9)	5.3 (5.0 - 5.7)	0.5 (0.4 - 0.7)	0.1 (0.0 - 0.2)
Mental disorders & substance use	Drug use disorders	Female	All	72610	NR	97.1 (97.0 - 97.2)	93.7 (93.5 - 93.9)	90.6 (90.4 - 90.9)	83.7 (83.4 - 84.0)	77.2 (76.8 - 77.6)	70.7 (70.1 - 71.2)
Mental disorders & substance use	Drug use disorders	Female	16-24	23867	NR	99.4 (99.3 - 99.5)	98.7 (98.5 - 98.8)	97.9 (97.7 - 98.1)	96.1 (95.8 - 96.4)	94.5 (94.1 - 94.9)	93.0 (92.4 - 93.6)
Mental disorders & substance use	Drug use disorders	Female	25-34	13941	NR	99.1 (99.0 - 99.3)	97.7 (97.5 - 98.0)	96.7 (96.3 - 97.0)	94.1 (93.6 - 94.5)	91.3 (90.6 - 91.9)	88.0 (87.0 - 88.9)
Mental disorders & substance use	Drug use disorders	Female	35-44	10515	NR	98.5 (98.2 - 98.7)	96.8 (96.5 - 97.2)	95.2 (94.8 - 95.6)	91.2 (90.5 - 91.8)	85.6 (84.7 - 86.5)	78.0 (76.6 - 79.3)
Mental disorders & substance use	Drug use disorders	Female	45-54	9318	NR	97.9 (97.6 - 98.2)	95.3 (94.9 - 95.8)	92.3 (91.7 - 92.8)	84.7 (83.9 - 85.6)	75.6 (74.4 - 76.7)	64.6 (62.8 - 66.3)
Mental disorders & substance use	Drug use disorders	Female	55-64	6311	17.04 (17.61 - 16.37)	95.3 (94.7 - 95.8)	90.0 (89.2 - 90.8)	84.7 (83.7 - 85.6)	70.9 (69.5 - 72.1)	56.5 (54.8 - 58.1)	41.6 (39.3 - 43.8)
Mental disorders & substance use	Drug use disorders	Female	65-74	4200	10.05 (10.47 - 9.56)	91.5 (90.6 - 92.3)	81.3 (80.1 - 82.5)	72.0 (70.5 - 73.4)	50.1 (48.3 - 51.9)	31.1 (29.0 - 33.3)	16.3 (13.8 - 19.0)
Mental disorders & substance use	Drug use disorders	Female	75-84	2989	5.49 (5.83 - 5.27)	85.1 (83.8 - 86.4)	68.3 (66.5 - 70.0)	54.0 (52.0 - 55.9)	24.5 (22.6 - 26.5)	6.8 (5.4 - 8.4)	1.7 (0.9 - 3.2)
Mental disorders & substance use	Drug use disorders	Female	85-95	1408	2.7 (2.94 - 2.45)	73.6 (71.2 - 75.9)	46.9 (44.1 - 49.6)	27.4 (24.8 - 29.9)	4.6 (3.3 - 6.2)	0.4 (0.1 - 1.4)	0.0 (0.0 - 0.0)
Mental disorders & substance use	Dysthymia	Female	All	16256	NR	99.0 (98.8 - 99.1)	97.3 (97.1 - 97.6)	95.4 (95.0 - 95.7)	90.3 (89.8 - 90.8)	84.9 (84.2 - 85.5)	77.8 (76.7 - 78.9)
Mental disorders & substance use	Dysthymia	Female	16-24	3676	NR	99.9 (99.7 - 99.9)	99.6 (99.3 - 99.7)	99.3 (98.9 - 99.5)	98.3 (97.7 - 98.7)	97.4 (96.6 - 98.0)	96.9 (95.9 - 97.6)
Mental disorders & substance use	Dysthymia	Female	25-34	2844	NR	99.7 (99.4 - 99.8)	99.3 (98.9 - 99.6)	99.0 (98.6 - 99.3)	98.2 (97.6 - 98.7)	97.1 (96.2 - 97.8)	95.4 (93.7 - 96.6)
Mental disorders & substance use	Dysthymia	Female	35-44	2854	NR	99.7 (99.4 - 99.9)	99.3 (98.9 - 99.5)	98.6 (98.1 - 99.0)	97.2 (96.5 - 97.8)	95.0 (94.0 - 95.9)	92.0 (90.2 - 93.5)
Mental disorders & substance use	Dysthymia	Female	45-54	3014	NR	99.6 (99.3 - 99.8)	99.1 (98.7 - 99.4)	98.1 (97.5 - 98.5)	95.0 (94.1 - 95.8)	90.7 (89.3 - 91.9)	84.8 (82.5 - 86.8)
Mental disorders & substance use	Dysthymia	Female	55-64	2153	NR	98.9 (98.4 - 99.3)	97.1 (96.3 - 97.7)	95.0 (93.9 - 95.8)	88.3 (86.7 - 89.7)	79.5 (77.3 - 81.5)	64.5 (60.8 - 67.8)
Mental disorders & substance use	Dysthymia	Female	65-74	909	13.57 (14.72 - 12.71)	97.3 (96.0 - 98.2)	91.4 (89.3 - 93.0)	85.1 (82.6 - 87.4)	65.9 (62.4 - 69.2)	44.2 (40.1 - 48.2)	20.8 (16.4 - 25.6)
Mental disorders & substance use	Dysthymia	Female	75-84	618	7.05 (7.51 - 6.57)	93.4 (91.2 - 95.2)	80.3 (76.9 - 83.3)	65.9 (61.9 - 69.6)	33.6 (29.6 - 37.6)	13.3 (10.3 - 16.7)	2.6 (1.0 - 5.5)
Mental disorders & substance use	Dysthymia	Female	85-95	185	3.47 (4.01 - 3.01)	79.2 (72.6 - 84.4)	58.0 (50.4 - 64.8)	34.0 (27.1 - 41.0)	6.6 (3.4 - 11.2)	1.3 (0.1 - 5.7)	1.3 (0.1 - 5.7)
Mental disorders & substance use	Major depression	Female	All	408504	NR	96.5 (96.5 - 96.6)	92.8 (92.7 - 92.9)	89.3 (89.2 - 89.4)	82.0 (81.9 - 82.1)	75.9 (75.7 - 76.1)	70.0 (69.8 - 70.3)
Mental disorders & substance use	Major depression	Female	16-24	91763	NR	99.9 (99.8 - 99.9)	99.6 (99.6 - 99.7)	99.4 (99.4 - 99.5)	98.8 (98.8 - 98.9)	98.2 (98.1 - 98.3)	97.5 (97.3 - 97.7)

Appendix Table A7: Unadjusted Median Survival and Survival Probabilities by Age, females (Page 14 of 18)

Group	Diagnosis Category	Sex	Age	N	Median Survival (y)	1y (%)	3y (%)	5y (%)	10y (%)	15y (%)	20y (%)
Mental disorders & substance use	Major depression	Female	25-34	75260	NR	99.8 (99.8 - 99.8)	99.5 (99.5 - 99.6)	99.3 (99.2 - 99.3)	98.5 (98.4 - 98.6)	97.6 (97.4 - 97.7)	96.2 (95.9 - 96.5)
Mental disorders & substance use	Major depression	Female	35-44	63162	NR	99.7 (99.6 - 99.7)	99.2 (99.1 - 99.3)	98.7 (98.6 - 98.8)	97.2 (97.1 - 97.4)	95.3 (95.1 - 95.5)	92.4 (92.1 - 92.8)
Mental disorders & substance use	Major depression	Female	45-54	53752	NR	99.1 (99.0 - 99.2)	97.9 (97.8 - 98.0)	96.8 (96.6 - 96.9)	93.5 (93.3 - 93.8)	89.3 (89.0 - 89.6)	83.1 (82.5 - 83.6)
Mental disorders & substance use	Major depression	Female	55-64	39951	NR	97.4 (97.3 - 97.6)	94.5 (94.3 - 94.7)	91.7 (91.4 - 92.0)	84.0 (83.6 - 84.4)	74.2 (73.7 - 74.7)	61.1 (60.3 - 62.0)
Mental disorders & substance use	Major depression	Female	65-74	30526	11.95 (12.09 - 11.81)	92.7 (92.4 - 93.0)	84.8 (84.3 - 85.2)	77.0 (76.5 - 77.5)	57.9 (57.2 - 58.5)	37.9 (37.2 - 38.6)	19.9 (19.0 - 20.7)
Mental disorders & substance use	Major depression	Female	75-84	34858	5.85 (5.93 - 5.78)	86.3 (85.9 - 86.6)	71.2 (70.7 - 71.7)	56.2 (55.7 - 56.8)	25.4 (24.9 - 26.0)	8.2 (7.8 - 8.6)	1.7 (1.4 - 2.0)
Mental disorders & substance use	Major depression	Female	85-95	18575	2.89 (2.96 - 2.83)	74.1 (73.5 - 74.8)	48.8 (48.0 - 49.5)	29.2 (28.6 - 29.9)	5.3 (5.0 - 5.7)	0.5 (0.4 - 0.7)	0.1 (0.0 - 0.2)
Mental disorders & substance use	Opioid use disorders	Female	All	17839	NR	95.0 (94.7 - 95.3)	89.8 (89.4 - 90.3)	85.6 (85.0 - 86.1)	77.1 (76.4 - 77.8)	69.3 (68.4 - 70.2)	62.1 (60.7 - 63.4)
Mental disorders & substance use	Opioid use disorders	Female	16-24	3527	NR	99.0 (98.6 - 99.2)	96.7 (96.0 - 97.2)	95.1 (94.3 - 95.8)	91.6 (90.4 - 92.6)	88.1 (86.6 - 89.5)	86.0 (84.0 - 87.8)
Mental disorders & substance use	Opioid use disorders	Female	25-34	3940	NR	98.3 (97.9 - 98.7)	96.2 (95.5 - 96.7)	94.1 (93.2 - 94.8)	90.6 (89.4 - 91.6)	85.4 (83.7 - 86.9)	80.4 (77.8 - 82.7)
Mental disorders & substance use	Opioid use disorders	Female	35-44	3191	NR	98.0 (97.4 - 98.4)	95.5 (94.7 - 96.2)	93.3 (92.3 - 94.1)	87.6 (86.3 - 88.9)	81.1 (79.2 - 82.9)	73.2 (70.4 - 75.8)
Mental disorders & substance use	Opioid use disorders	Female	45-54	2708	22.94	97.1 (96.4 - 97.7)	93.8 (92.8 - 94.7)	89.7 (88.4 - 90.8)	81.4 (79.6 - 83.0)	70.0 (67.4 - 72.3)	57.5 (53.6 - 61.2)
Mental disorders & substance use	Opioid use disorders	Female	55-64	1785	15.63 (17.05 - 14.53)	93.2 (91.9 - 94.2)	87.0 (85.3 - 88.5)	80.9 (78.9 - 82.7)	65.6 (62.9 - 68.2)	52.7 (49.4 - 56.0)	37.9 (33.1 - 42.6)
Mental disorders & substance use	Opioid use disorders	Female	65-74	1240	8.13 (9.04 - 7.59)	88.8 (86.9 - 90.4)	75.7 (73.0 - 78.1)	66.5 (63.5 - 69.2)	42.2 (38.6 - 45.8)	25.6 (21.5 - 29.9)	16.5 (11.1 - 22.8)
Mental disorders & substance use	Opioid use disorders	Female	75-84	869	4.19 (4.92 - 3.78)	80.5 (77.6 - 83.0)	60.6 (57.0 - 63.9)	45.3 (41.6 - 48.9)	21.0 (17.5 - 24.6)	4.0 (2.0 - 7.2)	0.0 (0.0 - 0.0)
Mental disorders & substance use	Opioid use disorders	Female	85-95	544	1.81 (2.15 - 1.54)	62.8 (58.5 - 66.8)	36.5 (32.2 - 40.7)	19.8 (16.3 - 23.7)	2.1 (0.8 - 4.6)	0.0 (0.0 - 0.0)	0.0 (0.0 - 0.0)
Mental disorders & substance use	Schizophrenia	Female	All	18640	22.1 (22.73 - 21.58)	95.8 (95.5 - 96.0)	91.1 (90.7 - 91.5)	86.7 (86.2 - 87.2)	75.7 (75.0 - 76.3)	64.9 (64.1 - 65.7)	54.6 (53.6 - 55.6)
Mental disorders & substance use	Schizophrenia	Female	16-24	1457	NR	99.7 (99.2 - 99.9)	98.7 (98.0 - 99.2)	98.0 (97.1 - 98.6)	96.3 (95.1 - 97.3)	94.6 (93.0 - 95.9)	93.0 (90.9 - 94.7)
Mental disorders & substance use	Schizophrenia	Female	25-34	2652	NR	99.4 (99.0 - 99.6)	98.5 (97.9 - 98.9)	97.9 (97.2 - 98.4)	96.1 (95.1 - 96.8)	94.5 (93.3 - 95.4)	90.7 (88.8 - 92.3)
Mental disorders & substance use	Schizophrenia	Female	35-44	3369	NR	99.3 (99.0 - 99.6)	98.4 (97.9 - 98.8)	97.2 (96.6 - 97.7)	94.1 (93.2 - 94.9)	89.1 (87.9 - 90.3)	82.5 (80.7 - 84.2)
Mental disorders & substance use	Schizophrenia	Female	45-54	3755	NR	98.7 (98.3 - 99.0)	96.7 (96.0 - 97.2)	94.8 (94.0 - 95.5)	87.5 (86.3 - 88.6)	77.9 (76.4 - 79.4)	65.2 (63.0 - 67.2)
Mental disorders & substance use	Schizophrenia	Female	55-64	3374	16.56 (17.23 - 15.97)	96.3 (95.6 - 96.9)	91.6 (90.6 - 92.5)	86.7 (85.5 - 87.9)	72.5 (70.8 - 74.0)	55.3 (53.4 - 57.2)	39.0 (36.7 - 41.3)
Mental disorders & substance use	Schizophrenia	Female	65-74	2206	9.66 (10.27 - 9.22)	91.5 (90.3 - 92.6)	82.9 (81.2 - 84.4)	72.9 (71.0 - 74.8)	48.7 (46.5 - 50.9)	25.5 (23.4 - 27.6)	9.6 (7.8 - 11.6)
Mental disorders & substance use	Schizophrenia	Female	75-84	1411	4.83 (5.23 - 4.45)	82.3 (80.2 - 84.2)	63.9 (61.3 - 66.4)	48.7 (46.0 - 51.3)	18.6 (16.5 - 20.8)	5.2 (3.9 - 6.7)	1.3 (0.6 - 2.7)
Mental disorders & substance use	Schizophrenia	Female	85-95	406	2.43 (2.76 - 2.06)	67.6 (62.8 - 72.0)	43.0 (38.1 - 47.8)	23.6 (19.5 - 27.9)	3.9 (2.2 - 6.4)	0.8 (0.2 - 2.6)	0.0 (0.0 - 0.0)
Mental disorders & substance use	Self-harm	Female	All	76714	NR	97.3 (97.2 - 97.4)	94.5 (94.3 - 94.7)	91.9 (91.7 - 92.1)	86.5 (86.2 - 86.8)	81.8 (81.5 - 82.1)	76.8 (76.4 - 77.3)
Mental disorders & substance use	Self-harm	Female	16-24	29446	NR	99.5 (99.4 - 99.6)	98.8 (98.7 - 99.0)	98.3 (98.2 - 98.5)	97.2 (97.0 - 97.4)	96.1 (95.8 - 96.3)	95.0 (94.6 - 95.4)
Mental disorders & substance use	Self-harm	Female	25-34	13395	NR	98.8 (98.6 - 99.0)	97.8 (97.5 - 98.0)	96.9 (96.6 - 97.2)	95.0 (94.6 - 95.4)	93.4 (92.9 - 93.9)	91.0 (90.3 - 91.7)
Mental disorders & substance use	Self-harm	Female	35-44	10623	NR	98.2 (97.9 - 98.4)	96.5 (96.1 - 96.8)	95.1 (94.7 - 95.5)	91.7 (91.1 - 92.2)	87.6 (86.8 - 88.3)	82.6 (81.5 - 83.6)
Mental disorders & substance use	Self-harm	Female	45-54	9585	NR	97.5 (97.2 - 97.8)	95.2 (94.7 - 95.6)	92.7 (92.1 - 93.2)	86.6 (85.8 - 87.3)	79.0 (77.9 - 80.0)	69.4 (67.9 - 70.9)
Mental disorders & substance use	Self-harm	Female	55-64	5789	18.95 (19.88 - 18.36)	94.5 (93.8 - 95.0)	89.5 (88.7 - 90.3)	84.7 (83.7 - 85.7)	73.5 (72.2 - 74.7)	61.9 (60.3 - 63.5)	47.5 (45.4 - 49.5)
Mental disorders & substance use	Self-harm	Female	65-74	3517	10.62 (11.13 - 10.22)	91.0 (90.0 - 91.9)	81.7 (80.3 - 83.0)	72.7 (71.1 - 74.2)	52.7 (50.8 - 54.6)	33.3 (31.2 - 35.4)	18.8 (16.5 - 21.3)
Mental disorders & substance use	Self-harm	Female	75-84	2811	5.94 (6.1 - 5.73)	86.3 (85.0 - 87.5)	71.9 (70.2 - 73.6)	57.3 (55.3 - 59.2)	24.3 (22.4 - 26.3)	8.6 (7.2 - 10.2)	1.6 (0.8 - 2.8)
Mental disorders & substance use	Self-harm	Female	85-95	1490	3.37 (3.56 - 3.11)	78.8 (76.6 - 80.8)	54.3 (51.7 - 56.9)	34.0 (31.3 - 36.6)	6.7 (5.2 - 8.4)	0.2 (0.0 - 1.0)	0.0 (0.0 - 0.0)
Musculoskeletal disorders	Gout	Female	All	41047	5.59 (5.69 - 5.49)	83.6 (83.2 - 83.9)	66.6 (66.1 - 67.1)	53.4 (52.9 - 53.9)	31.3 (30.7 - 31.8)	19.5 (18.9 - 20.0)	13.4 (12.8 - 14.1)
Musculoskeletal disorders	Gout	Female	16-24	137	NR	100.0 (100.0 - 100.0)	100.0 (100.0 - 100.0)	100.0 (100.0 - 100.0)	100.0 (100.0 - 100.0)	100.0 (100.0 - 100.0)	98.2 (88.2 - 99.8)
Musculoskeletal disorders	Gout	Female	25-34	335	NR	99.4 (97.5 - 99.8)	98.7 (96.7 - 99.5)	97.4 (94.8 - 98.7)	94.4 (90.8 - 96.6)	91.0 (86.0 - 94.2)	86.4 (79.4 - 91.2)
Musculoskeletal disorders	Gout	Female	35-44	621	NR	99.3 (98.3 - 99.8)	98.3 (96.9 - 99.1)	97.6 (95.9 - 98.5)	93.2 (90.6 - 95.2)	89.4 (85.6 - 92.2)	85.0 (80.0 - 88.9)
Musculoskeletal disorders	Gout	Female	45-54	1523	NR	98.4 (97.6 - 98.9)	95.6 (94.4 - 96.6)	92.8 (91.3 - 94.0)	86.2 (84.1 - 88.1)	78.3 (75.3 - 81.0)	69.3 (65.0 - 73.2)
Musculoskeletal disorders	Gout	Female	55-64	3282	17.48 (18.18 - 16.83)	96.5 (95.8 - 97.1)	91.4 (90.3 - 92.3)	86.4 (85.2 - 87.6)	73.3 (71.5 - 75.0)	57.6 (55.3 - 59.9)	41.8 (38.6 - 45.0)
Musculoskeletal disorders	Gout	Female	65-74	8281	10.13 (10.46 - 9.83)	92.7 (92.1 - 93.2)	83.1 (82.2 - 83.9)	73.4 (72.4 - 74.4)	50.5 (49.2 - 51.8)	29.8 (28.3 - 31.4)	16.1 (14.3 - 18.0)
Musculoskeletal disorders	Gout	Female	75-84	14490	5.34 (5.46 - 5.21)	85.2 (84.6 - 85.8)	67.9 (67.1 - 68.7)	52.5 (51.6 - 53.4)	22.0 (21.2 - 22.9)	6.4 (5.8 - 7.1)	1.1 (0.7 - 1.7)
Musculoskeletal disorders	Gout	Female	85-95	11607	2.38 (2.45 - 2.31)	70.1 (69.3 - 71.0)	42.4 (41.4 - 43.3)	24.1 (23.3 - 25.0)	3.9 (3.5 - 4.4)	0.4 (0.2 - 0.6)	0.0 (0.0 - 0.0)
Musculoskeletal disorders	Low back pain	Female	All	304031	NR	97.9 (97.8 - 97.9)	94.7 (94.7 - 94.8)	91.3 (91.2 - 91.4)	82.8 (82.7 - 83.0)	74.7 (74.5 - 74.9)	67.4 (67.2 - 67.7)
Musculoskeletal disorders	Low back pain	Female	16-24	17874	NR	99.9 (99.9 - 100.0)	99.8 (99.8 - 99.9)	99.7 (99.6 - 99.8)	99.5 (99.4 - 99.6)	99.1 (99.0 - 99.3)	98.7 (98.3 - 98.9)
Musculoskeletal disorders	Low back pain	Female	25-34	35542	NR	99.9 (99.8 - 99.9)	99.7 (99.6 - 99.7)	99.5 (99.4 - 99.6)	99.1 (99.0 - 99.2)	98.5 (98.3 - 98.6)	97.4 (97.1 - 97.7)
Musculoskeletal disorders	Low back pain	Female	35-44	49543	NR	99.8 (99.7 - 99.8)	99.5 (99.4 - 99.6)	99.2 (99.2 - 99.3)	98.4 (98.3 - 98.5)	97.2 (97.0 - 97.4)	95.5 (95.1 - 95.8)
Musculoskeletal disorders	Low back pain	Female	45-54	55447	NR	99.5 (99.5 - 99.6)	99.0 (98.9 - 99.1)	98.4 (98.3 - 98.5)	96.5 (96.3 - 96.7)	93.6 (93.3 - 93.8)	89.1 (88.6 - 89.6)
Musculoskeletal disorders	Low back pain	Female	55-64	49992	NR	98.8 (98.7 - 98.9)	97.2 (97.1 - 97.4)	95.6 (95.4 - 95.8)	90.5 (90.2 - 90.8)	83.0 (82.6 - 83.4)	71.9 (71.2 - 72.7)
Musculoskeletal disorders	Low back pain	Female	65-74	43092	16.37 (16.53 - 16.18)	97.1 (97.0 - 97.3)	93.4 (93.1 - 93.6)	89.0 (88.7 - 89.3)	75.5 (75.0 - 76.0)	55.9 (55.2 - 56.6)	32.8 (31.8 - 33.7)

Appendix Table A7: Unadjusted Median Survival and Survival Probabilities by Age, females (Page 15 of 18)

Group	Diagnosis Category	Sex	Age	N	Median Survival (y)	1y (%)	3y (%)	5y (%)	10y (%)	15y (%)	20y (%)
Musculoskeletal disorders	Low back pain	Female	75-84	37095	8.9 (9.0 - 8.82)	94.5 (94.3 - 94.7)	85.4 (85.0 - 85.8)	74.4 (73.9 - 74.9)	43.2 (42.6 - 43.8)	17.3 (16.7 - 17.9)	4.6 (4.1 - 5.1)
Musculoskeletal disorders	Low back pain	Female	85-95	14953	4.48 (4.56 - 4.39)	86.5 (85.9 - 87.0)	65.7 (64.9 - 66.5)	44.7 (43.9 - 45.6)	11.1 (10.5 - 11.7)	1.3 (1.0 - 1.6)	0.0 (0.0 - 0.0)
Musculoskeletal disorders	Lupus	Female	All	13982	NR	97.3 (97.0 - 97.6)	93.9 (93.4 - 94.2)	90.6 (90.0 - 91.1)	81.7 (81.0 - 82.4)	73.0 (72.1 - 73.9)	63.7 (62.6 - 64.8)
Musculoskeletal disorders	Lupus	Female	16-24	982	NR	99.8 (99.2 - 99.9)	99.6 (98.8 - 99.8)	99.6 (98.8 - 99.8)	99.4 (98.6 - 99.8)	98.8 (97.4 - 99.4)	98.4 (96.7 - 99.2)
Musculoskeletal disorders	Lupus	Female	25-34	1874	NR	99.6 (99.1 - 99.8)	99.5 (99.0 - 99.7)	99.1 (98.6 - 99.5)	98.6 (97.8 - 99.0)	97.7 (96.7 - 98.4)	96.2 (94.7 - 97.3)
Musculoskeletal disorders	Lupus	Female	35-44	2242	NR	99.9 (99.6 - 100.0)	99.5 (99.1 - 99.7)	99.1 (98.6 - 99.5)	97.6 (96.8 - 98.2)	95.3 (94.1 - 96.3)	91.6 (89.9 - 93.1)
Musculoskeletal disorders	Lupus	Female	45-54	2453	NR	99.3 (98.8 - 99.5)	98.2 (97.5 - 98.6)	97.0 (96.2 - 97.6)	93.5 (92.4 - 94.5)	88.1 (86.5 - 89.5)	78.8 (76.6 - 80.9)
Musculoskeletal disorders	Lupus	Female	55-64	2575	22.14	98.5 (98.0 - 98.9)	96.0 (95.1 - 96.7)	92.5 (91.4 - 93.5)	84.4 (82.8 - 85.8)	73.1 (71.0 - 75.0)	59.6 (57.0 - 62.2)
Musculoskeletal disorders	Lupus	Female	65-74	2207	13.84 (14.57 - 13.47)	95.1 (94.2 - 96.0)	89.3 (87.9 - 90.5)	83.7 (82.0 - 85.2)	65.9 (63.6 - 68.0)	45.8 (43.1 - 48.5)	22.3 (19.3 - 25.5)
Musculoskeletal disorders	Lupus	Female	75-84	1296	7.25 (7.8 - 6.67)	90.2 (88.4 - 91.7)	76.7 (74.2 - 78.9)	65.4 (62.6 - 68.1)	34.8 (31.8 - 37.7)	12.8 (10.5 - 15.3)	2.4 (1.3 - 4.1)
Musculoskeletal disorders	Lupus	Female	85-95	348	3.8 (4.39 - 3.14)	78.9 (74.2 - 82.8)	58.4 (53.0 - 63.5)	40.7 (35.3 - 46.0)	6.5 (3.8 - 10.1)	0.8 (0.1 - 3.7)	0.0 (0.0 - 0.0)
Musculoskeletal disorders	Neck pain	Female	All	140516	NR	99.0 (98.9 - 99.0)	97.4 (97.3 - 97.5)	95.7 (95.6 - 95.9)	91.2 (91.0 - 91.4)	86.0 (85.8 - 86.3)	80.4 (80.0 - 80.8)
Musculoskeletal disorders	Neck pain	Female	16-24	9248	NR	99.9 (99.9 - 100.0)	99.9 (99.8 - 99.9)	99.7 (99.6 - 99.8)	99.5 (99.3 - 99.6)	99.0 (98.7 - 99.3)	98.8 (98.4 - 99.1)
Musculoskeletal disorders	Neck pain	Female	25-34	16685	NR	99.9 (99.9 - 100.0)	99.8 (99.7 - 99.8)	99.6 (99.5 - 99.7)	99.2 (99.0 - 99.4)	98.7 (98.4 - 98.9)	97.8 (97.4 - 98.2)
Musculoskeletal disorders	Neck pain	Female	35-44	29346	NR	99.9 (99.8 - 99.9)	99.6 (99.6 - 99.7)	99.4 (99.3 - 99.5)	98.7 (98.5 - 98.8)	97.6 (97.3 - 97.8)	95.9 (95.5 - 96.3)
Musculoskeletal disorders	Neck pain	Female	45-54	35836	NR	99.7 (99.7 - 99.8)	99.3 (99.2 - 99.4)	98.9 (98.7 - 99.0)	97.2 (96.9 - 97.4)	94.7 (94.3 - 95.0)	90.7 (90.1 - 91.3)
Musculoskeletal disorders	Neck pain	Female	55-64	24634	NR	99.3 (99.2 - 99.4)	98.1 (97.9 - 98.3)	96.9 (96.7 - 97.1)	92.9 (92.5 - 93.3)	86.4 (85.8 - 87.0)	76.0 (74.9 - 77.1)
Musculoskeletal disorders	Neck pain	Female	65-74	13342	17.28 (17.51 - 17.0)	97.8 (97.5 - 98.0)	94.8 (94.3 - 95.1)	91.3 (90.7 - 91.8)	79.1 (78.2 - 79.9)	60.5 (59.2 - 61.8)	37.3 (35.2 - 39.4)
Musculoskeletal disorders	Neck pain	Female	75-84	8600	9.82 (10.01 - 9.54)	95.4 (94.9 - 95.8)	86.8 (86.1 - 87.6)	77.1 (76.1 - 78.0)	48.7 (47.3 - 50.1)	20.8 (19.3 - 22.4)	5.8 (4.5 - 7.4)
Musculoskeletal disorders	Neck pain	Female	85-95	2737	4.42 (4.6 - 4.17)	84.6 (83.2 - 86.0)	63.7 (61.7 - 65.5)	44.1 (42.1 - 46.2)	12.0 (10.4 - 13.7)	1.9 (1.0 - 3.3)	0.0 (0.0 - 0.0)
Musculoskeletal disorders	Osteoarthritis	Female	All	479058	20.09 (20.18 - 20.0)	98.4 (98.4 - 98.5)	95.3 (95.2 - 95.3)	91.4 (91.3 - 91.5)	79.4 (79.3 - 79.5)	65.0 (64.8 - 65.2)	50.3 (50.0 - 50.5)
Musculoskeletal disorders	Osteoarthritis	Female	16-24	1738	NR	100.0 (100.0 - 100.0)	99.7 (99.3 - 99.9)	99.3 (98.8 - 99.6)	99.1 (98.5 - 99.5)	98.8 (98.1 - 99.3)	98.8 (98.1 - 99.3)
Musculoskeletal disorders	Osteoarthritis	Female	25-34	4872	NR	99.9 (99.8 - 100.0)	99.7 (99.5 - 99.8)	99.6 (99.3 - 99.7)	98.9 (98.5 - 99.2)	98.0 (97.5 - 98.5)	96.9 (96.0 - 97.6)
Musculoskeletal disorders	Osteoarthritis	Female	35-44	19002	NR	99.9 (99.8 - 99.9)	99.7 (99.6 - 99.7)	99.3 (99.2 - 99.4)	98.4 (98.2 - 98.6)	97.2 (96.9 - 97.5)	95.1 (94.6 - 95.6)
Musculoskeletal disorders	Osteoarthritis	Female	45-54	67876	NR	99.8 (99.8 - 99.9)	99.4 (99.3 - 99.4)	98.8 (98.7 - 98.9)	97.0 (96.9 - 97.2)	93.9 (93.7 - 94.2)	89.0 (88.5 - 89.4)
Musculoskeletal disorders	Osteoarthritis	Female	55-64	126690	NR	99.7 (99.6 - 99.7)	98.7 (98.7 - 98.8)	97.6 (97.5 - 97.7)	93.6 (93.5 - 93.8)	86.8 (86.5 - 87.0)	75.4 (75.0 - 75.9)
Musculoskeletal disorders	Osteoarthritis	Female	65-74	135386	18.12 (18.19 - 18.03)	99.2 (99.1 - 99.2)	97.1 (97.0 - 97.2)	94.3 (94.2 - 94.4)	83.5 (83.3 - 83.7)	65.1 (64.7 - 65.5)	40.1 (39.5 - 40.6)
Musculoskeletal disorders	Osteoarthritis	Female	75-84	96758	10.82 (10.88 - 10.77)	97.4 (97.3 - 97.5)	91.6 (91.4 - 91.7)	83.7 (83.4 - 83.9)	55.4 (55.0 - 55.8)	24.8 (24.5 - 25.2)	6.4 (6.1 - 6.7)
Musculoskeletal disorders	Osteoarthritis	Female	85-95	25770	5.08 (5.16 - 5.02)	88.2 (87.8 - 88.6)	69.8 (69.2 - 70.3)	50.8 (50.1 - 51.4)	15.2 (14.7 - 15.7)	2.3 (2.0 - 2.6)	0.2 (0.1 - 0.3)
Musculoskeletal disorders	Rheumatoid arthritis	Female	All	84498	21.05 (21.33 - 20.79)	96.5 (96.3 - 96.6)	91.5 (91.3 - 91.7)	86.6 (86.3 - 86.8)	74.4 (74.1 - 74.7)	62.6 (62.2 - 63.0)	52.0 (51.5 - 52.4)
Musculoskeletal disorders	Rheumatoid arthritis	Female	16-24	3140	NR	100.0 (100.0 - 100.0)	100.0 (99.8 - 100.0)	99.9 (99.7 - 100.0)	99.6 (99.3 - 99.8)	99.2 (98.7 - 99.5)	99.1 (98.4 - 99.4)
Musculoskeletal disorders	Rheumatoid arthritis	Female	25-34	5280	NR	99.9 (99.8 - 100.0)	99.9 (99.8 - 100.0)	99.7 (99.5 - 99.8)	99.3 (99.0 - 99.5)	98.9 (98.5 - 99.2)	97.9 (97.2 - 98.4)
Musculoskeletal disorders	Rheumatoid arthritis	Female	35-44	8024	NR	99.8 (99.7 - 99.9)	99.7 (99.5 - 99.8)	99.3 (99.1 - 99.5)	98.3 (97.9 - 98.6)	96.4 (95.9 - 96.9)	94.1 (93.3 - 94.8)
Musculoskeletal disorders	Rheumatoid arthritis	Female	45-54	12694	NR	99.5 (99.4 - 99.6)	98.8 (98.6 - 99.0)	97.9 (97.6 - 98.2)	95.5 (95.1 - 95.9)	91.9 (91.3 - 92.4)	85.3 (84.4 - 86.2)
Musculoskeletal disorders	Rheumatoid arthritis	Female	55-64	18347	NR	99.0 (98.9 - 99.2)	97.3 (97.0 - 97.5)	95.1 (94.7 - 95.4)	88.4 (87.9 - 88.9)	78.1 (77.4 - 78.8)	64.4 (63.4 - 65.4)
Musculoskeletal disorders	Rheumatoid arthritis	Female	65-74	18026	14.43 (14.64 - 14.22)	97.2 (96.9 - 97.4)	92.6 (92.2 - 93.0)	87.4 (86.9 - 87.9)	69.8 (69.1 - 70.6)	47.3 (46.3 - 48.2)	24.1 (23.1 - 25.2)
Musculoskeletal disorders	Rheumatoid arthritis	Female	75-84	14250	7.61 (7.78 - 7.48)	92.3 (91.9 - 92.7)	80.3 (79.7 - 81.0)	67.6 (66.8 - 68.4)	35.4 (34.5 - 36.3)	12.0 (11.3 - 12.8)	1.6 (1.2 - 2.0)
Musculoskeletal disorders	Rheumatoid arthritis	Female	85-95	4597	3.06 (3.2 - 2.91)	77.2 (76.0 - 78.4)	50.7 (49.2 - 52.1)	30.9 (29.5 - 32.3)	7.3 (6.5 - 8.2)	0.8 (0.5 - 1.3)	0.0 (0.0 - 0.0)
Neurological disorders	Alzheimer's disease	Female	All	80211	4.44 (4.48 - 4.4)	85.1 (84.9 - 85.4)	64.7 (64.4 - 65.0)	44.7 (44.3 - 45.1)	12.7 (12.4 - 13.0)	3.0 (2.8 - 3.1)	1.0 (0.8 - 1.1)
Neurological disorders	Alzheimer's disease	Female	16-24	5	NR	100.0 (100.0 - 100.0)	100.0 (100.0 - 100.0)	100.0 (100.0 - 100.0)	100.0 (100.0 - 100.0)	100.0 (100.0 - 100.0)	100.0 (100.0 - 100.0)
Neurological disorders	Alzheimer's disease	Female	25-34	10	NR	100.0 (100.0 - 100.0)	100.0 (100.0 - 100.0)	100.0 (100.0 - 100.0)	100.0 (100.0 - 100.0)	100.0 (100.0 - 100.0)	100.0 (100.0 - 100.0)
Neurological disorders	Alzheimer's disease	Female	35-44	38	17.18	97.4 (82.8 - 99.6)	94.6 (80.0 - 98.6)	83.5 (66.8 - 92.2)	55.9 (35.4 - 72.2)	50.8 (30.2 - 68.1)	43.5 (22.3 - 63.0)
Neurological disorders	Alzheimer's disease	Female	45-54	496	10.12 (11.37 - 9.12)	97.8 (96.0 - 98.8)	92.0 (89.1 - 94.1)	83.7 (79.9 - 86.9)	50.7 (45.1 - 56.0)	28.7 (23.2 - 34.4)	18.9 (13.1 - 25.5)
Neurological disorders	Alzheimer's disease	Female	55-64	3064	9.06 (9.38 - 8.83)	97.5 (96.8 - 98.0)	92.0 (90.9 - 92.9)	81.1 (79.6 - 82.6)	43.4 (41.2 - 45.6)	17.5 (15.6 - 19.5)	8.8 (7.1 - 10.7)
Neurological disorders	Alzheimer's disease	Female	65-74	14633	6.94 (7.03 - 6.84)	95.1 (94.7 - 95.4)	84.1 (83.4 - 84.7)	67.8 (67.0 - 68.7)	26.7 (25.8 - 27.6)	7.1 (6.5 - 7.8)	1.4 (1.0 - 1.9)
Neurological disorders	Alzheimer's disease	Female	75-84	37511	4.67 (4.73 - 4.62)	88.0 (87.7 - 88.3)	68.2 (67.7 - 68.7)	46.7 (46.1 - 47.2)	11.0 (10.6 - 11.4)	1.5 (1.4 - 1.7)	0.3 (0.2 - 0.4)
Neurological disorders	Alzheimer's disease	Female	85-95	23823	2.59 (2.63 - 2.53)	73.6 (73.0 - 74.2)	44.3 (43.6 - 45.0)	22.7 (22.1 - 23.3)	2.1 (1.8 - 2.3)	0.1 (0.1 - 0.2)	0.1 (0.0 - 0.2)
Neurological disorders	Alzheimer's disease and other dementias	Female	All	218999	2.94 (2.96 - 2.92)	73.6 (73.4 - 73.8)	49.3 (49.1 - 49.5)	30.8 (30.6 - 31.1)	8.2 (8.0 - 8.3)	2.4 (2.3 - 2.5)	1.1 (1.0 - 1.1)
Neurological disorders	Alzheimer's disease and other dementias	Female	16-24	70	NR	98.6 (90.3 - 99.8)	97.0 (88.6 - 99.3)	95.4 (86.3 - 98.5)	93.6 (83.6 - 97.5)	90.6 (78.1 - 96.2)	90.6 (78.1 - 96.2)
Neurological disorders	Alzheimer's disease and other dementias	Female	25-34	132	NR	98.5 (94.1 - 99.6)	97.7 (93.0 - 99.3)	95.3 (89.7 - 97.8)	92.5 (86.0 - 96.0)	89.5 (81.4 - 94.2)	86.4 (75.3 - 92.8)

Appendix Table A7: Unadjusted Median Survival and Survival Probabilities by Age, females (Page 16 of 18)

Group	Diagnosis Category	Sex	Age	N	Median Survival (y)	1y (%)	3y (%)	5y (%)	10y (%)	15y (%)	20y (%)
Neurological disorders	Alzheimer's disease and other dementias	Female	35-44	290	NR	97.6 (95.0 - 98.8)	94.7 (91.3 - 96.7)	89.3 (84.9 - 92.4)	73.4 (67.2 - 78.6)	65.6 (58.7 - 71.6)	59.7 (51.5 - 67.0)
Neurological disorders	Alzheimer's disease and other dementias	Female	45-54	1275	13.55 (14.89 - 12.09)	95.6 (94.4 - 96.6)	88.6 (86.7 - 90.3)	82.4 (80.1 - 84.5)	60.8 (57.6 - 63.8)	45.5 (41.9 - 49.0)	37.2 (33.0 - 41.3)
Neurological disorders	Alzheimer's disease and other dementias	Female	55-64	5772	8.99 (9.24 - 8.79)	94.2 (93.5 - 94.7)	86.2 (85.3 - 87.1)	75.2 (74.0 - 76.4)	44.2 (42.7 - 45.7)	22.3 (20.8 - 23.8)	12.7 (11.2 - 14.2)
Neurological disorders	Alzheimer's disease and other dementias	Female	65-74	26173	6.15 (6.22 - 6.06)	90.5 (90.2 - 90.9)	76.7 (76.1 - 77.2)	59.8 (59.1 - 60.4)	23.5 (22.9 - 24.1)	6.9 (6.4 - 7.4)	1.6 (1.3 - 2.0)
Neurological disorders	Alzheimer's disease and other dementias	Female	75-84	87783	3.69 (3.72 - 3.65)	80.2 (79.9 - 80.4)	57.5 (57.1 - 57.8)	36.7 (36.4 - 37.1)	7.9 (7.7 - 8.1)	1.1 (1.0 - 1.2)	0.2 (0.1 - 0.2)
Neurological disorders	Alzheimer's disease and other dementias	Female	85-95	92218	1.75 (1.77 - 1.73)	62.6 (62.3 - 62.9)	32.7 (32.4 - 33.1)	14.8 (14.5 - 15.0)	1.1 (1.0 - 1.2)	0.1 (0.0 - 0.1)	0.0 (0.0 - 0.0)
Neurological disorders	Dementia as a sign with others	Female	All	70493	4.53 (4.57 - 4.49)	86.4 (86.2 - 86.7)	66.0 (65.6 - 66.3)	45.4 (45.0 - 45.8)	12.9 (12.6 - 13.2)	2.9 (2.7 - 3.1)	0.9 (0.8 - 1.1)
Neurological disorders	Dementia as a sign with others	Female	16-24	6	NR	83.3 (27.3 - 97.5)	83.3 (27.3 - 97.5)	83.3 (27.3 - 97.5)	55.6 (7.3 - 87.6)	55.6 (7.3 - 87.6)	55.6 (7.3 - 87.6)
Neurological disorders	Dementia as a sign with others	Female	25-34	11	NR	100.0 (100.0 - 100.0)	100.0 (100.0 - 100.0)	90.9 (50.8 - 98.7)	71.6 (35.0 - 89.9)	71.6 (35.0 - 89.9)	71.6 (35.0 - 89.9)
Neurological disorders	Dementia as a sign with others	Female	35-44	47	7.21	100.0 (100.0 - 100.0)	93.4 (80.8 - 97.8)	72.9 (57.2 - 83.6)	40.0 (23.9 - 55.6)	40.0 (23.9 - 55.6)	32.0 (14.6 - 51.0)
Neurological disorders	Dementia as a sign with others	Female	45-54	503	10.07 (11.05 - 9.12)	97.0 (95.0 - 98.2)	90.0 (86.9 - 92.4)	82.6 (78.7 - 85.8)	50.2 (44.7 - 55.4)	27.3 (21.7 - 33.1)	17.9 (12.0 - 24.7)
Neurological disorders	Dementia as a sign with others	Female	55-64	3199	8.71 (8.92 - 8.46)	97.2 (96.5 - 97.7)	90.3 (89.2 - 91.3)	78.1 (76.5 - 79.6)	40.5 (38.4 - 42.6)	15.5 (13.7 - 17.4)	6.5 (5.0 - 8.3)
Neurological disorders	Dementia as a sign with others	Female	65-74	13523	6.72 (6.83 - 6.61)	95.1 (94.7 - 95.5)	83.3 (82.7 - 84.0)	66.0 (65.1 - 66.8)	25.5 (24.6 - 26.4)	6.5 (5.8 - 7.2)	1.4 (1.0 - 1.9)
Neurological disorders	Dementia as a sign with others	Female	75-84	32790	4.7 (4.76 - 4.65)	89.0 (88.7 - 89.3)	69.0 (68.5 - 69.5)	46.9 (46.4 - 47.5)	11.2 (10.7 - 11.6)	1.5 (1.3 - 1.7)	0.3 (0.2 - 0.4)
Neurological disorders	Dementia as a sign with others	Female	85-95	19921	2.69 (2.75 - 2.64)	75.1 (74.5 - 75.7)	45.7 (45.0 - 46.5)	23.5 (22.8 - 24.1)	2.1 (1.9 - 2.4)	0.1 (0.1 - 0.2)	0.1 (0.0 - 0.1)
Neurological disorders	Idiopathic epilepsy	Female	All	69166	15.84 (16.17 - 15.5)	86.7 (86.4 - 86.9)	77.2 (76.8 - 77.5)	70.3 (70.0 - 70.7)	58.8 (58.4 - 59.2)	51.1 (50.7 - 51.5)	45.6 (45.1 - 46.0)
Neurological disorders	Idiopathic epilepsy	Female	16-24	7358	NR	99.5 (99.3 - 99.6)	98.7 (98.4 - 98.9)	98.2 (97.8 - 98.5)	96.8 (96.4 - 97.2)	95.6 (95.0 - 96.1)	94.4 (93.7 - 95.1)
Neurological disorders	Idiopathic epilepsy	Female	25-34	7190	NR	98.7 (98.4 - 99.0)	97.7 (97.3 - 98.0)	96.6 (96.1 - 97.0)	94.3 (93.7 - 94.9)	92.5 (91.7 - 93.1)	90.2 (89.3 - 91.1)
Neurological disorders	Idiopathic epilepsy	Female	35-44	7005	NR	97.4 (97.0 - 97.7)	94.8 (94.3 - 95.3)	93.3 (92.7 - 93.9)	89.2 (88.4 - 89.9)	85.7 (84.8 - 86.6)	81.9 (80.7 - 83.0)
Neurological disorders	Idiopathic epilepsy	Female	45-54	7952	NR	93.4 (92.8 - 93.9)	88.6 (87.9 - 89.3)	85.1 (84.3 - 85.9)	78.4 (77.4 - 79.3)	71.5 (70.3 - 72.6)	63.7 (62.3 - 65.1)
Neurological disorders	Idiopathic epilepsy	Female	55-64	9457	16.47 (17.01 - 15.81)	88.3 (87.7 - 89.0)	80.4 (79.6 - 81.2)	75.2 (74.2 - 76.0)	63.9 (62.8 - 64.9)	52.9 (51.7 - 54.1)	42.0 (40.6 - 43.4)
Neurological disorders	Idiopathic epilepsy	Female	65-74	10998	8.92 (9.19 - 8.64)	83.8 (83.1 - 84.4)	72.8 (71.9 - 73.6)	64.6 (63.6 - 65.5)	46.1 (45.1 - 47.2)	28.6 (27.5 - 29.8)	14.6 (13.5 - 15.8)
Neurological disorders	Idiopathic epilepsy	Female	75-84	12172	4.17 (4.3 - 4.03)	76.8 (76.0 - 77.5)	58.9 (58.0 - 59.8)	44.3 (43.4 - 45.2)	19.1 (18.3 - 20.0)	6.0 (5.4 - 6.6)	1.4 (1.0 - 1.8)
Neurological disorders	Idiopathic epilepsy	Female	85-95	6783	1.84 (1.93 - 1.76)	62.5 (61.3 - 63.7)	36.8 (35.6 - 37.9)	19.7 (18.7 - 20.8)	3.0 (2.5 - 3.5)	0.4 (0.2 - 0.7)	0.0 (0.0 - 0.0)
Neurological disorders	Motor neuron disease	Female	All	4258	1.46 (1.53 - 1.4)	61.9 (60.4 - 63.4)	29.9 (28.4 - 31.3)	21.5 (20.2 - 22.8)	14.7 (13.5 - 15.9)	11.8 (10.7 - 13.0)	9.6 (8.5 - 10.8)
Neurological disorders	Motor neuron disease	Female	16-24	51	NR	94.1 (82.7 - 98.1)	94.1 (82.7 - 98.1)	89.8 (77.2 - 95.6)	84.4 (69.7 - 92.3)	84.4 (69.7 - 92.3)	84.4 (69.7 - 92.3)
Neurological disorders	Motor neuron disease	Female	25-34	69	NR	98.6 (90.2 - 99.8)	90.6 (80.3 - 95.7)	87.2 (76.0 - 93.4)	87.2 (76.0 - 93.4)	84.5 (71.8 - 91.8)	81.6 (67.6 - 89.9)
Neurological disorders	Motor neuron disease	Female	35-44	152	NR	94.7 (89.6 - 97.3)	84.2 (77.2 - 89.2)	73.1 (64.9 - 79.6)	63.4 (54.6 - 70.9)	57.5 (48.0 - 65.8)	55.3 (45.3 - 64.3)
Neurological disorders	Motor neuron disease	Female	45-54	357	3.64 (4.52 - 3.02)	85.1 (80.9 - 88.5)	55.5 (50.0 - 60.7)	43.2 (37.7 - 48.6)	32.8 (27.5 - 38.2)	29.1 (23.8 - 34.6)	22.4 (17.0 - 28.3)
Neurological disorders	Motor neuron disease	Female	55-64	827	2.06 (2.24 - 1.87)	76.4 (73.4 - 79.2)	36.1 (32.7 - 39.6)	25.3 (22.2 - 28.5)	17.2 (14.5 - 20.1)	13.7 (11.1 - 16.6)	9.5 (7.0 - 12.3)
Neurological disorders	Motor neuron disease	Female	65-74	1355	1.36 (1.47 - 1.25)	62.1 (59.4 - 64.6)	22.5 (20.2 - 24.8)	14.4 (12.5 - 16.5)	7.6 (6.1 - 9.4)	5.2 (3.8 - 6.9)	2.2 (1.0 - 4.3)
Neurological disorders	Motor neuron disease	Female	75-84	1162	0.81 (0.87 - 0.72)	42.9 (40.0 - 45.7)	15.9 (13.7 - 18.1)	9.7 (8.0 - 11.7)	4.2 (3.0 - 5.7)	0.7 (0.2 - 1.8)	0.0 (0.0 - 0.0)
Neurological disorders	Motor neuron disease	Female	85-95	283	0.56 (0.66 - 0.42)	35.0 (29.4 - 40.7)	15.0 (11.0 - 19.6)	7.3 (4.5 - 11.0)	1.4 (0.4 - 3.7)	0.0 (0.0 - 0.0)	0.0 (0.0 - 0.0)
Neurological disorders	Multiple sclerosis	Female	All	19034	NR	98.8 (98.6 - 98.9)	97.1 (96.8 - 97.3)	95.5 (95.2 - 95.8)	90.6 (90.1 - 91.0)	83.8 (83.1 - 84.4)	75.8 (74.9 - 76.6)
Neurological disorders	Multiple sclerosis	Female	16-24	1484	NR	99.9 (99.5 - 100.0)	99.7 (99.3 - 99.9)	99.7 (99.3 - 99.9)	99.5 (98.9 - 99.8)	98.8 (97.8 - 99.4)	97.3 (95.1 - 98.5)
Neurological disorders	Multiple sclerosis	Female	25-34	4099	NR	100.0 (100.0 - 100.0)	99.9 (99.8 - 100.0)	99.7 (99.4 - 99.8)	99.2 (98.8 - 99.4)	98.3 (97.7 - 98.8)	96.9 (96.0 - 97.7)
Neurological disorders	Multiple sclerosis	Female	35-44	4550	NR	99.8 (99.7 - 99.9)	99.6 (99.3 - 99.7)	99.4 (99.1 - 99.6)	98.1 (97.6 - 98.5)	95.9 (95.1 - 96.5)	92.2 (91.0 - 93.3)
Neurological disorders	Multiple sclerosis	Female	45-54	4165	NR	99.6 (99.4 - 99.8)	98.8 (98.4 - 99.1)	97.5 (97.0 - 98.0)	94.3 (93.5 - 95.0)	88.2 (87.0 - 89.3)	79.6 (77.9 - 81.2)
Neurological disorders	Multiple sclerosis	Female	55-64	2763	22.06 (22.83 - 21.57)	98.4 (97.9 - 98.8)	95.9 (95.1 - 96.6)	93.9 (92.9 - 94.8)	85.8 (84.3 - 87.1)	72.3 (70.3 - 74.1)	57.4 (54.9 - 59.8)
Neurological disorders	Multiple sclerosis	Female	65-74	1333	13.36 (14.06 - 12.82)	94.9 (93.6 - 96.0)	89.1 (87.3 - 90.7)	83.9 (81.8 - 85.8)	65.0 (62.1 - 67.7)	43.3 (40.1 - 46.4)	21.7 (18.4 - 25.2)
Neurological disorders	Multiple sclerosis	Female	75-84	524	6.12 (6.91 - 5.41)	86.7 (83.4 - 89.3)	72.3 (68.2 - 76.0)	58.1 (53.6 - 62.3)	27.1 (23.0 - 31.4)	9.8 (6.9 - 13.3)	1.4 (0.3 - 4.2)
Neurological disorders	Multiple sclerosis	Female	85-95	113	1.98 (3.01 - 1.44)	75.0 (65.9 - 82.0)	41.6 (32.3 - 50.6)	26.5 (18.5 - 35.3)	2.6 (0.5 - 8.1)	0.0 (0.0 - 0.0)	0.0 (0.0 - 0.0)
Neurological disorders	Parkinson's disease	Female	All	26575	7.31 (7.43 - 7.21)	91.3 (91.0 - 91.7)	78.6 (78.1 - 79.1)	64.5 (63.9 - 65.1)	35.7 (35.1 - 36.4)	17.9 (17.2 - 18.5)	8.9 (8.3 - 9.5)
Neurological disorders	Parkinson's disease	Female	16-24	43	NR	100.0 (100.0 - 100.0)	97.1 (81.4 - 99.6)	97.1 (81.4 - 99.6)	97.1 (81.4 - 99.6)	97.1 (81.4 - 99.6)	97.1 (81.4 - 99.6)
Neurological disorders	Parkinson's disease	Female	25-34	67	NR	100.0 (100.0 - 100.0)	100.0 (100.0 - 100.0)	98.2 (87.8 - 99.7)	98.2 (87.8 - 99.7)	95.4 (82.2 - 98.9)	95.4 (82.2 - 98.9)
Neurological disorders	Parkinson's disease	Female	35-44	219	NR	99.1 (96.3 - 99.8)	98.1 (95.1 - 99.3)	96.6 (92.9 - 98.4)	92.9 (88.0 - 95.8)	87.3 (80.7 - 91.7)	68.4 (55.6 - 78.2)
Neurological disorders	Parkinson's disease	Female	45-54	926	22.59	99.0 (98.1 - 99.5)	97.5 (96.2 - 98.3)	95.3 (93.6 - 96.6)	87.6 (84.8 - 89.8)	78.3 (74.5 - 81.7)	63.2 (57.5 - 68.3)
Neurological disorders	Parkinson's disease	Female	55-64	3104	15.89 (16.4 - 15.46)	98.3 (97.8 - 98.7)	95.0 (94.1 - 95.7)	90.7 (89.6 - 91.7)	75.5 (73.7 - 77.2)	54.9 (52.4 - 57.2)	29.9 (27.1 - 32.8)
Neurological disorders	Parkinson's disease	Female	65-74	8267	10.03 (10.22 - 9.81)	96.5 (96.1 - 96.9)	89.2 (88.5 - 89.9)	79.2 (78.2 - 80.1)	50.1 (48.8 - 51.4)	20.2 (18.9 - 21.5)	5.5 (4.6 - 6.6)
Neurological disorders	Parkinson's disease	Female	75-84	10584	5.42 (5.53 - 5.3)	89.6 (89.0 - 90.2)	73.3 (72.4 - 74.2)	54.0 (53.0 - 55.1)	16.5 (15.7 - 17.4)	2.3 (1.9 - 2.8)	0.4 (0.2 - 0.7)

Appendix Table A7: Unadjusted Median Survival and Survival Probabilities by Age, females (Page 17 of 18)

Group	Diagnosis Category	Sex	Age	N	Median Survival (y)	1y (%)	3y (%)	5y (%)	10y (%)	15y (%)	20y (%)
Neurological disorders	Parkinson's disease	Female	85-95	3300	2.77 (2.87 - 2.65)	75.3 (73.8 - 76.7)	46.7 (45.0 - 48.5)	24.9 (23.3 - 26.5)	2.5 (1.9 - 3.2)	0.2 (0.1 - 0.6)	0.2 (0.1 - 0.6)
Neurological disorders	Unspecified dementia	Female	All	135616	2.04 (2.06 - 2.02)	65.0 (64.8 - 65.3)	38.5 (38.2 - 38.8)	21.2 (21.0 - 21.4)	4.4 (4.2 - 4.5)	1.2 (1.2 - 1.3)	0.6 (0.5 - 0.7)
Neurological disorders	Unspecified dementia	Female	16-24	20	NR	100.0 (100.0 - 100.0)	100.0 (100.0 - 100.0)	94.1 (65.0 - 99.1)	94.1 (65.0 - 99.1)	94.1 (65.0 - 99.1)	94.1 (65.0 - 99.1)
Neurological disorders	Unspecified dementia	Female	25-34	35	NR	100.0 (100.0 - 100.0)	100.0 (100.0 - 100.0)	100.0 (100.0 - 100.0)	96.3 (76.5 - 99.5)	90.3 (65.1 - 97.6)	90.3 (65.1 - 97.6)
Neurological disorders	Unspecified dementia	Female	35-44	95	NR	96.8 (90.5 - 99.0)	92.3 (84.5 - 96.3)	89.9 (81.5 - 94.6)	75.6 (64.1 - 83.8)	64.1 (50.1 - 75.1)	64.1 (50.1 - 75.1)
Neurological disorders	Unspecified dementia	Female	45-54	500	12.81 (16.31 - 10.88)	92.9 (90.3 - 94.9)	84.4 (80.8 - 87.3)	76.8 (72.7 - 80.4)	56.6 (51.6 - 61.3)	46.4 (40.9 - 51.6)	41.5 (35.2 - 47.7)
Neurological disorders	Unspecified dementia	Female	55-64	2102	7.76 (8.12 - 7.22)	88.4 (86.9 - 89.7)	77.2 (75.3 - 78.9)	64.8 (62.6 - 66.9)	40.0 (37.6 - 42.4)	22.4 (20.1 - 24.9)	16.0 (13.6 - 18.6)
Neurological disorders	Unspecified dementia	Female	65-74	10896	4.55 (4.67 - 4.43)	82.6 (81.9 - 83.3)	64.5 (63.6 - 65.4)	46.2 (45.2 - 47.1)	15.8 (15.0 - 16.7)	5.0 (4.4 - 5.6)	1.3 (0.9 - 1.8)
Neurological disorders	Unspecified dementia	Female	75-84	49867	2.77 (2.8 - 2.73)	72.2 (71.8 - 72.6)	47.3 (46.9 - 47.8)	27.6 (27.2 - 28.1)	4.9 (4.7 - 5.1)	0.6 (0.5 - 0.7)	0.1 (0.0 - 0.1)
Neurological disorders	Unspecified dementia	Female	85-95	67519	1.43 (1.46 - 1.41)	57.6 (57.2 - 58.0)	27.8 (27.5 - 28.2)	11.6 (11.3 - 11.8)	0.8 (0.7 - 0.9)	0.0 (0.0 - 0.0)	0.0 (0.0 - 0.0)
Neurological disorders	Vascular dementia	Female	All	40685	2.67 (2.72 - 2.63)	72.6 (72.2 - 73.1)	46.2 (45.7 - 46.7)	27.3 (26.9 - 27.8)	6.7 (6.4 - 7.0)	2.0 (1.8 - 2.2)	0.7 (0.6 - 0.9)
Neurological disorders	Vascular dementia	Female	16-24	13	NR	100.0 (100.0 - 100.0)	100.0 (100.0 - 100.0)	100.0 (100.0 - 100.0)	100.0 (100.0 - 100.0)	88.9 (43.3 - 98.4)	88.9 (43.3 - 98.4)
Neurological disorders	Vascular dementia	Female	25-34	17	NR	100.0 (100.0 - 100.0)	100.0 (100.0 - 100.0)	100.0 (100.0 - 100.0)	100.0 (100.0 - 100.0)	83.3 (27.3 - 97.5)	83.3 (27.3 - 97.5)
Neurological disorders	Vascular dementia	Female	35-44	36	NR	97.1 (81.4 - 99.6)	94.3 (79.0 - 98.5)	91.1 (75.0 - 97.1)	64.8 (44.0 - 79.5)	51.0 (30.2 - 68.5)	51.0 (30.2 - 68.5)
Neurological disorders	Vascular dementia	Female	45-54	154	16.56	94.7 (89.8 - 97.3)	87.3 (80.7 - 91.7)	85.0 (78.2 - 89.9)	65.3 (56.0 - 73.0)	54.4 (44.5 - 63.3)	46.3 (34.6 - 57.2)
Neurological disorders	Vascular dementia	Female	55-64	762	8.79 (9.48 - 7.97)	91.8 (89.5 - 93.5)	80.2 (77.1 - 82.9)	70.2 (66.6 - 73.4)	42.6 (38.6 - 46.6)	23.2 (19.4 - 27.3)	10.3 (6.8 - 14.7)
Neurological disorders	Vascular dementia	Female	65-74	4350	5.06 (5.24 - 4.89)	86.5 (85.5 - 87.5)	68.2 (66.8 - 69.6)	50.5 (48.9 - 52.1)	19.9 (18.5 - 21.3)	6.4 (5.5 - 7.5)	1.5 (0.8 - 2.4)
Neurological disorders	Vascular dementia	Female	75-84	17683	3.23 (3.29 - 3.16)	77.8 (77.2 - 78.4)	52.7 (51.9 - 53.4)	32.1 (31.3 - 32.8)	6.3 (5.9 - 6.8)	0.9 (0.7 - 1.1)	0.1 (0.0 - 0.2)
Neurological disorders	Vascular dementia	Female	85-95	17071	1.77 (1.82 - 1.73)	63.5 (62.8 - 64.2)	32.5 (31.8 - 33.3)	14.1 (13.6 - 14.7)	1.1 (0.9 - 1.3)	0.1 (0.0 - 0.2)	0.0 (0.0 - 0.1)
Other	Decubitus ulcer	Female	All	40244	2.03 (2.08 - 1.97)	60.8 (60.3 - 61.3)	41.8 (41.3 - 42.3)	30.0 (29.5 - 30.5)	15.6 (15.1 - 16.0)	9.7 (9.2 - 10.1)	6.7 (6.2 - 7.1)
Other	Decubitus ulcer	Female	16-24	339	NR	96.7 (94.1 - 98.2)	94.8 (91.7 - 96.7)	94.1 (90.9 - 96.2)	86.7 (82.0 - 90.2)	83.9 (78.4 - 88.1)	80.6 (73.8 - 85.7)
Other	Decubitus ulcer	Female	25-34	438	NR	93.5 (90.7 - 95.4)	89.1 (85.6 - 91.7)	87.6 (83.9 - 90.5)	81.7 (77.1 - 85.5)	78.2 (72.9 - 82.6)	73.5 (66.5 - 79.3)
Other	Decubitus ulcer	Female	35-44	654	NR	92.5 (90.2 - 94.3)	87.3 (84.4 - 89.7)	82.9 (79.6 - 85.7)	72.5 (68.4 - 76.2)	63.6 (58.7 - 68.1)	55.5 (49.1 - 61.4)
Other	Decubitus ulcer	Female	45-54	1302	11.53 (13.04 - 10.76)	83.8 (81.6 - 85.7)	75.8 (73.3 - 78.1)	68.1 (65.3 - 70.7)	54.9 (51.8 - 58.0)	41.1 (37.4 - 44.8)	31.1 (26.3 - 36.1)
Other	Decubitus ulcer	Female	55-64	3003	6.03 (6.63 - 5.42)	75.3 (73.7 - 76.8)	62.5 (60.7 - 64.3)	53.2 (51.2 - 55.0)	37.7 (35.7 - 39.8)	26.7 (24.6 - 28.9)	18.8 (16.2 - 21.7)
Other	Decubitus ulcer	Female	65-74	7067	3.6 (3.89 - 3.36)	68.8 (67.7 - 69.9)	53.3 (52.0 - 54.5)	43.1 (41.8 - 44.3)	24.7 (23.5 - 26.0)	13.8 (12.6 - 15.1)	7.1 (5.8 - 8.5)
Other	Decubitus ulcer	Female	75-84	12921	2.01 (2.08 - 1.91)	60.9 (60.1 - 61.8)	40.9 (40.0 - 41.8)	27.4 (26.5 - 28.2)	9.0 (8.4 - 9.6)	2.4 (2.0 - 2.8)	0.2 (0.1 - 0.6)
Other	Decubitus ulcer	Female	85-95	13224	0.97 (1.02 - 0.92)	49.5 (48.7 - 50.4)	25.8 (25.1 - 26.6)	12.2 (11.6 - 12.9)	1.4 (1.2 - 1.7)	0.0 (0.0 - 0.2)	0.0 (0.0 - 0.0)
Other	Endo/metab/blood/immune	Female	All	1016357	NR	94.5 (94.5 - 94.6)	89.8 (89.7 - 89.8)	85.4 (85.3 - 85.5)	75.1 (75.0 - 75.2)	65.0 (64.9 - 65.1)	55.9 (55.7 - 56.0)
Other	Endo/metab/blood/immune	Female	16-24	53260	NR	99.8 (99.8 - 99.8)	99.6 (99.5 - 99.6)	99.4 (99.3 - 99.5)	98.9 (98.8 - 99.0)	98.5 (98.4 - 98.6)	97.9 (97.6 - 98.1)
Other	Endo/metab/blood/immune	Female	25-34	155423	NR	99.8 (99.8 - 99.8)	99.6 (99.6 - 99.7)	99.5 (99.5 - 99.5)	99.1 (99.0 - 99.1)	98.4 (98.3 - 98.5)	97.3 (97.1 - 97.4)
Other	Endo/metab/blood/immune	Female	35-44	127825	NR	99.5 (99.5 - 99.5)	99.0 (99.0 - 99.1)	98.6 (98.6 - 98.7)	97.6 (97.5 - 97.7)	96.0 (95.8 - 96.1)	93.4 (93.2 - 93.7)
Other	Endo/metab/blood/immune	Female	45-54	122146	NR	98.5 (98.4 - 98.6)	97.2 (97.1 - 97.3)	96.2 (96.1 - 96.3)	93.3 (93.2 - 93.5)	89.4 (89.2 - 89.7)	83.5 (83.2 - 83.9)
Other	Endo/metab/blood/immune	Female	55-64	152697	NR	96.7 (96.6 - 96.8)	94.0 (93.9 - 94.1)	91.7 (91.5 - 91.8)	85.4 (85.2 - 85.6)	76.5 (76.3 - 76.8)	64.0 (63.6 - 64.5)
Other	Endo/metab/blood/immune	Female	65-74	179515	15.14 (15.22 - 15.05)	93.8 (93.7 - 93.9)	88.6 (88.4 - 88.7)	83.7 (83.5 - 83.9)	69.5 (69.3 - 69.8)	50.6 (50.2 - 50.9)	29.2 (28.8 - 29.7)
Other	Endo/metab/blood/immune	Female	75-84	156814	7.85 (7.91 - 7.8)	88.4 (88.3 - 88.6)	77.5 (77.3 - 77.7)	66.5 (66.2 - 66.7)	38.0 (37.7 - 38.3)	15.1 (14.8 - 15.4)	3.6 (3.3 - 3.8)
Other	Endo/metab/blood/immune	Female	85-95	65808	3.19 (3.23 - 3.15)	74.2 (73.8 - 74.5)	51.9 (51.5 - 52.3)	33.5 (33.1 - 33.9)	7.9 (7.6 - 8.1)	0.9 (0.7 - 1.0)	0.2 (0.1 - 0.3)
Other	HIV/AIDS	Female	All	20506	NR	96.9 (96.6 - 97.1)	94.0 (93.6 - 94.3)	91.6 (91.2 - 92.0)	86.6 (86.1 - 87.1)	82.1 (81.4 - 82.7)	76.3 (75.4 - 77.2)
Other	HIV/AIDS	Female	16-24	3920	NR	99.6 (99.4 - 99.8)	99.5 (99.2 - 99.7)	99.1 (98.7 - 99.4)	98.5 (98.1 - 98.9)	98.0 (97.4 - 98.4)	97.3 (96.4 - 97.9)
Other	HIV/AIDS	Female	25-34	4221	NR	99.6 (99.3 - 99.7)	99.2 (98.9 - 99.4)	98.7 (98.3 - 99.0)	97.8 (97.3 - 98.3)	96.9 (96.2 - 97.4)	95.7 (94.7 - 96.5)
Other	HIV/AIDS	Female	35-44	3448	NR	99.2 (98.8 - 99.4)	98.5 (98.0 - 98.9)	97.8 (97.3 - 98.3)	96.2 (95.5 - 96.8)	94.5 (93.6 - 95.4)	91.8 (90.4 - 93.1)
Other	HIV/AIDS	Female	45-54	2812	NR	98.3 (97.8 - 98.8)	96.6 (95.9 - 97.2)	94.9 (94.0 - 95.7)	90.8 (89.6 - 91.9)	86.3 (84.7 - 87.8)	80.5 (78.2 - 82.5)
Other	HIV/AIDS	Female	55-64	2692	NR	96.2 (95.4 - 96.9)	92.2 (91.1 - 93.2)	88.8 (87.5 - 90.0)	80.1 (78.3 - 81.7)	71.7 (69.6 - 73.8)	59.0 (56.0 - 61.9)
Other	HIV/AIDS	Female	65-74	2021	13.91 (14.65 - 12.85)	92.0 (90.7 - 93.1)	83.3 (81.6 - 84.9)	76.8 (74.8 - 78.7)	61.5 (59.0 - 63.9)	46.0 (43.0 - 49.0)	25.9 (22.2 - 29.8)
Other	HIV/AIDS	Female	75-84	1067	6.5 (7.04 - 5.9)	83.4 (81.0 - 85.6)	70.7 (67.8 - 73.4)	59.1 (55.9 - 62.1)	31.6 (28.3 - 35.0)	9.0 (6.6 - 11.9)	1.6 (0.5 - 4.0)
Other	HIV/AIDS	Female	85-95	315	2.41 (2.79 - 2.08)	71.0 (65.6 - 75.7)	43.4 (37.7 - 49.0)	24.5 (19.6 - 29.8)	5.6 (3.0 - 9.5)	1.1 (0.1 - 4.6)	1.1 (0.1 - 4.6)
Other	Hemoglobinopathies	Female	All	356494	6.36 (6.42 - 6.31)	79.3 (79.2 - 79.5)	65.1 (65.0 - 65.3)	55.3 (55.1 - 55.5)	39.7 (39.5 - 39.9)	30.9 (30.6 - 31.1)	25.5 (25.2 - 25.7)
Other	Hemoglobinopathies	Female	16-24	11164	NR	99.1 (98.9 - 99.2)	98.3 (98.0 - 98.5)	97.7 (97.4 - 98.0)	96.9 (96.6 - 97.3)	96.3 (95.9 - 96.8)	95.6 (95.0 - 96.2)
Other	Hemoglobinopathies	Female	25-34	25166	NR	98.9 (98.7 - 99.0)	98.0 (97.8 - 98.2)	97.6 (97.4 - 97.8)	96.6 (96.3 - 96.9)	95.7 (95.4 - 96.1)	94.4 (93.8 - 94.9)
Other	Hemoglobinopathies	Female	35-44	26549	NR	96.8 (96.5 - 97.0)	94.8 (94.5 - 95.1)	93.7 (93.4 - 94.0)	91.6 (91.2 - 92.0)	89.2 (88.6 - 89.6)	86.1 (85.3 - 86.8)

Appendix Table A7: Unadjusted Median Survival and Survival Probabilities by Age, females (Page 18 of 18)

Group	Diagnosis Category	Sex	Age	N	Median Survival (y)	1y (%)	3y (%)	5y (%)	10y (%)	15y (%)	20y (%)
Other	Hemoglobinopathies	Female	45-54	27711	NR	91.2 (90.8 - 91.5)	86.6 (86.1 - 87.0)	83.9 (83.5 - 84.4)	79.1 (78.5 - 79.6)	74.3 (73.6 - 74.9)	69.1 (68.2 - 70.0)
Other	Hemoglobinopathies	Female	55-64	33517	12.74 (13.07 - 12.38)	81.7 (81.2 - 82.1)	71.9 (71.4 - 72.3)	66.1 (65.6 - 66.7)	55.4 (54.8 - 56.0)	45.5 (44.8 - 46.2)	35.6 (34.6 - 36.6)
Other	Hemoglobinopathies	Female	65-74	61118	7.29 (7.41 - 7.16)	79.1 (78.7 - 79.4)	66.8 (66.4 - 67.1)	58.3 (57.9 - 58.7)	41.0 (40.5 - 41.5)	25.9 (25.4 - 26.5)	13.0 (12.4 - 13.7)
Other	Hemoglobinopathies	Female	75-84	92826	4.15 (4.21 - 4.1)	75.9 (75.6 - 76.1)	58.3 (57.9 - 58.6)	44.5 (44.2 - 44.9)	19.8 (19.5 - 20.1)	6.4 (6.1 - 6.6)	1.2 (1.1 - 1.4)
Other	Hemoglobinopathies	Female	85-95	73261	2.0 (2.03 - 1.97)	64.7 (64.3 - 65.0)	38.3 (37.9 - 38.7)	21.3 (21.0 - 21.6)	3.6 (3.4 - 3.8)	0.3 (0.3 - 0.4)	0.0 (0.0 - 0.1)
Other	Psoriasis	Female	All	122429	NR	98.6 (98.5 - 98.6)	96.1 (96.0 - 96.2)	93.5 (93.4 - 93.7)	86.2 (86.0 - 86.5)	78.0 (77.7 - 78.3)	69.2 (68.8 - 69.6)
Other	Psoriasis	Female	16-24	8970	NR	100.0 (99.9 - 100.0)	99.9 (99.8 - 99.9)	99.8 (99.7 - 99.9)	99.5 (99.3 - 99.7)	99.2 (98.9 - 99.4)	98.9 (98.5 - 99.2)
Other	Psoriasis	Female	25-34	13376	NR	99.9 (99.9 - 100.0)	99.9 (99.8 - 99.9)	99.8 (99.7 - 99.8)	99.4 (99.3 - 99.6)	98.9 (98.6 - 99.1)	98.3 (98.0 - 98.6)
Other	Psoriasis	Female	35-44	16157	NR	99.9 (99.8 - 99.9)	99.7 (99.6 - 99.8)	99.5 (99.3 - 99.6)	98.6 (98.4 - 98.8)	97.4 (97.1 - 97.7)	95.1 (94.6 - 95.6)
Other	Psoriasis	Female	45-54	22632	NR	99.7 (99.6 - 99.7)	99.0 (98.8 - 99.1)	98.3 (98.1 - 98.4)	95.8 (95.5 - 96.1)	91.8 (91.3 - 92.2)	85.2 (84.5 - 86.0)
Other	Psoriasis	Female	55-64	26974	NR	99.4 (99.3 - 99.4)	97.9 (97.7 - 98.1)	96.2 (96.0 - 96.5)	90.6 (90.2 - 90.9)	82.4 (81.8 - 83.0)	69.9 (69.0 - 70.8)
Other	Psoriasis	Female	65-74	20600	16.62 (16.86 - 16.42)	98.1 (97.9 - 98.2)	94.4 (94.0 - 94.7)	90.5 (90.1 - 91.0)	77.2 (76.5 - 77.8)	57.4 (56.4 - 58.3)	33.9 (32.6 - 35.2)
Other	Psoriasis	Female	75-84	10695	9.3 (9.46 - 9.1)	94.7 (94.2 - 95.1)	85.6 (84.9 - 86.2)	74.9 (74.0 - 75.7)	45.6 (44.5 - 46.7)	19.3 (18.2 - 20.3)	5.4 (4.5 - 6.3)
Other	Psoriasis	Female	85-95	2924	4.2 (4.39 - 4.0)	84.1 (82.7 - 85.4)	61.5 (59.7 - 63.3)	42.2 (40.4 - 44.1)	11.4 (10.1 - 12.8)	1.2 (0.7 - 1.9)	0.1 (0.0 - 0.6)
Other	Tuberculosis	Female	All	22362	NR	95.4 (95.1 - 95.7)	91.8 (91.4 - 92.1)	88.5 (88.1 - 88.9)	81.6 (81.1 - 82.2)	75.2 (74.5 - 75.9)	68.1 (67.1 - 69.0)
Other	Tuberculosis	Female	16-24	2737	NR	99.9 (99.7 - 100.0)	99.8 (99.5 - 99.9)	99.6 (99.2 - 99.8)	99.3 (98.9 - 99.6)	99.0 (98.4 - 99.4)	98.1 (96.8 - 98.8)
Other	Tuberculosis	Female	25-34	4720	NR	99.8 (99.6 - 99.9)	99.6 (99.4 - 99.8)	99.5 (99.2 - 99.6)	99.2 (98.9 - 99.4)	98.7 (98.3 - 99.1)	97.5 (96.4 - 98.4)
Other	Tuberculosis	Female	35-44	3771	NR	99.7 (99.4 - 99.8)	99.2 (98.9 - 99.4)	98.7 (98.3 - 99.0)	98.0 (97.5 - 98.4)	97.0 (96.3 - 97.6)	95.7 (94.5 - 96.6)
Other	Tuberculosis	Female	45-54	2847	NR	98.7 (98.2 - 99.0)	97.7 (97.0 - 98.2)	96.8 (96.0 - 97.4)	94.3 (93.3 - 95.1)	91.2 (89.9 - 92.4)	85.8 (83.3 - 87.9)
Other	Tuberculosis	Female	55-64	2551	NR	97.9 (97.3 - 98.4)	95.4 (94.5 - 96.2)	92.7 (91.6 - 93.7)	87.0 (85.5 - 88.3)	77.3 (75.2 - 79.3)	63.9 (60.6 - 67.0)
Other	Tuberculosis	Female	65-74	2463	13.27 (13.8 - 12.63)	92.8 (91.7 - 93.7)	87.0 (85.6 - 88.3)	80.2 (78.6 - 81.8)	63.3 (61.1 - 65.3)	44.0 (41.5 - 46.5)	24.4 (21.5 - 27.4)
Other	Tuberculosis	Female	75-84	2297	5.99 (6.35 - 5.66)	82.0 (80.3 - 83.5)	67.4 (65.4 - 69.3)	56.1 (54.0 - 58.1)	29.8 (27.8 - 31.8)	11.1 (9.6 - 12.8)	2.1 (1.2 - 3.4)
Other	Tuberculosis	Female	85-95	939	2.54 (2.9 - 2.2)	68.5 (65.5 - 71.4)	46.0 (42.8 - 49.2)	28.9 (26.0 - 31.9)	5.5 (4.0 - 7.3)	1.0 (0.4 - 2.1)	0.0 (0.0 - 0.0)
Other	Urinary tract infections and interstitial nephritis	Female	All	742780	14.78 (14.89 - 14.66)	88.3 (88.3 - 88.4)	78.0 (77.9 - 78.1)	70.0 (69.9 - 70.1)	57.2 (57.1 - 57.3)	49.7 (49.6 - 49.9)	44.6 (44.4 - 44.7)
Other	Urinary tract infections and interstitial nephritis	Female	16-24	84908	NR	99.9 (99.9 - 99.9)	99.8 (99.8 - 99.8)	99.7 (99.7 - 99.7)	99.4 (99.3 - 99.5)	99.1 (99.0 - 99.2)	98.7 (98.6 - 98.9)
Other	Urinary tract infections and interstitial nephritis	Female	25-34	75185	NR	99.8 (99.8 - 99.8)	99.6 (99.5 - 99.6)	99.4 (99.3 - 99.4)	98.9 (98.8 - 99.0)	98.3 (98.2 - 98.4)	97.5 (97.3 - 97.7)
Other	Urinary tract infections and interstitial nephritis	Female	35-44	60059	NR	99.3 (99.2 - 99.3)	98.6 (98.5 - 98.7)	98.2 (98.0 - 98.3)	96.9 (96.8 - 97.1)	95.3 (95.1 - 95.5)	93.4 (93.1 - 93.7)
Other	Urinary tract infections and interstitial nephritis	Female	45-54	60370	NR	97.8 (97.7 - 98.0)	96.2 (96.0 - 96.3)	94.9 (94.7 - 95.1)	91.8 (91.6 - 92.1)	87.5 (87.2 - 87.9)	82.1 (81.6 - 82.6)
Other	Urinary tract infections and interstitial nephritis	Female	55-64	70312	22.83	94.3 (94.1 - 94.5)	90.0 (89.8 - 90.2)	86.8 (86.6 - 87.1)	79.2 (78.8 - 79.5)	70.3 (69.8 - 70.7)	58.7 (58.0 - 59.3)
Other	Urinary tract infections and interstitial nephritis	Female	65-74	99447	11.88 (12.0 - 11.77)	89.2 (89.0 - 89.4)	80.6 (80.4 - 80.9)	73.4 (73.1 - 73.7)	56.5 (56.2 - 56.9)	39.2 (38.7 - 39.6)	21.8 (21.2 - 22.3)
Other	Urinary tract infections and interstitial nephritis	Female	75-84	154401	5.09 (5.13 - 5.05)	82.3 (82.1 - 82.5)	65.4 (65.2 - 65.7)	50.6 (50.3 - 50.8)	22.7 (22.5 - 23.0)	7.7 (7.5 - 7.9)	1.7 (1.6 - 1.9)
Other	Urinary tract infections and interstitial nephritis	Female	85-95	129630	2.44 (2.46 - 2.42)	70.1 (69.9 - 70.4)	43.2 (42.9 - 43.5)	24.1 (23.8 - 24.3)	3.9 (3.8 - 4.1)	0.4 (0.4 - 0.5)	0.1 (0.0 - 0.1)

Appendix Table A8: Person-Years of Survival Lost: Low (Q1) and Middle (Q2 + Q3) Income vs High Income

NS = not statistically significant (p≥0.05); totals include all point estimates; subcauses excluded from table

Group	Diagnosis	N (Q1)	N (Q2-Q3)	N (Q4)	RMST Q1 (95% CI)	RMST Q2-Q3 (95% CI)	RMST Q4 (95% CI)	PY Lost Q1 (95% CI)	PY Lost Q2-Q3 (95% CI)	PY Lost Total (95% CI)
Cardiovascular diseases	Hypertension	540,820	1,019,628	462,684	7.52 (7.51-7.53)	7.88 (7.88-7.89)	8.17 (8.16-8.18)	352,494 (346,542-358,447)	293,260 (283,636-302,885)	645,755 (634,438-657,071)
Other	Endo/metab/blood/immune	466,093	833,623	394,185	8.12 (8.12-8.13)	8.43 (8.43-8.44)	8.65 (8.64-8.65)	244,265 (239,070-249,460)	179,070 (171,385-186,755)	423,335 (414,059-432,611)
Cardiovascular diseases	Atrial fibrillation	210,947	407,366	196,435	5.82 (5.81-5.84)	6.30 (6.29-6.32)	6.76 (6.75-6.77)	197,145 (192,879-201,410)	184,719 (177,782-191,656)	381,863 (373,720-390,007)
Other	Urinary tract infections and interstitial nephritis	344,206	573,655	271,577	6.61 (6.61-6.62)	6.93 (6.92-6.94)	7.21 (7.20-7.22)	206,089 (201,305-210,873)	162,084 (155,084-169,084)	368,173 (359,694-376,652)
Cardiovascular diseases	Stroke	217,136	386,438	178,352	5.94 (5.92-5.95)	6.41 (6.40-6.42)	6.83 (6.81-6.84)	193,196 (188,901-197,492)	159,251 (152,608-165,893)	352,447 (344,537-360,357)
Cardiovascular diseases	Heart failure	212,381	351,668	144,025	4.29 (4.28-4.31)	4.72 (4.71-4.73)	5.15 (5.13-5.16)	181,085 (176,097-186,074)	150,701 (143,317-158,086)	331,787 (322,876-340,698)
Cardiovascular diseases	Ischemic heart disease	231,612	409,117	171,425	6.60 (6.59-6.61)	7.02 (7.01-7.03)	7.36 (7.34-7.37)	175,692 (171,674-179,710)	139,473 (133,360-145,585)	315,165 (307,850-322,479)
Diabetes & CKD	Diabetes	242,652	366,721	140,279	7.44 (7.43-7.45)	7.76 (7.75-7.77)	8.03 (8.01-8.04)	142,038 (137,460-146,615)	97,524 (91,381-103,668)	239,562 (231,901-247,223)
Digestive diseases	Upper digest diseases	283,066	445,685	186,147	8.19 (8.18-8.20)	8.49 (8.48-8.49)	8.69 (8.69-8.70)	142,687 (138,781-146,593)	92,645 (87,442-97,849)	235,333 (228,826-241,839)
Chronic respiratory	COPD	128,490	181,032	63,937	5.73 (5.71-5.75)	6.29 (6.27-6.30)	6.79 (6.77-6.82)	136,442 (132,465-140,419)	92,417 (87,146-97,689)	228,859 (222,256-235,462)
Diabetes & CKD	Chronic kidney disease	139,906	222,947	93,645	4.38 (4.36-4.40)	4.88 (4.86-4.89)	5.28 (5.25-5.30)	125,862 (121,591-130,132)	89,416 (83,095-95,737)	215,278 (207,649-222,906)
Other	Hemoglobinopathies	197,518	305,256	135,376	5.26 (5.25-5.28)	5.56 (5.54-5.57)	5.86 (5.84-5.88)	117,976 (113,206-122,746)	92,655 (85,836-99,474)	210,631 (202,309-218,953)
Musculoskeletal disorders	Osteoarthritis	188,012	433,632	223,086	8.87 (8.86-8.88)	9.08 (9.07-9.09)	9.24 (9.24-9.25)	70,556 (67,961-73,150)	70,664 (66,134-75,195)	141,220 (136,000-146,441)
Neurological disorders	Alzheimer's disease and other dementias	101,123	184,530	88,611	3.30 (3.29-3.32)	3.55 (3.54-3.56)	3.90 (3.88-3.92)	60,442 (57,630-63,254)	64,927 (60,272-69,582)	125,369 (119,931-130,808)
Cardiovascular diseases	Nonrheum valv diseases	70,452	136,933	65,314	6.33 (6.30-6.35)	6.76 (6.74-6.78)	7.18 (7.16-7.21)	60,420 (57,946-62,894)	57,963 (53,820-62,106)	118,383 (113,558-123,209)
Digestive diseases	Diverticular disease	89,710	193,936	92,675	8.03 (8.02-8.05)	8.33 (8.32-8.35)	8.58 (8.56-8.60)	48,935 (46,571-51,298)	47,947 (43,753-52,141)	96,881 (92,067-101,695)
Cardiovascular diseases	Peripheral artery	58,200	97,711	37,279	5.51 (5.48-5.53)	6.03 (6.01-6.05)	6.46 (6.42-6.49)	55,246 (52,847-57,645)	41,389 (37,699-45,079)	96,634 (92,233-101,036)
Musculoskeletal disorders	Low back pain	165,380	273,040	128,932	8.99 (8.98-9.00)	9.15 (9.15-9.16)	9.30 (9.29-9.31)	51,754 (49,430-54,077)	39,802 (36,653-42,952)	91,556 (87,642-95,470)
Chronic respiratory	Asthma	109,407	200,262	90,876	8.54 (8.52-8.55)	8.81 (8.80-8.82)	9.01 (8.99-9.02)	51,179 (49,097-53,261)	40,033 (36,822-43,244)	91,212 (87,385-95,039)
Mental disorders & substance use	Anxiety disorders	310,667	435,731	166,042	9.21 (9.21-9.22)	9.32 (9.31-9.32)	9.39 (9.39-9.40)	56,672 (53,511-59,833)	33,754 (29,816-37,691)	90,426 (85,377-95,474)
Mental disorders & substance use	Alcohol use disorders	126,889	173,699	68,720	8.68 (8.67-8.70)	8.95 (8.94-8.96)	9.13 (9.12-9.15)	57,181 (54,491-59,872)	32,100 (28,793-35,408)	89,282 (85,018-93,545)
Digestive diseases	Gallbladder & biliary	143,523	252,463	103,573	8.36 (8.34-8.37)	8.58 (8.57-8.59)	8.71 (8.70-8.73)	51,312 (48,543-54,081)	32,773 (28,645-36,901)	84,085 (79,114-89,056)
Cancers	Prostate cancer	52,560	128,246	72,522	7.01 (6.98-7.03)	7.51 (7.49-7.52)	7.82 (7.80-7.84)	42,977 (41,159-44,795)	40,575 (37,025-44,124)	83,552 (79,564-87,540)
Mental disorders & substance use	Depressive disorders	229,431	335,088	125,904	8.83 (8.82-8.84)	8.94 (8.93-8.95)	9.04 (9.03-9.05)	48,456 (45,632-51,281)	34,296 (30,527-38,065)	82,752 (78,042-87,462)
Digestive diseases	Cirrhosis	68,480	65,239	24,706	7.44 (7.41-7.46)	7.81 (7.78-7.83)	8.18 (8.15-8.21)	50,885 (48,033-53,737)	24,480 (21,882-27,078)	75,365 (71,507-79,223)
Cancers	Colorectal cancer	40,151	80,125	38,090	5.18 (5.14-5.21)	5.68 (5.65-5.71)	6.04 (6.00-6.08)	34,505 (32,431-36,579)	28,718 (25,114-32,321)	63,222 (59,065-67,380)
Musculoskeletal disorders	Gout	36,494	67,383	30,263	6.08 (6.04-6.11)	6.47 (6.45-6.50)	6.90 (6.86-6.94)	29,950 (28,114-31,785)	28,620 (25,684-31,555)	58,569 (55,107-62,032)
Cardiovascular diseases	Aortic aneurysm	29,532	57,321	23,977	6.15 (6.11-6.20)	6.72 (6.69-6.75)	7.12 (7.08-7.17)	28,712 (26,977-30,447)	23,450 (20,579-26,322)	52,162 (48,807-55,517)
Chronic respiratory	Sleep Apnea	71,852	151,621	70,036	9.08 (9.06-9.10)	9.38 (9.37-9.39)	9.52 (9.51-9.53)	31,315 (29,628-33,002)	20,776 (18,139-23,413)	52,092 (48,961-55,222)
Digestive diseases	Inflammatory bowel	59,929	119,618	55,630	8.33 (8.31-8.35)	8.58 (8.57-8.59)	8.78 (8.76-8.80)	26,894 (25,333-28,454)	23,694 (21,116-26,272)	50,588 (47,574-53,601)
Cancers	Lung cancer	34,259	54,330	20,328	2.11 (2.08-2.14)	2.49 (2.46-2.52)	2.90 (2.85-2.95)	27,153 (25,112-29,194)	22,426 (19,290-25,562)	49,579 (45,837-53,321)
Cancers	Breast cancer	43,078	94,230	52,249	7.92 (7.90-7.95)	8.30 (8.28-8.31)	8.52 (8.50-8.54)	25,808 (24,310-27,306)	21,354 (18,822-23,885)	47,162 (44,220-50,103)
Neurological disorders	Idiopathic epilepsy	49,106	69,002	29,973	6.84 (6.82-6.87)	7.27 (7.24-7.29)	7.46 (7.43-7.50)	30,468 (28,353-32,583)	13,784 (11,045-16,523)	44,252 (40,792-47,713)
Other	Psoriasis	56,678	120,307	56,041	9.05 (9.03-9.06)	9.30 (9.29-9.31)	9.43 (9.41-9.44)	21,569 (20,375-22,764)	15,638 (13,689-17,588)	37,208 (34,921-39,494)
Digestive diseases	Pancreatitis	33,823	54,078	22,335	7.37 (7.34-7.41)	7.76 (7.73-7.78)	8.03 (7.99-8.06)	22,054 (20,245-23,863)	14,538 (12,021-17,056)	36,593 (33,493-39,693)
Cardiovascular diseases	Cardiomyopathy	23,932	41,752	19,330	7.28 (7.24-7.32)	7.79 (7.76-7.82)	8.12 (8.08-8.16)	20,226 (18,859-21,594)	13,855 (11,754-15,956)	34,081 (31,574-36,588)
Cancers	Bladder cancer	18,216	36,414	17,208	5.77 (5.72-5.83)	6.32 (6.28-6.36)	6.75 (6.70-6.80)	17,784 (16,433-19,135)	15,712 (13,435-17,988)	33,496 (30,849-36,143)
Chronic respiratory	Interstitial lung disease	17,286	33,858	14,896	6.04 (5.99-6.09)	6.47 (6.43-6.50)	6.89 (6.84-6.95)	14,848 (13,598-16,098)	14,516 (12,376-16,655)	29,364 (26,886-31,842)
Musculoskeletal disorders	Rheumatoid arthritis	31,738	63,057	27,995	8.37 (8.34-8.39)	8.63 (8.62-8.65)	8.85 (8.82-8.87)	15,156 (14,026-16,286)	13,432 (11,567-15,298)	28,589 (26,407-30,770)
Cardiovascular diseases	Hypertensive heart disease	19,705	34,736	14,832	6.28 (6.24-6.32)	6.66 (6.63-6.69)	7.04 (7.00-7.09)	15,088 (13,933-16,243)	13,328 (11,530-15,126)	28,416 (26,279-30,553)
Cancers	Melanoma	16,032	47,450	30,037	7.60 (7.55-7.65)	8.03 (8.00-8.06)	8.33 (8.29-8.36)	11,672 (10,669-12,676)	14,330 (12,222-16,438)	26,003 (23,668-28,338)
Other	Decubitus ulcer	25,793	35,675	15,364	3.36 (3.32-3.40)	3.59 (3.56-3.63)	3.91 (3.85-3.97)	14,260 (12,424-16,095)	11,381 (8,933-13,828)	25,640 (22,581-28,700)
Musculoskeletal disorders	Neck pain	70,505	123,321	55,990	9.37 (9.35-9.38)	9.52 (9.51-9.53)	9.59 (9.58-9.60)	15,847 (14,455-17,239)	8,990 (7,057-10,923)	24,837 (22,455-27,219)
Mental disorders & substance use	Drug use disorders	80,615	78,895	24,267	9.03 (9.02-9.05)	9.17 (9.15-9.18)	9.22 (9.20-9.25)	15,374 (12,887-17,860)	4,473 (2,057-6,889)	19,847 (16,379-23,314)
Cancers	Leukemia	10,940	22,232	11,429	4.98 (4.91-5.05)	5.47 (5.42-5.52)	5.89 (5.83-5.96)	9,936 (8,889-10,982)	9,359 (7,558-11,161)	19,295 (17,212-21,379)
Digestive diseases	Vascular intestinal	9,489	15,623	6,318	4.88 (4.80-4.96)	5.50 (5.43-5.56)	6.03 (5.93-6.12)	10,839 (9,658-12,020)	8,268 (6,496-10,039)	19,106 (16,977-21,235)
Cancers	Lymphoma	12,502	25,488	12,954	5.85 (5.78-5.91)	6.30 (6.26-6.35)	6.64 (6.58-6.70)	9,890 (8,802-10,977)	8,486 (6,595-10,377)	18,376 (16,194-20,557)
Cancers	Lip oral cavity cancer	7,964	14,593	7,243	5.98 (5.90-6.07)	6.85 (6.79-6.91)	7.29 (7.20-7.36)	10,367 (9,460-11,274)	6,307 (4,871-7,743)	16,674 (14,976-18,373)
Neurological disorders	Parkinson's disease	15,589	32,278	17,868	6.03 (5.98-6.08)	6.36 (6.32-6.39)	6.58 (6.53-6.62)	8,552 (7,447-9,658)	7,030 (5,124-8,935)	15,582 (13,379-17,785)
Cardiovascular diseases	Rheumatic heart disease	9,114	15,165	7,334	5.30 (5.22-5.39)	5.79 (5.74-5.86)	6.25 (6.16-6.33)	8,600 (7,530-9,671)	6,930 (5,368-8,492)	15,530 (13,637-17,424)
Mental disorders & substance use	Self-harm	56,405	61,345	18,365	8.89 (8.87-8.91)	9.02 (9.00-9.04)	9.08 (9.05-9.11)	10,691 (8,729-12,652)	3,696 (1,602-5,790)	14,387 (11,518-17,256)
Cancers	Kidney cancer	10,677	19,931	9,025	5.28 (5.20-5.35)	5.77 (5.72-5.82)	6.04 (5.96-6.12)	8,182 (7,049-9,315)	5,350 (3,465-7,236)	13,532 (11,333-15,732)
Cancers	Uterine cancer	10,258	19,493	9,621	6.96 (6.90-7.03)	7.35 (7.30-7.40)	7.66 (7.60-7.72)	7,121 (6,185-8,057)	5,935 (4,395-7,476)	13,056 (11,254-14,859)
Cardiovascular diseases	Endocarditis	7,292	10,868	4,886	4.91 (4.83-4.99)	5.64 (5.57-5.71)	6.03 (5.93-6.13)	8,205 (7,265-9,144)	4,259 (2,944-5,573)	12,463 (10,847-14,079)
Mental disorders & substance use	Bipolar disorder	38,126	50,320	16,614	9.28 (9.26-9.30)	9.43 (9.41-9.44)	9.50 (9.48-9.52)	8,467 (7,452-9,482)	3,814 (2,593-5,036)	12,281 (10,693-13,870)
Cancers	Myeloma	6,957	14,201	7,974	4.98 (4.89-5.06)	5.41 (5.35-5.47)	5.84 (5.76-5.93)	6,025 (5,209-6,841)	6,223 (4,775-7,671)	12,248 (10,586-13,910)
Cancers	Pancreatic cancer	12,051	22,981	10,982	1.35 (1.31-1.39)	1.59 (1.56-1.62)	1.85 (1.80-1.91)	6,011 (5,176-6,847)	5,958 (4,466-7,450)	11,969 (10,259-13,679)
Mental disorders & substance use	ADHD	73,785	95,590	29,216	9.79 (9.78-9.80)	9.87 (9.86-9.88)	9.90 (9.89-9.91)	8,268 (7,067-9,469)	2,662 (1,326-3,998)	10,930 (9,133-12,726)
Mental disorders & substance use	Schizophrenia	25,512	13,059	2,549	8.72 (8.69-8.74)	8.92 (8.89-8.96)	9.05 (8.97-9.12)	8,506 (6,454-10,558)	1,658 (558-2,757)	10,163 (7,835-12,491)
Cancers	Other pharynx cancer	3,318	5,017	2,363	5.21 (5.06-5.34)	6.45 (6.34-6.55)	7.11 (6.97-7.25)	6,329 (5,683-6,974)	3,327 (2,443-4,211)	9,656 (8,561-10,750)
Other	Tuberculosis	19,090	15,264	6,345	8.59 (8.55-8.62)	8.75 (8.72-8.78)	8.92 (8.88-8.96)	6,299 (5,314-7,285)	2,648 (1,910-3,385)	8,947 (7,716-10,178)
Cancers	Ovarian cancer	6,525	13,448	6,825	5.10 (5.01-5.19)	5.29 (5.23-5.36)	5.63 (5.54-5.73)	3,471 (2,648-4,294)	4,594 (3,132-6,055)	8,064 (6,387-9,742)
Cancers	Stomach cancer	7,407	13,113	5,527	2.72 (2.65-2.80)	3.04 (2.98-3.10)	3.30 (3.21-3.40)	4,273 (3,376-5,170)	3,478 (2,000-4,957)	7,751 (6,022-9,481)
Other	HIV/AIDS	12,162	20,586	10,923	8.98 (8.94-9.02)	9.14 (9.11-9.17)	9.31 (9.28-9.35)	4,084 (3,446-4,723)	3,609 (2,723-4,494)	7,693 (6,601-8,784)
Cancers	Soft tissue and other extraosseous sarcomas	4,359	9,111	5,066	5.55 (5.44-5.67)	5.96 (5.87-6.03)	6.34 (6.23-6.44)	3,430 (2,748-4,113)	3,483 (2,254-4,713)	6,914 (5,507-8,320)
Cardiovascular diseases	PAH	4,219	6,691	2,512	3.26 (3.16-3.36)	3.76 (3.68-3.85)	4.13 (3.98-4.27)	3,690 (2,954-4,426)	2,483 (1,376-3,590)	6,173 (4,843-7,503)
Cancers	Esophageal cancer	4,616	7,516	3,100	1.88 (1.80-1.96)	2.29 (2.22-2.36)	2.60 (2.49-2.72)	3,328 (2,664-3,991)	2,341 (1,289-3,392)	5,668 (4,425-6,912)
Cancers	Brain cancer	6,835	14,251	7,173	3.15 (3.08-3.23)	3.30 (3.24-3.35)	3.46 (3.39-3.55)	2,131 (1,371-2,891)	2,396 (1,022-3,769)	4,527 (2,957-6,097)
Cancers	Larynx cancer	2,102	2,583	1,017	5.31 (5.15-5.47)	6.28 (6.13-6.42)	6.77 (6.55-6.99)	3,071 (2,505-3,636)	1,271 (596-1,945)	4,341 (3,461-5,222)
Cancers	Gallbladder cancer	4,743	8,629	3,827	1.59 (1.52-1.67)	1.91 (1.85-1.97)	2.12 (2.02-2.22)	2,503 (1,917-3,089)	1,826 (815-2,838)	4,329 (3,160-5,498)
Cancers	Liver cancer	5,732	7,412	3,096	2.25 (2.17-2.33)	2.40 (2.32-2.47)	2.65 (2.53-2.79)	2,298 (1,401-3,196)	1,905 (771-3,039)	4,204 (2,757-5,650)
Cancers	Cervical cancer	4,334	7,701	3,560	6.97 (6.88-7.06)	7.41 (7.34-7.48)	7.55 (7.45-7.66)	2,524 (1,924-3,124)	1,101 (126-2,076)	3,625 (2,480-4,770)
Musculoskeletal disorders	Lupus	4,739	8,635	4,053	8.80 (8.73-8.86)	8.96 (8.91-9.00)	9.14 (9.08-9.19)	1,603 (1,216-1,991)	1,566 (968-2,164)	3,169 (2,457-3,882)
Cancers	Thyroid cancer	3,862	7,178	3,550	7.97 (7.87-8.06)	8.21 (8.14-8.27)	8.41 (8.33-8.50)	1,690 (1,193-2,188)	1,479 (697-2,262)	3,169 (2,242-4,097)
Neurological disorders	Multiple sclerosis	6,794	14,056	6,993	9.38 (9.35-9.42)	9.50 (9.48-9.53)	9.60 (9.57-9.63)	1,468 (1,150-1,786)	1,378 (854-1,902)	2,846 (2,233-3,459)
Cancers	Malignant neoplasm of bone & articular cartilage	2,052	3,893	1,887	4.17 (4.02-4.32)	4.67 (4.55-4.79)	4.93 (4.76-5.10)	1,559 (1,101-2,017)	1,001 (203-1,798)	2,560 (1,640-3,479)
Neurological disorders	Motor neuron disease	2,400	5,144	2,652	3.03 (2.92-3.15)	3.41 (3.32-3.50)	3.58 (3.46-3.70)	1,315 (912-1,718)	870 (97-1,643)	2,185 (1,313-3,057)
Chronic respiratory	Pneumoconiosis	1,953	4,632	1,400	5.83 (5.70-5.97)	6.20 (6.11-6.29)	6.41 (6.25-6.55)	1,119 (713-1,525)	968 (140-1,796)	2,087 (1,165-3,009)
Cancers	Hodgkin lymphoma	2,104	4,071	2,171	7.58 (7.46-7.70)	7.79 (7.70-7.87)	8.00 (7.88-8.11)	871 (527-1,214)	833 (253-1,414)	1,704 (1,030-2,379)
Cancers	Testicular cancer	2,136	5,337	2,840	9.04 (8.96-9.12)	9.29 (9.23-9.33)	9.36 (9.29-9.42)	673 (448-898)	NS	673 (448-898)
Mental disorders & substance use	Anorexia nervosa	3,857	8,783	4,752	9.73 (9.69-9.77)	9.78 (9.75-9.81)	9.84 (9.80-9.87)	403 (195-610)	474 (66-882)	877 (419-1,334)
Diabetes & CKD	Acute glomerulonephritis	1,066	2,102	1,077	8.16 (8.01-8.31)	8.57 (8.46-8.66)	8.68 (8.53-8.82)	550 (323-778)	NS	550 (323-778)
Cancers	Nasopharynx cancer	559	832	337	5.58 (5.29-5.86)	6.25 (6.01-6.48)	6.16 (5.79-6.53)	328 (68-589)	NS	328 (68-589)
	**TOTAL**	**6,111,010**	**10,405,971**	**4,610,572**	—	—	—	**3,528,339 (3,379,153-3,677,525)**	**2,745,899 (2,526,214-2,965,584)**	**6,274,238 (5,905,367-6,643,109)**
