# Supplementary material for: Income-related differences in survival across disease categories in Sweden: a retrospective population-based register study
Source: Lancet Reg Health Eur. 2026 Oct 15;71:101896. doi: 10.1016/j.lanepe.2026.101896 (PMC13694925; doi:10.1016/j.lanepe.2026.101896)
Supplement: Supplementary file 2 [file mmc2.docx]

### Appendix:

**Supplementary Methods Appendix**

Contents:

A. Diagnosis records and disease classification
B. The disposable family income measure
C. The method for creating matched control cohorts
D. Analysis and modelling strategy
E. Model validations and robustness checks
F. STROBE statement

### Figures

**Appendix Figure A1: Scatter of Q1 vs Q4 10-year Survival Probabilities.** Scatter plot displaying Q1 (lowest income) survival on the y axis and Q4 (highest income) survival on the x axis. Points are sized based on the total number of diagnosed individuals and colored based on the ratio of low-income to high-income diagnosed individuals.

### Tables

**Appendix Table A1: ICD Code Mapping for Disease Categories.** Complete listing of ICD-10 codes used to define each of the 100 disease categories, including diagnostic codes and any relevant history codes, organized by disease group.

**Appendix Table A2: Comorbidity Crosstabulation.** Distribution of the number of distinct disease groups in which patients received at least one diagnosis, stratified by overall income quartile. Income quartile reflects each patient's modal disposable income quartile across all observed calendar years (2001–2023), with ties assigned to the lower quartile.

**Appendix Table A3: Summary Statistics by Income Quartile.** Descriptive statistics for each of the 100 disease categories by income quartile, including sample size, proportion female, mean age at diagnosis, Kaplan-Meier 10-year survival probability, median survival time, and 10-year restricted mean survival time (RMST).

**Appendix Table A4: Risk Ratios for Diagnosed vs. Matched Undiagnosed Controls.** Comparison of 10-year survival probabilities between individuals with an incident diagnosis and their matched undiagnosed controls, expressed as mortality risk ratios, for each disease category.

**Appendix Table A5: Unadjusted Median Survival and Survival Probabilities by Age, both sexes.** Median survival and survival probabilities from Kaplan-Meier estimator for specific age ranges 16-24, 25-34, 35-44, 45-54, 55-64, 65-74, 75-84, 85-95

**Appendix Table A6: Unadjusted Median Survival and Survival Probabilities by Age, males.** Median survival and survival probabilities from Kaplan-Meier estimator for specific age ranges 16-24, 25-34, 35-44, 45-54, 55-64, 65-74, 75-84, 85-95

**Appendix Table A7: Unadjusted Median Survival and Survival Probabilities by Age, females.** Median survival and survival probabilities from Kaplan-Meier estimator for specific age ranges 16-24, 25-34, 35-44, 45-54, 55-64, 65-74, 75-84, 85-95

**Appendix Table A8: Person-Years of Survival Lost: Low (Q1) and Middle (Q2 + Q3) Income vs High Income.** Estimated person-years of survival lost among low-income (Q1) and middle income (Q2 + Q3) individuals compared to the counterfactual scenario where these groups experienced high-income (Q4) survival patterns, calculated as the difference in 10-year RMST multiplied by the number of individuals in each group, for each disease category (excluding subcauses).
